# Commercial NIRS May Not Detect Hemispheric Regional Disparity in Continuously Measured COx/COx-a: An Exploratory Healthy and Cranial Trauma Time-Series Analysis

**DOI:** 10.3390/bioengineering12030247

**Published:** 2025-02-28

**Authors:** Amanjyot Singh Sainbhi, Logan Froese, Kevin Y. Stein, Nuray Vakitbilir, Alwyn Gomez, Abrar Islam, Tobias Bergmann, Noah Silvaggio, Mansoor Hayat, Frederick A. Zeiler

**Affiliations:** 1 Department of Biomedical Engineering, Price Faculty of Engineering, University of Manitoba, Winnipeg, MB R3T 5V6, Canada; steink34@myumanitoba.ca (K.Y.S.); vakitbir@myumanitoba.ca (N.V.); islama9@myumanitoba.ca (A.I.); bergmant@myumanitoba.ca (T.B.); 2 Department of Clinical Neurosciences, Karolinska Institutet, 171 77 Stockholm, Sweden; logan.froese@ki.se; 3 Undergraduate Medicine, Rady Faculty of Health Sciences, University of Manitoba, Winnipeg, MB R3E 3P5, Canada; 4 Section of Neurosurgery, Department of Surgery, Rady Faculty of Health Sciences, University of Manitoba, Winnipeg, MB R3A 1R9, Canada; gomeza35@myumanitoba.ca (A.G.); mansoor.hayat@umanitoba.ca (M.H.); 5 Department of Human Anatomy and Cell Science, Rady Faculty of Health Sciences, University of Manitoba, Winnipeg, MB R3E 0J9, Canada; silvaggn@myumanitoba.ca; 6 Pan Am Clinic Foundation, Winnipeg, MB R3M 3E4, Canada; 7 Division of Anaesthesia, Department of Medicine, Addenbrooke’s Hospital, University of Cambridge, Cambridge CB2 0QQ, UK

**Keywords:** autocorrelative structure, cerebral autoregulation, cerebral oximetry index, nirs, regional disparity, regional cerebral oxygen saturation, time series

## Abstract

Continuous metrics of cerebral autoregulation (CA) assessment have been developed using various multimodal cerebral physiological monitoring devices. However, CA regional disparity remains unclear in states of health and disease. Leveraging existing archived data sources, we preliminarily evaluated regional hemispheric disparity in CA using the near infrared spectroscopy (NIRS)-derived cerebral oximetry index (COx/COx-a). Along with bilateral NIRS, regional cerebral oxygen saturation, arterial blood pressure, cerebral perfusion pressure, and bilateral COx/COx-a were derived using three different temporal resolutions—10 s, 1 min, and 5 min—based on non-overlapping mean values. The regional disparity between hemispheres was evaluated based on median and median absolute deviation. Further, patient-level autoregressive integrative moving average models were calculated for each signal stream and used to generate personalized vector autoregressive models. Multi-variate cerebral physiologic relationships between hemispheres were assessed via impulse response functions and Granger causality analyses. Data from 102 healthy control volunteers, 27 spinal surgery patients, and 95 TBI patients (varying in frontal lobe pathology impacting the optode path; 64 without bifrontal lobe pathology, 15 without left frontal lobe pathology, 11 without right frontal lobe pathology, and 5 with bifrontal lobe pathology) were retrospectively analyzed. For subjects with or without cranial pathology, no difference in COx/COx-a was found between hemispheres regardless of the analytic method. In TBI patients without pathology underneath the NIRS sensor, distant parenchymal injury does not seem to have an effect on the CA of uninjured frontal lobes. Further work is required to characterize regional disparities with multi-channel CA measurements in healthy and disease states.

## 1. Introduction

The ability of cerebral blood vessels to maintain a relatively constant cerebral blood flow (CBF) over a wide range of systemic arterial blood pressure (ABP) is termed cerebral autoregulation (CA) [1,2]. By constriction and dilation of cerebral blood vessels, a constant CBF is maintained by the mechanism referred to as cerebrovascular reactivity (CVR) [1,2]. CVR is a broader term for CA, which describes the physiologic process, but they are not entirely interchangeable because CVR can occur outside the limits of CA. Various neuropathological states such as traumatic brain injury (TBI) have documented impairments in CA [3,4,5,6,7,8,9], which can shift the lower and upper limits of autoregulation (LLA and ULA, respectively) as well as narrow the CBF plateau region between them [1,2,10]. Hence, the literature suggests that exposure to impaired CA in various neurological conditions (i.e., hypoperfusion or hyperperfusion) is a significant driver of poor long-term outcomes [5,6,8,11,12,13]. The continuous monitoring of CA at the patient’s bedside has a potential, at early detection and future intervention, to help prevent prolonged states of pressure passive flow.

Indirect measurements of CA at the bedside can be achieved using CVR metrics, which are surrogate measures of CA [3,14,15], and evaluate the relationship between the slow vasogenic fluctuations in cerebral perfusion pressure (CPP) or ABP and a surrogate for pulsatile CBF or cerebral blood volume (CBv [16]). Time-domain CVR metrics are more readily studied over the frequency-domain CVR metrics due to their widespread adoption by clinicians at the bedside and are derived as moving Pearson correlation coefficients between slow-wave (i.e., 0.05–0.005 Hz) fluctuations [17,18] in driving pressure (CPP/ABP) and pulsatile CBF/CBv surrogate [19,20,21,22,23,24,25,26,27]. Currently, the pressure reactivity index (PRx, correlation between intracranial pressure [ICP] and ABP) is the most established invasive method for continuous assessment of CVR in the time domain [28,29,30]. However, PRx requires invasive ICP monitoring to derive and typically can only provide a single region assessment, which is used as a global assessment of CVR. It has been suggested from the neuroimaging and transcranial Doppler literature that regional disparity in CBF and CA/CVR exists in different healthy and pathologic states. As such, PRx is limited in its utility for comprehensive assessments of regional disparities in CA/CVR.

Recent advances in non-invasive cerebral physiologic monitoring have leveraged near infrared spectroscopy (NIRS) to obtain a bifrontal assessment of regional cerebral oxygen saturations (rSO_2_). These rSO_2_ values can be leveraged to derive the NIRS-based CVR index called the cerebral oximetry index, which is the correlation index between NIRS-based rSO_2_ and CPP/ABP (COx/COx-a, respectively) [19,20,22,23,24,25,26,31,32,33,34]. Both ICP and NIRS-based CVR metrics have been shown to be able to measure aspects of the LLA in the pre-clinical literature. Also, high-frequency NIRS has been shown to identify executive dysfunction in neurocognitive disorder patients after TBI using the detection of hemoglobin levels in the bilateral frontopolar region [35]. NIRS-based CVR carries two important benefits over standard ICP-based CVR monitoring. First, it can be derived entirely non-invasively, using both NIRS and non-invasive ABP monitoring systems (only COx-a). This widens its potential application to many populations where invasive CVR monitoring would otherwise not be possible. Second, NIRS can easily be applied in a multi-channel capacity, overcoming the spatial resolution issues associated with PRx, and can provide, in theory, information on regional disparity in CA/CVR.

However, to date, the literature surrounding the ability of commercially available cerebral NIRS systems, with low sampling rate, to characterize CA/CVR regional disparity is limited, with most systems employed in healthcare settings limited to bilateral (i.e., hemispheric) assessments. A previous scoping review by our group has shown that, in both health and disease, varying results have been found in the current human literature that assessed regional disparity using a NIRS rSO_2_-based CVR index [36]. It remains unknown if commercially available NIRS systems with lower resolution applied in healthcare settings carry the ability to discern regional or hemispheric differences in CA/CVR. The goal of this retrospective database study is to explore the ability of a commercially available cerebral NIRS system to detect regional/hemispheric disparity in CA/CVR, as assessed through the COx and COx-a CVR metrics, in healthy and cranial trauma populations. We hypothesize that signals from the commercially available NIRS system would be able to provide an indication of regional or hemispheric differences in CA/CVR in the cranial trauma population versus healthy groups who were not suffering from any neurological illness.

## 2. Materials and Methods

### 2.1. Study Design and Human Populations

High-resolution cerebral physiologic data were retrospectively accessed from the collection of multimodal monitoring physiology at the University of Manitoba Multi-omic Analytics and Integrative Neuroinformatics in the HUman Brain (MAIN-HUB) Lab, similar to previous works from our group [37,38,39]. Three separate populations of archived data were accessed for this study, including A. healthy volunteers [38,40], B. elective spinal surgery patients [38], and C. TBI patients [41,42] (hereon denoted as HC, SP, and TBI, respectively). These three populations were accessed to provide comparisons between those in normal states (i.e., HC), normal states under anesthesia (i.e., SP), and neuropathological states (i.e., TBI).

The HC group consisted of volunteers who were over the age of 18 and had no personal history of any neurological or cardiovascular conditions. They participated in having bifrontal NIRS and non-invasive ABP data recorded for 30 mins in an awake sitting state.

The SP group consisted of adults, age 18 years or older, who underwent elective spinal surgery (no intradural work) with bifrontal NIRS and invasive ABP data collected throughout their procedure. Also, they were exclusively sedated with intravenous anesthetics to a depth of general anesthesia.

The TBI cohort included adult patients (age 18 years or older) with moderate/severe TBI, admitted to the Health Sciences Centre intensive care unit (ICU; Shared Health Manitoba) between the years of 2017 and 2023. Invasive ICP monitoring, care provision, and sedation administration were in line with existing standard Brain Trauma Foundation (BTF) guidelines [43]. Such patients had ICP, invasive ABP, and NIRS data collected during their acute phase of care in the ICU. The TBI patients were further subdivided into four groups based on underlying pathology: without bifrontal lobe pathology (TBI-GLR), without left frontal lobe pathology (TBI-GL), without right frontal lobe pathology (TBI-GR), and with bifrontal lobe pathology (TBI-BLR). The presence of pathology was defined as underlaying extra-axial or intra-axial lesions on admission computed tomography (CT) of the brain.

### 2.2. Ethical Consideration

Data were collected following full approval by the University of Manitoba Health Research Ethics Board (TBI = H2017:181, H2017:188, B2018:103, B2023:001, H2024:266; HC = B2020:019; SP = B2020:104) and the Shared Health/Health Sciences Centre Research Impact Committee.

### 2.3. Data Collection

For all three populations, high-frequency ABP data, sampled at 100 Hz, along with left and right rSO_2_ (rSO_2__L and rSO_2__R, respectively) data, sampled at 1 Hz, were collected. For the HC group, ABP was non-invasively collected using the Finapres Nova finger cuff (Finapres Medical Systems, Enschede, The Netherlands). For the SP group, ABP was invasively obtained using arterial lines connected to pressure transducers zeroed at the level of the tragus (Baxter Healthcare Corp. CardioVascular Group, Irvine, CA, USA). For the TBI group, ABP was captured using radial arterial lines, ICP was invasively measured using either intra-parenchymal strain gauge probes (Codman ICP MicroSensor; Codman & Shurtlef Inc., Raynham, MA, USA) placed in the frontal lobe or external ventricular drains (n = 4) (Medtronic, Minneapolis, MN, USA), and CPP was derived as the arithmetic difference between mean ABP and ICP. For all groups, the rSO_2__L and rSO_2__R were determined with NIRS regional oximetry monitoring using bilateral disposable adhesive pads on the left and right forehead (with transmitting and receiving optodes at 180 degrees), superficial to the frontal lobes. This device uses a proprietary algorithm to calculate the rSO_2_ value from relative amounts of deoxy-hemoglobin and total hemoglobin in a spatially resolved manner to avoid superficial extracranial tissue contamination (Covidien, INVOS 5100C or 7100, Troy, MI, USA).

All physiological data were recorded and digitized in high-frequency resolution (up to 100 Hz where available) using Intensive Care Monitoring “Plus” (ICM+) data acquisition software (Version 8.5, Cambridge Enterprise Ltd., Cambridge, UK, https://icmplus.neurosurg.cam.ac.uk/, accessed on 31 August 2023), and analog-to-digital signal converters (Data Translations, DT9804 or DT9826) where needed. ABP in all populations and CPP in the TBI population were recorded at a 100 Hz sampling rate, while the NIRS signals were recorded at a 1 Hz sampling rate and subsequently up-sampled.

The patient demographic data were extracted for cohort characterization and included duration of recording, age, biological sex, hand dominance, procedure type, anesthetic regimen, admission Glasgow Coma Scale (GCS; total and motor sub-score), hypoxia, hypotension, arterial partial pressure of carbon dioxide (pCO_2_), arterial partial pressure of oxygen (pO_2_), procedure type, admission pupillary response, Marshall CT score, and Rotterdam CT score.

### 2.4. Physiologic Data Cleaning and Processing

The recorded high-resolution physiologic data were cleaned and processed afterwards using ICM+ software. All signal artifacts were removed from the data streams using manual methods by qualified personnel who looked at each patient recording and removed artifacts over the full length of the recording, similar to past work by our lab [37,38,39]. For each patient, the ABP, rSO_2__L, and rSO_2__R (ICP and CPP as well for TBI patients) were decimated using non-overlapping moving average filters of 10 s duration to focus on the slow-wave vasogenic fluctuations associated with CA [14,17,18,44]. Then, moving Pearson correlation coefficients were calculated using 30 consecutive 10 s mean windows (i.e., five minutes of data), updated every 10 s, to derive the following rSO_2_-based CVR index for both sides using ABP [25,45,46,47]: left cerebral oximetry index with ABP (COx-a_L–correlation between rSO_2__L and ABP) and right cerebral oximetry index with ABP (COx-a_R–correlation between rSO_2__R and ABP). For TBI patients, an additional rSO_2_-based CVR index for both sides using CPP was derived: left cerebral oximetry index with CPP (COx_L–correlation between rSO_2__L and CPP) and right cerebral oximetry index with CPP (COx_R–correlation between rSO_2__R and CPP). These CVR indices have a range from −1 to +1 since they were computed using Pearson correlation coefficients, with higher values (closer to +1) indicating impaired CVR and values below the range of 0 to +0.4 indicating intact CVR [45,48,49].

All the data were obtained in the highest temporal resolution of 10 s, and it was also reduced to 1 min and 5 min resolutions (using non-overlapping moving average filters). The lowest 5 min resolution was chosen to corroborate results from higher temporal resolutions since bias may have been introduced in the data of higher temporal resolutions because the output frequency of the calculated Pearson correlation coefficients was less than its window size (i.e., five minutes of data). The analysis, discussed in the next section, was performed using all three data resolutions. The reduction in temporal resolution was achieved with a custom script developed in R statistical software (Version 4.3.1, R Foundation for Statistical Computing, Vienna, Austria, https://www.r-project.org/, accessed on 31 August 2023), which applied a non-overlapping moving average to each physiologic signal column of the 10 s temporal resolution data for a patient. The non-overlapping moving average was performed using the “running” function from the gtools package (Version 3.9.5, https://cran.r-project.org/web/packages/gtools/index.html, accessed on 20 November 2023).

### 2.5. Statistical Data Analysis

All statistical analyses were performed in R statistical software with alpha (α) set at 0.05 for statistical significance with no corrections for multiple comparisons due to the exploratory nature of this study. General data distributions were summarized using median, interquartile range (IQR), number, and percentage, where appropriate. The overall analysis of hemispheric disparities (comparing left to right side in the three populations) encompassed the following:

(A) Median and variance differences (using entire recording periods);
(B) Absolute regional hemispheric differences between left and right (in 10 s, 1 min, and 5 min temporal resolutions);
(C) Time-series autocorrelative structure differences for rSO_2_ and COx/COx-a (in 10 s, 1 min, and 5 min temporal resolutions);
(D) Time-series impulse response function differences for the ABP/CPP on rSO_2_ relationships (in 10 s, 1 min, and 5 min temporal resolutions);
(E) The difference in causal relationships between ABP/CPP and rSO_2_ (in 10 s, 1 min, and 5 min temporal resolutions);
(F) In the sub-sections to follow, the individual methods will be briefly covered.

#### 2.5.1. General Statistical Evaluation–Median Hemispheric Values

The median, IQR, and median absolute deviation (MAD) were calculated for each signal source (rSO_2_, COx, and COx-a) for both the left and right sides over the entire recording period of each subject in each population. Similarly, percent time over various literature-defined threshold values was calculated for bilateral rSO_2_, COx, and COx-a signals as follows: percent time of bilateral rSO_2_ above 60%, 70%, 80%, and 90%; and percent time of bilateral COx/COx-a above 0, +0.2, and +0.3. Finally, a comparison between the left and right hemispheres was conducted on these recording-based summary metrics using the Wilcoxon Rank Sum test (Mann–Whitney U testing).

#### 2.5.2. Absolute Regional Hemispheric Difference—10 s, 1 min, and 5 min Data

The regional hemispheric disparity was determined in individual patients from all three populations using the left and right side data of original rSO_2_, COx (where available), and COx-a signals in 10 s, 1 min, and 5 min data resolutions. With a custom script developed in R statistical software, the absolute value of the hemispheric difference in a signal was calculated for each patient using the signal’s left and right data to determine the absolute regional hemispheric difference (ARHD). The ARHD is able to provide a quantifiable magnitude of the difference in a signal between the hemispheres. We were able to derive the MAD of the ARHDs for the entire population and sub-group analysis by finding the median of the absolute deviation of each ARHD value to the median of ARHD.

#### 2.5.3. Personalized Time-Series Structures of rSO_2_, COx, and COx-a Signals—10 s, 1 min, and 5 min Data

General methods for personalized time-series autoregressive integrative moving average (ARIMA) structures are described in File S1a and S1b and our previous work on the subject [39,50]. In brief, data stationarity and personalized autoregressive order (p-order) and moving average order (q-order) were determined for rSO_2_, COx, and COx-a signal sources in all three data resolutions, in all three human populations, using standard Box–Jenkin’s methodologies while the integrative order (d-order) was held at 1. Stationarity analysis was performed for each physiologic signal at an individual level using Augmented Dickey–Fuller (ADF) and Kwiatkowski–Phillips–Schmidt–Shin (KPSS) tests to make sure the data were truly stationary, which is required for linear modeling, after it was detrended as it has been performed in our previous work [39]. Personalized ARIMA model orders were determined using the lowest Akaike Information Criterion (AIC) values for the derived ARIMA models. A comparison of the differences between hemispheres in this time-series structure was conducted qualitatively, with median personalized structures tabulated for all temporal resolutions and populations for each signal.

#### 2.5.4. Time-Series Impulse Response Function (IRF) for ABP/CPP and rSO_2_—10 s, 1 min, 5 min Data

A general description of vector autoregressive (VAR) models can be found in File S1c and our previous work in the area [37,50,51,52]. In brief, VAR models were derived to evaluate for hemispheric differences in the multi-variate relationship between ABP/CPP and rSO_2_ (i.e., the constituents of NIRS-based CVR indices COx and COx-a). The autoregressive order (p-order) for the VAR model was calculated by taking the product of the personalized ARIMA p-orders of the two signals being evaluated, as suggested in the past literature [53]. IRF coefficients of the VAR model were generated using the personalized VAR p-order and VAR p-order cap for the normalized first-order-differenced data in all three temporal resolutions. Using Bootstrap, the confidence interval was derived from 100 runs, and all this information can be graphically represented to verify the interactions between each signal pair of a patient. The responsiveness of various models was checked to see if it was greater than an absolute value of 0.001 threshold. This threshold was chosen to look at a change in the normalized response of at least 0.1% in the majority of five lags after lag 10 (lags 11 to 15). In each population, the number of patients showing a greater response than 0.1% was counted for each directionality relationship between two signals, and it was used to compare the cerebral physiologic relationships between hemispheres.

#### 2.5.5. Differences in Granger Causality Relationships between ABP/CPP and rSO_2_—10 s, 1 min, 5 min Data

To evaluate the interdependence between ABP/CPP and rSO_2_ (i.e., the constituents of NIRS-based CA/CVR indices COx and COx-a) and any hemispheric disparities, we leveraged Granger causality testing. Granger causality testing was used to assess the ability of one signal to predict another signal beyond the ability of the signal to predict itself. Using the first-order-differenced data in 10 s, 1 min, and 5 min data resolutions, the responses of Granger causality tests, both F-test statistic value and *p*-values [54], were recorded. The identification of reciprocal influences between two signals was assessed from these responses, and the cerebral physiologic relationships between hemispheres were compared.

#### 2.5.6. Sub-Group Analysis

All the above aspects of analysis for the hemispheric disparity in rSO_2_, COx, and COx-a were also conducted in various sub-groups for the HC, SP, and TBI populations. The common parameters included for all groups were age (<60 vs. ≥60; moderate vs. old age) and biological sex (male vs. female). Further sub-group analysis in the HC group was hand dominance (left-hand vs. right-hand dominance). The SP group was sub-analyzed based on procedure type and anesthetic regimen. Finally, the TBI group was sub-analyzed based on injury pattern/severity and sedation regimen. File S1d lists the sub-group parameters in detail.

## 3. Results

### 3.1. Population Demographics

A total of 102 HC, 27 SP, and 95 TBI patients were included in this study. The TBI patients were further subdivided into a total of 64 TBI-GLR, 15 TBI-GL, 11 TBI-GR, and 5 TBI-BLR. The median recording duration was 31.1 min (IQR: 28.7–34.8 min) for HC, 185 min (IQR: 165.8–206.8 min) for SP, 4586.8 min (IQR: 2523.7–7685 min) for TBI-GLR, 5305 min (IQR: 3196.8–10,707.7 min) for TBI-GL, 3245.5 min (IQR: 2379.4–5993.6 min) for TBI-GR, and 9685.5 min (IQR: 7380.7–14,308.2 min) for TBI-BLR. Table 1 shows the summary of patient admission demographics and injury information data for HC, SP, and TBI-GLR populations, and File S2 shows this information for TBI-GL, TBI-GR, and TBI-BLR populations.

### 3.2. Median Entire Recording Summary Metrics–Hemispheric Differences

Various population physiologic variables were looked at, including the ABP, CPP, rSO_2__L, rSO_2__R, COx_L, COx_R, COx-a_L, and COx-a_R, their MAD values, and performing Mann–Whitney U test between the bilateral signals. The percent time of bilateral rSO_2_ above 60%, 70%, 80%, and 90%, and the percent time of bilateral COx and COx-a above 0, +0.2, and +0.3 were also calculated along with performing the Mann–Whitney U test between the bilateral percent time of a signal. In general, across all data resolutions, no significant *p*-values were reported for all populations between left and right rSO_2_, COx, and COx-a, along with the MAD of these signals and the calculated percent time these signals were above the reported thresholds. The only significant *p*-value reported between the left and right sides was for the percent time of rSO_2_ above 70% in the HC 1 min data (*p* = 0.0306). The summary of the patient physiology results with Mann–Whitney U test *p*-values using 10 s data resolution are given in Table 2 for HC, SP, and TBI-GLR, while File S3 shows similar results using 1 min and 5 min data resolutions and other sub-groups of TBI patients.

### 3.3. Absolute Regional Hemispheric Differences (ARHD)—10 s, 1 min, 5 min Data

Overall, across all temporal resolutions, the ARHD and MAD were minimal between hemispheres for rSO_2_. However, for COx and COx-a, the ARHD and MAD were of a magnitude that may be clinically relevant. The regional hemispheric disparity analysis on 10 s data resolution is given in Table 3, and similarly, the regional hemispheric disparity analyses at 1 min and 5 min data resolutions are given in File S4 along with analyses for other sub-groups of TBI.

### 3.4. Hemispheric Difference in Personalized ARIMA Structure for rSO_2,_ COx and COx-a—10 s, 1 min, 5 min Data

Overall, across all three populations and temporal resolutions, there was no significant difference in rSO_2_, COx, or COx-a signal stationarity between left and right hemispheres, with all signals demonstrating a similar level of non-stationary behavior on ADF and KPSS testing, requiring first-order differencing to mitigate. Table 4 provides the general results for ADF and KPSS testing on raw and first-order-differenced COx and COx-a data streams, while File S5 provides such analyses on the remaining signals and sub-groups. Note in these tables/appendices, NA represents when stationarity could not be determined for some patients due to inadequate data points.

Similarly, there was no significant difference between the left and right hemispheres for median personalized ARIMA orders, regardless of population or data temporal resolution. However, when evaluating the ARHD in personalized ARIMA orders, there was a slight difference of ~2 seen for the TBI patients, suggesting the potential of NIRS to discriminate hemispheric disparity based on time-series statistical structures of the rSO_2_, Cox, and COx-a signals. The global median personalized ARIMA orders using 10 s data resolution are given in Table 5, with similar data on the 1 min and 5 min resolutions, as well as TBI sub-groups, found in File S5.

### 3.5. Hemispheric Difference in Impulse Response Function (IRF) of ABP/CPP on rSO_2_—10 s, 1 min, 5 min Data

The responsiveness of bivariate VAR IRF models was checked for variations in hemispheric regional disparity. In general, across all populations and data resolutions tested, there was no significant difference in IRF responses of ABP/CPP on rSO_2_ between the left and right hemispheres. It was found that the percentage of patients showing a greater response than 0.1%, using personalized and capped p-orders, were mostly above 50% of the population for either direction of the following signal combinations using 10 s data resolution: ABP and rSO_2__L, ABP and rSO_2__R, CPP and rSO_2__L, and CPP and rSO_2__R. This % with a response above 0.1% decreased with lower temporal resolution data streams (i.e., 1 min and 5 min). Table 6 provides IRF results for 10 s resolution data for all three populations, with analysis for other data resolutions and sub-groups found in File S6.

### 3.6. Hemispheric Difference in Granger Causality Between ABP/CPP and rSO_2_—10 s, 1 min, 5 min Data

Overall, regardless of data resolution or population tested, there was no significant difference in Granger causality testing results between left and right hemispheres for ABP/CPP on rSO_2_ relationships. A similar directional relationship, favoring ABP/CPP leading to a directional change in rSO_2_, was seen in both left and right hemispheres, regardless of the population, sub-group, or data resolutions. Table 7 provides analysis results for 10 s data resolution, with analyses for other data resolutions and sub-groups found in File S7.

### 3.7. Sub-Group Analysis Assessment

Sub-group analysis across all populations and data resolutions occurred using the sub-group indicators outlined in the Methods (Section 2.5.6), with all results outlined in detail in File S8. Overall, across all categories, minimal differences were discernable between the left and right hemispheres within each individual sub-group analyzed. Only through entire-recording median summary metric evaluations were some weak statistical differences between the left and right hemispheres found for biological sex (HC) and age (HC) for % time above/below rSO_2_ thresholds. ARHD/MAD, time-series ARIMA structures, IRF, and Granger causality analysis failed to conclusively delineate hemispheric disparity in CA/CVR within any of the sub-groups analyzed.

## 4. Discussion

To assess the regional hemispheric disparity in CA/CVR using NIRS-derived COx/COx-a indices, we employed various statistical methods, including A. entire-recording median summary metrics for rSO_2_/COx/COx-a, B. ARHD/MAD of rSO_2_/COx/COx-a data streams, C. time-series personalized ARIMA structures of rSO_2_/COx/COx-a data streams, D. vector IRF analysis of the ABP/CPP on rSO_2_ relationships (i.e., constituents of NIRS-based COx/COx-a indices), and E. Granger causality testing of the ABP/CPP on rSO_2_ relationships (i.e., constituents of NIRS-based COx/COx-a indices) using three different population types and three different data resolution (10 s, 1 min, 5 min). Such an analytic approach represents, to our knowledge, the most comprehensive hemispheric disparity analysis using commercial (i.e., readily available in healthcare settings) cerebral NIRS data streams. Through this exhaustive analysis, some important features regarding low-resolution cerebral NIRS data streams were highlighted.

First, regardless of the performed analysis, population, or data resolutions, current commercially available cerebral NIRS devices that are deployed in healthcare settings may not be able to reliably discern hemispheric or regional disparity. This was demonstrated by a lack of significant differences in left and right hemispheric measures based on grand median summary metrics, time-series structures, IRF, and Granger testing. This was not entirely unexpected for the HC and SP groups, who were not suffering from any neurological illness that would predispose them to regional differences in CA/CVR and is in keeping with the existing literature on the topic [36]. Only ARHD/MAD analysis demonstrated a potential for such NIRS platforms to detect a hemispheric difference in those with moderate/severe biomechanical brain trauma. However, it must be acknowledged that raw values and percent time above/below the threshold for rSO_2_/COx/COx-a did not demonstrate such hemispheric differences, including on sub-group analysis based on injury severity or type. Previous literature reviews have highlighted the potential for spatially resolved CA/CVR techniques to discern hemispheric differences in those with unilateral brain pathology [32,36,55,56,57,58]. Given the lack of significant findings in our study, particularly for the TBI population, this leaves us with the persistent unknown regarding the utility of such commercial systems for spatially resolved CA/CVR analysis and raises the question about high-resolution research-grade NIRS systems for such regional disparity assessments.

Second, this general lack of hemispheric differences across populations and data resolutions held true even for more comprehensive signal statistical structures and modeling that were conducted. There was no hemispheric difference in ARIMA time-series model structures for rSO_2_/COx/COx-a or IRF/Granger directional relationships for the constituent signals of COx/COx-a (i.e., ABP/CPP on rSO_2_ relationship). These findings emphasize that there do not appear to be hidden more complex signal structure disparities in these NIRS signals that could be used for quantifying hemispheric differences. Again, this may be a result of signal resolution from these commercially available systems, further raising the question about research-grade NIRS platforms and their ability to highlight spatial discrepancies.

Third, comprehensive sub-group analysis also failed to demonstrate a reliable capacity for such commercial NIRS signals to discern hemispheric disparities. This is not a surprise necessarily for the HC and SP groups because the lack of hemispheric differences found is in keeping with the established literature, which informs us that there should be minimal to no difference between left and right sides in a CVR index for patients with no cranial trauma [33,36,59]. However, for the TBI population, we expected to demonstrate some more robust results in the ability of such NIRS metrics to highlight hemispheric differences, especially in a neurologic state where regional differences in injury patterns are prevalent. This may be a function of small sample sizes when evaluating such sub-groups or actually represent a true limitation of these commercially available systems. There is varying literature on the regional disparity in the TBI population, while in other cranial trauma such as endarterectomy and subarachnoid hemorrhage, a regional disparity exists [31,32,33,36,60].

Fourth, treatment regimens in the SP and TBI populations, particularly in the area of sedation medications, failed to demonstrate an impact on regional CA/CVR. This is in keeping with the growing established literature body highlighting the limited impact of vasopressor and sedation drugs on various continuously measured CVR indices [51,61,62,63,64,65,66]. However, in our case, we must further acknowledge the low sample size as a potential factor in our negative analysis.

Fifth, the lack of our hemispheric disparity results of the frontal brain region using commercial NIRS, along with the varying results in the TBI literature on regional disparity, could indicate that low-frequency NIRS may not be able to reliably discern regional hemispheric differences. NIRS has potential in the guidance of precision medicine with the support of the current body of literature [19,20,22,23,24,25,26,31,32,33,34,35,67], but a focus comparing commercial (low-frequency) NIRS with research-grade (high-frequency) NIRS is needed for future studies. This will help further investigate the reason for the variance in the current TBI literature on regional disparity and build evidence in support of the use of NIRS in the acute and post-acute phases of injury.

## 5. Limitations

Due to the exploratory nature of the analysis, there are many overarching limitations. First, it was a retrospective analysis of a decent-sized HC population but a relatively small-sized SP population, along with most of the subdivided heterogeneous TBI cohorts having fewer than or equal to 15 patients. Most of the TBI sub-groups had patient numbers of fewer than or equal to 15. As such, our findings should only be considered exploratory and should be taken with caution. Second, these findings may not be generalizable to other HC, SP, or TBI populations and warrant validation in larger multi-center high-frequency physiologic datasets. Third, the commercial NIRS system used had a poor sampling rate of 1 Hz, and these data did not contain pulse waveform information, limiting our analysis to purely time-domain features. Fourth, we were unable to comment on the computation method of the commercial NIRS rSO_2_ signal because a proprietary algorithm was used to calculate this rSO_2_ value. As such, without complete transparency in the NIRS technology, its method for extracranial tissue contamination management and means of oxy/deoxy-hemoglobin and rSO_2_ derivation, the findings of regional disparity in this study need to be taken with caution. As proprietary NIRS algorithms are a common feature of most commercially available NIRS systems, future regional disparity assessments with NIRS would benefit from leveraging the transparency afforded by research-grade functional NIRS (fNIRS) platforms, where there is a clear method to follow the data streams from optical density to derived metrics. Fifth, the recorded spatial resolution was low, which only allowed for the monitoring of the regional hemispheric differences in the frontal lobe of the brain. Sixth, recordings from a single commercial NIRS system were analyzed, which is a potential bias. Seventh, due to the exploratory and retrospective nature of this analysis, we were not able to add additional datasets to boost statistical power. Similarly, to date, there is no universal consensus on the definition of impaired CA or rSO_2_, nor the incidence rates across various populations/pathologies. Thus, we could not prospectively address statistical power in this study, which is a limitation. We avoided correcting for multiple comparisons during statistical testing, which assuredly would have made any of the trends seen in our dataset even less significant. This raises the natural question about statistical power for the analysis conducted. To our knowledge, the TBI dataset used is currently the largest single-center NIRS dataset in this population. Whereas both the healthy volunteer and spine populations had datasets on the smaller side of the spectrum. As such, given the complexity of the sub-group analysis performed, we caution the readers as definitive conclusions regarding regional disparity within these sub-groups cannot be made. This challenge with statistical power is particularly an issue in the TBI sub-groups based on lesion locations and viable NIRS channels, where regional differences in pathology would have been expected to manifest in disparity in NIRS signals. However, within this progressive TBI sub-group analysis, diminishing group sizes may have impacted the trends in regional disparity that were seen. Future validation work is required with robust and larger datasets so that sufficient statistical power is maintained during such sub-group analytics. This may partially be remedied with rSO_2_ data being collected as part of the ongoing prospective multi-center NeurO_2_ study (ClinicalTrials.gov ID NCT04935866). Finally, the time needed to run the statistical tests was computationally tasking and implementing these methodologies on a larger cohort would benefit from more robust central computing services to automatically handle multiple patient analyses running in parallel.

## 6. Conclusions

Signal sources from commercially available cerebral NIRS systems with low sampling rates may not be able to reliably discern hemispheric differences in CA/CVR. In those with asymmetric cerebral pathology, there may be a role for such platforms to characterize hemispheric disparity in CA/CVR, as highlighted in our ARHD/MAD analysis of the TBI populations. A possibility of the lack of hemispheric disparity found in this analysis could be due to the lower sampling rate of the commercial NIRS devices used. High-frequency research-grade NIRS platforms, with increased sampling rates, are required to obtain waveform data along with increased spatial configuration to monitor more brain regions simultaneously. Also, the role of newer high-frequency NIRS systems in such spatial disparity analysis remains unclear and requires further evaluation in healthy and pathologic cohorts.

## Figures and Tables

**Table 1:** Demographic data for HC, SP, and TBI-GLR populations.

Variable	Median (IQR) or Number (%)
**Healthy control (HC) volunteers**	
Duration of recording (minutes)	31.1 (28.7–34.8)
Number of patients	102
Age (years)	26 (22.2–31)
Biological sex (male)	42 (41.2%)
Hand dominance (right)	93 (91.2%)
**Elective spinal surgery (SP) patients**	
Duration of recording (minutes)	185 (165.8–206.8)
Number of patients	27
Age (years)	57 (52–65.5)
Biological sex (male)	22 (81.5%)
Arterial pCO_2_ (mmHg)	42 (40–44.5)
Arterial pO_2_ (mmHg)	231 (211.5–295)
**Procedure type**	
ACDF	6 (22.2%)
PCDF	14 (51.9%)
ACDF and PCDF	3 (11.1%)
Cervical incision and drain	1 (3.7%)
Corpectomy	1 (3.7%)
Laminectomy	1 (3.7%)
Thoracic decompression and instrumental fusion	1 (3.7%)
**Anesthetic regimen**	
Propofol + Sufentanil	6 (22.2%)
Ketamine + Propofol + Sufentanil	6 (22.2%)
Midazolam + Propofol + Remi-Fentanyl	1 (3.7%)
Midazolam + Propofol + Sufentanil	4 (14.8%)
Propofol + Remi-Fentanyl + Sufentanil	2 (7.4%)
Ketamine + Midazolam + Propofol + Sufentanil	7 (25.9%)
Ketamine + Midazolam + Propofol + Remi-Fentanyl + Sufentanil	1 (3.7%)
**Traumatic brain injury patients (without bifrontal lobe pathology; TBI-GLR)**	
Duration of recordings (minutes)	4586.8 (2523.7–7685)
Number of patients	64
Age (years)	41 (26.6–57.5)
Biological sex (male)	51 (79.7%)
GCS	6 (4–8)
GCS motor	4 (2–5)
Hypoxia (yes)	19 (29.7%)
Hypotension (yes)	9 (14.1%)
Arterial pCO_2_ (mmHg)	37 (35–39.3)
Arterial pO_2_ (mmHg)	106.5 (93.3–123)
Focal injury (contusion, EDH, SDH, or aSDH)	42 (65.6%)
Diffuse injury (DAI or tSAH)	22 (34.4%)
**Pupils**	
Bilateral unreactive	8 (12.5%)
Unilateral unreactive	14 (21.9%)
Bilateral reactive	42 (65.6%)
**Marshall CT score**	
V	29 (45.3%)
IV	8 (12.5%)
III	25 (39.1%)
II	2 (3.1%)
**Rotterdam CT score**	
6	14 (21.9%)
5	16 (25.0%)
4	17 (26.6%)
3	13 (20.3%)
2	3 (4.7%)
1	1 (1.6%)
**Anesthetic regimen**	
None	2 (3.1%)
Propofol	14 (21.9%)
Fentanyl + Propofol	22 (34.4%)
Ketamine + Propofol	2 (3.1%)
Midazolam + Propofol	1 (1.6%)
Fentanyl + Ketamine + Propofol	5 (7.8%)
Fentanyl + Midazolam + Propofol	13 (20.3%)
Fentanyl + Ketamine + Midazolam + Propofol	5 (7.8%)

ACDF, anterior cervical discectomy and fusion; CT, computed tomography; DAI, diffuse axonal injury; EDH, epidural hematoma; GCS, Glasgow Coma Score; IQR, interquartile range; PCDF, posterior cervical discectomy and fusion; pCO_2_, partial pressure of carbon dioxide; pO_2_, partial pressure of oxygen; tSAH, traumatic subarachnoid hemorrhage; SDH, subdural hematoma; and aSDH, acute subdural hematoma.

**Table 2:** Physiologic results using 10 s data resolution for HC, SP, and TBI-GLR populations.

Physiologic Variable	HC		SP		TBI-GLR	
	Median (IQR) or Median (IQR; MAD)	*p*-Value	Median (IQR) or Median (IQR; MAD)	*p*-Value	Median (IQR) or Median (IQR; MAD)	*p*-Value
ABP (mmHg)	102.98 (97.54–107.01)	–	83.83 (76.85–88.87)	–	82.01 (75.76–89.66)	–
CPP (mmHg)	–		–		73.54 (67.25–80.96)	
rSO_2__L (%)	73.52 (72.8–74.46)	0.2394	66.26 (62.99–69)	0.9862	69.49 (66.43–74.38)	0.8172
rSO_2__R (%)	72.24 (71.47–73.56)		67.39 (66.69–68.85)		69 (65–73.9)	
COx_L (au)	–	–	–	–	0 (−0.18–0.22)	0.7582
COx_R (au)	–		–		0 (−0.18–0.23)	
COx-a_L (au)	0.14 (−0.08–0.34)	0.5993	0.17 (−0.11–0.44)	0.5054	0.05 (−0.1–0.28)	0.8710
COx-a_R (au)	0.12 (−0.09–0.32)		0.21 (−0.09–0.48)		0.05 (−0.11–0.27)	
MAD of ABP (mmHg)	7.52 (5.52–10.08)	–	8.82 (6.13–10.38)	–	8.88 (7.08–11.01)	–
MAD of CPP (mmHg)	–		–		8.72 (6.79–11.47)	
MAD of rSO_2__L (%)	1.45 (1.08–1.68)	0.1543	3.04 (2.44–4.45)	0.4834	4.98 (3.04–7.22)	0.7822
MAD of rSO_2__R (%)	1.47 (1.21–1.86)		2.94 (1.55–3.78)		4.86 (3.19–7.25)	
MAD of COx_L (au)	–	–	–	–	0.31 (0.27–0.35)	0.3767
MAD of COx_R (au)	–		–		0.33 (0.28–0.36)	
MAD of COx-a_L (au)	0.29 (0.23–0.35)	0.2624	0.44 (0.32–0.54)	0.6780	0.29 (0.26–0.32)	0.3022
MAD of COx-a_R (au)	0.27 (0.23–0.33)		0.43 (0.35–0.55)		0.3 (0.27–0.33)	
% time rSO_2__L > 60%	100 (100–100; 0)	0.1153	96.56 (46.76–99.78; 5.09)	0.9102	92.18 (57.43–99.38; 11.51)	0.5121
% time rSO_2__R > 60%	100 (100–100; 0)		97.56 (45.9–99.89; 3.62)		93.47 (62.06–99.83; 9.66)	
% time rSO_2__L > 70%	99.32 (42.87–100; 1.01)	0.1025	14.64 (0.32–85.31; 21.71)	0.9861	46.95 (5.42–81.56; 57.24)	0.7440
% time rSO_2__R > 70%	93.17 (1.03–100; 10.13)		13.51 (1.66–65.9; 20.03)		40.36 (14.54–77.29; 47.18)	
% time rSO_2__L > 80%	0 (0–29.91; 0)	0.9956	0 (0–8.38; 0)	0.6954	2.09 (0–24.39; 3.11)	0.9215
% time rSO_2__R > 80%	0 (0–23.39; 0)		0.18 (0–1.58; 0.27)		4.21 (0.01–20.17; 6.25)	
% time rSO_2__L > 90%	0 (0–0; 0)	0.5422	0 (0–0; 0)	0.5316	0 (0–0.07; 0)	0.6062
% time rSO_2__R > 90%	0 (0–0; 0)		0 (0–0; 0)		0 (0–0.41; 0)	
% time COx_L > 0	–	–	–	–	49.55 (42.77–55.47; 9.5)	0.9981
% time COx_R > 0	–		–		49.36 (43.55–56.82; 9.7)	
% time COx_L > 0.2	–	–	–	–	27.42 (23.79–35.25; 7.8)	0.8321
% time COx_R > 0.2	–		–		28 (21.51–37.92; 11.81)	
% time COx_L > 0.3	–	–	–	–	18.55 (15.56–26.37; 7.62)	0.7226
% time COx_R > 0.3	–		–		19.61 (13.78–29.1; 9.51)	
% time COx-a_L > 0	67.35 (51.11–79.83; 20.49)	0.5142	64.39 (55.55–71.61; 13.08)	0.4706	55.67 (48.79–64.69; 11.74)	0.9563
% time COx-a_R > 0	63.99 (53.85–77.73; 17.67)		66.88 (58.05–73.9; 11.88)		56.12 (49.98–65.28; 10.51)	
% time COx-a_L > 0.2	42.87 (27.83–55.62; 21.23)	0.5343	46.95 (41.21–55.91; 12.84)	0.4600	33.35 (27.58–39.52; 8.89)	0.8292
% time COx-a_R > 0.2	39.14 (29.31–52.66; 17.57)		51.16 (42.32–58.76; 12.75)		32.9 (28.06–41.19; 11.25)	
% time COx-a_L > 0.3	29.13 (17.09–41.45; 18.27)	0.4679	38.07 (31.75–44.57; 9.79)	0.3101	23.37 (18.33–28.79; 7.95)	0.7154
% time COx-a_R > 0.3	27.03 (15.51–40.36; 18.34)		41.83 (34.63–48.57; 10.78)		22.86 (18.76–30.84; 9.7)	

The *p*-values in the table are derived using Mann–Whitney U test between the bilateral signals. ABP, arterial blood pressure; au, arbitrary units; CPP, cerebral perfusion pressure; COx, cerebral oximetry index with CPP; COx-a, cerebral oximetry index with ABP; MAD, median absolute deviation; HC, healthy control volunteer group; IQR, interquartile range; mmHg, millimeters of mercury; rSO_2_, regional cerebral oxygen saturation; SP, elective spinal surgery patient group; and TBI-GLR, traumatic brain injury patient group without bifrontal lobe pathology.

**Table 3:** Regional hemispheric disparity analysis on 10 s data resolution for HC, SP, and TBI-GLR populations.

Physiologic Variable	Median (IQR)		
	HC	SP	TBI-GLR
ARHD of rSO_2_ (%)	3.87 (2.95–4.74)	4.41 (3.01–5.58)	5 (2.5–8.01)
ARHD of COx (au)	–	–	0.17 (0.07–0.32)
ARHD of COx-a (au)	0.14 (0.07–0.27)	0.15 (0.06–0.31)	0.16 (0.07–0.31)
MAD of ARHD rSO_2_ (%)	1.05 (0.79–1.47)	1.48 (1.02–1.99)	2.98 (2.1–4.45)
MAD of ARHD COx (au)	–	–	0.16 (0.13–0.19)
MAD of ARHD COx-a (au)	0.13 (0.1–0.15)	0.16 (0.13–0.2)	0.16 (0.14–0.18)

ARHD, absolute regional hemispheric difference; au, arbitrary units; COx, cerebral oximetry index with cerebral perfusion pressure; COx-a, cerebral oximetry index with arterial blood pressure; HC, healthy control volunteer group; IQR, interquartile range; MAD, median absolute deviation; rSO_2_, regional cerebral oxygen saturation; SP, elective spinal surgery patient group; and TBI-GLR, traumatic brain injury patient group without bifrontal lobe pathology.

**Table 4:** ADF and KPSS results showing stationary vs. non-stationary vs. NA for calculated CA indices—original and first-order-differenced HC, SP, and TBI-GLR data.

**ADF results for non-differenced data**													
**Dataset**	**Time Resolution**	**COx_L**			**COx_R**			**COx-a_L**			**COx-a_R**		
		**S**	**NS**	**NA**	**S**	**NS**	**NA**	**S**	**NS**	**NA**	**S**	**NS**	**NA**
HC	10 s	–	–	–	–	–	–	9	93	0	5	97	0
	1 min	–	–	–	–	–	–	27	75	0	27	75	0
	5 min	–	–	–	–	–	–	5	17	80	4	18	80
SP	10 s	–	–	–	–	–	–	25	2	0	22	5	0
	1 min	–	–	–	–	–	–	27	0	0	27	0	0
	5 min	–	–	–	–	–	–	16	11	0	14	13	0
TBI-GLR	10 s	63	1	0	63	1	1	64	0	0	64	0	0
	1 min	63	1	0	63	1	1	64	0	0	64	0	0
	5 min	61	2	1	61	2	1	64	0	0	64	0	0
**ADF results for first-order-differenced data**													
**Dataset**	**Time Resolution**	**COx_L**			**COx_R**			**COx-a_L**			**COx-a_R**		
		**S**	**NS**	**NA**	**S**	**NS**	**NA**	**S**	**NS**	**NA**	**S**	**NS**	**NA**
HC	10 s	–	–	–	–	–	–	102	0	0	102	0	0
	1 min	–	–	–	–	–	–	65	37	0	66	36	0
	5 min	–	–	–	–	–	–	1	6	95	1	6	95
SP	10 s	–	–	–	–	–	–	27	0	0	27	0	0
	1 min	–	–	–	–	–	–	27	0	0	27	0	0
	5 min	–	–	–	–	–	–	25	1	1	27	0	0
TBI-GLR	10 s	64	0	0	64	0	0	64	0	0	64	0	0
	1 min	63	1	0	63	1	0	64	0	0	64	0	0
	5 min	63	0	1	63	0	1	64	0	0	64	0	0
**KPSS results for non-differenced data**													
**Dataset**	**Time Resolution**	**COx_L**			**COx_R**			**COx-a_L**			**COx-a_R**		
		**S**	**NS**	**NA**	**S**	**NS**	**NA**	**S**	**NS**	**NA**	**S**	**NS**	**NA**
HC	10 s	–	–	–	–	–	–	39	63	0	47	55	0
	1 min	–	–	–	–	–	–	81	21	0	87	15	0
	5 min	–	–	–	–	–	–	102	0	0	102	0	0
SP	10 s	–	–	–	–	–	–	12	15	0	9	18	0
	1 min	–	–	–	–	–	–	25	2	0	23	4	0
	5 min	–	–	–	–	–	–	26	1	0	25	2	0
TBI-GLR	10 s	9	55	0	4	60	0	8	56	0	8	56	0
	1 min	19	45	0	13	51	0	22	42	0	20	44	0
	5 min	25	39	0	22	42	0	29	35	0	28	36	0
**KPSS results for first-order-differenced data**													
**Dataset**	**Time Resolution**	**COx_L**			**COx_R**			**COx-a_L**			**COx-a_R**		
		**S**	**NS**	**NA**	**S**	**NS**	**NA**	**S**	**NS**	**NA**	**S**	**NS**	**NA**
HC	10 s	–	–	–	–	–	–	101	1	0	102	0	0
	1 min	–	–	–	–	–	–	102	0	0	101	1	0
	5 min	–	–	–	–	–	–	98	4	0	99	3	0
SP	10 s	–	–	–	–	–	–	27	0	0	27	0	0
	1 min	–	–	–	–	–	–	27	0	0	27	0	0
	5 min	–	–	–	–	–	–	27	0	0	27	0	0
TBI-GLR	10 s	64	0	0	64	0	0	64	0	0	64	0	0
	1 min	64	0	0	64	0	0	64	0	0	64	0	0
	5 min	64	0	0	64	0	0	64	0	0	64	0	0

ADF, Augmented Dickey–Fuller; COx, cerebral oximetry index with cerebral perfusion pressure; COx-a, cerebral oximetry index with arterial blood pressure; HC, healthy control volunteer group; NA, unable to access stationarity; NS, non-stationary; S, stationary; SP, elective spinal surgery patient group; and TBI-GLR, traumatic brain injury patient group without bifrontal lobe pathology.

**Table 5:** Personalized ARIMA p-orders and q-orders based on AIC and their hemispheric disparity in 10 s data resolution for HC, SP, and TBI-GLR populations.

**Population**	**Personalized ARIMA p-orders [Median (IQR)]**					
	**rSO_2__L**	**rSO_2__R**	**COx_L**	**COx_R**	**COx-a_L**	**COx-a_R**
HC	2 (1–4)	2 (1–3)	–	–	3 (1–4)	3 (1–5)
SP	5 (2.5–8)	6 (3–8)	–	–	6 (5–8.5)	6 (5–8)
TBI-GLR	8 (6–10)	8.5 (6.75–10)	8 (7–9)	8 (6.75–9)	8 (7–9)	8 (6–9)
**Population**	**ARHD of personalized ARIMA p-orders [Median (IQR)]**					
	**rSO_2_**		**COx**		**COx-a**	
HC	1 (1–3)		–		2 (1–3)	
SP	3 (1–5)		–		2 (1–3)	
TBI-GLR	1.5 (0–3)		2 (1–2.25)		2 (1–2.25)	
**Population**	**Personalized ARIMA q-orders [Median (IQR)]**					
	**rSO_2__L**	**rSO_2__R**	**COx_L**	**COx_R**	**COx-a_L**	**COx-a_R**
HC	2 (1–4)	3 (1–5)	–	–	2 (0–4)	2 (0–4.75)
SP	7 (4–9.5)	7 (2.5–9.5)	–	–	5 (3–8)	5 (3–9)
TBI-GLR	9 (7–10)	9 (7–10)	10 (7–10)	9 (7–10)	9 (8–10)	9 (7–10)
**Population**	**ARHD of personalized ARIMA q-orders [Median (IQR)]**					
	**rSO_2_**		**COx**		**COx-a**	
HC	2 (1–3)		–		2 (1–4)	
SP	3 (2–6)		–		3 (1–6)	
TBI-GLR	1 (0–3)		1 (0–3)		2 (0–4)	

AIC, Akaike Information Criterion; ARHD, absolute regional hemispheric difference; COx, cerebral oximetry index with cerebral perfusion pressure; COx-a, cerebral oximetry index with arterial blood pressure; HC, healthy control volunteer group; IQR, interquartile range; p-order, autoregressive order; q-order, moving average order; rSO_2_, regional cerebral oxygen saturation; SP, elective spinal surgery patient group; and TBI-GLR, traumatic brain injury patient group without bifrontal lobe pathology.

**Table 6:** Hemispheric responsiveness using impulse response coefficients of VAR model using personalized and capped VAR p-orders in 10 s data resolution for HC, SP, and TBI-GLR populations.

Direction	HC (n = 102)		SP (n = 27)		TBI-GLR (n = 64)	
	>0.1% [% (count)]	NA [% (count)]	>0.1% [% (count)]	NA [% (count)]	>0.1% [% (count)]	NA [% (count)]
**Personalized VAR p-order**						
ABP → rSO_2__L	64.7% (66)	1% (1)	88.9% (24)	0% (0)	96.9% (62)	0% (0)
ABP → rSO_2__R	62.7% (64)	0% (0)	85.2% (23)	0% (0)	98.4% (63)	0% (0)
rSO_2__L → ABP	59.8% (61)	1% (1)	88.9% (24)	0% (0)	93.8% (60)	0% (0)
rSO_2__R → ABP	61.8% (63)	0% (0)	85.2% (23)	0% (0)	95.3% (61)	0% (0)
CPP → rSO_2__L	–	–	–	–	95.3% (61)	0% (0)
CPP → rSO_2__R	–	–	–	–	96.9% (62)	0% (0)
rSO_2__L → CPP	–	–	–	–	95.3% (61)	0% (0)
rSO_2__R → CPP	–	–	–	–	92.2% (59)	0% (0)
**Capped VAR p-order at 10**						
ABP → rSO_2__L	64.7% (66)	1% (1)	88.9% (24)	0% (0)	87.5% (56)	0% (0)
ABP → rSO_2__R	62.7% (64)	0% (0)	77.8% (21)	0% (0)	85.9% (55)	0% (0)
rSO_2__L → ABP	59.8% (61)	1% (1)	88.9% (24)	0% (0)	73.4% (47)	0% (0)
rSO_2__R → ABP	61.8% (63)	0% (0)	85.2% (23)	0% (0)	81.3% (52)	0% (0)
CPP → rSO_2__L	–	–	–	–	85.9% (55)	0% (0)
CPP → rSO_2__R	–	–	–	–	85.9% (55)	0% (0)
rSO_2__L → CPP	–	–	–	–	75% (48)	0% (0)
rSO_2__R → CPP	–	–	–	–	78.1% (50)	0% (0)

ABP, arterial blood pressure; CPP, cerebral perfusion pressure; HC, healthy control volunteer group; p-order, autoregressive order; rSO_2_, regional cerebral oxygen saturation; SP, elective spinal surgery patient group; TBI-GLR, traumatic brain injury patient group without bifrontal lobe pathology; and VAR, vector autoregressive.

**Table 7:** Granger causal directionality results based on greater F-statistic in 10 s data resolution for HC, SP, and TBI-GLR populations.

Signal	Direction	HC (n = 102) [% (Count)]	SP (n = 27) [% (Count)]	TBI-GLR (n = 64) [% (Count)]
**Personalized VAR p-order**				
ABP and rSO_2__L	ABP → rSO_2__L	65.7% (67)	74.1% (20)	81.3% (52)
	rSO_2__L → ABP	33.3% (34)	25.9% (7)	18.8% (12)
	NA	1% (1)	0% (0)	0% (0)
ABP and rSO_2__R	ABP → rSO_2__R	70.6% (72)	74.1% (20)	79.7% (51)
	rSO_2__R → ABP	29.4% (30)	25.9% (7)	20.3% (13)
	NA	0% (0)	0% (0)	0% (0)
CPP and rSO_2__L	CPP → rSO_2__L	–	–	82.8% (53)
	rSO_2__L → CPP	–	–	17.2% (11)
	NA	–	–	0% (0)
CPP and rSO_2__R	CPP → rSO_2__R	–	–	82.8% (53)
	rSO_2__R → CPP	–	–	17.2% (11)
	NA	–	–	0% (0)
**Capped VAR p-order at 10**				
ABP and rSO_2__L	ABP → rSO_2__L	71.6% (73)	70.4% (19)	87.5% (56)
	rSO_2__L → ABP	28.4% (29)	29.6% (8)	12.5% (8)
	NA	0% (0)	0% (0)	0% (0)
ABP and rSO_2__R	ABP → rSO_2__R	69.6% (71)	63% (17)	92.2% (59)
	rSO_2__R → ABP	30.4% (31)	37% (10)	7.8% (5)
	NA	0% (0)	0% (0)	0% (0)
CPP and rSO_2__L	CPP → rSO_2__L	–	–	82.8% (53)
	rSO_2__L → CPP	–	–	17.2% (11)
	NA	–	–	0% (0)
CPP and rSO_2__R	CPP → rSO_2__R	–	–	90.6% (58)
	rSO_2__R → CPP	–	–	9.4% (6)
	NA	–	–	0% (0)

ABP, arterial blood pressure; HC, healthy control volunteer group; p-order, autoregressive order; rSO_2_, regional cerebral oxygen saturation; SP, elective spinal surgery patient group; TBI-GLR, traumatic brain injury patient group without bifrontal lobe pathology.

## Data Availability

Research ethics board approval at our institution does not facilitate the free and open sharing of human data, regardless of the data being in a de-identified fashion. All such data are protected under both ethics and privacy acts within the Province of Manitoba, preventing such open sharing of data. All the data analyzed and used are available from the corresponding author upon reasonable request.

## Supplementary Materials

The following supporting information can be downloaded at: https://www.mdpi.com/article/10.3390/bioengineering12030247/s1, File S1: Methodology Appendix [39,50,53,68,69,70,71]; File S2: Demographic Data for TBI-GL, TBI-GR, TBI-BLR Populations; File S3: Mean Summary Metrics of Entire Recording Period; File S4: Absolute Regional Hemispheric Disparity (ARHD) and Median Absolute Deviation (MAD) Analysis; File S5: Time-Series Stationarity and Autoregressive Integrative Moving Average (ARIMA) Analysis; File S6: Impulse Response Function (IRF) Analysis; File S7: Granger Causality Analysis; File S8: Sub-group Analysis Appendix [39,50,53,68,69,70,71].

## Abbreviations

The following abbreviations are used in this manuscript:

ABP	Arterial blood pressure
ADF	Augmented Dickey–Fuller
AIC	Akaike Information Criterion
ARHD	Absolute regional hemispheric difference
ARIMA	Autoregressive integrative moving average
BTF	Brain Trauma Foundation
CA	Cerebral autoregulation
CBF	Cerebral blood flow
CBv	Cerebral blood volume
COx	Cerebral oximetry index with CPP
COx_L	Cerebral oximetry index with CPP of left frontal lobe
COx_R	Cerebral oximetry index with CPP of right frontal lobe
COx-a	Cerebral oximetry index with ABP
COx-a_L	Cerebral oximetry index with ABP of left frontal lobe
COx-a_R	Cerebral oximetry index with ABP of right frontal lobe
CPP	Cerebral perfusion pressure
CT	Computed tomography
CVR	Cerebrovascular reactivity
d-order	Moving average order
GCS	Glasgow Coma Scale
HC	Healthy volunteers
ICM+	Intensive Care Monitoring “Plus”
ICP	Intracranial pressure
ICU	Intensive care unit
IQR	Interquartile range
IRF	Impulse Response Function
KPSS	Kwiatkowski–Phillips–Schmidt–Shin
LLA	Lower Limit of autoregulation
MAIN-HUB	Multi-omic Analytics and Integrative Neuroinformatics in the HUman Brain
MAD	Median absolute deviation
NA	Represents missing value or undetermined value due to inadequate data
NIRS	Near infrared spectroscopy
pCO_2_	Partial pressure of carbon dioxide
pO_2_	Partial pressure of oxygen
p-order	Autoregressive order
PRx	Pressure reactivity index
q-order	Moving average order
rSO_2_	Regional cerebral oxygen saturation
rSO_2__L	Regional cerebral oxygen saturation of left frontal lobe
rSO_2__R	Regional cerebral oxygen saturation of right frontal lobe
SP	Elective spinal surgery patients
TBI	Traumatic brain injury
TBI-BLR	Traumatic brain injury patients with bifrontal lobe pathology
TBI-GL	Traumatic brain injury patients without left frontal lobe pathology
TBI_GLR	Traumatic brain injury patients without bifrontal lobe pathology
TBI-GR	Traumatic brain injury patients without right frontal lobe pathology
ULA	Upper limit of autoregulation
VAR	Vector autoregressive
