# Supplementary material for: Commercial NIRS May Not Detect Hemispheric Regional Disparity in Continuously Measured COx/COx-a: An Exploratory Healthy and Cranial Trauma Time-Series Analysis
Source: Bioengineering (Basel). 2025 Feb 28;12(3):247. doi: 10.3390/bioengineering12030247 (PMC11939202; doi:10.3390/bioengineering12030247)
Supplement: Supplementary file 1 [file bioengineering-12-00247-s001.zip › File S1.docx]

**File S1 – Methodology Appendix**

File S1 – Table of Contents

[File S1a: Evaluation of Data Stationarity 2](#_Toc191506415)

[File S1b: Evaluation of personalized Autoregressive Integrated Moving Average (ARIMA) orders 3](#_Toc191506416)

[File S1c: Generation of vector autoregressive (VAR) model 4](#_Toc191506417)

[File S1d: Subgroup Analysis 5](#_Toc191506418)

[File S1e: References 6](#_Toc191506419)

File S1a: Evaluation of Data Stationarity

Stationarity analysis was performed for each physiologic signal at an individual level using Augmented Dickey-Fuller (ADF) and Kwiatkowski-Phillips-Schmidt-Shin (KPSS) tests for the 10-second, 1-minute, and 5-minute data resolutions, in keeping with previous work from our group. The ADF test informs if the series is trend-stationary and KPSS test informs if the series is stationary around a linear trend [1]. The ADF and KPSS tests were run on each patient data using the “adf.test” and “kpss.test” functions from the *tseries* package ([https://cran.r-project.org/web/packages/tseries/‌index.html](https://cran.r-project.org/web/packages/tseries/index.html)) with an example of patient results shown for each dataset given in Appendices E1-E6.

The data from populations were 1^st^ order differenced and the above-mentioned stationarity analysis was re-run on each differenced data in the 10-second, 1-minute, and 5-minute data resolutions, with an example of patient results for each dataset shown in Appendices E1-E6. It is important to note that for the lower data resolutions (1-minute and 5-minute), the differencing was performed after the resolution was reduced because differencing at the native resolution before resolution reduction does not address the trend in the data as shown previously from our lab [2].

File S1b: Evaluation of personalized Autoregressive Integrated Moving Average (ARIMA) orders

Using R statistical software, Autoregressive Integrated Moving Average (ARIMA) model fit was performed in accordance with standard Box-Jenkin’s time-series methodologies [1,3–5]. Various ARIMA models were fit to each univariate physiologic time-series to model every 1^st^ order differenced signal for each patient in 10-second, 1-minute, and 5-minute data resolutions. The Akaike Information Criterion (AIC) value was obtained for ARIMA model fit on all physiologic variables (arterial blood pressure [ABP], cerebral perfusion pressure [CPP], left and right regional oxygen saturation [rSO_2__L and rSO_2__R, respectively], left and right cerebral oximetry index derived with CPP [COx_L and COx_R, respectively], and left and right cerebral oximetry index derived with ABP [COx-a_L and COx-a_R, respectively) where available since it is neither stringent or lenient as compared to other performance values [2]. Based on previous research from our lab [2,6], the list of ARIMA models were obtained by varying the autoregressive order (p-order), and the moving average order (q-order) from 1 to 10, and 0 to 10, respectively. The integrative order (d-order) was separately varied from 0 to 1 using the previously described differencing method. An example of these outputs are shown in File S5g.

Using AIC, an ARIMA p-order was determined for each physiologic signal of a patient. For a physiologic signal, the AIC values for the various ARIMA model fit were differenced using the lowest AIC value. Since a difference of more than four units is strong evidence of difference in models [7], all the differenced values lower than four were kept and used to check the number of times a p-order was present in this list. By selecting the most commonly occurred p-order, the personalized ARIMA p-orders, based on AIC, for each physiologic signal of a patient were saved. Similar method was used to obtain personalized ARIMA q-orders, based on AIC, for each physiologic signal of a patient. An example of a patient table is given in File S5h. The variation of these personalized ARIMA p-orders between the three sides of a signal were looked at along with calculating the hemispheric disparity of these p-orders and q-orders using the previously mentioned method.

File S1c: Generation of vector autoregressive (VAR) model

To represent the relationship between two types of signals, we derived vector autoregressive (VAR) models. The p-order for the VAR model was calculated by taking a product of the previously saved personalized ARIMA p-orders for the two signals being evaluated, as suggested from past literature [5]. This created a personalized VAR p-order for each 1^st^ order differenced signal pair in a patient’s data for 10-second, 1-minute, and 5-minute data resolutions. A cap for the personalized VAR p-order was set for all data resolutions at VAR p-order of 10. These VAR p-order caps were found by determining the AICs for VAR models of orders ranging from 1 – 100 and their plots indicated that the percent improvement, represented by lower AIC values, to lag order was best at the found caps in all signals in their respective resolutions. In certain instances where the VAR model could not be generated using the personalized VAR p-order due to signal length, the VAR p-order cap for that resolution was used to create the VAR model.

File S1d: Subgroup Analysis

All the populations were subdivided using simple parameters to see if re-evaluation of physiologic results, regional hemispheric disparity analysis, personalized ARIMA p-orders based on AIC, VAR IRF analysis, and Granger Causality analysis produced any outlying groups. The common parameters included for all groups were age (<60 vs ≥60; moderate vs old age), and biological sex (male vs female). Extra parameters included for the HC group was hand dominance (left hand vs right hand dominance. The SP group had the following additional parameters included in the subgroup analysis: procedure type (anterior cervical discectomy and fusion [ACDF], posterior cervical discectomy and fusion [PCDF], ACDF and PCDF, cervical incision and drain, corpectomy, laminectomy, and thoracic decompression and instrumental fusion), and anesthetic regimen (Propofol + Sufentanil, Ketamine + Propofol + Sufentanil, Midazolam + Propofol + Remi-Fentanyl, Midazolam + Propofol + Sufentanil, Propofol + Remi-Fentanyl + Sufentanil, Ketamine + Midazolam + Propofol + Sufentanil, and Ketamine + Midazolam + Propofol + Remi-Fentanyl + Sufentanil). Additional parameters for the TBI’s subgroup analysis included Focal Injury (Contusion, epidural hematoma [EDH], subdural hematoma [SDH], or acute SDH [aSDH]), Diffuse Injury (diffuse axonal injury [DAI] or traumatic subarachnoid hemorrhage [tSAH]), Marshall CT Score (V – II), Rotterdam CT Score (6 – 1), and anesthetic regimen (no anesthetic, Propofol, Fentanyl + Propofol, Ketamine + Propofol, Midazolam + Propofol, Fentanyl + Ketamine + Propofol, Fentanyl + Midazolam + Propofol, and Fentanyl + Ketamine + Midazolam + Propofol).

File S1e: References

1. Chatfield, C.; Xing, H. *The Analysis of Time Series: An Introduction with R*; 7th ed.; Chapman and Hall/CRC, 2019; ISBN 978-1-138-06613-7.

2. Sainbhi, A.S.; Vakitbilir, N.; Gomez, A.; Stein, K.Y.; Froese, L.; Zeiler, F.A. Time-Series Autocorrelative Structure of Cerebrovascular Reactivity Metrics in Severe Neural Injury: An Evaluation of the Impact of Data Resolution. *Biomedical Signal Processing and Control* **2024**, *95*, 106403, doi:10.1016/j.bspc.2024.106403.

3. Chatfield, C. *The Analysis of Time Series: An Introduction, Sixth Edition*; 6th ed.; Chapman and Hall/CRC: New York, 2003; ISBN 978-0-429-20870-6.

4. Zeiler, F.A.; Smielewski, P.; Stevens, A.; Czosnyka, M.; Menon, D.K.; Ercole, A. Non-Invasive Pressure Reactivity Index Using Doppler Systolic Flow Parameters: A Pilot Analysis. *Journal of Neurotrauma* **2019**, *36*, 713–720, doi:10.1089/neu.2018.5987.

5. Lütkepohl, H. *New Introduction to Multiple Time Series Analysis*; New York : Springer: Berlin, 2005; ISBN 978-3-540-40172-8.

6. Froese, L.; Gomez, A.; Sainbhi, A.S.; Batson, C.; Stein, K.; Alizadeh, A.; Zeiler, F.A. Dynamic Temporal Relationship Between Autonomic Function and Cerebrovascular Reactivity in Moderate/Severe Traumatic Brain Injury. *Frontiers in Network Physiology* **2022**, *2*, doi:10.3389/fnetp.2022.837860.

7. Mark C., G. *Intermediate Statistics with R*; Montana State University, 2014;
