# Supplementary material for: Commercial NIRS May Not Detect Hemispheric Regional Disparity in Continuously Measured COx/COx-a: An Exploratory Healthy and Cranial Trauma Time-Series Analysis
Source: Bioengineering (Basel). 2025 Feb 28;12(3):247. doi: 10.3390/bioengineering12030247 (PMC11939202; doi:10.3390/bioengineering12030247)
Supplement: Supplementary file 1 [file bioengineering-12-00247-s001.zip › File S2.docx]

**File S2 – Demographic Data for TBI-GL, TBI-GR, TBI-BLR Populations**

| **Variable** | **Median (IQR)  or Number (%)** |
| --- | --- |
| **Traumatic Brain Injury Patients (without left frontal lobe pathology; TBI-GL)** | |
| **Duration of Recordings (minutes)** | 5305 (3196.8 – 10707.7) |
| **Number of Patients** | 15 |
| **Age (years)** | 50 (32 – 56.5) |
| **Biological Sex (Male)** | 14 (93.3%) |
| **GCS** | 6 (4 – 7.5) |
| **GCS Motor** | 4 (2 – 5) |
| **Hypoxia (Yes)** | 3 (20%) |
| **Hypotension (Yes)** | 0 (0%) |
| **Arterial pCO_2_ (mmHg)** | 38.8 (37.3 – 40.8) |
| **Arterial pO_2_ (mmHg)** | 108 (97.1 – 122.2) |
| **Focal Injury (Contusion, EDH, SDH, or aSDH)** | 14 (93.3%) |
| **Diffuse Injury (DAI or tSAH)** | 1 (6.7%) |
| **Pupils** | |
| Bilateral Unreactive | 2 (13.3%) |
| Unilateral Unreactive | 4 (26.7%) |
| Bilateral Reactive | 9 (60%) |
| **Marshall CT Score** | |
| V | 8 (53.3%) |
| IV | 5 (33.3%) |
| III | 2 (13.3%) |
| II | 0 (0%) |
| **Rotterdam CT Score** | |
| 6 | 1 (6.7%) |
| 5 | 7 (46.7%) |
| 4 | 5 (33.3%) |
| 3 | 2 (13.3%) |
| **Anesthetic Regimen** | |
| Propofol | 5 (33.3%) |
| Fentanyl + Propofol | 3 (20%) |
| Fentanyl + Ketamine + Propofol | 1 (6.7%) |
| Fentanyl + Midazolam + Propofol | 4 (26.7%) |
| Fentanyl + Ketamine + Midazolam + Propofol | 2 (13.3%) |
| **Traumatic Brain Injury Patients (without right frontal lobe pathology; TBI-GR)** | |
| **Duration of Recordings (minutes)** | 3245.5 (2379.4 – 5993.6) |
| **Number of Patients** | 11 |
| **Age (years)** | 53 (36 – 67) |
| **Biological Sex (Male)** | 7 (63.6%) |
| **GCS** | 4 (3.5 – 6.5) |
| **GCS Motor** | 2 (1.5 – 4) |
| **Hypoxia (Yes)** | 5 (45.5%) |
| **Hypotension (Yes)** | 1 (9.1%) |
| **Arterial pCO_2_ (mmHg)** | 34.5 (33.5 – 35.6) |
| **Arterial pO_2_ (mmHg)** | 111 (103 – 130.1) |
| **Focal Injury (Contusion, EDH, SDH, or aSDH)** | 10 (90.9%) |
| **Diffuse Injury (DAI or tSAH)** | 1 (9.1%) |
| **Pupils** | |
| Bilateral Unreactive | 5 (45.5%) |
| Unilateral Unreactive | 2 (18.2%) |
| Bilateral Reactive | 4 (36.4%) |
| **Marshall CT Score** | |
| V | 8 (72.7%) |
| IV | 2 (18.2%) |
| III | 0 (0%) |
| II | 1 (9.1%) |
| **Rotterdam CT Score** | |
| 6 | 5 (45.5%) |
| 5 | 1 (9.1%) |
| 4 | 4 (36.4%) |
| 2 | 1 (9.1%) |
| **Anesthetic Regimen** | |
| Propofol | 3 (27.3%) |
| Fentanyl + Propofol | 6 (54.5%) |
| Fentanyl + Midazolam + Propofol | 2 (18.2%) |
| **Traumatic Brain Injury Patients (with bifrontal lobe pathology; TBI-BLR)** | |
| **Duration of Recordings (minutes)** | 9685.5 (7380.7 – 14308.2) |
| **Number of Patients** | 5 |
| **Age (years)** | 27 (23 – 32) |
| **Biological Sex (Male)** | 5 (100%) |
| **GCS** | 7 (6 – 8) |
| **GCS Motor** | 4 (4 – 5) |
| **Hypoxia (Yes)** | 1 (20%) |
| **Hypotension (Yes)** | 0 (0%) |
| **Arterial pCO_2_ (mmHg)** | 38 (35.3 – 42.3) |
| **Arterial pO_2_ (mmHg)** | 99.5 (81.8 – 133.5) |
| **Focal Injury (Contusion, EDH, SDH, or aSDH)** | 4 (80.0%) |
| **Diffuse Injury (DAI or tSAH)** | 1 (20.0%) |
| **Pupils** | |
| Bilateral Unreactive | 0 (0%) |
| Unilateral Unreactive | 2 (40%) |
| Bilateral Reactive | 3 (60%) |
| **Marshall CT Score** | |
| V | 3 (60%) |
| IV | 1 (20%) |
| III | 1 (20%) |
| II | 0 (0%) |
| **Rotterdam CT Score** | |
| 6 | 2 (40.0%) |
| 4 | 3 (60.0%) |
| **Anesthetic Regimen** | |
| Fentanyl + Propofol | 1 (20%) |
| Fentanyl + Ketamine + Propofol | 1 (20%) |
| Fentanyl + Midazolam + Propofol | 1 (20%) |
| Fentanyl + Ketamine + Midazolam + Propofol | 2 (40%) |
| *ACDF, anterior cervical discectomy and fusion; CT, computed tomography; DAI, diffuse axonal injury; EDH, epidural hematoma; GCS, Glasgow Coma Score; IQR, interquartile range; PCDF, posterior cervical discectomy and fusion; pCO_2_, partial pressure of carbon dioxide; pO_2_, partial pressure of oxygen; tSAH, traumatic subarachnoid hemorrhage; SDH, subdural hematoma; aSDH, acute subdural hematoma.* | |
