# Supplementary material for: Commercial NIRS May Not Detect Hemispheric Regional Disparity in Continuously Measured COx/COx-a: An Exploratory Healthy and Cranial Trauma Time-Series Analysis
Source: Bioengineering (Basel). 2025 Feb 28;12(3):247. doi: 10.3390/bioengineering12030247 (PMC11939202; doi:10.3390/bioengineering12030247)
Supplement: Supplementary file 1 [file bioengineering-12-00247-s001.zip › File S3.docx]

**File S3 – Mean Summary Metrics of Entire Recording Period**

File S3 – Table of Contents

[File S3a: Physiologic Results using 1-Minute and 5-Minute Data Resolutions for HC, SP, and TBI-GLR Populations 2](#_Toc191507461)

[File S3b: Physiologic Results using 10-Second, 1-Minute, and 5-Minute Data Resolutions for TBI-GL, TBI-GR, and TBI-BLR Populations 4](#_Toc191507462)

File S3a: Physiologic Results using 1-Minute and 5-Minute Data Resolutions for HC, SP, and TBI-GLR Populations

| **Physiologic Results for 1-Minute Data Resolution** | | | | | | |
| --- | --- | --- | --- | --- | --- | --- |
| **Physiologic Variable** | **HC** | | **SP** | | **TBI-GLR** | |
|  | **Median (IQR) or  Median (IQR; MAD)** | **p-value** | **Median (IQR) or  Median (IQR; MAD)** | **p-value** | **Median (IQR) or  Median (IQR; MAD)** | **p-value** |
| **ABP (mmHg)** | 102.44 (97.98 – 105.53) | – | 83.76 (77.01 – 88.92) | – | 82.09 (75.92 – 89.72) | – |
| **CPP (mmHg)** | – |  | – |  | 73.64 (67.41 – 80.83) |  |
| **rSO_2__L (%)** | 73.58 (72.8 – 74.28) | 0.2192 | 66.33 (62.99 – 69) | 1 | 69.42 (66.43 – 74.38) | 0.8265 |
| **rSO_2__R (%)** | 72.17 (71.49 – 73.34) |  | 67.38 (66.62 – 68.78) |  | 68.92 (65.17 – 73.92) |  |
| **COx_L (au)** | – | – | – | – | 0.02 (-0.18 – 0.24) | 0.9203 |
| **COx_R (au)** | – |  | – |  | 0.03 (-0.19 – 0.25) |  |
| **COx-a_L (au)** | 0.13 (-0.06 – 0.32) | 0.5668 | 0.19 (-0.11 – 0.45) | 0.7574 | 0.08 (-0.12 – 0.29) | 0.8732 |
| **COx-a_R (au)** | 0.11 (-0.07 – 0.3) |  | 0.2 (-0.08 – 0.46) |  | 0.08 (-0.13 – 0.28) |  |
| **MAD of ABP (mmHg)** | 5.73 (3.96 – 8.85) | – | 8.31 (5.86 – 10.17) | – | 8.67 (6.82 – 10.96) | – |
| **MAD of CPP (mmHg)** | – |  | – |  | 8.49 (6.7 – 11.44) |  |
| **MAD of rSO_2__L (%)** | 1.17 (0.88 – 1.59) | 0.1348 | 3.12 (2.37 – 4.53) | 0.4812 | 4.84 (3.07 – 7) | 0.7786 |
| **MAD of rSO_2__R (%)** | 1.23 (0.97 – 1.67) |  | 2.9 (1.8 – 3.67) |  | 4.77 (3.21 – 7.04) |  |
| **MAD of COx_L (au)** | – | – | – | – | 0.31 (0.29 – 0.35) | 0.3515 |
| **MAD of COx_R (au)** | – |  | – |  | 0.33 (0.3 – 0.36) |  |
| **MAD of COx-a_L (au)** | 0.28 (0.22 – 0.34) | 0.1169 | 0.44 (0.3 – 0.52) | 0.8503 | 0.29 (0.27 – 0.33) | 0.3454 |
| **MAD of COx-a_R (au)** | 0.25 (0.21 – 0.32) |  | 0.45 (0.34 – 0.53) |  | 0.3 (0.28 – 0.33) |  |
| **% time rSO_2__L > 60%** | 100 (100 – 100; 0) | 0.0681 | 97.63 (46.78 – 100; 3.51) | 0.8944 | 92.65 (58.13 – 99.42; 10.86) | 0.4815 |
| **% time rSO_2__R > 60%** | 100 (100 – 100; 0) |  | 97.92 (49.02 – 100; 3.09) |  | 93.78 (62.29 – 99.81; 9.22) |  |
| **% time rSO_2__L > 70%** | 100 (45.11 – 100; 0) | **0.0306** | 16.55 (0.54 – 84.47; 24.54) | 0.9097 | 48.22 (5.61 – 81.82; 58.26) | 0.7549 |
| **% time rSO_2__R > 70%** | 95.27 (0 – 100; 7.01) |  | 13.68 (1 – 66.02; 20.27) |  | 40.85 (14.9 – 78.29; 47.14) |  |
| **% time rSO_2__L > 80%** | 0 (0 – 30.39; 0) | 0.6877 | 0 (0 – 9.01; 0) | 0.8995 | 2.26 (0 – 24.99; 3.36) | 0.9023 |
| **% time rSO_2__R > 80%** | 0 (0 – 25.69; 0) |  | 0 (0 – 1.41; 0) |  | 4.1 (0 – 20.61; 6.07) |  |
| **% time rSO_2__L > 90%** | 0 (0 – 0; 0) | 0.8339 | 0 (0 – 0; 0) | 0.3509 | 0 (0 – 0.08; 0) | 0.7939 |
| **% time rSO_2__R > 90%** | 0 (0 – 0; 0) |  | 0 (0 – 0; 0) |  | 0 (0 – 0.39; 0) |  |
| **% time COx_L > 0** | – | – | – | – | 53.46 (47.86 – 64.52; 10.65) | 0.9279 |
| **% time COx_R > 0** | – |  | – |  | 52.72 (47.47 – 63.02; 12.65) |  |
| **% time COx_L > 0.2** | – | – | – | – | 28.41 (24.09 – 39.31; 9.62) | 0.8061 |
| **% time COx_R > 0.2** | – |  | – |  | 29.28 (22.33 – 41.07; 12.64) |  |
| **% time COx_L > 0.3** | – | – | – | – | 19.59 (15.43 – 28.17; 9.23) | 0.6837 |
| **% time COx_R > 0.3** | – |  | – |  | 20.13 (13.93 – 30.92; 11.06) |  |
| **% time COx-a_L > 0** | 67.8 (50.89 – 81.13; 20.56) | 0.4751 | 67.92 (58.35 – 75.14; 12.2) | 0.8638 | 61.41 (54.08 – 67.61; 10.56) | 0.7951 |
| **% time COx-a_R > 0** | 65.75 (54.17 – 77.5; 17.5) |  | 69.77 (58.36 – 75.91; 11.7) |  | 60.46 (53.14 – 69.64; 12.04) |  |
| **% time COx-a_L > 0.2** | 41.74 (26.4 – 55.7; 22.59) | 0.5421 | 49.07 (41.08 – 57.39; 12.56) | 0.5977 | 34.65 (28.31 – 42.44; 9.94) | 0.9867 |
| **% time COx-a_R > 0.2** | 39.74 (28.57 – 51.85; 17.96) |  | 50.16 (41.24 – 59.2; 13.56) |  | 33.84 (28.01 – 42.39; 10.98) |  |
| **% time COx-a_L > 0.3** | 29.06 (15.21 – 43.53; 21.2) | 0.4372 | 37.96 (31.61 – 45.24; 10.48) | 0.4600 | 24.18 (19.44 – 30.19; 8.08) | 0.8882 |
| **% time COx-a_R > 0.3** | 26.57 (13.57 – 36.79; 18.21) |  | 42.54 (33.62 – 49.09; 12.41) |  | 23.46 (18.36 – 31.91; 9.77) |  |
| **Physiologic Results for 5-Minute Data Resolution** | | | | | | |
| **Physiologic Variable** | **HC** | | **SP** | | **TBI-GLR** | |
|  | **Median (IQR) or  Median (IQR; MAD)** | **p-value** | **Median (IQR) or  Median (IQR; MAD)** | **p-value** | **Median (IQR) or  Median (IQR; MAD)** | **p-value** |
| **ABP (mmHg)** | 102.85 (99.44 – 104.84) | – | 83.74 (77.57 – 88.19) | – | 82.21 (76.12 – 89.75) | – |
| **CPP (mmHg)** | – |  | – |  | 73.69 (67.52 – 80.7) |  |
| **rSO_2__L (%)** | 73.47 (72.96 – 73.99) | 0.2032 | 66.3 (63.16 – 68.62) | 0.9453 | 69.45 (66.47 – 74.35) | 0.8209 |
| **rSO_2__R (%)** | 72.36 (71.92 – 72.98) |  | 67.34 (66.71 – 69.01) |  | 68.85 (65.19 – 73.81) |  |
| **COx_L (au)** | – | – | – | – | 0.02 (-0.15 – 0.2) | 0.9943 |
| **COx_R (au)** | – |  | – |  | 0.02 (-0.16 – 0.22) |  |
| **COx-a_L (au)** | 0.13 (0.02 – 0.25) | 0.3834 | 0.17 (-0.06 – 0.38) | 0.7969 | 0.08 (-0.09 – 0.25) | 0.8135 |
| **COx-a_R (au)** | 0.11 (-0.01 – 0.24) |  | 0.22 (-0.02 – 0.42) |  | 0.08 (-0.1 – 0.25) |  |
| **MAD of ABP (mmHg)** | 4.69 (2.73 – 7.31) | – | 7.47 (5.52 – 9.53) | – | 8.95 (6.66 – 11.01) | – |
| **MAD of CPP (mmHg)** | – |  | – |  | 8.45 (6.48 – 11.28) |  |
| **MAD of rSO_2__L (%)** | 0.84 (0.47 – 1.24) | 0.3209 | 2.83 (2.13 – 4.55) | 0.5593 | 4.81 (3.01 – 6.97) | 0.7405 |
| **MAD of rSO_2__R (%)** | 0.87 (0.47 – 1.47) |  | 2.75 (1.77 – 3.61) |  | 4.8 (3.23 – 7.01) |  |
| **MAD of COx_L (au)** | – | – | – | – | 0.26 (0.24 – 0.31) | 0.3067 |
| **MAD of COx_R (au)** | – |  | – |  | 0.28 (0.24 – 0.31) |  |
| **MAD of COx-a_L (au)** | 0.2 (0.14 – 0.28) | 0.1348 | 0.35 (0.24 – 0.41) | 1 | 0.25 (0.23 – 0.28) | 0.6084 |
| **MAD of COx-a_R (au)** | 0.16 (0.12 – 0.26) |  | 0.34 (0.27 – 0.38) |  | 0.25 (0.23 – 0.28) |  |
| **% time rSO_2__L > 60%** | 100 (100 – 100; 0) | 0.1699 | 96.51 (47.48 – 100; 5.17) | 0.9707 | 92.78 (58.48 – 99.32; 10.67) | 0.4359 |
| **% time rSO_2__R > 60%** | 100 (100 – 100; 0) |  | 97.47 (54.04 – 100; 3.75) |  | 94 (62.49 – 99.86; 8.9) |  |
| **% time rSO_2__L > 70%** | 100 (40 – 100; 0) | 0.0964 | 15.15 (1.28 – 84.34; 22.46) | 0.9442 | 48.55 (5.66 – 81.64; 58.09) | 0.7368 |
| **% time rSO_2__R > 70%** | 100 (0 – 100; 0) |  | 16.67 (0 – 65.37; 24.71) |  | 42.76 (14.98 – 76.73; 46.33) |  |
| **% time rSO_2__L > 80%** | 0 (0 – 25.6; 0) | 0.8725 | 0 (0 – 9; 0) | 0.5990 | 2.03 (0 – 25.83; 3.02) | 0.9172 |
| **% time rSO_2__R > 80%** | 0 (0 – 20; 0) |  | 0 (0 – 1.88; 0) |  | 3.12 (0 – 20.85; 4.62) |  |
| **% time rSO_2__L > 90%** | 0 (0 – 0; 0) | 0.5569 | 0 (0 – 0; 0) | 0.4928 | 0 (0 – 0.01; 0) | 0.7897 |
| **% time rSO_2__R > 90%** | 0 (0 – 0; 0) |  | 0 (0 – 0; 0) |  | 0 (0 – 0.24; 0) |  |
| **% time COx_L > 0** | – | – | – | – | 54.56 (48.88 – 67.22; 13.01) | 0.7030 |
| **% time COx_R > 0** | – |  | – |  | 53.61 (46.31 – 64.53; 14.66) |  |
| **% time COx_L > 0.2** | – | – | – | – | 25.17 (20.2 – 36.75; 11.81) | 0.7513 |
| **% time COx_R > 0.2** | – |  | – |  | 27.2 (18.66 – 40.08; 14.5) |  |
| **% time COx_L > 0.3** | – | – | – | – | 15.29 (11.2 – 24; 8.6) | 0.6995 |
| **% time COx_R > 0.3** | – |  | – |  | 16.9 (10.41 – 26.99; 11.15) |  |
| **% time COx-a_L > 0** | 71.43 (50 – 85.71; 31.77) | 0.6221 | 69.23 (62.21 – 80.21; 13.71) | 0.8491 | 63.75 (54.39 – 70.62; 11.74) | 0.7841 |
| **% time COx-a_R > 0** | 70.71 (50 – 83.33; 18.71) |  | 72 (61.09 – 79.05; 12.08) |  | 62.77 (54.26 – 72.54; 13.1) |  |
| **% time COx-a_L > 0.2** | 37.5 (17.5 – 57.14; 30) | 0.6866 | 45.45 (36.63 – 60.56; 21.57) | 0.6342 | 32.71 (25.11 – 40.57; 11.6) | 0.9468 |
| **% time COx-a_R > 0.2** | 35.42 (16.67 – 50; 27.8) |  | 54.43 (38.41 – 61.43; 18.64) |  | 31.15 (24.5 – 41.38; 11.62) |  |
| **% time COx-a_L > 0.3** | 20 (3.13 – 48.21; 29.65) | 0.1120 | 31.43 (26.62 – 42.83; 14.45) | 0.2428 | 19.68 (15.64 – 26.38; 7.75) | 0.9203 |
| **% time COx-a_R > 0.3** | 20 (0 – 33.33; 29.65) |  | 36.11 (31.82 – 47.82; 16.47) |  | 18.56 (14.71 – 28.12; 9.92) |  |
| *The p-values in the table are derived using Mann-Whitney U test between the bilateral signals.*  *ABP, arterial blood pressure; au, arbitrary units; CPP, cerebral perfusion pressure; COx, cerebral oximetry index with CPP; COx-a, cerebral oximetry index with ABP; MAD, median absolute deviation; HC, healthy control volunteer group; IQR, interquartile range; mmHg, millimeters of mercury; rSO_2_, regional cerebral oxygen saturation; SP, elective spinal surgery patient group; TBI-GLR, traumatic brain injury patient group without bifrontal lobe pathology.* | | | | | | |

File S3b: Physiologic Results using 10-Second, 1-Minute, and 5-Minute Data Resolutions for TBI-GL, TBI-GR, and TBI-BLR Populations

| **Physiologic Results for 10-Second Data Resolution** | | | | | | |
| --- | --- | --- | --- | --- | --- | --- |
| **Physiologic Variable** | **TBI-GL** | | **TBI-GR** | | **TBI-BLR** | |
|  | **Median (IQR) or  Median (IQR; MAD)** | **p-value** | **Median (IQR) or  Median (IQR; MAD)** | **p-value** | **Median (IQR) or  Median (IQR; MAD)** | **p-value** |
| **ABP (mmHg)** | 83.06 (76.59 – 90.75) | – | 79.52 (76.39 – 82.75) | – | 88 (82.88 – 95.86) | – |
| **CPP (mmHg)** | 73.51 (68.22 – 80.4) |  | 72.91 (65.25 – 80.15) |  | 73.92 (68.33 – 83.73) |  |
| **rSO_2__L (%)** | 66.99 (60.99 – 71.99) | 0.7874 | 74 (69.99 – 79.5) | 1 | 66.48 (61.99 – 74.5) | 0.0952 |
| **rSO_2__R (%)** | 69.35 (64.07 – 73.02) |  | 76.65 (72.99 – 78.99) |  | 72.74 (68.32 – 77) |  |
| **COx_L (au)** | 0 (-0.17 – 0.25) | 0.9661 | 0 (-0.12 – 0.21) | 0.2738 | 0 (-0.19 – 0.16) | 0.6558 |
| **COx_R (au)** | 0 (-0.12 – 0.22) |  | -0.01 (-0.23 – 0.14) |  | 0 (-0.19 – 0.2) |  |
| **COx-a_L (au)** | 0.03 (-0.1 – 0.27) | 0.4244 | 0 (-0.11 – 0.22) | 0.7856 | 0 (-0.18 – 0.18) | 0.6558 |
| **COx-a_R (au)** | 0.01 (-0.07 – 0.22) |  | 0 (-0.15 – 0.19) |  | 0 (-0.16 – 0.21) |  |
| **MAD of ABP (mmHg)** | 10.22 (7.83 – 11.88) | – | 7.44 (5.9 – 8.08) | – | 9.04 (7.29 – 9.77) | – |
| **MAD of CPP (mmHg)** | 10.38 (7.4 – 11.29) |  | 7.19 (6.11 – 9.41) |  | 9.41 (9.33 – 10.76) |  |
| **MAD of rSO_2__L (%)** | 5.14 (4.4 – 7.48) | 0.1709 | 3.68 (2.98 – 5.2) | 0.3402 | 5.93 (2.94 – 6.69) | 0.3095 |
| **MAD of rSO_2__R (%)** | 3.83 (3.04 – 6.55) |  | 4.45 (3.96 – 7.42) |  | 6.72 (4.54 – 7.56) |  |
| **MAD of COx_L (au)** | 0.32 (0.26 – 0.33) | 0.3245 | 0.27 (0.24 – 0.29) | 0.6522 | 0.3 (0.26 – 0.3) | 1 |
| **MAD of COx_R (au)** | 0.28 (0.26 – 0.31) |  | 0.29 (0.23 – 0.31) |  | 0.29 (0.29 – 0.3) |  |
| **MAD of COx-a_L (au)** | 0.29 (0.27 – 0.33) | 0.1607 | 0.27 (0.23 – 0.29) | 0.7969 | 0.27 (0.25 – 0.3) | 1 |
| **MAD of COx-a_R (au)** | 0.28 (0.25 – 0.3) |  | 0.26 (0.23 – 0.29) |  | 0.29 (0.28 – 0.29) |  |
| **% time rSO_2__L > 60%** | 80.07 (73.99 – 95.13; 16.26) | 0.3892 | 99.91 (97.72 – 100; 0.14) | 0.1356 | 84.08 (71.39 – 97.28; 19.56) | 0.3095 |
| **% time rSO_2__R > 60%** | 94.14 (71.48 – 99.71; 8.68) |  | 98.21 (92.95 – 99.91; 2.63) |  | 96.62 (93.67 – 99.93; 4.92) |  |
| **% time rSO_2__L > 70%** | 33.91 (11.88 – 62.41; 41.41) | 0.4937 | 71.92 (40.9 – 99.98; 41.64) | 0.8177 | 43.01 (27.79 – 43.44; 4.34) | 0.2222 |
| **% time rSO_2__R > 70%** | 46.78 (16.37 – 63.16; 33.3) |  | 84.39 (38.42 – 99.89; 23.12) |  | 65.4 (42.67 – 81.84; 33.71) |  |
| **% time rSO_2__L > 80%** | 0.53 (0.26 – 3.77; 0.55) | 0.9337 | 17.61 (1.61 – 50.32; 26.12) | 0.8434 | 0.29 (0.2 – 6.63; 0.43) | 0.3095 |
| **% time rSO_2__R > 80%** | 0.65 (0.01 – 22.55; 0.97) |  | 14.96 (5.74 – 56.69; 22.18) |  | 12.52 (4.74 – 29.12; 18.55) |  |
| **% time rSO_2__L > 90%** | 0 (0 – 0.03; 0) | 0.5325 | 0 (0 – 7.38; 0) | 0.6233 | 0 (0 – 0.13; 0) | 0.5038 |
| **% time rSO_2__R > 90%** | 0 (0 – 0.84; 0) |  | 0.08 (0 – 1.47; 0.11) |  | 0.01 (0 – 2.56; 0.01) |  |
| **% time COx_L > 0** | 50.02 (45.82 – 52.92; 5.7) | 0.9025 | 48.96 (43.68 – 55.44; 9.62) | 0.0759 | 40.79 (36.87 – 45.85; 7.5) | 0.2222 |
| **% time COx_R > 0** | 49.84 (46.03 – 56.56; 6.43) |  | 41 (35.15 – 43.3; 5.7) |  | 46.42 (46.17 – 46.98; 0.84) |  |
| **% time COx_L > 0.2** | 28.62 (23.65 – 31.61; 7.19) | 0.6529 | 25.54 (22.51 – 29.03; 7.29) | 0.1932 | 21.07 (16.45 – 21.73; 6.86) | 0.1508 |
| **% time COx_R > 0.2** | 26.89 (23.95 – 32.83; 5.07) |  | 21.2 (18.97 – 23.66; 4.96) |  | 25.09 (23.14 – 25.8; 2.9) |  |
| **% time COx_L > 0.3** | 21.55 (15.45 – 22.63; 5.08) | 0.5949 | 17.63 (14.52 – 20.25; 6.5) | 0.3000 | 13.49 (10.73 – 14.13; 4.1) | 0.3095 |
| **% time COx_R > 0.3** | 17.69 (15.83 – 23.54; 4.75) |  | 14.27 (13.09 – 16.97; 2.47) |  | 16.93 (14.05 – 19.05; 4.27) |  |
| **% time COx-a_L > 0** | 53.68 (50.48 – 59.23; 6.32) | 0.4610 | 48.25 (41.61 – 55.35; 13) | 0.3653 | 46.6 (45.88 – 50.06; 5.13) | 0.8413 |
| **% time COx-a_R > 0** | 51.96 (46.83 – 60.95; 9.34) |  | 44.05 (41.12 – 48.29; 4.72) |  | 46.89 (46.48 – 49.85; 0.78) |  |
| **% time COx-a_L > 0.2** | 31.2 (29.19 – 37.74; 3.48) | 0.1261 | 26.47 (21.96 – 29; 4.48) | 0.6063 | 23.27 (20.82 – 25.11; 3.63) | 0.5476 |
| **% time COx-a_R > 0.2** | 27.23 (23.43 – 39.25; 8) |  | 24.23 (20.71 – 26.53; 4.85) |  | 25.94 (22.23 – 27.36; 5.5) |  |
| **% time COx-a_L > 0.3** | 22.53 (21.43 – 27.96; 3.46) | 0.1485 | 17.03 (14.13 – 20.4; 4.82) | 0.6994 | 14.45 (12.51 – 17.15; 4) | 0.5476 |
| **% time COx-a_R > 0.3** | 17.88 (16.1 – 29.05; 7.02) |  | 16.2 (13.34 – 18.48; 4.06) |  | 19.18 (13.62 – 19.35; 6.65) |  |
| **Physiologic Results for 1-Minute Data Resolution** | | | | | | |
| **Physiologic Variable** | **TBI-GL** | | **TBI-GR** | | **TBI-BLR** | |
|  | **Median (IQR) or  Median (IQR; MAD)** | **p-value** | **Median (IQR) or  Median (IQR; MAD)** | **p-value** | **Median (IQR) or  Median (IQR; MAD)** | **p-value** |
| **ABP (mmHg)** | 83.13 (76.68 – 90.45) | – | 79.56 (76.38 – 82.57) | – | 88.02 (83.01 – 95.85) | – |
| **CPP (mmHg)** | 73.58 (68.31 – 80.57) |  | 72.91 (65.24 – 80.05) |  | 73.85 (68.42 – 83.73) |  |
| **rSO_2__L (%)** | 66.99 (60.99 – 72.04) | 0.7437 | 74 (69.99 – 79.48) | 1 | 66.45 (61.75 – 74.49) | 0.0952 |
| **rSO_2__R (%)** | 69.34 (64.27 – 73.16) |  | 76.59 (72.86 – 78.99) |  | 72.67 (68.4 – 77.19) |  |
| **COx_L (au)** | 0.05 (-0.18 – 0.29) | 0.8381 | 0.05 (-0.14 – 0.23) | 0.2703 | -0.01 (-0.19 – 0.18) | 0.6905 |
| **COx_R (au)** | 0.04 (-0.16 – 0.27) |  | -0.05 (-0.26 – 0.17) |  | 0 (-0.21 – 0.21) |  |
| **COx-a_L (au)** | 0.08 (-0.13 – 0.31) | 0.4124 | 0.04 (-0.14 – 0.22) | 0.4779 | 0 (-0.19 – 0.2) | 0.6905 |
| **COx-a_R (au)** | 0.05 (-0.15 – 0.26) |  | 0.01 (-0.2 – 0.2) |  | 0.02 (-0.18 – 0.23) |  |
| **MAD of ABP (mmHg)** | 9.85 (7.77 – 11.72) | – | 7.42 (5.69 – 8) | – | 8.8 (7.04 – 9.65) | – |
| **MAD of CPP (mmHg)** | 10.49 (7.45 – 11.05) |  | 7.16 (5.89 – 8.94) |  | 9.32 (9.12 – 10.97) |  |
| **MAD of rSO_2__L (%)** | 5.17 (4.31 – 7.65) | 0.1485 | 3.68 (3.08 – 5.25) | 0.4009 | 5.47 (2.88 – 6.83) | 0.4263 |
| **MAD of rSO_2__R (%)** | 3.73 (3.12 – 6.59) |  | 4.29 (3.89 – 7.54) |  | 6.77 (4.57 – 7.8) |  |
| **MAD of COx_L (au)** | 0.33 (0.3 – 0.35) | 0.0675 | 0.29 (0.28 – 0.31) | 0.0759 | 0.32 (0.29 – 0.32) | 1 |
| **MAD of COx_R (au)** | 0.3 (0.28 – 0.32) |  | 0.33 (0.3 – 0.35) |  | 0.31 (0.28 – 0.32) |  |
| **MAD of COx-a_L (au)** | 0.32 (0.3 – 0.34) | 0.0892 | 0.29 (0.28 – 0.31) | 0.9476 | 0.32 (0.26 – 0.32) | 1 |
| **MAD of COx-a_R (au)** | 0.3 (0.28 – 0.31) |  | 0.28 (0.28 – 0.33) |  | 0.31 (0.26 – 0.31) |  |
| **% time rSO_2__L > 60%** | 81.41 (74.7 – 95.56; 15.01) | 0.4304 | 99.84 (97.73 – 100; 0.23) | 0.2049 | 85.09 (72.49 – 97.24; 18.55) | 0.3095 |
| **% time rSO_2__R > 60%** | 94.68 (72.45 – 99.67; 7.89) |  | 98.2 (93.21 – 99.89; 2.67) |  | 96.64 (93.72 – 99.92; 4.87) |  |
| **% time rSO_2__L > 70%** | 34.15 (12.28 – 63.77; 41.44) | 0.4937 | 73.25 (43.17 – 100; 39.67) | 0.8423 | 43.15 (28.97 – 43.97; 5.65) | 0.0952 |
| **% time rSO_2__R > 70%** | 46.63 (16.65 – 64.17; 32.27) |  | 84.53 (38.88 – 99.85; 22.94) |  | 66.49 (44.13 – 82.96; 33.14) |  |
| **% time rSO_2__L > 80%** | 0.48 (0.28 – 4.16; 0.52) | 0.9006 | 19.68 (1.98 – 52.98; 29.17) | 0.8430 | 0.4 (0.21 – 6.81; 0.6) | 0.3457 |
| **% time rSO_2__R > 80%** | 0.75 (0.01 – 23.46; 1.12) |  | 16.2 (6.34 – 57.43; 24.01) |  | 12.83 (5.07 – 29.27; 19.02) |  |
| **% time rSO_2__L > 90%** | 0 (0 – 0.02; 0) | 0.5325 | 0 (0 – 8.2; 0) | 0.8018 | 0 (0 – 0.11; 0) | 0.5038 |
| **% time rSO_2__R > 90%** | 0 (0 – 0.94; 0) |  | 0.09 (0 – 1.82; 0.14) |  | 0.01 (0 – 2.61; 0.02) |  |
| **% time COx_L > 0** | 55.22 (50.18 – 60.57; 8.15) | 0.7748 | 57.15 (46.66 – 61.38; 10.8) | 0.2169 | 48.53 (42.11 – 54.47; 9.51) | 0.6905 |
| **% time COx_R > 0** | 54.68 (51.54 – 63.22; 6.62) |  | 43.86 (38.84 – 54.47; 10.91) |  | 50.22 (49.49 – 52.13; 2.82) |  |
| **% time COx_L > 0.2** | 31.77 (24.62 – 35.77; 9.35) | 0.9674 | 28.48 (23.27 – 34.26; 8.85) | 0.3000 | 22 (21.06 – 30.76; 9.71) | 0.6905 |
| **% time COx_R > 0.2** | 30.66 (25.1 – 36.48; 8.91) |  | 22.53 (19.44 – 29.06; 4.93) |  | 26.44 (25.54 – 26.67; 1.33) |  |
| **% time COx_L > 0.3** | 24.36 (15.65 – 25.37; 7.89) | 0.7748 | 18.35 (14.45 – 22.34; 6.4) | 0.6063 | 14.04 (13.62 – 22.77; 6.07) | 0.8413 |
| **% time COx_R > 0.3** | 22.25 (15.98 – 24.81; 9.04) |  | 16.05 (13.31 – 20.29; 4.23) |  | 17.78 (14.98 – 20; 4.15) |  |
| **% time COx-a_L > 0** | 60.84 (57.26 – 62.75; 4.56) | 0.5668 | 56.49 (47.17 – 61.84; 10.49) | 0.4385 | 50 (48.59 – 51.42; 2.11) | 0.6905 |
| **% time COx-a_R > 0** | 56.04 (52.02 – 67.24; 7.9) |  | 51.05 (46.74 – 56.5; 6.66) |  | 52.46 (50.87 – 54.08; 2.4) |  |
| **% time COx-a_L > 0.2** | 36.29 (32.02 – 37.85; 3.68) | 0.3669 | 27.58 (23.25 – 34.62; 9.93) | 0.7477 | 25.09 (22.18 – 29.73; 5.69) | 1 |
| **% time COx-a_R > 0.2** | 30.59 (25.89 – 42.58; 11.1) |  | 25.08 (22.11 – 30.57; 7.16) |  | 27.3 (24.22 – 28.95; 4.57) |  |
| **% time COx-a_L > 0.3** | 25.71 (23.19 – 27.51; 2.71) | 0.3453 | 18.34 (14.62 – 24.41; 6.7) | 0.8977 | 16.93 (13.79 – 21.48; 6.75) | 1 |
| **% time COx-a_R > 0.3** | 22.01 (17.31 – 29.73; 8.56) |  | 17.72 (14.32 – 21.37; 6.05) |  | 20.22 (14.45 – 20.44; 8.56) |  |
| **Physiologic Results for 5-Minute Data Resolution** | | | | | | |
| **Physiologic Variable** | **TBI-GL** | | **TBI-GR** | | **TBI-BLR** | |
|  | **Median (IQR) or  Median (IQR; MAD)** | **p-value** | **Median (IQR) or  Median (IQR; MAD)** | **p-value** | **Median (IQR) or  Median (IQR; MAD)** | **p-value** |
| **ABP (mmHg)** | 83.24 (76.75 – 90.4) | – | 79.56 (76.61 – 82.61) | – | 88.08 (83.18 – 96.03) | – |
| **CPP (mmHg)** | 73.62 (68.33 – 80.41) |  | 72.71 (65.71 – 80.19) |  | 73.78 (68.64 – 84.02) |  |
| **rSO_2__L (%)** | 66.99 (60.99 – 72.14) | 0.7748 | 73.98 (70.04 – 79.42) | 1 | 66.44 (61.67 – 74.69) | 0.0952 |
| **rSO_2__R (%)** | 69.33 (64.2 – 73.17) |  | 76.56 (72.82 – 79.06) |  | 72.64 (68.43 – 77.19) |  |
| **COx_L (au)** | 0.03 (-0.13 – 0.24) | 0.7130 | 0.05 (-0.1 – 0.19) | 0.1330 | 0 (-0.17 – 0.15) | 0.8413 |
| **COx_R (au)** | 0.03 (-0.13 – 0.23) |  | -0.05 (-0.21 – 0.14) |  | 0 (-0.17 – 0.18) |  |
| **COx-a_L (au)** | 0.08 (-0.09 – 0.26) | 0.4363 | 0.04 (-0.11 – 0.2) | 0.5190 | -0.01 (-0.16 – 0.16) | 0.6905 |
| **COx-a_R (au)** | 0.04 (-0.1 – 0.23) |  | 0.01 (-0.16 – 0.19) |  | 0.01 (-0.14 – 0.19) |  |
| **MAD of ABP (mmHg)** | 9.66 (7.71 – 11.45) | – | 7.04 (5.53 – 7.77) | – | 8.73 (6.9 – 9.59) | – |
| **MAD of CPP (mmHg)** | 10.11 (7.35 – 10.81) |  | 7.28 (5.59 – 9.11) |  | 9.17 (9.12 – 10.78) |  |
| **MAD of rSO_2__L (%)** | 5.29 (4.08 – 7.78) | 0.1485 | 3.64 (3.08 – 5.6) | 0.4009 | 5.25 (2.8 – 6.94) | 0.4206 |
| **MAD of rSO_2__R (%)** | 3.8 (3.16 – 6.55) |  | 4.27 (3.89 – 7.87) |  | 6.84 (4.51 – 7.52) |  |
| **MAD of COx_L (au)** | 0.27 (0.26 – 0.3) | 0.3046 | 0.24 (0.23 – 0.25) | 0.0557 | 0.26 (0.24 – 0.26) | 1 |
| **MAD of COx_R (au)** | 0.26 (0.23 – 0.28) |  | 0.28 (0.25 – 0.29) |  | 0.26 (0.24 – 0.27) |  |
| **MAD of COx-a_L (au)** | 0.26 (0.25 – 0.28) | 0.1064 | 0.24 (0.23 – 0.26) | 0.6994 | 0.25 (0.22 – 0.26) | 1 |
| **MAD of COx-a_R (au)** | 0.24 (0.22 – 0.27) |  | 0.23 (0.22 – 0.28) |  | 0.24 (0.22 – 0.26) |  |
| **% time rSO_2__L > 60%** | 82.51 (74.54 – 95.63; 13.99) | 0.3291 | 99.61 (97.92 – 100; 0.58) | 0.2670 | 85.08 (72.8 – 96.91; 18.21) | 0.3096 |
| **% time rSO_2__R > 60%** | 95.12 (72.72 – 99.77; 7.23) |  | 98.17 (93.63 – 99.9; 2.71) |  | 96.62 (93.39 – 99.88; 4.84) |  |
| **% time rSO_2__L > 70%** | 34.16 (12.54 – 63.77; 41.59) | 0.4805 | 75.21 (44.29 – 100; 36.75) | 0.9468 | 43.66 (29.27 – 44.2; 5.74) | 0.0952 |
| **% time rSO_2__R > 70%** | 46.21 (16.78 – 64.47; 31.42) |  | 84.43 (39.37 – 99.8; 23.08) |  | 66.44 (44.61 – 83.47; 32.38) |  |
| **% time rSO_2__L > 80%** | 0.56 (0.21 – 4.5; 0.84) | 0.9665 | 20.12 (2.08 – 55.04; 29.83) | 0.8430 | 0.41 (0 – 7.04; 0.61) | 0.2904 |
| **% time rSO_2__R > 80%** | 0.8 (0 – 23.91; 1.18) |  | 17.27 (6.72 – 57.81; 25.61) |  | 12.75 (5.32 – 29.15; 18.9) |  |
| **% time rSO_2__L > 90%** | 0 (0 – 0; 0) | 0.5383 | 0 (0 – 8.55; 0) | 0.8490 | 0 (0 – 0; 0) | 0.4407 |
| **% time rSO_2__R > 90%** | 0 (0 – 0.92; 0) |  | 0 (0 – 1.79; 0) |  | 0 (0 – 2.57; 0) |  |
| **% time COx_L > 0** | 55.46 (50.23 – 62.13; 9.27) | 0.7748 | 58.06 (48.01 – 62.44; 9.87) | 0.2170 | 49.07 (41.1 – 53.51; 11.81) | 0.8413 |
| **% time COx_R > 0** | 54.88 (50.95 – 65.28; 6.88) |  | 41.57 (37.74 – 55.12; 10.09) |  | 50.22 (48.8 – 52.32; 3.12) |  |
| **% time COx_L > 0.2** | 29.78 (20.85 – 33.22; 11.96) | 1 | 23.94 (18.86 – 32.2; 12.03) | 0.5190 | 18.74 (17.47 – 26.42; 8.91) | 0.8413 |
| **% time COx_R > 0.2** | 28.35 (21.44 – 32.64; 9.48) |  | 19.31 (15.99 – 26.35; 5.9) |  | 22.67 (21.83 – 22.76; 1.23) |  |
| **% time COx_L > 0.3** | 20.54 (12.19 – 21.6; 8.82) | 0.6827 | 15.48 (10.47 – 19.5; 7.53) | 0.6063 | 11.2 (9.83 – 17.39; 5.49) | 1 |
| **% time COx_R > 0.3** | 17.93 (11.24 – 22.45; 9.48) |  | 12.82 (9.6 – 17.01; 5.41) |  | 13.59 (11.04 – 15.47; 3.79) |  |
| **% time COx-a_L > 0** | 62.78 (57.99 – 64.82; 4.13) | 0.6236 | 58.88 (48.86 – 64.53; 10.11) | 0.3653 | 49.06 (49.02 – 51.16; 2.5) | 0.6905 |
| **% time COx-a_R > 0** | 57.58 (52.57 – 70.37; 12.39) |  | 52.01 (46 – 58.55; 10.24) |  | 52.06 (50.4 – 55.52; 5.13) |  |
| **% time COx-a_L > 0.2** | 32.9 (28.78 – 36.21; 5.59) | 0.3245 | 25.64 (18.8 – 33.37; 13.37) | 0.8977 | 22.07 (19.29 – 23.59; 4.12) | 0.8413 |
| **% time COx-a_R > 0.2** | 27.83 (22.43 – 39.47; 12.92) |  | 23.45 (18.33 – 27.66; 7.62) |  | 24.93 (20.35 – 25.91; 6.79) |  |
| **% time COx-a_L > 0.3** | 22.3 (16.68 – 24.02; 3.78) | 0.4363 | 12.82 (9.26 – 19.35; 6.21) | 0.5619 | 13.36 (8.96 – 16.94; 6.53) | 1 |
| **% time COx-a_R > 0.3** | 18.72 (13.48 – 25.51; 8.43) |  | 14.97 (10.47 – 17.07; 5.75) |  | 16.02 (10.53 – 19.36; 8.14) |  |
| *The p-values in the table are derived using Mann-Whitney U test between the bilateral signals.*  *ABP, arterial blood pressure; au, arbitrary units; CPP, cerebral perfusion pressure; COx, cerebral oximetry index with CPP; COx-a, cerebral oximetry index with ABP; MAD, median absolute deviation; IQR, interquartile range; mmHg, millimeters of mercury; rSO_2_, regional cerebral oxygen saturation; TBI-BLR, traumatic brain injury patient group with bifrontal lobe pathology; TBI-GL, traumatic brain injury patient group without left frontal lobe pathology; TBI-GR, traumatic brain injury patient group without right frontal lobe pathology.* | | | | | | |
