# Supplementary material for: Commercial NIRS May Not Detect Hemispheric Regional Disparity in Continuously Measured COx/COx-a: An Exploratory Healthy and Cranial Trauma Time-Series Analysis
Source: Bioengineering (Basel). 2025 Feb 28;12(3):247. doi: 10.3390/bioengineering12030247 (PMC11939202; doi:10.3390/bioengineering12030247)
Supplement: Supplementary file 1 [file bioengineering-12-00247-s001.zip › File S4.docx]

**File S4 – Absolute Regional Hemispheric Disparity (ARHD) and Median Absolute Deviation (MAD) Analysis**

File S4 – Table of Contents

[File S4a: Absolute Regional Hemispheric Disparity Analysis on 1-Minute and 5-Minute Data Resolutions for HC, SP, and TBI-GLR Populations 2](#_Toc191507547)

[File S4b: Absolute Regional Hemispheric Disparity Analysis using 10-Second, 1-Minute, and 5-Minute Data Resolutions for TBI-GL, TBI-GR, and TBI-BLR Populations 3](#_Toc191507548)

File S4a: Absolute Regional Hemispheric Disparity Analysis on 1-Minute and 5-Minute Data Resolutions for HC, SP, and TBI-GLR Populations

| **Physiologic Results for 1-Minute Data Resolution** | | | |
| --- | --- | --- | --- |
| **Physiologic Variable** | **Median (IQR)** | | |
|  | **HC** | **SP** | **TBI-GLR** |
| **ARHD of rSO_2_ (%)** | 3.9 (2.95 – 4.69) | 4.35 (3.01 – 5.53) | 5.1 (2.53 – 8.11) |
| **ARHD of COx (au)** | – | – | 0.16 (0.07 – 0.3) |
| **ARHD of COx-a (au)** | 0.14 (0.07 – 0.25) | 0.15 (0.06 – 0.3) | 0.16 (0.07 – 0.3) |
| **MAD of ARHD rSO_2_ (%)** | 0.91 (0.6 – 1.37) | 1.43 (0.89 – 2.08) | 3.1 (1.97 – 4.44) |
| **MAD of ARHD COx (au)** | – | – | 0.15 (0.13 – 0.18) |
| **MAD of ARHD COx-a (au)** | 0.12 (0.09 – 0.16) | 0.15 (0.12 – 0.18) | 0.15 (0.13 – 0.17) |
| **Physiologic Results for 5-Minute Data Resolution** | | | |
| **Physiologic Variable** | **Median (IQR)** | | |
|  | **HC** | **SP** | **TBI-GLR** |
| **ARHD of rSO_2_ (%)** | 3.83 (3.28 – 4.36) | 4.35 (3.08 – 5.51) | 5.11 (2.51 – 8.11) |
| **ARHD of COx (au)** | – | – | 0.14 (0.06 – 0.26) |
| **ARHD of COx-a (au)** | 0.13 (0.08 – 0.2) | 0.15 (0.07 – 0.26) | 0.14 (0.06 – 0.26) |
| **MAD of ARHD rSO_2_ (%)** | 0.57 (0.37 – 0.96) | 1.34 (0.81 – 1.9) | 3.05 (1.98 – 4.41) |
| **MAD of ARHD COx (au)** | – | – | 0.13 (0.11 – 0.15) |
| **MAD of ARHD COx-a (au)** | 0.09 (0.05 – 0.14) | 0.13 (0.1 – 0.15) | 0.13 (0.11 – 0.15) |
| *ARHD, absolute regional hemispheric difference; au, arbitrary units; COx, cerebral oximetry index with cerebral perfusion pressure; COx-a, cerebral oximetry index with arterial blood pressure; HC, healthy control volunteer group; IQR, interquartile range; MAD, median absolute deviation; rSO_2_, regional cerebral oxygen saturation; SP, elective spinal surgery patient group; TBI-GLR, traumatic brain injury patient group without bifrontal lobe pathology.* | | | |

File S4b: Absolute Regional Hemispheric Disparity Analysis using 10-Second, 1-Minute, and 5-Minute Data Resolutions for TBI-GL, TBI-GR, and TBI-BLR Populations

| **Physiologic Results for 10-Second Data Resolution** | | | |
| --- | --- | --- | --- |
| **Physiologic Variable** | **Median (IQR)** | | |
|  | **TBI-GL** | **TBI-GR** | **TBI-BLR** |
| **ARHD of rSO_2_ (%)** | 7 (4.7 – 10.83) | 6.08 (3.63 – 8.77) | 5.42 (2.99 – 7.59) |
| **ARHD of COx (au)** | 0.18 (0.08 – 0.33) | 0.17 (0.07 – 0.35) | 0.17 (0.07 – 0.31) |
| **ARHD of COx-a (au)** | 0.18 (0.08 – 0.33) | 0.17 (0.07 – 0.32) | 0.16 (0.07 – 0.3) |
| **MAD of ARHD rSO_2_ (%)** | 3.8 (2.99 – 5.31) | 3.71 (1.85 – 4.15) | 2.59 (2.22 – 3.07) |
| **MAD of ARHD COx (au)** | 0.17 (0.16 – 0.2) | 0.18 (0.17 – 0.21) | 0.16 (0.15 – 0.16) |
| **MAD of ARHD COx-a (au)** | 0.17 (0.16 – 0.19) | 0.17 (0.16 – 0.18) | 0.16 (0.15 – 0.16) |
| **Physiologic Results for 1-Minute Data Resolution** | | | |
| **Physiologic Variable** | **Median (IQR)** | | |
|  | **TBI-GL** | **TBI-GR** | **TBI-BLR** |
| **ARHD of rSO_2_ (%)** | 7 (4.6 – 10.72) | 6.24 (3.65 – 8.73) | 5.39 (2.63 – 7.58) |
| **ARHD of COx (au)** | 0.18 (0.09 – 0.32) | 0.19 (0.08 – 0.34) | 0.16 (0.07 – 0.3) |
| **ARHD of COx-a (au)** | 0.18 (0.08 – 0.32) | 0.17 (0.07 – 0.31) | 0.15 (0.07 – 0.29) |
| **MAD of ARHD rSO_2_ (%)** | 3.8 (3.1 – 5.25) | 3.76 (1.82 – 4.08) | 2.52 (2.17 – 2.99) |
| **MAD of ARHD COx (au)** | 0.17 (0.15 – 0.19) | 0.18 (0.16 – 0.21) | 0.15 (0.15 – 0.16) |
| **MAD of ARHD COx-a (au)** | 0.17 (0.16 – 0.19) | 0.16 (0.16 – 0.18) | 0.15 (0.14 – 0.15) |
| **Physiologic Results for 5-Minute Data Resolution** | | | |
| **Physiologic Variable** | **Median (IQR)** | | |
|  | **TBI-GL** | **TBI-GR** | **TBI-BLR** |
| **ARHD of rSO_2_ (%)** | 7.01 (4.66 – 10.69) | 6.27 (3.64 – 8.67) | 5.35 (2.65 – 7.53) |
| **ARHD of COx (au)** | 0.16 (0.07 – 0.28) | 0.16 (0.07 – 0.3) | 0.15 (0.06 – 0.26) |
| **ARHD of COx-a (au)** | 0.16 (0.07 – 0.28) | 0.15 (0.07 – 0.26) | 0.14 (0.07 – 0.25) |
| **MAD of ARHD rSO_2_ (%)** | 3.79 (3.13 – 5.29) | 3.83 (1.78 – 4.03) | 2.46 (2.16 – 2.94) |
| **MAD of ARHD COx (au)** | 0.14 (0.13 – 0.17) | 0.15 (0.14 – 0.18) | 0.13 (0.13 – 0.15) |
| **MAD of ARHD COx-a (au)** | 0.15 (0.14 – 0.16) | 0.13 (0.13 – 0.15) | 0.13 (0.12 – 0.15) |
| *ARHD, absolute regional hemispheric difference; au, arbitrary units; COx, cerebral oximetry index with cerebral perfusion pressure; COx-a, cerebral oximetry index with arterial blood pressure; IQR, interquartile range; MAD, median absolute deviation; rSO_2_, regional cerebral oxygen saturation; TBI-BLR, traumatic brain injury patient group with bifrontal lobe pathology; TBI-GL, traumatic brain injury patient group without left frontal lobe pathology; TBI-GR, traumatic brain injury patient group without right frontal lobe pathology.* | | | |
