# Supplementary material for: Commercial NIRS May Not Detect Hemispheric Regional Disparity in Continuously Measured COx/COx-a: An Exploratory Healthy and Cranial Trauma Time-Series Analysis
Source: Bioengineering (Basel). 2025 Feb 28;12(3):247. doi: 10.3390/bioengineering12030247 (PMC11939202; doi:10.3390/bioengineering12030247)
Supplement: Supplementary file 1 [file bioengineering-12-00247-s001.zip › File S5.docx]

**File S5 – Time-Series Stationarity and Autoregressive Integrative Moving Average (ARIMA) Analysis**

File S5 – Table of Contents

[File S5a: ADF and KPSS P-Values for Non-Differenced and 1^st^ Order Differenced in 1-Minute Data Resolution – HC Volunteers 2](#_Toc191507774)

[File S5b: ADF and KPSS P-Values for Non-Differenced and 1^st^ Order Differenced 1-Minute Data Resolution – SP Patients 5](#_Toc191507775)

[File S5c: ADF and KPSS P-Values for Non-Differenced and 1^st^ Order Differenced 1-Minute Data Resolution – TBI-GLR Patients 6](#_Toc191507776)

[File S5d: ADF and KPSS P-Values for Non-Differenced and 1^st^ Order Differenced 1-Minute Data Resolution – TBI-GL Patients 8](#_Toc191507777)

[File S5e: ADF and KPSS P-Values for Non-Differenced and 1^st^ Order Differenced 1-Minute Data Resolution – TBI-GR Patients 9](#_Toc191507778)

[File S5f: ADF and KPSS P-Values for Non-Differenced and 1^st^ Order Differenced 1-Minute Data Resolution – TBI-BLR Patients 10](#_Toc191507779)

[File S5g: Recorded AIC while fitting various ARIMA models – TBI-GLR Patient Example 11](#_Toc191507780)

[File S5h: Personalized ARIMA models P-Order and Q-Order based on AIC – TBI-GLR Patient Example 13](#_Toc191507781)

[File S5i: ADF and KPSS results showing stationary vs non-stationary vs NA for physiologic signals – Original and 1st order differenced HC, SP, and TBI-GLR Data 14](#_Toc191507782)

[File S5j: ADF and KPSS results showing stationary vs non-stationary vs NA for physiologic signals – Original and 1st order differenced TBI-GL, TBI-GR, and TBI-BLR Data 15](#_Toc191507783)

[File S5k: Personalized ARIMA P-Orders based on AIC for HC, SP, and TBI-GLR Populations – 10-Second Data Resolution Example 16](#_Toc191507784)

[File S5l: Personalized ARIMA P-Orders based on for TBI-GL, TBI-GR, and TBI-BLR Populations – 10-Second Data Resolution Example 18](#_Toc191507785)

[File S5m: Personalized ARIMA Q-Orders based on AIC for HC, SP, and TBI-GLR Populations – 10-Second Data Resolution Example 19](#_Toc191507786)

[File S5n: Personalized ARIMA Q-Orders based on AIC for TBI-GL, TBI-GR, and TBI-BLR Populations – 10-Second Data Resolution Example 21](#_Toc191507787)

[File S5o: Regional Hemispheric Disparity of Personalized ARIMA p-orders based on AIC for HC, SP, and TBI-GLR Populations – 10-Second Data Resolution Example 22](#_Toc191507788)

[File S5p: Regional Hemispheric Disparity of Personalized ARIMA P-Orders based on AIC in 10-Second Data Resolution for TBI-GL, TBI-GR, and TBI-BLR Populations 24](#_Toc191507789)

[File S5q: Regional Hemispheric Disparity of Personalized ARIMA Q-Orders based on AIC for HC, SP, and TBI-GLR Populations – 10-Second Data Resolution Example 25](#_Toc191507790)

[File S5r: Regional Hemispheric Disparity of Personalized ARIMA Q-Orders based on AIC for TBI-GL, TBI-GR, and TBI-BLR Populations – 10-Second Data Resolution Example 27](#_Toc191507791)

[File S5s: Personalized ARIMA P-Orders and Q-Orders based on AIC and their Hemispheric Disparity in 1-Minute and 5-Minute Data Resolutions for HC, SP, and TBI-GLR Populations 28](#_Toc191507792)

[File S5t: Personalized ARIMA P-Orders and Q-Orders based on AIC and their Hemispheric Disparity in 10-Second, 1-Minute, and 5-Minute Data Resolutions for TBI-GL, TBI-GR, and TBI-BLR Populations 29](#_Toc191507793)

File S5a: ADF and KPSS P-Values for Non-Differenced and 1^st^ Order Differenced in 1-Minute Data Resolution – HC Volunteers

| **ADF p-values for 1-minute data resolution** | | | | | | | | | | |
| --- | --- | --- | --- | --- | --- | --- | --- | --- | --- | --- |
| **Patient** | ***Non-Differenced*** | | | | | ***1^st^ Order Differenced*** | | | | |
|  | **ABP** | **rSO2_L** | **rSO2_R** | **COx-a_L** | **COx-a_R** | **ABP** | **rSO2_L** | **rSO2_R** | **COx-a_L** | **COx-a_R** |
| 1 | 0.08 | 0.45 | 0.37 | 0.34 | 0.47 | 0.01 | 0.01 | 0.01 | 0.09 | 0.19 |
| 2 | 0.59 | 0.50 | 0.24 | 0.09 | 0.36 | 0.02 | 0.01 | 0.01 | 0.03 | 0.04 |
| 3 | 0.37 | 0.71 | 0.70 | 0.40 | 0.60 | 0.01 | 0.04 | 0.01 | 0.01 | 0.31 |
| 4 | 0.44 | 0.01 | 0.09 | 0.01 | 0.01 | 0.02 | 0.01 | 0.01 | 0.01 | 0.01 |
| 5 | 0.57 | 0.84 | 0.35 | 0.56 | 0.90 | 0.07 | 0.02 | 0.01 | 0.02 | 0.01 |
| 6 | 0.17 | 0.40 | 0.03 | 0.58 | 0.82 | 0.09 | 0.01 | 0.01 | 0.21 | 0.23 |
| 7 | 0.06 | 0.06 | 0.25 | 0.02 | 0.01 | 0.02 | 0.01 | 0.01 | 0.01 | 0.01 |
| 8 | 0.43 | 0.05 | 0.10 | 0.01 | 0.01 | 0.05 | 0.21 | 0.01 | 0.01 | 0.01 |
| 9 | 0.01 | 0.27 | 0.37 | 0.01 | 0.43 | 0.01 | 0.02 | 0.01 | 0.01 | 0.06 |
| 10 | 0.48 | 0.22 | 0.13 | 0.44 | 0.31 | 0.19 | 0.02 | 0.02 | 0.22 | 0.25 |
| 11 | 0.15 | 0.26 | 0.39 | 0.22 | 0.43 | 0.01 | 0.01 | 0.04 | 0.02 | 0.01 |
| 12 | 0.01 | 0.46 | 0.52 | 0.39 | 0.40 | 0.01 | 0.01 | 0.01 | 0.04 | 0.01 |
| 13 | 0.63 | 0.17 | 0.02 | 0.21 | 0.33 | 0.01 | 0.01 | 0.01 | 0.02 | 0.08 |
| 14 | 0.34 | 0.96 | 0.54 | 0.06 | 0.02 | 0.01 | 0.01 | 0.01 | 0.09 | 0.03 |
| 15 | 0.63 | 0.34 | 0.37 | 0.01 | 0.01 | 0.01 | 0.01 | 0.01 | 0.01 | 0.01 |
| 16 | 0.36 | 0.64 | 0.40 | 0.12 | 0.01 | 0.13 | 0.18 | 0.01 | 0.06 | 0.15 |
| 17 | 0.89 | 0.19 | 0.46 | 0.30 | 0.10 | 0.02 | 0.01 | 0.01 | 0.02 | 0.03 |
| 18 | 0.43 | 0.21 | 0.01 | 0.29 | 0.24 | 0.01 | 0.01 | 0.01 | 0.01 | 0.02 |
| 19 | 0.30 | 0.62 | 0.89 | 0.06 | 0.30 | 0.01 | 0.01 | 0.01 | 0.06 | 0.09 |
| 20 | 0.02 | 0.50 | 0.47 | 0.34 | 0.26 | 0.01 | 0.02 | 0.09 | 0.01 | 0.03 |
| 21 | 0.43 | 0.03 | 0.03 | 0.12 | 0.17 | 0.01 | 0.01 | 0.01 | 0.01 | 0.01 |
| 22 | 0.02 | 0.01 | 0.05 | 0.95 | 0.85 | 0.01 | 0.01 | 0.01 | 0.01 | 0.06 |
| 23 | 0.15 | 0.54 | 0.24 | 0.27 | 0.54 | 0.01 | 0.01 | 0.01 | 0.03 | 0.01 |
| 24 | 0.60 | 0.48 | 0.01 | 0.48 | 0.84 | 0.10 | 0.34 | 0.01 | 0.26 | 0.40 |
| 25 | 0.89 | 0.52 | 0.49 | 0.31 | 0.47 | 0.01 | 0.01 | 0.01 | 0.01 | 0.01 |
| 26 | 0.45 | 0.47 | 0.17 | 0.08 | 0.01 | 0.03 | 0.01 | 0.15 | 0.01 | 0.01 |
| 27 | 0.70 | 0.01 | 0.02 | 0.01 | 0.01 | 0.01 | 0.01 | 0.01 | 0.01 | 0.01 |
| 28 | 0.21 | 0.43 | 0.13 | 0.04 | 0.17 | 0.01 | 0.07 | 0.05 | 0.07 | 0.04 |
| 29 | 0.73 | 0.39 | 0.03 | 0.25 | 0.10 | 0.05 | 0.01 | 0.05 | 0.07 | 0.23 |
| 30 | 0.24 | 0.21 | 0.07 | 0.02 | 0.04 | 0.01 | 0.01 | 0.01 | 0.02 | 0.03 |
| 31 | 0.21 | 0.08 | 0.06 | 0.04 | 0.02 | 0.01 | 0.01 | 0.01 | 0.01 | 0.01 |
| 32 | 0.01 | 0.02 | 0.19 | 0.45 | 0.46 | 0.01 | 0.01 | 0.01 | 0.24 | 0.10 |
| 33 | 0.01 | 0.66 | 0.25 | 0.37 | 0.58 | 0.01 | 0.01 | 0.01 | 0.01 | 0.09 |
| 34 | 0.34 | 0.46 | 0.52 | 0.39 | 0.56 | 0.09 | 0.13 | 0.12 | 0.43 | 0.29 |
| 35 | 0.52 | 0.63 | 0.46 | 0.01 | 0.07 | 0.01 | 0.01 | 0.02 | 0.01 | 0.01 |
| 36 | 0.34 | 0.59 | 0.12 | 0.01 | 0.24 | 0.02 | 0.02 | 0.01 | 0.01 | 0.06 |
| 37 | 0.98 | 0.41 | 0.48 | 0.01 | 0.03 | 0.05 | 0.02 | 0.02 | 0.32 | 0.37 |
| 38 | 0.02 | 0.13 | 0.32 | 0.19 | 0.10 | 0.01 | 0.01 | 0.01 | 0.04 | 0.04 |
| 39 | 0.08 | 0.57 | 0.01 | 0.37 | 0.01 | 0.01 | 0.01 | 0.01 | 0.09 | 0.01 |
| 40 | 0.08 | 0.72 | 0.07 | 0.51 | 0.63 | 0.01 | 0.01 | 0.01 | 0.04 | 0.25 |
| 41 | 0.09 | 0.54 | 0.04 | 0.01 | 0.04 | 0.01 | 0.05 | 0.01 | 0.01 | 0.01 |
| 42 | 0.01 | 0.06 | 0.60 | 0.10 | 0.10 | 0.01 | 0.01 | 0.01 | 0.08 | 0.01 |
| 43 | 0.32 | 0.02 | 0.08 | 0.47 | 0.07 | 0.01 | 0.01 | 0.29 | 0.58 | 0.37 |
| 44 | 0.46 | 0.46 | 0.01 | 0.01 | 0.01 | 0.01 | 0.01 | 0.01 | 0.07 | 0.03 |
| 45 | 0.26 | 0.36 | 0.47 | 0.25 | 0.56 | 0.01 | 0.01 | 0.01 | 0.03 | 0.02 |
| 46 | 0.59 | 0.22 | 0.01 | 0.05 | 0.11 | 0.21 | 0.09 | 0.01 | 0.04 | 0.30 |
| 47 | 0.95 | 0.26 | 0.29 | 0.27 | 0.14 | 0.01 | 0.01 | 0.01 | 0.01 | 0.04 |
| 48 | 0.77 | 0.01 | 0.20 | 0.02 | 0.04 | 0.01 | 0.01 | 0.01 | 0.02 | 0.04 |
| 49 | 0.62 | 0.07 | 0.48 | 0.20 | 0.02 | 0.02 | 0.01 | 0.01 | 0.02 | 0.01 |
| 50 | 0.01 | 0.14 | 0.62 | 0.03 | 0.36 | 0.01 | 0.01 | 0.01 | 0.04 | 0.01 |
| 51 | 0.02 | 0.04 | 0.26 | 0.01 | 0.17 | 0.01 | 0.01 | 0.01 | 0.01 | 0.01 |
| 52 | 0.05 | 0.03 | 0.04 | 0.09 | 0.58 | 0.01 | 0.01 | 0.01 | 0.19 | 0.48 |
| 53 | 0.98 | 0.06 | 0.95 | 0.70 | 0.31 | 0.02 | 0.01 | 0.02 | 0.03 | 0.02 |
| 54 | 0.29 | 0.18 | 0.17 | 0.20 | 0.75 | 0.01 | 0.03 | 0.01 | 0.27 | 0.34 |
| 55 | 0.05 | 0.12 | 0.80 | 0.45 | 0.03 | 0.01 | 0.01 | 0.16 | 0.09 | 0.01 |
| 56 | 0.30 | 0.22 | 0.17 | 0.25 | 0.23 | 0.01 | 0.01 | 0.01 | 0.01 | 0.10 |
| 57 | 0.53 | 0.68 | 0.22 | 0.01 | 0.04 | 0.05 | 0.01 | 0.01 | 0.02 | 0.01 |
| 58 | 0.07 | 0.77 | 0.01 | 0.91 | 0.17 | 0.01 | 0.05 | 0.32 | 0.06 | 0.02 |
| 59 | 0.03 | 0.59 | 0.11 | 0.61 | 0.71 | 0.01 | 0.57 | 0.07 | 0.31 | 0.67 |
| 60 | 0.44 | 0.99 | 0.82 | 0.10 | 0.40 | 0.01 | 0.06 | 0.02 | 0.05 | 0.03 |
| 61 | 0.43 | 0.01 | 0.01 | 0.03 | 0.19 | 0.01 | 0.01 | 0.01 | 0.02 | 0.01 |
| 62 | 0.18 | 0.03 | 0.55 | 0.28 | 0.08 | 0.01 | 0.01 | 0.01 | 0.34 | 0.02 |
| 63 | 0.02 | 0.59 | 0.66 | 0.22 | 0.20 | 0.01 | 0.02 | 0.43 | 0.05 | 0.02 |
| 64 | 0.08 | 0.21 | 0.13 | 0.35 | 0.49 | 0.01 | 0.10 | 0.06 | 0.25 | 0.04 |
| 65 | 0.26 | 0.22 | 0.46 | 0.03 | 0.01 | 0.01 | 0.01 | 0.01 | 0.01 | 0.01 |
| 66 | 0.20 | 0.36 | 0.55 | 0.57 | 0.66 | 0.02 | 0.17 | 0.10 | 0.04 | 0.09 |
| 67 | 0.10 | 0.03 | 0.30 | 0.42 | 0.11 | 0.01 | 0.01 | 0.01 | 0.02 | 0.01 |
| 68 | 0.38 | 0.20 | 0.49 | 0.06 | 0.07 | 0.03 | 0.04 | 0.01 | 0.01 | 0.04 |
| 69 | 0.63 | 0.38 | 0.44 | 0.68 | 0.25 | 0.02 | 0.01 | 0.01 | 0.01 | 0.01 |
| 70 | 0.60 | 0.47 | 0.12 | 0.49 | 0.26 | 0.01 | 0.01 | 0.01 | 0.03 | 0.09 |
| 71 | 0.10 | 0.36 | 0.72 | 0.52 | 0.59 | 0.01 | 0.01 | 0.01 | 0.10 | 0.06 |
| 72 | 0.01 | 0.74 | 0.05 | 0.30 | 0.27 | 0.01 | 0.01 | 0.02 | 0.06 | 0.01 |
| 73 | 0.48 | 0.26 | 0.28 | 0.05 | 0.09 | 0.01 | 0.01 | 0.03 | 0.02 | 0.02 |
| 74 | 0.31 | 0.18 | 0.16 | 0.26 | 0.15 | 0.01 | 0.01 | 0.01 | 0.01 | 0.01 |
| 75 | 0.10 | 0.01 | 0.04 | 0.02 | 0.09 | 0.01 | 0.01 | 0.01 | 0.01 | 0.01 |
| 76 | 0.35 | 0.92 | 0.92 | 0.05 | 0.04 | 0.01 | 0.02 | 0.05 | 0.04 | 0.18 |
| 77 | 0.08 | 0.01 | 0.04 | 0.37 | 0.27 | 0.02 | 0.01 | 0.01 | 0.31 | 0.01 |
| 78 | 0.53 | 0.90 | 0.44 | 0.06 | 0.05 | 0.01 | 0.09 | 0.01 | 0.04 | 0.24 |
| 79 | 0.35 | 0.89 | 0.82 | 0.04 | 0.34 | 0.03 | 0.01 | 0.01 | 0.01 | 0.01 |
| 80 | 0.60 | 0.44 | 0.43 | 0.03 | 0.02 | 0.02 | 0.01 | 0.04 | 0.05 | 0.03 |
| 81 | 0.46 | 0.89 | 0.62 | 0.14 | 0.04 | 0.05 | 0.92 | 0.83 | 0.03 | 0.01 |
| 82 | 0.01 | 0.39 | 0.47 | 0.92 | 0.58 | 0.01 | 0.04 | 0.01 | 0.02 | 0.40 |
| 83 | 0.57 | 0.34 | 0.17 | 0.24 | 0.29 | 0.02 | 0.01 | 0.01 | 0.05 | 0.03 |
| 84 | 0.31 | 0.77 | 0.46 | 0.10 | 0.08 | 0.01 | 0.01 | 0.01 | 0.02 | 0.01 |
| 85 | 0.42 | 0.11 | 0.04 | 0.39 | 0.48 | 0.04 | 0.01 | 0.01 | 0.22 | 0.01 |
| 86 | 0.47 | 0.86 | 0.99 | 0.30 | 0.36 | 0.01 | 0.01 | 0.08 | 0.50 | 0.49 |
| 87 | 0.05 | 0.74 | 0.54 | 0.42 | 0.17 | 0.01 | 0.01 | 0.01 | 0.21 | 0.06 |
| 88 | 0.07 | 0.43 | 0.04 | 0.77 | 0.32 | 0.10 | 0.02 | 0.02 | 0.02 | 0.01 |
| 89 | 0.45 | 0.30 | 0.15 | 0.30 | 0.20 | 0.01 | 0.01 | 0.01 | 0.10 | 0.09 |
| 90 | 0.01 | 0.01 | 0.04 | 0.50 | 0.02 | 0.01 | 0.01 | 0.01 | 0.30 | 0.02 |
| 91 | 0.64 | 0.28 | 0.31 | 0.08 | 0.06 | 0.01 | 0.01 | 0.02 | 0.01 | 0.01 |
| 92 | 0.68 | 0.27 | 0.11 | 0.10 | 0.07 | 0.02 | 0.01 | 0.01 | 0.01 | 0.01 |
| 93 | 0.08 | 0.22 | 0.19 | 0.40 | 0.02 | 0.01 | 0.01 | 0.01 | 0.01 | 0.04 |
| 94 | 0.21 | 0.47 | 0.08 | 0.08 | 0.24 | 0.01 | 0.01 | 0.01 | 0.06 | 0.18 |
| 95 | 0.59 | 0.93 | 0.95 | 0.34 | 0.01 | 0.01 | 0.04 | 0.07 | 0.08 | 0.01 |
| 96 | 0.42 | 0.18 | 0.08 | 0.04 | 0.03 | 0.03 | 0.01 | 0.01 | 0.01 | 0.03 |
| 97 | 0.48 | 0.05 | 0.02 | 0.01 | 0.01 | 0.01 | 0.01 | 0.01 | 0.01 | 0.01 |
| 98 | 0.60 | 0.60 | 0.79 | 0.39 | 0.27 | 0.02 | 0.01 | 0.01 | 0.38 | 0.40 |
| 99 | 0.28 | 0.07 | 0.01 | 0.48 | 0.43 | 0.01 | 0.01 | 0.01 | 0.01 | 0.13 |
| 100 | 0.15 | 0.13 | 0.46 | 0.33 | 0.26 | 0.01 | 0.01 | 0.05 | 0.07 | 0.17 |
| 101 | 0.27 | 0.18 | 0.42 | 0.18 | 0.56 | 0.01 | 0.04 | 0.01 | 0.01 | 0.03 |
| 102 | 0.01 | 0.33 | 0.04 | 0.15 | 0.03 | 0.01 | 0.01 | 0.01 | 0.01 | 0.01 |
| **KPSS p-values for 1-minute data resolution** | | | | | | | | | | |
| **Patient** | ***Non-Differenced*** | | | | | ***1^st^ Order Differenced*** | | | | |
|  | **ABP** | **rSO2_L** | **rSO2_R** | **COx-a_L** | **COx-a_R** | **ABP** | **rSO2_L** | **rSO2_R** | **COx-a_L** | **COx-a_R** |
| 1 | 0.10 | 0.10 | 0.10 | 0.02 | 0.07 | 0.10 | 0.10 | 0.10 | 0.10 | 0.10 |
| 2 | 0.02 | 0.10 | 0.10 | 0.02 | 0.02 | 0.10 | 0.10 | 0.10 | 0.10 | 0.10 |
| 3 | 0.01 | 0.10 | 0.10 | 0.10 | 0.10 | 0.10 | 0.10 | 0.10 | 0.10 | 0.10 |
| 4 | 0.02 | 0.10 | 0.04 | 0.10 | 0.10 | 0.10 | 0.10 | 0.10 | 0.10 | 0.10 |
| 5 | 0.01 | 0.01 | 0.02 | 0.10 | 0.10 | 0.10 | 0.10 | 0.10 | 0.10 | 0.05 |
| 6 | 0.10 | 0.03 | 0.10 | 0.07 | 0.10 | 0.10 | 0.10 | 0.10 | 0.10 | 0.04 |
| 7 | 0.07 | 0.10 | 0.10 | 0.10 | 0.10 | 0.10 | 0.10 | 0.10 | 0.10 | 0.10 |
| 8 | 0.10 | 0.10 | 0.02 | 0.10 | 0.10 | 0.10 | 0.10 | 0.10 | 0.10 | 0.10 |
| 9 | 0.01 | 0.01 | 0.01 | 0.01 | 0.10 | 0.10 | 0.10 | 0.10 | 0.10 | 0.10 |
| 10 | 0.10 | 0.10 | 0.01 | 0.10 | 0.10 | 0.10 | 0.10 | 0.10 | 0.10 | 0.10 |
| 11 | 0.01 | 0.09 | 0.10 | 0.10 | 0.10 | 0.10 | 0.10 | 0.10 | 0.10 | 0.10 |
| 12 | 0.10 | 0.10 | 0.02 | 0.10 | 0.10 | 0.10 | 0.10 | 0.10 | 0.10 | 0.10 |
| 13 | 0.10 | 0.05 | 0.06 | 0.10 | 0.10 | 0.10 | 0.10 | 0.10 | 0.10 | 0.10 |
| 14 | 0.10 | 0.10 | 0.10 | 0.10 | 0.10 | 0.10 | 0.09 | 0.10 | 0.10 | 0.10 |
| 15 | 0.02 | 0.01 | 0.02 | 0.04 | 0.10 | 0.10 | 0.10 | 0.10 | 0.10 | 0.10 |
| 16 | 0.10 | 0.03 | 0.01 | 0.10 | 0.10 | 0.10 | 0.10 | 0.10 | 0.09 | 0.10 |
| 17 | 0.02 | 0.03 | 0.01 | 0.10 | 0.10 | 0.10 | 0.10 | 0.10 | 0.10 | 0.10 |
| 18 | 0.10 | 0.10 | 0.04 | 0.10 | 0.10 | 0.10 | 0.10 | 0.10 | 0.10 | 0.10 |
| 19 | 0.01 | 0.01 | 0.01 | 0.10 | 0.04 | 0.10 | 0.10 | 0.10 | 0.10 | 0.10 |
| 20 | 0.01 | 0.10 | 0.10 | 0.01 | 0.10 | 0.10 | 0.10 | 0.10 | 0.10 | 0.10 |
| 21 | 0.10 | 0.10 | 0.10 | 0.10 | 0.10 | 0.10 | 0.10 | 0.10 | 0.10 | 0.10 |
| 22 | 0.01 | 0.03 | 0.01 | 0.07 | 0.10 | 0.10 | 0.10 | 0.10 | 0.10 | 0.10 |
| 23 | 0.10 | 0.10 | 0.09 | 0.10 | 0.10 | 0.10 | 0.10 | 0.07 | 0.10 | 0.10 |
| 24 | 0.04 | 0.10 | 0.08 | 0.10 | 0.10 | 0.10 | 0.10 | 0.10 | 0.10 | 0.10 |
| 25 | 0.09 | 0.03 | 0.10 | 0.10 | 0.10 | 0.10 | 0.10 | 0.10 | 0.10 | 0.10 |
| 26 | 0.10 | 0.03 | 0.10 | 0.10 | 0.10 | 0.10 | 0.10 | 0.10 | 0.10 | 0.10 |
| 27 | 0.01 | 0.01 | 0.10 | 0.01 | 0.02 | 0.10 | 0.10 | 0.10 | 0.10 | 0.10 |
| 28 | 0.02 | 0.01 | 0.01 | 0.10 | 0.10 | 0.10 | 0.10 | 0.10 | 0.10 | 0.10 |
| 29 | 0.01 | 0.10 | 0.02 | 0.10 | 0.02 | 0.10 | 0.10 | 0.10 | 0.10 | 0.10 |
| 30 | 0.10 | 0.10 | 0.02 | 0.10 | 0.09 | 0.10 | 0.10 | 0.10 | 0.10 | 0.10 |
| 31 | 0.10 | 0.10 | 0.07 | 0.06 | 0.02 | 0.10 | 0.10 | 0.10 | 0.10 | 0.10 |
| 32 | 0.01 | 0.08 | 0.10 | 0.05 | 0.10 | 0.10 | 0.10 | 0.10 | 0.10 | 0.10 |
| 33 | 0.10 | 0.10 | 0.10 | 0.10 | 0.10 | 0.10 | 0.10 | 0.10 | 0.10 | 0.10 |
| 34 | 0.01 | 0.10 | 0.10 | 0.04 | 0.05 | 0.10 | 0.10 | 0.10 | 0.10 | 0.10 |
| 35 | 0.10 | 0.10 | 0.09 | 0.10 | 0.10 | 0.10 | 0.10 | 0.10 | 0.10 | 0.10 |
| 36 | 0.10 | 0.10 | 0.04 | 0.10 | 0.10 | 0.10 | 0.10 | 0.10 | 0.10 | 0.10 |
| 37 | 0.01 | 0.02 | 0.04 | 0.10 | 0.10 | 0.07 | 0.10 | 0.10 | 0.10 | 0.10 |
| 38 | 0.10 | 0.10 | 0.09 | 0.10 | 0.10 | 0.10 | 0.10 | 0.10 | 0.10 | 0.10 |
| 39 | 0.01 | 0.02 | 0.10 | 0.10 | 0.10 | 0.10 | 0.10 | 0.10 | 0.10 | 0.10 |
| 40 | 0.01 | 0.02 | 0.04 | 0.10 | 0.10 | 0.10 | 0.10 | 0.10 | 0.10 | 0.10 |
| 41 | 0.02 | 0.01 | 0.01 | 0.10 | 0.10 | 0.10 | 0.10 | 0.10 | 0.10 | 0.10 |
| 42 | 0.01 | 0.10 | 0.01 | 0.07 | 0.10 | 0.10 | 0.10 | 0.10 | 0.10 | 0.10 |
| 43 | 0.02 | 0.02 | 0.10 | 0.10 | 0.10 | 0.10 | 0.10 | 0.10 | 0.10 | 0.10 |
| 44 | 0.10 | 0.01 | 0.01 | 0.10 | 0.10 | 0.10 | 0.10 | 0.10 | 0.10 | 0.10 |
| 45 | 0.05 | 0.10 | 0.01 | 0.10 | 0.07 | 0.10 | 0.10 | 0.10 | 0.10 | 0.10 |
| 46 | 0.10 | 0.05 | 0.10 | 0.03 | 0.10 | 0.10 | 0.10 | 0.10 | 0.10 | 0.10 |
| 47 | 0.01 | 0.01 | 0.01 | 0.10 | 0.10 | 0.10 | 0.10 | 0.10 | 0.10 | 0.10 |
| 48 | 0.10 | 0.01 | 0.10 | 0.10 | 0.10 | 0.10 | 0.10 | 0.10 | 0.10 | 0.10 |
| 49 | 0.08 | 0.02 | 0.01 | 0.10 | 0.03 | 0.05 | 0.10 | 0.10 | 0.10 | 0.10 |
| 50 | 0.10 | 0.08 | 0.10 | 0.06 | 0.10 | 0.10 | 0.10 | 0.10 | 0.10 | 0.10 |
| 51 | 0.10 | 0.08 | 0.01 | 0.10 | 0.10 | 0.10 | 0.10 | 0.10 | 0.10 | 0.10 |
| 52 | 0.10 | 0.04 | 0.06 | 0.04 | 0.10 | 0.10 | 0.10 | 0.10 | 0.10 | 0.10 |
| 53 | 0.01 | 0.10 | 0.10 | 0.10 | 0.10 | 0.09 | 0.10 | 0.09 | 0.10 | 0.10 |
| 54 | 0.04 | 0.07 | 0.01 | 0.10 | 0.10 | 0.10 | 0.10 | 0.10 | 0.10 | 0.10 |
| 55 | 0.10 | 0.10 | 0.10 | 0.10 | 0.10 | 0.10 | 0.10 | 0.10 | 0.10 | 0.10 |
| 56 | 0.10 | 0.03 | 0.02 | 0.09 | 0.10 | 0.10 | 0.10 | 0.10 | 0.10 | 0.10 |
| 57 | 0.10 | 0.10 | 0.09 | 0.02 | 0.10 | 0.10 | 0.10 | 0.10 | 0.10 | 0.10 |
| 58 | 0.10 | 0.10 | 0.10 | 0.02 | 0.10 | 0.10 | 0.10 | 0.10 | 0.10 | 0.10 |
| 59 | 0.04 | 0.10 | 0.02 | 0.10 | 0.08 | 0.10 | 0.10 | 0.10 | 0.10 | 0.10 |
| 60 | 0.10 | 0.08 | 0.10 | 0.10 | 0.10 | 0.10 | 0.07 | 0.10 | 0.10 | 0.10 |
| 61 | 0.10 | 0.08 | 0.10 | 0.10 | 0.10 | 0.10 | 0.10 | 0.10 | 0.10 | 0.10 |
| 62 | 0.10 | 0.01 | 0.10 | 0.10 | 0.10 | 0.10 | 0.10 | 0.10 | 0.10 | 0.10 |
| 63 | 0.10 | 0.02 | 0.02 | 0.08 | 0.10 | 0.10 | 0.10 | 0.10 | 0.10 | 0.10 |
| 64 | 0.10 | 0.10 | 0.10 | 0.03 | 0.04 | 0.10 | 0.10 | 0.10 | 0.10 | 0.10 |
| 65 | 0.10 | 0.10 | 0.10 | 0.03 | 0.10 | 0.10 | 0.03 | 0.10 | 0.10 | 0.10 |
| 66 | 0.10 | 0.04 | 0.10 | 0.10 | 0.10 | 0.10 | 0.10 | 0.10 | 0.10 | 0.10 |
| 67 | 0.01 | 0.01 | 0.01 | 0.10 | 0.10 | 0.10 | 0.10 | 0.10 | 0.10 | 0.10 |
| 68 | 0.02 | 0.10 | 0.07 | 0.10 | 0.10 | 0.10 | 0.10 | 0.10 | 0.10 | 0.10 |
| 69 | 0.01 | 0.01 | 0.07 | 0.10 | 0.10 | 0.10 | 0.10 | 0.10 | 0.10 | 0.10 |
| 70 | 0.04 | 0.04 | 0.10 | 0.10 | 0.10 | 0.10 | 0.10 | 0.10 | 0.10 | 0.10 |
| 71 | 0.03 | 0.01 | 0.01 | 0.09 | 0.04 | 0.10 | 0.10 | 0.10 | 0.10 | 0.10 |
| 72 | 0.10 | 0.03 | 0.01 | 0.10 | 0.04 | 0.10 | 0.10 | 0.10 | 0.10 | 0.10 |
| 73 | 0.10 | 0.10 | 0.10 | 0.10 | 0.10 | 0.10 | 0.10 | 0.10 | 0.10 | 0.10 |
| 74 | 0.10 | 0.01 | 0.02 | 0.10 | 0.10 | 0.10 | 0.10 | 0.10 | 0.10 | 0.10 |
| 75 | 0.01 | 0.10 | 0.10 | 0.03 | 0.10 | 0.10 | 0.10 | 0.10 | 0.10 | 0.10 |
| 76 | 0.01 | 0.04 | 0.10 | 0.10 | 0.10 | 0.10 | 0.04 | 0.10 | 0.10 | 0.10 |
| 77 | 0.10 | 0.10 | 0.08 | 0.04 | 0.10 | 0.10 | 0.10 | 0.10 | 0.10 | 0.10 |
| 78 | 0.08 | 0.07 | 0.10 | 0.02 | 0.10 | 0.10 | 0.10 | 0.10 | 0.10 | 0.10 |
| 79 | 0.10 | 0.03 | 0.05 | 0.10 | 0.10 | 0.10 | 0.10 | 0.10 | 0.10 | 0.10 |
| 80 | 0.10 | 0.10 | 0.04 | 0.10 | 0.10 | 0.10 | 0.10 | 0.10 | 0.10 | 0.10 |
| 81 | 0.01 | 0.01 | 0.01 | 0.02 | 0.03 | 0.10 | 0.10 | 0.10 | 0.10 | 0.10 |
| 82 | 0.10 | 0.05 | 0.02 | 0.03 | 0.06 | 0.07 | 0.10 | 0.10 | 0.08 | 0.10 |
| 83 | 0.10 | 0.02 | 0.01 | 0.10 | 0.10 | 0.10 | 0.10 | 0.10 | 0.10 | 0.10 |
| 84 | 0.03 | 0.10 | 0.10 | 0.10 | 0.10 | 0.10 | 0.10 | 0.10 | 0.10 | 0.10 |
| 85 | 0.10 | 0.10 | 0.01 | 0.10 | 0.06 | 0.10 | 0.10 | 0.10 | 0.10 | 0.10 |
| 86 | 0.10 | 0.09 | 0.09 | 0.10 | 0.10 | 0.10 | 0.10 | 0.04 | 0.10 | 0.10 |
| 87 | 0.10 | 0.02 | 0.02 | 0.10 | 0.10 | 0.10 | 0.10 | 0.10 | 0.10 | 0.10 |
| 88 | 0.01 | 0.10 | 0.10 | 0.10 | 0.10 | 0.10 | 0.10 | 0.10 | 0.10 | 0.10 |
| 89 | 0.10 | 0.10 | 0.10 | 0.10 | 0.10 | 0.10 | 0.10 | 0.10 | 0.10 | 0.10 |
| 90 | 0.01 | 0.10 | 0.09 | 0.04 | 0.01 | 0.10 | 0.10 | 0.10 | 0.10 | 0.10 |
| 91 | 0.03 | 0.10 | 0.10 | 0.01 | 0.01 | 0.10 | 0.10 | 0.10 | 0.10 | 0.10 |
| 92 | 0.10 | 0.01 | 0.10 | 0.05 | 0.07 | 0.10 | 0.10 | 0.10 | 0.10 | 0.10 |
| 93 | 0.10 | 0.01 | 0.04 | 0.10 | 0.10 | 0.10 | 0.10 | 0.10 | 0.10 | 0.10 |
| 94 | 0.07 | 0.10 | 0.10 | 0.10 | 0.10 | 0.10 | 0.10 | 0.10 | 0.10 | 0.10 |
| 95 | 0.01 | 0.06 | 0.10 | 0.10 | 0.10 | 0.09 | 0.10 | 0.10 | 0.10 | 0.10 |
| 96 | 0.01 | 0.01 | 0.03 | 0.09 | 0.02 | 0.10 | 0.10 | 0.10 | 0.10 | 0.10 |
| 97 | 0.01 | 0.10 | 0.03 | 0.10 | 0.10 | 0.10 | 0.10 | 0.09 | 0.10 | 0.10 |
| 98 | 0.01 | 0.01 | 0.08 | 0.10 | 0.04 | 0.10 | 0.10 | 0.10 | 0.10 | 0.10 |
| 99 | 0.10 | 0.10 | 0.10 | 0.10 | 0.10 | 0.10 | 0.10 | 0.10 | 0.10 | 0.10 |
| 100 | 0.03 | 0.10 | 0.10 | 0.10 | 0.10 | 0.10 | 0.10 | 0.10 | 0.10 | 0.10 |
| 101 | 0.10 | 0.01 | 0.03 | 0.10 | 0.10 | 0.10 | 0.10 | 0.10 | 0.10 | 0.10 |
| 102 | 0.03 | 0.05 | 0.04 | 0.10 | 0.01 | 0.10 | 0.10 | 0.10 | 0.10 | 0.10 |
| *ABP, arterial blood pressure; ADF, Augmented Dickey-Fuller; COx, cerebral oximetry index with CPP; COx-a, cerebral oximetry index with ABP; CPP, cerebral perfusion pressure; HC, healthy control volunteer group; KPSS, Kwiatkowski-Phillips-Schmidt-Shin; rSO_2_, regional cerebral oxygen saturation.* | | | | | | | | | | |

File S5b: ADF and KPSS P-Values for Non-Differenced and 1^st^ Order Differenced 1-Minute Data Resolution – SP Patients

| **ADF p-values for 1-minute data resolution** | | | | | | | | | | |
| --- | --- | --- | --- | --- | --- | --- | --- | --- | --- | --- |
| **Patient** | ***Non-Differenced*** | | | | | ***1^st^ Order Differenced*** | | | | |
|  | **ABP** | **rSO2_L** | **rSO2_R** | **COx-a_L** | **COx-a_R** | **ABP** | **rSO2_L** | **rSO2_R** | **COx-a_L** | **COx-a_R** |
| 1 | 0.74 | 0.59 | 0.57 | 0.01 | 0.01 | 0.01 | 0.01 | 0.01 | 0.01 | 0.01 |
| 2 | 0.45 | 0.01 | 0.01 | 0.01 | 0.01 | 0.01 | 0.01 | 0.01 | 0.01 | 0.01 |
| 3 | 0.01 | 0.01 | 0.01 | 0.01 | 0.01 | 0.01 | 0.01 | 0.01 | 0.01 | 0.01 |
| 4 | 0.02 | 0.99 | 0.99 | 0.01 | 0.01 | 0.01 | 0.01 | 0.01 | 0.01 | 0.01 |
| 5 | 0.01 | 0.66 | 0.74 | 0.01 | 0.01 | 0.01 | 0.01 | 0.01 | 0.01 | 0.01 |
| 6 | 0.01 | 0.01 | 0.68 | 0.04 | 0.04 | 0.01 | 0.01 | 0.01 | 0.01 | 0.01 |
| 7 | 0.01 | 0.09 | 0.15 | 0.01 | 0.01 | 0.01 | 0.01 | 0.01 | 0.01 | 0.01 |
| 8 | 0.01 | 0.94 | 0.01 | 0.01 | 0.01 | 0.01 | 0.01 | 0.01 | 0.01 | 0.01 |
| 9 | 0.01 | 0.01 | 0.88 | 0.01 | 0.01 | 0.01 | 0.01 | 0.01 | 0.01 | 0.01 |
| 10 | 0.01 | 0.76 | 0.98 | 0.01 | 0.01 | 0.01 | 0.01 | 0.01 | 0.01 | 0.01 |
| 11 | 0.01 | 0.01 | 0.03 | 0.01 | 0.01 | 0.01 | 0.01 | 0.01 | 0.01 | 0.01 |
| 12 | 0.01 | 0.09 | 0.08 | 0.01 | 0.01 | 0.01 | 0.01 | 0.01 | 0.01 | 0.01 |
| 13 | 0.01 | 0.66 | 0.31 | 0.01 | 0.01 | 0.01 | 0.01 | 0.01 | 0.01 | 0.01 |
| 14 | 0.02 | 0.94 | 0.69 | 0.01 | 0.01 | 0.01 | 0.01 | 0.01 | 0.01 | 0.01 |
| 15 | 0.01 | 0.77 | 0.01 | 0.01 | 0.01 | 0.01 | 0.01 | 0.01 | 0.01 | 0.01 |
| 16 | 0.01 | 0.45 | 0.66 | 0.01 | 0.01 | 0.01 | 0.01 | 0.01 | 0.01 | 0.01 |
| 17 | 0.02 | 0.10 | 0.01 | 0.01 | 0.01 | 0.01 | 0.01 | 0.01 | 0.01 | 0.01 |
| 18 | 0.19 | 0.03 | 0.22 | 0.01 | 0.01 | 0.01 | 0.01 | 0.01 | 0.01 | 0.01 |
| 19 | 0.01 | 0.02 | 0.79 | 0.01 | 0.01 | 0.01 | 0.01 | 0.01 | 0.01 | 0.01 |
| 20 | 0.01 | 0.09 | 0.41 | 0.01 | 0.01 | 0.01 | 0.01 | 0.01 | 0.01 | 0.01 |
| 21 | 0.01 | 0.08 | 0.01 | 0.01 | 0.01 | 0.01 | 0.01 | 0.01 | 0.01 | 0.01 |
| 22 | 0.01 | 0.27 | 0.04 | 0.01 | 0.01 | 0.01 | 0.01 | 0.01 | 0.01 | 0.01 |
| 23 | 0.01 | 0.19 | 0.30 | 0.01 | 0.01 | 0.01 | 0.01 | 0.01 | 0.01 | 0.01 |
| 24 | 0.01 | 0.24 | 0.34 | 0.01 | 0.01 | 0.01 | 0.01 | 0.01 | 0.01 | 0.01 |
| 25 | 0.39 | 0.95 | 0.66 | 0.01 | 0.01 | 0.01 | 0.01 | 0.01 | 0.01 | 0.01 |
| 26 | 0.15 | 0.62 | 0.79 | 0.01 | 0.01 | 0.01 | 0.01 | 0.01 | 0.01 | 0.01 |
| 27 | 0.10 | 0.89 | 0.81 | 0.01 | 0.01 | 0.01 | 0.01 | 0.01 | 0.01 | 0.01 |
| **KPSS p-values for 1-minute data resolution** | | | | | | | | | | |
| **Patient** | ***Non-Differenced*** | | | | | ***1^st^ Order Differenced*** | | | | |
|  | **ABP** | **rSO2_L** | **rSO2_R** | **COx-a_L** | **COx-a_R** | **ABP** | **rSO2_L** | **rSO2_R** | **COx-a_L** | **COx-a_R** |
| 1 | 0.08 | 0.01 | 0.01 | 0.10 | 0.10 | 0.10 | 0.10 | 0.10 | 0.10 | 0.10 |
| 2 | 0.01 | 0.01 | 0.01 | 0.08 | 0.10 | 0.10 | 0.10 | 0.10 | 0.10 | 0.10 |
| 3 | 0.01 | 0.01 | 0.01 | 0.10 | 0.10 | 0.10 | 0.10 | 0.10 | 0.10 | 0.10 |
| 4 | 0.01 | 0.01 | 0.01 | 0.10 | 0.10 | 0.10 | 0.05 | 0.01 | 0.10 | 0.10 |
| 5 | 0.10 | 0.01 | 0.01 | 0.10 | 0.10 | 0.10 | 0.10 | 0.10 | 0.10 | 0.10 |
| 6 | 0.04 | 0.01 | 0.10 | 0.10 | 0.10 | 0.10 | 0.10 | 0.10 | 0.10 | 0.10 |
| 7 | 0.01 | 0.01 | 0.01 | 0.10 | 0.10 | 0.10 | 0.10 | 0.10 | 0.10 | 0.10 |
| 8 | 0.01 | 0.01 | 0.02 | 0.06 | 0.03 | 0.10 | 0.10 | 0.10 | 0.10 | 0.10 |
| 9 | 0.07 | 0.01 | 0.01 | 0.10 | 0.01 | 0.10 | 0.10 | 0.10 | 0.10 | 0.10 |
| 10 | 0.05 | 0.07 | 0.07 | 0.10 | 0.10 | 0.10 | 0.10 | 0.10 | 0.10 | 0.10 |
| 11 | 0.01 | 0.05 | 0.01 | 0.10 | 0.10 | 0.10 | 0.10 | 0.10 | 0.10 | 0.10 |
| 12 | 0.10 | 0.09 | 0.01 | 0.10 | 0.10 | 0.10 | 0.10 | 0.10 | 0.10 | 0.10 |
| 13 | 0.10 | 0.01 | 0.09 | 0.10 | 0.10 | 0.10 | 0.10 | 0.10 | 0.10 | 0.10 |
| 14 | 0.02 | 0.09 | 0.07 | 0.10 | 0.10 | 0.10 | 0.10 | 0.10 | 0.10 | 0.10 |
| 15 | 0.10 | 0.03 | 0.10 | 0.10 | 0.10 | 0.10 | 0.10 | 0.10 | 0.10 | 0.10 |
| 16 | 0.10 | 0.01 | 0.01 | 0.01 | 0.07 | 0.10 | 0.10 | 0.10 | 0.10 | 0.10 |
| 17 | 0.02 | 0.07 | 0.01 | 0.10 | 0.07 | 0.10 | 0.10 | 0.10 | 0.10 | 0.10 |
| 18 | 0.10 | 0.01 | 0.01 | 0.10 | 0.10 | 0.10 | 0.10 | 0.10 | 0.10 | 0.10 |
| 19 | 0.01 | 0.05 | 0.01 | 0.10 | 0.10 | 0.10 | 0.10 | 0.10 | 0.10 | 0.10 |
| 20 | 0.08 | 0.01 | 0.01 | 0.06 | 0.10 | 0.10 | 0.10 | 0.10 | 0.10 | 0.10 |
| 21 | 0.02 | 0.01 | 0.01 | 0.10 | 0.10 | 0.10 | 0.10 | 0.10 | 0.10 | 0.10 |
| 22 | 0.01 | 0.10 | 0.08 | 0.10 | 0.02 | 0.10 | 0.10 | 0.10 | 0.10 | 0.10 |
| 23 | 0.01 | 0.10 | 0.07 | 0.05 | 0.01 | 0.10 | 0.10 | 0.10 | 0.10 | 0.10 |
| 24 | 0.10 | 0.03 | 0.01 | 0.10 | 0.10 | 0.10 | 0.10 | 0.10 | 0.10 | 0.10 |
| 25 | 0.01 | 0.01 | 0.01 | 0.10 | 0.10 | 0.10 | 0.05 | 0.01 | 0.10 | 0.10 |
| 26 | 0.01 | 0.03 | 0.03 | 0.10 | 0.10 | 0.10 | 0.04 | 0.08 | 0.10 | 0.10 |
| 27 | 0.10 | 0.01 | 0.01 | 0.10 | 0.09 | 0.10 | 0.10 | 0.10 | 0.10 | 0.10 |
| *ABP, arterial blood pressure; ADF, Augmented Dickey-Fuller; COx, cerebral oximetry index with CPP; COx-a, cerebral oximetry index with ABP; CPP, cerebral perfusion pressure; KPSS, Kwiatkowski-Phillips-Schmidt-Shin; rSO_2_, regional cerebral oxygen saturation; SP, elective spinal surgery patient group.* | | | | | | | | | | |

File S5c: ADF and KPSS P-Values for Non-Differenced and 1^st^ Order Differenced 1-Minute Data Resolution – TBI-GLR Patients

| **ADF p-values for 1-minute data** | | | | | | | | | | | | | | | | |
| --- | --- | --- | --- | --- | --- | --- | --- | --- | --- | --- | --- | --- | --- | --- | --- | --- |
| **Patient** | ***Non-Differenced*** | | | | | | | | ***1^st^ Order Differenced*** | | | | | | | |
|  | **ABP** | **CPP** | **rSO2_L** | **rSO2_R** | **COx_L** | **COx_R** | **COx-a_L** | **COx-a_R** | **ABP** | **CPP** | **rSO2_L** | **rSO2_R** | **COx_L** | **COx_R** | **COx-a_L** | **COx-a_R** |
| 1 | 0.01 | 0.01 | 0.01 | 0.01 | 0.01 | 0.01 | 0.01 | 0.01 | 0.01 | 0.01 | 0.01 | 0.01 | 0.01 | 0.01 | 0.01 | 0.01 |
| 2 | 0.01 | 0.01 | 0.01 | 0.55 | 0.01 | 0.01 | 0.01 | 0.01 | 0.01 | 0.01 | 0.01 | 0.01 | 0.01 | 0.01 | 0.01 | 0.01 |
| 3 | 0.01 | 0.01 | 0.24 | 0.01 | 0.01 | 0.01 | 0.01 | 0.01 | 0.01 | 0.01 | 0.01 | 0.01 | 0.01 | 0.01 | 0.01 | 0.01 |
| 4 | 0.01 | 0.01 | 0.01 | 0.01 | 0.01 | 0.01 | 0.01 | 0.01 | 0.01 | 0.01 | 0.01 | 0.01 | 0.01 | 0.01 | 0.01 | 0.01 |
| 5 | 0.01 | 0.27 | 0.01 | 0.01 | 0.01 | 0.01 | 0.01 | 0.01 | 0.01 | 0.01 | 0.01 | 0.01 | 0.01 | 0.01 | 0.01 | 0.01 |
| 6 | 0.01 | 0.01 | 0.01 | 0.01 | 0.01 | 0.01 | 0.01 | 0.01 | 0.01 | 0.01 | 0.01 | 0.01 | 0.01 | 0.01 | 0.01 | 0.01 |
| 7 | 0.01 | 0.01 | 0.01 | 0.01 | 0.01 | 0.01 | 0.01 | 0.01 | 0.01 | 0.01 | 0.01 | 0.01 | 0.01 | 0.01 | 0.01 | 0.01 |
| 8 | 0.01 | 0.16 | 0.05 | 0.43 | 0.01 | 0.01 | 0.01 | 0.01 | 0.01 | 0.01 | 0.01 | 0.01 | 0.01 | 0.01 | 0.01 | 0.01 |
| 9 | 0.01 | 0.02 | 0.01 | 0.01 | 0.01 | 0.01 | 0.01 | 0.01 | 0.01 | 0.01 | 0.01 | 0.01 | 0.01 | 0.01 | 0.01 | 0.01 |
| 10 | 0.01 | 0.01 | 0.01 | 0.01 | 0.01 | 0.01 | 0.01 | 0.01 | 0.01 | 0.01 | 0.01 | 0.01 | 0.01 | 0.01 | 0.01 | 0.01 |
| 11 | 0.01 | 0.01 | 0.01 | 0.01 | 0.01 | 0.01 | 0.01 | 0.01 | 0.01 | 0.01 | 0.01 | 0.01 | 0.01 | 0.01 | 0.01 | 0.01 |
| 12 | 0.01 | 0.01 | 0.01 | 0.01 | 0.01 | 0.01 | 0.01 | 0.01 | 0.01 | 0.01 | 0.01 | 0.01 | 0.01 | 0.01 | 0.01 | 0.01 |
| 13 | 0.01 | 0.01 | 0.01 | 0.01 | 0.01 | 0.01 | 0.01 | 0.01 | 0.01 | 0.01 | 0.01 | 0.01 | 0.01 | 0.01 | 0.01 | 0.01 |
| 14 | 0.01 | 0.01 | 0.01 | 0.01 | 0.01 | 0.01 | 0.01 | 0.01 | 0.01 | 0.01 | 0.01 | 0.01 | 0.01 | 0.01 | 0.01 | 0.01 |
| 15 | 0.01 | 0.01 | 0.01 | 0.01 | 0.01 | 0.01 | 0.01 | 0.01 | 0.01 | 0.01 | 0.01 | 0.01 | 0.01 | 0.01 | 0.01 | 0.01 |
| 16 | 0.01 | 0.01 | 0.01 | 0.01 | 0.01 | 0.01 | 0.01 | 0.01 | 0.01 | 0.01 | 0.01 | 0.01 | 0.01 | 0.01 | 0.01 | 0.01 |
| 17 | 0.01 | 0.01 | 0.01 | 0.01 | 0.01 | 0.01 | 0.01 | 0.01 | 0.01 | 0.01 | 0.01 | 0.01 | 0.01 | 0.01 | 0.01 | 0.01 |
| 18 | 0.01 | 0.01 | 0.01 | 0.95 | 0.01 | 0.01 | 0.01 | 0.01 | 0.01 | 0.01 | 0.01 | 0.01 | 0.01 | 0.01 | 0.01 | 0.01 |
| 19 | 0.01 | 0.01 | 0.06 | 0.06 | 0.01 | 0.01 | 0.01 | 0.01 | 0.01 | 0.01 | 0.01 | 0.01 | 0.01 | 0.01 | 0.01 | 0.01 |
| 20 | 0.01 | 0.01 | 0.02 | 0.24 | 0.01 | 0.01 | 0.01 | 0.01 | 0.01 | 0.01 | 0.01 | 0.01 | 0.01 | 0.01 | 0.01 | 0.01 |
| 21 | 0.01 | 0.01 | 0.49 | 0.39 | 0.01 | 0.01 | 0.01 | 0.01 | 0.01 | 0.01 | 0.01 | 0.01 | 0.01 | 0.01 | 0.01 | 0.01 |
| 22 | 0.01 | 0.01 | 0.06 | 0.01 | 0.01 | 0.01 | 0.01 | 0.01 | 0.01 | 0.01 | 0.01 | 0.01 | 0.01 | 0.01 | 0.01 | 0.01 |
| 23 | 0.01 | 0.01 | 0.01 | 0.01 | 0.01 | 0.01 | 0.01 | 0.01 | 0.01 | 0.01 | 0.01 | 0.01 | 0.01 | 0.01 | 0.01 | 0.01 |
| 24 | 0.01 | 0.01 | 0.01 | 0.01 | 0.01 | 0.01 | 0.01 | 0.01 | 0.01 | 0.01 | 0.01 | 0.01 | 0.01 | 0.01 | 0.01 | 0.01 |
| 25 | 0.01 | 0.01 | 0.01 | 0.01 | 0.01 | 0.01 | 0.01 | 0.01 | 0.01 | 0.01 | 0.01 | 0.01 | 0.01 | 0.01 | 0.01 | 0.01 |
| 26 | 0.01 | 0.01 | 0.01 | 0.01 | 0.01 | 0.01 | 0.01 | 0.01 | 0.01 | 0.01 | 0.01 | 0.01 | 0.01 | 0.01 | 0.01 | 0.01 |
| 27 | 0.01 | 0.04 | 0.34 | 0.75 | 0.01 | 0.01 | 0.01 | 0.01 | 0.01 | 0.01 | 0.01 | 0.01 | 0.01 | 0.01 | 0.01 | 0.01 |
| 28 | 0.01 | 0.01 | 0.01 | 0.01 | 0.01 | 0.01 | 0.01 | 0.01 | 0.01 | 0.01 | 0.01 | 0.01 | 0.01 | 0.01 | 0.01 | 0.01 |
| 29 | 0.01 | 0.01 | 0.01 | 0.01 | 0.01 | 0.01 | 0.01 | 0.01 | 0.01 | 0.01 | 0.01 | 0.01 | 0.01 | 0.01 | 0.01 | 0.01 |
| 30 | 0.01 | 0.01 | 0.01 | 0.01 | 0.01 | 0.01 | 0.01 | 0.01 | 0.01 | 0.01 | 0.01 | 0.01 | 0.01 | 0.01 | 0.01 | 0.01 |
| 31 | 0.01 | 0.01 | 0.01 | 0.01 | 0.01 | 0.01 | 0.01 | 0.01 | 0.01 | 0.01 | 0.01 | 0.01 | 0.01 | 0.01 | 0.01 | 0.01 |
| 32 | 0.01 | 0.01 | 0.01 | 0.01 | 0.01 | 0.01 | 0.01 | 0.01 | 0.01 | 0.01 | 0.01 | 0.01 | 0.01 | 0.01 | 0.01 | 0.01 |
| 33 | 0.01 | 0.01 | 0.01 | 0.01 | 0.01 | 0.01 | 0.01 | 0.01 | 0.01 | 0.01 | 0.01 | 0.01 | 0.01 | 0.01 | 0.01 | 0.01 |
| 34 | 0.01 | 0.01 | 0.17 | 0.03 | 0.01 | 0.01 | 0.01 | 0.01 | 0.01 | 0.01 | 0.01 | 0.01 | 0.01 | 0.01 | 0.01 | 0.01 |
| 35 | 0.01 | 0.01 | 0.01 | 0.01 | 0.01 | 0.01 | 0.01 | 0.01 | 0.01 | 0.01 | 0.01 | 0.01 | 0.01 | 0.01 | 0.01 | 0.01 |
| 36 | 0.01 | 0.01 | 0.01 | 0.01 | 0.01 | 0.01 | 0.01 | 0.01 | 0.01 | 0.01 | 0.01 | 0.01 | 0.01 | 0.01 | 0.01 | 0.01 |
| 37 | 0.01 | 0.01 | 0.01 | 0.01 | 0.01 | 0.01 | 0.01 | 0.01 | 0.01 | 0.01 | 0.01 | 0.01 | 0.01 | 0.01 | 0.01 | 0.01 |
| 38 | 0.01 | 0.01 | 0.01 | 0.01 | 0.01 | 0.01 | 0.01 | 0.01 | 0.01 | 0.01 | 0.01 | 0.01 | 0.01 | 0.01 | 0.01 | 0.01 |
| 39 | 0.01 | 0.01 | 0.01 | 0.01 | 0.01 | 0.01 | 0.01 | 0.01 | 0.01 | 0.01 | 0.01 | 0.01 | 0.01 | 0.01 | 0.01 | 0.01 |
| 40 | 0.01 | 0.01 | 0.01 | 0.01 | 0.01 | 0.01 | 0.01 | 0.01 | 0.01 | 0.01 | 0.01 | 0.01 | 0.01 | 0.01 | 0.01 | 0.01 |
| 41 | 0.01 | 0.01 | 0.01 | 0.01 | 0.01 | 0.01 | 0.01 | 0.01 | 0.01 | 0.01 | 0.01 | 0.01 | 0.01 | 0.01 | 0.01 | 0.01 |
| 42 | 0.01 | 0.01 | 0.01 | 0.01 | 0.01 | 0.01 | 0.01 | 0.01 | 0.01 | 0.01 | 0.01 | 0.01 | 0.01 | 0.01 | 0.01 | 0.01 |
| 43 | 0.01 | 0.01 | 0.01 | 0.01 | 0.01 | 0.01 | 0.01 | 0.01 | 0.01 | 0.01 | 0.01 | 0.01 | 0.01 | 0.01 | 0.01 | 0.01 |
| 44 | 0.01 | 0.01 | 0.01 | 0.01 | 0.01 | 0.01 | 0.01 | 0.01 | 0.01 | 0.01 | 0.01 | 0.01 | 0.01 | 0.01 | 0.01 | 0.01 |
| 45 | 0.01 | 0.01 | 0.01 | 0.01 | 0.01 | 0.01 | 0.01 | 0.01 | 0.01 | 0.01 | 0.01 | 0.01 | 0.01 | 0.01 | 0.01 | 0.01 |
| 46 | 0.01 | 0.01 | 0.01 | 0.01 | 0.01 | 0.01 | 0.01 | 0.01 | 0.01 | 0.01 | 0.01 | 0.01 | 0.01 | 0.01 | 0.01 | 0.01 |
| 47 | 0.01 | 0.01 | 0.01 | 0.01 | 0.01 | 0.01 | 0.01 | 0.01 | 0.01 | 0.01 | 0.01 | 0.01 | 0.01 | 0.01 | 0.01 | 0.01 |
| 48 | 0.01 | 0.01 | 0.01 | 0.02 | 0.01 | 0.01 | 0.01 | 0.01 | 0.01 | 0.01 | 0.01 | 0.01 | 0.01 | 0.01 | 0.01 | 0.01 |
| 49 | 0.01 | 0.34 | 0.01 | 0.01 | 0.48 | 0.70 | 0.01 | 0.01 | 0.01 | 0.05 | 0.01 | 0.01 | 0.37 | 0.69 | 0.01 | 0.01 |
| 50 | 0.01 | 0.01 | 0.01 | 0.01 | 0.01 | 0.01 | 0.01 | 0.01 | 0.01 | 0.01 | 0.01 | 0.01 | 0.01 | 0.01 | 0.01 | 0.01 |
| 51 | 0.01 | 0.01 | 0.01 | 0.01 | 0.01 | 0.01 | 0.01 | 0.01 | 0.01 | 0.01 | 0.01 | 0.01 | 0.01 | 0.01 | 0.01 | 0.01 |
| 52 | 0.01 | 0.01 | 0.01 | 0.01 | 0.01 | 0.01 | 0.01 | 0.01 | 0.01 | 0.01 | 0.01 | 0.01 | 0.01 | 0.01 | 0.01 | 0.01 |
| 53 | 0.01 | 0.01 | 0.01 | 0.01 | 0.01 | 0.01 | 0.01 | 0.01 | 0.01 | 0.01 | 0.01 | 0.01 | 0.01 | 0.01 | 0.01 | 0.01 |
| 54 | 0.01 | 0.01 | 0.01 | 0.01 | 0.01 | 0.01 | 0.01 | 0.01 | 0.01 | 0.01 | 0.01 | 0.01 | 0.01 | 0.01 | 0.01 | 0.01 |
| 55 | 0.01 | 0.01 | 0.93 | 0.02 | 0.01 | 0.01 | 0.01 | 0.01 | 0.01 | 0.01 | 0.52 | 0.01 | 0.01 | 0.01 | 0.01 | 0.01 |
| 56 | 0.01 | 0.01 | 0.01 | 0.01 | 0.01 | 0.01 | 0.01 | 0.01 | 0.01 | 0.01 | 0.01 | 0.01 | 0.01 | 0.01 | 0.01 | 0.01 |
| 57 | 0.01 | 0.01 | 0.01 | 0.01 | 0.01 | 0.01 | 0.01 | 0.01 | 0.01 | 0.01 | 0.01 | 0.01 | 0.01 | 0.01 | 0.01 | 0.01 |
| 58 | 0.01 | 0.01 | 0.01 | 0.01 | 0.01 | 0.01 | 0.01 | 0.01 | 0.01 | 0.01 | 0.01 | 0.01 | 0.01 | 0.01 | 0.01 | 0.01 |
| 59 | 0.01 | 0.01 | 0.01 | 0.01 | 0.01 | 0.01 | 0.01 | 0.01 | 0.01 | 0.01 | 0.01 | 0.01 | 0.01 | 0.01 | 0.01 | 0.01 |
| 60 | 0.01 | 0.01 | 0.01 | 0.02 | 0.01 | 0.01 | 0.01 | 0.01 | 0.01 | 0.01 | 0.01 | 0.01 | 0.01 | 0.01 | 0.01 | 0.01 |
| 61 | 0.01 | 0.01 | 0.01 | 0.01 | 0.01 | 0.01 | 0.01 | 0.01 | 0.01 | 0.01 | 0.01 | 0.01 | 0.01 | 0.01 | 0.01 | 0.01 |
| 62 | 0.01 | 0.01 | 0.01 | 0.01 | 0.01 | 0.01 | 0.01 | 0.01 | 0.01 | 0.01 | 0.01 | 0.01 | 0.01 | 0.01 | 0.01 | 0.01 |
| 63 | 0.01 | 0.01 | 0.01 | 0.01 | 0.01 | 0.01 | 0.01 | 0.01 | 0.01 | 0.01 | 0.01 | 0.01 | 0.01 | 0.01 | 0.01 | 0.01 |
| 64 | 0.01 | 0.01 | 0.01 | 0.01 | 0.01 | 0.01 | 0.01 | 0.01 | 0.01 | 0.01 | 0.01 | 0.01 | 0.01 | 0.01 | 0.01 | 0.01 |
| **KPSS p-values for 1-minute data resolution** | | | | | | | | | | | | | | | | |
| **Patient** | ***Non-Differenced*** | | | | | | | | ***1^st^ Order Differenced*** | | | | | | | |
|  | **ABP** | **CPP** | **rSO2_L** | **rSO2_R** | **COx_L** | **COx_R** | **COx-a_L** | **COx-a_R** | **ABP** | **CPP** | **rSO2_L** | **rSO2_R** | **COx_L** | **COx_R** | **COx-a_L** | **COx-a_R** |
| 1 | 0.01 | 0.01 | 0.01 | 0.01 | 0.10 | 0.10 | 0.09 | 0.01 | 0.10 | 0.10 | 0.10 | 0.02 | 0.10 | 0.10 | 0.10 | 0.10 |
| 2 | 0.01 | 0.01 | 0.01 | 0.01 | 0.01 | 0.01 | 0.01 | 0.01 | 0.10 | 0.10 | 0.10 | 0.10 | 0.10 | 0.10 | 0.10 | 0.10 |
| 3 | 0.01 | 0.01 | 0.01 | 0.01 | 0.10 | 0.05 | 0.10 | 0.02 | 0.10 | 0.10 | 0.10 | 0.10 | 0.10 | 0.10 | 0.10 | 0.10 |
| 4 | 0.01 | 0.01 | 0.01 | 0.01 | 0.04 | 0.10 | 0.06 | 0.10 | 0.10 | 0.10 | 0.10 | 0.10 | 0.10 | 0.10 | 0.10 | 0.10 |
| 5 | 0.01 | 0.10 | 0.01 | 0.01 | 0.10 | 0.10 | 0.01 | 0.09 | 0.10 | 0.10 | 0.09 | 0.10 | 0.10 | 0.10 | 0.10 | 0.10 |
| 6 | 0.07 | 0.01 | 0.01 | 0.01 | 0.10 | 0.10 | 0.10 | 0.10 | 0.10 | 0.10 | 0.10 | 0.10 | 0.10 | 0.10 | 0.10 | 0.10 |
| 7 | 0.01 | 0.01 | 0.01 | 0.01 | 0.01 | 0.01 | 0.01 | 0.01 | 0.10 | 0.10 | 0.10 | 0.01 | 0.10 | 0.10 | 0.10 | 0.10 |
| 8 | 0.05 | 0.01 | 0.01 | 0.01 | 0.02 | 0.01 | 0.04 | 0.01 | 0.10 | 0.10 | 0.10 | 0.10 | 0.10 | 0.10 | 0.10 | 0.10 |
| 9 | 0.01 | 0.01 | 0.01 | 0.01 | 0.09 | 0.01 | 0.02 | 0.01 | 0.10 | 0.10 | 0.10 | 0.10 | 0.10 | 0.10 | 0.10 | 0.10 |
| 10 | 0.01 | 0.01 | 0.01 | 0.01 | 0.10 | 0.01 | 0.10 | 0.05 | 0.10 | 0.10 | 0.10 | 0.10 | 0.10 | 0.10 | 0.10 | 0.10 |
| 11 | 0.01 | 0.01 | 0.01 | 0.01 | 0.01 | 0.01 | 0.01 | 0.02 | 0.10 | 0.10 | 0.10 | 0.10 | 0.10 | 0.10 | 0.10 | 0.10 |
| 12 | 0.01 | 0.01 | 0.01 | 0.01 | 0.02 | 0.01 | 0.10 | 0.02 | 0.10 | 0.10 | 0.10 | 0.10 | 0.10 | 0.10 | 0.10 | 0.10 |
| 13 | 0.01 | 0.01 | 0.01 | 0.01 | 0.01 | 0.01 | 0.01 | 0.01 | 0.10 | 0.10 | 0.10 | 0.10 | 0.10 | 0.10 | 0.10 | 0.10 |
| 14 | 0.01 | 0.01 | 0.01 | 0.01 | 0.01 | 0.01 | 0.01 | 0.10 | 0.10 | 0.10 | 0.10 | 0.10 | 0.10 | 0.10 | 0.10 | 0.10 |
| 15 | 0.01 | 0.02 | 0.01 | 0.01 | 0.02 | 0.01 | 0.09 | 0.04 | 0.10 | 0.10 | 0.10 | 0.10 | 0.10 | 0.10 | 0.10 | 0.10 |
| 16 | 0.01 | 0.07 | 0.01 | 0.01 | 0.01 | 0.01 | 0.01 | 0.01 | 0.10 | 0.10 | 0.10 | 0.10 | 0.10 | 0.10 | 0.10 | 0.10 |
| 17 | 0.01 | 0.01 | 0.01 | 0.01 | 0.01 | 0.01 | 0.04 | 0.01 | 0.10 | 0.10 | 0.10 | 0.10 | 0.10 | 0.10 | 0.10 | 0.10 |
| 18 | 0.01 | 0.01 | 0.01 | 0.05 | 0.10 | 0.10 | 0.08 | 0.10 | 0.10 | 0.10 | 0.10 | 0.10 | 0.10 | 0.10 | 0.10 | 0.10 |
| 19 | 0.01 | 0.01 | 0.01 | 0.01 | 0.10 | 0.01 | 0.10 | 0.01 | 0.10 | 0.10 | 0.10 | 0.10 | 0.10 | 0.10 | 0.10 | 0.10 |
| 20 | 0.01 | 0.01 | 0.01 | 0.01 | 0.01 | 0.01 | 0.01 | 0.01 | 0.10 | 0.10 | 0.10 | 0.10 | 0.10 | 0.10 | 0.10 | 0.10 |
| 21 | 0.01 | 0.01 | 0.01 | 0.01 | 0.10 | 0.10 | 0.10 | 0.02 | 0.10 | 0.10 | 0.01 | 0.10 | 0.10 | 0.10 | 0.10 | 0.10 |
| 22 | 0.01 | 0.01 | 0.01 | 0.01 | 0.01 | 0.01 | 0.01 | 0.01 | 0.10 | 0.10 | 0.10 | 0.10 | 0.10 | 0.10 | 0.10 | 0.10 |
| 23 | 0.01 | 0.01 | 0.01 | 0.01 | 0.01 | 0.03 | 0.10 | 0.10 | 0.10 | 0.08 | 0.10 | 0.10 | 0.10 | 0.10 | 0.10 | 0.10 |
| 24 | 0.01 | 0.01 | 0.01 | 0.01 | 0.10 | 0.10 | 0.10 | 0.10 | 0.10 | 0.10 | 0.10 | 0.10 | 0.10 | 0.10 | 0.10 | 0.10 |
| 25 | 0.01 | 0.01 | 0.01 | 0.01 | 0.10 | 0.01 | 0.10 | 0.01 | 0.10 | 0.10 | 0.10 | 0.10 | 0.10 | 0.10 | 0.10 | 0.10 |
| 26 | 0.01 | 0.01 | 0.01 | 0.01 | 0.01 | 0.01 | 0.01 | 0.01 | 0.10 | 0.10 | 0.10 | 0.10 | 0.10 | 0.10 | 0.10 | 0.10 |
| 27 | 0.01 | 0.10 | 0.01 | 0.01 | 0.01 | 0.02 | 0.01 | 0.08 | 0.10 | 0.10 | 0.10 | 0.10 | 0.10 | 0.10 | 0.10 | 0.10 |
| 28 | 0.01 | 0.01 | 0.01 | 0.01 | 0.01 | 0.01 | 0.01 | 0.01 | 0.10 | 0.04 | 0.10 | 0.10 | 0.10 | 0.10 | 0.10 | 0.10 |
| 29 | 0.01 | 0.01 | 0.01 | 0.01 | 0.01 | 0.02 | 0.01 | 0.10 | 0.10 | 0.10 | 0.10 | 0.10 | 0.10 | 0.10 | 0.10 | 0.10 |
| 30 | 0.01 | 0.01 | 0.01 | 0.01 | 0.02 | 0.04 | 0.07 | 0.05 | 0.10 | 0.10 | 0.05 | 0.10 | 0.10 | 0.10 | 0.10 | 0.10 |
| 31 | 0.03 | 0.01 | 0.01 | 0.01 | 0.01 | 0.01 | 0.01 | 0.01 | 0.10 | 0.10 | 0.09 | 0.10 | 0.10 | 0.10 | 0.10 | 0.10 |
| 32 | 0.01 | 0.06 | 0.01 | 0.01 | 0.01 | 0.05 | 0.04 | 0.07 | 0.10 | 0.10 | 0.10 | 0.10 | 0.10 | 0.10 | 0.10 | 0.10 |
| 33 | 0.01 | 0.01 | 0.01 | 0.01 | 0.01 | 0.01 | 0.10 | 0.10 | 0.10 | 0.10 | 0.10 | 0.10 | 0.10 | 0.10 | 0.10 | 0.10 |
| 34 | 0.01 | 0.01 | 0.01 | 0.04 | 0.10 | 0.10 | 0.02 | 0.06 | 0.10 | 0.10 | 0.10 | 0.10 | 0.10 | 0.10 | 0.10 | 0.10 |
| 35 | 0.01 | 0.01 | 0.01 | 0.01 | 0.01 | 0.01 | 0.01 | 0.01 | 0.10 | 0.10 | 0.10 | 0.10 | 0.10 | 0.10 | 0.10 | 0.10 |
| 36 | 0.01 | 0.01 | 0.01 | 0.01 | 0.10 | 0.10 | 0.10 | 0.10 | 0.10 | 0.10 | 0.10 | 0.03 | 0.10 | 0.10 | 0.10 | 0.10 |
| 37 | 0.01 | 0.01 | 0.01 | 0.01 | 0.01 | 0.01 | 0.01 | 0.01 | 0.10 | 0.10 | 0.10 | 0.10 | 0.10 | 0.10 | 0.10 | 0.10 |
| 38 | 0.01 | 0.01 | 0.01 | 0.01 | 0.01 | 0.01 | 0.01 | 0.02 | 0.10 | 0.10 | 0.10 | 0.10 | 0.10 | 0.10 | 0.10 | 0.10 |
| 39 | 0.01 | 0.01 | 0.01 | 0.01 | 0.01 | 0.10 | 0.01 | 0.10 | 0.10 | 0.10 | 0.10 | 0.04 | 0.10 | 0.10 | 0.10 | 0.10 |
| 40 | 0.01 | 0.02 | 0.01 | 0.01 | 0.01 | 0.01 | 0.01 | 0.02 | 0.10 | 0.10 | 0.10 | 0.10 | 0.10 | 0.10 | 0.10 | 0.10 |
| 41 | 0.01 | 0.01 | 0.01 | 0.01 | 0.10 | 0.01 | 0.01 | 0.01 | 0.10 | 0.10 | 0.10 | 0.10 | 0.10 | 0.10 | 0.10 | 0.10 |
| 42 | 0.01 | 0.01 | 0.01 | 0.01 | 0.01 | 0.02 | 0.01 | 0.01 | 0.10 | 0.10 | 0.10 | 0.10 | 0.10 | 0.10 | 0.10 | 0.10 |
| 43 | 0.10 | 0.10 | 0.01 | 0.01 | 0.10 | 0.01 | 0.10 | 0.01 | 0.10 | 0.10 | 0.09 | 0.10 | 0.10 | 0.10 | 0.10 | 0.10 |
| 44 | 0.01 | 0.01 | 0.01 | 0.01 | 0.01 | 0.01 | 0.10 | 0.01 | 0.10 | 0.10 | 0.10 | 0.10 | 0.10 | 0.10 | 0.10 | 0.10 |
| 45 | 0.01 | 0.01 | 0.01 | 0.01 | 0.01 | 0.01 | 0.01 | 0.01 | 0.10 | 0.10 | 0.10 | 0.10 | 0.10 | 0.10 | 0.10 | 0.10 |
| 46 | 0.01 | 0.01 | 0.01 | 0.01 | 0.01 | 0.01 | 0.01 | 0.01 | 0.10 | 0.10 | 0.10 | 0.10 | 0.10 | 0.10 | 0.10 | 0.10 |
| 47 | 0.01 | 0.01 | 0.01 | 0.01 | 0.01 | 0.01 | 0.01 | 0.01 | 0.10 | 0.10 | 0.10 | 0.10 | 0.10 | 0.10 | 0.10 | 0.10 |
| 48 | 0.01 | 0.01 | 0.01 | 0.01 | 0.01 | 0.01 | 0.01 | 0.01 | 0.10 | 0.10 | 0.10 | 0.10 | 0.10 | 0.10 | 0.10 | 0.10 |
| 49 | 0.01 | 0.10 | 0.01 | 0.01 | 0.08 | 0.10 | 0.10 | 0.05 | 0.10 | 0.10 | 0.10 | 0.10 | 0.10 | 0.10 | 0.10 | 0.10 |
| 50 | 0.01 | 0.01 | 0.01 | 0.01 | 0.01 | 0.01 | 0.01 | 0.01 | 0.10 | 0.10 | 0.10 | 0.10 | 0.10 | 0.10 | 0.10 | 0.10 |
| 51 | 0.04 | 0.01 | 0.01 | 0.01 | 0.01 | 0.01 | 0.01 | 0.01 | 0.10 | 0.10 | 0.10 | 0.10 | 0.10 | 0.10 | 0.10 | 0.10 |
| 52 | 0.01 | 0.01 | 0.01 | 0.01 | 0.01 | 0.01 | 0.03 | 0.10 | 0.10 | 0.10 | 0.10 | 0.10 | 0.10 | 0.10 | 0.10 | 0.10 |
| 53 | 0.01 | 0.01 | 0.01 | 0.01 | 0.01 | 0.01 | 0.01 | 0.01 | 0.10 | 0.10 | 0.10 | 0.10 | 0.10 | 0.10 | 0.10 | 0.10 |
| 54 | 0.01 | 0.01 | 0.01 | 0.01 | 0.10 | 0.04 | 0.05 | 0.01 | 0.10 | 0.10 | 0.10 | 0.10 | 0.10 | 0.10 | 0.10 | 0.10 |
| 55 | 0.01 | 0.01 | 0.01 | 0.01 | 0.10 | 0.10 | 0.10 | 0.10 | 0.10 | 0.10 | 0.10 | 0.10 | 0.10 | 0.10 | 0.10 | 0.10 |
| 56 | 0.01 | 0.01 | 0.01 | 0.01 | 0.01 | 0.01 | 0.01 | 0.01 | 0.10 | 0.10 | 0.10 | 0.10 | 0.10 | 0.10 | 0.10 | 0.10 |
| 57 | 0.01 | 0.01 | 0.01 | 0.01 | 0.10 | 0.10 | 0.01 | 0.01 | 0.10 | 0.10 | 0.10 | 0.10 | 0.10 | 0.10 | 0.10 | 0.10 |
| 58 | 0.03 | 0.01 | 0.10 | 0.01 | 0.01 | 0.03 | 0.01 | 0.01 | 0.10 | 0.10 | 0.10 | 0.10 | 0.10 | 0.10 | 0.10 | 0.10 |
| 59 | 0.01 | 0.01 | 0.01 | 0.01 | 0.01 | 0.01 | 0.01 | 0.01 | 0.10 | 0.10 | 0.10 | 0.10 | 0.10 | 0.10 | 0.10 | 0.10 |
| 60 | 0.01 | 0.01 | 0.01 | 0.01 | 0.01 | 0.01 | 0.01 | 0.10 | 0.10 | 0.10 | 0.10 | 0.10 | 0.10 | 0.10 | 0.10 | 0.10 |
| 61 | 0.01 | 0.01 | 0.01 | 0.01 | 0.01 | 0.01 | 0.03 | 0.01 | 0.10 | 0.10 | 0.10 | 0.10 | 0.10 | 0.10 | 0.10 | 0.10 |
| 62 | 0.01 | 0.01 | 0.01 | 0.01 | 0.01 | 0.01 | 0.01 | 0.01 | 0.10 | 0.10 | 0.10 | 0.10 | 0.10 | 0.10 | 0.10 | 0.10 |
| 63 | 0.05 | 0.02 | 0.01 | 0.01 | 0.01 | 0.01 | 0.07 | 0.01 | 0.10 | 0.10 | 0.10 | 0.10 | 0.10 | 0.10 | 0.10 | 0.10 |
| 64 | 0.01 | 0.01 | 0.01 | 0.01 | 0.01 | 0.01 | 0.01 | 0.01 | 0.10 | 0.10 | 0.10 | 0.10 | 0.10 | 0.10 | 0.10 | 0.10 |
| *ABP, arterial blood pressure; ADF, Augmented Dickey-Fuller; COx, cerebral oximetry index with CPP; COx-a, cerebral oximetry index with ABP; CPP, cerebral perfusion pressure; KPSS, Kwiatkowski-Phillips-Schmidt-Shin; rSO_2_, regional cerebral oxygen saturation; TBI-GLR, traumatic brain injury patient group without bifrontal lobe pathology.* | | | | | | | | | | | | | | | | |

File S5d: ADF and KPSS P-Values for Non-Differenced and 1^st^ Order Differenced 1-Minute Data Resolution – TBI-GL Patients

| **ADF p-values for 1-minute data** | | | | | | | | | | | | | | | | |
| --- | --- | --- | --- | --- | --- | --- | --- | --- | --- | --- | --- | --- | --- | --- | --- | --- |
| **Patient** | ***Non-Differenced*** | | | | | | | | ***1^st^ Order Differenced*** | | | | | | | |
|  | **ABP** | **CPP** | **rSO2_L** | **rSO2_R** | **COx_L** | **COx_R** | **COx-a_L** | **COx-a_R** | **ABP** | **CPP** | **rSO2_L** | **rSO2_R** | **COx_L** | **COx_R** | **COx-a_L** | **COx-a_R** |
| 1 | 0.01 | 0.01 | 0.01 | 0.62 | 0.01 | 0.01 | 0.01 | 0.01 | 0.01 | 0.01 | 0.01 | 0.01 | 0.01 | 0.01 | 0.01 | 0.01 |
| 2 | 0.01 | 0.01 | 0.01 | 0.02 | 0.01 | 0.01 | 0.01 | 0.01 | 0.01 | 0.01 | 0.01 | 0.01 | 0.01 | 0.01 | 0.01 | 0.01 |
| 3 | 0.01 | 0.01 | 0.03 | 0.01 | 0.01 | 0.01 | 0.01 | 0.01 | 0.01 | 0.01 | 0.01 | 0.01 | 0.01 | 0.01 | 0.01 | 0.01 |
| 4 | 0.01 | 0.01 | 0.08 | 0.01 | 0.01 | 0.01 | 0.01 | 0.01 | 0.01 | 0.01 | 0.01 | 0.01 | 0.01 | 0.01 | 0.01 | 0.01 |
| 5 | 0.01 | 0.01 | 0.01 | 0.01 | 0.01 | 0.01 | 0.01 | 0.01 | 0.01 | 0.01 | 0.01 | 0.01 | 0.01 | 0.01 | 0.01 | 0.01 |
| 6 | 0.01 | 0.01 | 0.01 | 0.01 | 0.01 | 0.01 | 0.01 | 0.01 | 0.01 | 0.01 | 0.01 | 0.01 | 0.01 | 0.01 | 0.01 | 0.01 |
| 7 | 0.01 | 0.01 | 0.01 | 0.01 | 0.01 | 0.01 | 0.01 | 0.01 | 0.01 | 0.01 | 0.01 | 0.01 | 0.01 | 0.01 | 0.01 | 0.01 |
| 8 | 0.01 | 0.01 | 0.01 | 0.01 | 0.01 | 0.01 | 0.01 | 0.01 | 0.01 | 0.01 | 0.01 | 0.01 | 0.01 | 0.01 | 0.01 | 0.01 |
| 9 | 0.01 | 0.01 | 0.01 | 0.01 | 0.01 | 0.01 | 0.01 | 0.01 | 0.01 | 0.01 | 0.01 | 0.01 | 0.01 | 0.01 | 0.01 | 0.01 |
| 10 | 0.01 | 0.01 | 0.03 | 0.01 | 0.01 | 0.01 | 0.01 | 0.01 | 0.01 | 0.01 | 0.01 | 0.01 | 0.01 | 0.01 | 0.01 | 0.01 |
| 11 | 0.01 | 0.01 | 0.01 | 0.01 | 0.01 | 0.01 | 0.01 | 0.01 | 0.01 | 0.01 | 0.01 | 0.01 | 0.01 | 0.01 | 0.01 | 0.01 |
| 12 | 0.01 | 0.01 | 0.01 | 0.01 | 0.01 | 0.01 | 0.01 | 0.01 | 0.01 | 0.01 | 0.01 | 0.01 | 0.01 | 0.01 | 0.01 | 0.01 |
| 13 | 0.01 | 0.01 | 0.01 | 0.01 | 0.01 | 0.01 | 0.01 | 0.01 | 0.01 | 0.01 | 0.01 | 0.01 | 0.01 | 0.01 | 0.01 | 0.01 |
| 14 | 0.01 | 0.01 | 0.01 | 0.01 | 0.01 | 0.01 | 0.01 | 0.01 | 0.01 | 0.01 | 0.01 | 0.01 | 0.01 | 0.01 | 0.01 | 0.01 |
| 15 | 0.01 | 0.01 | 0.01 | 0.01 | 0.01 | 0.01 | 0.01 | 0.01 | 0.01 | 0.01 | 0.01 | 0.01 | 0.01 | 0.01 | 0.01 | 0.01 |
| **KPSS p-values for 1-minute data resolution** | | | | | | | | | | | | | | | | |
| **Patient** | ***Non-Differenced*** | | | | | | | | ***1^st^ Order Differenced*** | | | | | | | |
|  | **ABP** | **CPP** | **rSO2_L** | **rSO2_R** | **COx_L** | **COx_R** | **COx-a_L** | **COx-a_R** | **ABP** | **CPP** | **rSO2_L** | **rSO2_R** | **COx_L** | **COx_R** | **COx-a_L** | **COx-a_R** |
| 1 | 0.01 | 0.01 | 0.01 | 0.01 | 0.01 | 0.01 | 0.01 | 0.01 | 0.10 | 0.10 | 0.10 | 0.10 | 0.10 | 0.10 | 0.10 | 0.10 |
| 2 | 0.01 | 0.01 | 0.01 | 0.01 | 0.01 | 0.01 | 0.01 | 0.01 | 0.10 | 0.10 | 0.10 | 0.10 | 0.10 | 0.10 | 0.10 | 0.10 |
| 3 | 0.01 | 0.01 | 0.01 | 0.01 | 0.10 | 0.10 | 0.05 | 0.10 | 0.10 | 0.10 | 0.10 | 0.10 | 0.10 | 0.10 | 0.10 | 0.10 |
| 4 | 0.01 | 0.04 | 0.01 | 0.01 | 0.04 | 0.10 | 0.10 | 0.04 | 0.10 | 0.10 | 0.10 | 0.10 | 0.10 | 0.10 | 0.10 | 0.10 |
| 5 | 0.01 | 0.01 | 0.01 | 0.01 | 0.10 | 0.01 | 0.10 | 0.01 | 0.10 | 0.10 | 0.10 | 0.10 | 0.10 | 0.10 | 0.10 | 0.10 |
| 6 | 0.10 | 0.02 | 0.01 | 0.01 | 0.10 | 0.10 | 0.10 | 0.04 | 0.10 | 0.10 | 0.10 | 0.10 | 0.10 | 0.10 | 0.10 | 0.10 |
| 7 | 0.01 | 0.01 | 0.01 | 0.02 | 0.05 | 0.10 | 0.02 | 0.10 | 0.10 | 0.10 | 0.10 | 0.10 | 0.10 | 0.10 | 0.10 | 0.10 |
| 8 | 0.01 | 0.01 | 0.01 | 0.01 | 0.06 | 0.01 | 0.10 | 0.02 | 0.10 | 0.10 | 0.10 | 0.08 | 0.10 | 0.10 | 0.10 | 0.10 |
| 9 | 0.01 | 0.01 | 0.01 | 0.01 | 0.01 | 0.01 | 0.01 | 0.01 | 0.10 | 0.10 | 0.10 | 0.10 | 0.10 | 0.10 | 0.10 | 0.10 |
| 10 | 0.01 | 0.01 | 0.01 | 0.01 | 0.01 | 0.10 | 0.01 | 0.10 | 0.10 | 0.10 | 0.10 | 0.10 | 0.10 | 0.10 | 0.10 | 0.10 |
| 11 | 0.01 | 0.01 | 0.01 | 0.01 | 0.01 | 0.01 | 0.01 | 0.01 | 0.10 | 0.10 | 0.10 | 0.10 | 0.10 | 0.10 | 0.10 | 0.10 |
| 12 | 0.01 | 0.01 | 0.01 | 0.01 | 0.01 | 0.04 | 0.01 | 0.01 | 0.10 | 0.10 | 0.10 | 0.10 | 0.10 | 0.10 | 0.10 | 0.10 |
| 13 | 0.01 | 0.01 | 0.01 | 0.01 | 0.01 | 0.01 | 0.01 | 0.01 | 0.10 | 0.10 | 0.10 | 0.10 | 0.10 | 0.10 | 0.10 | 0.10 |
| 14 | 0.01 | 0.01 | 0.01 | 0.01 | 0.01 | 0.03 | 0.01 | 0.05 | 0.10 | 0.10 | 0.10 | 0.10 | 0.10 | 0.10 | 0.10 | 0.10 |
| 15 | 0.01 | 0.01 | 0.01 | 0.01 | 0.01 | 0.01 | 0.01 | 0.01 | 0.10 | 0.10 | 0.10 | 0.10 | 0.10 | 0.10 | 0.10 | 0.10 |
| *ABP, arterial blood pressure; ADF, Augmented Dickey-Fuller; COx, cerebral oximetry index with CPP; COx-a, cerebral oximetry index with ABP; CPP, cerebral perfusion pressure; KPSS, Kwiatkowski-Phillips-Schmidt-Shin; rSO_2_, regional cerebral oxygen saturation; TBI-GL, traumatic brain injury patient group without left frontal lobe pathology.* | | | | | | | | | | | | | | | | |

File S5e: ADF and KPSS P-Values for Non-Differenced and 1^st^ Order Differenced 1-Minute Data Resolution – TBI-GR Patients

| **ADF p-values for 1-minute data** | | | | | | | | | | | | | | | | |
| --- | --- | --- | --- | --- | --- | --- | --- | --- | --- | --- | --- | --- | --- | --- | --- | --- |
| **Patient** | ***Non-Differenced*** | | | | | | | | ***1^st^ Order Differenced*** | | | | | | | |
|  | **ABP** | **CPP** | **rSO2_L** | **rSO2_R** | **COx_L** | **COx_R** | **COx-a_L** | **COx-a_R** | **ABP** | **CPP** | **rSO2_L** | **rSO2_R** | **COx_L** | **COx_R** | **COx-a_L** | **COx-a_R** |
| 1 | 0.01 | 0.01 | 0.43 | 0.01 | 0.01 | 0.01 | 0.01 | 0.01 | 0.01 | 0.01 | 0.01 | 0.01 | 0.01 | 0.01 | 0.01 | 0.01 |
| 2 | 0.01 | 0.01 | 0.01 | 0.01 | 0.01 | 0.01 | 0.01 | 0.01 | 0.01 | 0.01 | 0.01 | 0.01 | 0.01 | 0.01 | 0.01 | 0.01 |
| 3 | 0.01 | 0.01 | 0.04 | 0.01 | 0.01 | 0.01 | 0.01 | 0.01 | 0.01 | 0.01 | 0.01 | 0.01 | 0.01 | 0.01 | 0.01 | 0.01 |
| 4 | 0.01 | 0.01 | 0.01 | 0.01 | 0.01 | 0.01 | 0.01 | 0.01 | 0.01 | 0.01 | 0.01 | 0.01 | 0.01 | 0.01 | 0.01 | 0.01 |
| 5 | 0.01 | 0.01 | 0.01 | 0.01 | 0.01 | 0.01 | 0.01 | 0.01 | 0.01 | 0.01 | 0.01 | 0.01 | 0.01 | 0.01 | 0.01 | 0.01 |
| 6 | 0.01 | 0.01 | 0.01 | 0.01 | 0.01 | 0.01 | 0.01 | 0.01 | 0.01 | 0.01 | 0.01 | 0.01 | 0.01 | 0.01 | 0.01 | 0.01 |
| 7 | 0.01 | 0.01 | 0.06 | 0.01 | 0.01 | 0.01 | 0.01 | 0.01 | 0.01 | 0.01 | 0.01 | 0.01 | 0.01 | 0.01 | 0.01 | 0.01 |
| 8 | 0.01 | 0.01 | 0.99 | 0.99 | 0.01 | 0.01 | 0.01 | 0.01 | 0.01 | 0.01 | 0.01 | 0.01 | 0.01 | 0.01 | 0.01 | 0.01 |
| 9 | 0.01 | 0.01 | 0.01 | 0.01 | 0.01 | 0.01 | 0.01 | 0.01 | 0.01 | 0.01 | 0.01 | 0.01 | 0.01 | 0.01 | 0.01 | 0.01 |
| 10 | 0.01 | 0.01 | 0.01 | 0.01 | 0.01 | 0.01 | 0.01 | 0.01 | 0.01 | 0.01 | 0.01 | 0.01 | 0.01 | 0.01 | 0.01 | 0.01 |
| 11 | 0.01 | 0.01 | 0.01 | 0.01 | 0.01 | 0.01 | 0.01 | 0.01 | 0.01 | 0.01 | 0.01 | 0.01 | 0.01 | 0.01 | 0.01 | 0.01 |
| **KPSS p-values for 1-minute data resolution** | | | | | | | | | | | | | | | | |
| **Patient** | ***Non-Differenced*** | | | | | | | | ***1^st^ Order Differenced*** | | | | | | | |
|  | **ABP** | **CPP** | **rSO2_L** | **rSO2_R** | **COx_L** | **COx_R** | **COx-a_L** | **COx-a_R** | **ABP** | **CPP** | **rSO2_L** | **rSO2_R** | **COx_L** | **COx_R** | **COx-a_L** | **COx-a_R** |
| 1 | 0.01 | 0.01 | 0.01 | 0.01 | 0.07 | 0.03 | 0.10 | 0.03 | 0.10 | 0.10 | 0.10 | 0.10 | 0.10 | 0.10 | 0.10 | 0.10 |
| 2 | 0.01 | 0.01 | 0.01 | 0.01 | 0.01 | 0.01 | 0.04 | 0.01 | 0.10 | 0.10 | 0.07 | 0.10 | 0.10 | 0.10 | 0.10 | 0.10 |
| 3 | 0.01 | 0.01 | 0.01 | 0.01 | 0.04 | 0.01 | 0.02 | 0.01 | 0.10 | 0.10 | 0.10 | 0.10 | 0.10 | 0.10 | 0.10 | 0.10 |
| 4 | 0.01 | 0.01 | 0.01 | 0.01 | 0.02 | 0.10 | 0.02 | 0.09 | 0.10 | 0.10 | 0.10 | 0.10 | 0.10 | 0.10 | 0.10 | 0.10 |
| 5 | 0.01 | 0.02 | 0.01 | 0.01 | 0.10 | 0.01 | 0.10 | 0.01 | 0.10 | 0.10 | 0.10 | 0.10 | 0.10 | 0.10 | 0.10 | 0.10 |
| 6 | 0.01 | 0.01 | 0.01 | 0.01 | 0.10 | 0.01 | 0.07 | 0.02 | 0.10 | 0.10 | 0.10 | 0.10 | 0.10 | 0.10 | 0.10 | 0.10 |
| 7 | 0.01 | 0.01 | 0.01 | 0.01 | 0.05 | 0.10 | 0.01 | 0.10 | 0.10 | 0.10 | 0.10 | 0.10 | 0.10 | 0.10 | 0.10 | 0.10 |
| 8 | 0.01 | 0.01 | 0.01 | 0.01 | 0.05 | 0.01 | 0.10 | 0.01 | 0.10 | 0.10 | 0.10 | 0.10 | 0.10 | 0.10 | 0.10 | 0.10 |
| 9 | 0.01 | 0.01 | 0.01 | 0.01 | 0.10 | 0.01 | 0.10 | 0.01 | 0.10 | 0.10 | 0.10 | 0.10 | 0.10 | 0.10 | 0.10 | 0.10 |
| 10 | 0.01 | 0.01 | 0.01 | 0.01 | 0.01 | 0.04 | 0.01 | 0.10 | 0.10 | 0.10 | 0.10 | 0.10 | 0.10 | 0.10 | 0.10 | 0.10 |
| 11 | 0.01 | 0.01 | 0.01 | 0.01 | 0.01 | 0.01 | 0.01 | 0.01 | 0.10 | 0.10 | 0.10 | 0.10 | 0.10 | 0.10 | 0.10 | 0.10 |
| *ABP, arterial blood pressure; ADF, Augmented Dickey-Fuller; COx, cerebral oximetry index with CPP; COx-a, cerebral oximetry index with ABP; CPP, cerebral perfusion pressure; KPSS, Kwiatkowski-Phillips-Schmidt-Shin; rSO_2_, regional cerebral oxygen saturation; TBI-GR, traumatic brain injury patient group without right frontal lobe pathology.* | | | | | | | | | | | | | | | | |

File S5f: ADF and KPSS P-Values for Non-Differenced and 1^st^ Order Differenced 1-Minute Data Resolution – TBI-BLR Patients

| **ADF p-values for 1-minute data** | | | | | | | | | | | | | | | | |
| --- | --- | --- | --- | --- | --- | --- | --- | --- | --- | --- | --- | --- | --- | --- | --- | --- |
| **Patient** | ***Non-Differenced*** | | | | | | | | ***1^st^ Order Differenced*** | | | | | | | |
|  | **ABP** | **CPP** | **rSO2_L** | **rSO2_R** | **COx_L** | **COx_R** | **COx-a_L** | **COx-a_R** | **ABP** | **CPP** | **rSO2_L** | **rSO2_R** | **COx_L** | **COx_R** | **COx-a_L** | **COx-a_R** |
| 1 | 0.01 | 0.01 | 0.55 | 0.01 | 0.01 | 0.01 | 0.01 | 0.01 | 0.01 | 0.01 | 0.01 | 0.01 | 0.01 | 0.01 | 0.01 | 0.01 |
| 2 | 0.01 | 0.01 | 0.01 | 0.01 | 0.01 | 0.01 | 0.01 | 0.01 | 0.01 | 0.01 | 0.01 | 0.01 | 0.01 | 0.01 | 0.01 | 0.01 |
| 3 | 0.01 | 0.01 | 0.01 | 0.01 | 0.01 | 0.01 | 0.01 | 0.01 | 0.01 | 0.01 | 0.01 | 0.01 | 0.01 | 0.01 | 0.01 | 0.01 |
| 4 | 0.01 | 0.01 | 0.01 | 0.01 | 0.01 | 0.01 | 0.01 | 0.01 | 0.01 | 0.01 | 0.01 | 0.01 | 0.01 | 0.01 | 0.01 | 0.01 |
| 5 | 0.01 | 0.01 | 0.01 | 0.01 | 0.01 | 0.01 | 0.01 | 0.01 | 0.01 | 0.01 | 0.01 | 0.01 | 0.01 | 0.01 | 0.01 | 0.01 |
| **KPSS p-values for 1-minute data resolution** | | | | | | | | | | | | | | | | |
| **Patient** | ***Non-Differenced*** | | | | | | | | ***1^st^ Order Differenced*** | | | | | | | |
|  | **ABP** | **CPP** | **rSO2_L** | **rSO2_R** | **COx_L** | **COx_R** | **COx-a_L** | **COx-a_R** | **ABP** | **CPP** | **rSO2_L** | **rSO2_R** | **COx_L** | **COx_R** | **COx-a_L** | **COx-a_R** |
| 1 | 0.01 | 0.01 | 0.01 | 0.01 | 0.01 | 0.01 | 0.01 | 0.01 | 0.06 | 0.10 | 0.10 | 0.10 | 0.10 | 0.10 | 0.10 | 0.10 |
| 2 | 0.01 | 0.01 | 0.01 | 0.01 | 0.01 | 0.01 | 0.01 | 0.01 | 0.10 | 0.10 | 0.10 | 0.10 | 0.10 | 0.10 | 0.10 | 0.10 |
| 3 | 0.01 | 0.01 | 0.01 | 0.01 | 0.01 | 0.01 | 0.01 | 0.01 | 0.10 | 0.10 | 0.10 | 0.10 | 0.10 | 0.10 | 0.10 | 0.10 |
| 4 | 0.01 | 0.01 | 0.01 | 0.01 | 0.01 | 0.01 | 0.01 | 0.01 | 0.10 | 0.10 | 0.10 | 0.10 | 0.10 | 0.10 | 0.10 | 0.10 |
| 5 | 0.01 | 0.01 | 0.03 | 0.01 | 0.01 | 0.01 | 0.01 | 0.01 | 0.10 | 0.10 | 0.10 | 0.10 | 0.10 | 0.10 | 0.10 | 0.10 |
| *ABP, arterial blood pressure; ADF, Augmented Dickey-Fuller; COx, cerebral oximetry index with CPP; COx-a, cerebral oximetry index with ABP; CPP, cerebral perfusion pressure; KPSS, Kwiatkowski-Phillips-Schmidt-Shin; rSO_2_, regional cerebral oxygen saturation; TBI-BLR, traumatic brain injury patient group with bifrontal lobe pathology.* | | | | | | | | | | | | | | | | |

File S5g: Recorded AIC while fitting various ARIMA models – TBI-GLR Patient Example

| **ARIMA Model** | **ABP** | **CPP** | **rSO_2__L** | **rSO_2__R** | **COx_L** | **COx_R** | **COx_a_L** | **COx_a_R** |
| --- | --- | --- | --- | --- | --- | --- | --- | --- |
| (1,1,0) | 115067.9596 | 105925.3525 | 58390.6927 | 60227.9860 | -7515.3808 | -8395.8510 | -7458.6901 | -8465.5108 |
| (1,1,1) | 115018.6252 | 105905.0248 | 58315.9306 | 59982.1518 | -8065.9646 | -8911.0404 | -8039.0077 | -9012.6746 |
| (1,1,2) | 115020.4209 | 105876.5969 | 57858.2239 | 59255.4823 | -9563.3769 | -10484.6654 | -9543.9911 | -10626.5842 |
| (1,1,3) | 115018.1206 | 105870.5720 | 57768.9904 | 59231.7558 | -9706.1675 | -10656.8600 | -9680.7753 | -10775.0157 |
| (1,1,4) | 115020.1022 | 105872.7089 | 57761.8845 | 59226.5235 | -9706.4333 | -10660.6416 | -9680.1432 | -10775.0832 |
| (1,1,5) | 115019.9472 | 105852.0760 | 57719.4963 | 59228.1273 | -9704.4992 | -10659.3409 | -9680.9615 | -10773.0856 |
| (1,1,6) | 115021.2974 | 105864.7126 | 57718.8411 | 59228.7882 | -9708.2588 | -10669.3403 | -9684.4948 | -10782.5349 |
| (1,1,7) | 115022.0454 | 105866.3947 | 57711.6403 | 59228.0471 | -9706.3371 | -10667.5642 | -9682.6943 | -10780.7826 |
| (1,1,8) | 115023.5883 | 105868.1726 | 57713.3162 | 59230.0174 | -9706.8986 | -10669.6354 | -9688.2047 | -10785.8197 |
| (1,1,9) | 115025.1821 | 105869.3904 | 57702.7711 | 59231.9845 | -9707.8232 | -10668.5786 | -9689.3407 | -10784.5799 |
| (1,1,10) | 115026.9003 | 105870.6384 | 57696.4330 | 59223.5208 | -9708.3582 | -10666.8108 | -9689.8010 | -10783.8896 |
| (2,1,0) | 115014.4321 | 105890.8837 | 58128.8509 | 59627.2706 | -8451.8984 | -9309.8748 | -8445.0788 | -9425.3952 |
| (2,1,1) | 115016.4333 | 105868.9799 | 57815.7120 | 59342.7576 | -9498.1964 | -10490.8230 | -9439.6162 | -10570.4492 |
| (2,1,2) | 115018.5168 | 105870.1516 | 57810.4284 | 59239.0954 | -9701.8387 | -10656.1866 | -9672.8743 | -10769.3893 |
| (2,1,3) | 115020.1523 | 105850.5561 | 57719.4610 | 59223.4271 | -9706.1842 | -10660.4463 | -9679.7780 | -10774.9041 |
| (2,1,4) | 115022.1355 | 105853.7524 | 57706.0906 | 59226.4686 | -9703.5638 | -10655.5340 | -9678.4225 | -10771.7781 |
| (2,1,5) | 115021.9670 | 105849.5585 | 57707.7482 | 59224.2235 | -9702.8618 | -10662.4236 | -9676.2556 | -10773.1916 |
| (2,1,6) | 115023.3216 | 105852.4465 | 57704.1062 | 59225.3881 | -9704.7636 | -10664.6646 | -9683.2304 | -10781.4810 |
| (2,1,7) | 115024.0581 | 105868.4623 | 57708.7840 | 59186.9685 | -9704.2578 | -10665.4104 | -9681.4132 | -10779.5702 |
| (2,1,8) | 115025.6003 | 105870.2151 | 57714.0306 | 59186.8407 | -9702.6389 | -10665.2867 | -9679.8836 | -10777.6539 |
| (2,1,9) | 115027.1926 | 105871.4458 | 57629.2539 | 59187.7255 | -9703.8376 | -10665.6948 | -9686.2728 | -10783.5885 |
| (2,1,10) | 115028.9088 | 105872.7121 | 57629.9584 | 59187.7663 | -9707.5465 | -10665.3682 | -9688.1193 | -10782.8365 |
| (3,1,0) | 115016.4321 | 105874.0028 | 57939.7072 | 59562.7828 | -8570.2843 | -9470.1373 | -8559.3163 | -9560.9764 |
| (3,1,1) | 115018.4321 | 105870.3124 | 57805.2420 | 59231.8811 | -9708.7322 | -10665.8377 | -9685.7138 | -10779.2267 |
| (3,1,2) | 115020.4757 | 105851.1032 | 57750.9464 | 59231.5010 | -9708.3444 | -10663.8423 | -9684.7525 | -10777.8929 |
| (3,1,3) | 115021.9751 | 105851.8677 | 57709.8294 | 59224.5370 | -9702.9821 | -10660.1357 | -9674.4372 | -10773.3444 |
| (3,1,4) | 115013.6753 | 105853.2152 | 57708.3093 | 59226.8319 | -9702.1969 | -10657.0355 | -9681.1382 | -10771.2052 |
| (3,1,5) | 115015.2565 | 105851.5262 | 57706.5351 | 59225.5020 | -9702.4270 | -10662.4115 | -9679.0964 | -10772.7862 |
| (3,1,6) | 115025.3039 | 105853.4980 | 57705.5819 | 59232.0317 | -9703.0266 | -10669.9849 | -9681.1154 | -10779.3782 |
| (3,1,7) | 115026.0334 | 105855.9686 | 57712.2717 | 59187.3836 | -9701.4561 | -10667.2043 | -9679.5605 | -10777.5996 |
| (3,1,8) | 115027.5795 | 105856.6688 | 57696.1416 | 59190.8699 | -9700.3204 | -10662.3246 | -9678.9680 | -10777.8920 |
| (3,1,9) | 115029.1752 | 105858.6145 | 57629.9445 | 59187.4482 | -9698.9373 | -10660.9154 | -9681.9135 | -10778.6430 |
| (3,1,10) | 115030.8879 | 105859.7924 | 57617.0090 | 59182.9490 | -9700.6553 | -10663.8633 | -9684.8086 | -10781.1935 |
| (4,1,0) | 115018.3830 | 105872.7605 | 57929.9697 | 59501.4180 | -8716.8080 | -9639.7931 | -8692.4421 | -9716.9607 |
| (4,1,1) | 115020.4300 | 105872.5684 | 57773.6783 | 59230.9832 | -9708.0900 | -10663.8417 | -9684.6202 | -10777.7628 |
| (4,1,2) | 115022.4301 | 105852.6433 | 57700.6929 | 59233.0747 | -9705.2868 | -10661.9264 | -9682.3147 | -10775.2462 |
| (4,1,3) | 115022.8806 | 105854.8541 | 57700.8529 | 59223.4632 | -9704.5651 | -10661.0318 | -9680.7598 | -10775.0566 |
| (4,1,4) | 115024.2463 | 105853.4388 | 57702.1753 | 59225.4044 | -9709.8272 | -10650.1241 | -9678.9189 | -10782.0535 |
| (4,1,5) | 115017.1502 | 105853.1161 | 57704.1499 | 59193.0242 | -9703.2448 | -10662.5660 | -9680.0398 | -10778.2270 |
| (4,1,6) | 115018.8983 | 105854.3841 | 57706.0648 | 59194.0189 | -9701.2235 | -10660.6573 | -9678.7913 | -10787.2002 |
| (4,1,7) | 115028.0389 | 105857.1447 | 57690.6227 | 59194.7794 | -9699.2402 | -10657.9803 | -9676.1265 | -10788.4744 |
| (4,1,8) | 115029.5852 | 105858.4990 | 57695.4380 | 59189.4286 | -9697.2592 | -10667.2396 | -9676.1939 | -10776.3095 |
| (4,1,9) | 115031.1814 | 105860.1720 | 57649.5752 | 59192.0448 | -9696.9953 | -10662.0488 | -9685.6714 | -10780.1129 |
| (4,1,10) | 115026.5602 | 105862.1620 | 57619.0814 | 59182.8102 | -9698.8767 | -10661.3448 | -9683.5419 | -10783.4100 |
| (5,1,0) | 115019.0633 | 105868.6284 | 57836.7874 | 59424.0514 | -8824.2708 | -9762.2676 | -8784.6912 | -9833.6759 |
| (5,1,1) | 115020.7394 | 105847.1387 | 57766.6884 | 59232.1762 | -9707.7582 | -10665.4598 | -9683.7415 | -10778.0553 |
| (5,1,2) | 115022.6269 | 105849.5310 | 57701.3618 | 59224.3124 | -9704.1257 | -10660.3831 | -9680.6467 | -10774.3541 |
| (5,1,3) | 115024.6492 | 105852.1350 | 57702.8220 | 59225.8398 | -9704.4165 | -10659.8609 | -9690.9910 | -10783.4761 |
| (5,1,4) | 115025.6221 | 105853.3356 | 57709.8638 | 59227.3629 | -9703.9848 | -10665.2336 | -9681.2041 | -10780.8829 |
| (5,1,5) | 115019.1083 | 105854.9516 | 57697.5402 | 59194.0320 | -9701.2564 | -10665.5611 | -9677.9764 | -10782.6339 |
| (5,1,6) | 115021.3596 | 105856.5492 | 57708.1346 | 59195.5418 | -9699.2483 | -10661.6805 | -9676.3452 | -10786.3415 |
| (5,1,7) | 115023.0938 | 105858.7050 | 57703.6725 | 59197.2488 | -9697.0979 | -10664.5764 | -9675.2115 | -10773.9748 |
| (5,1,8) | 115025.1797 | 105860.2958 | 57662.8398 | 59186.6736 | -9694.2059 | -10662.4666 | -9680.1383 | -10780.3429 |
| (5,1,9) | 115026.9882 | 105862.5364 | 57655.7120 | 59184.8574 | -9693.9638 | -10663.8339 | -9674.0953 | -10778.3522 |
| (5,1,10) | 115028.9573 | 105864.0497 | 57619.9961 | 59186.1457 | -9695.9372 | -10658.3677 | -9688.3692 | -10782.2077 |
| (6,1,0) | 115020.3054 | 105866.4926 | 57837.4470 | 59391.0611 | -8909.2257 | -9830.7705 | -8888.8955 | -9915.2771 |
| (6,1,1) | 115021.9855 | 105849.0358 | 57749.3410 | 59231.4647 | -9706.3840 | -10665.3194 | -9681.7793 | -10777.2766 |
| (6,1,2) | 115023.8176 | 105850.7579 | 57702.3226 | 59225.6172 | -9705.6791 | -10667.8627 | -9680.1925 | -10779.1411 |
| (6,1,3) | 115017.6421 | 105853.2686 | 57704.0753 | 59227.6230 | -9703.6036 | -10665.8966 | -9679.6691 | -10781.1073 |
| (6,1,4) | 115020.2831 | 105854.3870 | 57697.8575 | 59229.3269 | -9700.4563 | -10663.7821 | -9675.0912 | -10781.8947 |
| (6,1,5) | 115020.8321 | 105856.4426 | 57697.2407 | 59194.0566 | -9699.3803 | -10659.3384 | -9676.0593 | -10780.6640 |
| (6,1,6) | 115023.1485 | 105858.1824 | 57707.2157 | 59196.4017 | -9697.1651 | -10663.4755 | -9684.8727 | -10786.6102 |
| (6,1,7) | 115025.1414 | 105859.8087 | 57634.4130 | 59198.4708 | -9695.0510 | -10663.9236 | -9680.3574 | -10776.3156 |
| (6,1,8) | 115026.7387 | 105861.6737 | 57629.5861 | 59185.3281 | -9691.8518 | -10656.3030 | -9680.6276 | -10778.3328 |
| (6,1,9) | 115028.7464 | 105864.0018 | 57630.1221 | 59186.7374 | -9705.8335 | -10662.2907 | -9687.2000 | -10772.9798 |
| (6,1,10) | 115030.5576 | 105865.4331 | 57614.5839 | 59148.3792 | -9701.2172 | -10656.9730 | -9681.9029 | -10782.4163 |
| (7,1,0) | 115020.6915 | 105863.8862 | 57799.4856 | 59323.8153 | -9019.9932 | -9935.5417 | -9001.0857 | -10031.1670 |
| (7,1,1) | 115022.3845 | 105865.6606 | 57776.0379 | 59233.3525 | -9704.6741 | -10663.6132 | -9682.6776 | -10775.3408 |
| (7,1,2) | 115024.2596 | 105852.6962 | 57703.5352 | 59227.6143 | -9703.4964 | -10665.9962 | -9678.2749 | -10777.1464 |
| (7,1,3) | 115019.0092 | 105855.0149 | 57705.6458 | 59229.5816 | -9701.5862 | -10663.6883 | -9688.6306 | -10784.1262 |
| (7,1,4) | 115021.2920 | 105856.8385 | 57704.2539 | 59198.2818 | -9699.2597 | -10661.3948 | -9674.9727 | -10776.2838 |
| (7,1,5) | 115023.8047 | 105858.5327 | 57704.5647 | 59196.1203 | -9697.5489 | -10665.9911 | -9687.3934 | -10779.4183 |
| (7,1,6) | 115025.4973 | 105860.3274 | 57663.3696 | 59191.3390 | -9697.0279 | -10665.1328 | -9677.3094 | -10780.7668 |
| (7,1,7) | 115026.7342 | 105861.7685 | 57633.9960 | 59185.9925 | -9708.4808 | -10662.8130 | -9693.2009 | -10788.5007 |
| (7,1,8) | 115029.0529 | 105864.5249 | 57631.0896 | 59186.5125 | -9707.3475 | -10657.4608 | -9684.0634 | -10785.0310 |
| (7,1,9) | 115030.9522 | 105865.9990 | 57618.7194 | 59187.8586 | -9701.8901 | -10658.0805 | -9683.5554 | -10786.0987 |
| (7,1,10) | 115032.8861 | 105867.3509 | 57616.7049 | 59150.2695 | -9699.0979 | -10658.5763 | -9687.1933 | -10786.4331 |
| (8,1,0) | 115021.8756 | 105865.3620 | 57788.1754 | 59318.3502 | -9072.2701 | -9974.2736 | -9043.2288 | -10059.4941 |
| (8,1,1) | 115023.6440 | 105867.2613 | 57777.9153 | 59320.2947 | -9710.3095 | -10666.5048 | -9690.1660 | -10781.5035 |
| (8,1,2) | 115025.5251 | 105854.1994 | 57705.0813 | 59217.3033 | -9704.2802 | -10665.3293 | -9684.4516 | -10778.9403 |
| (8,1,3) | 115020.7810 | 105856.6640 | 57702.4582 | 59222.4951 | -9701.5091 | -10663.1343 | -9683.0570 | -10784.6281 |
| (8,1,4) | 115022.9409 | 105858.1739 | 57699.1007 | 59179.4694 | -9698.7023 | -10661.1728 | -9688.9117 | -10786.0622 |
| (8,1,5) | 115025.4410 | 105860.6665 | 57665.9806 | 59179.7792 | -9696.7018 | -10659.9388 | -9676.5109 | -10785.8701 |
| (8,1,6) | 115027.6998 | 105861.8873 | 57648.8148 | 59183.7172 | -9694.7320 | -10656.8311 | -9690.4108 | -10786.4943 |
| (8,1,7) | 115028.7297 | 105863.7692 | 57650.8082 | 59168.4243 | -9707.7199 | -10656.9882 | -9677.1125 | -10786.4133 |
| (8,1,8) | 115030.9561 | 105866.2686 | 57620.1064 | 59165.3360 | -9704.3045 | -10662.3253 | -9686.7494 | -10799.3011 |
| (8,1,9) | 115032.4243 | 105867.9320 | 57621.3752 | 59159.3895 | -9697.3726 | -10662.4375 | -9690.3322 | -10788.3032 |
| (8,1,10) | 115034.3899 | 105869.5457 | 57619.1916 | 59159.0615 | -9701.1516 | -10657.9286 | -9682.8288 | -10790.1312 |
| (9,1,0) | 115023.1805 | 105866.4769 | 57753.1476 | 59320.2260 | -9113.3572 | -10028.7159 | -9094.5042 | -10119.1233 |
| (9,1,1) | 115025.0317 | 105868.3549 | 57672.9555 | 59322.2890 | -9708.3786 | -10665.7023 | -9688.2319 | -10782.9285 |
| (9,1,2) | 115026.9272 | 105870.2395 | 57674.8867 | 59218.5910 | -9706.3307 | -10664.0495 | -9686.4910 | -10779.1103 |
| (9,1,3) | 115028.9736 | 105858.5595 | 57673.6469 | 59184.6166 | -9702.6094 | -10661.1873 | -9683.8473 | -10780.6470 |
| (9,1,4) | 115024.8813 | 105860.1838 | 57643.5177 | 59174.2417 | -9703.7446 | -10661.6776 | -9682.8268 | -10783.4990 |
| (9,1,5) | 115027.0454 | 105862.1618 | 57629.9315 | 59176.5075 | -9699.4143 | -10661.6791 | -9692.3048 | -10786.8308 |
| (9,1,6) | 115028.9832 | 105864.2318 | 57650.7714 | 59172.5341 | -9706.8596 | -10656.4253 | -9686.2851 | -10785.5840 |
| (9,1,7) | 115031.1752 | 105865.7155 | 57652.7493 | 59172.3508 | -9707.3334 | -10668.3569 | -9688.0232 | -10792.3814 |
| (9,1,8) | 115032.9596 | 105868.5855 | 57617.2281 | 59176.0128 | -9693.8217 | -10660.1853 | -9688.7324 | -10790.7968 |
| (9,1,9) | 115034.3899 | 105869.5326 | 57623.6934 | 59159.1259 | -9700.8335 | -10656.6437 | -9687.0375 | -10790.0050 |
| (9,1,10) | 115036.7821 | 105871.6481 | 57620.0883 | 59160.0672 | -9701.1860 | -10649.9677 | -9680.4081 | -10784.4320 |
| (10,1,0) | 115024.8792 | 105867.7373 | 57718.8656 | 59322.2036 | -9149.2580 | -10075.3764 | -9127.5687 | -10176.0427 |
| (10,1,1) | 115026.7223 | 105869.5232 | 57674.8480 | 59316.0550 | -9706.3839 | -10663.9095 | -9686.9241 | -10781.6441 |
| (10,1,2) | 115028.6451 | 105857.6853 | 57676.8934 | 59219.6741 | -9704.5186 | -10662.1900 | -9684.5370 | -10778.9355 |
| (10,1,3) | 115030.6958 | 105860.4809 | 57678.8748 | 59213.0122 | -9702.3492 | -10659.7435 | -9683.1932 | -10778.5968 |
| (10,1,4) | 115026.8378 | 105862.1287 | 57643.9993 | 59175.7529 | -9700.5076 | -10658.2959 | -9683.2860 | -10776.7023 |
| (10,1,5) | 115029.0309 | 105863.9747 | 57635.5908 | 59177.2304 | -9698.5056 | -10656.3117 | -9688.0982 | -10785.7875 |
| (10,1,6) | 115030.7539 | 105865.6881 | 57617.1662 | 59173.0912 | -9696.5039 | -10654.4438 | -9684.4949 | -10788.6632 |
| (10,1,7) | 115032.9634 | 105867.6907 | 57619.2801 | 59173.2048 | -9705.3408 | -10663.1457 | -9685.5297 | -10795.8763 |
| (10,1,8) | 115034.9374 | 105869.9132 | 57619.7129 | 59173.7937 | -9704.1919 | -10660.8390 | -9684.1929 | -10776.4360 |
| (10,1,9) | 115036.2598 | 105871.5377 | 57623.2393 | 59159.9002 | -9702.6948 | -10656.6160 | -9682.0089 | -10781.5958 |
| (10,1,10) | 115038.7388 | 105873.6090 | 57624.6974 | 59165.6736 | -9696.6056 | -10657.5096 | -9682.9081 | -10773.8528 |
| *ABP, arterial blood pressure; AIC, Akaike Information Criterion; ARIMA, autoregressive integrative moving average; COx, cerebral oximetry index with CPP; COx-a, cerebral oximetry index with ABP; CPP, cerebral perfusion pressure; rSO_2_, regional cerebral oxygen saturation; TBI-GLR, traumatic brain injury patient group without bifrontal lobe pathology.* | | | | | | | | |

File S5h: Personalized ARIMA models P-Order and Q-Order based on AIC – TBI-GLR Patient Example

| **Personalized ARIMA P-Orders** | | | | | | | | |
| --- | --- | --- | --- | --- | --- | --- | --- | --- |
| **Data Resolution** | **ABP** | **CPP** | **rSO_2__L** | **rSO_2__R** | **COx_L** | **COx_R** | **COx-a_L** | **COx-a_R** |
| 10-Second | 6 | 9 | 10 | 7 | 10 | 9 | 9 | 9 |
| 1-Minute | 3 | 2 | 3 | 6 | 1 | 1 | 8 | 8 |
| 5-Minute | 10 | 10 | 7 | 10 | 8 | 3 | 10 | 3 |
| **Personalized ARIMA Q-Orders** | | | | | | | | |
| **Data Resolution** | **ABP** | **CPP** | **rSO_2__L** | **rSO_2__R** | **COx_L** | **COx_R** | **COx-a_L** | **COx-a_R** |
| 10-Second | 10 | 10 | 10 | 10 | 10 | 10 | 10 | 10 |
| 1-Minute | 0 | 2 | 10 | 10 | 1 | 7 | 9 | 7 |
| 5-Minute | 9 | 10 | 10 | 10 | 9 | 1 | 8 | 1 |
| *ABP, arterial blood pressure; AIC, Akaike Information Criterion; ARIMA, autoregressive integrative moving average; COx, cerebral oximetry index with CPP; COx-a, cerebral oximetry index with ABP; CPP, cerebral perfusion pressure; rSO_2_, regional cerebral oxygen saturation; TBI-GLR, traumatic brain injury patient group without bifrontal lobe pathology.* | | | | | | | | |

File S5i: ADF and KPSS results showing stationary vs non-stationary vs NA for physiologic signals – Original and 1st order differenced HC, SP, and TBI-GLR Data

| **ADF results for non-differenced data** | | | | | | | | | | | | | |
| --- | --- | --- | --- | --- | --- | --- | --- | --- | --- | --- | --- | --- | --- |
| **Dataset** | **Time Resolution** | **ABP** | | | **CPP** | | | **rSO_2__L** | | | **rSO_2__R** | | |
|  |  | **Stationary** | **Non-stationary** | **NA** | **Stationary** | **Non-stationary** | **NA** | **Stationary** | **Non-stationary** | **NA** | **Stationary** | **Non-stationary** | **NA** |
| HC | 10-second | 86 | 16 | 0 | – | – | – | 82 | 20 | 0 | 91 | 11 | 0 |
|  | 1-minute | 18 | 84 | 0 | – | – | – | 17 | 85 | 0 | 22 | 80 | 0 |
|  | 5-minute | 10 | 13 | 79 | – | – | – | 9 | 15 | 78 | 8 | 16 | 78 |
| SP | 10-second | 23 | 4 | 0 | – | – | – | 13 | 14 | 0 | 12 | 15 | 0 |
|  | 1-minute | 21 | 6 | 0 | – | – | – | 7 | 20 | 0 | 8 | 19 | 0 |
|  | 5-minute | 11 | 16 | 0 | – | – | – | 4 | 23 | 0 | 6 | 21 | 0 |
| TBI-GLR | 10-second | 64 | 0 | 0 | 63 | 1 | 0 | 60 | 4 | 0 | 58 | 6 | 0 |
|  | 1-minute | 64 | 0 | 0 | 61 | 3 | 0 | 57 | 7 | 0 | 57 | 7 | 0 |
|  | 5-minute | 63 | 1 | 0 | 57 | 6 | 1 | 52 | 12 | 0 | 51 | 13 | 0 |
| **ADF results for 1^st^ order differenced data** | | | | | | | | | | | | | |
| **Dataset** | **Time Resolution** | **ABP** | | | **CPP** | | | **rSO_2__L** | | | **rSO_2__R** | | |
|  |  | **Stationary** | **Non-stationary** | **NA** | **Stationary** | **Non-stationary** | **NA** | **Stationary** | **Non-stationary** | **NA** | **Stationary** | **Non-stationary** | **NA** |
| HC | 10-second | 102 | 0 | 0 | – | – | – | 102 | 0 | 0 | 102 | 0 | 0 |
|  | 1-minute | 93 | 9 | 0 | – | – | – | 90 | 12 | 0 | 87 | 15 | 0 |
|  | 5-minute | 3 | 4 | 95 | – | – | – | 2 | 6 | 94 | 1 | 7 | 94 |
| SP | 10-second | 27 | 0 | 0 | – | – | – | 27 | 0 | 0 | 27 | 0 | 0 |
|  | 1-minute | 27 | 0 | 0 | – | – | – | 27 | 0 | 0 | 27 | 0 | 0 |
|  | 5-minute | 25 | 2 | 0 | – | – | – | 16 | 11 | 0 | 16 | 11 | 0 |
| TBI-GLR | 10-second | 64 | 0 | 0 | 64 | 0 | 0 | 64 | 0 | 0 | 64 | 0 | 0 |
|  | 1-minute | 64 | 0 | 0 | 64 | 0 | 0 | 63 | 1 | 0 | 64 | 0 | 0 |
|  | 5-minute | 64 | 0 | 0 | 62 | 1 | 1 | 64 | 0 | 0 | 64 | 0 | 0 |
| **KPSS results for non-differenced data** | | | | | | | | | | | | | |
| **Dataset** | **Time Resolution** | **ABP** | | | **CPP** | | | **rSO_2__L** | | | **rSO_2__R** | | |
|  |  | **Stationary** | **Non-stationary** | **NA** | **Stationary** | **Non-stationary** | **NA** | **Stationary** | **Non-stationary** | **NA** | **Stationary** | **Non-stationary** | **NA** |
| HC | 10-second | 29 | 73 | 0 | – | – | – | 26 | 76 | 0 | 20 | 82 | 0 |
|  | 1-minute | 56 | 46 | 0 | – | – | – | 57 | 45 | 0 | 58 | 44 | 0 |
|  | 5-minute | 101 | 1 | 0 | – | – | – | 102 | 0 | 0 | 101 | 1 | 0 |
| SP | 10-second | 1 | 26 | 0 | – | – | – | 0 | 27 | 0 | 1 | 26 | 0 |
|  | 1-minute | 12 | 15 | 0 | – | – | – | 6 | 21 | 0 | 7 | 20 | 0 |
|  | 5-minute | 17 | 10 | 0 | – | – | – | 15 | 12 | 0 | 13 | 14 | 0 |
| TBI-GLR | 10-second | 0 | 64 | 0 | 0 | 64 | 0 | 0 | 64 | 0 | 0 | 64 | 0 |
|  | 1-minute | 3 | 61 | 0 | 6 | 58 | 0 | 1 | 63 | 0 | 0 | 64 | 0 |
|  | 5-minute | 13 | 51 | 0 | 14 | 50 | 0 | 7 | 57 | 0 | 7 | 57 | 0 |
| **KPSS results for 1^st^ order differenced data** | | | | | | | | | | | | | |
| **Dataset** | **Time Resolution** | **ABP** | | | **CPP** | | | **rSO_2__L** | | | **rSO_2__R** | | |
|  |  | **Stationary** | **Non-stationary** | **NA** | **Stationary** | **Non-stationary** | **NA** | **Stationary** | **Non-stationary** | **NA** | **Stationary** | **Non-stationary** | **NA** |
| HC | 10-second | 102 | 0 | 0 | – | – | – | 102 | 0 | 0 | 102 | 0 | 0 |
|  | 1-minute | 102 | 0 | 0 | – | – | – | 100 | 2 | 0 | 101 | 1 | 0 |
|  | 5-minute | 99 | 3 | 0 | – | – | – | 98 | 4 | 0 | 97 | 5 | 0 |
| SP | 10-second | 27 | 0 | 0 | – | – | – | 25 | 2 | 0 | 24 | 3 | 0 |
|  | 1-minute | 27 | 0 | 0 | – | – | – | 24 | 3 | 0 | 25 | 2 | 0 |
|  | 5-minute | 27 | 0 | 0 | – | – | – | 25 | 2 | 0 | 26 | 1 | 0 |
| TBI-GLR | 10-second | 64 | 0 | 0 | 61 | 3 | 0 | 63 | 1 | 0 | 62 | 2 | 0 |
|  | 1-minute | 64 | 0 | 0 | 63 | 1 | 0 | 62 | 2 | 0 | 60 | 4 | 0 |
|  | 5-minute | 64 | 0 | 0 | 64 | 0 | 0 | 61 | 3 | 0 | 62 | 2 | 0 |
| *ABP, arterial blood pressure; ADF, Augmented Dickey-Fuller; COx, cerebral oximetry index with CPP; COx-a, cerebral oximetry index with ABP; CPP, cerebral perfusion pressure; HC, healthy control volunteer group; rSO_2_, regional cerebral oxygen saturation; SP, elective spinal surgery patient group; TBI-GLR, traumatic brain injury patient group without bifrontal lobe pathology.* | | | | | | | | | | | | | |

File S5j: ADF and KPSS results showing stationary vs non-stationary vs NA for physiologic signals – Original and 1st order differenced TBI-GL, TBI-GR, and TBI-BLR Data

| **ADF results for non-differenced data** | | | | | | | | | | | | | | | | | | | | | | | | | |
| --- | --- | --- | --- | --- | --- | --- | --- | --- | --- | --- | --- | --- | --- | --- | --- | --- | --- | --- | --- | --- | --- | --- | --- | --- | --- |
| **Dataset** | **Time Resolution** | **ABP** | | | **CPP** | | | **rSO_2__L** | | | **rSO_2__R** | | | **COx_L** | | | **COx_R** | | | **COx-a_L** | | | **COx-a_R** | | |
|  |  | **Stationary** | **Non-stationary** | **NA** | **Stationary** | **Non-stationary** | **NA** | **Stationary** | **Non-stationary** | **NA** | **Stationary** | **Non-stationary** | **NA** | **Stationary** | **Non-stationary** | **NA** | **Stationary** | **Non-stationary** | **NA** | **Stationary** | **Non-stationary** | **NA** | **Stationary** | **Non-stationary** | **NA** |
| TBI-GL | 10-second | 15 | 0 | 0 | 15 | 0 | 0 | 15 | 0 | 0 | 14 | 1 | 0 | 15 | 0 | 0 | 15 | 0 | 0 | 15 | 0 | 0 | 15 | 0 | 0 |
|  | 1-minute | 15 | 0 | 0 | 15 | 0 | 0 | 14 | 1 | 0 | 14 | 1 | 0 | 15 | 0 | 0 | 15 | 0 | 0 | 15 | 0 | 0 | 15 | 0 | 0 |
|  | 5-minute | 15 | 0 | 0 | 15 | 0 | 0 | 14 | 1 | 0 | 10 | 5 | 0 | 15 | 0 | 0 | 15 | 0 | 0 | 15 | 0 | 0 | 15 | 0 | 0 |
| TBI-GR | 10-second | 11 | 0 | 0 | 11 | 0 | 0 | 10 | 1 | 0 | 10 | 1 | 0 | 11 | 0 | 0 | 11 | 0 | 0 | 11 | 0 | 0 | 11 | 0 | 0 |
|  | 1-minute | 11 | 0 | 0 | 11 | 0 | 0 | 8 | 3 | 0 | 10 | 1 | 0 | 11 | 0 | 0 | 11 | 0 | 0 | 11 | 0 | 0 | 11 | 0 | 0 |
|  | 5-minute | 10 | 1 | 0 | 10 | 1 | 0 | 5 | 6 | 0 | 9 | 2 | 0 | 11 | 0 | 0 | 11 | 0 | 0 | 11 | 0 | 0 | 11 | 0 | 0 |
| TBI-BLR | 10-second | 5 | 0 | 0 | 5 | 0 | 0 | 4 | 1 | 0 | 5 | 0 | 0 | 5 | 0 | 0 | 5 | 0 | 0 | 5 | 0 | 0 | 5 | 0 | 0 |
|  | 1-minute | 5 | 0 | 0 | 5 | 0 | 0 | 4 | 1 | 0 | 5 | 0 | 0 | 5 | 0 | 0 | 5 | 0 | 0 | 5 | 0 | 0 | 5 | 0 | 0 |
|  | 5-minute | 4 | 1 | 0 | 4 | 1 | 0 | 4 | 1 | 0 | 4 | 1 | 0 | 5 | 0 | 0 | 5 | 0 | 0 | 5 | 0 | 0 | 5 | 0 | 0 |
| **ADF results for 1^st^ order differenced data** | | | | | | | | | | | | | | | | | | | | | | | | | |
| **Dataset** | **Time Resolution** | **ABP** | | | **CPP** | | | **rSO_2__L** | | | **rSO_2__R** | | | **COx_L** | | | **COx_R** | | | **COx-a_L** | | | **COx-a_R** | | |
|  |  | **Stationary** | **Non-stationary** | **NA** | **Stationary** | **Non-stationary** | **NA** | **Stationary** | **Non-stationary** | **NA** | **Stationary** | **Non-stationary** | **NA** | **Stationary** | **Non-stationary** | **NA** | **Stationary** | **Non-stationary** | **NA** | **Stationary** | **Non-stationary** | **NA** | **Stationary** | **Non-stationary** | **NA** |
| TBI-GL | 10-second | 15 | 0 | 0 | 15 | 0 | 0 | 15 | 0 | 0 | 15 | 0 | 0 | 15 | 0 | 0 | 15 | 0 | 0 | 15 | 0 | 0 | 15 | 0 | 0 |
|  | 1-minute | 15 | 0 | 0 | 15 | 0 | 0 | 15 | 0 | 0 | 15 | 0 | 0 | 15 | 0 | 0 | 15 | 0 | 0 | 15 | 0 | 0 | 15 | 0 | 0 |
|  | 5-minute | 15 | 0 | 0 | 15 | 0 | 0 | 15 | 0 | 0 | 15 | 0 | 0 | 15 | 0 | 0 | 15 | 0 | 0 | 15 | 0 | 0 | 15 | 0 | 0 |
| TBI-GR | 10-second | 11 | 0 | 0 | 11 | 0 | 0 | 11 | 0 | 0 | 11 | 0 | 0 | 11 | 0 | 0 | 11 | 0 | 0 | 11 | 0 | 0 | 11 | 0 | 0 |
|  | 1-minute | 11 | 0 | 0 | 11 | 0 | 0 | 11 | 0 | 0 | 11 | 0 | 0 | 11 | 0 | 0 | 11 | 0 | 0 | 11 | 0 | 0 | 11 | 0 | 0 |
|  | 5-minute | 11 | 0 | 0 | 11 | 0 | 0 | 11 | 0 | 0 | 11 | 0 | 0 | 11 | 0 | 0 | 11 | 0 | 0 | 11 | 0 | 0 | 11 | 0 | 0 |
| TBI-BLR | 10-second | 5 | 0 | 0 | 5 | 0 | 0 | 5 | 0 | 0 | 5 | 0 | 0 | 5 | 0 | 0 | 5 | 0 | 0 | 5 | 0 | 0 | 5 | 0 | 0 |
|  | 1-minute | 5 | 0 | 0 | 5 | 0 | 0 | 5 | 0 | 0 | 5 | 0 | 0 | 5 | 0 | 0 | 5 | 0 | 0 | 5 | 0 | 0 | 5 | 0 | 0 |
|  | 5-minute | 5 | 0 | 0 | 5 | 0 | 0 | 5 | 0 | 0 | 5 | 0 | 0 | 5 | 0 | 0 | 5 | 0 | 0 | 5 | 0 | 0 | 5 | 0 | 0 |
| **KPSS results for non-differenced data** | | | | | | | | | | | | | | | | | | | | | | | | | |
| **Dataset** | **Time Resolution** | **ABP** | | | **CPP** | | | **rSO_2__L** | | | **rSO_2__R** | | | **COx_L** | | | **COx_R** | | | **COx-a_L** | | | **COx-a_R** | | |
|  |  | **Stationary** | **Non-stationary** | **NA** | **Stationary** | **Non-stationary** | **NA** | **Stationary** | **Non-stationary** | **NA** | **Stationary** | **Non-stationary** | **NA** | **Stationary** | **Non-stationary** | **NA** | **Stationary** | **Non-stationary** | **NA** | **Stationary** | **Non-stationary** | **NA** | **Stationary** | **Non-stationary** | **NA** |
| TBI-GL | 10-second | 1 | 14 | 0 | 0 | 15 | 0 | 0 | 15 | 0 | 0 | 15 | 0 | 3 | 12 | 0 | 1 | 14 | 0 | 2 | 13 | 0 | 1 | 14 | 0 |
|  | 1-minute | 1 | 14 | 0 | 0 | 15 | 0 | 0 | 15 | 0 | 0 | 15 | 0 | 4 | 11 | 0 | 5 | 10 | 0 | 4 | 11 | 0 | 4 | 11 | 0 |
|  | 5-minute | 2 | 13 | 0 | 3 | 12 | 0 | 1 | 14 | 0 | 2 | 13 | 0 | 6 | 9 | 0 | 7 | 8 | 0 | 6 | 9 | 0 | 7 | 8 | 0 |
| TBI-GR | 10-second | 0 | 11 | 0 | 0 | 11 | 0 | 0 | 11 | 0 | 0 | 11 | 0 | 3 | 8 | 0 | 0 | 11 | 0 | 2 | 9 | 0 | 1 | 10 | 0 |
|  | 1-minute | 0 | 11 | 0 | 0 | 11 | 0 | 0 | 11 | 0 | 0 | 11 | 0 | 6 | 5 | 0 | 2 | 9 | 0 | 5 | 6 | 0 | 3 | 8 | 0 |
|  | 5-minute | 1 | 10 | 0 | 2 | 9 | 0 | 2 | 9 | 0 | 1 | 10 | 0 | 8 | 3 | 0 | 4 | 7 | 0 | 8 | 3 | 0 | 5 | 6 | 0 |
| TBI-BLR | 10-second | 0 | 5 | 0 | 0 | 5 | 0 | 0 | 5 | 0 | 0 | 5 | 0 | 0 | 5 | 0 | 0 | 5 | 0 | 0 | 5 | 0 | 0 | 5 | 0 |
|  | 1-minute | 0 | 5 | 0 | 0 | 5 | 0 | 0 | 5 | 0 | 0 | 5 | 0 | 0 | 5 | 0 | 0 | 5 | 0 | 0 | 5 | 0 | 0 | 5 | 0 |
|  | 5-minute | 1 | 4 | 0 | 1 | 4 | 0 | 1 | 4 | 0 | 0 | 5 | 0 | 0 | 5 | 0 | 0 | 5 | 0 | 0 | 5 | 0 | 0 | 5 | 0 |
| **KPSS results for 1^st^ order differenced data** | | | | | | | | | | | | | | | | | | | | | | | | | |
| **Dataset** | **Time Resolution** | **ABP** | | | **CPP** | | | **rSO_2__L** | | | **rSO_2__R** | | | **COx_L** | | | **COx_R** | | | **COx-a_L** | | | **COx-a_R** | | |
|  |  | **Stationary** | **Non-stationary** | **NA** | **Stationary** | **Non-stationary** | **NA** | **Stationary** | **Non-stationary** | **NA** | **Stationary** | **Non-stationary** | **NA** | **Stationary** | **Non-stationary** | **NA** | **Stationary** | **Non-stationary** | **NA** | **Stationary** | **Non-stationary** | **NA** | **Stationary** | **Non-stationary** | **NA** |
| TBI-GL | 10-second | 15 | 0 | 0 | 15 | 0 | 0 | 15 | 0 | 0 | 15 | 0 | 0 | 15 | 0 | 0 | 15 | 0 | 0 | 15 | 0 | 0 | 15 | 0 | 0 |
|  | 1-minute | 15 | 0 | 0 | 15 | 0 | 0 | 15 | 0 | 0 | 15 | 0 | 0 | 15 | 0 | 0 | 15 | 0 | 0 | 15 | 0 | 0 | 15 | 0 | 0 |
|  | 5-minute | 15 | 0 | 0 | 15 | 0 | 0 | 15 | 0 | 0 | 15 | 0 | 0 | 15 | 0 | 0 | 15 | 0 | 0 | 15 | 0 | 0 | 15 | 0 | 0 |
| TBI-GR | 10-second | 11 | 0 | 0 | 11 | 0 | 0 | 11 | 0 | 0 | 11 | 0 | 0 | 11 | 0 | 0 | 11 | 0 | 0 | 11 | 0 | 0 | 11 | 0 | 0 |
|  | 1-minute | 11 | 0 | 0 | 11 | 0 | 0 | 11 | 0 | 0 | 11 | 0 | 0 | 11 | 0 | 0 | 11 | 0 | 0 | 11 | 0 | 0 | 11 | 0 | 0 |
|  | 5-minute | 11 | 0 | 0 | 11 | 0 | 0 | 11 | 0 | 0 | 11 | 0 | 0 | 11 | 0 | 0 | 11 | 0 | 0 | 11 | 0 | 0 | 11 | 0 | 0 |
| TBI-BLR | 10-second | 5 | 0 | 0 | 4 | 1 | 0 | 5 | 0 | 0 | 5 | 0 | 0 | 5 | 0 | 0 | 5 | 0 | 0 | 5 | 0 | 0 | 5 | 0 | 0 |
|  | 1-minute | 5 | 0 | 0 | 5 | 0 | 0 | 5 | 0 | 0 | 5 | 0 | 0 | 5 | 0 | 0 | 5 | 0 | 0 | 5 | 0 | 0 | 5 | 0 | 0 |
|  | 5-minute | 4 | 1 | 0 | 5 | 0 | 0 | 5 | 0 | 0 | 5 | 0 | 0 | 5 | 0 | 0 | 5 | 0 | 0 | 5 | 0 | 0 | 5 | 0 | 0 |
| *ABP, arterial blood pressure; ADF, Augmented Dickey-Fuller; COx, cerebral oximetry index with CPP; COx-a, cerebral oximetry index with ABP; CPP, cerebral perfusion pressure; rSO_2_, regional cerebral oxygen saturation; TBI-BLR, traumatic brain injury patient group with bifrontal lobe pathology; TBI-GL, traumatic brain injury patient group without left frontal lobe pathology; TBI-GLR, traumatic brain injury patient group without bifrontal lobe pathology; TBI-GR, traumatic brain injury patient group without right frontal lobe pathology.* | | | | | | | | | | | | | | | | | | | | | | | | | |

File S5k: Personalized ARIMA P-Orders based on AIC for HC, SP, and TBI-GLR Populations – 10-Second Data Resolution Example

| **HC Patient** | **rSO_2__L** | **rSO_2__R** | **COx-a_L** | **COx-a_R** |  | **SP Patient** | **rSO_2__L** | **rSO_2__R** | **COx-a_L** | **COx-a_R** |  | **TBI-GLR Patient** | **rSO_2__L** | **rSO_2__R** | **COx_L** | **COx_R** | **COx-a_L** | **COx-a_R** |
| --- | --- | --- | --- | --- | --- | --- | --- | --- | --- | --- | --- | --- | --- | --- | --- | --- | --- | --- |
| 1 | 1 | 3 | 1 | 2 |  | 1 | 2 | 3 | 6 | 8 |  | 1 | 9 | 10 | 9 | 6 | 6 | 6 |
| 2 | 3 | 4 | 7 | 2 |  | 2 | 8 | 9 | 6 | 8 |  | 2 | 3 | 7 | 7 | 7 | 3 | 6 |
| 3 | 2 | 1 | 3 | 2 |  | 3 | 8 | 8 | 9 | 6 |  | 3 | 1 | 8 | 9 | 7 | 9 | 9 |
| 4 | 2 | 1 | 5 | 6 |  | 4 | 1 | 9 | 10 | 4 |  | 4 | 7 | 5 | 4 | 8 | 4 | 7 |
| 5 | 4 | 6 | 3 | 1 |  | 5 | 1 | 7 | 8 | 7 |  | 5 | 10 | 3 | 6 | 1 | 7 | 8 |
| 6 | 1 | 2 | 2 | 6 |  | 6 | 4 | 3 | 2 | 2 |  | 6 | 9 | 6 | 9 | 6 | 8 | 6 |
| 7 | 4 | 2 | 1 | 6 |  | 7 | 7 | 10 | 6 | 5 |  | 7 | 10 | 10 | 10 | 7 | 8 | 6 |
| 8 | 2 | 2 | 8 | 10 |  | 8 | 2 | 8 | 9 | 5 |  | 8 | 8 | 10 | 9 | 6 | 5 | 7 |
| 9 | 3 | 3 | 2 | 1 |  | 9 | 6 | 1 | 6 | 6 |  | 9 | 10 | 5 | 7 | 7 | 8 | 6 |
| 10 | 1 | 6 | 4 | 4 |  | 10 | 3 | 3 | 8 | 10 |  | 10 | 10 | 6 | 8 | 9 | 8 | 5 |
| 11 | 5 | 3 | 1 | 4 |  | 11 | 10 | 1 | 4 | 6 |  | 11 | 10 | 10 | 10 | 9 | 7 | 6 |
| 12 | 2 | 4 | 1 | 1 |  | 12 | 3 | 10 | 6 | 5 |  | 12 | 10 | 9 | 9 | 7 | 9 | 9 |
| 13 | 6 | 5 | 1 | 6 |  | 13 | 1 | 2 | 4 | 9 |  | 13 | 7 | 10 | 10 | 8 | 7 | 10 |
| 14 | 3 | 2 | 5 | 3 |  | 14 | 8 | 3 | 7 | 6 |  | 14 | 10 | 10 | 4 | 8 | 4 | 6 |
| 15 | 1 | 1 | 1 | 1 |  | 15 | 3 | 6 | 5 | 4 |  | 15 | 8 | 8 | 9 | 8 | 9 | 7 |
| 16 | 1 | 3 | 2 | 1 |  | 16 | 9 | 1 | 4 | 2 |  | 16 | 10 | 6 | 8 | 10 | 8 | 8 |
| 17 | 4 | 3 | 4 | 1 |  | 17 | 10 | 10 | 9 | 4 |  | 17 | 8 | 10 | 7 | 6 | 10 | 6 |
| 18 | 1 | 4 | 2 | 3 |  | 18 | 2 | 6 | 5 | 8 |  | 18 | 8 | 7 | 6 | 8 | 7 | 10 |
| 19 | 2 | 2 | 3 | 1 |  | 19 | 5 | 7 | 5 | 10 |  | 19 | 6 | 8 | 10 | 8 | 9 | 7 |
| 20 | 1 | 2 | 3 | 6 |  | 20 | 8 | 8 | 9 | 8 |  | 20 | 8 | 8 | 6 | 9 | 9 | 9 |
| 21 | 2 | 1 | 5 | 9 |  | 21 | 7 | 10 | 10 | 10 |  | 21 | 5 | 8 | 8 | 6 | 7 | 10 |
| 22 | 2 | 1 | 1 | 1 |  | 22 | 2 | 6 | 4 | 3 |  | 22 | 7 | 10 | 10 | 4 | 8 | 9 |
| 23 | 1 | 2 | 1 | 2 |  | 23 | 4 | 3 | 6 | 8 |  | 23 | 10 | 9 | 6 | 8 | 7 | 6 |
| 24 | 1 | 2 | 4 | 1 |  | 24 | 8 | 4 | 9 | 6 |  | 24 | 3 | 2 | 10 | 9 | 9 | 9 |
| 25 | 3 | 2 | 4 | 1 |  | 25 | 3 | 1 | 8 | 8 |  | 25 | 1 | 4 | 6 | 8 | 6 | 8 |
| 26 | 4 | 2 | 6 | 8 |  | 26 | 6 | 4 | 5 | 7 |  | 26 | 8 | 10 | 9 | 9 | 6 | 10 |
| 27 | 1 | 2 | 10 | 3 |  | 27 | 10 | 5 | 6 | 10 |  | 27 | 6 | 2 | 7 | 9 | 4 | 4 |
| 28 | 10 | 1 | 1 | 2 |  |  |  |  |  |  |  | 28 | 7 | 10 | 10 | 10 | 10 | 10 |
| 29 | 4 | 2 | 1 | 4 |  |  |  |  |  |  |  | 29 | 6 | 7 | 10 | 10 | 10 | 7 |
| 30 | 1 | 1 | 1 | 5 |  |  |  |  |  |  |  | 30 | 6 | 5 | 6 | 7 | 7 | 10 |
| 31 | 2 | 2 | 3 | 4 |  |  |  |  |  |  |  | 31 | 8 | 7 | 9 | 9 | 8 | 9 |
| 32 | 1 | 6 | 3 | 2 |  |  |  |  |  |  |  | 32 | 5 | 10 | 7 | 6 | 8 | 6 |
| 33 | 1 | 1 | 4 | 1 |  |  |  |  |  |  |  | 33 | 9 | 9 | 7 | 8 | 9 | 5 |
| 34 | 8 | 2 | 1 | 1 |  |  |  |  |  |  |  | 34 | 2 | 5 | 9 | 10 | 9 | 5 |
| 35 | 2 | 1 | 5 | 4 |  |  |  |  |  |  |  | 35 | 10 | 10 | 7 | 8 | 9 | 9 |
| 36 | 1 | 2 | 4 | 3 |  |  |  |  |  |  |  | 36 | 8 | 10 | 4 | 6 | 4 | 5 |
| 37 | 1 | 2 | 2 | 10 |  |  |  |  |  |  |  | 37 | 6 | 9 | 7 | 9 | 7 | 7 |
| 38 | 1 | 1 | 1 | 1 |  |  |  |  |  |  |  | 38 | 5 | 3 | 10 | 9 | 9 | 10 |
| 39 | 1 | 10 | 6 | 4 |  |  |  |  |  |  |  | 39 | 9 | 10 | 8 | 6 | 8 | 6 |
| 40 | 3 | 4 | 9 | 2 |  |  |  |  |  |  |  | 40 | 9 | 2 | 6 | 8 | 8 | 7 |
| 41 | 1 | 1 | 4 | 1 |  |  |  |  |  |  |  | 41 | 10 | 9 | 10 | 9 | 10 | 8 |
| 42 | 1 | 7 | 5 | 7 |  |  |  |  |  |  |  | 42 | 6 | 8 | 8 | 7 | 8 | 9 |
| 43 | 1 | 2 | 2 | 1 |  |  |  |  |  |  |  | 43 | 10 | 10 | 9 | 6 | 7 | 6 |
| 44 | 2 | 2 | 8 | 1 |  |  |  |  |  |  |  | 44 | 9 | 8 | 9 | 9 | 9 | 9 |
| 45 | 2 | 2 | 1 | 10 |  |  |  |  |  |  |  | 45 | 10 | 10 | 9 | 10 | 10 | 9 |
| 46 | 1 | 3 | 4 | 3 |  |  |  |  |  |  |  | 46 | 4 | 4 | 9 | 10 | 9 | 8 |
| 47 | 2 | 3 | 10 | 1 |  |  |  |  |  |  |  | 47 | 5 | 8 | 8 | 10 | 10 | 8 |
| 48 | 4 | 2 | 3 | 3 |  |  |  |  |  |  |  | 48 | 10 | 10 | 9 | 6 | 10 | 8 |
| 49 | 2 | 2 | 3 | 4 |  |  |  |  |  |  |  | 49 | 8 | 1 | 1 | 1 | 7 | 7 |
| 50 | 5 | 1 | 2 | 1 |  |  |  |  |  |  |  | 50 | 10 | 10 | 3 | 6 | 10 | 8 |
| 51 | 7 | 3 | 5 | 3 |  |  |  |  |  |  |  | 51 | 9 | 9 | 9 | 8 | 9 | 9 |
| 52 | 5 | 3 | 6 | 5 |  |  |  |  |  |  |  | 52 | 10 | 10 | 10 | 10 | 8 | 9 |
| 53 | 4 | 6 | 2 | 1 |  |  |  |  |  |  |  | 53 | 1 | 9 | 7 | 7 | 7 | 10 |
| 54 | 2 | 1 | 3 | 1 |  |  |  |  |  |  |  | 54 | 9 | 7 | 6 | 7 | 4 | 7 |
| 55 | 1 | 1 | 2 | 4 |  |  |  |  |  |  |  | 55 | 3 | 5 | 8 | 5 | 10 | 8 |
| 56 | 1 | 1 | 1 | 2 |  |  |  |  |  |  |  | 56 | 9 | 9 | 7 | 9 | 7 | 9 |
| 57 | 6 | 10 | 5 | 7 |  |  |  |  |  |  |  | 57 | 10 | 10 | 10 | 4 | 5 | 3 |
| 58 | 6 | 5 | 1 | 5 |  |  |  |  |  |  |  | 58 | 9 | 10 | 6 | 8 | 10 | 5 |
| 59 | 5 | 4 | 5 | 8 |  |  |  |  |  |  |  | 59 | 10 | 10 | 9 | 10 | 9 | 10 |
| 60 | 1 | 2 | 1 | 3 |  |  |  |  |  |  |  | 60 | 7 | 7 | 7 | 10 | 8 | 9 |
| 61 | 7 | 3 | 1 | 2 |  |  |  |  |  |  |  | 61 | 8 | 9 | 9 | 8 | 7 | 8 |
| 62 | 1 | 2 | 1 | 2 |  |  |  |  |  |  |  | 62 | 10 | 8 | 9 | 10 | 10 | 7 |
| 63 | 2 | 3 | 1 | 1 |  |  |  |  |  |  |  | 63 | 10 | 9 | 4 | 7 | 4 | 5 |
| 64 | 1 | 2 | 2 | 1 |  |  |  |  |  |  |  | 64 | 10 | 7 | 10 | 9 | 9 | 9 |
| 65 | 8 | 1 | 3 | 4 |  |  |  |  |  |  |  |  |  |  |  |  |  |  |
| 66 | 7 | 4 | 1 | 1 |  |  |  |  |  |  |  |  |  |  |  |  |  |  |
| 67 | 4 | 1 | 1 | 9 |  |  |  |  |  |  |  |  |  |  |  |  |  |  |
| 68 | 4 | 1 | 7 | 5 |  |  |  |  |  |  |  |  |  |  |  |  |  |  |
| 69 | 1 | 3 | 2 | 4 |  |  |  |  |  |  |  |  |  |  |  |  |  |  |
| 70 | 4 | 4 | 1 | 4 |  |  |  |  |  |  |  |  |  |  |  |  |  |  |
| 71 | 2 | 4 | 1 | 4 |  |  |  |  |  |  |  |  |  |  |  |  |  |  |
| 72 | 4 | 1 | 2 | 6 |  |  |  |  |  |  |  |  |  |  |  |  |  |  |
| 73 | 3 | 1 | 3 | 5 |  |  |  |  |  |  |  |  |  |  |  |  |  |  |
| 74 | 1 | 6 | 4 | 1 |  |  |  |  |  |  |  |  |  |  |  |  |  |  |
| 75 | 8 | 4 | 6 | 7 |  |  |  |  |  |  |  |  |  |  |  |  |  |  |
| 76 | 2 | 1 | 4 | 9 |  |  |  |  |  |  |  |  |  |  |  |  |  |  |
| 77 | 1 | 2 | 8 | 2 |  |  |  |  |  |  |  |  |  |  |  |  |  |  |
| 78 | 8 | 2 | 2 | 7 |  |  |  |  |  |  |  |  |  |  |  |  |  |  |
| 79 | 1 | 5 | 6 | 1 |  |  |  |  |  |  |  |  |  |  |  |  |  |  |
| 80 | 1 | 7 | 3 | 1 |  |  |  |  |  |  |  |  |  |  |  |  |  |  |
| 81 | 8 | 4 | 2 | 4 |  |  |  |  |  |  |  |  |  |  |  |  |  |  |
| 82 | 1 | 1 | 4 | 2 |  |  |  |  |  |  |  |  |  |  |  |  |  |  |
| 83 | 9 | 2 | 1 | 1 |  |  |  |  |  |  |  |  |  |  |  |  |  |  |
| 84 | 2 | 1 | 4 | 1 |  |  |  |  |  |  |  |  |  |  |  |  |  |  |
| 85 | 2 | 2 | 1 | 2 |  |  |  |  |  |  |  |  |  |  |  |  |  |  |
| 86 | 1 | 1 | 1 | 4 |  |  |  |  |  |  |  |  |  |  |  |  |  |  |
| 87 | 2 | 1 | 4 | 3 |  |  |  |  |  |  |  |  |  |  |  |  |  |  |
| 88 | 9 | 1 | 2 | 1 |  |  |  |  |  |  |  |  |  |  |  |  |  |  |
| 89 | 1 | 9 | 4 | 1 |  |  |  |  |  |  |  |  |  |  |  |  |  |  |
| 90 | 3 | 8 | 1 | 1 |  |  |  |  |  |  |  |  |  |  |  |  |  |  |
| 91 | 3 | 2 | 6 | 6 |  |  |  |  |  |  |  |  |  |  |  |  |  |  |
| 92 | 3 | 1 | 6 | 2 |  |  |  |  |  |  |  |  |  |  |  |  |  |  |
| 93 | 2 | 1 | 2 | 3 |  |  |  |  |  |  |  |  |  |  |  |  |  |  |
| 94 | 1 | 1 | 1 | 5 |  |  |  |  |  |  |  |  |  |  |  |  |  |  |
| 95 | 2 | 2 | 2 | 2 |  |  |  |  |  |  |  |  |  |  |  |  |  |  |
| 96 | 2 | 3 | 3 | 10 |  |  |  |  |  |  |  |  |  |  |  |  |  |  |
| 97 | 2 | 1 | 1 | 5 |  |  |  |  |  |  |  |  |  |  |  |  |  |  |
| 98 | 1 | 3 | 1 | 2 |  |  |  |  |  |  |  |  |  |  |  |  |  |  |
| 99 | 1 | 8 | 2 | 2 |  |  |  |  |  |  |  |  |  |  |  |  |  |  |
| 100 | 1 | 2 | 7 | 5 |  |  |  |  |  |  |  |  |  |  |  |  |  |  |
| 101 | 1 | 1 | 3 | 3 |  |  |  |  |  |  |  |  |  |  |  |  |  |  |
| 102 | 7 | 2 | 4 | 1 |  |  |  |  |  |  |  |  |  |  |  |  |  |  |
|  |  |  |  |  |  |  |  |  |  |  |  |  |  |  |  |  |  |  |
| *AIC, Akaike Information Criterion; ARIMA, autoregressive integrative moving average; COx, cerebral oximetry index with cerebral perfusion pressure; COx-a, cerebral oximetry index with arterial blood pressure; HC, healthy control volunteer group; rSO_2_, regional cerebral oxygen saturation; SP, elective spinal surgery patient group; TBI-GLR, traumatic brain injury patient group without bifrontal lobe pathology.* | | | | | | | | | | | | | | | | | | |

File S5l: Personalized ARIMA P-Orders based on for TBI-GL, TBI-GR, and TBI-BLR Populations – 10-Second Data Resolution Example

| **TBI-GL Patient** | **rSO_2__L** | **rSO_2__R** | **COx_L** | **COx_R** | **COx-a_L** | **COx-a_R** |  | **TBI-GR Patient** | **rSO_2__L** | **rSO_2__R** | **COx_L** | **COx_R** | **COx-a_L** | **COx-a_R** |  | **TBI-BLR Patient** | **rSO_2__L** | **rSO_2__R** | **COx_L** | **COx_R** | **COx-a_L** | **COx-a_R** |
| --- | --- | --- | --- | --- | --- | --- | --- | --- | --- | --- | --- | --- | --- | --- | --- | --- | --- | --- | --- | --- | --- | --- |
| 1 | 9 | 9 | 8 | 8 | 8 | 7 |  | 1 | 4 | 3 | 8 | 9 | 6 | 8 |  | 1 | 8 | 10 | 8 | 5 | 6 | 8 |
| 2 | 10 | 1 | 7 | 10 | 7 | 10 |  | 2 | 10 | 9 | 8 | 10 | 10 | 9 |  | 2 | 10 | 10 | 7 | 10 | 7 | 10 |
| 3 | 3 | 2 | 9 | 9 | 5 | 9 |  | 3 | 5 | 8 | 8 | 7 | 8 | 7 |  | 3 | 10 | 9 | 9 | 8 | 8 | 8 |
| 4 | 2 | 7 | 6 | 10 | 6 | 10 |  | 4 | 9 | 1 | 6 | 9 | 6 | 5 |  | 4 | 10 | 10 | 10 | 9 | 9 | 10 |
| 5 | 2 | 8 | 10 | 4 | 8 | 9 |  | 5 | 9 | 1 | 6 | 8 | 7 | 8 |  | 5 | 10 | 8 | 10 | 8 | 6 | 5 |
| 6 | 6 | 9 | 5 | 8 | 4 | 10 |  | 6 | 9 | 9 | 7 | 6 | 7 | 10 |  |  |  |  |  |  |  |  |
| 7 | 10 | 9 | 6 | 9 | 10 | 10 |  | 7 | 6 | 9 | 8 | 9 | 8 | 8 |  |  |  |  |  |  |  |  |
| 8 | 8 | 9 | 7 | 8 | 7 | 10 |  | 8 | 7 | 1 | 7 | 10 | 9 | 7 |  |  |  |  |  |  |  |  |
| 9 | 3 | 1 | 9 | 9 | 10 | 9 |  | 9 | 1 | 6 | 9 | 8 | 9 | 7 |  |  |  |  |  |  |  |  |
| 10 | 7 | 10 | 8 | 9 | 8 | 7 |  | 10 | 10 | 5 | 9 | 5 | 9 | 5 |  |  |  |  |  |  |  |  |
| 11 | 8 | 1 | 7 | 4 | 7 | 4 |  | 11 | 8 | 8 | 7 | 6 | 6 | 8 |  |  |  |  |  |  |  |  |
| 12 | 9 | 4 | 7 | 8 | 10 | 9 |  |  |  |  |  |  |  |  |  |  |  |  |  |  |  |  |
| 13 | 9 | 10 | 10 | 10 | 7 | 8 |  |  |  |  |  |  |  |  |  |  |  |  |  |  |  |  |
| 14 | 10 | 10 | 8 | 8 | 7 | 10 |  |  |  |  |  |  |  |  |  |  |  |  |  |  |  |  |
| 15 | 10 | 9 | 9 | 9 | 9 | 10 |  |  |  |  |  |  |  |  |  |  |  |  |  |  |  |  |
|  |  |  |  |  |  |  |  |  |  |  |  |  |  |  |  |  |  |  |  |  |  |  |
| *AIC, Akaike Information Criterion; ARIMA, autoregressive integrative moving average; COx, cerebral oximetry index with cerebral perfusion pressure; COx-a, cerebral oximetry index with arterial blood pressure; rSO_2_, regional cerebral oxygen saturation; TBI-BLR, traumatic brain injury patient group with bifrontal lobe pathology; TBI-GL, traumatic brain injury patient group without left frontal lobe pathology; TBI-GR, traumatic brain injury patient group without right frontal lobe pathology.* | | | | | | | | | | | | | | | | | | | | | | |

File S5m: Personalized ARIMA Q-Orders based on AIC for HC, SP, and TBI-GLR Populations – 10-Second Data Resolution Example

| **HC Patient** | **rSO_2__L** | **rSO_2__R** | **COx-a_L** | **COx-a_R** |  | **SP Patient** | **rSO_2__L** | **rSO_2__R** | **COx-a_L** | **COx-a_R** |  | **TBI-GLR Patient** | **rSO_2__L** | **rSO_2__R** | **COx_L** | **COx_R** | **COx-a_L** | **COx-a_R** |
| --- | --- | --- | --- | --- | --- | --- | --- | --- | --- | --- | --- | --- | --- | --- | --- | --- | --- | --- |
| 1 | 1 | 3 | 0 | 0 |  | 1 | 4 | 1 | 10 | 4 |  | 1 | 10 | 4 | 8 | 6 | 6 | 6 |
| 2 | 3 | 4 | 7 | 1 |  | 2 | 10 | 8 | 9 | 7 |  | 2 | 3 | 9 | 4 | 7 | 4 | 10 |
| 3 | 2 | 2 | 4 | 0 |  | 3 | 10 | 10 | 1 | 4 |  | 3 | 3 | 6 | 8 | 7 | 8 | 10 |
| 4 | 2 | 3 | 1 | 1 |  | 4 | 7 | 10 | 10 | 3 |  | 4 | 8 | 5 | 4 | 7 | 3 | 7 |
| 5 | 4 | 5 | 0 | 0 |  | 5 | 9 | 7 | 10 | 10 |  | 5 | 10 | 10 | 6 | 7 | 6 | 10 |
| 6 | 2 | 3 | 2 | 0 |  | 6 | 10 | 4 | 2 | 2 |  | 6 | 10 | 10 | 10 | 7 | 9 | 5 |
| 7 | 5 | 2 | 3 | 4 |  | 7 | 8 | 9 | 5 | 3 |  | 7 | 10 | 10 | 10 | 7 | 8 | 9 |
| 8 | 6 | 3 | 6 | 4 |  | 8 | 2 | 9 | 3 | 1 |  | 8 | 8 | 6 | 3 | 3 | 10 | 6 |
| 9 | 2 | 2 | 1 | 0 |  | 9 | 8 | 1 | 8 | 1 |  | 9 | 10 | 8 | 10 | 10 | 10 | 6 |
| 10 | 1 | 7 | 4 | 3 |  | 10 | 3 | 1 | 2 | 10 |  | 10 | 10 | 5 | 10 | 6 | 4 | 9 |
| 11 | 3 | 3 | 0 | 3 |  | 11 | 10 | 1 | 4 | 5 |  | 11 | 9 | 9 | 9 | 9 | 7 | 10 |
| 12 | 3 | 3 | 0 | 0 |  | 12 | 2 | 10 | 3 | 5 |  | 12 | 10 | 10 | 10 | 10 | 10 | 8 |
| 13 | 6 | 5 | 0 | 4 |  | 13 | 4 | 1 | 5 | 9 |  | 13 | 10 | 10 | 7 | 10 | 10 | 10 |
| 14 | 7 | 3 | 2 | 10 |  | 14 | 8 | 2 | 7 | 1 |  | 14 | 9 | 8 | 4 | 8 | 4 | 1 |
| 15 | 0 | 6 | 0 | 0 |  | 15 | 1 | 4 | 1 | 4 |  | 15 | 10 | 10 | 7 | 9 | 10 | 10 |
| 16 | 0 | 3 | 0 | 0 |  | 16 | 10 | 0 | 1 | 10 |  | 16 | 10 | 10 | 7 | 10 | 7 | 9 |
| 17 | 7 | 6 | 4 | 1 |  | 17 | 10 | 10 | 3 | 3 |  | 17 | 9 | 1 | 10 | 6 | 10 | 5 |
| 18 | 1 | 5 | 2 | 10 |  | 18 | 0 | 7 | 1 | 9 |  | 18 | 6 | 9 | 10 | 10 | 6 | 10 |
| 19 | 4 | 4 | 3 | 0 |  | 19 | 9 | 7 | 6 | 6 |  | 19 | 4 | 7 | 10 | 9 | 10 | 10 |
| 20 | 1 | 0 | 3 | 7 |  | 20 | 4 | 10 | 4 | 5 |  | 20 | 10 | 10 | 10 | 8 | 9 | 10 |
| 21 | 7 | 3 | 5 | 8 |  | 21 | 10 | 10 | 10 | 4 |  | 21 | 7 | 5 | 1 | 9 | 10 | 3 |
| 22 | 2 | 1 | 0 | 0 |  | 22 | 2 | 6 | 4 | 9 |  | 22 | 7 | 10 | 10 | 10 | 10 | 10 |
| 23 | 1 | 2 | 0 | 2 |  | 23 | 5 | 4 | 5 | 9 |  | 23 | 8 | 10 | 7 | 10 | 8 | 8 |
| 24 | 1 | 3 | 4 | 0 |  | 24 | 6 | 4 | 8 | 5 |  | 24 | 3 | 2 | 9 | 10 | 9 | 10 |
| 25 | 0 | 7 | 3 | 0 |  | 25 | 6 | 3 | 8 | 10 |  | 25 | 1 | 7 | 10 | 8 | 8 | 9 |
| 26 | 3 | 0 | 8 | 9 |  | 26 | 4 | 10 | 6 | 5 |  | 26 | 10 | 10 | 6 | 10 | 10 | 2 |
| 27 | 5 | 3 | 8 | 1 |  | 27 | 9 | 7 | 4 | 3 |  | 27 | 6 | 4 | 9 | 10 | 3 | 10 |
| 28 | 1 | 1 | 2 | 6 |  |  |  |  |  |  |  | 28 | 10 | 10 | 10 | 10 | 9 | 10 |
| 29 | 4 | 2 | 0 | 2 |  |  |  |  |  |  |  | 29 | 8 | 1 | 10 | 10 | 10 | 10 |
| 30 | 0 | 1 | 0 | 10 |  |  |  |  |  |  |  | 30 | 2 | 7 | 7 | 6 | 10 | 5 |
| 31 | 2 | 3 | 7 | 9 |  |  |  |  |  |  |  | 31 | 0 | 10 | 6 | 5 | 10 | 5 |
| 32 | 6 | 7 | 2 | 2 |  |  |  |  |  |  |  | 32 | 7 | 6 | 10 | 9 | 10 | 8 |
| 33 | 1 | 2 | 3 | 3 |  |  |  |  |  |  |  | 33 | 8 | 9 | 10 | 10 | 8 | 8 |
| 34 | 10 | 1 | 8 | 1 |  |  |  |  |  |  |  | 34 | 2 | 5 | 2 | 1 | 10 | 5 |
| 35 | 1 | 0 | 5 | 4 |  |  |  |  |  |  |  | 35 | 9 | 10 | 10 | 9 | 8 | 7 |
| 36 | 1 | 2 | 4 | 5 |  |  |  |  |  |  |  | 36 | 10 | 7 | 9 | 9 | 10 | 10 |
| 37 | 2 | 1 | 10 | 7 |  |  |  |  |  |  |  | 37 | 9 | 9 | 10 | 9 | 8 | 10 |
| 38 | 0 | 1 | 0 | 2 |  |  |  |  |  |  |  | 38 | 9 | 10 | 10 | 10 | 8 | 10 |
| 39 | 1 | 10 | 3 | 5 |  |  |  |  |  |  |  | 39 | 10 | 10 | 10 | 10 | 9 | 7 |
| 40 | 3 | 3 | 10 | 0 |  |  |  |  |  |  |  | 40 | 2 | 5 | 4 | 8 | 8 | 7 |
| 41 | 0 | 1 | 3 | 2 |  |  |  |  |  |  |  | 41 | 10 | 9 | 10 | 10 | 10 | 8 |
| 42 | 1 | 5 | 5 | 6 |  |  |  |  |  |  |  | 42 | 4 | 8 | 9 | 8 | 10 | 7 |
| 43 | 3 | 0 | 0 | 0 |  |  |  |  |  |  |  | 43 | 6 | 9 | 10 | 6 | 10 | 9 |
| 44 | 1 | 1 | 10 | 2 |  |  |  |  |  |  |  | 44 | 10 | 10 | 10 | 10 | 10 | 10 |
| 45 | 4 | 1 | 0 | 9 |  |  |  |  |  |  |  | 45 | 10 | 9 | 10 | 9 | 9 | 10 |
| 46 | 1 | 3 | 4 | 10 |  |  |  |  |  |  |  | 46 | 10 | 8 | 10 | 9 | 10 | 10 |
| 47 | 2 | 1 | 2 | 0 |  |  |  |  |  |  |  | 47 | 10 | 10 | 10 | 9 | 10 | 9 |
| 48 | 4 | 1 | 2 | 2 |  |  |  |  |  |  |  | 48 | 10 | 10 | 10 | 9 | 10 | 9 |
| 49 | 4 | 2 | 5 | 0 |  |  |  |  |  |  |  | 49 | 7 | 6 | 0 | 0 | 6 | 10 |
| 50 | 6 | 3 | 0 | 0 |  |  |  |  |  |  |  | 50 | 9 | 10 | 4 | 10 | 9 | 10 |
| 51 | 5 | 5 | 8 | 2 |  |  |  |  |  |  |  | 51 | 7 | 6 | 10 | 10 | 10 | 10 |
| 52 | 2 | 7 | 4 | 2 |  |  |  |  |  |  |  | 52 | 10 | 9 | 10 | 10 | 10 | 10 |
| 53 | 4 | 6 | 4 | 0 |  |  |  |  |  |  |  | 53 | 10 | 10 | 10 | 9 | 7 | 8 |
| 54 | 1 | 4 | 10 | 0 |  |  |  |  |  |  |  | 54 | 9 | 8 | 10 | 4 | 10 | 10 |
| 55 | 1 | 2 | 0 | 1 |  |  |  |  |  |  |  | 55 | 6 | 10 | 1 | 5 | 9 | 2 |
| 56 | 1 | 3 | 0 | 3 |  |  |  |  |  |  |  | 56 | 4 | 10 | 8 | 10 | 10 | 8 |
| 57 | 6 | 8 | 10 | 5 |  |  |  |  |  |  |  | 57 | 10 | 8 | 10 | 1 | 1 | 5 |
| 58 | 2 | 5 | 0 | 4 |  |  |  |  |  |  |  | 58 | 9 | 10 | 9 | 10 | 10 | 10 |
| 59 | 3 | 3 | 2 | 8 |  |  |  |  |  |  |  | 59 | 10 | 10 | 8 | 10 | 8 | 10 |
| 60 | 1 | 2 | 0 | 2 |  |  |  |  |  |  |  | 60 | 10 | 7 | 10 | 10 | 8 | 10 |
| 61 | 10 | 5 | 0 | 3 |  |  |  |  |  |  |  | 61 | 9 | 10 | 10 | 10 | 9 | 10 |
| 62 | 1 | 3 | 0 | 3 |  |  |  |  |  |  |  | 62 | 10 | 10 | 8 | 10 | 10 | 10 |
| 63 | 1 | 2 | 0 | 2 |  |  |  |  |  |  |  | 63 | 10 | 10 | 4 | 10 | 4 | 7 |
| 64 | 0 | 2 | 3 | 4 |  |  |  |  |  |  |  | 64 | 10 | 10 | 10 | 10 | 10 | 10 |
| 65 | 6 | 1 | 4 | 7 |  |  |  |  |  |  |  |  |  |  |  |  |  |  |
| 66 | 1 | 4 | 0 | 8 |  |  |  |  |  |  |  |  |  |  |  |  |  |  |
| 67 | 2 | 1 | 3 | 3 |  |  |  |  |  |  |  |  |  |  |  |  |  |  |
| 68 | 3 | 1 | 6 | 7 |  |  |  |  |  |  |  |  |  |  |  |  |  |  |
| 69 | 1 | 2 | 0 | 0 |  |  |  |  |  |  |  |  |  |  |  |  |  |  |
| 70 | 1 | 5 | 0 | 4 |  |  |  |  |  |  |  |  |  |  |  |  |  |  |
| 71 | 1 | 5 | 0 | 5 |  |  |  |  |  |  |  |  |  |  |  |  |  |  |
| 72 | 10 | 1 | 2 | 3 |  |  |  |  |  |  |  |  |  |  |  |  |  |  |
| 73 | 4 | 2 | 2 | 5 |  |  |  |  |  |  |  |  |  |  |  |  |  |  |
| 74 | 0 | 8 | 0 | 0 |  |  |  |  |  |  |  |  |  |  |  |  |  |  |
| 75 | 8 | 3 | 1 | 3 |  |  |  |  |  |  |  |  |  |  |  |  |  |  |
| 76 | 2 | 1 | 6 | 10 |  |  |  |  |  |  |  |  |  |  |  |  |  |  |
| 77 | 1 | 1 | 2 | 2 |  |  |  |  |  |  |  |  |  |  |  |  |  |  |
| 78 | 5 | 1 | 0 | 3 |  |  |  |  |  |  |  |  |  |  |  |  |  |  |
| 79 | 7 | 5 | 5 | 0 |  |  |  |  |  |  |  |  |  |  |  |  |  |  |
| 80 | 2 | 8 | 2 | 0 |  |  |  |  |  |  |  |  |  |  |  |  |  |  |
| 81 | 2 | 7 | 3 | 3 |  |  |  |  |  |  |  |  |  |  |  |  |  |  |
| 82 | 1 | 1 | 2 | 2 |  |  |  |  |  |  |  |  |  |  |  |  |  |  |
| 83 | 6 | 4 | 0 | 0 |  |  |  |  |  |  |  |  |  |  |  |  |  |  |
| 84 | 1 | 2 | 5 | 0 |  |  |  |  |  |  |  |  |  |  |  |  |  |  |
| 85 | 4 | 6 | 0 | 2 |  |  |  |  |  |  |  |  |  |  |  |  |  |  |
| 86 | 2 | 4 | 0 | 0 |  |  |  |  |  |  |  |  |  |  |  |  |  |  |
| 87 | 3 | 1 | 4 | 3 |  |  |  |  |  |  |  |  |  |  |  |  |  |  |
| 88 | 3 | 3 | 0 | 0 |  |  |  |  |  |  |  |  |  |  |  |  |  |  |
| 89 | 1 | 2 | 0 | 1 |  |  |  |  |  |  |  |  |  |  |  |  |  |  |
| 90 | 3 | 8 | 0 | 3 |  |  |  |  |  |  |  |  |  |  |  |  |  |  |
| 91 | 5 | 2 | 10 | 6 |  |  |  |  |  |  |  |  |  |  |  |  |  |  |
| 92 | 4 | 1 | 5 | 4 |  |  |  |  |  |  |  |  |  |  |  |  |  |  |
| 93 | 1 | 1 | 2 | 0 |  |  |  |  |  |  |  |  |  |  |  |  |  |  |
| 94 | 1 | 1 | 4 | 3 |  |  |  |  |  |  |  |  |  |  |  |  |  |  |
| 95 | 4 | 2 | 1 | 9 |  |  |  |  |  |  |  |  |  |  |  |  |  |  |
| 96 | 1 | 3 | 7 | 10 |  |  |  |  |  |  |  |  |  |  |  |  |  |  |
| 97 | 5 | 1 | 0 | 6 |  |  |  |  |  |  |  |  |  |  |  |  |  |  |
| 98 | 1 | 8 | 0 | 4 |  |  |  |  |  |  |  |  |  |  |  |  |  |  |
| 99 | 9 | 7 | 2 | 2 |  |  |  |  |  |  |  |  |  |  |  |  |  |  |
| 100 | 1 | 4 | 6 | 4 |  |  |  |  |  |  |  |  |  |  |  |  |  |  |
| 101 | 1 | 1 | 3 | 0 |  |  |  |  |  |  |  |  |  |  |  |  |  |  |
| 102 | 5 | 5 | 4 | 2 |  |  |  |  |  |  |  |  |  |  |  |  |  |  |
|  |  |  |  |  |  |  |  |  |  |  |  |  |  |  |  |  |  |  |
| *AIC, Akaike Information Criterion; ARIMA, autoregressive integrative moving average; COx, cerebral oximetry index with cerebral perfusion pressure; COx-a, cerebral oximetry index with arterial blood pressure; HC, healthy control volunteer group; rSO_2_, regional cerebral oxygen saturation; SP, elective spinal surgery patient group; TBI-GLR, traumatic brain injury patient group without bifrontal lobe pathology.* | | | | | | | | | | | | | | | | | | |

File S5n: Personalized ARIMA Q-Orders based on AIC for TBI-GL, TBI-GR, and TBI-BLR Populations – 10-Second Data Resolution Example

| **TBI-GL Patient** | **rSO_2__L** | **rSO_2__R** | **COx_L** | **COx_R** | **COx-a_L** | **COx-a_R** |  | **TBI-GR Patient** | **rSO_2__L** | **rSO_2__R** | **COx_L** | **COx_R** | **COx-a_L** | **COx-a_R** |  | **TBI-BLR Patient** | **rSO_2__L** | **rSO_2__R** | **COx_L** | **COx_R** | **COx-a_L** | **COx-a_R** |
| --- | --- | --- | --- | --- | --- | --- | --- | --- | --- | --- | --- | --- | --- | --- | --- | --- | --- | --- | --- | --- | --- | --- |
| 1 | 8 | 9 | 9 | 8 | 10 | 4 |  | 1 | 10 | 1 | 10 | 9 | 10 | 7 |  | 1 | 8 | 4 | 7 | 4 | 9 | 9 |
| 2 | 10 | 4 | 10 | 10 | 10 | 10 |  | 2 | 10 | 10 | 10 | 10 | 10 | 10 |  | 2 | 10 | 10 | 10 | 10 | 10 | 10 |
| 3 | 2 | 10 | 6 | 4 | 10 | 10 |  | 3 | 5 | 10 | 10 | 7 | 10 | 10 |  | 3 | 9 | 9 | 8 | 10 | 8 | 10 |
| 4 | 6 | 10 | 7 | 10 | 4 | 10 |  | 4 | 3 | 2 | 4 | 4 | 4 | 5 |  | 4 | 10 | 10 | 9 | 10 | 8 | 9 |
| 5 | 1 | 3 | 10 | 10 | 1 | 6 |  | 5 | 8 | 10 | 7 | 9 | 8 | 10 |  | 5 | 10 | 9 | 6 | 10 | 6 | 6 |
| 6 | 10 | 10 | 1 | 1 | 1 | 8 |  | 6 | 1 | 10 | 7 | 9 | 7 | 8 |  |  |  |  |  |  |  |  |
| 7 | 10 | 10 | 10 | 10 | 6 | 10 |  | 7 | 6 | 10 | 10 | 10 | 10 | 8 |  |  |  |  |  |  |  |  |
| 8 | 10 | 2 | 8 | 10 | 8 | 10 |  | 8 | 1 | 2 | 10 | 5 | 9 | 5 |  |  |  |  |  |  |  |  |
| 9 | 6 | 7 | 10 | 10 | 9 | 10 |  | 9 | 0 | 7 | 7 | 10 | 6 | 9 |  |  |  |  |  |  |  |  |
| 10 | 10 | 10 | 7 | 7 | 8 | 7 |  | 10 | 10 | 10 | 10 | 5 | 9 | 5 |  |  |  |  |  |  |  |  |
| 11 | 3 | 1 | 10 | 5 | 10 | 4 |  | 11 | 10 | 10 | 7 | 9 | 7 | 10 |  |  |  |  |  |  |  |  |
| 12 | 9 | 7 | 10 | 6 | 10 | 9 |  |  |  |  |  |  |  |  |  |  |  |  |  |  |  |  |
| 13 | 8 | 10 | 8 | 10 | 8 | 7 |  |  |  |  |  |  |  |  |  |  |  |  |  |  |  |  |
| 14 | 7 | 10 | 8 | 7 | 10 | 10 |  |  |  |  |  |  |  |  |  |  |  |  |  |  |  |  |
| 15 | 9 | 10 | 10 | 10 | 10 | 10 |  |  |  |  |  |  |  |  |  |  |  |  |  |  |  |  |
|  |  |  |  |  |  |  |  |  |  |  |  |  |  |  |  |  |  |  |  |  |  |  |
| *AIC, Akaike Information Criterion; ARIMA, autoregressive integrative moving average; COx, cerebral oximetry index with cerebral perfusion pressure; COx-a, cerebral oximetry index with arterial blood pressure; rSO_2_, regional cerebral oxygen saturation; TBI-BLR, traumatic brain injury patient group with bifrontal lobe pathology; TBI-GL, traumatic brain injury patient group without left frontal lobe pathology; TBI-GR, traumatic brain injury patient group without right frontal lobe pathology.* | | | | | | | | | | | | | | | | | | | | | | |

File S5o: Regional Hemispheric Disparity of Personalized ARIMA p-orders based on AIC for HC, SP, and TBI-GLR Populations – 10-Second Data Resolution Example

| **HC Patient** | **ARHD of rSO_2_** | **ARHD of COx-a** |  | **SP Patient** | **ARHD of rSO_2_** | **ARHD of COx-a** |  | **TBI-GLR Patient** | **ARHD of rSO_2_** | **ARHD of COx** | **ARHD of COx-a** |
| --- | --- | --- | --- | --- | --- | --- | --- | --- | --- | --- | --- |
| 1 | 2 | 1 |  | 1 | 1 | 2 |  | 1 | 1 | 3 | 0 |
| 2 | 1 | 5 |  | 2 | 1 | 2 |  | 2 | 4 | 0 | 3 |
| 3 | 1 | 1 |  | 3 | 0 | 3 |  | 3 | 7 | 2 | 0 |
| 4 | 1 | 1 |  | 4 | 8 | 6 |  | 4 | 2 | 4 | 3 |
| 5 | 2 | 2 |  | 5 | 6 | 1 |  | 5 | 7 | 5 | 1 |
| 6 | 1 | 4 |  | 6 | 1 | 0 |  | 6 | 3 | 3 | 2 |
| 7 | 2 | 5 |  | 7 | 3 | 1 |  | 7 | 0 | 3 | 2 |
| 8 | 0 | 2 |  | 8 | 6 | 4 |  | 8 | 2 | 3 | 2 |
| 9 | 0 | 1 |  | 9 | 5 | 0 |  | 9 | 5 | 0 | 2 |
| 10 | 5 | 0 |  | 10 | 0 | 2 |  | 10 | 4 | 1 | 3 |
| 11 | 2 | 3 |  | 11 | 9 | 2 |  | 11 | 0 | 1 | 1 |
| 12 | 2 | 0 |  | 12 | 7 | 1 |  | 12 | 1 | 2 | 0 |
| 13 | 1 | 5 |  | 13 | 1 | 5 |  | 13 | 3 | 2 | 3 |
| 14 | 1 | 2 |  | 14 | 5 | 1 |  | 14 | 0 | 4 | 2 |
| 15 | 0 | 0 |  | 15 | 3 | 1 |  | 15 | 0 | 1 | 2 |
| 16 | 2 | 1 |  | 16 | 8 | 2 |  | 16 | 4 | 2 | 0 |
| 17 | 1 | 3 |  | 17 | 0 | 5 |  | 17 | 2 | 1 | 4 |
| 18 | 3 | 1 |  | 18 | 4 | 3 |  | 18 | 1 | 2 | 3 |
| 19 | 0 | 2 |  | 19 | 2 | 5 |  | 19 | 2 | 2 | 2 |
| 20 | 1 | 3 |  | 20 | 0 | 1 |  | 20 | 0 | 3 | 0 |
| 21 | 1 | 4 |  | 21 | 3 | 0 |  | 21 | 3 | 2 | 3 |
| 22 | 1 | 0 |  | 22 | 4 | 1 |  | 22 | 3 | 6 | 1 |
| 23 | 1 | 1 |  | 23 | 1 | 2 |  | 23 | 1 | 2 | 1 |
| 24 | 1 | 3 |  | 24 | 4 | 3 |  | 24 | 1 | 1 | 0 |
| 25 | 1 | 3 |  | 25 | 2 | 0 |  | 25 | 3 | 2 | 2 |
| 26 | 2 | 2 |  | 26 | 2 | 2 |  | 26 | 2 | 0 | 4 |
| 27 | 1 | 7 |  | 27 | 5 | 4 |  | 27 | 4 | 2 | 0 |
| 28 | 9 | 1 |  |  |  |  |  | 28 | 3 | 0 | 0 |
| 29 | 2 | 3 |  |  |  |  |  | 29 | 1 | 0 | 3 |
| 30 | 0 | 4 |  |  |  |  |  | 30 | 1 | 1 | 3 |
| 31 | 0 | 1 |  |  |  |  |  | 31 | 1 | 0 | 1 |
| 32 | 5 | 1 |  |  |  |  |  | 32 | 5 | 1 | 2 |
| 33 | 0 | 3 |  |  |  |  |  | 33 | 0 | 1 | 4 |
| 34 | 6 | 0 |  |  |  |  |  | 34 | 3 | 1 | 4 |
| 35 | 1 | 1 |  |  |  |  |  | 35 | 0 | 1 | 0 |
| 36 | 1 | 1 |  |  |  |  |  | 36 | 2 | 2 | 1 |
| 37 | 1 | 8 |  |  |  |  |  | 37 | 3 | 2 | 0 |
| 38 | 0 | 0 |  |  |  |  |  | 38 | 2 | 1 | 1 |
| 39 | 9 | 2 |  |  |  |  |  | 39 | 1 | 2 | 2 |
| 40 | 1 | 7 |  |  |  |  |  | 40 | 7 | 2 | 1 |
| 41 | 0 | 3 |  |  |  |  |  | 41 | 1 | 1 | 2 |
| 42 | 6 | 2 |  |  |  |  |  | 42 | 2 | 1 | 1 |
| 43 | 1 | 1 |  |  |  |  |  | 43 | 0 | 3 | 1 |
| 44 | 0 | 7 |  |  |  |  |  | 44 | 1 | 0 | 0 |
| 45 | 0 | 9 |  |  |  |  |  | 45 | 0 | 1 | 1 |
| 46 | 2 | 1 |  |  |  |  |  | 46 | 0 | 1 | 1 |
| 47 | 1 | 9 |  |  |  |  |  | 47 | 3 | 2 | 2 |
| 48 | 2 | 0 |  |  |  |  |  | 48 | 0 | 3 | 2 |
| 49 | 0 | 1 |  |  |  |  |  | 49 | 7 | 0 | 0 |
| 50 | 4 | 1 |  |  |  |  |  | 50 | 0 | 3 | 2 |
| 51 | 4 | 2 |  |  |  |  |  | 51 | 0 | 1 | 0 |
| 52 | 2 | 1 |  |  |  |  |  | 52 | 0 | 0 | 1 |
| 53 | 2 | 1 |  |  |  |  |  | 53 | 8 | 0 | 3 |
| 54 | 1 | 2 |  |  |  |  |  | 54 | 2 | 1 | 3 |
| 55 | 0 | 2 |  |  |  |  |  | 55 | 2 | 3 | 2 |
| 56 | 0 | 1 |  |  |  |  |  | 56 | 0 | 2 | 2 |
| 57 | 4 | 2 |  |  |  |  |  | 57 | 0 | 6 | 2 |
| 58 | 1 | 4 |  |  |  |  |  | 58 | 1 | 2 | 5 |
| 59 | 1 | 3 |  |  |  |  |  | 59 | 0 | 1 | 1 |
| 60 | 1 | 2 |  |  |  |  |  | 60 | 0 | 3 | 1 |
| 61 | 4 | 1 |  |  |  |  |  | 61 | 1 | 1 | 1 |
| 62 | 1 | 1 |  |  |  |  |  | 62 | 2 | 1 | 3 |
| 63 | 1 | 0 |  |  |  |  |  | 63 | 1 | 3 | 1 |
| 64 | 1 | 1 |  |  |  |  |  | 64 | 3 | 1 | 0 |
| 65 | 7 | 1 |  |  |  |  |  |  |  |  |  |
| 66 | 3 | 0 |  |  |  |  |  |  |  |  |  |
| 67 | 3 | 8 |  |  |  |  |  |  |  |  |  |
| 68 | 3 | 2 |  |  |  |  |  |  |  |  |  |
| 69 | 2 | 2 |  |  |  |  |  |  |  |  |  |
| 70 | 0 | 3 |  |  |  |  |  |  |  |  |  |
| 71 | 2 | 3 |  |  |  |  |  |  |  |  |  |
| 72 | 3 | 4 |  |  |  |  |  |  |  |  |  |
| 73 | 2 | 2 |  |  |  |  |  |  |  |  |  |
| 74 | 5 | 3 |  |  |  |  |  |  |  |  |  |
| 75 | 4 | 1 |  |  |  |  |  |  |  |  |  |
| 76 | 1 | 5 |  |  |  |  |  |  |  |  |  |
| 77 | 1 | 6 |  |  |  |  |  |  |  |  |  |
| 78 | 6 | 5 |  |  |  |  |  |  |  |  |  |
| 79 | 4 | 5 |  |  |  |  |  |  |  |  |  |
| 80 | 6 | 2 |  |  |  |  |  |  |  |  |  |
| 81 | 4 | 2 |  |  |  |  |  |  |  |  |  |
| 82 | 0 | 2 |  |  |  |  |  |  |  |  |  |
| 83 | 7 | 0 |  |  |  |  |  |  |  |  |  |
| 84 | 1 | 3 |  |  |  |  |  |  |  |  |  |
| 85 | 0 | 1 |  |  |  |  |  |  |  |  |  |
| 86 | 0 | 3 |  |  |  |  |  |  |  |  |  |
| 87 | 1 | 1 |  |  |  |  |  |  |  |  |  |
| 88 | 8 | 1 |  |  |  |  |  |  |  |  |  |
| 89 | 8 | 3 |  |  |  |  |  |  |  |  |  |
| 90 | 5 | 0 |  |  |  |  |  |  |  |  |  |
| 91 | 1 | 0 |  |  |  |  |  |  |  |  |  |
| 92 | 2 | 4 |  |  |  |  |  |  |  |  |  |
| 93 | 1 | 1 |  |  |  |  |  |  |  |  |  |
| 94 | 0 | 4 |  |  |  |  |  |  |  |  |  |
| 95 | 0 | 0 |  |  |  |  |  |  |  |  |  |
| 96 | 1 | 7 |  |  |  |  |  |  |  |  |  |
| 97 | 1 | 4 |  |  |  |  |  |  |  |  |  |
| 98 | 2 | 1 |  |  |  |  |  |  |  |  |  |
| 99 | 7 | 0 |  |  |  |  |  |  |  |  |  |
| 100 | 1 | 2 |  |  |  |  |  |  |  |  |  |
| 101 | 0 | 0 |  |  |  |  |  |  |  |  |  |
| 102 | 5 | 3 |  |  |  |  |  |  |  |  |  |
|  |  |  |  |  |  |  |  |  |  |  |  |
| *AIC, Akaike Information Criterion; ARHD, absolute regional hemispheric difference; ARIMA, autoregressive integrative moving average; COx, cerebral oximetry index with cerebral perfusion pressure; COx-a, cerebral oximetry index with arterial blood pressure; HC, healthy control volunteer group; rSO_2_, regional cerebral oxygen saturation; SP, elective spinal surgery patient group; TBI-GLR, traumatic brain injury patient group without bifrontal lobe pathology.* | | | | | | | | | | | |

File S5p: Regional Hemispheric Disparity of Personalized ARIMA P-Orders based on AIC in 10-Second Data Resolution for TBI-GL, TBI-GR, and TBI-BLR Populations

| **TBI-GL Patient** | **ARHD of rSO_2_** | **ARHD of COx** | **ARHD of COx-a** |  | **TBI-GR Patient** | **ARHD of rSO_2_** | **ARHD of COx** | **ARHD of COx-a** |  | **TBI-BLR Patient** | **ARHD of rSO_2_** | **ARHD of COx** | **ARHD of COx-a** |
| --- | --- | --- | --- | --- | --- | --- | --- | --- | --- | --- | --- | --- | --- |
| 1 | 0 | 0 | 1 |  | 1 | 1 | 1 | 2 |  | 1 | 2 | 3 | 2 |
| 2 | 9 | 3 | 3 |  | 2 | 1 | 2 | 1 |  | 2 | 0 | 3 | 3 |
| 3 | 1 | 0 | 4 |  | 3 | 3 | 1 | 1 |  | 3 | 1 | 1 | 0 |
| 4 | 5 | 4 | 4 |  | 4 | 8 | 3 | 1 |  | 4 | 0 | 1 | 1 |
| 5 | 6 | 6 | 1 |  | 5 | 8 | 2 | 1 |  | 5 | 2 | 2 | 1 |
| 6 | 3 | 3 | 6 |  | 6 | 0 | 1 | 3 |  |  |  |  |  |
| 7 | 1 | 3 | 0 |  | 7 | 3 | 1 | 0 |  |  |  |  |  |
| 8 | 1 | 1 | 3 |  | 8 | 6 | 3 | 2 |  |  |  |  |  |
| 9 | 2 | 0 | 1 |  | 9 | 5 | 1 | 2 |  |  |  |  |  |
| 10 | 3 | 1 | 1 |  | 10 | 5 | 4 | 4 |  |  |  |  |  |
| 11 | 7 | 3 | 3 |  | 11 | 0 | 1 | 2 |  |  |  |  |  |
| 12 | 5 | 1 | 1 |  |  |  |  |  |  |  |  |  |  |
| 13 | 1 | 0 | 1 |  |  |  |  |  |  |  |  |  |  |
| 14 | 0 | 0 | 3 |  |  |  |  |  |  |  |  |  |  |
| 15 | 1 | 0 | 1 |  |  |  |  |  |  |  |  |  |  |
|  |  |  |  |  |  |  |  |  |  |  |  |  |  |
| *AIC, Akaike Information Criterion; ARHD, absolute regional hemispheric difference; ARIMA, autoregressive integrative moving average; COx, cerebral oximetry index with cerebral perfusion pressure; COx-a, cerebral oximetry index with arterial blood pressure; rSO_2_, regional cerebral oxygen saturation; TBI-BLR, traumatic brain injury patient group with bifrontal lobe pathology; TBI-GL, traumatic brain injury patient group without left frontal lobe pathology; TBI-GR, traumatic brain injury patient group without right frontal lobe pathology.* | | | | | | | | | | | | | |

File S5q: Regional Hemispheric Disparity of Personalized ARIMA Q-Orders based on AIC for HC, SP, and TBI-GLR Populations – 10-Second Data Resolution Example

| **HC Patient** | **ARHD of rSO_2_** | **ARHD of COx-a** |  | **SP Patient** | **ARHD of rSO_2_** | **ARHD of COx-a** |  | **TBI-GLR Patient** | **ARHD of rSO_2_** | **ARHD of COx** | **ARHD of COx-a** |
| --- | --- | --- | --- | --- | --- | --- | --- | --- | --- | --- | --- |
| 1 | 2 | 0 |  | 1 | 3 | 6 |  | 1 | 6 | 2 | 0 |
| 2 | 1 | 6 |  | 2 | 2 | 2 |  | 2 | 6 | 3 | 6 |
| 3 | 0 | 4 |  | 3 | 0 | 3 |  | 3 | 3 | 1 | 2 |
| 4 | 1 | 0 |  | 4 | 3 | 7 |  | 4 | 3 | 3 | 4 |
| 5 | 1 | 0 |  | 5 | 2 | 0 |  | 5 | 0 | 1 | 4 |
| 6 | 1 | 2 |  | 6 | 6 | 0 |  | 6 | 0 | 3 | 4 |
| 7 | 3 | 1 |  | 7 | 1 | 2 |  | 7 | 0 | 3 | 1 |
| 8 | 3 | 2 |  | 8 | 7 | 2 |  | 8 | 2 | 0 | 4 |
| 9 | 0 | 1 |  | 9 | 7 | 7 |  | 9 | 2 | 0 | 4 |
| 10 | 6 | 1 |  | 10 | 2 | 8 |  | 10 | 5 | 4 | 5 |
| 11 | 0 | 3 |  | 11 | 9 | 1 |  | 11 | 0 | 0 | 3 |
| 12 | 0 | 0 |  | 12 | 8 | 2 |  | 12 | 0 | 0 | 2 |
| 13 | 1 | 4 |  | 13 | 3 | 4 |  | 13 | 0 | 3 | 0 |
| 14 | 4 | 8 |  | 14 | 6 | 6 |  | 14 | 1 | 4 | 3 |
| 15 | 6 | 0 |  | 15 | 3 | 3 |  | 15 | 0 | 2 | 0 |
| 16 | 3 | 0 |  | 16 | 10 | 9 |  | 16 | 0 | 3 | 2 |
| 17 | 1 | 3 |  | 17 | 0 | 0 |  | 17 | 8 | 4 | 5 |
| 18 | 4 | 8 |  | 18 | 7 | 8 |  | 18 | 3 | 0 | 4 |
| 19 | 0 | 3 |  | 19 | 2 | 0 |  | 19 | 3 | 1 | 0 |
| 20 | 1 | 4 |  | 20 | 6 | 1 |  | 20 | 0 | 2 | 1 |
| 21 | 4 | 3 |  | 21 | 0 | 6 |  | 21 | 2 | 8 | 7 |
| 22 | 1 | 0 |  | 22 | 4 | 5 |  | 22 | 3 | 0 | 0 |
| 23 | 1 | 2 |  | 23 | 1 | 4 |  | 23 | 2 | 3 | 0 |
| 24 | 2 | 4 |  | 24 | 2 | 3 |  | 24 | 1 | 1 | 1 |
| 25 | 7 | 3 |  | 25 | 3 | 2 |  | 25 | 6 | 2 | 1 |
| 26 | 3 | 1 |  | 26 | 6 | 1 |  | 26 | 0 | 4 | 8 |
| 27 | 2 | 7 |  | 27 | 2 | 1 |  | 27 | 2 | 1 | 7 |
| 28 | 0 | 4 |  |  |  |  |  | 28 | 0 | 0 | 1 |
| 29 | 2 | 2 |  |  |  |  |  | 29 | 7 | 0 | 0 |
| 30 | 1 | 10 |  |  |  |  |  | 30 | 5 | 1 | 5 |
| 31 | 1 | 2 |  |  |  |  |  | 31 | 10 | 1 | 5 |
| 32 | 1 | 0 |  |  |  |  |  | 32 | 1 | 1 | 2 |
| 33 | 1 | 0 |  |  |  |  |  | 33 | 1 | 0 | 0 |
| 34 | 9 | 7 |  |  |  |  |  | 34 | 3 | 1 | 5 |
| 35 | 1 | 1 |  |  |  |  |  | 35 | 1 | 1 | 1 |
| 36 | 1 | 1 |  |  |  |  |  | 36 | 3 | 0 | 0 |
| 37 | 1 | 3 |  |  |  |  |  | 37 | 0 | 1 | 2 |
| 38 | 1 | 2 |  |  |  |  |  | 38 | 1 | 0 | 2 |
| 39 | 9 | 2 |  |  |  |  |  | 39 | 0 | 0 | 2 |
| 40 | 0 | 10 |  |  |  |  |  | 40 | 3 | 4 | 1 |
| 41 | 1 | 1 |  |  |  |  |  | 41 | 1 | 0 | 2 |
| 42 | 4 | 1 |  |  |  |  |  | 42 | 4 | 1 | 3 |
| 43 | 3 | 0 |  |  |  |  |  | 43 | 3 | 4 | 1 |
| 44 | 0 | 8 |  |  |  |  |  | 44 | 0 | 0 | 0 |
| 45 | 3 | 9 |  |  |  |  |  | 45 | 1 | 1 | 1 |
| 46 | 2 | 6 |  |  |  |  |  | 46 | 2 | 1 | 0 |
| 47 | 1 | 2 |  |  |  |  |  | 47 | 0 | 1 | 1 |
| 48 | 3 | 0 |  |  |  |  |  | 48 | 0 | 1 | 1 |
| 49 | 2 | 5 |  |  |  |  |  | 49 | 1 | 0 | 4 |
| 50 | 3 | 0 |  |  |  |  |  | 50 | 1 | 6 | 1 |
| 51 | 0 | 6 |  |  |  |  |  | 51 | 1 | 0 | 0 |
| 52 | 5 | 2 |  |  |  |  |  | 52 | 1 | 0 | 0 |
| 53 | 2 | 4 |  |  |  |  |  | 53 | 0 | 1 | 1 |
| 54 | 3 | 10 |  |  |  |  |  | 54 | 1 | 6 | 0 |
| 55 | 1 | 1 |  |  |  |  |  | 55 | 4 | 4 | 7 |
| 56 | 2 | 3 |  |  |  |  |  | 56 | 6 | 2 | 2 |
| 57 | 2 | 5 |  |  |  |  |  | 57 | 2 | 9 | 4 |
| 58 | 3 | 4 |  |  |  |  |  | 58 | 1 | 1 | 0 |
| 59 | 0 | 6 |  |  |  |  |  | 59 | 0 | 2 | 2 |
| 60 | 1 | 2 |  |  |  |  |  | 60 | 3 | 0 | 2 |
| 61 | 5 | 3 |  |  |  |  |  | 61 | 1 | 0 | 1 |
| 62 | 2 | 3 |  |  |  |  |  | 62 | 0 | 2 | 0 |
| 63 | 1 | 2 |  |  |  |  |  | 63 | 0 | 6 | 3 |
| 64 | 2 | 1 |  |  |  |  |  | 64 | 0 | 0 | 0 |
| 65 | 5 | 3 |  |  |  |  |  |  |  |  |  |
| 66 | 3 | 8 |  |  |  |  |  |  |  |  |  |
| 67 | 1 | 0 |  |  |  |  |  |  |  |  |  |
| 68 | 2 | 1 |  |  |  |  |  |  |  |  |  |
| 69 | 1 | 0 |  |  |  |  |  |  |  |  |  |
| 70 | 4 | 4 |  |  |  |  |  |  |  |  |  |
| 71 | 4 | 5 |  |  |  |  |  |  |  |  |  |
| 72 | 9 | 1 |  |  |  |  |  |  |  |  |  |
| 73 | 2 | 3 |  |  |  |  |  |  |  |  |  |
| 74 | 8 | 0 |  |  |  |  |  |  |  |  |  |
| 75 | 5 | 2 |  |  |  |  |  |  |  |  |  |
| 76 | 1 | 4 |  |  |  |  |  |  |  |  |  |
| 77 | 0 | 0 |  |  |  |  |  |  |  |  |  |
| 78 | 4 | 3 |  |  |  |  |  |  |  |  |  |
| 79 | 2 | 5 |  |  |  |  |  |  |  |  |  |
| 80 | 6 | 2 |  |  |  |  |  |  |  |  |  |
| 81 | 5 | 0 |  |  |  |  |  |  |  |  |  |
| 82 | 0 | 0 |  |  |  |  |  |  |  |  |  |
| 83 | 2 | 0 |  |  |  |  |  |  |  |  |  |
| 84 | 1 | 5 |  |  |  |  |  |  |  |  |  |
| 85 | 2 | 2 |  |  |  |  |  |  |  |  |  |
| 86 | 2 | 0 |  |  |  |  |  |  |  |  |  |
| 87 | 2 | 1 |  |  |  |  |  |  |  |  |  |
| 88 | 0 | 0 |  |  |  |  |  |  |  |  |  |
| 89 | 1 | 1 |  |  |  |  |  |  |  |  |  |
| 90 | 5 | 3 |  |  |  |  |  |  |  |  |  |
| 91 | 3 | 4 |  |  |  |  |  |  |  |  |  |
| 92 | 3 | 1 |  |  |  |  |  |  |  |  |  |
| 93 | 0 | 2 |  |  |  |  |  |  |  |  |  |
| 94 | 0 | 1 |  |  |  |  |  |  |  |  |  |
| 95 | 2 | 8 |  |  |  |  |  |  |  |  |  |
| 96 | 2 | 3 |  |  |  |  |  |  |  |  |  |
| 97 | 4 | 6 |  |  |  |  |  |  |  |  |  |
| 98 | 7 | 4 |  |  |  |  |  |  |  |  |  |
| 99 | 2 | 0 |  |  |  |  |  |  |  |  |  |
| 100 | 3 | 2 |  |  |  |  |  |  |  |  |  |
| 101 | 0 | 3 |  |  |  |  |  |  |  |  |  |
| 102 | 0 | 2 |  |  |  |  |  |  |  |  |  |
|  |  |  |  |  |  |  |  |  |  |  |  |
| *AIC, Akaike Information Criterion; ARHD, absolute regional hemispheric difference; ARIMA, autoregressive integrative moving average; COx, cerebral oximetry index with cerebral perfusion pressure; COx-a, cerebral oximetry index with arterial blood pressure; HC, healthy control volunteer group; rSO_2_, regional cerebral oxygen saturation; SP, elective spinal surgery patient group; TBI-GLR, traumatic brain injury patient group without bifrontal lobe pathology.* | | | | | | | | | | | |

File S5r: Regional Hemispheric Disparity of Personalized ARIMA Q-Orders based on AIC for TBI-GL, TBI-GR, and TBI-BLR Populations – 10-Second Data Resolution Example

| **TBI-GL Patient** | **ARHD of rSO_2_** | **ARHD of COx** | **ARHD of COx-a** |  | **TBI-GR Patient** | **ARHD of rSO_2_** | **ARHD of COx** | **ARHD of COx-a** |  | **TBI-BLR Patient** | **ARHD of rSO_2_** | **ARHD of COx** | **ARHD of COx-a** |
| --- | --- | --- | --- | --- | --- | --- | --- | --- | --- | --- | --- | --- | --- |
| 1 | 1 | 1 | 6 |  | 1 | 9 | 1 | 3 |  | 1 | 4 | 3 | 0 |
| 2 | 6 | 0 | 0 |  | 2 | 0 | 0 | 0 |  | 2 | 0 | 0 | 0 |
| 3 | 8 | 2 | 0 |  | 3 | 5 | 3 | 0 |  | 3 | 0 | 2 | 2 |
| 4 | 4 | 3 | 6 |  | 4 | 1 | 0 | 1 |  | 4 | 0 | 1 | 1 |
| 5 | 2 | 0 | 5 |  | 5 | 2 | 2 | 2 |  | 5 | 1 | 4 | 0 |
| 6 | 0 | 0 | 7 |  | 6 | 9 | 2 | 1 |  |  |  |  |  |
| 7 | 0 | 0 | 4 |  | 7 | 4 | 0 | 2 |  |  |  |  |  |
| 8 | 8 | 2 | 2 |  | 8 | 1 | 5 | 4 |  |  |  |  |  |
| 9 | 1 | 0 | 1 |  | 9 | 7 | 3 | 3 |  |  |  |  |  |
| 10 | 0 | 0 | 1 |  | 10 | 0 | 5 | 4 |  |  |  |  |  |
| 11 | 2 | 5 | 6 |  | 11 | 0 | 2 | 3 |  |  |  |  |  |
| 12 | 2 | 4 | 1 |  |  |  |  |  |  |  |  |  |  |
| 13 | 2 | 2 | 1 |  |  |  |  |  |  |  |  |  |  |
| 14 | 3 | 1 | 0 |  |  |  |  |  |  |  |  |  |  |
| 15 | 1 | 0 | 0 |  |  |  |  |  |  |  |  |  |  |
|  |  |  |  |  |  |  |  |  |  |  |  |  |  |
| *AIC, Akaike Information Criterion; ARHD, absolute regional hemispheric difference; ARIMA, autoregressive integrative moving average; COx, cerebral oximetry index with cerebral perfusion pressure; COx-a, cerebral oximetry index with arterial blood pressure; rSO_2_, regional cerebral oxygen saturation; TBI-BLR, traumatic brain injury patient group with bifrontal lobe pathology; TBI-GL, traumatic brain injury patient group without left frontal lobe pathology; TBI-GR, traumatic brain injury patient group without right frontal lobe pathology.* | | | | | | | | | | | | | |

File S5s: Personalized ARIMA P-Orders and Q-Orders based on AIC and their Hemispheric Disparity in 1-Minute and 5-Minute Data Resolutions for HC, SP, and TBI-GLR Populations

| **Population** | **Personalized ARIMA *P-Orders* [Median (IQR)]** | | | | | | **ARHD of Personalized ARIMA *P-Orders* [Median (IQR)]** | | |
| --- | --- | --- | --- | --- | --- | --- | --- | --- | --- |
|  | **rSO_2__L** | **rSO_2__R** | **COx_L** | **COx_R** | **COx-a_L** | **COx-a_R** | **ARHD of rSO_2_** | **ARHD of COx** | **ARHD of COx-a** |
| **1-Minute Data Resolution** | | | | | | | | | |
| HC | 1 (1 – 2) | 1 (1 – 2) | – | – | 2 (1 – 2) | 2 (1 – 3) | 0 (0 – 1) | – | 1 (0 – 2.75) |
| SP | 3 (1 – 5) | 2 (2 – 4.5) | – | – | 3 (2 – 4) | 2 (2 – 3) | 2 (0 – 3.5) | – | 1 (0.5 – 2) |
| TBI-GLR | 6 (2.75 – 9) | 7 (3 – 9) | 3 (1 – 5) | 3 (1 – 6) | 3 (1 – 5) | 3 (1 – 7) | 3 (1 – 6) | 3 (1 – 5) | 2 (1 – 5) |
| **5-Minute Data Resolution** | | | | | | | | | |
| HC | 4 (3 – 4) | 4 (3 – 4) | – | – | 4 (3 – 4) | 4 (3 – 4) | 0 (0 – 1) | – | 0 (0 – 1) |
| SP | 1 (1 – 2.5) | 1 (1 – 3.5) | – | – | 1 (1 – 2) | 2 (2 – 4) | 1 (0 – 3) | – | 1 (0.5 – 2.5) |
| TBI-GLR | 5 (2 – 7.25) | 3.5 (2 – 7) | 2 (1 – 4) | 2 (1 – 4.25) | 2 (1 – 5) | 2 (1 – 4) | 2 (1 – 4) | 1 (0.75 – 3.25) | 1 (0.75 – 4) |
| **Population** | **Personalized ARIMA *Q-Orders* [Median (IQR)]** | | | | | | **ARHD of Personalized ARIMA *Q-Orders* [Median (IQR)]** | | |
|  | **rSO_2__L** | **rSO_2__R** | **COx_L** | **COx_R** | **COx-a_L** | **COx-a_R** | **ARHD of rSO_2_** | **ARHD of COx** | **ARHD of COx-a** |
| **1-Minute Data Resolution** | | | | | | | | | |
| HC | 1 (0 – 2) | 1 (0 – 2) | – | – | 1 (0 – 2) | 1 (0 – 2) | 1 (0 – 2) | – | 1 (0 – 2) |
| SP | 2 (0 – 5) | 2 (1.5 – 6.5) | – | – | 3 (2 – 4.5) | 4 (2 – 6) | 2 (1 – 4) | – | 2 (1 – 4) |
| TBI-GLR | 8 (4 – 10) | 7.5 (4 – 10) | 4.5 (1.75 – 7) | 5 (3 – 7) | 5 (1 – 7) | 4 (1 – 7) | 4 (1 – 5.25) | 3 (1 – 5) | 3.5 (1 – 5.25) |
| **5-Minute Data Resolution** | | | | | | | | | |
| HC | 2 (1 – 6) | 2 (0 – 4) | – | – | 1.5 (0 – 4) | 2 (1 – 5) | 2 (1 – 4) | – | 2 (1 – 5) |
| SP | 1 (0 – 1.5) | 0 (0 – 2) | – | – | 1 (1 – 1.5) | 1 (0 – 2) | 1 (0 – 2) | – | 1 (0.5 – 1) |
| TBI-GLR | 5 (3 – 9) | 5.5 (2 – 9) | 3 (1 – 5) | 2.5 (1 – 4) | 3 (1 – 5) | 3 (1 – 4.25) | 3 (1 – 5.25) | 2 (1 – 5.25) | 2 (0 – 4) |
| *AIC, Akaike Information Criterion; ARHD, Absolute Regional Hemispheric Disparity; au, arbitrary units; COx, cerebral oximetry index with cerebral perfusion pressure; COx-a, cerebral oximetry index with arterial blood pressure; HC, healthy control volunteer group; IQR, interquartile range; p-order, autoregressive order; q-order, moving average order; rSO_2_, regional cerebral oxygen saturation; SP, elective spinal surgery patient group; TBI-GLR, traumatic brain injury patient group without bifrontal lobe pathology.* | | | | | | | | | |

File S5t: Personalized ARIMA P-Orders and Q-Orders based on AIC and their Hemispheric Disparity in 10-Second, 1-Minute, and 5-Minute Data Resolutions for TBI-GL, TBI-GR, and TBI-BLR Populations

| **Population** | **Personalized ARIMA *P-Orders* [Median (IQR)]** | | | | | | **ARHD of Personalized ARIMA *P-Orders* [Median (IQR)]** | | |
| --- | --- | --- | --- | --- | --- | --- | --- | --- | --- |
|  | **rSO_2__L** | **rSO_2__R** | **COx_L** | **COx_R** | **COx-a_L** | **COx-a_R** | **ARHD of rSO_2_** | **ARHD of COx** | **ARHD of COx-a** |
| **10-Second Data Resolution** | | | | | | | | | |
| TBI-GL | 8 (4.5 – 9.5) | 9 (3 – 9) | 8 (7 – 9) | 9 (8 – 9) | 7 (7 – 8.5) | 9 (8.5 – 10) | 2 (1 – 5) | 1 (0 – 3) | 1 (1 – 3) |
| TBI-GR | 8 (5.5 – 9) | 6 (2 – 8.5) | 8 (7 – 8) | 8 (6.5 – 9) | 8 (6.5 – 9) | 8 (7 – 8) | 3 (1 – 5.5) | 1 (1 – 2.5) | 2 (1 – 2) |
| TBI-BLR | 10 (10 – 10) | 10 (9 – 10) | 9 (8 – 10) | 8 (8 – 9) | 7 (6 – 8) | 8 (8 – 10) | 1 (0 – 2) | 2 (1 – 3) | 1 (1 – 2) |
| **1-Minute Data Resolution** | | | | | | | | | |
| TBI-GL | 5 (2 – 6.5) | 4 (1.5 – 8) | 3 (2 – 5) | 5 (1 – 6) | 2 (1 – 5) | 3 (1 – 5) | 2 (1 – 5) | 2 (0 – 4) | 3 (1 – 4.5) |
| TBI-GR | 3 (3 – 8) | 5 (3.5 – 5.5) | 5 (2 – 6.5) | 6 (3.5 – 7.5) | 5 (3.5 – 7.5) | 6 (1.5 – 7) | 3 (2 – 4.5) | 2 (2 – 4) | 2 (1.5 – 4) |
| TBI-BLR | 7 (6 – 9) | 8 (3 – 9) | 6 (1 – 7) | 5 (3 – 5) | 6 (1 – 7) | 3 (3 – 5) | 1 (0 – 1) | 2 (2 – 3) | 3 (2 – 4) |
| **5-Minute Data Resolution** | | | | | | | | | |
| TBI-GL | 6 (2.5 – 8) | 5 (1 – 9) | 3 (1 – 4.5) | 2 (1 – 4.5) | 3 (1 – 4.5) | 2 (1 – 4) | 3 (1.5 – 4.5) | 3 (0 – 3.5) | 2 (1 – 3) |
| TBI-GR | 6 (1 – 7.5) | 2 (1.5 – 3) | 2 (1 – 3.5) | 3 (2 – 5) | 2 (2 – 4.5) | 2 (1.5 – 4) | 4 (0 – 5.5) | 3 (2 – 4) | 2 (0 – 3.5) |
| TBI-BLR | 7 (4 – 8) | 9 (1 – 9) | 3 (1 – 3) | 2 (2 – 5) | 2 (2 – 2) | 2 (1 – 3) | 3 (2 – 6) | 2 (1 – 2) | 1 (1 – 2) |
| **Population** | **Personalized ARIMA *Q-Orders* [Median (IQR)]** | | | | | | **ARHD of Personalized ARIMA *Q-Orders* [Median (IQR)]** | | |
|  | **rSO_2__L** | **rSO_2__R** | **COx_L** | **COx_R** | **COx-a_L** | **COx-a_R** | **ARHD of rSO_2_** | **ARHD of COx** | **ARHD of COx-a** |
| **10-Second Data Resolution** | | | | | | | | | |
| TBI-GL | 8 (6 – 10) | 10 (5.5 – 10) | 9 (7.5 – 10) | 10 (6.5 – 10) | 9 (7 – 10) | 10 (7 – 10) | 2 (1 – 3.5) | 1 (0 – 2) | 1 (0.5 – 5.5) |
| TBI-GR | 6 (2 – 10) | 10 (4.5 – 10) | 10 (7 – 10) | 9 (6 – 9.5) | 9 (7 – 10) | 8 (6 – 10) | 2 (0.5 – 6) | 2 (0.5 – 3) | 2 (1 – 3) |
| TBI-BLR | 10 (9 – 10) | 9 (9 – 10) | 8 (7 – 9) | 10 (10 – 10) | 8 (8 – 9) | 9 (9 – 10) | 0 (0 – 1) | 2 (1 – 3) | 0 (0 – 1) |
| **1-Minute Data Resolution** | | | | | | | | | |
| TBI-GL | 6 (4 – 10) | 6 (2 – 8.5) | 3 (1.5 – 6) | 4 (1.5 – 5) | 4 (1 – 6) | 6 (4 – 6.5) | 4 (0.5 – 5) | 2 (2 – 4) | 3 (0 – 7) |
| TBI-GR | 3 (1.5 – 7.5) | 5 (3.5 – 10) | 5 (4.5 – 8) | 5 (2 – 7) | 6 (3.5 – 7) | 4 (1.5 – 5) | 5 (2 – 5.5) | 3 (1 – 4) | 2 (2 – 4) |
| TBI-BLR | 10 (10 – 10) | 6 (5 – 9) | 5 (5 – 7) | 6 (5 – 10) | 5 (5 – 5) | 1 (1 – 1) | 5 (1 – 5) | 2 (1 – 4) | 4 (4 – 4) |
| **5-Minute Data Resolution** | | | | | | | | | |
| TBI-GL | 5 (3 – 10) | 5 (1.5 – 8) | 3 (1 – 4) | 3 (1 – 5) | 3 (1 – 3.5) | 2 (1 – 8.5) | 2 (1.5 – 5) | 1 (0 – 2.5) | 2 (1 – 5.5) |
| TBI-GR | 5 (1 – 7) | 3 (1.5 – 8.5) | 4 (2 – 5) | 4 (2 – 5.5) | 3 (1.5 – 5) | 3 (1 – 4.5) | 5 (1.5 – 6) | 1 (0 – 2.5) | 2 (0.5 – 5.5) |
| TBI-BLR | 8 (6 – 10) | 6 (1 – 8) | 3 (3 – 4) | 4 (3 – 5) | 2 (1 – 5) | 3 (1 – 4) | 3 (2 – 4) | 4 (0 – 4) | 4 (2 – 9) |
| *AIC, Akaike Information Criterion; ARHD, Absolute Regional Hemispheric Disparity; au, arbitrary units; COx, cerebral oximetry index with cerebral perfusion pressure; COx-a, cerebral oximetry index with arterial blood pressure; IQR, interquartile range;* *p-order, autoregressive order; q-order, moving average order; rSO_2_, regional cerebral oxygen saturation; TBI-BLR, traumatic brain injury patient group with bifrontal lobe pathology; TBI-GL, traumatic brain injury patient group without left frontal lobe pathology; TBI-GR, traumatic brain injury patient group without right frontal lobe pathology .* | | | | | | | | | |
