# Supplementary material for: Commercial NIRS May Not Detect Hemispheric Regional Disparity in Continuously Measured COx/COx-a: An Exploratory Healthy and Cranial Trauma Time-Series Analysis
Source: Bioengineering (Basel). 2025 Feb 28;12(3):247. doi: 10.3390/bioengineering12030247 (PMC11939202; doi:10.3390/bioengineering12030247)
Supplement: Supplementary file 1 [file bioengineering-12-00247-s001.zip › File S6.docx]

**File S6 – Impulse Response Function (IRF) Analysis**

File S6 – Table of Contents

[File S6a: Hemispheric Responsiveness using Impulse Response Coefficients of VAR model using Personalized and Capped VAR P-Order in 1-Minute and 5-Minute Data Resolutions for HC, SP, and TBI-GLR Populations 2](#_Toc191507886)

[File S6b: Hemispheric Responsiveness using Impulse Response Coefficients of VAR model using Personalized and Capped VAR P-Order in 10-Second, 1-Minute, and 5-Minute Data Resolutions for TBI-GL, TBI-GR, and TBI-BLR Populations 3](#_Toc191507887)

File S6a: Hemispheric Responsiveness using Impulse Response Coefficients of VAR model using Personalized and Capped VAR P-Order in 1-Minute and 5-Minute Data Resolutions for HC, SP, and TBI-GLR Populations

| **Direction** | **HC (n=102)** | | | | **SP (n=27)** | | | | **TBI-GLR (n=64)** | | | |  |
| --- | --- | --- | --- | --- | --- | --- | --- | --- | --- | --- | --- | --- | --- |
|  |  |  |  |  |  |  |  |  |  |  |  |  |  |
|  | **1-Minute** | | **5-Minute** | | **1-Minute** | | **5-Minute** | | **1-Minute** | | **5-Minute** | |  |
|  | **>0.1%**  **[% (count)]** | **NA**  **[% (count)]** | **>0.1%**  **[% (count)]** | **NA**  **[% (count)]** | **>0.1%**  **[% (count)]** | **NA**  **[% (count)]** | **>0.1%**  **[% (count)]** | **NA**  **[% (count)]** | **>0.1%**  **[% (count)]** | **NA**  **[% (count)]** | **>0.1%**  **[% (count)]** | **NA**  **[% (count)]** |  |
| **Personalized VAR P-Order** | | | | | | | | | | | | |  |
| ABP ® rSO_2__L | **34.3% (35)** | 13.7% (14) | 0% (0) | 100% (102) | 74.1% (20) | 7.4% (2) | 44.4% (12) | 11.1% (3) | 82.8% (53) | 0% (0) | 79.7% (51) | 0% (0) |  |
| ABP ® rSO_2__R | **41.2% (42)** | 13.7% (14) | 0% (0) | 100% (102) | 66.7% (18) | 14.8% (4) | 48.1% (13) | 14.8% (4) | 84.4% (54) | 0% (0) | 82.8% (53) | 0% (0) |  |
| rSO_2__L ® ABP | 33.3% (34) | 13.7% (14) | 0% (0) | 100% (102) | 66.7% (18) | 7.4% (2) | 44.4% (12) | 11.1% (3) | 81.3% (52) | 0% (0) | 79.7% (51) | 0% (0) |  |
| rSO_2__R ® ABP | 40.2% (41) | 13.7% (14) | 0% (0) | 100% (102) | 59.3% (16) | 14.8% (4) | 44.4% (12) | 14.8% (4) | 84.4% (54) | 0% (0) | 81.3% (52) | 0% (0) |  |
| CPP ® rSO_2__L | – | – | – | – | – | – | – | – | 82.8% (53) | 1.6% (1) | 75% (48) | 3.1% (2) |  |
| CPP ® rSO_2__R | – | – | – | – | – | – | – | – | 76.6% (49) | 1.6% (1) | 79.7% (51) | 3.1% (2) |  |
| rSO_2__L ® CPP | – | – | – | – | – | – | – | – | 81.3% (52) | 1.6% (1) | 75% (48) | 3.1% (2) |  |
| rSO_2__R ® CPP | – | – | – | – | – | – | – | – | 76.6% (49) | 1.6% (1) | 75% (48) | 3.1% (2) |  |
| **Capped VAR P-Order at 10** | | | | | | | | | | | | |  |
| ABP ® rSO_2__L | **34.3% (35)** | 13.7% (14) | 0% (0) | 100% (102) | 74.1% (20) | 7.4% (2) | 44.4% (12) | 11.1% (3) | 81.3% (52) | 0% (0) | 79.7% (51) | 0% (0) |  |
| ABP ® rSO_2__R | **41.2% (42)** | 13.7% (14) | 0% (0) | 100% (102) | 66.7% (18) | 14.8% (4) | 48.1% (13) | 14.8% (4) | 84.4% (54) | 0% (0) | 81.3% (52) | 0% (0) |  |
| rSO_2__L ® ABP | 33.3% (34) | 13.7% (14) | 0% (0) | 100% (102) | 66.7% (18) | 7.4% (2) | 44.4% (12) | 11.1% (3) | 81.3% (52) | 0% (0) | 78.1% (50) | 0% (0) |  |
| rSO_2__R ® ABP | 40.2% (41) | 13.7% (14) | 0% (0) | 100% (102) | 59.3% (16) | 14.8% (4) | 44.4% (12) | 14.8% (4) | 84.4% (54) | 0% (0) | 79.7% (51) | 0% (0) |  |
| CPP ® rSO_2__L | – | – | – | – | – | – | – | – | 81.3% (52) | 1.6% (1) | 75% (48) | 3.1% (2) |  |
| CPP ® rSO_2__R | – | – | – | – | – | – | – | – | 76.6% (49) | 1.6% (1) | 78.1% (50) | 3.1% (2) |  |
| rSO_2__L ® CPP | – | – | – | – | – | – | – | – | 79.7% (51) | 1.6% (1) | 73.4% (47) | 3.1% (2) |  |
| rSO_2__R ® CPP | – | – | – | – | – | – | – | – | 73.4% (47) | 1.6% (1) | 75% (48) | 3.1% (2) |  |
| *ABP, arterial blood pressure; CPP, cerebral perfusion pressure; HC, healthy control volunteer group;* *p-order, autoregressive order; rSO_2_, regional cerebral oxygen saturation; SP, elective spinal surgery patient group; TBI-GLR, traumatic brain injury patient group without bifrontal lobe pathology; VAR, vector autoregressive.* | | | | | | | | | | | | |  |
|  |  |  |  |  |  |  |  |  |  |  |  |  |  |

File S6b: Hemispheric Responsiveness using Impulse Response Coefficients of VAR model using Personalized and Capped VAR P-Order in 10-Second, 1-Minute, and 5-Minute Data Resolutions for TBI-GL, TBI-GR, and TBI-BLR Populations

| **Direction** | **TBI-GL (n=15)** | | | | | | **TBI-GR (n=11)** | | | | | | **TBI-BLR (n=5)** | | | | | |  |
| --- | --- | --- | --- | --- | --- | --- | --- | --- | --- | --- | --- | --- | --- | --- | --- | --- | --- | --- | --- |
|  |  |  |  |  |  |  |  |  |  |  |  |  |  |  |  |  |  |  |  |
|  | **10-Second** | | **1-Minute** | | **5-Minute** | | **10-Second** | | **1-Minute** | | **5-Minute** | | **10-Second** | | **1-Minute** | | **5-Minute** | |  |
|  | **>0.1%**  **[% (count)]** | **NA**  **[% (count)]** | **>0.1%**  **[% (count)]** | **NA**  **[% (count)]** | **>0.1%**  **[% (count)]** | **NA**  **[% (count)]** | **>0.1%**  **[% (count)]** | **NA**  **[% (count)]** | **>0.1%**  **[% (count)]** | **NA**  **[% (count)]** | **>0.1%**  **[% (count)]** | **NA**  **[% (count)]** | **>0.1%**  **[% (count)]** | **NA**  **[% (count)]** | **>0.1%**  **[% (count)]** | **NA**  **[% (count)]** | **>0.1%**  **[% (count)]** | **NA**  **[% (count)]** |  |
| **Personalized VAR P-Order** | | | | | | | | | | | | | | | | | | |  |
| ABP ® rSO_2__L | **86.7% (13)** | 0% (0) | 80% (12) | 0% (0) | **93.3% (14)** | 0% (0) | 90.9% (10) | 0% (0) | **72.7% (8)** | 0% (0) | **63.6% (7)** | 0% (0) | 100% (5) | 0% (0) | 80% (4) | 0% (0) | **100% (5)** | 0% (0) |  |
| ABP ® rSO_2__R | **73.3% (11)** | 0% (0) | 73.3% (11) | 0% (0) | **66.7% (10)** | 0% (0) | 90.9% (10) | 0% (0) | **54.5% (6)** | 0% (0) | **27.3% (3)** | 0% (0) | 100% (5) | 0% (0) | 80% (4) | 0% (0) | **60% (3)** | 0% (0) |  |
| rSO_2__L ® ABP | **80% (12)** | 0% (0) | 73.3% (11) | 0% (0) | **93.3% (14)** | 0% (0) | 100% (11) | 0% (0) | **72.7% (8)** | 0% (0) | **63.6% (7)** | 0% (0) | **100% (5)** | 0% (0) | 80% (4) | 0% (0) | **100% (5)** | 0% (0) |  |
| rSO_2__R ® ABP | **66.7% (10)** | 0% (0) | 73.3% (11) | 0% (0) | **66.7% (10)** | 0% (0) | 90.9% (10) | 0% (0) | **45.5% (5)** | 0% (0) | **27.3% (3)** | 0% (0) | **80% (4)** | 0% (0) | 80% (4) | 0% (0) | **60% (3)** | 0% (0) |  |
| CPP ® rSO_2__L | **100% (15)** | 0% (0) | 80% (12) | 0% (0) | 80% (12) | 0% (0) | 100% (11) | 0% (0) | **90.9% (10)** | 0% (0) | **72.7% (8)** | 0% (0) | **80% (4)** | 0% (0) | **100% (5)** | 0% (0) | 100% (5) | 0% (0) |  |
| CPP ® rSO_2__R | **86.7% (13)** | 0% (0) | 73.3% (11) | 0% (0) | 80% (12) | 0% (0) | 90.9% (10) | 0% (0) | **72.7% (8)** | 0% (0) | **54.5% (6)** | 0% (0) | **100% (5)** | 0% (0) | **80% (4)** | 0% (0) | 100% (5) | 0% (0) |  |
| rSO_2__L ® CPP | **100% (15)** | 0% (0) | 80% (12) | 0% (0) | 80% (12) | 0% (0) | **100% (11)** | 0% (0) | **81.8% (9)** | 0% (0) | **72.7% (8)** | 0% (0) | 100% (5) | 0% (0) | **100% (5)** | 0% (0) | 100% (5) | 0% (0) |  |
| rSO_2__R ® CPP | **80% (12)** | 0% (0) | 73.3% (11) | 0% (0) | 80% (12) | 0% (0) | **81.8% (9)** | 0% (0) | **63.6% (7)** | 0% (0) | **45.5% (5)** | 0% (0) | 100% (5) | 0% (0) | **80% (4)** | 0% (0) | 100% (5) | 0% (0) |  |
| **Capped VAR P-Order at 10** | | | | | | | | | | | | | | | | | | |  |
| ABP ® rSO_2__L | **40% (6)** | 0% (0) | **80% (12)** | 0% (0) | **93.3% (14)** | 0% (0) | **81.8% (9)** | 0% (0) | **72.7% (8)** | 0% (0) | **63.6% (7)** | 0% (0) | 80% (4) | 0% (0) | 80% (4) | 0% (0) | **100% (5)** | 0% (0) |  |
| ABP ® rSO_2__R | **60% (9)** | 0% (0) | **66.7% (10)** | 0% (0) | **66.7% (10)** | 0% (0) | **54.5% (6)** | 0% (0) | **45.5% (5)** | 0% (0) | **27.3% (3)** | 0% (0) | 80% (4) | 0% (0) | 80% (4) | 0% (0) | **60% (3)** | 0% (0) |  |
| rSO_2__L ® ABP | **60% (9)** | 0% (0) | 66.7% (10) | 0% (0) | **93.3% (14)** | 0% (0) | **90.9% (10)** | 0% (0) | **72.7% (8)** | 0% (0) | **63.6% (7)** | 0% (0) | 80% (4) | 0% (0) | 80% (4) | 0% (0) | **100% (5)** | 0% (0) |  |
| rSO_2__R ® ABP | **46.7% (7)** | 0% (0) | 66.7% (10) | 0% (0) | **66.7% (10)** | 0% (0) | **63.6% (7)** | 0% (0) | **45.5% (5)** | 0% (0) | **27.3% (3)** | 0% (0) | 80% (4) | 0% (0) | 80% (4) | 0% (0) | **60% (3)** | 0% (0) |  |
| CPP ® rSO_2__L | 66.7% (10) | 0% (0) | 80% (12) | 0% (0) | 80% (12) | 0% (0) | **90.9% (10)** | 0% (0) | **81.8% (9)** | 0% (0) | **72.7% (8)** | 0% (0) | **60% (3)** | 0% (0) | **100% (5)** | 0% (0) | 100% (5) | 0% (0) |  |
| CPP ® rSO_2__R | 73.3% (11) | 0% (0) | 73.3% (11) | 0% (0) | 80% (12) | 0% (0) | **63.6% (7)** | 0% (0) | **63.6% (7)** | 0% (0) | **54.5% (6)** | 0% (0) | **80% (4)** | 0% (0) | **80% (4)** | 0% (0) | 100% (5) | 0% (0) |  |
| rSO_2__L ® CPP | 60% (9) | 0% (0) | **80% (12)** | 0% (0) | 80% (12) | 0% (0) | **81.8% (9)** | 0% (0) | **81.8% (9)** | 0% (0) | **72.7% (8)** | 0% (0) | **60% (3)** | 0% (0) | **100% (5)** | 0% (0) | 100% (5) | 0% (0) |  |
| rSO_2__R ® CPP | 60% (9) | 0% (0) | **66.7% (10)** | 0% (0) | 80% (12) | 0% (0) | **63.6% (7)** | 0% (0) | **54.5% (6)** | 0% (0) | **45.5% (5)** | 0% (0) | **80% (4)** | 0% (0) | **80% (4)** | 0% (0) | 100% (5) | 0% (0) |  |
| *ABP, arterial blood pressure; CPP, cerebral perfusion pressure; p-order, autoregressive order; rSO_2_, regional cerebral oxygen saturation; TBI-BLR, traumatic brain injury patient group with bifrontal lobe pathology; TBI-GL, traumatic brain injury patient group without left frontal lobe pathology; TBI-GR, traumatic brain injury patient group without right frontal lobe pathology; VAR, vector autoregressive.* | | | | | | | | | | | | | | | | | | |  |
