# Supplementary material for: Commercial NIRS May Not Detect Hemispheric Regional Disparity in Continuously Measured COx/COx-a: An Exploratory Healthy and Cranial Trauma Time-Series Analysis
Source: Bioengineering (Basel). 2025 Feb 28;12(3):247. doi: 10.3390/bioengineering12030247 (PMC11939202; doi:10.3390/bioengineering12030247)
Supplement: Supplementary file 1 [file bioengineering-12-00247-s001.zip › File S7.docx]

**File S7 – Granger Causality Analysis**

File S7 – Table of Contents

[File S7a: Granger Causality Test Results for HC Population in 10-Second, 1-Minute, and 5-Minute Data Resolutions 2](#_Toc191508042)

[File S7b: Granger Causality Test Results for SP Population in 10-Second, 1-Minute, and 5-Minute Data Resolutions 6](#_Toc191508043)

[File S7c: Granger Causality Test Results for TBI-GLR Population in 10-Second, 1-Minute, and 5-Minute Data Resolutions 7](#_Toc191508044)

[File S7d: Granger Causality Test Results for TBI-GL Population in 10-Second, 1-Minute, and 5-Minute Data Resolutions 10](#_Toc191508045)

[File S7e: Granger Causality Test Results for TBI-GR Population in 10-Second, 1-Minute, and 5-Minute Data Resolutions 11](#_Toc191508046)

[File S7f: Granger Causality Test Results for TBI-BLR Population in 10-Second, 1-Minute, and 5-Minute Data Resolutions 12](#_Toc191508047)

[File S7g: Granger Causal Directionality Results based on greater F-Statistic in 1-Minute and 5-Minute Data Resolutions for HC, SP, and TBI-GLR Populations 13](#_Toc191508048)

[File S7h: Granger Causal Directionality Results based on greater F-Statistic in 10-Second, 1-Minute, and 5-Minute Data Resolutions for TBI-GL, TBI-GR, and TBI-BLR Populations 14](#_Toc191508049)

File S7a: Granger Causality Test Results for HC Population in 10-Second, 1-Minute, and 5-Minute Data Resolutions

| **Patient** | **ABP ® rSO_2__L (F Statistic)** | **ABP ® rSO_2__L (P-Value)** | **rSO_2__L ® ABP (F Statistic)** | **rSO_2__L ® ABP (P-Value)** | **ABP ® rSO_2__R (F Statistic)** | **ABP ® rSO_2__R (P-Value)** | **rSO_2__R ® ABP (F Statistic)** | **rSO_2__R ® ABP (P-Value)** |
| --- | --- | --- | --- | --- | --- | --- | --- | --- |
| **10-Second Data Resolution** | | | | | | | | |
| 1 | 8.7843 | 0.0034 | 5.9627 | 0.0155 | 4.6734 | 0.0036 | 1.1427 | 0.3330 |
| 2 | 2.8567 | 0.0386 | 0.4344 | 0.7286 | 7.4634 | 0.0000 | 1.6219 | 0.1708 |
| 3 | 3.6401 | 0.0069 | 3.7109 | 0.0061 | 0.5295 | 0.5897 | 0.3454 | 0.7083 |
| 4 | 2.8804 | 0.0101 | 1.5118 | 0.1754 | 3.3672 | 0.0194 | 1.0863 | 0.3557 |
| 5 | 0.4617 | 0.8815 | 0.7671 | 0.6322 | 1.8252 | 0.0489 | 1.2744 | 0.2394 |
| 6 | 1.5889 | 0.2083 | 3.0196 | 0.0524 | 0.9527 | 0.4363 | 1.8361 | 0.1264 |
| 7 | 1.3275 | 0.1384 | 1.3594 | 0.1196 | 1.8470 | 0.0245 | 0.8341 | 0.6580 |
| 8 | 3.4130 | 0.0353 | 2.5551 | 0.0807 | 2.9063 | 0.0575 | 0.3606 | 0.6978 |
| 9 | 6.0978 | 0.0000 | 1.4590 | 0.1953 | 1.9802 | 0.0712 | 0.4042 | 0.8755 |
| 10 | 0.7864 | 0.4579 | 2.6364 | 0.0759 | 1.6890 | 0.0832 | 0.6322 | 0.8092 |
| 11 | 2.4763 | 0.0087 | 2.2015 | 0.0201 | 7.2535 | 0.0000 | 2.4448 | 0.0271 |
| 12 | 1.1250 | 0.3328 | 1.2152 | 0.2526 | 0.8247 | 0.7418 | 0.6436 | 0.9325 |
| 13 | 1.2215 | 0.2707 | 1.6954 | 0.0704 | 0.3331 | 0.9713 | 0.4308 | 0.9303 |
| 14 | 1.1455 | 0.3324 | 0.0307 | 0.9927 | 0.2387 | 0.7879 | 0.3048 | 0.7377 |
| 15 | 3.5322 | 0.0047 | 0.1855 | 0.9678 | 4.3338 | 0.0010 | 1.2015 | 0.3109 |
| 16 | 7.0611 | 0.0012 | 1.4782 | 0.2318 | 1.3230 | 0.2521 | 0.2224 | 0.9689 |
| 17 | 0.8212 | 0.6284 | 0.1925 | 0.9986 | 1.0279 | 0.4198 | 0.9147 | 0.5139 |
| 18 | 6.9405 | 0.0090 | 2.8132 | 0.0948 | 0.5624 | 0.6902 | 2.1033 | 0.0813 |
| 19 | 1.4141 | 0.1429 | 2.3288 | 0.0044 | 1.4292 | 0.1354 | 0.8218 | 0.6593 |
| 20 | 1.3464 | 0.2549 | 0.5578 | 0.6936 | 1.1177 | 0.3542 | 0.8335 | 0.5744 |
| 21 | 2.3837 | 0.0951 | 1.5371 | 0.2178 | 9.6628 | 0.0022 | 1.5895 | 0.2090 |
| 22 | 3.4423 | 0.0099 | 0.9039 | 0.4631 | 4.4703 | 0.0128 | 0.4647 | 0.6291 |
| 23 | 1.5809 | 0.1677 | 2.1031 | 0.0671 | 2.4821 | 0.0086 | 0.4040 | 0.9433 |
| 24 | 1.5748 | 0.2124 | 0.0989 | 0.7539 | 1.2127 | 0.3018 | 0.2861 | 0.7518 |
| 25 | 1.7589 | 0.1565 | 1.1688 | 0.3229 | 2.6756 | 0.0714 | 1.6908 | 0.1871 |
| 26 | 0.7657 | 0.6334 | 1.4003 | 0.1984 | 2.6107 | 0.0366 | 0.7426 | 0.5640 |
| 27 | 0.3423 | 0.5591 | 0.5906 | 0.4431 | 0.0052 | 0.9948 | 1.1118 | 0.3310 |
| 28 | NA | NA | NA | NA | 5.3358 | 0.0000 | 0.5987 | 0.7562 |
| 29 | 0.9010 | 0.4647 | 0.9527 | 0.4350 | 1.6282 | 0.1993 | 0.0984 | 0.9063 |
| 30 | 2.8119 | 0.0119 | 0.7935 | 0.5760 | 1.9054 | 0.0815 | 0.6148 | 0.7183 |
| 31 | 0.9937 | 0.4307 | 1.1809 | 0.3179 | 0.2417 | 0.9621 | 0.4587 | 0.8382 |
| 32 | 3.0793 | 0.0485 | 0.5582 | 0.5733 | 1.5487 | 0.1135 | 1.5062 | 0.1282 |
| 33 | 0.0687 | 0.9336 | 0.1317 | 0.8767 | 2.0636 | 0.1296 | 0.6177 | 0.5402 |
| 34 | 1.5262 | 0.1561 | 0.9396 | 0.4871 | 0.6704 | 0.5133 | 0.2214 | 0.8017 |
| 35 | 1.5436 | 0.1912 | 1.9616 | 0.1020 | 3.3599 | 0.0367 | 0.6701 | 0.5128 |
| 36 | 0.9664 | 0.4492 | 0.3408 | 0.9146 | 1.9105 | 0.0358 | 0.7719 | 0.6788 |
| 37 | 1.1636 | 0.2822 | 0.0242 | 0.8764 | 3.0263 | 0.0511 | 0.3186 | 0.7276 |
| 38 | 2.4227 | 0.0496 | 1.3147 | 0.2659 | 2.5476 | 0.0407 | 1.3991 | 0.2357 |
| 39 | 3.8709 | 0.0507 | 0.9633 | 0.3277 | 1.0814 | 0.3801 | 0.8161 | 0.6136 |
| 40 | 1.3064 | 0.2050 | 0.5774 | 0.8887 | 1.1569 | 0.3019 | 1.2476 | 0.2265 |
| 41 | 1.7469 | 0.1405 | 2.2428 | 0.0652 | 0.8917 | 0.4696 | 1.2308 | 0.2985 |
| 42 | 0.0004 | 0.9838 | 3.1761 | 0.0761 | 1.6680 | 0.1184 | 0.7768 | 0.6074 |
| 43 | 0.1863 | 0.6669 | 0.9746 | 0.3258 | 0.8079 | 0.4487 | 3.2184 | 0.0442 |
| 44 | 0.8981 | 0.5190 | 1.1900 | 0.3065 | 0.7932 | 0.6092 | 0.9715 | 0.4594 |
| 45 | 0.5045 | 0.8045 | 0.7099 | 0.6420 | 0.6196 | 0.7145 | 0.5893 | 0.7387 |
| 46 | 6.2553 | 0.0025 | 1.7395 | 0.1796 | 1.4032 | 0.2188 | 0.4757 | 0.8253 |
| 47 | 0.6318 | 0.5327 | 0.3546 | 0.7019 | 1.2689 | 0.2862 | 1.8786 | 0.1345 |
| 48 | 0.6545 | 0.7307 | 1.9947 | 0.0505 | 0.4538 | 0.7695 | 1.3356 | 0.2589 |
| 49 | 2.3330 | 0.0579 | 0.6451 | 0.6311 | 1.1907 | 0.3168 | 0.2911 | 0.8835 |
| 50 | 0.4030 | 0.9438 | 0.1745 | 0.9978 | 0.7459 | 0.4757 | 1.1583 | 0.3162 |
| 51 | 0.7134 | 0.8558 | 0.6114 | 0.9392 | 0.7050 | 0.7462 | 1.9306 | 0.0311 |
| 52 | 0.4511 | 0.8121 | 0.7599 | 0.5798 | 0.1619 | 0.9219 | 1.2281 | 0.3007 |
| 53 | 1.0465 | 0.3857 | 0.5556 | 0.6953 | 2.6636 | 0.0181 | 0.8730 | 0.5168 |
| 54 | 2.1292 | 0.0807 | 1.7420 | 0.1445 | 2.4692 | 0.0884 | 2.2424 | 0.1101 |
| 55 | 1.3358 | 0.2520 | 0.3964 | 0.8507 | 1.3991 | 0.2276 | 1.0735 | 0.3773 |
| 56 | 0.0433 | 0.8353 | 2.7567 | 0.0984 | 0.4090 | 0.5232 | 2.2244 | 0.1374 |
| 57 | 1.1539 | 0.3203 | 0.8270 | 0.6225 | 1.3997 | 0.1307 | 0.7417 | 0.7777 |
| 58 | 2.2230 | 0.0139 | 0.5501 | 0.8779 | 0.6693 | 0.7513 | 0.6805 | 0.7411 |
| 59 | 0.5483 | 0.7394 | 1.1441 | 0.3409 | 0.2494 | 0.9096 | 1.2690 | 0.2857 |
| 60 | 1.2562 | 0.2874 | 1.5616 | 0.2128 | 0.3822 | 0.8211 | 0.1405 | 0.9669 |
| 61 | 1.4634 | 0.1348 | 0.8449 | 0.6196 | 1.5945 | 0.1526 | 1.3635 | 0.2331 |
| 62 | 2.8346 | 0.0264 | 1.0687 | 0.3740 | 3.0551 | 0.0033 | 0.6415 | 0.7418 |
| 63 | 1.4103 | 0.1980 | 0.6479 | 0.7362 | 1.3636 | 0.1931 | 0.8178 | 0.6318 |
| 64 | 1.6007 | 0.1773 | 0.0733 | 0.9901 | 1.5889 | 0.1338 | 0.9837 | 0.4516 |
| 65 | 2.8304 | 0.0055 | 0.9698 | 0.4610 | 0.0940 | 0.7594 | 0.9681 | 0.3263 |
| 66 | 1.3042 | 0.1904 | 1.7076 | 0.0404 | 1.1153 | 0.3518 | 0.7340 | 0.7532 |
| 67 | 1.1949 | 0.3032 | 0.7986 | 0.6044 | 2.4359 | 0.0897 | 0.9087 | 0.4045 |
| 68 | 3.9038 | 0.0047 | 0.7808 | 0.5392 | 3.6449 | 0.0579 | 0.9914 | 0.3208 |
| 69 | 2.0175 | 0.0941 | 0.1467 | 0.9643 | 0.6841 | 0.7648 | 1.4365 | 0.1553 |
| 70 | 5.4891 | 0.0003 | 2.1736 | 0.0728 | 2.1402 | 0.0767 | 0.9524 | 0.4345 |
| 71 | 0.6509 | 0.7680 | 1.1640 | 0.3198 | 0.7492 | 0.7673 | 1.3279 | 0.1756 |
| 72 | 1.3669 | 0.2155 | 1.2014 | 0.3019 | 0.1378 | 0.8714 | 0.0726 | 0.9300 |
| 73 | 1.4237 | 0.2083 | 0.3711 | 0.8965 | 0.4743 | 0.6231 | 0.0790 | 0.9240 |
| 74 | 1.1963 | 0.3144 | 0.1013 | 0.9819 | 0.9917 | 0.4835 | 0.7033 | 0.8383 |
| 75 | 1.6583 | 0.0617 | 1.7957 | 0.0371 | 1.1025 | 0.3640 | 0.6560 | 0.7295 |
| 76 | 3.0581 | 0.0032 | 1.5361 | 0.1492 | 1.6616 | 0.1614 | 0.1902 | 0.9432 |
| 77 | 0.4722 | 0.4929 | 1.3164 | 0.2529 | 0.8997 | 0.4087 | 1.0943 | 0.3372 |
| 78 | 0.8791 | 0.5943 | 0.7399 | 0.7482 | 2.2809 | 0.0632 | 0.3805 | 0.8223 |
| 79 | 5.4778 | 0.0004 | 1.4147 | 0.2318 | 1.2147 | 0.2579 | 1.0627 | 0.3996 |
| 80 | 7.2716 | 0.0077 | 1.5271 | 0.2182 | 3.3924 | 0.0021 | 1.1186 | 0.3540 |
| 81 | 4.2805 | 0.0001 | 1.8248 | 0.0772 | 5.2255 | 0.0006 | 1.7537 | 0.1411 |
| 82 | 4.8409 | 0.0089 | 16.3597 | 0.0000 | 4.7450 | 0.0098 | 1.8842 | 0.1548 |
| 83 | 1.6457 | 0.1071 | 1.5747 | 0.1276 | 0.1601 | 0.8522 | 0.6150 | 0.5418 |
| 84 | 3.0850 | 0.0479 | 0.7418 | 0.4776 | 0.3706 | 0.5434 | 0.7073 | 0.4013 |
| 85 | 3.8195 | 0.0054 | 1.5546 | 0.1888 | 1.4903 | 0.2074 | 0.8005 | 0.5264 |
| 86 | 13.6093 | 0.0003 | 0.1918 | 0.6620 | 0.7643 | 0.3834 | 1.6550 | 0.2002 |
| 87 | 11.5242 | 0.0000 | 2.9458 | 0.0548 | 29.5062 | 0.0000 | 7.2672 | 0.0076 |
| 88 | 0.8879 | 0.6256 | 0.9482 | 0.5458 | 1.8347 | 0.1432 | 1.3053 | 0.2748 |
| 89 | 0.8774 | 0.4790 | 0.3847 | 0.8193 | 0.8555 | 0.6887 | 0.6861 | 0.8868 |
| 90 | 0.9007 | 0.5481 | 1.5779 | 0.1049 | 0.9585 | 0.5400 | 1.8412 | 0.0155 |
| 91 | 3.1033 | 0.0066 | 0.4935 | 0.8126 | 2.4996 | 0.0444 | 0.8574 | 0.4908 |
| 92 | 2.4468 | 0.0062 | 1.7438 | 0.0632 | 4.6893 | 0.0013 | 2.1942 | 0.0717 |
| 93 | 0.1901 | 0.8271 | 0.0287 | 0.9718 | 0.0114 | 0.9152 | 2.3730 | 0.1254 |
| 94 | 1.3515 | 0.2597 | 0.7601 | 0.5181 | 0.8323 | 0.4780 | 1.4145 | 0.2406 |
| 95 | 0.2019 | 0.9370 | 0.1020 | 0.9816 | 0.3597 | 0.8370 | 0.6650 | 0.6172 |
| 96 | 14.7547 | 0.0000 | 0.2779 | 0.8921 | 7.8561 | 0.0000 | 0.7765 | 0.5892 |
| 97 | 1.0646 | 0.3932 | 1.6484 | 0.0827 | 1.1921 | 0.3123 | 0.5735 | 0.7511 |
| 98 | 1.2640 | 0.2851 | 0.4783 | 0.6207 | 0.7487 | 0.6113 | 0.8233 | 0.5534 |
| 99 | 4.8983 | 0.0086 | 0.6947 | 0.5007 | 1.0892 | 0.3728 | 0.7112 | 0.7781 |
| 100 | 3.5595 | 0.0307 | 0.5723 | 0.5653 | 2.0503 | 0.0900 | 0.3613 | 0.8358 |
| 101 | 4.8049 | 0.0093 | 1.7475 | 0.1771 | 5.9669 | 0.0031 | 0.9592 | 0.3851 |
| 102 | 1.7547 | 0.1001 | 2.2412 | 0.0336 | 4.0511 | 0.0191 | 0.8992 | 0.4088 |
| **1-Minute Data Resolution** | | | | | | | | |
| 1 | 1.4611 | 0.2512 | 0.3880 | 0.8147 | NA | NA | NA | NA |
| 2 | 0.0741 | 0.7876 | 0.6132 | 0.4407 | 0.9523 | 0.3381 | 0.2151 | 0.6466 |
| 3 | 2.7574 | 0.1069 | 0.0368 | 0.8490 | 0.6363 | 0.4311 | 0.5824 | 0.4511 |
| 4 | 1.8148 | 0.1574 | 0.8255 | 0.5943 | 1.4676 | 0.2456 | 0.1449 | 0.8657 |
| 5 | NA | NA | NA | NA | NA | NA | NA | NA |
| 6 | NA | NA | NA | NA | NA | NA | NA | NA |
| 7 | 11.4794 | 0.0001 | 3.6540 | 0.0239 | 15.7209 | 0.0000 | 0.8394 | 0.5181 |
| 8 | 2.7547 | 0.1100 | 2.7804 | 0.1084 | 3.1859 | 0.0869 | 0.6092 | 0.4427 |
| 9 | 1.7363 | 0.1911 | 0.6111 | 0.6932 | 0.4999 | 0.7715 | 0.5197 | 0.7575 |
| 10 | NA | NA | NA | NA | NA | NA | NA | NA |
| 11 | 2.3303 | 0.1385 | 0.1014 | 0.7526 | 10.0731 | 0.0007 | 1.4545 | 0.2534 |
| 12 | 2.1047 | 0.1438 | 0.2108 | 0.8114 | 1.3718 | 0.2728 | 0.5804 | 0.5673 |
| 13 | 0.6677 | 0.5203 | 0.8525 | 0.4364 | NA | NA | NA | NA |
| 14 | 0.7215 | 0.4041 | 3.3432 | 0.0799 | 2.0865 | 0.1615 | 4.7605 | 0.0391 |
| 15 | 0.6905 | 0.4139 | 2.6322 | 0.1173 | 3.4026 | 0.0770 | 0.1467 | 0.7050 |
| 16 | 0.0124 | 0.9126 | 0.4751 | 0.4994 | 0.9156 | 0.4215 | 0.6814 | 0.5209 |
| 17 | 3.7381 | 0.0634 | 1.3543 | 0.2544 | 0.5389 | 0.4690 | 0.8735 | 0.3580 |
| 18 | 0.5298 | 0.8063 | 1.6029 | 0.4476 | 1.5338 | 0.2182 | 1.4339 | 0.2509 |
| 19 | 2.6159 | 0.1170 | 0.0687 | 0.7952 | 0.0530 | 0.8197 | 6.7812 | 0.0146 |
| 20 | 0.1117 | 0.7410 | 3.4005 | 0.0771 | 0.0238 | 0.8787 | 0.8857 | 0.3557 |
| 21 | NA | NA | NA | NA | NA | NA | NA | NA |
| 22 | 0.7951 | 0.5117 | 1.1167 | 0.3671 | 0.0120 | 0.9881 | 2.7052 | 0.0890 |
| 23 | 0.7436 | 0.3961 | 10.2241 | 0.0035 | 2.8299 | 0.1041 | 3.1702 | 0.0863 |
| 24 | NA | NA | NA | NA | 0.6842 | 0.4231 | 0.0096 | 0.9234 |
| 25 | 1.2663 | 0.2700 | 0.0781 | 0.7820 | 5.5696 | 0.0255 | 0.5702 | 0.4565 |
| 26 | 0.5597 | 0.4598 | 0.2344 | 0.6316 | 1.7608 | 0.1939 | 0.5575 | 0.4607 |
| 27 | 0.7775 | 0.4696 | 3.6927 | 0.0382 | 1.8006 | 0.1897 | 1.0064 | 0.3238 |
| 28 | 0.5083 | 0.6095 | 0.7009 | 0.5085 | 0.8391 | 0.4475 | 0.6439 | 0.5363 |
| 29 | 0.0397 | 0.8435 | 3.8867 | 0.0586 | 0.1508 | 0.7009 | 0.0048 | 0.9454 |
| 30 | 0.0575 | 0.8120 | 0.0201 | 0.8882 | 0.6908 | 0.4120 | 0.0724 | 0.7896 |
| 31 | 1.2077 | 0.4109 | 0.3249 | 0.9401 | 0.2422 | 0.6261 | 0.0075 | 0.9316 |
| 32 | 0.1000 | 0.9052 | 3.0769 | 0.0663 | 2.2404 | 0.1301 | 7.0781 | 0.0042 |
| 33 | 1.0191 | 0.3206 | 1.6064 | 0.2144 | 0.4910 | 0.4887 | 0.0737 | 0.7878 |
| 34 | NA | NA | NA | NA | 1.1975 | 0.2882 | 2.3396 | 0.1435 |
| 35 | 0.0299 | 0.8639 | 0.0019 | 0.9655 | 0.7735 | 0.3864 | 2.5007 | 0.1246 |
| 36 | 0.0073 | 0.9325 | 0.0134 | 0.9087 | 0.7694 | 0.5566 | 0.1983 | 0.9366 |
| 37 | 3.8711 | 0.0371 | 2.5050 | 0.1058 | 3.0044 | 0.0712 | 1.6424 | 0.2174 |
| 38 | 1.1831 | 0.3559 | 1.3368 | 0.2937 | 0.7222 | 0.6154 | 0.4617 | 0.7995 |
| 39 | 0.2889 | 0.5957 | 0.3055 | 0.5854 | 0.3694 | 0.6954 | 0.7761 | 0.4724 |
| 40 | 2.3783 | 0.1347 | 4.4255 | 0.0449 | 0.2668 | 0.7681 | 1.3896 | 0.2685 |
| 41 | 1.8330 | 0.1842 | 0.0079 | 0.9297 | 0.4965 | 0.8037 | 0.7048 | 0.6492 |
| 42 | 0.4875 | 0.8430 | 1.5483 | 0.2386 | 0.3479 | 0.8428 | 0.3523 | 0.8398 |
| 43 | 2.2466 | 0.1578 | 0.1876 | 0.6721 | 1.2508 | 0.2836 | 1.3968 | 0.2584 |
| 44 | 3.1603 | 0.0568 | 0.4569 | 0.6376 | 0.2362 | 0.6302 | 0.6037 | 0.4427 |
| 45 | NA | NA | NA | NA | NA | NA | NA | NA |
| 46 | NA | NA | NA | NA | 1.9214 | 0.2089 | 0.9274 | 0.5160 |
| 47 | 0.0003 | 0.9861 | 0.0320 | 0.8593 | 1.0922 | 0.5686 | 10.5736 | 0.0894 |
| 48 | NA | NA | NA | NA | NA | NA | NA | NA |
| 49 | 0.0942 | 0.7615 | 0.4644 | 0.5018 | 0.8665 | 0.6893 | 3.4249 | 0.3979 |
| 50 | 0.6417 | 0.4299 | 0.0038 | 0.9516 | 3.2209 | 0.0835 | 0.2081 | 0.6518 |
| 51 | 0.0128 | 0.9103 | 3.1351 | 0.0834 | 1.0487 | 0.3959 | 3.0284 | 0.0299 |
| 52 | 0.4417 | 0.7254 | 1.0478 | 0.3903 | 0.4209 | 0.8277 | 0.8700 | 0.5213 |
| 53 | 1.0165 | 0.3841 | 0.0997 | 0.9056 | 0.8575 | 0.3661 | 0.6495 | 0.4303 |
| 54 | 0.6482 | 0.6979 | 15.2739 | 0.0099 | 0.0625 | 0.9396 | 0.7383 | 0.4935 |
| 55 | 4.1962 | 0.0521 | 0.8556 | 0.3646 | 0.3882 | 0.5394 | 2.1138 | 0.1595 |
| 56 | 2.2243 | 0.1470 | 1.7138 | 0.2011 | 1.0861 | 0.3063 | 0.3836 | 0.5407 |
| 57 | 1.9212 | 0.1406 | 0.4398 | 0.8149 | 2.2020 | 0.2798 | 1.1211 | 0.5216 |
| 58 | 6.4412 | 0.0069 | 1.2380 | 0.3113 | 0.4903 | 0.6196 | 0.9331 | 0.4098 |
| 59 | 1.9536 | 0.1749 | 0.8299 | 0.5026 | 1.5905 | 0.2364 | 0.1243 | 0.8840 |
| 60 | 2.0743 | 0.1563 | 1.5517 | 0.2655 | 0.7200 | 0.6457 | 1.0771 | 0.4479 |
| 61 | 0.3101 | 0.5830 | 2.1807 | 0.1533 | 8.8191 | 0.0069 | 0.1997 | 0.6591 |
| 62 | 6.5050 | 0.0179 | 1.1088 | 0.3033 | 0.2696 | 0.6085 | 0.7761 | 0.3875 |
| 63 | 0.0805 | 0.9868 | 2.1371 | 0.1387 | 0.5880 | 0.5658 | 3.6246 | 0.0476 |
| 64 | 2.9241 | 0.1020 | 0.8332 | 0.3717 | NA | NA | NA | NA |
| 65 | 6.8584 | 0.0137 | 0.0256 | 0.8739 | 11.7998 | 0.0002 | 0.1775 | 0.8383 |
| 66 | 1.8214 | 0.1895 | 1.7832 | 0.1965 | 0.0545 | 0.8178 | 0.1215 | 0.7310 |
| 67 | 1.3500 | 0.2736 | 0.4782 | 0.6242 | 0.5353 | 0.5907 | 2.8370 | 0.0734 |
| 68 | NA | NA | NA | NA | 1.7453 | 0.1925 | 0.3722 | 0.8248 |
| 69 | 0.7197 | 0.5519 | 0.8374 | 0.4892 | NA | NA | NA | NA |
| 70 | 0.3277 | 0.5707 | 0.6448 | 0.4274 | 0.1109 | 0.7411 | 0.3951 | 0.5337 |
| 71 | NA | NA | NA | NA | 0.7018 | 0.7369 | 914.5844 | 0.0257 |
| 72 | 0.4873 | 0.7450 | 0.8822 | 0.4979 | 0.4662 | 0.8174 | 2.5155 | 0.1032 |
| 73 | 1.1046 | 0.3029 | 3.4644 | 0.0740 | 1.6557 | 0.4326 | 2.7496 | 0.2951 |
| 74 | 0.4034 | 0.6729 | 0.3436 | 0.7130 | 1.5484 | 0.3547 | 0.3494 | 0.9040 |
| 75 | 0.1769 | 0.6774 | 1.2094 | 0.2812 | 0.0151 | 0.9031 | 1.0730 | 0.3095 |
| 76 | 0.4785 | 0.6263 | 0.1946 | 0.8246 | 0.1283 | 0.7233 | 0.2073 | 0.6530 |
| 77 | 1.4919 | 0.2489 | 1.2828 | 0.2991 | 0.1592 | 0.6936 | 1.1439 | 0.2959 |
| 78 | 1.1466 | 0.2959 | 0.5219 | 0.4776 | 5.2519 | 0.0319 | 0.8598 | 0.3639 |
| 79 | 2.7860 | 0.1093 | 0.0210 | 0.8860 | 4.4944 | 0.0455 | 0.1337 | 0.7181 |
| 80 | NA | NA | NA | NA | NA | NA | NA | NA |
| 81 | 0.8385 | 0.3698 | 0.5964 | 0.4482 | 0.0254 | 0.8749 | 0.0005 | 0.9827 |
| 82 | 0.6391 | 0.4308 | 0.0160 | 0.9003 | 0.8152 | 0.3743 | 1.2807 | 0.2674 |
| 83 | 0.0219 | 0.8834 | 0.0303 | 0.8633 | 0.2886 | 0.5957 | 0.0159 | 0.9005 |
| 84 | 2.6145 | 0.0923 | 1.3449 | 0.2781 | 0.1924 | 0.9005 | 1.7096 | 0.1929 |
| 85 | 0.4272 | 0.8577 | 1.4073 | 0.3928 | 0.8921 | 0.5891 | 1.9212 | 0.2761 |
| 86 | 7.0521 | 0.0055 | 0.4242 | 0.6607 | 2.9659 | 0.0770 | 0.4801 | 0.6264 |
| 87 | 0.3913 | 0.6798 | 0.3279 | 0.7232 | 0.3710 | 0.6934 | 0.2343 | 0.7926 |
| 88 | NA | NA | NA | NA | NA | NA | NA | NA |
| 89 | 0.1181 | 0.7343 | 0.1324 | 0.7193 | 1.0867 | 0.3080 | 0.2500 | 0.6218 |
| 90 | 1.1182 | 0.3008 | 2.8605 | 0.1037 | 0.2163 | 0.6461 | 0.3123 | 0.5815 |
| 91 | 0.4494 | 0.5085 | 0.4801 | 0.4945 | 0.0094 | 0.9234 | 0.1072 | 0.7460 |
| 92 | 0.9467 | 0.5042 | 0.1493 | 0.9849 | 0.4909 | 0.8016 | 0.5419 | 0.7660 |
| 93 | 0.4382 | 0.7790 | 0.7369 | 0.5821 | 0.1475 | 0.9299 | 2.0001 | 0.1522 |
| 94 | 1.3528 | 0.2567 | 1.0023 | 0.3272 | 0.0950 | 0.7607 | 1.7942 | 0.1935 |
| 95 | 9.8000 | 0.0010 | 0.3052 | 0.7402 | 1.5211 | 0.2462 | 0.9079 | 0.4842 |
| 96 | 1.3918 | 0.2461 | 0.0152 | 0.9025 | 5.6334 | 0.0232 | 0.0262 | 0.8724 |
| 97 | 2.1430 | 0.1540 | 0.8411 | 0.3666 | 0.0630 | 0.8035 | 0.0933 | 0.7622 |
| 98 | 0.4838 | 0.4931 | 0.8127 | 0.3759 | 0.2664 | 0.6103 | 0.2789 | 0.6021 |
| 99 | 0.9932 | 0.4433 | 0.7608 | 0.5678 | 0.0540 | 0.8183 | 1.2135 | 0.2820 |
| 100 | NA | NA | NA | NA | 2.1058 | 0.1626 | 8.1090 | 0.0047 |
| 101 | 0.7803 | 0.6411 | 1.1360 | 0.4649 | 1.0676 | 0.4942 | 0.6867 | 0.6973 |
| 102 | 1.1254 | 0.3794 | 3.9259 | 0.0209 | 1.0291 | 0.4224 | 2.0455 | 0.1361 |
| **5-Minute Data Resolution** | | | | | | | | |
| 1 | NA | NA | NA | NA | NA | NA | NA | NA |
| 2 | NA | NA | NA | NA | NA | NA | NA | NA |
| 3 | NA | NA | NA | NA | NA | NA | NA | NA |
| 4 | NA | NA | NA | NA | NA | NA | NA | NA |
| 5 | NA | NA | NA | NA | NA | NA | NA | NA |
| 6 | NA | NA | NA | NA | NA | NA | NA | NA |
| 7 | NA | NA | NA | NA | NA | NA | NA | NA |
| 8 | NA | NA | NA | NA | NA | NA | NA | NA |
| 9 | NA | NA | NA | NA | NA | NA | NA | NA |
| 10 | NA | NA | NA | NA | NA | NA | NA | NA |
| 11 | NA | NA | NA | NA | NA | NA | NA | NA |
| 12 | NA | NA | NA | NA | NA | NA | NA | NA |
| 13 | NA | NA | NA | NA | NA | NA | NA | NA |
| 14 | NA | NA | NA | NA | NA | NA | NA | NA |
| 15 | NA | NA | NA | NA | NA | NA | NA | NA |
| 16 | NA | NA | NA | NA | NA | NA | NA | NA |
| 17 | NA | NA | NA | NA | NA | NA | NA | NA |
| 18 | NA | NA | NA | NA | NA | NA | NA | NA |
| 19 | NA | NA | NA | NA | NA | NA | NA | NA |
| 20 | NA | NA | NA | NA | NA | NA | NA | NA |
| 21 | NA | NA | NA | NA | NA | NA | NA | NA |
| 22 | NA | NA | NA | NA | NA | NA | NA | NA |
| 23 | NA | NA | NA | NA | NA | NA | NA | NA |
| 24 | NA | NA | NA | NA | NA | NA | NA | NA |
| 25 | NA | NA | NA | NA | NA | NA | NA | NA |
| 26 | NA | NA | NA | NA | NA | NA | NA | NA |
| 27 | NA | NA | NA | NA | NA | NA | NA | NA |
| 28 | NA | NA | NA | NA | NA | NA | NA | NA |
| 29 | NA | NA | NA | NA | NA | NA | NA | NA |
| 30 | NA | NA | NA | NA | NA | NA | NA | NA |
| 31 | NA | NA | NA | NA | NA | NA | NA | NA |
| 32 | NA | NA | NA | NA | NA | NA | NA | NA |
| 33 | NA | NA | NA | NA | NA | NA | NA | NA |
| 34 | NA | NA | NA | NA | NA | NA | NA | NA |
| 35 | NA | NA | NA | NA | NA | NA | NA | NA |
| 36 | NA | NA | NA | NA | NA | NA | NA | NA |
| 37 | NA | NA | NA | NA | NA | NA | NA | NA |
| 38 | NA | NA | NA | NA | NA | NA | NA | NA |
| 39 | NA | NA | NA | NA | NA | NA | NA | NA |
| 40 | NA | NA | NA | NA | NA | NA | NA | NA |
| 41 | NA | NA | NA | NA | NA | NA | NA | NA |
| 42 | NA | NA | NA | NA | NA | NA | NA | NA |
| 43 | NA | NA | NA | NA | NA | NA | NA | NA |
| 44 | NA | NA | NA | NA | NA | NA | NA | NA |
| 45 | NA | NA | NA | NA | NA | NA | NA | NA |
| 46 | NA | NA | NA | NA | NA | NA | NA | NA |
| 47 | NA | NA | NA | NA | NA | NA | NA | NA |
| 48 | NA | NA | NA | NA | NA | NA | NA | NA |
| 49 | NA | NA | NA | NA | NA | NA | NA | NA |
| 50 | NA | NA | NA | NA | NA | NA | NA | NA |
| 51 | NA | NA | NA | NA | NA | NA | NA | NA |
| 52 | NA | NA | NA | NA | NA | NA | NA | NA |
| 53 | NA | NA | NA | NA | NA | NA | NA | NA |
| 54 | NA | NA | NA | NA | NA | NA | NA | NA |
| 55 | NA | NA | NA | NA | NA | NA | NA | NA |
| 56 | NA | NA | NA | NA | NA | NA | NA | NA |
| 57 | NA | NA | NA | NA | NA | NA | NA | NA |
| 58 | NA | NA | NA | NA | NA | NA | NA | NA |
| 59 | NA | NA | NA | NA | NA | NA | NA | NA |
| 60 | NA | NA | NA | NA | NA | NA | NA | NA |
| 61 | NA | NA | NA | NA | NA | NA | NA | NA |
| 62 | NA | NA | NA | NA | NA | NA | NA | NA |
| 63 | NA | NA | NA | NA | NA | NA | NA | NA |
| 64 | NA | NA | NA | NA | NA | NA | NA | NA |
| 65 | NA | NA | NA | NA | NA | NA | NA | NA |
| 66 | NA | NA | NA | NA | NA | NA | NA | NA |
| 67 | NA | NA | NA | NA | NA | NA | NA | NA |
| 68 | NA | NA | NA | NA | NA | NA | NA | NA |
| 69 | NA | NA | NA | NA | NA | NA | NA | NA |
| 70 | NA | NA | NA | NA | NA | NA | NA | NA |
| 71 | NA | NA | NA | NA | NA | NA | NA | NA |
| 72 | NA | NA | NA | NA | NA | NA | NA | NA |
| 73 | NA | NA | NA | NA | NA | NA | NA | NA |
| 74 | NA | NA | NA | NA | NA | NA | NA | NA |
| 75 | NA | NA | NA | NA | NA | NA | NA | NA |
| 76 | NA | NA | NA | NA | NA | NA | NA | NA |
| 77 | NA | NA | NA | NA | NA | NA | NA | NA |
| 78 | NA | NA | NA | NA | NA | NA | NA | NA |
| 79 | NA | NA | NA | NA | NA | NA | NA | NA |
| 80 | NA | NA | NA | NA | NA | NA | NA | NA |
| 81 | NA | NA | NA | NA | NA | NA | NA | NA |
| 82 | NA | NA | NA | NA | NA | NA | NA | NA |
| 83 | NA | NA | NA | NA | NA | NA | NA | NA |
| 84 | NA | NA | NA | NA | NA | NA | NA | NA |
| 85 | NA | NA | NA | NA | NA | NA | NA | NA |
| 86 | NA | NA | NA | NA | NA | NA | NA | NA |
| 87 | NA | NA | NA | NA | NA | NA | NA | NA |
| 88 | NA | NA | NA | NA | NA | NA | NA | NA |
| 89 | NA | NA | NA | NA | NA | NA | NA | NA |
| 90 | NA | NA | NA | NA | NA | NA | NA | NA |
| 91 | NA | NA | NA | NA | NA | NA | NA | NA |
| 92 | NA | NA | NA | NA | NA | NA | NA | NA |
| 93 | NA | NA | NA | NA | NA | NA | NA | NA |
| 94 | NA | NA | NA | NA | NA | NA | NA | NA |
| 95 | NA | NA | NA | NA | NA | NA | NA | NA |
| 96 | NA | NA | NA | NA | NA | NA | NA | NA |
| 97 | NA | NA | NA | NA | NA | NA | NA | NA |
| 98 | NA | NA | NA | NA | NA | NA | NA | NA |
| 99 | NA | NA | NA | NA | NA | NA | NA | NA |
| 100 | NA | NA | NA | NA | NA | NA | NA | NA |
| 101 | NA | NA | NA | NA | NA | NA | NA | NA |
| 102 | NA | NA | NA | NA | NA | NA | NA | NA |
| *ABP, arterial blood pressure; HC, healthy control volunteer group; rSO_2_, regional cerebral oxygen saturation.* | | | | | | | | |

File S7b: Granger Causality Test Results for SP Population in 10-Second, 1-Minute, and 5-Minute Data Resolutions

| **Patient** | **ABP ® rSO_2__L (F Statistic)** | **ABP ® rSO_2__L (P-Value)** | **rSO_2__L ® ABP (F Statistic)** | **rSO_2__L ® ABP (P-Value)** | **ABP ® rSO_2__R (F Statistic)** | **ABP ® rSO_2__R (P-Value)** | **rSO_2__R ® ABP (F Statistic)** | **rSO_2__R ® ABP (P-Value)** |
| --- | --- | --- | --- | --- | --- | --- | --- | --- |
| **10-Second Data Resolution** | | | | | | | | |
| 1 | 2.2126 | 0.0397 | 1.0147 | 0.4140 | 3.5070 | 0.0003 | 0.7517 | 0.6615 |
| 2 | 0.5017 | 0.9999 | 0.6363 | 0.9926 | 0.4337 | 1.0000 | 0.3630 | 1.0000 |
| 3 | 1.1466 | 0.2283 | 0.5132 | 0.9978 | 1.0089 | 0.4564 | 0.7366 | 0.9109 |
| 4 | 2.3668 | 0.0159 | 1.7386 | 0.0856 | 1.2135 | 0.1168 | 0.8514 | 0.8038 |
| 5 | 11.8061 | 0.0000 | 0.6723 | 0.6957 | 2.7475 | 0.0000 | 1.1253 | 0.2605 |
| 6 | 2.1769 | 0.0275 | 2.2580 | 0.0220 | 20.0394 | 0.0000 | 2.8450 | 0.0096 |
| 7 | 0.1421 | 1.0000 | 0.4369 | 0.9955 | 0.2110 | 1.0000 | 1.3333 | 0.0819 |
| 8 | 0.4376 | 0.7815 | 0.0123 | 0.9997 | 0.2741 | 0.9980 | 0.6457 | 0.8478 |
| 9 | 20.5566 | 0.0000 | 9.2741 | 0.0000 | 2.1397 | 0.0298 | 1.1198 | 0.3469 |
| 10 | 4.3703 | 0.0046 | 1.0539 | 0.3678 | 4.5587 | 0.0035 | 0.4753 | 0.6996 |
| 11 | 2.0905 | 0.0001 | 6.3483 | 0.0000 | 0.5663 | 0.6871 | 0.3032 | 0.8759 |
| 12 | 0.9296 | 0.5609 | 1.1437 | 0.2874 | 0.9623 | 0.5734 | 1.2741 | 0.0600 |
| 13 | 1.0187 | 0.4257 | 0.7534 | 0.6740 | 2.4372 | 0.0005 | 1.8115 | 0.0165 |
| 14 | 0.9738 | 0.4550 | 0.7078 | 0.6848 | 2.9756 | 0.0308 | 3.0601 | 0.0275 |
| 15 | 1.8554 | 0.0037 | 1.3469 | 0.1022 | 2.4464 | 0.0000 | 1.5087 | 0.0090 |
| 16 | 1.6625 | 0.0015 | 0.5484 | 0.9982 | 1.3155 | 0.2396 | 0.1476 | 0.9942 |
| 17 | 1.1455 | 0.2013 | 0.9166 | 0.6698 | 0.7571 | 0.9294 | 0.8936 | 0.7189 |
| 18 | 1.6447 | 0.0369 | 0.9790 | 0.4854 | 0.7946 | 0.8688 | 0.3830 | 1.0000 |
| 19 | 3.6563 | 0.0000 | 0.3318 | 1.0000 | 4.4488 | 0.0000 | 1.8345 | 0.0003 |
| 20 | 0.3864 | 1.0000 | 0.8343 | 0.8396 | 0.6763 | 0.9841 | 0.6653 | 0.9872 |
| 21 | 0.2001 | 1.0000 | 0.3257 | 1.0000 | 0.3526 | 1.0000 | 0.2229 | 1.0000 |
| 22 | 4.0858 | 0.0171 | 0.8207 | 0.4404 | 0.5921 | 0.7369 | 4.0254 | 0.0005 |
| 23 | 2.2592 | 0.0000 | 1.5880 | 0.0131 | 2.8057 | 0.0000 | 2.5458 | 0.0000 |
| 24 | 0.8407 | 0.8325 | 0.7200 | 0.9661 | 0.6567 | 0.9505 | 1.2593 | 0.1344 |
| 25 | 7.4140 | 0.0000 | 1.8492 | 0.0561 | 39.5156 | 0.0000 | 0.7624 | 0.5153 |
| 26 | 3.3463 | 0.0000 | 1.0198 | 0.4375 | 2.3451 | 0.0000 | 1.6302 | 0.0155 |
| 27 | 5.0494 | 0.0000 | 0.9727 | 0.5204 | 3.6516 | 0.0000 | 2.1190 | 0.0030 |
| **1-Minute Data Resolution** | | | | | | | | |
| 1 | 2.8089 | 0.0032 | 0.7997 | 0.6292 | 4.9892 | 0.0008 | 0.1382 | 0.9679 |
| 2 | 0.6359 | 0.6372 | 2.2967 | 0.0586 | 0.7458 | 0.7062 | 1.2095 | 0.2744 |
| 3 | 0.9135 | 0.3397 | 0.2174 | 0.6412 | 1.3364 | 0.2483 | 0.0065 | 0.9357 |
| 4 | 1.9197 | 0.0146 | 1.3165 | 0.1773 | 2.3162 | 0.0030 | 1.0353 | 0.4285 |
| 5 | 2.9409 | 0.0001 | 0.8261 | 0.6972 | 2.7834 | 0.0019 | 0.9170 | 0.5317 |
| 6 | 1.9224 | 0.0976 | 13.6129 | 0.0000 | NA | NA | NA | NA |
| 7 | 0.4247 | 0.9654 | 0.5155 | 0.9221 | 0.5533 | 0.8976 | 1.1180 | 0.3452 |
| 8 | 0.0042 | 0.9485 | 0.0041 | 0.9491 | 0.1593 | 0.6902 | 3.1699 | 0.0766 |
| 9 | 2.7860 | 0.0009 | 0.4941 | 0.9402 | 2.1376 | 0.0519 | 2.1452 | 0.0511 |
| 10 | 2.7313 | 0.0105 | 0.5034 | 0.8310 | 2.3921 | 0.0373 | 0.8379 | 0.6940 |
| 11 | 0.3982 | 0.9212 | 4.3054 | 0.0001 | 0.5330 | 0.8314 | 0.4566 | 0.8859 |
| 12 | 1.1196 | 0.3492 | 1.6812 | 0.1568 | 2.9078 | 0.0234 | 3.8656 | 0.0050 |
| 13 | 0.7316 | 0.7654 | 1.1345 | 0.4414 | NA | NA | NA | NA |
| 14 | 0.7436 | 0.5279 | 1.9758 | 0.1205 | 1.2494 | 0.2898 | 6.1756 | 0.0027 |
| 15 | 0.9385 | 0.5739 | 0.9991 | 0.4918 | 2.0761 | 0.0223 | 1.8445 | 0.0469 |
| 16 | 1.0794 | 0.3860 | 2.0412 | 0.0120 | NA | NA | NA | NA |
| 17 | 0.9754 | 0.5039 | 1.0260 | 0.4413 | 1.5383 | 0.1933 | 0.3151 | 0.8676 |
| 18 | 0.4547 | 0.8094 | 0.6049 | 0.6962 | 1.3632 | 0.2037 | 0.2078 | 0.9953 |
| 19 | 2.0704 | 0.0106 | 2.6367 | 0.0009 | 0.4208 | 0.9866 | 0.9134 | 0.6168 |
| 20 | 0.3407 | 0.9788 | 1.1479 | 0.3354 | 3.0951 | 0.0079 | 1.3480 | 0.2428 |
| 21 | 0.0108 | 0.9175 | 0.3478 | 0.5561 | 0.2238 | 0.6366 | 0.0563 | 0.8127 |
| 22 | 1.5810 | 0.1813 | 1.0013 | 0.4083 | 2.8497 | 0.0015 | 4.1605 | 0.0000 |
| 23 | NA | NA | NA | NA | NA | NA | NA | NA |
| 24 | 1.4028 | 0.1954 | 1.4400 | 0.1797 | 1.2004 | 0.3020 | 1.1456 | 0.3372 |
| 25 | 0.9500 | 0.3891 | 0.7653 | 0.4670 | 1.1472 | 0.3385 | 1.3450 | 0.2414 |
| 26 | 24.7515 | 0.0000 | 5.4046 | 0.0053 | 6.9716 | 0.0000 | 2.6213 | 0.0367 |
| 27 | 1.0600 | 0.4039 | 0.9153 | 0.5544 | 4.1947 | 0.0002 | 1.8443 | 0.0755 |
| **5-Minute Data Resolution** | | | | | | | | |
| 1 | 2.0429 | 0.1182 | 2.4583 | 0.0704 | 3.2657 | 0.0270 | 3.0620 | 0.0342 |
| 2 | 1.2224 | 0.3091 | 1.0519 | 0.3955 | 0.4014 | 0.8460 | 1.3132 | 0.2702 |
| 3 | 0.2224 | 0.9252 | 0.3726 | 0.8275 | 0.1531 | 0.9610 | 0.3969 | 0.8103 |
| 4 | 1.3079 | 0.3009 | 0.5683 | 0.6886 | 7.1690 | 0.0012 | 1.5299 | 0.2358 |
| 5 | 1.7266 | 0.1772 | 1.0313 | 0.4498 | 4.2889 | 0.0463 | 0.0010 | 0.9744 |
| 6 | NA | NA | NA | NA | NA | NA | NA | NA |
| 7 | 1.0189 | 0.3709 | 0.4979 | 0.6118 | 0.3054 | 0.7387 | 0.1910 | 0.8270 |
| 8 | 8.2507 | 0.0070 | 0.1876 | 0.6676 | 6.3370 | 0.0167 | 0.0000 | 0.9958 |
| 9 | 0.5120 | 0.8149 | 1.0917 | 0.5322 | 4.8011 | 0.0363 | 0.3604 | 0.5528 |
| 10 | 0.3400 | 0.5641 | 0.8072 | 0.3759 | 0.5592 | 0.5779 | 0.6715 | 0.5190 |
| 11 | 0.0600 | 0.9418 | 2.4130 | 0.0983 | 0.1915 | 0.6632 | 1.2210 | 0.2734 |
| 12 | 3.0105 | 0.0389 | 0.4129 | 0.8591 | 3.9750 | 0.0307 | 0.2531 | 0.7782 |
| 13 | 3.6379 | 0.0717 | 0.2296 | 0.6373 | NA | NA | NA | NA |
| 14 | 1.0708 | 0.4233 | 1.2161 | 0.3594 | NA | NA | NA | NA |
| 15 | 2.9670 | 0.0684 | 3.8362 | 0.0342 | 4.0292 | 0.0290 | 0.8335 | 0.4450 |
| 16 | 5.6436 | 0.0105 | 14.4603 | 0.0001 | 4.1452 | 0.0172 | 12.3889 | 0.0001 |
| 17 | 2.0526 | 0.1467 | 0.8098 | 0.4547 | 2.6339 | 0.0889 | 0.3600 | 0.7008 |
| 18 | 1.0065 | 0.3793 | 1.0212 | 0.3742 | 0.4199 | 0.5221 | 0.4294 | 0.5175 |
| 19 | 0.7482 | 0.3935 | 2.2085 | 0.1470 | 2.0250 | 0.1186 | 5.7874 | 0.0018 |
| 20 | NA | NA | NA | NA | 1.3738 | 0.3050 | 1.9635 | 0.1699 |
| 21 | 1.1965 | 0.2815 | 3.9043 | 0.0561 | 0.2846 | 0.5971 | 0.2788 | 0.6009 |
| 22 | 1.4873 | 0.2313 | 14.4306 | 0.0006 | 1.1497 | 0.3303 | 4.5806 | 0.0184 |
| 23 | 2.2376 | 0.1445 | 7.6693 | 0.0056 | 2.9987 | 0.0596 | 2.4186 | 0.1018 |
| 24 | 1.1037 | 0.3932 | 2.9642 | 0.0574 | NA | NA | NA | NA |
| 25 | 0.3011 | 0.5878 | 2.3571 | 0.1368 | 1.0009 | 0.3263 | 1.3642 | 0.2534 |
| 26 | 29.3323 | 0.0000 | 0.6527 | 0.4251 | 14.7820 | 0.0005 | 0.2443 | 0.6245 |
| 27 | 0.6680 | 0.6967 | 3.5984 | 0.0699 | 0.3795 | 0.8746 | 6.2984 | 0.0076 |
| *ABP, arterial blood pressure; rSO_2_, regional cerebral oxygen saturation; SP, elective spinal surgery patient group.* | | | | | | | | |

File S7c: Granger Causality Test Results for TBI-GLR Population in 10-Second, 1-Minute, and 5-Minute Data Resolutions

| **Patient** | **ABP ® rSO_2__L (F Statistic)** | **ABP ® rSO_2__L (P-Value)** | **rSO_2__L ® ABP (F Statistic)** | **rSO_2__L ® ABP (P-Value)** | **ABP ® rSO_2__R (F Statistic)** | **ABP ® rSO_2__R (P-Value)** | **rSO_2__R ® ABP (F Statistic)** | **rSO_2__R ® ABP (P-Value)** | **CPP ® rSO_2__L (F Statistic)** | **CPP ® rSO_2__L (P-Value)** | **rSO_2__L ® CPP (F Statistic)** | **rSO_2__L ® CPP (P-Value)** | **CPP ® rSO_2__R (F Statistic)** | **CPP ® rSO_2__R (P-Value)** | **rSO_2__R ® CPP (F Statistic)** | **rSO_2__R ® CPP (P-Value)** |
| --- | --- | --- | --- | --- | --- | --- | --- | --- | --- | --- | --- | --- | --- | --- | --- | --- |
| **10-Second Data Resolution** | | | | | | | | | | | | | | | | |
| 1 | 7.1334 | 0.0000 | 1.2487 | 0.2599 | 8.8304 | 0.0000 | 0.9846 | 0.4542 | 1.8425 | 0.0001 | 1.3837 | 0.0242 | 2.6868 | 0.0000 | 2.0577 | 0.0000 |
| 2 | 4.0376 | 0.0000 | 3.3161 | 0.0000 | 2.1971 | 0.0000 | 2.0526 | 0.0000 | 5.3249 | 0.0000 | 4.4647 | 0.0000 | 3.1870 | 0.0000 | 1.1456 | 0.2909 |
| 3 | 16.8840 | 0.0000 | 0.9317 | 0.5024 | 3.2529 | 0.0000 | 1.4387 | 0.0066 | 21.4593 | 0.0000 | 1.7683 | 0.0689 | 3.5222 | 0.0000 | 1.1538 | 0.1770 |
| 4 | 1.5562 | 0.0124 | 1.6399 | 0.0058 | 3.1402 | 0.0000 | 1.4483 | 0.0541 | 2.8672 | 0.0000 | 2.2716 | 0.0008 | 1.3627 | 0.1562 | 1.4681 | 0.1077 |
| 5 | 1.3611 | 0.0456 | 1.3960 | 0.0336 | 3.9344 | 0.0000 | 1.4589 | 0.1110 | 0.7198 | 0.9318 | 1.7907 | 0.0005 | 2.3456 | 0.0023 | 0.8624 | 0.6072 |
| 6 | 2.0533 | 0.0000 | 1.3237 | 0.0274 | 0.9792 | 0.5178 | 0.9012 | 0.6796 | 0.9143 | 0.7058 | 0.3423 | 1.0000 | 1.6761 | 0.0008 | 1.5264 | 0.0054 |
| 7 | 4.2560 | 0.0000 | 3.6092 | 0.0000 | 3.1599 | 0.0000 | 5.4544 | 0.0000 | 2.8817 | 0.0000 | 1.4134 | 0.0288 | 1.7820 | 0.0006 | 1.4186 | 0.0275 |
| 8 | 3.6890 | 0.0000 | 3.1459 | 0.0000 | 13.6234 | 0.0000 | 4.5083 | 0.0000 | 1.0806 | 0.3405 | 0.5723 | 0.9851 | 1.0034 | 0.4692 | 0.3575 | 1.0000 |
| 9 | 1.4786 | 0.0059 | 1.0877 | 0.2883 | 2.7161 | 0.0000 | 2.0455 | 0.0003 | 1.3087 | 0.0269 | 0.6149 | 0.9985 | 3.5800 | 0.0000 | 1.5720 | 0.0086 |
| 10 | 0.8217 | 0.8564 | 1.1156 | 0.2384 | 0.8435 | 0.7528 | 1.2268 | 0.1497 | 1.3573 | 0.0349 | 1.2063 | 0.1324 | 0.9519 | 0.5511 | 1.3780 | 0.0656 |
| 11 | 40.7648 | 0.0000 | 8.9073 | 0.0000 | 30.3572 | 0.0000 | 7.9963 | 0.0000 | 23.5999 | 0.0000 | 5.7977 | 0.0000 | 15.6234 | 0.0000 | 5.2113 | 0.0000 |
| 12 | 5.3671 | 0.0000 | 2.5551 | 0.0000 | 7.3162 | 0.0000 | 1.6998 | 0.0010 | 4.7862 | 0.0000 | 1.9451 | 0.0000 | 9.1772 | 0.0000 | 1.6553 | 0.0004 |
| 13 | 7.4315 | 0.0000 | 2.4772 | 0.0000 | 4.2820 | 0.0000 | 1.5425 | 0.0024 | 28.0907 | 0.0000 | 12.6651 | 0.0000 | 23.0413 | 0.0000 | 9.4810 | 0.0000 |
| 14 | 2.3260 | 0.0000 | 1.1586 | 0.1577 | 2.9082 | 0.0000 | 1.6348 | 0.0003 | 3.2485 | 0.0000 | 1.2807 | 0.0708 | 3.4498 | 0.0000 | 1.4669 | 0.0109 |
| 15 | 3.0008 | 0.0000 | 0.8864 | 0.7561 | 0.9807 | 0.5279 | 0.5920 | 0.9986 | 2.2586 | 0.0000 | 1.3827 | 0.0232 | 0.9652 | 0.5557 | 0.7039 | 0.9652 |
| 16 | 11.9299 | 0.0000 | 3.2828 | 0.0000 | 17.4943 | 0.0000 | 4.3124 | 0.0000 | 11.7494 | 0.0000 | 3.3521 | 0.0000 | 19.6011 | 0.0000 | 4.1852 | 0.0000 |
| 17 | 7.1020 | 0.0000 | 1.8092 | 0.0000 | 6.9870 | 0.0000 | 1.9695 | 0.0000 | 7.4210 | 0.0000 | 1.2569 | 0.0699 | 3.3974 | 0.0000 | 2.3746 | 0.0000 |
| 18 | 4.0925 | 0.0000 | 2.7816 | 0.0000 | 11.3727 | 0.0000 | 3.9900 | 0.0000 | 3.6916 | 0.0000 | 2.8103 | 0.0000 | 10.6706 | 0.0000 | 3.7032 | 0.0000 |
| 19 | 14.2953 | 0.0000 | 1.9961 | 0.0004 | 3.4833 | 0.0000 | 1.5553 | 0.0084 | 10.8186 | 0.0000 | 2.2828 | 0.0000 | 6.2903 | 0.0000 | 1.7053 | 0.0017 |
| 20 | 14.2799 | 0.0000 | 7.3186 | 0.0000 | 16.4070 | 0.0000 | 6.7841 | 0.0000 | 12.2982 | 0.0000 | 5.2107 | 0.0000 | 13.1630 | 0.0000 | 5.9831 | 0.0000 |
| 21 | 1.7664 | 0.0012 | 1.8416 | 0.0005 | 2.6223 | 0.0000 | 2.6682 | 0.0000 | 1.5551 | 0.0074 | 2.7482 | 0.0000 | 1.5921 | 0.0006 | 2.9374 | 0.0000 |
| 22 | 8.9703 | 0.0000 | 2.7747 | 0.0000 | 6.9536 | 0.0000 | 1.1357 | 0.2055 | 4.5200 | 0.0000 | 1.5105 | 0.0056 | 4.1619 | 0.0000 | 1.5172 | 0.0012 |
| 23 | 2.8711 | 0.0000 | 0.8652 | 0.7996 | 2.2973 | 0.0000 | 1.7006 | 0.0002 | 3.7015 | 0.0000 | 1.4441 | 0.0090 | 1.4009 | 0.0197 | 1.4613 | 0.0100 |
| 24 | 36.8372 | 0.0000 | 2.1231 | 0.0244 | 34.5139 | 0.0000 | 2.4480 | 0.0229 | 12.5282 | 0.0000 | 1.5503 | 0.0421 | 13.2389 | 0.0000 | 2.2742 | 0.0026 |
| 25 | 378.8441 | 0.0000 | 35.6146 | 0.0000 | 229.8952 | 0.0000 | 10.2185 | 0.0000 | 30.2875 | 0.0000 | 42.2634 | 0.0000 | 422.2722 | 0.0000 | 49.6380 | 0.0000 |
| 26 | 6.7254 | 0.0000 | 2.2339 | 0.0000 | 14.6032 | 0.0000 | 1.9600 | 0.0000 | 5.8394 | 0.0000 | 2.3410 | 0.0000 | 11.2419 | 0.0000 | 1.7411 | 0.0000 |
| 27 | 1.3398 | 0.0435 | 1.8297 | 0.0001 | 0.6947 | 0.8352 | 1.2188 | 0.2278 | 2.1435 | 0.0001 | 1.0514 | 0.3884 | 3.1376 | 0.0001 | 2.0843 | 0.0114 |
| 28 | 3.4852 | 0.0000 | 2.0154 | 0.0000 | 1.9493 | 0.0000 | 2.2561 | 0.0000 | 3.8348 | 0.0000 | 2.0048 | 0.0000 | 6.3283 | 0.0000 | 1.6602 | 0.0001 |
| 29 | 0.4484 | 0.9982 | 10.7313 | 0.0000 | 4.1501 | 0.0000 | 0.9945 | 0.4811 | 0.8450 | 0.5349 | 0.6866 | 0.6605 | 6.5932 | 0.0000 | 0.7370 | 0.6406 |
| 30 | 2.2123 | 0.0000 | 0.9939 | 0.4850 | 3.5676 | 0.0000 | 0.3165 | 1.0000 | 1.5870 | 0.0027 | 0.7644 | 0.9102 | 2.4678 | 0.0000 | 0.2851 | 1.0000 |
| 31 | 0.6654 | 0.9816 | 0.4659 | 0.9999 | 0.7761 | 0.8884 | 1.1160 | 0.2580 | 0.5483 | 0.9996 | 0.4747 | 1.0000 | 0.6620 | 0.9866 | 1.0180 | 0.4362 |
| 32 | 3.4534 | 0.0000 | 2.8085 | 0.0000 | 1.9064 | 0.0000 | 0.4679 | 1.0000 | 2.6302 | 0.0000 | 3.2103 | 0.0000 | 1.6299 | 0.0001 | 0.5196 | 1.0000 |
| 33 | 1.0815 | 0.2804 | 0.8389 | 0.8630 | 0.9197 | 0.6926 | 1.5760 | 0.0004 | 1.4427 | 0.0039 | 1.0657 | 0.3155 | 1.4929 | 0.0017 | 1.8054 | 0.0000 |
| 34 | 18.0735 | 0.0000 | 6.6315 | 0.0000 | 3.4661 | 0.0002 | 1.9345 | 0.0366 | 14.7720 | 0.0000 | 5.0890 | 0.0004 | 2.7349 | 0.0024 | 1.7338 | 0.0679 |
| 35 | 0.8505 | 0.8570 | 1.4291 | 0.0032 | 1.3950 | 0.0056 | 1.2502 | 0.0460 | 0.8021 | 0.9272 | 1.5995 | 0.0001 | 1.3352 | 0.0142 | 1.1678 | 0.1207 |
| 36 | 0.3047 | 1.0000 | 0.4012 | 1.0000 | 0.7475 | 0.9643 | 0.9056 | 0.7261 | 0.3099 | 1.0000 | 0.4870 | 1.0000 | 0.9591 | 0.5967 | 1.2450 | 0.0504 |
| 37 | 3.5026 | 0.0000 | 1.0811 | 0.3113 | 1.1854 | 0.1112 | 0.4491 | 1.0000 | 2.3111 | 0.0000 | 1.2316 | 0.1448 | 1.3883 | 0.0227 | 0.5367 | 0.9990 |
| 38 | 9.5175 | 0.0000 | 7.8523 | 0.0000 | 25.0077 | 0.0000 | 9.2797 | 0.0000 | 12.6474 | 0.0000 | 12.0993 | 0.0000 | 19.2918 | 0.0000 | 10.7560 | 0.0000 |
| 39 | 3.0450 | 0.0000 | 2.2143 | 0.0000 | 0.5213 | 0.9992 | 0.4785 | 0.9998 | 4.1446 | 0.0000 | 2.3359 | 0.0000 | 0.4727 | 0.9999 | 0.6272 | 0.9938 |
| 40 | 2.2565 | 0.0000 | 0.4801 | 1.0000 | 10.1719 | 0.0000 | 2.1962 | 0.0024 | 1.1051 | 0.2656 | 0.7529 | 0.9274 | 0.8142 | 0.6545 | 0.2943 | 0.9947 |
| 41 | 3.3505 | 0.0000 | 2.4581 | 0.0000 | 8.6624 | 0.0000 | 3.8184 | 0.0000 | 3.2520 | 0.0000 | 2.4116 | 0.0000 | 8.6131 | 0.0000 | 3.6709 | 0.0000 |
| 42 | 4.3170 | 0.0000 | 0.6156 | 0.9883 | 2.2233 | 0.0000 | 0.3785 | 1.0000 | 2.4201 | 0.0000 | 1.5664 | 0.0250 | 1.5245 | 0.0179 | 0.6278 | 0.9682 |
| 43 | 17.1869 | 0.0000 | 2.1219 | 0.0197 | 3.5987 | 0.0001 | 68.2156 | 0.0000 | 18.1672 | 0.0000 | 2.3936 | 0.0078 | 7.8486 | 0.0000 | 4.6153 | 0.0000 |
| 44 | 7.6391 | 0.0000 | 4.1879 | 0.0000 | 6.9567 | 0.0000 | 4.3179 | 0.0000 | 6.2867 | 0.0000 | 4.2315 | 0.0000 | 7.2499 | 0.0000 | 4.7415 | 0.0000 |
| 45 | 22.7775 | 0.0000 | 9.0004 | 0.0000 | 19.0358 | 0.0000 | 8.0639 | 0.0000 | 37.1063 | 0.0000 | 21.0852 | 0.0000 | 28.9430 | 0.0000 | 16.7282 | 0.0000 |
| 46 | 15.4985 | 0.0000 | 5.1263 | 0.0000 | 15.0037 | 0.0000 | 3.6140 | 0.0000 | 56.0017 | 0.0000 | 20.0845 | 0.0000 | 28.8626 | 0.0000 | 27.6125 | 0.0000 |
| 47 | 54.8504 | 0.0000 | 4.6302 | 0.0000 | 25.9524 | 0.0000 | 3.2159 | 0.0000 | 19.6994 | 0.0000 | 2.2713 | 0.0000 | 13.4258 | 0.0000 | 1.8414 | 0.0000 |
| 48 | 9.8893 | 0.0000 | 3.8517 | 0.0000 | 8.1127 | 0.0000 | 3.0483 | 0.0000 | 10.7037 | 0.0000 | 3.5864 | 0.0000 | 4.7532 | 0.0000 | 2.3346 | 0.0000 |
| 49 | 9.3547 | 0.0000 | 2.4224 | 0.0000 | 40.3574 | 0.0000 | 2.1821 | 0.0328 | 0.4812 | 0.8679 | 0.9131 | 0.5077 | 3.4074 | 0.0668 | 0.4226 | 0.5166 |
| 50 | 2.2468 | 0.0000 | 0.7592 | 0.9652 | 3.0816 | 0.0000 | 0.7184 | 0.9849 | 0.6531 | 0.9971 | 0.5739 | 0.9998 | 2.4408 | 0.0000 | 0.8444 | 0.8675 |
| 51 | 1.8640 | 0.0000 | 2.5381 | 0.0000 | 1.1548 | 0.1501 | 1.9633 | 0.0000 | 3.3524 | 0.0000 | 3.2728 | 0.0000 | 1.1683 | 0.1432 | 2.2195 | 0.0000 |
| 52 | 2.6019 | 0.0000 | 0.9484 | 0.6269 | 2.2326 | 0.0000 | 1.1010 | 0.2308 | 2.7617 | 0.0000 | 0.8396 | 0.8753 | 2.3185 | 0.0000 | 1.0547 | 0.3352 |
| 53 | 84.8412 | 0.0000 | 2.9910 | 0.0014 | 5.3229 | 0.0000 | 0.6657 | 0.9911 | 99.9098 | 0.0000 | 3.6015 | 0.0001 | 5.8501 | 0.0000 | 0.7971 | 0.9213 |
| 54 | 17.6192 | 0.0000 | 1.5294 | 0.0044 | 2.3367 | 0.0000 | 1.1567 | 0.2107 | 16.9068 | 0.0000 | 1.4004 | 0.1194 | 2.1710 | 0.0068 | 1.0171 | 0.4321 |
| 55 | 3.8466 | 0.0000 | 2.0515 | 0.0008 | 3.6111 | 0.0000 | 1.8646 | 0.0003 | 3.0493 | 0.0000 | 2.2551 | 0.0003 | 3.1898 | 0.0000 | 1.9016 | 0.0004 |
| 56 | 3.1079 | 0.0000 | 1.2021 | 0.1165 | 10.2632 | 0.0000 | 1.9898 | 0.0000 | 1.7562 | 0.0000 | 1.2432 | 0.0592 | 3.6818 | 0.0000 | 1.7822 | 0.0000 |
| 57 | 2.1364 | 0.0000 | 1.4840 | 0.0020 | 2.4569 | 0.0000 | 0.9243 | 0.6812 | 3.7232 | 0.0000 | 2.2036 | 0.0002 | 55.4986 | 0.0000 | 2.4541 | 0.0000 |
| 58 | 2.1693 | 0.0000 | 1.4635 | 0.0097 | 1.2559 | 0.0728 | 1.0230 | 0.4241 | 2.3979 | 0.0000 | 1.4195 | 0.0160 | 1.0608 | 0.3415 | 0.9568 | 0.5802 |
| 59 | 2.9577 | 0.0000 | 2.0062 | 0.0000 | 4.0061 | 0.0000 | 2.0694 | 0.0000 | 2.7741 | 0.0000 | 1.3366 | 0.0140 | 4.7780 | 0.0000 | 1.6217 | 0.0001 |
| 60 | 1.9671 | 0.0000 | 2.1210 | 0.0000 | 1.5554 | 0.0021 | 2.3072 | 0.0000 | 1.5265 | 0.0031 | 2.2558 | 0.0000 | 1.0748 | 0.3132 | 2.2401 | 0.0000 |
| 61 | 1.7116 | 0.0001 | 3.0340 | 0.0000 | 2.5434 | 0.0000 | 2.9268 | 0.0000 | 1.6152 | 0.0004 | 2.1078 | 0.0000 | 1.5875 | 0.0003 | 2.2712 | 0.0000 |
| 62 | 1.5704 | 0.0002 | 1.8113 | 0.0000 | 1.4428 | 0.0059 | 2.3850 | 0.0000 | 1.2738 | 0.0925 | 1.5808 | 0.0055 | 3.9064 | 0.0000 | 0.8234 | 0.7781 |
| 63 | 3.3745 | 0.0000 | 2.5534 | 0.0000 | 3.2384 | 0.0000 | 2.8698 | 0.0000 | 1.3444 | 0.0390 | 1.6309 | 0.0015 | 1.7004 | 0.0010 | 1.2674 | 0.0896 |
| 64 | 13.8021 | 0.0000 | 7.0634 | 0.0000 | 18.4038 | 0.0000 | 6.9457 | 0.0000 | 11.1322 | 0.0000 | 5.3243 | 0.0000 | 11.8355 | 0.0000 | 6.3325 | 0.0000 |
| **1-Minute Data Resolution** | | | | | | | | | | | | | | | | |
| 1 | 0.4485 | 0.9958 | 0.4181 | 0.9978 | 0.7896 | 0.8055 | 2.1084 | 0.0002 | 0.6898 | 0.9475 | 1.0471 | 0.3872 | 0.7062 | 0.9502 | 1.7879 | 0.0004 |
| 2 | 1.1417 | 0.2967 | 1.4466 | 0.0874 | 1.6033 | 0.0033 | 0.7021 | 0.9593 | 1.1056 | 0.3347 | 1.1836 | 0.2562 | 1.5166 | 0.0085 | 0.7473 | 0.9249 |
| 3 | 1.4068 | 0.1989 | 0.7157 | 0.6588 | 1.6488 | 0.0040 | 1.5394 | 0.0116 | 1.1778 | 0.3056 | 3.4983 | 0.0003 | 1.1039 | 0.2767 | 1.4162 | 0.0215 |
| 4 | 1.1716 | 0.3192 | 0.3425 | 0.9144 | 2.6105 | 0.0003 | 1.0466 | 0.4038 | 0.6983 | 0.6511 | 0.7536 | 0.6066 | 1.3638 | 0.1415 | 1.4809 | 0.0890 |
| 5 | 1.6962 | 0.0000 | 1.1878 | 0.0985 | 5.7995 | 0.0000 | 5.2813 | 0.0000 | 1.6239 | 0.0388 | 1.4466 | 0.0895 | 1.1880 | 0.3049 | 0.5405 | 0.5825 |
| 6 | 2.8614 | 0.0221 | 2.7230 | 0.0279 | 0.7843 | 0.5025 | 3.0164 | 0.0287 | 0.3466 | 0.9999 | 0.1924 | 1.0000 | 0.4110 | 0.9970 | 0.4019 | 0.9975 |
| 7 | 2.2247 | 0.0000 | 1.7416 | 0.0001 | 7.5496 | 0.0000 | 3.9405 | 0.0000 | 1.0027 | 0.4742 | 0.8222 | 0.9004 | 4.6944 | 0.0000 | 2.1089 | 0.0000 |
| 8 | 6.1055 | 0.0000 | 5.0825 | 0.0000 | 6.3842 | 0.0000 | 3.3004 | 0.0000 | 2.1569 | 0.0956 | 0.4063 | 0.7487 | 2.1074 | 0.0828 | 1.7905 | 0.1339 |
| 9 | 2.9418 | 0.0073 | 1.3415 | 0.2351 | 4.5064 | 0.0013 | 0.1233 | 0.9741 | 1.4464 | 0.1377 | 2.4346 | 0.0038 | 5.1191 | 0.0000 | 7.2571 | 0.0000 |
| 10 | 3.6385 | 0.0000 | 1.3748 | 0.1513 | 3.7480 | 0.0001 | 3.1415 | 0.0006 | 5.2411 | 0.0000 | 1.5846 | 0.1146 | 3.8299 | 0.0009 | 3.9707 | 0.0006 |
| 11 | 10.5877 | 0.0000 | 2.9507 | 0.0000 | 8.2023 | 0.0000 | 2.7128 | 0.0000 | 5.2985 | 0.0000 | 2.2795 | 0.0001 | 4.2970 | 0.0000 | 2.0715 | 0.0001 |
| 12 | 2.8982 | 0.0000 | 3.2846 | 0.0000 | 1.7430 | 0.0005 | 6.1471 | 0.0000 | 1.7854 | 0.0030 | 2.4727 | 0.0000 | 1.1067 | 0.2970 | 1.7714 | 0.0020 |
| 13 | 7.0770 | 0.0000 | 16.5921 | 0.0000 | 1.3494 | 0.0266 | 1.1090 | 0.2475 | 7.8608 | 0.0000 | 12.7831 | 0.0000 | 0.9355 | 0.6520 | 1.4117 | 0.0065 |
| 14 | 1.0416 | 0.3846 | 1.8170 | 0.0000 | 1.0199 | 0.4354 | 3.7148 | 0.0000 | 1.1072 | 0.3337 | 4.2179 | 0.0000 | 2.6330 | 0.0008 | 6.8691 | 0.0000 |
| 15 | 1.1232 | 0.3050 | 1.6445 | 0.0228 | 0.4136 | 0.9981 | 0.3969 | 0.9987 | 1.1054 | 0.3256 | 1.8501 | 0.0062 | 0.5546 | 0.9765 | 0.4178 | 0.9979 |
| 16 | 2.5957 | 0.0000 | 2.7385 | 0.0000 | 2.9501 | 0.0000 | 1.9355 | 0.0000 | 2.9542 | 0.0000 | 3.5365 | 0.0000 | 2.3591 | 0.0000 | 2.6216 | 0.0000 |
| 17 | 2.6368 | 0.0102 | 52.7941 | 0.0000 | 5.9432 | 0.0000 | 40.2864 | 0.0000 | 2.6206 | 0.0000 | 8.3567 | 0.0000 | 2.0746 | 0.0000 | 5.0448 | 0.0000 |
| 18 | 3.8532 | 0.0000 | 3.4057 | 0.0002 | 3.2921 | 0.0003 | 1.9215 | 0.0380 | 1.1207 | 0.2193 | 1.0248 | 0.4192 | 1.1292 | 0.2050 | 0.9683 | 0.5594 |
| 19 | 2.8835 | 0.0014 | 4.2692 | 0.0000 | 0.6844 | 0.5616 | 43.1766 | 0.0000 | 2.9137 | 0.0013 | 0.8461 | 0.5840 | 2.3327 | 0.0724 | 21.4343 | 0.0000 |
| 20 | 3.1424 | 0.0000 | 7.5760 | 0.0000 | 3.1313 | 0.0000 | 2.9977 | 0.0000 | 4.3847 | 0.0000 | 8.3243 | 0.0000 | 3.2289 | 0.0000 | 3.8869 | 0.0000 |
| 21 | 5.7583 | 0.0000 | 2.4206 | 0.0339 | 0.6534 | 0.4190 | 1.6040 | 0.2055 | 2.5704 | 0.0253 | 1.2685 | 0.2750 | 0.0369 | 0.8477 | 0.3289 | 0.5664 |
| 22 | 2.3888 | 0.0000 | 2.2852 | 0.0000 | 2.1581 | 0.0000 | 2.7215 | 0.0000 | 5.0279 | 0.0000 | 4.4739 | 0.0000 | 2.6646 | 0.0031 | 3.4895 | 0.0001 |
| 23 | 0.7053 | 0.9842 | 1.5202 | 0.0012 | 1.8081 | 0.0000 | 2.4330 | 0.0000 | 0.7447 | 0.9576 | 2.0284 | 0.0000 | 1.7188 | 0.0001 | 1.6898 | 0.0001 |
| 24 | 0.9807 | 0.4965 | 0.8830 | 0.6491 | 0.8809 | 0.7074 | 0.8318 | 0.7919 | 1.9582 | 0.0686 | 2.4940 | 0.0210 | 1.6420 | 0.0896 | 1.0367 | 0.4097 |
| 25 | 10.2775 | 0.0000 | 104.8514 | 0.0000 | 4.2574 | 0.0000 | 29.7368 | 0.0000 | 23.7260 | 0.0000 | 35.9084 | 0.0000 | 3.7381 | 0.0000 | 14.0982 | 0.0000 |
| 26 | 1.3341 | 0.1914 | 9.5911 | 0.0000 | 1.5913 | 0.0061 | 2.6320 | 0.0000 | 2.4535 | 0.0010 | 5.2368 | 0.0000 | 1.6563 | 0.0009 | 2.7309 | 0.0000 |
| 27 | 1.8199 | 0.0635 | 1.0218 | 0.4219 | 1.6710 | 0.0861 | 1.7418 | 0.0703 | 0.5148 | 0.8587 | 1.5407 | 0.1527 | 0.7882 | 0.6399 | 1.2407 | 0.2839 |
| 28 | 24.6241 | 0.0000 | 0.0385 | 0.8444 | 22.6337 | 0.0000 | 0.4242 | 0.5149 | 4.3310 | 0.0132 | 0.1999 | 0.8188 | 6.5064 | 0.0015 | 1.9026 | 0.1492 |
| 29 | 0.8385 | 0.5913 | 3.1225 | 0.0006 | 1.2744 | 0.2388 | 1.5793 | 0.1062 | 0.0236 | 0.8778 | 0.2999 | 0.5840 | 0.0630 | 0.8018 | 0.0053 | 0.9419 |
| 30 | 2.1188 | 0.1206 | 19.7793 | 0.0000 | 0.5641 | 0.8441 | 5.2979 | 0.0000 | 3.3682 | 0.0348 | 12.1066 | 0.0000 | 0.4476 | 0.9230 | 3.4597 | 0.0002 |
| 31 | 0.6319 | 0.9821 | 0.3649 | 1.0000 | 1.3629 | 0.2267 | 0.4136 | 0.8703 | 0.8024 | 0.8205 | 0.4159 | 0.9998 | 1.5827 | 0.1623 | 0.1196 | 0.9881 |
| 32 | 8.9655 | 0.0000 | 10.6208 | 0.0000 | 1.2984 | 0.2250 | 3.1952 | 0.0004 | 6.4126 | 0.0000 | 4.2354 | 0.0000 | 0.8889 | 0.6020 | 1.1475 | 0.2919 |
| 33 | 0.6917 | 0.9884 | 2.3024 | 0.0000 | 6.8415 | 0.0000 | 1.3685 | 0.0089 | 1.0014 | 0.4718 | 1.9469 | 0.0000 | 1.7860 | 0.0002 | 0.4044 | 1.0000 |
| 34 | 4.5096 | 0.0343 | 5.7853 | 0.0166 | 0.9588 | 0.4529 | 4.0807 | 0.0005 | 8.6304 | 0.0002 | 1.5788 | 0.2074 | 1.3748 | 0.1750 | 2.0454 | 0.0196 |
| 35 | 2.0576 | 0.0000 | 0.5825 | 0.9990 | 21.4685 | 0.0000 | 4.5448 | 0.0000 | 2.2748 | 0.0000 | 1.1886 | 0.1316 | 6.9434 | 0.0000 | 2.9591 | 0.0016 |
| 36 | 13.7245 | 0.0000 | 0.3615 | 0.9996 | 2.5520 | 0.0025 | 1.3490 | 0.1845 | 4.1890 | 0.0000 | 0.3459 | 0.9998 | 3.5028 | 0.0000 | 1.9528 | 0.0252 |
| 37 | 6.4504 | 0.0000 | 1.0600 | 0.3875 | 3.1116 | 0.0002 | 4.8671 | 0.0000 | 41.0066 | 0.0000 | 1.4651 | 0.2220 | 24.6883 | 0.0000 | 4.2857 | 0.0138 |
| 38 | 12.4256 | 0.0000 | 44.6761 | 0.0000 | 9.2997 | 0.0000 | 53.5572 | 0.0000 | 13.3136 | 0.0000 | 11.8820 | 0.0000 | 14.4487 | 0.0000 | 16.5138 | 0.0000 |
| 39 | 4.8540 | 0.0001 | 4.5330 | 0.0001 | 2.4239 | 0.0072 | 0.5626 | 0.8455 | 4.3749 | 0.0002 | 3.7956 | 0.0009 | 2.7206 | 0.0025 | 0.7425 | 0.6848 |
| 40 | 0.2421 | 0.9996 | 2.7632 | 0.0001 | 1.1673 | 0.3207 | 3.7802 | 0.0101 | 0.0991 | 0.9965 | 0.2380 | 0.9640 | 0.5665 | 0.4518 | 0.0004 | 0.9849 |
| 41 | 34.5619 | 0.0000 | 31.2232 | 0.0000 | 1.2932 | 0.1532 | 3.6530 | 0.0000 | 14.9803 | 0.0000 | 28.1496 | 0.0000 | 2.3106 | 0.0000 | 2.4776 | 0.0000 |
| 42 | 0.4572 | 0.9987 | 0.5848 | 0.9831 | 2.5340 | 0.0000 | 5.0464 | 0.0000 | 0.6568 | 0.9533 | 0.4257 | 0.9994 | 1.1803 | 0.2022 | 0.6516 | 0.9563 |
| 43 | 1.2204 | 0.2925 | 2.9812 | 0.0067 | 5.4939 | 0.0000 | 2.1674 | 0.0342 | 0.6639 | 0.6789 | 1.1053 | 0.3566 | 5.0343 | 0.0000 | 1.5245 | 0.1541 |
| 44 | 9.6465 | 0.0000 | 3.2550 | 0.0034 | 3.9023 | 0.0036 | 1.0533 | 0.3780 | 9.4351 | 0.0000 | 4.9312 | 0.0000 | 4.3291 | 0.0017 | 0.5367 | 0.7088 |
| 45 | 2.8281 | 0.0025 | 1.1535 | 0.3206 | 1.6061 | 0.1546 | 1.0963 | 0.3600 | 5.8670 | 0.0000 | 1.7501 | 0.0724 | 4.6223 | 0.0003 | 2.7789 | 0.0163 |
| 46 | 2.0138 | 0.0281 | 1.7642 | 0.0614 | 1.8450 | 0.0033 | 1.8107 | 0.0043 | 8.7855 | 0.0000 | 0.8772 | 0.5539 | 2.0098 | 0.0009 | 4.0430 | 0.0000 |
| 47 | 1.4653 | 0.0071 | 14.7504 | 0.0000 | 1.5694 | 0.0064 | 2.9222 | 0.0000 | 36.0355 | 0.0000 | 19.9706 | 0.0000 | 17.5586 | 0.0000 | 11.8029 | 0.0000 |
| 48 | 1.9718 | 0.0003 | 1.1404 | 0.2509 | 1.3223 | 0.0230 | 1.6372 | 0.0002 | 2.6973 | 0.0000 | 0.9616 | 0.5288 | 1.7778 | 0.0001 | 1.8499 | 0.0000 |
| 49 | 1.3368 | 0.0297 | 3.0192 | 0.0000 | 4.2839 | 0.0000 | 13.0985 | 0.0000 | NA | NA | NA | NA | 0.5169 | 0.7936 | 1.7517 | 0.4135 |
| 50 | 1.7640 | 0.0615 | 0.9295 | 0.5044 | 3.1410 | 0.0044 | 4.2623 | 0.0003 | 1.7242 | 0.0693 | 0.7058 | 0.7200 | 3.4710 | 0.0020 | 2.3006 | 0.0320 |
| 51 | 0.9907 | 0.5056 | 1.6405 | 0.0002 | 0.7545 | 0.7557 | 2.1351 | 0.0035 | 1.2295 | 0.0956 | 1.2537 | 0.0764 | 0.9131 | 0.5438 | 3.7973 | 0.0000 |
| 52 | 5.2944 | 0.0012 | 11.2832 | 0.0000 | 0.8738 | 0.6267 | 3.5759 | 0.0000 | 2.6531 | 0.0211 | 2.2588 | 0.0460 | 0.9328 | 0.5820 | 1.9530 | 0.0007 |
| 53 | 1.8239 | 0.0137 | 4.1019 | 0.0000 | 4.6878 | 0.0000 | 7.3285 | 0.0000 | 1.9395 | 0.0072 | 3.5161 | 0.0000 | 5.0224 | 0.0000 | 6.9418 | 0.0000 |
| 54 | 9.0358 | 0.0000 | 20.2092 | 0.0000 | 1.6579 | 0.0229 | 16.4390 | 0.0000 | 8.2999 | 0.0000 | 11.4145 | 0.0000 | 1.8377 | 0.0052 | 4.6462 | 0.0000 |
| 55 | 3.5173 | 0.0317 | 1.0207 | 0.3624 | 0.4711 | 0.7569 | 1.2380 | 0.2965 | 5.5110 | 0.0199 | 1.0031 | 0.3179 | 0.8041 | 0.4491 | 1.6693 | 0.1912 |
| 56 | 1.4204 | 0.1822 | 2.1574 | 0.0277 | 9.2000 | 0.0024 | 62.0221 | 0.0000 | 1.1260 | 0.3418 | 2.3509 | 0.0160 | 14.3149 | 0.0002 | 13.1620 | 0.0003 |
| 57 | 0.9919 | 0.4970 | 1.4314 | 0.0110 | 0.5265 | 0.9993 | 1.2566 | 0.0837 | 0.7734 | 0.7483 | 1.6824 | 0.0297 | 1.3324 | 0.1573 | 14.3251 | 0.0000 |
| 58 | 1.0661 | 0.3844 | 4.9084 | 0.0000 | 2.3281 | 0.0171 | 3.8470 | 0.0002 | 1.7648 | 0.0696 | 6.0648 | 0.0000 | 1.6126 | 0.1155 | 3.9417 | 0.0001 |
| 59 | 1.3464 | 0.0562 | 0.8887 | 0.6902 | 1.5547 | 0.0033 | 1.5523 | 0.0034 | 0.8177 | 0.8269 | 0.5030 | 0.9991 | 1.2583 | 0.0705 | 0.9808 | 0.5242 |
| 60 | 3.3355 | 0.0679 | 10.5904 | 0.0012 | 2.0944 | 0.0219 | 0.5084 | 0.8853 | 1.5247 | 0.1432 | 1.8804 | 0.0588 | 1.3806 | 0.0154 | 0.7432 | 0.9566 |
| 61 | 2.1834 | 0.0041 | 2.3805 | 0.0015 | 3.5120 | 0.0000 | 3.5380 | 0.0000 | 1.0025 | 0.4729 | 1.2405 | 0.0726 | 0.9208 | 0.6894 | 1.1482 | 0.1606 |
| 62 | 1.0412 | 0.4081 | 1.4391 | 0.1022 | 2.1846 | 0.0004 | 3.1286 | 0.0000 | 3.4521 | 0.0021 | 2.0300 | 0.0582 | 1.8545 | 0.0539 | 6.4118 | 0.0000 |
| 63 | 1.3833 | 0.0272 | 1.6871 | 0.0008 | 1.5303 | 0.0109 | 1.7856 | 0.0007 | 3.3896 | 0.0002 | 3.2802 | 0.0003 | 4.3853 | 0.0000 | 3.7052 | 0.0003 |
| 64 | 30.9144 | 0.0000 | 46.4057 | 0.0000 | 13.8819 | 0.0000 | 20.7332 | 0.0000 | 31.6446 | 0.0000 | 57.1176 | 0.0000 | 15.6434 | 0.0000 | 27.2113 | 0.0000 |
| **5-Minute Data Resolution** | | | | | | | | | | | | | | | | |
| 1 | 0.3186 | 0.9996 | 0.5234 | 0.9758 | 2.5132 | 0.0009 | 0.7532 | 0.7529 | 1.1712 | 0.2492 | 0.8160 | 0.7996 | 2.0184 | 0.0012 | 1.1723 | 0.2438 |
| 2 | 1.1375 | 0.2872 | 0.1401 | 0.7085 | 1.6302 | 0.1852 | 6.7590 | 0.0003 | 1.1624 | 0.3145 | 1.1117 | 0.3307 | 0.2919 | 0.9399 | 1.0180 | 0.4165 |
| 3 | 1.6659 | 0.0446 | 0.9643 | 0.5112 | 1.2783 | 0.2271 | 1.4062 | 0.1567 | 1.5498 | 0.1121 | 0.5143 | 0.9033 | 1.5680 | 0.1379 | 0.4748 | 0.8727 |
| 4 | 0.6216 | 0.5383 | 1.6886 | 0.1878 | 1.2423 | 0.2733 | 2.4326 | 0.0130 | 0.2531 | 0.9075 | 1.0026 | 0.4078 | 0.9403 | 0.5323 | 0.5159 | 0.9466 |
| 5 | 1.6117 | 0.0031 | 1.5502 | 0.0062 | 0.9365 | 0.6094 | 1.4894 | 0.0117 | 0.5250 | 0.8162 | 0.3251 | 0.9429 | 2.0074 | 0.0510 | 15.9578 | 0.0000 |
| 6 | 1.3036 | 0.1457 | 0.5027 | 0.9807 | 0.3281 | 0.9926 | 0.3798 | 0.9842 | 0.7701 | 0.7117 | 0.7037 | 0.7822 | 0.5564 | 0.8331 | 0.6315 | 0.7707 |
| 7 | 0.5822 | 0.9143 | 1.1015 | 0.3444 | 3.6012 | 0.0000 | 6.3779 | 0.0000 | 1.3929 | 0.1487 | 0.3065 | 0.9933 | 0.8789 | 0.7378 | 5.5302 | 0.0000 |
| 8 | 1.9780 | 0.0045 | 0.9577 | 0.5317 | 4.9491 | 0.0000 | 5.1629 | 0.0000 | 0.3408 | 0.9151 | 2.0626 | 0.1541 | 0.1421 | 0.8683 | 0.8493 | 0.4402 |
| 9 | 0.8789 | 0.6532 | 0.3465 | 0.9996 | 0.7834 | 0.7201 | 0.5449 | 0.9355 | 1.3149 | 0.1898 | 0.3697 | 0.9857 | 2.4331 | 0.0106 | 0.7443 | 0.6682 |
| 10 | 0.9541 | 0.4332 | 2.2506 | 0.0640 | 0.4713 | 0.8931 | 0.5666 | 0.8239 | 1.1310 | 0.3123 | 0.9303 | 0.5609 | 0.8825 | 0.6922 | 1.0530 | 0.4013 |
| 11 | 7.2925 | 0.0000 | 5.5767 | 0.0000 | 16.4102 | 0.0000 | 17.4014 | 0.0000 | 2.8115 | 0.0000 | 1.7562 | 0.0056 | 2.1113 | 0.0006 | 3.0582 | 0.0000 |
| 12 | 4.8923 | 0.0000 | 1.3745 | 0.0927 | 3.5524 | 0.0000 | 2.0199 | 0.0001 | 2.9254 | 0.0001 | 1.0871 | 0.3619 | 2.0699 | 0.0009 | 1.2660 | 0.1608 |
| 13 | 8.1577 | 0.0000 | 5.7774 | 0.0000 | 9.0950 | 0.0000 | 12.2591 | 0.0000 | 2.5189 | 0.0000 | 1.5736 | 0.0292 | 3.1263 | 0.0002 | 2.9128 | 0.0005 |
| 14 | 1.5798 | 0.0247 | 1.4675 | 0.0502 | 5.4526 | 0.0000 | 1.0644 | 0.3867 | 0.9874 | 0.4894 | 1.3148 | 0.1198 | 4.2133 | 0.0000 | 0.7445 | 0.7485 |
| 15 | 3.4121 | 0.0000 | 2.5752 | 0.0012 | 0.1006 | 0.9983 | 0.7315 | 0.6453 | 2.3607 | 0.0007 | 1.1796 | 0.2642 | 0.2291 | 0.9935 | 0.5064 | 0.8864 |
| 16 | 0.5289 | 0.9999 | 0.8712 | 0.8064 | 0.5594 | 0.9998 | 0.6527 | 0.9960 | 1.1726 | 0.1959 | 1.1204 | 0.2659 | 1.1385 | 0.2401 | 1.0935 | 0.3075 |
| 17 | 1.2863 | 0.0655 | 1.1329 | 0.2245 | 1.3514 | 0.0353 | 1.1209 | 0.2434 | 1.3380 | 0.0469 | 1.2052 | 0.1408 | 1.2209 | 0.1250 | 1.2049 | 0.1411 |
| 18 | 1.3093 | 0.2581 | 3.0263 | 0.0104 | 0.6193 | 0.5386 | 8.4666 | 0.0002 | 1.2399 | 0.2887 | 2.6600 | 0.0216 | 0.7067 | 0.4936 | 8.7580 | 0.0002 |
| 19 | 3.6145 | 0.0136 | 3.5138 | 0.0156 | 1.8490 | 0.0283 | 1.1840 | 0.2834 | 2.7901 | 0.0958 | 3.8277 | 0.0513 | 3.2115 | 0.0077 | 1.8420 | 0.1044 |
| 20 | 0.6393 | 0.4242 | 18.9482 | 0.0000 | 1.1658 | 0.3122 | 28.9035 | 0.0000 | 0.6413 | 0.6682 | 4.6380 | 0.0004 | 2.2931 | 0.0118 | 5.6930 | 0.0000 |
| 21 | 0.8197 | 0.6763 | 1.1268 | 0.3267 | 1.2377 | 0.2439 | 1.2439 | 0.2395 | 0.9076 | 0.6088 | 1.3274 | 0.1314 | 1.0620 | 0.3897 | 0.7837 | 0.7602 |
| 22 | 1.3574 | 0.1834 | 2.3262 | 0.0068 | 1.4636 | 0.0656 | 1.5781 | 0.0351 | 1.6655 | 0.1569 | 1.8335 | 0.1213 | 1.2516 | 0.2615 | 0.9122 | 0.5143 |
| 23 | 1.3291 | 0.0518 | 0.6957 | 0.9616 | 1.3541 | 0.0485 | 1.4225 | 0.0266 | 1.9968 | 0.0306 | 0.7289 | 0.6977 | 0.6679 | 0.7385 | 3.1726 | 0.0009 |
| 24 | 1.8427 | 0.0224 | 0.8501 | 0.6396 | 0.5621 | 0.8710 | 1.6312 | 0.0842 | 1.8009 | 0.0268 | 0.9529 | 0.5158 | 0.6347 | 0.8115 | 1.8974 | 0.0357 |
| 25 | 1.1386 | 0.2741 | 1.0132 | 0.4557 | 0.7060 | 0.9065 | 0.9826 | 0.5059 | 0.5682 | 0.9677 | 0.7946 | 0.7715 | 0.6783 | 0.8992 | 1.0436 | 0.4093 |
| 26 | 5.3871 | 0.0000 | 1.1984 | 0.2884 | 1.3062 | 0.2228 | 1.1147 | 0.3480 | 3.5221 | 0.0000 | 0.8290 | 0.6205 | 1.1523 | 0.3143 | 0.9739 | 0.4723 |
| 27 | 0.1615 | 0.8512 | 1.2284 | 0.2993 | 0.1081 | 0.8977 | 0.6395 | 0.5307 | NA | NA | NA | NA | NA | NA | NA | NA |
| 28 | 1.1042 | 0.3574 | 2.9534 | 0.0044 | 0.2376 | 1.0000 | 1.3513 | 0.1031 | 9.5175 | 0.0021 | 10.2887 | 0.0014 | 0.3246 | 0.8616 | 3.8680 | 0.0039 |
| 29 | 12.3837 | 0.0005 | 0.0216 | 0.8831 | 3.0160 | 0.0064 | 4.9160 | 0.0001 | 1.5773 | 0.1640 | 1.1293 | 0.3431 | 1.1077 | 0.3175 | 0.2882 | 0.9999 |
| 30 | 1.6731 | 0.0740 | 1.5467 | 0.1091 | 6.4021 | 0.0000 | 2.6971 | 0.0149 | 1.2276 | 0.2927 | 2.2612 | 0.0384 | 10.0998 | 0.0000 | 2.0834 | 0.1029 |
| 31 | 1.9957 | 0.0500 | 3.3239 | 0.0015 | 5.6280 | 0.0043 | 4.7375 | 0.0099 | 1.6641 | 0.1109 | 3.5546 | 0.0008 | 9.4947 | 0.0001 | 5.3301 | 0.0056 |
| 32 | 1.4240 | 0.0672 | 1.5270 | 0.0362 | 0.9372 | 0.5867 | 0.5430 | 0.9923 | 1.4743 | 0.0825 | 1.9545 | 0.0075 | 1.0288 | 0.4250 | 0.5406 | 0.9757 |
| 33 | 4.4276 | 0.0000 | 0.6773 | 0.9592 | 3.2409 | 0.0000 | 7.1975 | 0.0000 | 3.3494 | 0.0002 | 0.7257 | 0.7008 | 8.2123 | 0.0000 | 11.0426 | 0.0000 |
| 34 | 1.0881 | 0.3690 | 1.9503 | 0.1114 | 0.1923 | 0.8255 | 0.1128 | 0.8935 | 0.9577 | 0.3883 | 0.9428 | 0.3939 | 0.2374 | 0.6274 | 1.1407 | 0.2887 |
| 35 | 5.8028 | 0.0000 | 5.4640 | 0.0000 | 4.3212 | 0.0000 | 3.1570 | 0.0005 | 6.8863 | 0.0000 | 4.7659 | 0.0000 | 4.8999 | 0.0000 | 1.6646 | 0.0832 |
| 36 | 1.6196 | 0.1021 | 12.2203 | 0.0000 | 0.3315 | 0.8936 | 0.7422 | 0.5926 | 3.3016 | 0.0005 | 2.9893 | 0.0015 | 1.2414 | 0.2904 | 2.3822 | 0.0392 |
| 37 | 1.3170 | 0.1227 | 1.4370 | 0.0637 | 1.4061 | 0.1381 | 2.0217 | 0.0122 | 1.4040 | 0.0622 | 1.5967 | 0.0165 | 1.5097 | 0.0802 | 2.3668 | 0.0012 |
| 38 | 7.6036 | 0.0000 | 3.3329 | 0.0000 | 6.8487 | 0.0000 | 1.3193 | 0.1510 | 5.4046 | 0.0000 | 4.2123 | 0.0001 | 5.6643 | 0.0000 | 2.3828 | 0.0048 |
| 39 | 3.5377 | 0.0144 | 1.9185 | 0.1251 | 0.2286 | 0.6327 | 1.0445 | 0.3072 | 3.9806 | 0.0079 | 1.9058 | 0.1272 | 0.2235 | 0.6366 | 2.0081 | 0.1570 |
| 40 | 1.3276 | 0.1040 | 1.1215 | 0.2945 | 2.6803 | 0.0098 | 0.9141 | 0.4950 | 0.9519 | 0.5337 | 1.4615 | 0.0800 | 0.5429 | 0.7437 | 1.2045 | 0.3073 |
| 41 | 1.1708 | 0.1727 | 1.5767 | 0.0031 | 6.2060 | 0.0000 | 3.4686 | 0.0011 | 1.9065 | 0.0471 | 0.8971 | 0.5270 | 6.9953 | 0.0082 | 9.7092 | 0.0019 |
| 42 | 1.0718 | 0.3225 | 4.9623 | 0.0000 | 0.4121 | 0.9915 | 0.6014 | 0.9205 | 1.7392 | 0.0000 | 4.2709 | 0.0000 | 0.8585 | 0.6748 | 0.8884 | 0.6307 |
| 43 | 0.1040 | 0.7472 | 0.4263 | 0.5141 | 0.3161 | 0.7291 | 13.5857 | 0.0000 | 0.0030 | 0.9563 | 0.6578 | 0.4178 | 1.1605 | 0.3141 | 6.8097 | 0.0012 |
| 44 | 0.6242 | 0.7773 | 2.3313 | 0.0131 | 1.7781 | 0.1141 | 4.3858 | 0.0006 | 0.4570 | 0.9927 | 1.0052 | 0.4570 | 1.4201 | 0.1288 | 1.5062 | 0.0945 |
| 45 | 7.5899 | 0.0000 | 4.6909 | 0.0000 | 7.1172 | 0.0000 | 2.6881 | 0.0041 | 5.1021 | 0.0000 | 1.5285 | 0.1319 | 5.0060 | 0.0000 | 0.9594 | 0.4719 |
| 46 | 2.1039 | 0.0000 | 1.7493 | 0.0002 | 9.9467 | 0.0000 | 3.9263 | 0.0003 | 3.3677 | 0.0000 | 1.3643 | 0.0145 | 21.5528 | 0.0000 | 1.8996 | 0.0478 |
| 47 | 3.3262 | 0.0000 | 1.7980 | 0.0003 | 5.9688 | 0.0000 | 2.8612 | 0.0000 | 2.4916 | 0.0000 | 3.0083 | 0.0000 | 5.0313 | 0.0000 | 2.9434 | 0.0000 |
| 48 | 2.5038 | 0.0206 | 5.8372 | 0.0000 | 4.5433 | 0.0000 | 5.8941 | 0.0000 | 0.8248 | 0.5506 | 4.3121 | 0.0003 | 2.3421 | 0.0169 | 9.4696 | 0.0000 |
| 49 | 1.0819 | 0.3406 | 3.6716 | 0.0269 | 5.4876 | 0.0003 | 3.2333 | 0.0132 | NA | NA | NA | NA | NA | NA | NA | NA |
| 50 | 0.9253 | 0.5670 | 1.2349 | 0.1985 | 4.4494 | 0.0000 | 1.6365 | 0.1093 | 0.8962 | 0.5682 | 2.0513 | 0.0097 | 5.4952 | 0.0000 | 1.0164 | 0.4062 |
| 51 | 52.5487 | 0.0000 | 3.5191 | 0.0610 | 15.7961 | 0.0001 | 5.0502 | 0.0249 | 10.3145 | 0.0014 | 0.3252 | 0.5687 | 9.6764 | 0.0019 | 0.1730 | 0.6776 |
| 52 | 2.0528 | 0.0004 | 1.1803 | 0.2209 | 1.9411 | 0.0368 | 5.6433 | 0.0000 | 2.1627 | 0.0019 | 0.5457 | 0.9519 | 3.1193 | 0.0050 | 3.8093 | 0.0009 |
| 53 | 28.9647 | 0.0000 | 6.8466 | 0.0090 | 5.0054 | 0.0000 | 0.6854 | 0.7389 | 8.8960 | 0.0000 | 3.1982 | 0.0227 | 2.2474 | 0.0001 | 1.3594 | 0.0941 |
| 54 | 7.3235 | 0.0000 | 3.0292 | 0.0000 | 8.0505 | 0.0000 | 1.4185 | 0.1369 | 4.8379 | 0.0000 | 2.2878 | 0.0008 | 4.3296 | 0.0000 | 1.2626 | 0.2241 |
| 55 | 5.0616 | 0.0125 | 8.2669 | 0.0013 | 1.4471 | 0.2480 | 3.4906 | 0.0214 | 10.1422 | 0.0031 | 10.6052 | 0.0026 | 1.9522 | 0.1590 | 3.7975 | 0.0335 |
| 56 | 3.6776 | 0.0055 | 1.9009 | 0.1079 | 2.0529 | 0.0010 | 0.9883 | 0.4828 | 0.0012 | 0.9724 | 3.1135 | 0.0779 | 9.0563 | 0.0000 | 1.9603 | 0.0573 |
| 57 | 2.5745 | 0.0062 | 2.4637 | 0.0088 | 2.0690 | 0.0296 | 3.9064 | 0.0001 | 1.4243 | 0.0626 | 0.7972 | 0.7912 | 4.7276 | 0.0000 | 1.2727 | 0.1464 |
| 58 | 0.6816 | 0.7425 | 1.7690 | 0.0615 | 0.6849 | 0.5043 | 3.6236 | 0.0269 | 0.7120 | 0.7139 | 2.0469 | 0.0258 | 1.8437 | 0.1586 | 3.0874 | 0.0459 |
| 59 | 1.5296 | 0.1330 | 2.9739 | 0.0017 | 1.2305 | 0.2169 | 1.8028 | 0.0151 | 0.1793 | 0.9105 | 3.4287 | 0.0167 | 1.2578 | 0.2685 | 2.3299 | 0.0234 |
| 60 | 2.6697 | 0.0000 | 0.9585 | 0.5319 | 2.5147 | 0.0001 | 2.4888 | 0.0001 | 1.9434 | 0.0025 | 0.9202 | 0.5907 | 1.8351 | 0.0099 | 2.1766 | 0.0012 |
| 61 | 0.5532 | 0.9991 | 1.2399 | 0.0878 | 0.7020 | 0.9839 | 1.2772 | 0.0447 | 0.7221 | 0.9613 | 0.8729 | 0.7669 | 0.8745 | 0.7912 | 1.0170 | 0.4383 |
| 62 | 0.8831 | 0.7439 | 0.8053 | 0.8779 | 1.6206 | 0.0001 | 0.8841 | 0.7852 | 0.9161 | 0.6631 | 0.9263 | 0.6410 | 1.5607 | 0.0007 | 0.8863 | 0.7675 |
| 63 | 0.9779 | 0.5306 | 0.9751 | 0.5370 | 1.0543 | 0.3796 | 1.3319 | 0.0795 | 1.1214 | 0.2387 | 0.8836 | 0.7391 | 1.2727 | 0.1175 | 1.0422 | 0.3997 |
| 64 | 5.5152 | 0.0000 | 2.6686 | 0.0000 | 3.2598 | 0.0000 | 1.6206 | 0.0001 | 5.7361 | 0.0000 | 2.6552 | 0.0000 | 3.2161 | 0.0000 | 1.7614 | 0.0000 |
| *ABP, arterial blood pressure; CPP, cerebral perfusion pressure; rSO_2_, regional cerebral oxygen saturation; TBI-GLR, traumatic brain injury patient group without bifrontal lobe pathology.* | | | | | | | | | | | | | | | | |

File S7d: Granger Causality Test Results for TBI-GL Population in 10-Second, 1-Minute, and 5-Minute Data Resolutions

| **Patient** | **ABP ® rSO_2__L (F Statistic)** | **ABP ® rSO_2__L (P-Value)** | **rSO_2__L ® ABP (F Statistic)** | **rSO_2__L ® ABP (P-Value)** | **ABP ® rSO_2__R (F Statistic)** | **ABP ® rSO_2__R (P-Value)** | **rSO_2__R ® ABP (F Statistic)** | **rSO_2__R ® ABP (P-Value)** | **CPP ® rSO_2__L (F Statistic)** | **CPP ® rSO_2__L (P-Value)** | **rSO_2__L ® CPP (F Statistic)** | **rSO_2__L ® CPP (P-Value)** | **CPP ® rSO_2__R (F Statistic)** | **CPP ® rSO_2__R (P-Value)** | **rSO_2__R ® CPP (F Statistic)** | **rSO_2__R ® CPP (P-Value)** |
| --- | --- | --- | --- | --- | --- | --- | --- | --- | --- | --- | --- | --- | --- | --- | --- | --- |
| **10-Second Data Resolution** | | | | | | | | | | | | | | | | |
| 1 | 15.3368 | 0.0000 | 1.9177 | 0.0000 | 1.1503 | 0.1564 | 0.9441 | 0.6302 | 11.0525 | 0.0000 | 2.3455 | 0.0000 | 0.9969 | 0.4871 | 0.8304 | 0.8628 |
| 2 | 124.6787 | 0.0000 | 5.9146 | 0.0000 | 1218.0554 | 0.0000 | 67.0737 | 0.0000 | 7.1498 | 0.0000 | 1.2406 | 0.0997 | 189.8054 | 0.0000 | 11.5655 | 0.0000 |
| 3 | 4.0416 | 0.0000 | 1.3792 | 0.1023 | 15.3882 | 0.0000 | 4.2461 | 0.0000 | 2.1429 | 0.0003 | 1.0897 | 0.3363 | 5.9645 | 0.0000 | 2.5830 | 0.0001 |
| 4 | 4.9237 | 0.0000 | 2.1275 | 0.0024 | 4.9713 | 0.0000 | 4.4435 | 0.0000 | 9.7420 | 0.0000 | 2.1667 | 0.0029 | 2.3342 | 0.0000 | 1.5265 | 0.0047 |
| 5 | 82.6038 | 0.0000 | 61.8599 | 0.0000 | 19.3466 | 0.0000 | 15.2390 | 0.0000 | 14.1550 | 0.0000 | 4.6357 | 0.0000 | 2.6055 | 0.0000 | 1.8635 | 0.0003 |
| 6 | 0.7503 | 0.6092 | 0.3363 | 0.9180 | 0.1978 | 0.9945 | 0.1245 | 0.9991 | 1.8183 | 0.0086 | 2.4948 | 0.0001 | 0.4275 | 0.9989 | 0.4317 | 0.9988 |
| 7 | 12.1400 | 0.0000 | 0.2383 | 1.0000 | 0.7171 | 0.9557 | 0.4483 | 1.0000 | 2.2916 | 0.0001 | 0.5817 | 0.9666 | 4.4921 | 0.0000 | 0.6449 | 0.9202 |
| 8 | 16.8170 | 0.0000 | 5.6806 | 0.0000 | 0.9280 | 0.6616 | 0.8109 | 0.8915 | 18.1920 | 0.0000 | 5.7601 | 0.0000 | 1.0375 | 0.3907 | 0.7523 | 0.9421 |
| 9 | 63.9020 | 0.0000 | 117.6012 | 0.0000 | 135.5614 | 0.0000 | 65.3600 | 0.0000 | 124.7278 | 0.0000 | 42.1404 | 0.0000 | 452.1420 | 0.0000 | 122.8223 | 0.0000 |
| 10 | 3.2926 | 0.0000 | 3.5250 | 0.0000 | 3.2133 | 0.0000 | 1.8192 | 0.0000 | 3.9940 | 0.0000 | 4.1664 | 0.0000 | 2.1671 | 0.0000 | 1.3821 | 0.0070 |
| 11 | 10.6900 | 0.0000 | 1.4488 | 0.0331 | 32.8330 | 0.0000 | 7.2309 | 0.0000 | 6.8115 | 0.0000 | 1.3667 | 0.0467 | 18.1679 | 0.0000 | 3.4538 | 0.0021 |
| 12 | 11.5601 | 0.0000 | 2.9665 | 0.0000 | 0.9564 | 0.5434 | 0.4316 | 0.9988 | 6.6634 | 0.0000 | 2.3931 | 0.0000 | 0.8597 | 0.7074 | 0.3397 | 0.9999 |
| 13 | 1.5632 | 0.0016 | 1.5988 | 0.0009 | 2.6061 | 0.0000 | 2.4003 | 0.0000 | 0.7630 | 0.9540 | 1.1553 | 0.1490 | 1.8855 | 0.0000 | 1.5255 | 0.0006 |
| 14 | 2.6949 | 0.0000 | 0.7597 | 0.9459 | 1.7918 | 0.0000 | 1.6106 | 0.0005 | 2.3712 | 0.0000 | 0.7853 | 0.7915 | 1.8364 | 0.0035 | 1.1176 | 0.3003 |
| 15 | 3.1333 | 0.0000 | 0.6277 | 0.9987 | 2.7216 | 0.0000 | 0.9779 | 0.5397 | 2.5265 | 0.0000 | 0.5943 | 0.9996 | 2.4204 | 0.0000 | 0.7431 | 0.9675 |
| **1-Minute Data Resolution** | | | | | | | | | | | | | | | | |
| 1 | 3.4015 | 0.0000 | 3.5003 | 0.0000 | 2.3759 | 0.0000 | 1.5417 | 0.0298 | 4.3678 | 0.0000 | 3.4977 | 0.0000 | 2.2295 | 0.0001 | 1.5309 | 0.0320 |
| 2 | 219.6137 | 0.0000 | 51.0497 | 0.0000 | 54.9527 | 0.0000 | 44.8553 | 0.0000 | 10.5603 | 0.0000 | 2.7493 | 0.0050 | 2.1246 | 0.0011 | 2.4501 | 0.0001 |
| 3 | 0.8108 | 0.7770 | 0.6058 | 0.9673 | 0.9370 | 0.5601 | 1.3226 | 0.1202 | 0.9030 | 0.6323 | 0.7829 | 0.8153 | 0.9110 | 0.5999 | 0.6555 | 0.9160 |
| 4 | 0.9328 | 0.5666 | 0.9826 | 0.4913 | 12.0742 | 0.0000 | 3.0760 | 0.0032 | 1.9210 | 0.0283 | 1.8662 | 0.0344 | 4.3464 | 0.0047 | 2.3913 | 0.0671 |
| 5 | 1.4831 | 0.2234 | 35.5170 | 0.0000 | 53.3570 | 0.0000 | 46.7515 | 0.0000 | 0.0679 | 0.7945 | 47.8959 | 0.0000 | 52.2036 | 0.0000 | 69.1691 | 0.0000 |
| 6 | 1.3681 | 0.1252 | 84.8126 | 0.0000 | 2.5696 | 0.0532 | 0.3642 | 0.7789 | 0.2983 | 0.9546 | 0.7624 | 0.6191 | 0.3425 | 0.5586 | 0.0505 | 0.8223 |
| 7 | 18.8107 | 0.0000 | 282.8606 | 0.0000 | 0.2997 | 0.9814 | 0.2036 | 0.9960 | 0.4411 | 0.5068 | 0.5118 | 0.4745 | 7.2542 | 0.0000 | 0.3779 | 0.9565 |
| 8 | 12.0302 | 0.0000 | 3.9424 | 0.0000 | 0.4263 | 1.0000 | 1.6083 | 0.0005 | 11.3565 | 0.0000 | 3.4634 | 0.0000 | 0.4189 | 1.0000 | 2.0751 | 0.0000 |
| 9 | 67.3090 | 0.0000 | 381.6258 | 0.0000 | 63.9815 | 0.0000 | 318.1004 | 0.0000 | 19.5049 | 0.0000 | 131.6198 | 0.0000 | 16.9844 | 0.0000 | 117.7748 | 0.0000 |
| 10 | 1.7945 | 0.0263 | 1.8203 | 0.0234 | 1.5534 | 0.0416 | 1.7658 | 0.0120 | 1.9793 | 0.0058 | 1.5083 | 0.0678 | 2.0556 | 0.0006 | 2.4983 | 0.0000 |
| 11 | 63.7608 | 0.0000 | 0.8382 | 0.5402 | 47.8580 | 0.0000 | 2.9908 | 0.0503 | 19.4509 | 0.0000 | 0.9011 | 0.4930 | 19.8003 | 0.0000 | 0.8569 | 0.4245 |
| 12 | 2.3309 | 0.0000 | 1.5074 | 0.0119 | 1.6053 | 0.0022 | 0.2828 | 1.0000 | 2.3163 | 0.0000 | 1.9992 | 0.0004 | 1.5607 | 0.0119 | 0.3578 | 1.0000 |
| 13 | 2.3164 | 0.0000 | 2.0193 | 0.0000 | 1.2278 | 0.2831 | 3.8856 | 0.0003 | 1.8836 | 0.0001 | 1.8575 | 0.0001 | 1.4414 | 0.1836 | 2.5817 | 0.0117 |
| 14 | 1.6230 | 0.0015 | 1.1340 | 0.2202 | 1.1642 | 0.1666 | 1.2412 | 0.0861 | 1.6581 | 0.0001 | 1.0281 | 0.4082 | 1.1856 | 0.1029 | 1.0681 | 0.3053 |
| 15 | 7.8735 | 0.0000 | 1.0758 | 0.3768 | 9.5342 | 0.0000 | 7.7121 | 0.0000 | 2.0457 | 0.0000 | 1.2524 | 0.0538 | 1.5790 | 0.0004 | 2.4970 | 0.0000 |
| **5-Minute Data Resolution** | | | | | | | | | | | | | | | | |
| 1 | 0.6500 | 0.9954 | 0.4646 | 1.0000 | 1.3906 | 0.0114 | 0.9087 | 0.7141 | 0.7361 | 0.9503 | 0.3954 | 1.0000 | 1.8499 | 0.0000 | 1.1947 | 0.1353 |
| 2 | 4.4986 | 0.0000 | 1.4340 | 0.1307 | 7.2962 | 0.0000 | 4.5440 | 0.0001 | 2.2782 | 0.0047 | 2.1335 | 0.0088 | 5.3752 | 0.0000 | 5.1563 | 0.0000 |
| 3 | 0.7922 | 0.6238 | 1.2722 | 0.2499 | 4.8409 | 0.0001 | 0.3063 | 0.9336 | 0.5884 | 0.8068 | 1.3123 | 0.2281 | 1.8858 | 0.0818 | 0.6509 | 0.6894 |
| 4 | 0.5780 | 0.8588 | 0.2603 | 0.9942 | 3.6365 | 0.0280 | 0.2860 | 0.7515 | 0.8571 | 0.5915 | 0.2244 | 0.9971 | 1.6971 | 0.1857 | 0.8792 | 0.4166 |
| 5 | 0.7744 | 0.6774 | 1.7985 | 0.0446 | 0.7159 | 0.8598 | 1.2600 | 0.1682 | 0.8212 | 0.6287 | 1.8548 | 0.0366 | 0.6233 | 0.9368 | 1.3539 | 0.1062 |
| 6 | 0.4508 | 0.8683 | 151.6556 | 0.0000 | 0.8278 | 0.6026 | 0.1567 | 0.9986 | 1.3733 | 0.1763 | 0.9598 | 0.4982 | 2.1888 | 0.0057 | 0.6911 | 0.8269 |
| 7 | 5.2912 | 0.0000 | 19.6540 | 0.0000 | 0.2140 | 0.6440 | 0.1292 | 0.7195 | 0.5886 | 0.9349 | 0.6832 | 0.8616 | 5.4483 | 0.0013 | 0.7758 | 0.5087 |
| 8 | 18.4434 | 0.0000 | 0.9573 | 0.4120 | 3.0442 | 0.0000 | 1.3474 | 0.1208 | 11.9917 | 0.0000 | 0.7529 | 0.5839 | 2.0790 | 0.0001 | 1.5440 | 0.0164 |
| 9 | 5.0077 | 0.0001 | 36.3117 | 0.0000 | 9.1360 | 0.0025 | 20.4811 | 0.0000 | 1.6267 | 0.0177 | 5.0719 | 0.0000 | 3.4659 | 0.0021 | 25.0130 | 0.0000 |
| 10 | 2.6808 | 0.0000 | 2.9447 | 0.0000 | 2.9659 | 0.0000 | 2.4534 | 0.0000 | 2.8670 | 0.0000 | 3.0555 | 0.0000 | 3.1535 | 0.0000 | 3.3075 | 0.0000 |
| 11 | 21.6017 | 0.0000 | 0.5150 | 0.8643 | 12.2589 | 0.0000 | 0.5971 | 0.8002 | 84.5063 | 0.0000 | 3.9931 | 0.0461 | 52.1332 | 0.0000 | 1.7862 | 0.1817 |
| 12 | 4.4941 | 0.0000 | 0.8876 | 0.5355 | 1.1867 | 0.1890 | 0.9140 | 0.6352 | 27.1478 | 0.0000 | 3.4099 | 0.0332 | 0.4058 | 0.9444 | 1.5955 | 0.1025 |
| 13 | 1.3199 | 0.0513 | 1.9879 | 0.0000 | 1.5719 | 0.1511 | 4.5302 | 0.0001 | 1.2910 | 0.0538 | 2.0896 | 0.0000 | 1.8726 | 0.0699 | 3.0186 | 0.0037 |
| 14 | 1.3591 | 0.1178 | 1.6722 | 0.0234 | 2.0665 | 0.0008 | 1.0130 | 0.4484 | 1.3294 | 0.1349 | 1.7279 | 0.0170 | 1.9724 | 0.0016 | 0.9100 | 0.6068 |
| 15 | 2.1400 | 0.0000 | 1.9007 | 0.0000 | 1.4484 | 0.0044 | 3.0378 | 0.0000 | 2.1273 | 0.0000 | 1.8450 | 0.0000 | 1.5204 | 0.0015 | 2.9482 | 0.0000 |
| *ABP, arterial blood pressure; CPP, cerebral perfusion pressure; rSO_2_, regional cerebral oxygen saturation; TBI-GL, traumatic brain injury patient group without left frontal lobe pathology.* | | | | | | | | | | | | | | | | |

File S7e: Granger Causality Test Results for TBI-GR Population in 10-Second, 1-Minute, and 5-Minute Data Resolutions

| **Patient** | **ABP ® rSO_2__L (F Statistic)** | **ABP ® rSO_2__L (P-Value)** | **rSO_2__L ® ABP (F Statistic)** | **rSO_2__L ® ABP (P-Value)** | **ABP ® rSO_2__R (F Statistic)** | **ABP ® rSO_2__R (P-Value)** | **rSO_2__R ® ABP (F Statistic)** | **rSO_2__R ® ABP (P-Value)** | **CPP ® rSO_2__L (F Statistic)** | **CPP ® rSO_2__L (P-Value)** | **rSO_2__L ® CPP (F Statistic)** | **rSO_2__L ® CPP (P-Value)** | **CPP ® rSO_2__R (F Statistic)** | **CPP ® rSO_2__R (P-Value)** | **rSO_2__R ® CPP (F Statistic)** | **rSO_2__R ® CPP (P-Value)** |
| --- | --- | --- | --- | --- | --- | --- | --- | --- | --- | --- | --- | --- | --- | --- | --- | --- |
| **10-Second Data Resolution** | | | | | | | | | | | | | | | | |
| 1 | 1.8483 | 0.0015 | 1.9789 | 0.0004 | 11.2244 | 0.0000 | 3.4125 | 0.0000 | 2.8839 | 0.0000 | 3.1896 | 0.0000 | 27.9008 | 0.0000 | 7.9406 | 0.0000 |
| 2 | 0.5006 | 1.0000 | 0.9981 | 0.4868 | 0.4756 | 1.0000 | 0.2698 | 1.0000 | 0.5279 | 0.9999 | 0.9993 | 0.4821 | 0.4477 | 1.0000 | 0.2509 | 1.0000 |
| 3 | 8.4530 | 0.0000 | 2.6342 | 0.0001 | 6.0470 | 0.0000 | 5.5609 | 0.0000 | 4.6276 | 0.0000 | 2.1620 | 0.0019 | 1.1403 | 0.2679 | 2.1974 | 0.0001 |
| 4 | 1.6045 | 0.0125 | 1.6637 | 0.0077 | 9.0805 | 0.0000 | 2.2197 | 0.0644 | 1.1618 | 0.2572 | 1.5745 | 0.0298 | 4.3603 | 0.0045 | 0.9780 | 0.4020 |
| 5 | 1.7438 | 0.0000 | 2.9049 | 0.0000 | 2.0714 | 0.0232 | 0.7893 | 0.6393 | 1.5587 | 0.0006 | 2.5817 | 0.0000 | 71.2972 | 0.0000 | 6.7590 | 0.0000 |
| 6 | 3.5529 | 0.0000 | 0.9964 | 0.4866 | 5.1488 | 0.0000 | 2.1966 | 0.0000 | 2.3828 | 0.0000 | 0.9431 | 0.6324 | 4.0591 | 0.0000 | 1.7096 | 0.0000 |
| 7 | 1.5623 | 0.0052 | 0.7382 | 0.9242 | 0.3832 | 1.0000 | 0.2433 | 1.0000 | 2.5197 | 0.0000 | 0.6284 | 0.9429 | 0.5774 | 0.9896 | 0.3499 | 1.0000 |
| 8 | 1.8459 | 0.0000 | 2.0461 | 0.0000 | 46.6731 | 0.0000 | 4.5587 | 0.0000 | 2.1379 | 0.0000 | 1.9869 | 0.0000 | 90.5710 | 0.0000 | 7.3516 | 0.0000 |
| 9 | 9.6325 | 0.0000 | 2.3707 | 0.0113 | 4.9843 | 0.0000 | 3.9363 | 0.0000 | 4.6693 | 0.0000 | 1.9909 | 0.0435 | 2.8165 | 0.0000 | 3.2751 | 0.0000 |
| 10 | 2.4197 | 0.0000 | 0.6047 | 0.9980 | 1.3693 | 0.0601 | 1.2663 | 0.1209 | 1.4887 | 0.0012 | 0.8495 | 0.8583 | 1.5831 | 0.0054 | 0.9957 | 0.4821 |
| 11 | 6.6837 | 0.0000 | 9.7974 | 0.0000 | 0.1948 | 1.0000 | 14.7008 | 0.0000 | 6.3274 | 0.0000 | 8.4804 | 0.0000 | 0.2842 | 1.0000 | 10.1367 | 0.0000 |
| **1-Minute Data Resolution** | | | | | | | | | | | | | | | | |
| 1 | 10.2113 | 0.0000 | 1.1622 | 0.3261 | 0.7513 | 0.5572 | 1.4881 | 0.2038 | 1.9260 | 0.0283 | 1.5714 | 0.0945 | 1.7506 | 0.0523 | 3.1769 | 0.0002 |
| 2 | 0.5323 | 0.8329 | 0.9738 | 0.4545 | 0.0335 | 0.9671 | 0.1775 | 0.8374 | 0.6071 | 0.7726 | 1.0499 | 0.3958 | 0.1659 | 0.8471 | 0.2296 | 0.7949 |
| 3 | 6.8180 | 0.0000 | 18.4102 | 0.0000 | 2.0264 | 0.1080 | 14.3928 | 0.0000 | 5.3582 | 0.0003 | 6.8493 | 0.0000 | 0.0413 | 0.9888 | 9.6601 | 0.0000 |
| 4 | 1.6685 | 0.1024 | 1.3144 | 0.2327 | 6.6332 | 0.0000 | 3.7439 | 0.0050 | 0.8083 | 0.7284 | 1.0691 | 0.3741 | 1.5089 | 0.1154 | 1.9056 | 0.0306 |
| 5 | 6.0529 | 0.0000 | 17.9309 | 0.0000 | 4.2097 | 0.0000 | 1.6776 | 0.0097 | 6.6271 | 0.0000 | 15.4440 | 0.0000 | 2.2024 | 0.0000 | 1.9943 | 0.0002 |
| 6 | 0.2616 | 0.8531 | 0.7331 | 0.5322 | 3.5195 | 0.0018 | 1.8426 | 0.0868 | 13.6081 | 0.0000 | 0.5460 | 0.8415 | 6.9510 | 0.0000 | 4.3576 | 0.0000 |
| 7 | 1.6864 | 0.0112 | 1.2114 | 0.1986 | 0.3923 | 1.0000 | 0.2330 | 1.0000 | 1.3374 | 0.1143 | 1.2302 | 0.1915 | 0.7006 | 0.9738 | 0.4796 | 0.9999 |
| 8 | 11.7596 | 0.0000 | 10.8337 | 0.0000 | 1.2543 | 0.2812 | 1.7012 | 0.1310 | 8.8890 | 0.0000 | 11.3432 | 0.0000 | 1.5392 | 0.1744 | 1.9204 | 0.0879 |
| 9 | 2.6948 | 0.0000 | 2.1860 | 0.0002 | 3.4114 | 0.0000 | 2.4447 | 0.0000 | 1.7660 | 0.0087 | 2.5743 | 0.0000 | 3.2922 | 0.0000 | 2.3457 | 0.0000 |
| 10 | 2.3704 | 0.0086 | 0.5285 | 0.8712 | 4.2023 | 0.0008 | 2.1760 | 0.0541 | 0.9791 | 0.4593 | 0.6945 | 0.7306 | 4.3744 | 0.0006 | 5.7511 | 0.0000 |
| 11 | 3.1213 | 0.0000 | 6.4133 | 0.0000 | 3.8233 | 0.0000 | 15.3588 | 0.0000 | 2.2117 | 0.0000 | 5.8748 | 0.0000 | 2.5340 | 0.0000 | 12.8711 | 0.0000 |
| **5-Minute Data Resolution** | | | | | | | | | | | | | | | | |
| 1 | 2.6307 | 0.1067 | 2.4679 | 0.1181 | 1.2161 | 0.3056 | 3.2356 | 0.0238 | 0.7416 | 0.4779 | 5.5176 | 0.0048 | 1.3696 | 0.2302 | 1.6717 | 0.1314 |
| 2 | 0.5027 | 0.8327 | 1.2215 | 0.2889 | 0.3108 | 0.5774 | 1.9275 | 0.1656 | 0.6768 | 0.8572 | 1.3024 | 0.1670 | 0.9075 | 0.4372 | 1.3753 | 0.2495 |
| 3 | 5.2042 | 0.0000 | 10.4472 | 0.0000 | 2.7585 | 0.0412 | 4.0497 | 0.0071 | 1.7585 | 0.0007 | 2.6067 | 0.0000 | 0.8823 | 0.6152 | 1.3551 | 0.1313 |
| 4 | 0.2965 | 0.9746 | 2.0233 | 0.0415 | 4.2962 | 0.0153 | 4.3547 | 0.0145 | 0.2944 | 0.9752 | 1.4000 | 0.1946 | 2.4927 | 0.0861 | 3.1951 | 0.0438 |
| 5 | 0.6782 | 0.6404 | 0.4089 | 0.8424 | 3.3909 | 0.0047 | 2.0484 | 0.0692 | 0.4345 | 0.8554 | 0.2549 | 0.9570 | 7.5559 | 0.0000 | 1.2808 | 0.2628 |
| 6 | 0.0150 | 0.9024 | 1.1776 | 0.2785 | 3.6034 | 0.0030 | 3.1734 | 0.0074 | 1.3110 | 0.2529 | 2.8663 | 0.0912 | 6.5347 | 0.0000 | 14.5926 | 0.0000 |
| 7 | 1.4147 | 0.1543 | 0.6247 | 0.8220 | 0.6213 | 0.8249 | 0.4847 | 0.9241 | 1.4146 | 0.1543 | 0.3798 | 0.9706 | 0.6699 | 0.7811 | 0.2879 | 0.9912 |
| 8 | 1.4012 | 0.1343 | 1.4618 | 0.1075 | 0.6939 | 0.8172 | 1.1904 | 0.2667 | 0.3364 | 0.9172 | 1.7947 | 0.1010 | 0.5401 | 0.7776 | 1.0167 | 0.4140 |
| 9 | 2.6616 | 0.0035 | 2.1568 | 0.0190 | 16.4964 | 0.0000 | 5.6249 | 0.0038 | 1.9930 | 0.0319 | 2.0674 | 0.0252 | 12.0864 | 0.0000 | 5.4506 | 0.0045 |
| 10 | 0.5950 | 0.9802 | 0.9441 | 0.5739 | 2.3628 | 0.0034 | 1.1428 | 0.3167 | 0.5723 | 0.9683 | 0.6183 | 0.9459 | 1.2868 | 0.2341 | 2.1957 | 0.0167 |
| 11 | 1.5048 | 0.2224 | 101.0152 | 0.0000 | 1.0364 | 0.3550 | 135.7145 | 0.0000 | 0.9171 | 0.4689 | 36.8641 | 0.0000 | 0.7782 | 0.5654 | 47.7853 | 0.0000 |
| *ABP, arterial blood pressure; CPP, cerebral perfusion pressure; rSO_2_, regional cerebral oxygen saturation; TBI-GR, traumatic brain injury patient group without right frontal lobe pathology.* | | | | | | | | | | | | | | | | |

File S7f: Granger Causality Test Results for TBI-BLR Population in 10-Second, 1-Minute, and 5-Minute Data Resolutions

| **Patient** | **ABP ® rSO_2__L (F Statistic)** | **ABP ® rSO_2__L (P-Value)** | **rSO_2__L ® ABP (F Statistic)** | **rSO_2__L ® ABP (P-Value)** | **ABP ® rSO_2__R (F Statistic)** | **ABP ® rSO_2__R (P-Value)** | **rSO_2__R ® ABP (F Statistic)** | **rSO_2__R ® ABP (P-Value)** | **CPP ® rSO_2__L (F Statistic)** | **CPP ® rSO_2__L (P-Value)** | **rSO_2__L ® CPP (F Statistic)** | **rSO_2__L ® CPP (P-Value)** | **CPP ® rSO_2__R (F Statistic)** | **CPP ® rSO_2__R (P-Value)** | **rSO_2__R ® CPP (F Statistic)** | **rSO_2__R ® CPP (P-Value)** |
| --- | --- | --- | --- | --- | --- | --- | --- | --- | --- | --- | --- | --- | --- | --- | --- | --- |
| **10-Second Data Resolution** | | | | | | | | | | | | | | | | |
| 1 | 3.0800 | 0.0000 | 2.6895 | 0.0000 | 2.4590 | 0.0000 | 2.1063 | 0.0000 | 2.0936 | 0.0000 | 2.7883 | 0.0000 | 2.0629 | 0.0000 | 2.0972 | 0.0000 |
| 2 | 2.1867 | 0.0000 | 1.2296 | 0.0694 | 1.5099 | 0.0013 | 1.7474 | 0.0000 | 1.3738 | 0.0110 | 1.5537 | 0.0006 | 0.9420 | 0.6357 | 1.1610 | 0.1411 |
| 3 | 14.7099 | 0.0000 | 0.9618 | 0.5762 | 4.2443 | 0.0000 | 0.7092 | 0.9708 | 7.8933 | 0.0000 | 1.5398 | 0.0014 | 2.4506 | 0.0000 | 0.7401 | 0.9518 |
| 4 | 3.8001 | 0.0000 | 2.9776 | 0.0000 | 2.4533 | 0.0000 | 1.6904 | 0.0001 | 3.6116 | 0.0000 | 2.0692 | 0.0000 | 1.7914 | 0.0000 | 1.8160 | 0.0000 |
| 5 | 2.5698 | 0.0000 | 0.7388 | 0.9612 | 1.1412 | 0.2061 | 0.8447 | 0.8071 | 5.0966 | 0.0000 | 0.9287 | 0.5778 | 1.5725 | 0.0370 | 0.7831 | 0.7628 |
| **1-Minute Data Resolution** | | | | | | | | | | | | | | | | |
| 1 | 9.4379 | 0.0000 | 4.2472 | 0.0000 | 7.9130 | 0.0000 | 2.1326 | 0.0037 | 8.9279 | 0.0000 | 5.9965 | 0.0000 | 4.8440 | 0.0000 | 1.7948 | 0.0209 |
| 2 | 2.1945 | 0.0000 | 1.3000 | 0.0709 | 2.0829 | 0.0046 | 1.4874 | 0.0835 | 2.1579 | 0.0000 | 1.2222 | 0.1419 | 0.7559 | 0.7374 | 2.1261 | 0.0054 |
| 3 | 21.8025 | 0.0000 | 1.5370 | 0.1285 | 0.5840 | 0.8114 | 0.1731 | 0.9967 | 16.3023 | 0.0000 | 2.2268 | 0.0177 | 1.4471 | 0.1617 | 0.1499 | 0.9981 |
| 4 | 1.2941 | 0.1408 | 2.2823 | 0.0002 | 1.7540 | 0.0066 | 1.8231 | 0.0039 | 1.1153 | 0.2146 | 1.1964 | 0.1000 | 1.0213 | 0.4228 | 1.2282 | 0.0616 |
| 5 | 1.2963 | 0.0970 | 0.7556 | 0.8739 | 1.3074 | 0.0776 | 1.0478 | 0.3845 | 0.9043 | 0.6760 | 0.6361 | 0.9839 | 0.6642 | 0.9815 | 0.8769 | 0.7455 |
| **5-Minute Data Resolution** | | | | | | | | | | | | | | | | |
| 1 | 3.9028 | 0.0000 | 2.3978 | 0.0046 | 5.7339 | 0.0008 | 2.1967 | 0.0879 | 4.3754 | 0.0000 | 3.7288 | 0.0000 | 4.2376 | 0.0004 | 1.7636 | 0.1053 |
| 2 | 2.0707 | 0.0002 | 0.6145 | 0.9701 | 1.4502 | 0.2030 | 1.7352 | 0.1231 | 1.5453 | 0.0057 | 0.7450 | 0.9351 | 0.5549 | 0.8347 | 1.3038 | 0.2293 |
| 3 | 10.9392 | 0.0000 | 1.7058 | 0.0003 | 11.8472 | 0.0000 | 17.3275 | 0.0000 | 9.4864 | 0.0000 | 2.6763 | 0.0000 | 8.9480 | 0.0000 | 9.7892 | 0.0000 |
| 4 | 1.4433 | 0.0572 | 1.4252 | 0.0637 | 0.7168 | 0.9785 | 1.2975 | 0.0348 | 2.0316 | 0.0008 | 1.4770 | 0.0465 | 0.8125 | 0.8978 | 1.0134 | 0.4471 |
| 5 | 3.5178 | 0.0000 | 0.9511 | 0.5432 | 3.0283 | 0.0000 | 0.8293 | 0.7263 | 2.2300 | 0.0153 | 3.5447 | 0.0002 | 2.0423 | 0.0279 | 2.6723 | 0.0035 |
| *ABP, arterial blood pressure; CPP, cerebral perfusion pressure; rSO_2_, regional cerebral oxygen saturation; TBI-BLR, traumatic brain injury patient group with bifrontal lobe pathology.* | | | | | | | | | | | | | | | | |

File S7g: Granger Causal Directionality Results based on greater F-Statistic in 1-Minute and 5-Minute Data Resolutions for HC, SP, and TBI-GLR Populations

| **Signal** | **Direction** | **HC (n=102)** | | **SP (n=27)** | | **TBI-GLR (n=64)** | |
| --- | --- | --- | --- | --- | --- | --- | --- |
|  |  | **1-Minute**  **[% (count)]** | **5-Minute**  **[% (count)]** | **1-Minute**  **[% (count)]** | **5-Minute**  **[% (count)]** | **1-Minute**  **[% (count)]** | **5-Minute**  **[% (count)]** |
| **Personalized VAR P-Order** | | | | | | | |
| ABP & rSO_2__L | ABP ® rSO_2__L | 46.1% (47) | 0% (0) | **40.7% (11)** | **33.3% (9)** | **45.3% (29)** | 57.8% (37) |
|  | rSO_2__L ® ABP | 40.2% (41) | 0% (0) | **55.6% (15)** | **59.3% (16)** | **54.7% (35)** | 42.2% (27) |
|  | NA | 13.7% (14) | 100% (102) | 3.7% (1) | 7.4% (2) | 0% (0) | 0% (0) |
| ABP & rSO_2__R | ABP ® rSO_2__R | **41.2% (42)** | 0% (0) | 51.9% (14) | 44.4% (12) | **42.2% (27)** | **42.2% (27)** |
|  | rSO_2__R ® ABP | **47.1% (48)** | 0% (0) | 33.3% (9) | 40.7% (11) | **57.8% (37)** | **57.8% (37)** |
|  | NA | 11.8% (12) | 100% (102) | 14.8% (4) | 14.8% (4) | 0% (0) | 0% (0) |
| CPP & rSO_2__L | CPP ® rSO_2__L | – | – | – | – | 48.4% (31) | 54.7% (35) |
|  | rSO_2__L ® CPP | – | – | – | – | 50% (32) | 42.2% (27) |
|  | NA | – | – | – | – | 1.6% (1) | 3.1% (2) |
| CPP & rSO_2__R | CPP ® rSO_2__R | – | – | – | – | **45.3% (29)** | 48.4% (31) |
|  | rSO_2__R ® CPP | – | – | – | – | **54.7% (35)** | 48.4% (31) |
|  | NA | – | – | – | – | 0% (0) | 3.1% (2) |
| **Capped VAR P-Order at 10** | | | | | | | |
| ABP & rSO_2__L | ABP ® rSO_2__L | 45.1% (46) | 0% (0) | **48.1% (13)** | **37% (10)** | **37.5% (24)** | 54.7% (35) |
|  | rSO_2__L ® ABP | 42.2% (43) | 0% (0) | **51.9% (14)** | **55.6% (15)** | **62.5% (40)** | 45.3% (29) |
|  | NA | 12.7% (13) | 100% (102) | 0% (0) | 7.4% (2) | 0% (0) | 0% (0) |
| ABP & rSO_2__R | ABP ® rSO_2__R | **42.2% (43)** | 0% (0) | 55.6% (15) | 48.1% (13) | **37.5% (24)** | **48.4% (31)** |
|  | rSO_2__R ® ABP | **49% (50)** | 0% (0) | 44.4% (12) | 37% (10) | **62.5% (40)** | **51.6% (33)** |
|  | NA | 8.8% (9) | 100% (102) | 0% (0) | 14.8% (4) | 0% (0) | 0% (0) |
| CPP & rSO_2__L | CPP ® rSO_2__L | – | – | – | – | **48.4% (31)** | **56.3% (36)** |
|  | rSO_2__L ® CPP | – | – | – | – | **50% (32)** | **40.6% (26)** |
|  | NA | – | – | – | – | 1.6% (1) | 3.1% (2) |
| CPP & rSO_2__R | CPP ® rSO_2__R | – | – | – | – | **45.3% (29)** | **54.7% (35)** |
|  | rSO_2__R ® CPP | – | – | – | – | **54.7% (35)** | **42.2% (27)** |
|  | NA | – | – | – | – | 0% (0) | 3.1% (2) |
| *ABP, arterial blood pressure; HC, healthy control volunteer group;* *p-order, autoregressive order; rSO_2_, regional cerebral oxygen saturation; SP, elective spinal surgery patient group; TBI-GLR, traumatic brain injury patient group without bifrontal lobe pathology.* | | | | | | | |

File S7h: Granger Causal Directionality Results based on greater F-Statistic in 10-Second, 1-Minute, and 5-Minute Data Resolutions for TBI-GL, TBI-GR, and TBI-BLR Populations

| **Signal** | **Direction** | **TBI-GL (n=15)** | | | **TBI-GR (n=11)** | | | **TBI-BLR (n=5)** | | |
| --- | --- | --- | --- | --- | --- | --- | --- | --- | --- | --- |
|  |  | **10-Second**  **[% (count)]** | **1-Minute**  **[% (count)]** | **5-Minute**  **[% (count)]** | **10-Second**  **[% (count)]** | **1-Minute**  **[% (count)]** | **5-Minute**  **[% (count)]** | **10-Second**  **[% (count)]** | **1-Minute**  **[% (count)]** | **5-Minute**  **[% (count)]** |
| **Personalized VAR P-Order** | | | | | | | | | | |
| ABP & rSO_2__L | ABP ® rSO_2__L | 80% (12) | 53.3% (8) | 46.7% (7) | **45.5% (5)** | 54.5% (6) | **36.4% (4)** | 100% (5) | 80% (4) | 100% (5) |
|  | rSO_2__L ® ABP | 20% (3) | 46.7% (7) | 53.3% (8) | **54.5% (6)** | 45.5% (5) | **63.6% (7)** | 0% (0) | 20% (1) | 0% (0) |
|  | NA | 0% (0) | 0% (0) | 0% (0) | 0% (0) | 0% (0) | 0% (0) | 0% (0) | 0% (0) | 0% (0) |
| ABP & rSO_2__R | ABP ® rSO_2__R | 100% (15) | 60% (9) | 73.3% (11) | 90.9% (10) | 54.5% (6) | **45.5% (5)** | 80% (4) | 80% (4) | **40% (2)** |
|  | rSO_2__R ® ABP | 0% (0) | 40% (6) | 26.7% (4) | 9.1% (1) | 45.5% (5) | **54.5% (6)** | 20% (1) | 20% (1) | **60% (3)** |
|  | NA | 0% (0) | 0% (0) | 0% (0) | 0% (0) | 0% (0) | 0% (0) | 0% (0) | 0% (0) | 0% (0) |
| CPP & rSO_2__L | CPP ® rSO_2__L | 80% (12) | 73.3% (11) | 53.3% (8) | 54.5% (6) | **36.4% (4)** | **18.2% (2)** | 60% (3) | 80% (4) | 80% (4) |
|  | rSO_2__L ® CPP | 20% (3) | 26.7% (4) | 46.7% (7) | 45.5% (5) | **63.6% (7)** | **81.8% (9)** | 40% (2) | 20% (1) | 20% (1) |
|  | NA | 0% (0) | 0% (0) | 0% (0) | 0% (0) | 0% (0) | 0% (0) | 0% (0) | 0% (0) | 0% (0) |
| CPP & rSO_2__R | CPP ® rSO_2__R | 93.3% (14) | 53.3% (8) | 60% (9) | 72.7% (8) | **36.4% (4)** | **27.3% (3)** | **40% (2)** | **40% (2)** | **20% (1)** |
|  | rSO_2__R ® CPP | 6.7% (1) | 46.7% (7) | 40% (6) | 27.3% (3) | **63.6% (7)** | **72.7% (8)** | **60% (3)** | **60% (3)** | **80% (4)** |
|  | NA | 0% (0) | 0% (0) | 0% (0) | 0% (0) | 0% (0) | 0% (0) | 0% (0) | 0% (0) | 0% (0) |
| **Capped VAR P-Order at 10** | | | | | | | | | | |
| ABP & rSO_2__L | ABP ® rSO_2__L | 93.3% (14) | 60% (9) | 60% (9) | **63.6% (7)** | 54.5% (6) | **45.5% (5)** | 100% (5) | 80% (4) | 60% (3) |
|  | rSO_2__L ® ABP | 6.7% (1) | 40% (6) | 40% (6) | **36.4% (4)** | 45.5% (5) | **54.5% (6)** | 0% (0) | 20% (1) | 40% (2) |
|  | NA | 0% (0) | 0% (0) | 0% (0) | 0% (0) | 0% (0) | 0% (0) | 0% (0) | 0% (0) | 0% (0) |
| ABP & rSO_2__R | ABP ® rSO_2__R | 93.3% (14) | 60% (9) | 66.7% (10) | 90.9% (10) | **45.5% (5)** | **45.5% (5)** | 100% (5) | 80% (4) | **40% (2)** |
|  | rSO_2__R ® ABP | 6.7% (1) | 40% (6) | 33.3% (5) | 9.1% (1) | **54.5% (6)** | **54.5% (6)** | 0% (0) | 20% (1) | **60% (3)** |
|  | NA | 0% (0) | 0% (0) | 0% (0) | 0% (0) | 0% (0) | 0% (0) | 0% (0) | 0% (0) | 0% (0) |
| CPP & rSO_2__L | CPP ® rSO_2__L | 93.3% (14) | 60% (9) | 73.3% (11) | 63.6% (7) | **45.5% (5)** | **27.3% (3)** | 60% (3) | 80% (4) | 60% (3) |
|  | rSO_2__L ® CPP | 6.7% (1) | 40% (6) | 26.7% (4) | 36.4% (4) | **54.5% (6)** | **72.7% (8)** | 40% (2) | 20% (1) | 40% (2) |
|  | NA | 0% (0) | 0% (0) | 0% (0) | 0% (0) | 0% (0) | 0% (0) | 0% (0) | 0% (0) | 0% (0) |
| CPP & rSO_2__R | CPP ® rSO_2__R | 93.3% (14) | 60% (9) | 60% (9) | 81.8% (9) | **18.2% (2)** | **27.3% (3)** | 100% (5) | **40% (2)** | **40% (2)** |
|  | rSO_2__R ® CPP | 6.7% (1) | 40% (6) | 40% (6) | 18.2% (2) | **81.8% (9)** | **72.7% (8)** | 0% (0) | **60% (3)** | **60% (3)** |
|  | NA | 0% (0) | 0% (0) | 0% (0) | 0% (0) | 0% (0) | 0% (0) | 0% (0) | 0% (0) | 0% (0) |
| *ABP, arterial blood pressure; p-order, autoregressive order; rSO_2_, regional cerebral oxygen saturation; TBI-BLR, traumatic brain injury patient group with bifrontal lobe pathology; TBI-GL, traumatic brain injury patient group without left frontal lobe pathology; TBI-GR, traumatic brain injury patient group without right frontal lobe pathology.* | | | | | | | | | | |
