# Supplementary material for: Commercial NIRS May Not Detect Hemispheric Regional Disparity in Continuously Measured COx/COx-a: An Exploratory Healthy and Cranial Trauma Time-Series Analysis
Source: Bioengineering (Basel). 2025 Feb 28;12(3):247. doi: 10.3390/bioengineering12030247 (PMC11939202; doi:10.3390/bioengineering12030247)
Supplement: Supplementary file 1 [file bioengineering-12-00247-s001.zip › File S8.docx]

**File S8 – Sub-group Analysis Appendix**

File S8 – Table of Contents

[File S8a: Summary of Sub-group Analysis 4](#_Toc191509039)

[File S8b: Sub-grouped Median Entire Recording Summary Metrics – Hemispheric Differences 5](#_Toc191509040)

[File S8c: Sub-grouped Absolute Regional Hemispheric Differences (ARHD) – 10-second, 1-minute, 5-minute Data 6](#_Toc191509041)

[File S8d: Sub-grouped Hemispheric Difference in Personalized ARIMA P-Order and Q-Order – 10-second, 1-minute, 5-minute Data 7](#_Toc191509042)

[File S8e: Sub-grouped Hemispheric Difference in Impulse Response Function (IRF) of ABP/CPP on rSO2 – 10-second, 1-minute, 5-minute Data 8](#_Toc191509043)

[File S8f: Sub-grouped Hemispheric Difference in Granger Causality Between ABP/CPP and rSO2 – 10-second, 1-minute, 5-minute Data 10](#_Toc191509044)

[File S8g: Sub-grouped Physiologic Results using 10-Second, 1-Minute, and 5-Minute Data Resolutions for HC Population 12](#_Toc191509045)

[File S8h: Sub-grouped Physiologic Results using 10-Second, 1-Minute, and 5-Minute Data Resolutions for SP Population 14](#_Toc191509046)

[File S8i: Sub-grouped Physiologic Results using 10-Second, 1-Minute Data, and 5-Minute Resolutions for TBI-GLR Population 20](#_Toc191509047)

[File S8j: Sub-grouped Physiologic Results using 10-Second, 1-Minute, and 5-Minute Data Resolutions for TBI-GL Population 29](#_Toc191509048)

[File S8k: Sub-grouped Physiologic Results using 10-Second, 1-Minute, and 5-Minute Data Resolutions for TBI-GR Population 35](#_Toc191509049)

[File S8l: Sub-grouped Physiologic Results using 10-Second, 1-Minute, and 5-Minute Data Resolutions for TBI-BLR Population 41](#_Toc191509050)

[File S8m: Sub-grouped Absolute Regional Hemispheric Disparity Analysis using 10-Second, 1-Minute, and 5-Minute Data Resolutions for HC Population 46](#_Toc191509051)

[File S8n: Sub-grouped Absolute Regional Hemispheric Disparity Analysis using 10-Second, 1-Minute, and 5-Minute Data Resolutions for SP Population 47](#_Toc191509052)

[File S8o: Sub-grouped Absolute Regional Hemispheric Disparity Analysis using 10-Second, 1-Minute, and 5-Minute Data Resolutions for TBI-GLR Population 48](#_Toc191509053)

[File S8p: Sub-grouped Absolute Regional Hemispheric Disparity Analysis using 10-Second, 1-Minute, and 5-Minute Data Resolutions for TBI-GL Population 50](#_Toc191509054)

[File S8q: Sub-grouped Absolute Regional Hemispheric Disparity Analysis using 10-Second, 1-Minute, and 5-Minute Data Resolutions for TBI-GR Population 51](#_Toc191509055)

[File S8r: Sub-grouped Absolute Regional Hemispheric Disparity Analysis using 10-Second, 1-Minute, and 5-Minute Data Resolutions for TBI-BLR Population 52](#_Toc191509056)

[File S8s: Sub-grouped Personalized ARIMA P-Orders and Q-Orders based on AIC and their Hemispheric Disparity using 10-Second, 1-Minute, and 5-Minute Data Resolutions for HC Population 53](#_Toc191509057)

[File S8t: Sub-grouped Personalized ARIMA P-Orders and Q-Orders based on AIC and their Hemispheric Disparity using 10-Second, 1-Minute, and 5-Minute Data Resolutions for SP Population 54](#_Toc191509058)

[File S8u: Sub-grouped Personalized ARIMA P-Orders and Q-Orders based on AIC and their Hemispheric Disparity using 10-Second, 1-Minute Data, and 5-Minute Resolutions for TBI-GLR Population 56](#_Toc191509059)

[File S8v: Sub-grouped Personalized ARIMA P-Orders and Q-Orders based on AIC and their Hemispheric Disparity using 10-Second, 1-Minute, and 5-Minute Data Resolutions for TBI-GL Population 59](#_Toc191509060)

[File S8w: Sub-grouped Personalized ARIMA P-Orders and Q-Orders based on AIC and their Hemispheric Disparity using 10-Second, 1-Minute, and 5-Minute Data Resolutions for TBI-GR Population 61](#_Toc191509061)

[File S8x: Sub-grouped Personalized ARIMA P-Orders and Q-Orders based on AIC and their Hemispheric Disparity using 10-Second, 1-Minute, and 5-Minute Data Resolutions for TBI-BLR Population 63](#_Toc191509062)

[File S8y: Sub-grouped Hemispheric Responsiveness using Impulse Response Coefficients of VAR model using Personalized and Capped VAR P-Order using 10-Second, 1-Minute, and 5-Minute Data Resolutions for HC Population 65](#_Toc191509063)

[File S8z: Sub-grouped Hemispheric Responsiveness using Impulse Response Coefficients of VAR model using Personalized and Capped VAR P-Order using 10-Second, 1-Minute, and 5-Minute Data Resolutions for SP Population 66](#_Toc191509064)

[File S8aa: Sub-grouped Hemispheric Responsiveness using Impulse Response Coefficients of VAR model using Personalized and Capped VAR P-Order using 10-Second, 1-Minute, and 5-Minute Data Resolutions for TBI-GLR Population 68](#_Toc191509065)

[File S8bb: Sub-grouped Hemispheric Responsiveness using Impulse Response Coefficients of VAR model using Personalized and Capped VAR P-Order using 10-Second, 1-Minute, and 5-Minute Data Resolutions for TBI-GL Population 73](#_Toc191509066)

[File S8cc: Sub-grouped Hemispheric Responsiveness using Impulse Response Coefficients of VAR model using Personalized and Capped VAR P-Order using 10-Second, 1-Minute, and 5-Minute Data Resolutions for TBI-GR Population 76](#_Toc191509067)

[File S8dd: Sub-grouped Hemispheric Responsiveness using Impulse Response Coefficients of VAR model using Personalized and Capped VAR P-Order using 10-Second, 1-Minute, and 5-Minute Data Resolutions for TBI-BLR Population 80](#_Toc191509068)

[File S8ee: Sub-grouped Granger Causal Directionality Results based on greater F-Statistic using 10-Second, 1-Minute, and 5-Minute Resolutions for HC Population 82](#_Toc191509069)

[File S8ff: Sub-grouped Granger Causal Directionality Results based on greater F-Statistic using 10-Second, 1-Minute, and 5-Minute Data Resolutions for SP Population 83](#_Toc191509070)

[File S8gg: Sub-grouped Granger Causal Directionality Results based on greater F-Statistic using 10-second, 1-Minute, and 5-Minute Data Resolutions for TBI-GLR Population 85](#_Toc191509071)

[File S8hh: Sub-grouped Granger Causal Directionality Results based on greater F-Statistic using 10-second, 1-Minute, and 5-Minute Data Resolutions for TBI-GL Population 91](#_Toc191509072)

[File S8ii: Sub-grouped Granger Causal Directionality Results based on greater F-Statistic using 10-second, 1-Minute, and 5-Minute Data Resolutions for TBI-GR Population 97](#_Toc191509073)

[File S8jj: Sub-grouped Granger Causal Directionality Results based on greater F-Statistic using 10-second, 1-Minute, and 5-Minute Data Resolutions for TBI-BLR Population 101](#_Toc191509074)

[File S8kk: References 107](#_Toc191509075)

File S8a: Summary of Sub-group Analysis

With each population subdivided using previous defined parameters, re-evaluation of the physiologic results, regional hemispheric disparity analysis, personalized autoregressive integrative moving average (ARIMA) p-orders based on Akaike Information Criterion (AIC), vector autoregressive (VAR) impulse response function (IRF) analysis, and Granger Causality analysis occurred to find if any produced outlying groups.

The healthy control volunteer (HC) population were sub-grouped into Age < 40 (n = 95), Age 40 – 60 (n = 6), Age > 60 (n = 1), Males (n = 42), Females (n = 60), Left Hand Dominance (n = 9), and Right Hand Dominance (n = 93).

The elective spinal surgery (SP) population were sub-grouped into Age < 40 (n = 1), Age 40 – 60 (n = 17), Age > 60 (n = 9), Males (n = 22), Females (n = 5), anterior cervical discectomy and fusion (ACDF; n = 6), posterior cervical discectomy and fusion (PCDF; n = 25), ACDF & PCDF (n = 3), Cervical Incision and Drain (n = 1), Corpectomy (n = 1), Laminectomy (n = 1), Thoracic Decompression & Instrumental Fusion (n = 1), Propofol + Sufentanil (n = 6), Ketamine + Propofol + Sufentanil (n = 6), Midazolam + Propofol + Remi-Fentanyl (n = 1), Midazolam + Propofol + Sufentanil (n = 4), Propofol + Remi-Fentanyl + Sufentanil (n = 2), Ketamine + Midazolam + Propofol + Sufentanil (n = 7), and Ketamine + Midazolam + Propofol + Remi-Fentanyl + Sufentanil (n = 1).

The traumatic brain injury without bifrontal lobe pathology (TBI-GLR) were sub-grouped into Age < 40 (n = 28), Age 40 – 60 (n = 23), Age > 60 (n = 13), Males (n = 51), Females (n = 13), Focal Injury (subdural hematoma [SDH], acute SDH [aSDH], epidural hematoma [EDH], or Contusion) (n = 42), Diffuse Injury (diffuse axonal injury [DAI] or traumatic subarachnoid hemorrhage [tSAH]) (n = 22), Marshall computed tomography (CT) V (n = 29), Marshall CT IV (n = 8), Marshall CT III (n = 25), Marshall CT II (n = 2), Rotterdam CT 6 (n = 14), Rotterdam CT 5 (n = 16), Rotterdam CT 4 (n = 17), Rotterdam CT 3 (n = 13), Rotterdam CT 2 (n = 3), Rotterdam CT 1 (n = 1), No Anesthetic (n = 2), Propofol (n = 14), Fentanyl + Propofol (n = 22), Ketamine + Propofol (n = 2), Midazolam + Propofol (n = 1), Fentanyl + Ketamine + Propofol (n = 5), Fentanyl + Midazolam + Propofol (n = 13), and Fentanyl + Ketamine + Midazolam + Propofol (n = 5).

The traumatic brain injury without left frontal lobe pathology (TBI-GL) were sub-grouped into Age < 40 (n = 5), Age 40 – 60 (n = 6), Age > 60 (n = 4), Males (n = 14), Females (n = 1), Focal Injury (aSDH, SDH, EDH, or Contusion) (n = 14), Diffuse Injury (DAI or tSAH) (n = 1), Marshall CT V (n = 8), Marshall CT IV (n = 5), Marshall CT III (n = 2), Rotterdam CT 6 (n = 1), Rotterdam CT 5 (n = 7), Rotterdam CT 4 (n = 5), Rotterdam CT 3 (n = 2), Propofol (n = 5), Fentanyl + Propofol (n = 3), Fentanyl + Ketamine + Propofol (n = 1), Fentanyl + Midazolam + Propofol (n = 4), and Fentanyl + Ketamine + Midazolam + Propofol (n = 2).

The traumatic brain injury without right frontal lobe pathology (TBI-GR) were sub-grouped into Age < 40 (n = 4), Age 40 – 60 (n = 3), Age > 60 (n = 4), Males (n = 7), Females (n = 4), Focal Injury (aSDH, SDH, EDH, or Contusion) (n = 10), Diffuse Injury (DAI or tSAH) (n = 1), Marshall CT V (n = 8), Marshall CT IV (n = 2), Marshall CT II (n = 1), Rotterdam CT 6 (n = 5), Rotterdam CT 5 (n = 1), Rotterdam CT 4 (n = 4), Rotterdam CT 2 (n = 1), Propofol (n = 3), Fentanyl + Propofol (n = 6), and Fentanyl + Midazolam + Propofol (n = 2).

The traumatic brain injury with bifrontal lobe pathology (TBI-BLR) were sub-grouped into Age < 40 (n = 4), Age 40 – 60 (n = 1), Males (n = 5), Focal Injury (aSDH, SDH, EDH, or Contusion) (n = 4), Diffuse Injury (DAI or tSAH) (n = 1), Marshall CT V (n = 3), Marshall CT IV (n = 1), Marshall CT III (n = 1), Rotterdam CT 6 (n = 2), Rotterdam CT 4 (n = 3), Fentanyl + Propofol (n = 1), Fentanyl + Ketamine + Propofol (n = 1), Fentanyl + Midazolam + Propofol (n = 1), and Fentanyl + Ketamine + Midazolam + Propofol (n = 2).

File S8b: Sub-grouped Median Entire Recording Summary Metrics – Hemispheric Differences

In the HC population, the female biological sex sub-group had significant p-values between left and right side of percent time of rSO2 above 70% and 90% (p = 0.041 and p = 0.0437, respectively), and the left hand dominance sub-group only had significant p-value between left and right side of percent time of rSO2 above 60% (p = 0.0339) in the 10-second data resolution. In the 1-minute data resolution of HC population, the female biological sex sub-group only had significant p-value between left and right side for percent time of rSO2 above 70% (p = 0.0159), and the left hand dominance sub-group continued to have a significant p-value between left and right side of percent time of rSO2 above 60% (p = 0.0339) with the addition of age less than 40 years sub-group having a significant p-value between left and right side of percent time rSO2 above 70 %(p = 0.0346). The TBI-GL population had a significant p-value between the left and right sides of MAD of COx-a (p = 0.026) only in the age between 40 – 60 years sub-group in 1-minute data resolution. While the TBI-GR population had significant p-values in 10-second data resolution for the age less than 40 years sub-group between the left and right sides of percent time of COx greater than 0 and 0.2, and in 1-minute data resolution, only the p-value between the left and right side of MAD of COx was significant in the males sub-group (p = 0.0379). No significant p-values were found in any of the sub-groups for the SP, TBI-GLR, and TBI-BLR populations in the three data resolutions. Appendices H7 to H12 shows the sub-grouped patient physiology results with Mann-Whitney U test p-values for HC, SP, TBI-GLR, TBI-GL, TBI-GR, and TBI-BLR populations, respectively, in the three data resolutions.

File S8c: Sub-grouped Absolute Regional Hemispheric Differences (ARHD) – 10-second, 1-minute, 5-minute Data

The HC sub-group analysis for regional hemispheric disparity failed to demonstrate any findings that would be considered different from the full data in the three data resolutions and these results are given in File S8m. In the SP population, the sub-group analysis only showed the Ketamine + Midazolam + Propofol + Remi-Fentanyl + Sufentanil anesthetic sub-group to have different ARHD of COx-a along with its MAD (0.25 and 0.28, respectively, in 10-second data resolution) compared to their global population medians (0.15 and 0.16, respectively, in 10-second data resolution) in the three data resolutions. File S8n shows these results for the SP population using the three data resolutions. The sub-group analysis for regional hemispheric disparity for TBI-GLR, TBI-GL, TBI-GR, and TBI-BLR populations failed to demonstrate any findings that would be considered different from the full data in the three data resolutions and these results are given in Appendices H15 to H18, respectively.

File S8d: Sub-grouped Hemispheric Difference in Personalized ARIMA P-Order and Q-Order – 10-second, 1-minute, 5-minute Data

In HC population, there were similar results amongst all sub-groups for median personalized ARIMA p-order and q-order between left and right sides for the rSO2 and COx-a with their median ARHD equal or below 3 in the three data resolutions. File S8s shows the sub-grouped personalized ARIMA p-order and q-order with hemispheric disparity results in the three data resolutions for the HC population. While the SP population had similar results amongst most sub-groups for median personalized ARIMA p-order and q-order between left and right sides for the rSO2 and COx-a, their median ARHD were mostly below 5 in the three data resolutions. File S8t shows the sub-grouped personalized ARIMA p-order with hemispheric disparity results in the three data resolutions for the SP population. Looking at the TBI-GLR population, similar median personalized ARIMA p-order and q-order between left and right sides for the rSO2, COx, and COx-a were found with their median ARHD mostly below 3 in the three data resolutions. File S8u shows the sub-grouped personalized ARIMA p-order with hemispheric disparity results in the three data resolutions for the TBI-GLR population. The TBI-GL population had similar results amongst most sub-groups for median personalized ARIMA p-order and q-order between left and right sides for the rSO2, COx, and COx-a, their median ARHD were mostly below 5 in the three data resolutions. File S8v shows the sub-grouped personalized ARIMA p-order with hemispheric disparity results in the three data resolutions for the TBI-GL population. Similar results to TBI-GL population can be seen for the TBI-GR population which are given in File S8w. In the TBI-BLR population, the hemispheric median personalized ARIMA p-orders for rSO2, COx, and COx-a were similar with their median ARHD equal to or less than 5 and these results are given in File S8x.

File S8e: Sub-grouped Hemispheric Difference in Impulse Response Function (IRF) of ABP/CPP on rSO2 – 10-second, 1-minute, 5-minute Data

As previously mentioned, the responsiveness of bivariate VAR IRF models were checked for variations in regional disparity by looking at the percentage of patients showing a greater response than 0.1%, using personalized and capped p-orders for each direction of signal combination in each sub-group of a population. The following signal combinations were looked at in each data resolution: ABP & rSO2_L, ABP & rSO2_R, CPP & rSO2_L, and CPP & rSO2_R. To compare the cerebral physiologic relationships between hemispheres for a signal combination such as ABP ® rSO2 signal directionality, the percentage of patients that showed a greater response than 0.1% for ABP ® rSO2_L and ABP ® rSO2_R were compared.

In the HC population, the sub-groups that had similar results, within 10% points, of comparing cerebral physiologic relationships between hemispheres for ABP & rSO2_L and ABP & rSO2_R signal combinations in the three data resolutions were Age < 40 years, Age > 60 years, Males, Females, and Right Hand Dominance. The sub-groups Age 40 – 60 years and Left Hand Dominance showed a regional hemispheric difference of greater than 10% points in all the signal combinations in the three data resolutions but contained less than 10 patients each. File S8y shows the sub-grouped Hemispheric Responsiveness using Impulse Response Coefficients of VAR model with Personalized and Capped VAR P-Orders in the three data resolutions for HC population.

The VAR IRF results for ABP & rSO2_L, ABP & rSO2_R signal combinations varied amongst sub-groups in the SP population where similar results, within 10% points, were mostly found in Age < 40 years, Males, Females, PCDF, Cervical Incision and Drain, Corpectomy, Laminectomy, Thoracic Decompression & Instrumental Fusion, Midazolam + Propofol + Remi-Fentanyl, Propofol + Remi-Fentanyl + Sufentanil, and Ketamine + Midazolam + Propofol + Remi-Fentanyl + Sufentanil sub-groups in the three data resolutions except for PCDF and Laminectomy sub-groups whose results changed to show a difference greater than 10% points in 1-minute data resolution. The sub-groups Age 40 – 60 years, Age > 60 years, ACDF, ACDF & PCDF, Propofol + Sufentanil, Ketamine + Propofol + Sufentanil, Midazolam + Propofol + Sufentanil, and Ketamine + Midazolam + Propofol + Sufentanil showed a regional hemispheric difference of greater than 10% points in all the signal combinations in the three data resolutions except for Ketamine + Propofol + Sufentanil, and Midazolam + Propofol + Sufentanil whose results changed in 1-minute data resolution to have a difference of less than 10% points. File S8z shows the sub-grouped Hemispheric Responsiveness using Impulse Response Coefficients of VAR model with Personalized and Capped VAR P-Orders in the three data resolutions for SP population.

In the TBI-GLR population, the sub-groups with mostly bilaterally similar cerebral physiologic relationships for all the signal combinations were Age < 40 years, Age 40 – 60 years, Age > 60 years, Males, Females, Focal Injury, Diffuse Injury, Marshall CT V – II, Rotterdam CT 6 – 1, and anesthetic regimens in the three data resolutions except for sub-groups Age < 40 years, Females, Diffuse Injury, Marshall CT V – III, Rotterdam CT 6 – 3, Propofol, Fentanyl + Ketamine + Propofol, and Fentanyl + Ketamine + Midazolam + Propofol whose results changed to show difference greater than 10% points in the 1-minute data resolution. File S8aa shows the sub-grouped Hemispheric Responsiveness using Impulse Response Coefficients of VAR model with Personalized and Capped VAR P-Orders in the three data resolutions for TBI-GLR population.

For the TBI-GL population, the sub-groups with bilaterally similar cerebral physiologic relationships for the signal combinations, within 10% points, were Age > 60 years, Females, Focal Injury, Marshall CT II, Fentanyl + Ketamine + Propofol, and Fentanyl + Ketamine + Midazolam + Propofol. The sub-groups with bilaterally dissimilar cerebral physiologic relationships were Age < 40 years, Age 40 – 60 years, Males, Diffuse Injury, Marshall CT V – III, Rotterdam CT 6 – 3, Propofol, Fentanyl + Propofol, and Fentanyl + Midazolam + Propofol. File S8bb shows the sub-grouped Hemispheric Responsiveness using Impulse Response Coefficients of VAR model with Personalized and Capped VAR P-Orders in the three data resolutions for TBI-GL population.

In the TBI-GR population, the Females, Diffuse Injury, Marshall CT IV & II, Rotterdam CT 2, and Fentanyl + Midazolam + Propofol sub-groups had bilaterally similar results for most of the signal combinations. The sub-groups Age < 40 years, Age 40 – 60 years, Age > 60 years, Focal Injury, Marshall CT V & IV, Rotterdam CT 6 – 4, Propofol, and Fentanyl + Propofol contained bilaterally dissimilar cerebral physiologic relationships for the signal combinations. File S8cc shows the sub-grouped Hemispheric Responsiveness using Impulse Response Coefficients of VAR model with Personalized and Capped VAR P-Orders in the three data resolutions for TBI-GL population.

For the TBI-BLR population, most of the sub-groups showed bilaterally similar cerebral physiologic relationship results for most of the signal combinations. File S8dd shows the sub-grouped Hemispheric Responsiveness using Impulse Response Coefficients of VAR model with Personalized and Capped VAR P-Orders in the three data resolutions for TBI-GL population.

File S8f: Sub-grouped Hemispheric Difference in Granger Causality Between ABP/CPP and rSO2 – 10-second, 1-minute, 5-minute Data

The Granger Causality Assessment was performed in a similar fashion for the sub-group analysis with the magnitudes of F-statistic used to identify the direction of Granger causal relationship between two signal separately in the sub-group. In all the HC sub-groups, the Granger causal relationship of the following combinations were greater than their reverse direction using the 10-second data resolution: ABP ® rSO2_L and ABP ® rSO2_R but the reverse direction started to have greater causal relationship in the 1-minute data resolution for Age < 40 years, Age 40 – 60 years, and Females sub-groups. Also, most of the sub-groups showed a similar percentage, within 10% points, between hemispheres of the number of patients for each combination directionality of ABP and rSO2L/R while two sub-groups had a greater than 10% points difference between hemispheres but did not include more than 10 patients. The File S8ee shows the Granger causal directionality results using the three data resolutions for HC population.

Most of the SP sub-groups showed a greater Granger causal relationship for the ABP ® rSO2_L and ABP ® rSO2_R combinations than their reverse direction in the 10-second data resolution. When looking at the 1-minute data resolution, some sub-groups started to show a greater Granger causal relationship in the reverse direction for one or both sides and they were Age 40 – 60 years, PCDF, and Ketamine + Midazolam + Propofol + Sufentanil. Also, half of the sub-groups showed a similar percentage, within 10% points, between hemispheres of the number of patients for each combination directionality of ABP and rSO2L/R while the other half of sub-groups which had a greater than 10% points difference between hemispheres and included more than 10 patients was only PCDF. The File S8ff shows the Granger causal directionality results using the three data resolutions for SP population.

The TBI-GLR sub-groups all showed a greater Granger causal relationship for the ABP ® rSO2_L, ABP ® rSO2_R, CPP ® rSO2_L, and CPP ® rSO2_R combinations than their reverse direction in the 10-second data resolution. The reverse direction of these combinations started to be greater in most of the sub-groups when using 1-minute data resolution. Also, most of the sub-groups showed a similar percentage, within 10% points, between hemispheres of the number of patients for each combination directionality of ABP/CPP and rSO2L/R while the sub-groups which had a greater than 10% points difference between hemispheres and included more than 10 patients were Age greater than 60 years, and Rotterdam CT (6, 5, 4). The File S8gg shows the Granger causal directionality results using the three data resolutions for TBI-GLR population.

Most of the TBI-GL sub-groups showed a greater Granger causal relationship for the ABP ® rSO2_L, ABP ® rSO2_R, CPP ® rSO2_L, and CPP ® rSO2_R combinations than their reverse direction in the 10-second data resolution. The reverse direction of these combinations started to be greater in a few of the sub-groups when using 1-minute data resolution. Also, there was a difference greater than 10% points between hemispheres of the number of patients for each combination directionality of ABP/CPP and rSO2L/R in most of the TBI-GL sub-groups. The File S8hh shows the Granger causal directionality results using the three data resolutions for TBI-GL population.

In the TBI-GR population, all of the sub-groups showed a greater Granger causal relationship for the ABP ® rSO2_R and CPP ® rSO2_R combinations than their reverse direction in the 10-second data resolution. The sub-groups were split into half that showed a greater Granger causal relationship for the ABP ® rSO2_L and CPP ® rSO2_L combinations (Age < 40 years, Age > 60 years, Females, Focal Injury, Marhsall CT V, Rotterdam CT 6 & 5, Propofol, and Fentanyl + Propofol) and other half showed greater Granger causal relationship in the reverse direction (Age 40 – 60 years, Males, Diffuse Injury, Marshal CT IV & II, Rotterdam CT 4 & 2, and Fentanyl + Midazolam + Propofol). The results using 1-minute data resolution, were similar but some sub-groups started to show a greater Granger causal relationship for the reverse direction of rSO2_R which were already showing greater relationship for the reverse direction of rSO2_L. Also, there was a difference greater than 10% points between hemispheres of the number of patients for each combination directionality of ABP/CPP and rSO2L/R in most of the sub-groups. The File S8ii shows the Granger causal directionality results using the three data resolutions for TBI-GR population.

The directionality of greater Granger causal relationships in the TBI-BLR population varied across the sub-groups for the ABP/CPP to or from rSO2_L/R combinations in both data resolutions. Also, there was a difference greater than 10% points between hemispheres of the number of patients for each combination directionality of ABP/CPP and rSO2L/R in most of the sub-groups. The File S8jj shows the Granger causal directionality results using both data resolutions for TBI-BLR population.

File S8g: Sub-grouped Physiologic Results using 10-Second, 1-Minute, and 5-Minute Data Resolutions for HC Population

| **Physiologic Variable** | **Sub-groups** | | | | | | | | | | | | | |
| --- | --- | --- | --- | --- | --- | --- | --- | --- | --- | --- | --- | --- | --- | --- |
|  | **Age < 40 [n = 95]** | | **Age 40 – 60 [n = 6]** | | **Age > 60 [n = 1]** | | **Males [n = 42]** | | **Females [n = 60]** | | **Left Hand Dominance [n = 9]** | | **Right Hand Dominance [n = 93]** | |
|  | **Median (IQR) or  Median (IQR; MAD)** | **p-value** | **Median (IQR) or  Median (IQR; MAD)** | **p-value** | **Median (IQR) or  Median (IQR; MAD)** | **p-value** | **Median (IQR) or  Median (IQR; MAD)** | **p-value** | **Median (IQR) or  Median (IQR; MAD)** | **p-value** | **Median (IQR) or  Median (IQR; MAD)** | **p-value** | **Median (IQR) or  Median (IQR; MAD)** | **p-value** |
| **10-Second Data Resolution** | | | | | | | | | | | | | | |
| **ABP (mmHg)** | 103.45 (97.74 – 107.88) | – | 91.14 (86.54 – 96.29) | – | 83.25 (79.51 – 87.69) | – | 109.1 (104.59 – 113.73) | – | 98.69 (94.72 – 102.65) | – | 103.45 (99.51 – 106.68) | – | 102.55 (97.34 – 107.35) | – |
| **rSO2_L (%)** | 74 (72.97 – 74.99) | 0.2735 | 68.44 (67.47 – 69.23) | 0.3939 | 74 (73.03 – 74.99) | 1 | 78.31 (77.5 – 78.99) | 0.8161 | 71.11 (70.26 – 72.2) | 0.08 | 74.02 (73.18 – 75.27) | 0.6665 | 72.99 (71.95 – 74) | 0.3109 |
| **rSO2_R (%)** | 72.48 (71.53 – 74) |  | 62.09 (60.78 – 63.49) |  | 73.39 (71.56 – 74.26) |  | 78 (76.82 – 79.07) |  | 68.21 (67.4 – 69.05) |  | 70.69 (70.24 – 71.28) |  | 72.35 (71.5 – 73.72) |  |
| **COx-a_L (au)** | 0.14 (-0.08 – 0.34) | 0.6405 | 0.12 (-0.14 – 0.26) | 0.6991 | 0.26 (-0.01 – 0.45) | 1 | 0.11 (-0.08 – 0.33) | 0.9894 | 0.16 (-0.08 – 0.35) | 0.5271 | 0.09 (-0.14 – 0.31) | 0.1359 | 0.14 (-0.08 – 0.35) | 0.836 |
| **COx-a_R (au)** | 0.13 (-0.09 – 0.33) |  | 0.07 (-0.13 – 0.22) |  | 0.12 (-0.08 – 0.43) |  | 0.1 (-0.08 – 0.33) |  | 0.13 (-0.11 – 0.31) |  | 0.05 (-0.15 – 0.28) |  | 0.13 (-0.08 – 0.33) |  |
| **MAD of ABP (mmHg)** | 7.81 (5.63 – 10.06) | – | 5.75 (5.43 – 9.17) | – | 6 (6 – 6) | – | 6.75 (5.24 – 9.18) | – | 8.15 (5.99 – 10.8) | – | 7.12 (5.37 – 9.79) | – | 7.56 (5.62 – 10.23) | – |
| **MAD of rSO2_L (%)** | 1.44 (1.06 – 1.69) | 0.2576 | 1.31 (1.13 – 1.55) | 0.3095 | 1.47 (1.47 – 1.47) | 1 | 1.48 (1.05 – 1.69) | 0.4104 | 1.38 (1.1 – 1.68) | 0.2669 | 1.44 (1.17 – 1.48) | 0.8633 | 1.47 (1.08 – 1.69) | 0.1656 |
| **MAD of rSO2_R (%)** | 1.45 (1.2 – 1.82) |  | 1.48 (1.4 – 1.8) |  | 1.95 (1.95 – 1.95) |  | 1.48 (1.28 – 1.94) |  | 1.46 (1.19 – 1.72) |  | 1.39 (1.02 – 1.87) |  | 1.47 (1.28 – 1.85) |  |
| **MAD of COx-a_L (au)** | 0.28 (0.22 – 0.34) | 0.4348 | 0.3 (0.26 – 0.35) | 0.2403 | 0.3 (0.3 – 0.3) | 1 | 0.29 (0.23 – 0.38) | 0.2871 | 0.28 (0.23 – 0.33) | 0.5798 | 0.25 (0.24 – 0.3) | 0.6665 | 0.29 (0.22 – 0.35) | 0.2082 |
| **MAD of COx-a_R (au)** | 0.27 (0.23 – 0.33) |  | 0.21 (0.19 – 0.21) |  | 0.38 (0.38 – 0.38) |  | 0.26 (0.23 – 0.34) |  | 0.27 (0.23 – 0.33) |  | 0.27 (0.25 – 0.33) |  | 0.26 (0.23 – 0.33) |  |
| **% time rSO2_L > 60%** | 100 (100 – 100; 0) | 0.1359 | 100 (79.44 – 100; 0) | 0.5914 | 100 (100 – 100; 0) | – | 100 (100 – 100; 0) | 1 | 100 (100 – 100; 0) | 0.1011 | 100 (100 – 100; 0) | **0.0339** | 100 (100 – 100; 0) | 0.3882 |
| **% time rSO2_R > 60%** | 100 (100 – 100; 0) |  | 64.64 (7.72 – 100; 52.42) |  | 100 (100 – 100; 0) |  | 100 (100 – 100; 0) |  | 100 (97.4 – 100; 0) |  | 100 (97.25 – 100; 0) |  | 100 (100 – 100; 0) |  |
| **% time rSO2_L > 70%** | 100 (55.08 – 100; 0) | 0.1197 | 19.01 (0 – 62.54; 28.18) | 0.7973 | 100 (100 – 100; 0) | 1 | 100 (96.57 – 100; 0) | 0.5953 | 79.49 (1.83 – 100; 30.41) | **0.041** | 100 (99.12 – 100; 0) | 0.2065 | 97.16 (42.11 – 100; 4.21) | 0.2026 |
| **% time rSO2_R > 70%** | 95 (2.29 – 100; 7.41) |  | 0.29 (0 – 16.85; 0.43) |  | 91.67 (91.67 – 91.67; 0) |  | 100 (94.42 – 100; 0) |  | 8.1 (0 – 96; 12.01) |  | 81.73 (0 – 100; 27.09) |  | 93.37 (1.1 – 100; 9.83) |  |
| **% time rSO2_L > 80%** | 0 (0 – 36.03; 0) | 0.9904 | 0 (0 – 0; 0) | – | 0 (0 – 0; 0) | – | 6.11 (0 – 98.67; 9.06) | 0.7506 | 0 (0 – 0; 0) | 0.971 | 0 (0 – 0; 0) | 0.2752 | 0 (0 – 38.41; 0) | 0.7745 |
| **% time rSO2_R > 80%** | 0 (0 – 30.54; 0) |  | 0 (0 – 0; 0) |  | 0 (0 – 0; 0) |  | 7.85 (0 – 70.55; 11.63) |  | 0 (0 – 0; 0) |  | 0 (0 – 5.29; 0) |  | 0 (0 – 23.43; 0) |  |
| **% time rSO2_L > 90%** | 0 (0 – 0; 0) | 0.5446 | 0 (0 – 0; 0) | – | 0 (0 – 0; 0) | – | 0 (0 – 0; 0) | 0.7355 | 0 (0 – 0; 0) | **0.0437** | 0 (0 – 0; 0) | – | 0 (0 – 0; 0) | 0.5453 |
| **% time rSO2_R > 90%** | 0 (0 – 0; 0) |  | 0 (0 – 0; 0) |  | 0 (0 – 0; 0) |  | 0 (0 – 0; 0) |  | 0 (0 – 0; 0) |  | 0 (0 – 0; 0) |  | 0 (0 – 0; 0) |  |
| **% time COx-a_L > 0** | 67.66 (51.27 – 80.73; 21.24) | 0.507 | 62.61 (48.75 – 69.39; 13.72) | 0.8182 | 74.7 (74.7 – 74.7; 0) | 1 | 66.34 (47.59 – 75.38; 23.61) | 0.9857 | 67.98 (57.23 – 80.93; 18.55) | 0.4529 | 63.7 (57.99 – 73.99; 15.25) | 0.2224 | 67.7 (50.76 – 79.9; 20.59) | 0.7243 |
| **% time COx-a_R > 0** | 64.1 (54.05 – 77.99; 18.46) |  | 56.89 (53.18 – 67.69; 13.4) |  | 62.65 (62.65 – 62.65; 0) |  | 61.05 (50.07 – 75.98; 19.04) |  | 64.09 (56.48 – 78.29; 14.92) |  | 56.52 (54.44 – 62.5; 8.86) |  | 66.18 (53.66 – 78.15; 18.57) |  |
| **% time COx-a_L > 0.2** | 43.04 (28.73 – 57.61; 22.47) | 0.5483 | 31.3 (16.58 – 43.7; 23.34) | 0.9372 | 53.01 (53.01 – 53.01; 0) | 1 | 36.83 (24.06 – 55.64; 24.13) | 0.9185 | 45.17 (27.87 – 55.43; 21.98) | 0.4657 | 33.33 (29.58 – 49.71; 19.77) | 0.8633 | 43.04 (27.39 – 56.52; 23.21) | 0.6959 |
| **% time COx-a_R > 0.2** | 39.16 (30.6 – 53.73; 17.41) |  | 27.51 (18.09 – 37.1; 17.89) |  | 45.78 (45.78 – 45.78; 0) |  | 38.54 (26.67 – 49.38; 17.13) |  | 40.3 (29.97 – 55.61; 19.3) |  | 33.54 (31.02 – 35.45; 3.74) |  | 39.69 (28.42 – 55.22; 20.17) |  |
| **% time COx-a_L > 0.3** | 29.17 (17.97 – 41.95; 18.01) | 0.5197 | 19.13 (9.6 – 31.63; 15.27) | 0.4225 | 47.59 (47.59 – 47.59; 0) | 1 | 28.88 (13.84 – 40.46; 20.08) | 0.9358 | 29.69 (19.66 – 42.29; 17.06) | 0.3793 | 25.93 (10 – 29.48; 23.52) | 0.8633 | 29.65 (18.66 – 42.24; 18.34) | 0.5159 |
| **% time COx-a_R > 0.3** | 27.72 (16.45 – 40.15; 17.83) |  | 11.62 (7.51 – 23.05; 7.88) |  | 42.17 (42.17 – 42.17; 0) |  | 27.03 (14.97 – 36.27; 17.71) |  | 26.81 (16.29 – 43.63; 19.08) |  | 23.6 (16.36 – 25.9; 10.28) |  | 28.75 (15.49 – 43.28; 19.94) |  |
| **1-Minute Data Resolution** | | | | | | | | | | | | | | |
| **ABP (mmHg)** | 102.63 (98.97 – 105.53) | – | 91.24 (86.41 – 96.37) | – | 83.56 (80.79 – 86.82) | – | 109.45 (105.51 – 111.62) | – | 99.2 (95.3 – 102.09) | – | 102.63 (101.32 – 105.48) | – | 102.25 (97.92 – 105.53) | – |
| **rSO2_L (%)** | 73.84 (72.86 – 74.93) | 0.2516 | 68.3 (67.6 – 69.14) | 0.3939 | 74.35 (73.1 – 74.8) | 1 | 78.3 (77.51 – 78.82) | 0.7792 | 71.15 (70.49 – 71.84) | 0.0765 | 74.05 (73.42 – 75.1) | 0.6665 | 73.02 (72.16 – 73.89) | 0.2789 |
| **rSO2_R (%)** | 72.48 (71.78 – 73.63) |  | 62.27 (61.07 – 63.43) |  | 73.39 (71.31 – 74.16) |  | 77.97 (76.94 – 78.94) |  | 68.23 (67.75 – 68.98) |  | 70.72 (70.23 – 71.13) |  | 72.35 (71.61 – 73.48) |  |
| **COx-a_L (au)** | 0.13 (-0.06 – 0.32) | 0.5977 | 0.13 (-0.11 – 0.24) | 0.6991 | 0.27 (-0.02 – 0.44) | 1 | 0.11 (-0.07 – 0.32) | 0.9681 | 0.15 (-0.03 – 0.33) | 0.5271 | 0.13 (-0.13 – 0.31) | 0.0625 | 0.13 (-0.05 – 0.33) | 0.8745 |
| **COx-a_R (au)** | 0.12 (-0.07 – 0.31) |  | 0.09 (-0.11 – 0.2) |  | 0.11 (-0.07 – 0.41) |  | 0.1 (-0.08 – 0.3) |  | 0.14 (-0.07 – 0.3) |  | 0.05 (-0.13 – 0.27) |  | 0.13 (-0.06 – 0.31) |  |
| **MAD of ABP (mmHg)** | 5.75 (3.97 – 8.85) | – | 5.59 (4.08 – 8.85) | – | 4.56 (4.56 – 4.56) | – | 5.04 (3.81 – 8.09) | – | 6.36 (4.36 – 9.22) | – | 7.51 (3.93 – 8.85) | – | 5.72 (4.22 – 8.85) | – |
| **MAD of rSO2_L (%)** | 1.17 (0.88 – 1.59) | 0.1853 | 1.21 (0.82 – 1.49) | 0.4848 | 1.53 (1.53 – 1.53) | 1 | 1.3 (0.83 – 1.61) | 0.385 | 1.1 (0.9 – 1.52) | 0.2264 | 1.25 (0.78 – 1.59) | 0.7962 | 1.17 (0.9 – 1.57) | 0.1384 |
| **MAD of rSO2_R (%)** | 1.22 (0.96 – 1.66) |  | 1.28 (1.07 – 1.85) |  | 1.74 (1.74 – 1.74) |  | 1.3 (1 – 1.79) |  | 1.19 (0.96 – 1.65) |  | 0.99 (0.75 – 1.78) |  | 1.23 (1 – 1.67) |  |
| **MAD of COx-a_L (au)** | 0.28 (0.22 – 0.35) | 0.1956 | 0.3 (0.27 – 0.33) | 0.3095 | 0.28 (0.28 – 0.28) | 1 | 0.28 (0.22 – 0.37) | 0.2306 | 0.28 (0.22 – 0.33) | 0.3302 | 0.23 (0.21 – 0.33) | 1 | 0.28 (0.23 – 0.35) | 0.1039 |
| **MAD of COx-a_R (au)** | 0.26 (0.21 – 0.32) |  | 0.2 (0.17 – 0.22) |  | 0.36 (0.36 – 0.36) |  | 0.26 (0.2 – 0.31) |  | 0.25 (0.22 – 0.32) |  | 0.25 (0.22 – 0.3) |  | 0.25 (0.21 – 0.32) |  |
| **% time rSO2_L > 60%** | 100 (100 – 100; 0) | 0.0745 | 100 (83.06 – 100; 0) | 0.5284 | 100 (100 – 100; 0) | – | 100 (100 – 100; 0) | 0.329 | 100 (100 – 100; 0) | 0.0968 | 100 (100 – 100; 0) | **0.0339** | 100 (100 – 100; 0) | 0.2721 |
| **% time rSO2_R > 60%** | 100 (100 – 100; 0) |  | 63.33 (6.67 – 100; 54.36) |  | 100 (100 – 100; 0) |  | 100 (100 – 100; 0) |  | 100 (97.38 – 100; 0) |  | 100 (96.67 – 100; 0) |  | 100 (100 – 100; 0) |  |
| **% time rSO2_L > 70%** | 100 (60.39 – 100; 0) | **0.0346** | 21.43 (0 – 68.21; 31.77) | 0.5914 | 100 (100 – 100; 0) | 1 | 100 (100 – 100; 0) | 0.4119 | 87.07 (0 – 100; 19.17) | **0.0159** | 100 (100 – 100; 0) | 0.1722 | 100 (42.86 – 100; 0) | 0.0682 |
| **% time rSO2_R > 70%** | 96.55 (0 – 100; 5.11) |  | 0 (0 – 19.29; 0) |  | 96.67 (96.67 – 96.67; 0) |  | 100 (96.74 – 100; 0) |  | 3.54 (0 – 100; 5.24) |  | 88.24 (0 – 100; 17.44) |  | 96.43 (0 – 100; 5.3) |  |
| **% time rSO2_L > 80%** | 0 (0 – 36.71; 0) | 0.6774 | 0 (0 – 0; 0) | – | 0 (0 – 0; 0) | – | 4.54 (0 – 100; 6.73) | 0.4352 | 0 (0 – 0; 0) | 0.976 | 0 (0 – 0; 0) | 0.4968 | 0 (0 – 42; 0) | 0.5426 |
| **% time rSO2_R > 80%** | 0 (0 – 29.29; 0) |  | 0 (0 – 0; 0) |  | 0 (0 – 0; 0) |  | 1.79 (0 – 72.54; 2.65) |  | 0 (0 – 0; 0) |  | 0 (0 – 0; 0) |  | 0 (0 – 25.93; 0) |  |
| **% time rSO2_L > 90%** | 0 (0 – 0; 0) | 0.8368 | 0 (0 – 0; 0) | – | 0 (0 – 0; 0) | – | 0 (0 – 0; 0) | 0.9067 | 0 (0 – 0; 0) | 0.3254 | 0 (0 – 0; 0) | – | 0 (0 – 0; 0) | 0.8377 |
| **% time rSO2_R > 90%** | 0 (0 – 0; 0) |  | 0 (0 – 0; 0) |  | 0 (0 – 0; 0) |  | 0 (0 – 0; 0) |  | 0 (0 – 0; 0) |  | 0 (0 – 0; 0) |  | 0 (0 – 0; 0) |  |
| **% time COx-a_L > 0** | 67.86 (51.79 – 81.37; 20.77) | 0.4803 | 63.12 (52.68 – 69.5; 15.44) | 0.9372 | 71.43 (71.43 – 71.43; 0) | 1 | 66.67 (50 – 75.65; 22.04) | 0.8615 | 68.41 (53.78 – 81.57; 20.65) | 0.4481 | 60 (53.57 – 75.86; 9.88) | 0.5953 | 67.86 (50 – 81.25; 20.7) | 0.6181 |
| **% time COx-a_R > 0** | 65.79 (54.36 – 78.47; 17.77) |  | 57.08 (53.39 – 68.57; 13.57) |  | 60.71 (60.71 – 60.71; 0) |  | 61.11 (48.79 – 75.42; 19.03) |  | 65.79 (57.14 – 78.33; 15.45) |  | 59.26 (53.57 – 61.11; 8.43) |  | 66.67 (54.17 – 79.17; 18.53) |  |
| **% time COx-a_L > 0.2** | 42.11 (26.79 – 57.14; 22.51) | 0.5352 | 28.75 (15.71 – 43; 22.64) | 0.9372 | 50 (50 – 50; 0) | 1 | 38.75 (22.1 – 55.7; 26.09) | 0.918 | 43.96 (26.86 – 55.19; 22.01) | 0.5426 | 35.71 (27.78 – 51.85; 15.89) | 0.5457 | 42.11 (26.32 – 57.14; 22.89) | 0.6938 |
| **% time COx-a_R > 0.2** | 40.63 (29.06 – 52.4; 17.31) |  | 26.79 (18.75 – 30.58; 10.81) |  | 46.43 (46.43 – 46.43; 0) |  | 38.68 (26.25 – 49.11; 16.79) |  | 43.1 (28.57 – 54.09; 20.81) |  | 32.14 (29.63 – 38.89; 5.29) |  | 41.67 (26.67 – 53.85; 18.86) |  |
| **% time COx-a_L > 0.3** | 28.95 (15.59 – 43.93; 20.62) | 0.5128 | 20.83 (11.16 – 30.57; 15.06) | 0.4696 | 50 (50 – 50; 0) | 1 | 28.76 (12.71 – 42.19; 22.82) | 0.9679 | 30.03 (19.23 – 43.43; 19.01) | 0.3652 | 28.57 (6.45 – 30.43; 31.77) | 0.8252 | 29.17 (15.79 – 44.12; 20.78) | 0.483 |
| **% time COx-a_R > 0.3** | 26.92 (14.72 – 36.43; 17.11) |  | 11.61 (8.04 – 21.88; 6.97) |  | 46.43 (46.43 – 46.43; 0) |  | 27.19 (12.71 – 35.12; 18.07) |  | 26.57 (14.29 – 38.04; 18.21) |  | 21.43 (11.11 – 22.22; 13.85) |  | 28.13 (14.29 – 40.74; 20.48) |  |
| **5-Minute Data Resolution** | | | | | | | | | | | | | | |
| **ABP (mmHg)** | 103.18 (99.47 – 105.44) | – | 90.58 (87.78 – 95.46) | – | 84.54 (81.5 – 85.71) | – | 109.81 (106.5 – 111.77) | – | 99.95 (96.03 – 100.71) | – | 102.84 (101.72 – 107.23) | – | 102.87 (98.99 – 104.73) | – |
| **rSO2_L (%)** | 73.91 (73.07 – 74.43) | 0.2341 | 68.38 (67.97 – 69.07) | 0.3939 | 74.11 (73.39 – 74.41) | 1 | 78.35 (77.54 – 78.72) | 0.7252 | 71.19 (70.71 – 71.74) | 0.0698 | 73.99 (73.51 – 75.02) | 0.6048 | 73.06 (72.3 – 73.71) | 0.2688 |
| **rSO2_R (%)** | 72.74 (72.48 – 73.5) |  | 62.57 (61.75 – 62.84) |  | 73.59 (71.6 – 73.98) |  | 78.08 (77.37 – 78.72) |  | 68.12 (67.74 – 68.68) |  | 70.59 (70.45 – 70.89) |  | 72.66 (71.92 – 73.06) |  |
| **COx-a_L (au)** | 0.13 (0.02 – 0.25) | 0.3868 | 0.09 (-0.11 – 0.16) | 0.9372 | 0.24 (-0.04 – 0.39) | 1 | 0.14 (0 – 0.22) | 0.7861 | 0.13 (0.02 – 0.27) | 0.4025 | 0.13 (-0.06 – 0.19) | 0.1135 | 0.13 (0.02 – 0.26) | 0.6086 |
| **COx-a_R (au)** | 0.12 (0.01 – 0.25) |  | 0.07 (-0.12 – 0.18) |  | 0.14 (-0.01 – 0.33) |  | 0.09 (0.01 – 0.22) |  | 0.14 (-0.01 – 0.25) |  | 0.06 (-0.04 – 0.14) |  | 0.14 (0.01 – 0.25) |  |
| **MAD of ABP (mmHg)** | 5.12 (2.7 – 7.6) | – | 4.21 (3.48 – 4.37) | – | 3.76 (3.76 – 3.76) | – | 4.87 (2.73 – 6.64) | – | 4.62 (2.79 – 7.49) | – | 5.12 (2.71 – 7.07) | – | 4.69 (2.79 – 7.39) | – |
| **MAD of rSO2_L (%)** | 0.83 (0.46 – 1.23) | 0.3544 | 0.87 (0.62 – 1.38) | 0.4848 | 0.93 (0.93 – 0.93) | 1 | 0.76 (0.54 – 1.23) | 0.1706 | 0.85 (0.46 – 1.22) | 0.8811 | 0.84 (0.74 – 0.93) | 0.9314 | 0.83 (0.46 – 1.25) | 0.2492 |
| **MAD of rSO2_R (%)** | 0.86 (0.45 – 1.46) |  | 1 (0.66 – 1.71) |  | 1.12 (1.12 – 1.12) |  | 1.02 (0.53 – 1.59) |  | 0.72 (0.45 – 1.32) |  | 0.8 (0.34 – 1.33) |  | 0.88 (0.47 – 1.52) |  |
| **MAD of COx-a_L (au)** | 0.2 (0.14 – 0.28) | 0.1717 | 0.17 (0.12 – 0.31) | 0.5887 | 0.27 (0.27 – 0.27) | 1 | 0.19 (0.15 – 0.28) | 0.0901 | 0.2 (0.12 – 0.28) | 0.587 | 0.21 (0.15 – 0.26) | 0.5457 | 0.2 (0.13 – 0.28) | 0.1767 |
| **MAD of COx-a_R (au)** | 0.17 (0.12 – 0.26) |  | 0.14 (0.11 – 0.2) |  | 0.27 (0.27 – 0.27) |  | 0.16 (0.11 – 0.25) |  | 0.17 (0.13 – 0.26) |  | 0.16 (0.11 – 0.22) |  | 0.17 (0.12 – 0.26) |  |
| **% time rSO2_L > 60%** | 100 (100 – 100; 0) | 0.1982 | 100 (87.5 – 100; 0) | 0.5284 | 100 (100 – 100; 0) | – | 100 (100 – 100; 0) | 0.329 | 100 (100 – 100; 0) | 0.2338 | 100 (100 – 100; 0) | 0.1693 | 100 (100 – 100; 0) | 0.3417 |
| **% time rSO2_R > 60%** | 100 (100 – 100; 0) |  | 58.33 (4.17 – 100; 61.78) |  | 100 (100 – 100; 0) |  | 100 (100 – 100; 0) |  | 100 (100 – 100; 0) |  | 100 (100 – 100; 0) |  | 100 (100 – 100; 0) |  |
| **% time rSO2_L > 70%** | 100 (58.33 – 100; 0) | 0.0997 | 20 (0 – 60; 29.65) | 0.5914 | 100 (100 – 100; 0) | – | 100 (100 – 100; 0) | 0.8478 | 100 (0 – 100; 0) | **0.0274** | 100 (100 – 100; 0) | 0.3587 | 100 (40 – 100; 0) | 0.1532 |
| **% time rSO2_R > 70%** | 100 (0 – 100; 0) |  | 0 (0 – 10.71; 0) |  | 100 (100 – 100; 0) |  | 100 (100 – 100; 0) |  | 0 (0 – 100; 0) |  | 100 (0 – 100; 0) |  | 100 (0 – 100; 0) |  |
| **% time rSO2_L > 80%** | 0 (0 – 29.29; 0) | 0.8617 | 0 (0 – 0; 0) | – | 0 (0 – 0; 0) | – | 0 (0 – 100; 0) | 0.5986 | 0 (0 – 0; 0) | 0.8273 | 0 (0 – 0; 0) | 0.1693 | 0 (0 – 30; 0) | 0.6208 |
| **% time rSO2_R > 80%** | 0 (0 – 26.67; 0) |  | 0 (0 – 0; 0) |  | 0 (0 – 0; 0) |  | 0 (0 – 80.36; 0) |  | 0 (0 – 0; 0) |  | 0 (0 – 0; 0) |  | 0 (0 – 20; 0) |  |
| **% time rSO2_L > 90%** | 0 (0 – 0; 0) | 0.5564 | 0 (0 – 0; 0) | – | 0 (0 – 0; 0) | – | 0 (0 – 0; 0) | 0.344 | 0 (0 – 0; 0) | 0.3254 | 0 (0 – 0; 0) | – | 0 (0 – 0; 0) | 0.5562 |
| **% time rSO2_R > 90%** | 0 (0 – 0; 0) |  | 0 (0 – 0; 0) |  | 0 (0 – 0; 0) |  | 0 (0 – 0; 0) |  | 0 (0 – 0; 0) |  | 0 (0 – 0; 0) |  | 0 (0 – 0; 0) |  |
| **% time COx-a_L > 0** | 71.43 (50 – 85.71; 31.77) | 0.74 | 65 (37.5 – 82.5; 37.07) | 0.8089 | 66.67 (66.67 – 66.67; 0) | 1 | 69.05 (50 – 83.33; 28.24) | 0.9569 | 75 (55.36 – 85.71; 24.36) | 0.5434 | 60 (50 – 80; 14.83) | 1 | 71.43 (50 – 85.71; 31.77) | 0.6899 |
| **% time COx-a_R > 0** | 71.43 (53.57 – 83.33; 17.65) |  | 55 (37.5 – 65; 24.71) |  | 50 (50 – 50; 0) |  | 71.43 (50 – 83.33; 31.77) |  | 68.33 (60 – 83.33; 17.3) |  | 66.67 (60 – 71.43; 9.88) |  | 71.43 (50 – 83.33; 21.18) |  |
| **% time COx-a_L > 0.2** | 37.5 (20 – 60; 30.89) | 0.6537 | 18.33 (16.67 – 42.5; 2.47) | 0.9347 | 50 (50 – 50; 0) | – | 33.33 (16.67 – 55.36; 24.71) | 0.8715 | 40 (20 – 57.86; 29.65) | 0.6677 | 28.57 (20 – 50; 17.65) | 0.2856 | 37.5 (16.67 – 57.14; 30.89) | 0.9325 |
| **% time COx-a_R > 0.2** | 37.5 (16.67 – 50; 29.12) |  | 26.67 (17.5 – 33.33; 12.36) |  | 50 (50 – 50; 0) |  | 33.33 (16.67 – 55.36; 24.71) |  | 40 (20 – 50; 27.53) |  | 20 (0 – 33.33; 29.65) |  | 37.5 (16.67 – 50; 29.12) |  |
| **% time COx-a_L > 0.3** | 20 (0 – 42.86; 29.65) | 0.1776 | 18.33 (16.67 – 42.5; 14.83) | 0.3227 | 50 (50 – 50; 0) | 1 | 20 (0 – 38.33; 29.65) | 0.268 | 29.29 (16.07 – 50; 30.71) | 0.2056 | 16.67 (0 – 20; 24.71) | 0.8545 | 20 (14.29 – 50; 29.65) | 0.1156 |
| **% time COx-a_R > 0.3** | 20 (0 – 33.33; 29.65) |  | 8.33 (0 – 29.17; 12.36) |  | 33.33 (33.33 – 33.33; 0) |  | 15.48 (0 – 33.33; 22.95) |  | 20 (14.29 – 33.33; 19.77) |  | 16.67 (0 – 20; 4.94) |  | 20 (0 – 33.33; 29.65) |  |
| *The p-values in the table are derived using Mann-Whitney U test between the bilateral signals.*  *ABP, arterial blood pressure; au, arbitrary units; CPP, cerebral perfusion pressure; COx, cerebral oximetry index with CPP; COx-a, cerebral oximetry index with ABP; MAD, median absolute deviation; HC, healthy control volunteer group; IQR, interquartile range; mmHg, millimeters of mercury; rSO2, regional cerebral oxygen saturation.* | | | | | | | | | | | | | | |

File S8h: Sub-grouped Physiologic Results using 10-Second, 1-Minute, and 5-Minute Data Resolutions for SP Population

| **Physiologic Variable** | **Sub-groups** | | | | | | | | | | | | | | | | | |
| --- | --- | --- | --- | --- | --- | --- | --- | --- | --- | --- | --- | --- | --- | --- | --- | --- | --- | --- |
|  | **Age < 40 [n = 1]** | | **Age 40 – 60 [n = 17]** | | **Age > 60 [n = 9)]** | | **Males [n = 22)]** | | **Females [n = 5]** | | **ACDF [n = 6]** | | **PCDF [n = 25]** | | **ACDF & PCDF [n = 3]** | | **Cervical Incision and Drain [n = 1]** | |
|  | **Median (IQR) or  Median (IQR; MAD)** | **p-value** | **Median (IQR) or  Median (IQR; MAD)** | **p-value** | **Median (IQR) or  Median (IQR; MAD)** | **p-value** | **Median (IQR) or  Median (IQR; MAD)** | **p-value** | **Median (IQR) or  Median (IQR; MAD)** | **p-value** | **Median (IQR) or  Median (IQR; MAD)** | **p-value** | **Median (IQR) or  Median (IQR; MAD)** | **p-value** | **Median (IQR) or  Median (IQR; MAD)** | **p-value** | **Median (IQR) or  Median (IQR; MAD)** | **p-value** |
| **10-Second Data Resolution** | | | | | | | | | | | | | | | | | | |
| **ABP (mmHg)** | 80.6 (75.55 – 87.71) | – | 84.74 (77.01 – 88.93) | – | 83.83 (76.13 – 88.04) | – | 84.46 (77.33 – 89.61) | – | 78.68 (72.85 – 85.6) | – | 82.77 (76.85 – 90.11) | – | 83.03 (75.28 – 88.9) | – | 86.45 (81.63 – 92.64) | – | 83.87 (77.65 – 87.77) | – |
| **rSO2_L (%)** | 89.99 (76.99 – 93.44) | 1 | 66.26 (62.99 – 69) | 0.7305 | 62.99 (61.78 – 68.23) | 0.5076 | 66.4 (63.54 – 69.04) | 0.9626 | 60.99 (60.99 – 61.99) | 1 | 66.63 (61.99 – 68.58) | 0.9372 | 64.49 (62.39 – 70.5) | 1 | 75.99 (74 – 78) | 0.6579 | 66.54 (64.29 – 68.23) | 1 |
| **rSO2_R (%)** | 83.95 (68.49 – 87.99) |  | 65.07 (61.99 – 66.27) |  | 69 (67.66 – 71.59) |  | 68.2 (67.18 – 70.22) |  | 56.98 (56 – 58.99) |  | 70.67 (62.25 – 73.15) |  | 63.99 (62.49 – 65.5) |  | 74 (72.79 – 75.99) |  | 71.38 (67.99 – 74) |  |
| **COx-a_L (au)** | 0.1 (-0.08 – 0.42) | 1 | 0.17 (-0.11 – 0.43) | 0.3394 | 0.24 (-0.01 – 0.54) | 0.6665 | 0.15 (-0.1 – 0.43) | 0.3219 | 0.22 (-0.11 – 0.55) | 1 | 0.17 (-0.08 – 0.44) | 0.0931 | 0.19 (-0.19 – 0.54) | 0.91 | 0.17 (-0.01 – 0.43) | 1 | 0.26 (0.09 – 0.49) | 1 |
| **COx-a_R (au)** | 0.3 (-0.13 – 0.6) |  | 0.21 (-0.03 – 0.48) |  | 0.18 (-0.09 – 0.52) |  | 0.2 (-0.09 – 0.47) |  | 0.24 (-0.03 – 0.57) |  | 0.25 (-0.06 – 0.52) |  | 0.11 (-0.14 – 0.46) |  | 0.18 (-0.05 – 0.46) |  | 0.27 (0 – 0.52) |  |
| **MAD of ABP (mmHg)** | 9.52 (9.52 – 9.52) | – | 8.82 (5.85 – 10.22) | – | 8.07 (6.42 – 11.12) | – | 8.45 (5.87 – 10.46) | – | 9.62 (8.61 – 10.22) | – | 9.71 (9 – 10.39) | – | 9.41 (5.81 – 10.52) | – | 8.07 (6.94 – 11.17) | – | 7.34 (7.34 – 7.34) | – |
| **MAD of rSO2_L (%)** | 7.41 (7.41 – 7.41) | 1 | 2.97 (2.39 – 4) | 0.5016 | 3.04 (2.82 – 5.65) | 0.5658 | 3.1 (2.67 – 4.46) | 0.3914 | 2.39 (1.48 – 4.02) | 1 | 3.06 (2.71 – 3.44) | 1 | 3.98 (2.57 – 4.46) | 0.1781 | 2.95 (2.22 – 5.18) | 1 | 3.04 (3.04 – 3.04) | 1 |
| **MAD of rSO2_R (%)** | 7.47 (7.47 – 7.47) |  | 2.83 (1.5 – 3.48) |  | 2.94 (2.02 – 4.55) |  | 2.88 (1.63 – 3.79) |  | 3.22 (1.6 – 3.48) |  | 2.78 (2.68 – 3.43) |  | 3.09 (2.18 – 3.69) |  | 1.5 (1.5 – 4.46) |  | 4.55 (4.55 – 4.55) |  |
| **MAD of COx-a_L (au)** | 0.36 (0.36 – 0.36) | 1 | 0.44 (0.33 – 0.54) | 0.6543 | 0.45 (0.29 – 0.54) | 0.8633 | 0.42 (0.29 – 0.53) | 0.7014 | 0.48 (0.44 – 0.54) | 1 | 0.35 (0.3 – 0.44) | 0.8182 | 0.49 (0.39 – 0.55) | 0.9459 | 0.4 (0.35 – 0.42) | 0.7 | 0.29 (0.29 – 0.29) | 1 |
| **MAD of COx-a_R (au)** | 0.55 (0.55 – 0.55) |  | 0.45 (0.41 – 0.51) |  | 0.38 (0.32 – 0.56) |  | 0.43 (0.35 – 0.55) |  | 0.47 (0.45 – 0.51) |  | 0.43 (0.34 – 0.52) |  | 0.45 (0.37 – 0.58) |  | 0.43 (0.39 – 0.46) |  | 0.38 (0.38 – 0.38) |  |
| **% time rSO2_L > 60%** | 100 (100 – 100; 0) | 1 | 95.62 (65.68 – 99.76; 6.49) | 0.9862 | 99.05 (27.85 – 99.74; 1.41) | 0.6585 | 97.19 (66.32 – 99.84; 4.16) | 0.953 | 94.99 (13.47 – 99.71; 7.01) | 0.8413 | 97.67 (75.08 – 99.93; 3.46) | 0.5182 | 89.94 (16.1 – 99.54; 14.92) | 0.6609 | 99.74 (98.78 – 99.8; 0.17) | 1 | 99.79 (99.79 – 99.79; 0) | 1 |
| **% time rSO2_R > 60%** | 97.56 (97.56 – 97.56; 0) |  | 90.41 (51.38 – 99.92; 14.22) |  | 99.65 (40.42 – 99.76; 0.52) |  | 97.82 (66.1 – 99.89; 3.23) |  | 18.54 (15.45 – 99.76; 23.82) |  | 82.47 (66.1 – 99.33; 25.38) |  | 94.25 (43.16 – 99.75; 8.53) |  | 99 (98.24 – 99.48; 1.41) |  | 99.89 (99.89 – 99.89; 0) |  |
| **% time rSO2_L > 70%** | 96.75 (96.75 – 96.75; 0) | 1 | 14.64 (0.09 – 84.52; 21.71) | 0.8896 | 10.68 (5.7 – 36.23; 15.83) | 0.479 | 18.11 (3.39 – 94.09; 26.85) | 0.8783 | 0.09 (0 – 36.23; 0.14) | 0.5258 | 15.96 (2.99 – 77.96; 23.19) | 0.8726 | 23.88 (1.43 – 64.48; 35.41) | 0.711 | 86.1 (44.36 – 91.94; 17.32) | 0.7 | 7.71 (7.71 – 7.71; 0) | 1 |
| **% time rSO2_R > 70%** | 72.36 (72.36 – 72.36; 0) |  | 8.28 (0.39 – 95.21; 12.27) |  | 33.79 (12.31 – 51.78; 31.85) |  | 23.03 (3.7 – 68.1; 34.14) |  | 6.6 (0.39 – 59.45; 9.79) |  | 37.54 (0.97 – 89.49; 55.37) |  | 10.72 (2.1 – 39.5; 15.89) |  | 97.32 (74.55 – 98.37; 3.11) |  | 55.34 (55.34 – 55.34; 0) |  |
| **% time rSO2_L > 80%** | 70.62 (70.62 – 70.62; 0) | 1 | 0 (0 – 4.62; 0) | 0.9405 | 0 (0 – 11.93; 0) | 0.6279 | 0 (0 – 10.86; 0) | 0.8912 | 0 (0 – 0; 0) | 0.6072 | 2.19 (0 – 18.9; 3.25) | 0.9338 | 0 (0 – 7.17; 0) | 0.9573 | 7.65 (3.82 – 27.7; 11.34) | 1 | 0 (0 – 0; 0) | 1 |
| **% time rSO2_R > 80%** | 61.09 (61.09 – 61.09; 0) |  | 0.09 (0 – 1.19; 0.14) |  | 0.63 (0 – 1.71; 0.94) |  | 0.53 (0 – 1.65; 0.79) |  | 0 (0 – 0.18; 0) |  | 8.1 (0.02 – 20.72; 12.01) |  | 0 (0 – 1.44; 0) |  | 1.1 (0.76 – 20.31; 0.98) |  | 0.63 (0.63 – 0.63; 0) |  |
| **% time rSO2_L > 90%** | 49.48 (49.48 – 49.48; 0) | 1 | 0 (0 – 0; 0) | 0.6547 | 0 (0 – 0; 0) | 0.5867 | 0 (0 – 0; 0) | 0.7517 | 0 (0 – 0; 0) | 0.4237 | 0 (0 – 0.31; 0) | 0.6553 | 0 (0 – 0; 0) | 0.9699 | 0 (0 – 0; 0) | 0.505 | 0 (0 – 0; 0) | – |
| **% time rSO2_R > 90%** | 5.23 (5.23 – 5.23; 0) |  | 0 (0 – 0; 0) |  | 0 (0 – 0; 0) |  | 0 (0 – 0; 0) |  | 0 (0 – 0; 0) |  | 0.73 (0 – 4.29; 1.09) |  | 0 (0 – 0; 0) |  | 0 (0 – 2.93; 0) |  | 0 (0 – 0; 0) |  |
| **% time COx-a_L > 0** | 57.73 (57.73 – 57.73; 0) | 1 | 64.39 (55.48 – 70.1; 13.08) | 0.3223 | 66.12 (61.31 – 77.18; 15.69) | 0.6665 | 62.85 (55.5 – 72.37; 12.64) | 0.4355 | 66.05 (63.46 – 67.27; 3.84) | 0.8413 | 63.85 (58.4 – 69.39; 9.17) | 0.3939 | 63.13 (53.76 – 71.54; 14.96) | 0.7688 | 68.44 (66.56 – 70.78; 5.58) | 0.7 | 83.2 (83.2 – 83.2; 0) | 1 |
| **% time COx-a_R > 0** | 67.1 (67.1 – 67.1; 0) |  | 66.6 (59.8 – 72.89; 10.09) |  | 66.88 (54.66 – 74.9; 14.51) |  | 66.74 (57.17 – 76.07; 14.57) |  | 70.93 (65.5 – 71.79; 2.92) |  | 69.01 (66.73 – 77.08; 8.62) |  | 63.82 (54.92 – 75.57; 16.17) |  | 66.88 (63.36 – 69.34; 7.29) |  | 74.9 (74.9 – 74.9; 0) |  |
| **% time COx-a_L > 0.2** | 42.39 (42.39 – 42.39; 0) | 1 | 46.62 (40.04 – 52.4; 9.76) | 0.3057 | 55.03 (43.33 – 56.85; 11.4) | 0.6665 | 45.21 (38.47 – 55.92; 12.77) | 0.2887 | 52.4 (49.73 – 55.61; 4.76) | 0.8413 | 46.78 (43.45 – 51.03; 7.42) | 0.1797 | 48.93 (36.48 – 56.07; 14.68) | 0.91 | 45.82 (45.21 – 51.33; 1.82) | 1 | 60.55 (60.55 – 60.55; 0) | 1 |
| **% time COx-a_R > 0.2** | 56.56 (56.56 – 56.56; 0) |  | 51.16 (42.48 – 59.16; 12.88) |  | 48.15 (37.53 – 58.37; 15.75) |  | 49.8 (42.5 – 58.96; 13.29) |  | 52.16 (42.48 – 55.95; 12.12) |  | 54.36 (49.37 – 58.51; 7.95) |  | 43.91 (38.08 – 60.19; 20.16) |  | 48.15 (46.73 – 49.66; 4.23) |  | 58.37 (58.37 – 58.37; 0) |  |
| **% time COx-a_L > 0.3** | 32.79 (32.79 – 32.79; 0) | 1 | 35.66 (30.72 – 44.26; 10.2) | 0.1497 | 44.48 (38.07 – 50.11; 9.5) | 0.6665 | 35.39 (30.38 – 44.42; 10.58) | 0.1867 | 44.32 (39.82 – 49.56; 6.87) | 1 | 35.82 (33.26 – 39.11; 5.21) | 0.026 | 41.11 (28.77 – 50.06; 16.69) | 0.9459 | 35.66 (35.39 – 42.88; 0.8) | 0.7 | 44.67 (44.67 – 44.67; 0) | 1 |
| **% time COx-a_R > 0.3** | 50.12 (50.12 – 50.12; 0) |  | 43.04 (34.7 – 47.42; 12.29) |  | 40.37 (27.95 – 45.76; 17.92) |  | 41.1 (34.71 – 45.69; 9.45) |  | 47.42 (34.16 – 49.73; 9.16) |  | 44.97 (43.51 – 46.82; 3.25) |  |  |  | 39.84 (38.95 – 40.11; 0.79) |  |  |  |
| **1-Minute Data Resolution** | | | | | | | | | | | | | | | | | | |
| **ABP (mmHg)** | 81 (76 – 88.68) | – | 84.79 (77.25 – 89.5) | – | 83.76 (76.55 – 87.29) | – | 84.29 (77.86 – 89.57) | – | 78.25 (72.94 – 85.81) | – | 83 (77.25 – 90.82) | – | 83.45 (75.88 – 89.21) | – | 86.5 (81.59 – 92.81) | – | 83.79 (78.47 – 87.24) | – |
| **rSO2_L (%)** | 90.07 (77.02 – 93.16) | 1 | 66.33 (62.99 – 69) | 0.7305 | 62.84 (61.75 – 68.49) | 0.6048 | 66.55 (63.62 – 69.08) | 0.9345 | 61.04 (60.99 – 61.75) | 1 | 66.66 (61.99 – 68.67) | 0.9372 | 64.57 (62.37 – 70.92) | 0.91 | 75.9 (74 – 78) | 0.7 | 66.77 (64.48 – 68.49) | 1 |
| **rSO2_R (%)** | 83.77 (68.48 – 87.77) |  | 65.18 (61.99 – 66.3) |  | 69.01 (67.66 – 71.55) |  | 68.19 (67.14 – 70.16) |  | 56.79 (56 – 58.84) |  | 70.65 (62.24 – 73.13) |  | 63.97 (62.49 – 65.4) |  | 73.88 (72.73 – 76.03) |  | 71.61 (67.97 – 74) |  |
| **COx-a_L (au)** | 0.19 (-0.17 – 0.47) | 1 | 0.17 (-0.11 – 0.44) | 0.4134 | 0.25 (-0.02 – 0.52) | 0.4363 | 0.16 (-0.12 – 0.44) | 0.5073 | 0.27 (-0.11 – 0.54) | 0.6905 | 0.18 (-0.11 – 0.44) | 0.3095 | 0.19 (-0.17 – 0.52) | 0.91 | 0.15 (0 – 0.42) | 0.7 | 0.26 (0.09 – 0.47) | 1 |
| **COx-a_R (au)** | 0.29 (-0.16 – 0.61) |  | 0.2 (-0.06 – 0.46) |  | 0.19 (-0.09 – 0.5) |  | 0.2 (-0.09 – 0.46) |  | 0.25 (-0.02 – 0.56) |  | 0.25 (-0.05 – 0.51) |  | 0.13 (-0.12 – 0.42) |  | 0.19 (-0.06 – 0.46) |  | 0.27 (0.01 – 0.5) |  |
| **MAD of ABP (mmHg)** | 9.73 (9.73 – 9.73) | – | 8.31 (5.64 – 10.09) | – | 7.93 (6.2 – 10.25) | – | 8.09 (5.66 – 10.12) | – | 9.44 (8.31 – 10.09) | – | 9.73 (8.63 – 10.6) | – | 8.92 (5.4 – 10.21) | – | 7.93 (6.83 – 10.85) | – | 6.58 (6.58 – 6.58) | – |
| **MAD of rSO2_L (%)** | 7.29 (7.29 – 7.29) | 1 | 2.88 (2.33 – 3.78) | 0.4536 | 3.12 (2.62 – 5.28) | 0.6048 | 3.13 (2.61 – 4.58) | 0.3953 | 2.4 (1.62 – 3.96) | 1 | 2.91 (2.62 – 3.35) | 0.5887 | 3.64 (2.46 – 4.58) | 0.2456 | 2.88 (2.2 – 5.14) | 1 | 2.94 (2.94 – 2.94) | 1 |
| **MAD of rSO2_R (%)** | 7.74 (7.74 – 7.74) |  | 2.56 (1.73 – 3.37) |  | 2.9 (1.93 – 4.52) |  | 2.82 (1.78 – 3.71) |  | 2.96 (1.86 – 3.54) |  | 2.51 (2.38 – 3.17) |  | 2.96 (2.13 – 3.59) |  | 1.73 (1.61 – 4.67) |  | 4.52 (4.52 – 4.52) |  |
| **MAD of COx-a_L (au)** | 0.47 (0.47 – 0.47) | 1 | 0.44 (0.31 – 0.54) | 0.8119 | 0.44 (0.28 – 0.5) | 0.9314 | 0.44 (0.28 – 0.52) | 0.9167 | 0.48 (0.45 – 0.5) | 1 | 0.37 (0.29 – 0.46) | 0.9372 | 0.49 (0.37 – 0.54) | 0.9459 | 0.39 (0.34 – 0.43) | 0.7 | 0.28 (0.28 – 0.28) | 1 |
| **MAD of COx-a_R (au)** | 0.53 (0.53 – 0.53) |  | 0.45 (0.39 – 0.5) |  | 0.35 (0.29 – 0.54) |  | 0.42 (0.32 – 0.53) |  | 0.48 (0.45 – 0.5) |  | 0.42 (0.32 – 0.51) |  | 0.45 (0.34 – 0.58) |  | 0.44 (0.39 – 0.47) |  | 0.35 (0.35 – 0.35) |  |
| **% time rSO2_L > 60%** | 100 (100 – 100; 0) | 1 | 96.17 (65.59 – 100; 5.68) | 0.972 | 99.24 (27.97 – 100; 1.13) | 0.6166 | 97.85 (66.33 – 100; 3.18) | 0.8106 | 13.59 (100 – 7.87; 2396.28) | 0.6558 | 98.09 (75.45 – 100; 2.84) | 0.5058 | 90.1 (17.48 – 99.87; 14.67) | 0.6403 | 99.24 (98.43 – 99.47; 0.7) | 1 | 100 (100 – 100; 0) | – |
| **% time rSO2_R > 60%** | 97.92 (97.92 – 97.92; 0) |  | 91.39 (56 – 100; 12.76) |  | 99.49 (42.05 – 100; 0.76) |  | 98.07 (66.05 – 100; 2.87) |  | 15.12 (100 – 22.9; 2365.16) |  | 82.67 (66.05 – 99.48; 25.51) |  | 94.8 (45.53 – 100; 7.71) |  | 98.71 (98.15 – 99.36; 1.68) |  | 100 (100 – 100; 0) |  |
| **% time rSO2_L > 70%** | 97.22 (97.22 – 97.22; 0) | 1 | 16.55 (0 – 83.19; 24.54) | 0.7791 | 11.02 (4.62 – 37.39; 16.33) | 0.5357 | 19.52 (3.1 – 94.35; 28.94) | 0.7682 | 0 (37.39 – 0; 1352.56) | 1 | 16.3 (3.33 – 78.54; 23.37) | 0.8717 | 24.94 (1.15 – 67.18; 36.98) | 0.5769 | 85.75 (44.17 – 91.69; 17.61) | 0.7 | 9.7 (9.7 – 9.7; 0) | 1 |
| **% time rSO2_R > 70%** | 72.22 (72.22 – 72.22; 0) |  | 7.07 (0 – 95.15; 10.47) |  | 32.14 (12.59 – 51.93; 29.33) |  | 22.91 (2.2 – 68.43; 33.96) |  | 0 (59.82 – 10.47; 1743.74) |  | 37.52 (0.7 – 89.41; 55.62) |  | 11.26 (0.5 – 41.73; 16.69) |  | 97.09 (74.51 – 98.12; 3.04) |  | 57.06 (57.06 – 57.06; 0) |  |
| **% time rSO2_L > 80%** | 70.83 (70.83 – 70.83; 0) | 1 | 0 (0 – 4.86; 0) | 0.8245 | 0 (0 – 12.02; 0) | 0.6279 | 0 (0 – 11.09; 0) | 0.9795 | 0 (0 – 0; 18.95) | 0.4237 | 2.15 (0 – 23.25; 3.19) | 1 | 0 (0 – 7.3; 0) | 0.9311 | 8.29 (4.15 – 28.07; 12.3) | 1 | 0 (0 – 0; 0) | 1 |
| **% time rSO2_R > 80%** | 62.5 (62.5 – 62.5; 0) |  | 0 (0 – 0.69; 0) |  | 0.61 (0 – 1.6; 0.91) |  | 0.14 (0 – 1.51; 0.21) |  | 0 (0 – 0; 91.84) |  | 8.49 (0 – 21.36; 12.59) |  | 0 (0 – 0.77; 0) |  | 1.21 (0.75 – 20.4; 1.37) |  | 0.61 (0.61 – 0.61; 0) |  |
| **% time rSO2_L > 90%** | 50 (50 – 50; 0) | 1 | 0 (0 – 0; 0) | 0.3559 | 0 (0 – 0; 0) | 0.5867 | 0 (0 – 0; 0) | 0.5299 | 0 (0 – 0; 0) | 1 | 0 (0 – 0; 0) | 0.446 | 0 (0 – 0; 0) | 0.9699 | 0 (0 – 0; 0) | 0.505 | 0 (0 – 0; 0) | – |
| **% time rSO2_R > 90%** | 7.64 (7.64 – 7.64; 0) |  | 0 (0 – 0; 0) |  | 0 (0 – 0; 0) |  | 0 (0 – 0; 0) |  | 0 (0 – 0; 0.92) |  | 1.26 (0 – 6.36; 1.86) |  | 0 (0 – 0; 0) |  | 0 (0 – 3.47; 0) |  | 0 (0 – 0; 0) |  |
| **% time COx-a_L > 0** | 68.55 (68.55 – 68.55; 0) | 1 | 66.36 (54.19 – 72.17; 11.52) | 0.3223 | 73.58 (62.2 – 78.92; 16.56) | 0.2973 | 68.24 (54.84 – 75.65; 15.18) | 0.7893 | 65.91 (72.17 – 3.67; 29.06) | 0.8413 | 69.06 (62.4 – 75.12; 12.33) | 0.6991 | 65.13 (54.84 – 73.23; 14.51) | 0.9459 | 74.13 (70.04 – 76.52; 7.09) | 0.4 | 84.76 (84.76 – 84.76; 0) | 1 |
| **% time COx-a_R > 0** | 66.9 (66.9 – 66.9; 0) |  | 71.55 (60.64 – 75.28; 11.56) |  | 69.77 (56.07 – 76.54; 11.62) |  | 69.08 (57.22 – 77.03; 12.76) |  | 66.18 (72.73 – 4.79; 144.95) |  | 70.22 (67.27 – 77.52; 8.64) |  | 64.05 (53.28 – 76.08; 18.3) |  | 70.7 (65.67 – 71.78; 3.22) |  | 76.54 (76.54 – 76.54; 0) |  |
| **% time COx-a_L > 0.2** | 47.58 (47.58 – 47.58; 0) | 1 | 46.62 (38.8 – 55.45; 11.69) | 0.2897 | 53.8 (43.35 – 60.38; 15.49) | 0.4013 | 46.59 (38.75 – 56.79; 11.78) | 0.4113 | 51.7 (57.24 – 3.1; 13.63) | 0.8413 | 47.07 (45.88 – 54.82; 7.18) | 0.3095 | 49.69 (37.86 – 57.02; 16.05) | 1 | 46.62 (46.02 – 56.22; 1.77) | 1 | 59.76 (59.76 – 59.76; 0) | 1 |
| **% time COx-a_R > 0.2** | 55.63 (55.63 – 55.63; 0) |  | 50.16 (41.67 – 60.33; 13.87) |  | 49.19 (37.56 – 58.02; 14.99) |  | 49.68 (41.17 – 60.07; 14.31) |  | 41.67 (58.99 – 11.18; 131.38) |  | 53.59 (49.77 – 59.15; 8.27) |  | 44.96 (38.17 – 59.25; 19.52) |  | 49.19 (47.37 – 49.68; 1.43) |  | 58.02 (58.02 – 58.02; 0) |  |
| **% time COx-a_L > 0.3** | 33.87 (33.87 – 33.87; 0) | 1 | 35.51 (30.98 – 44.16; 11.92) | 0.1932 | 42.07 (35.84 – 56.6; 18.73) | 0.4894 | 35.42 (30.37 – 43.97; 10.83) | 0.2578 | 44.08 (50 – 6.74; 25.94) | – | 34.46 (32.65 – 41.82; 4.23) | 0.0411 | 40.68 (27.96 – 50.35; 18.09) | 1 | 35.51 (35.42 – 47.38; 0.26) | 0.7 | 42.07 (42.07 – 42.07; 0) | 1 |
| **% time COx-a_R > 0.3** | 49.3 (49.3 – 49.3; 0) |  | 42.54 (33.91 – 48.88; 12.41) |  | 42.06 (24.14 – 45.68; 14.81) |  | 42.3 (34.41 – 48.39; 11.41) |  | 33.33 (48.88 – 9.96; 158.11) |  | 44.69 (43.05 – 47.07; 3.92) |  |  |  | 41.01 (39.56 – 41.88; 2.57) |  |  |  |
| **5-Minute Data Resolution** | | | | | | | | | | | | | | | | | | |
| **ABP (mmHg)** | 80.63 (77.12 – 87.95) | – | 84.91 (78.39 – 89.42) | – | 83.1 (77.26 – 87.11) | – | 84.96 (78.4 – 89.32) | – | 76.73 (73.97 – 86.81) | – | 82.64 (77.75 – 90.12) | – | 84.01 (76.78 – 88.8) | – | 87.07 (81.89 – 91.92) | – | 83.74 (78.05 – 86.24) | – |
| **rSO2_L (%)** | 90.62 (76.79 – 93.41) | 1 | 66.3 (63.16 – 68.57) | 0.7596 | 63.05 (61.88 – 68.62) | 0.6048 | 66.57 (63.73 – 68.92) | 1 | 61.13 (60.99 – 61.57) | 1 | 66.64 (62.08 – 68.43) | 0.9372 | 64.55 (62.37 – 70.41) | 0.982 | 75.78 (73.99 – 77.86) | 0.7 | 66.84 (64.68 – 68.62) | 1 |
| **rSO2_R (%)** | 85 (68.68 – 87.23) |  | 65.32 (62.03 – 66.42) |  | 69.06 (67.89 – 71.47) |  | 68.2 (67.3 – 70.24) |  | 56.84 (56.13 – 58.61) |  | 70.54 (62.4 – 72.96) |  | 63.97 (62.51 – 65.11) |  | 73.87 (72.83 – 76.07) |  | 71.61 (68.14 – 74.37) |  |
| **COx-a_L (au)** | 0.14 (-0.09 – 0.38) | 1 | 0.17 (-0.07 – 0.34) | 0.3753 | 0.25 (0.03 – 0.41) | 0.3865 | 0.15 (-0.07 – 0.35) | 0.6172 | 0.26 (0.03 – 0.46) | 1 | 0.15 (-0.1 – 0.38) | 0.1797 | 0.16 (-0.07 – 0.39) | 0.7345 | 0.18 (0.01 – 0.33) | 1 | 0.25 (0.17 – 0.45) | 1 |
| **COx-a_R (au)** | 0.23 (-0.08 – 0.58) |  | 0.2 (-0.02 – 0.39) |  | 0.22 (-0.02 – 0.45) |  | 0.21 (-0.02 – 0.39) |  | 0.27 (-0.02 – 0.49) |  | 0.24 (0.01 – 0.42) |  | 0.13 (-0.04 – 0.41) |  | 0.2 (-0.02 – 0.38) |  | 0.31 (0.02 – 0.46) |  |
| **MAD of ABP (mmHg)** | 9.29 (9.29 – 9.29) | – | 7.11 (5.47 – 9.52) | – | 7.66 (6.94 – 9.54) | – | 7.56 (5.49 – 9.53) | – | 7.35 (5.65 – 8.84) | – | 9.4 (8.43 – 10.56) | – | 7.29 (4.84 – 9.45) | – | 7.66 (6.42 – 10.92) | – | 6.94 (6.94 – 6.94) | – |
| **MAD of rSO2_L (%)** | 5.79 (5.79 – 5.79) | 1 | 2.67 (2.12 – 3.29) | 0.6584 | 2.94 (2.66 – 6.5) | 0.7304 | 2.87 (2.22 – 4.64) | 0.5376 | 2.59 (1.63 – 3.74) | 1 | 2.8 (2.2 – 3.27) | 0.9372 | 2.87 (2.61 – 4.64) | 0.4824 | 2.72 (2.15 – 5.04) | 1 | 2.94 (2.94 – 2.94) | 1 |
| **MAD of rSO2_R (%)** | 7.21 (7.21 – 7.21) |  | 2.61 (1.68 – 3.05) |  | 2.96 (1.85 – 4.49) |  | 2.73 (1.87 – 3.53) |  | 2.75 (1.57 – 3.62) |  | 2.46 (2.23 – 2.78) |  | 2.93 (2.04 – 3.53) |  | 1.9 (1.71 – 5.19) |  | 4.49 (4.49 – 4.49) |  |
| **MAD of COx-a_L (au)** | 0.37 (0.37 – 0.37) | 1 | 0.35 (0.27 – 0.41) | 1 | 0.25 (0.22 – 0.42) | 0.7962 | 0.34 (0.24 – 0.42) | 0.7361 | 0.35 (0.22 – 0.36) | 0.6905 | 0.31 (0.23 – 0.37) | 1 | 0.35 (0.24 – 0.48) | 0.6027 | 0.27 (0.25 – 0.35) | 1 | 0.21 (0.21 – 0.21) | 1 |
| **MAD of COx-a_R (au)** | 0.52 (0.52 – 0.52) |  | 0.34 (0.29 – 0.39) |  | 0.32 (0.22 – 0.37) |  | 0.32 (0.23 – 0.38) |  | 0.35 (0.35 – 0.39) |  | 0.31 (0.24 – 0.34) |  | 0.34 (0.24 – 0.41) |  | 0.37 (0.31 – 0.38) |  | 0.32 (0.32 – 0.32) |  |
| **% time rSO2_L > 60%** | 100 (100 – 100; 0) | – | 95.65 (65.79 – 100; 6.45) | 0.8723 | 100 (29.17 – 100; 0) | 0.8462 | 98.26 (66.7 – 100; 2.59) | 0.8902 | 95.65 (11.11 – 100; 6.45) | 1 | 97.56 (75.86 – 100; 3.62) | 0.6685 | 91.77 (17.64 – 100; 12.21) | 0.6984 | 100 (98.26 – 100; 0) | 0.6428 | 100 (100 – 100; 0) | – |
| **% time rSO2_R > 60%** | 100 (100 – 100; 0) |  | 90.91 (63.64 – 100; 13.48) |  | 100 (44.44 – 100; 0) |  | 98.73 (66.01 – 100; 1.88) |  | 19.44 (17.14 – 100; 25.3) |  | 83.33 (66.01 – 100; 24.71) |  | 95.45 (49.24 – 100; 6.74) |  | 97.47 (96.95 – 98.73; 1.54) |  | 100 (100 – 100; 0) |  |
| **% time rSO2_L > 70%** | 96.55 (96.55 – 96.55; 0) | 1 | 15.15 (0 – 82.61; 22.46) | 0.6976 | 8.33 (2.56 – 41.67; 12.36) | 0.479 | 17.33 (2.68 – 93.9; 25.7) | 0.8222 | 0 (0 – 41.67; 0) | 0.6558 | 15.31 (4.75 – 77.29; 20.75) | 0.8717 | 21.09 (0.64 – 63.99; 31.27) | 0.6235 | 86.08 (44.45 – 91.29; 15.47) | 0.7 | 5.88 (5.88 – 5.88; 0) | 1 |
| **% time rSO2_R > 70%** | 72.41 (72.41 – 72.41; 0) |  | 5.56 (0 – 95.12; 8.24) |  | 33.33 (13.33 – 53.16; 29.65) |  | 25 (0.69 – 68.7; 37.07) |  | 5.56 (0 – 58.33; 8.24) |  | 37.6 (0.69 – 89.44; 55.74) |  | 12.73 (0 – 46.79; 18.87) |  | 96.43 (74.8 – 98.21; 5.3) |  | 57.58 (57.58 – 57.58; 0) |  |
| **% time rSO2_L > 80%** | 72.41 (72.41 – 72.41; 0) | 1 | 0 (0 – 6.67; 0) | 0.4937 | 0 (0 – 13.51; 0) | 1 | 0 (0 – 12.46; 0) | 0.5221 | 0 (0 – 0; 0) | 1 | 1.22 (0 – 24.8; 1.81) | 1 | 0 (0 – 6.52; 0) | 0.7395 | 9.3 (4.65 – 29.33; 13.79) | 0.8248 | 0 (0 – 0; 0) | – |
| **% time rSO2_R > 80%** | 62.07 (62.07 – 62.07; 0) |  | 0 (0 – 0; 0) |  | 0 (0 – 2.56; 0) |  | 0 (0 – 2.22; 0) |  | 0 (0 – 0; 0) |  | 6.45 (0 – 19.69; 9.57) |  | 0 (0 – 0; 0) |  | 1.19 (0.6 – 20.85; 1.77) |  | 0 (0 – 0; 0) |  |
| **% time rSO2_L > 90%** | 51.72 (51.72 – 51.72; 0) | 1 | 0 (0 – 0; 0) | 0.5995 | 0 (0 – 0; 0) | 0.4968 | 0 (0 – 0; 0) | 0.7401 | 0 (0 – 0; 0) | 0.4237 | 0 (0 – 0; 0) | 0.7526 | 0 (0 – 0; 0) | 1 | 0 (0 – 0; 0) | 0.505 | 0 (0 – 0; 0) | – |
| **% time rSO2_R > 90%** | 6.9 (6.9 – 6.9; 0) |  | 0 (0 – 0; 0) |  | 0 (0 – 0; 0) |  | 0 (0 – 0; 0) |  | 0 (0 – 0; 0) |  | 0 (0 – 5.17; 0) |  | 0 (0 – 0; 0) |  | 0 (0 – 3.16; 0) |  | 0 (0 – 0; 0) |  |
| **% time COx-a_L > 0** | 69.23 (69.23 – 69.23; 0) | 1 | 68.24 (60.53 – 73.08; 11.43) | 0.4588 | 78.48 (64.29 – 83.33; 11.31) | 0.3401 | 68.33 (60.13 – 77.86; 13.11) | 0.5812 | 79.17 (69.44 – 81.25; 3.93) | 0.5476 | 68.83 (65.02 – 78.25; 12.87) | 0.3939 | 67.33 (59.23 – 78.38; 15.2) | 0.7828 | 78.48 (73.36 – 80.78; 6.81) | 0.4 | 88.24 (88.24 – 88.24; 0) | 1 |
| **% time COx-a_R > 0** | 68.97 (68.97 – 68.97; 0) |  | 72 (63.86 – 79.31; 12.08) |  | 73.42 (58.33 – 77.14; 16.95) |  | 72.44 (59.71 – 79.1; 15.09) |  | 72 (65.12 – 78.79; 10.21) |  | 74.12 (69.09 – 82.85; 9.34) |  | 68.46 (48.71 – 76.61; 21.73) |  | 73.42 (68.64 – 75.94; 7.48) |  | 84.85 (84.85 – 84.85; 0) |  |
| **% time COx-a_L > 0.2** | 38.46 (38.46 – 38.46; 0) | 1 | 45.45 (36.11 – 56.25; 16.01) | 0.3262 | 60 (37.14 – 67.65; 14.26) | 0.2002 | 43.41 (34.03 – 60.83; 22.72) | 0.4596 | 56.25 (52.78 – 60; 5.56) | 0.9166 | 40.28 (36.7 – 52.71; 14.93) | 0.2403 | 41.48 (29.76 – 60.83; 27.46) | 0.8903 | 46.15 (45.43 – 57.89; 2.15) | 1 | 67.65 (67.65 – 67.65; 0) | 1 |
| **% time COx-a_R > 0.2** | 55.17 (55.17 – 55.17; 0) |  | 50 (39.47 – 63.89; 17.89) |  | 54.43 (26.09 – 57.58; 12.49) |  | 52.22 (38.17 – 61.73; 19.21) |  | 54.55 (41.86 – 60; 13.85) |  | 54.86 (51.14 – 61.16; 9.75) |  | 39.9 (27.07 – 59.58; 25.28) |  | 47.69 (43.73 – 51.06; 9.99) |  | 57.58 (57.58 – 57.58; 0) |  |
| **% time COx-a_L > 0.3** | 26.92 (26.92 – 26.92; 0) | 1 | 30.77 (24.71 – 37.5; 9.98) | 0.0705 | 41.18 (29.17 – 52; 17.81) | 0.4363 | 29.49 (25.11 – 40.05; 11.57) | 0.1886 | 41.67 (37.5 – 44; 6.18) | 0.8413 | 27.35 (26.47 – 34.44; 7.67) | **0.0303** | 32.38 (27.74 – 49.57; 24.84) | 0.9817 | 30.77 (27.74 – 44.5; 8.99) | 0.7 | 41.18 (41.18 – 41.18; 0) | 1 |
| **% time COx-a_R > 0.3** | 48.28 (48.28 – 48.28; 0) |  | 36.11 (32.31 – 47.37; 15.65) |  | 36.11 (13.04 – 47.22; 26.95) |  | 36.11 (31.57 – 48.05; 16.58) |  | 45.45 (32.56 – 47.22; 15.63) |  | 46.06 (38.45 – 47.19; 2.61) |  |  |  | 32.31 (31.82 – 36.41; 1.46) |  |  |  |
| *The p-values in the table are derived using Mann-Whitney U test between the bilateral signals.*  *ABP, arterial blood pressure; au, arbitrary units; ACDF, anterior cervical discectomy and fusion; CPP, cerebral perfusion pressure; COx, cerebral oximetry index with CPP; COx-a, cerebral oximetry index with ABP; IQR, interquartile range; mmHg, millimeters of mercury; MAD, median absolute deviation; PCDF, posterior cervical discectomy and fusion; rSO2, regional cerebral oxygen saturation; SP, elective spinal surgery patient group.* | | | | | | | | | | | | | | | | | | |

| **Physiologic Variable** | **Sub-groups** | | | | | | | | | | | | | | | | | | | |
| --- | --- | --- | --- | --- | --- | --- | --- | --- | --- | --- | --- | --- | --- | --- | --- | --- | --- | --- | --- | --- |
|  | **Corpectomy [n = 1]** | | **Laminectomy [n = 1]** | | **Thoracic Decompression & Instrumental Fusion [n = 1]** | | **Propofol + Sufentanil [n = 6]** | | **Ketamine + Propofol + Sufentanil [n = 6]** | | **Midazolam + Propofol + Remi-Fentanyl [n = 1]** | | **Midazolam + Propofol + Sufentanil [n = 4]** | | **Propofol + Remi-Fentanyl + Sufentanil [n = 2]** | | **Ketamine + Midazolam + Propofol + Sufentanil [n = 7]** | | **Ketamine + Midazolam + Propofol + Remi-Fentanyl + Sufentanil [n = 1]** | |
|  | **Median (IQR) or  Median (IQR; MAD)** | **p-value** | **Median (IQR) or  Median (IQR; MAD)** | **p-value** | **Median (IQR) or  Median (IQR; MAD)** | **p-value** | **Median (IQR) or  Median (IQR; MAD)** | **p-value** | **Median (IQR) or  Median (IQR; MAD)** | **p-value** | **Median (IQR) or  Median (IQR; MAD)** | **p-value** | **Median (IQR) or  Median (IQR; MAD)** | **p-value** | **Median (IQR) or  Median (IQR; MAD)** | **p-value** | **Median (IQR) or  Median (IQR; MAD)** | **p-value** | **Median (IQR) or  Median (IQR; MAD)** | **p-value** |
| **10-Second Data Resolution** | | | | | | | | | | | | | | | | | | | | |
| **ABP (mmHg)** | 77.69 (72.85 – 85.27) | – | 82.22 (77.01 – 86.59) | – | 77.37 (73.7 – 81.64) | – | 83.85 (76.89 – 90.14) | – | 83.52 (75.23 – 90.53) | – | 92.59 (86.14 – 101.2) | – | 83.74 (77.93 – 90) | – | 80.46 (73.56 – 87.23) | – | 85.05 (81.72 – 88.93) | – | 75.81 (72.27 – 82.48) | – |
| **rSO2_L (%)** | 48.99 (47.99 – 50.99) | 1 | 71.99 (71.68 – 74.29) | 1 | 31.49 (27.49 – 35.97) | 1 | 63.63 (57.49 – 64.99) | 1 | 69.49 (67.89 – 75.5) | 1 | 66.99 (58.99 – 68.08) | 1 | 60.89 (59.5 – 64.03) | 0.8857 | 70.3 (67.7 – 72.5) | 0.6667 | 70.49 (68.48 – 74.16) | 0.8982 | 66.99 (63.99 – 71.99) | 1 |
| **rSO2_R (%)** | 56 (54.99 – 58.99) |  | 69.99 (69 – 73.65) |  | 35.06 (30.7 – 38.9) |  | 61.02 (55.02 – 64.78) |  | 69.29 (67.83 – 72.39) |  | 65.79 (54.99 – 66.99) |  | 66.39 (63.99 – 71.78) |  | 67.82 (64.99 – 69.49) |  | 63.99 (62.99 – 65) |  | 74 (71.99 – 78.99) |  |
| **COx-a_L (au)** | 0.2 (-0.11 – 0.71) | 1 | 0.11 (0 – 0.43) | 1 | 0.06 (-0.24 – 0.35) | 1 | 0.23 (-0.01 – 0.45) | 0.8182 | 0.08 (-0.21 – 0.42) | 0.6991 | 0.16 (-0.18 – 0.43) | 1 | 0.11 (-0.26 – 0.56) | 0.8857 | 0.33 (-0.11 – 0.65) | 0.6667 | 0.17 (-0.02 – 0.43) | 0.3829 | 0.17 (-0.18 – 0.55) | 1 |
| **COx-a_R (au)** | 0.3 (0 – 0.69) |  | 0.23 (0 – 0.48) |  | 0.06 (-0.35 – 0.4) |  | 0.25 (-0.01 – 0.5) |  | 0.12 (-0.18 – 0.38) |  | 0.14 (-0.09 – 0.47) |  | 0.18 (-0.15 – 0.57) |  | 0.41 (-0.01 – 0.67) |  | 0.22 (-0.03 – 0.48) |  | -0.04 (-0.41 – 0.35) |  |
| **MAD of ABP (mmHg)** | 8.61 (8.61 – 8.61) | – | 6.89 (6.89 – 6.89) | – | 5.85 (5.85 – 5.85) | – | 8.08 (6.28 – 10.12) | – | 9.59 (7.11 – 12.72) | – | 10.81 (10.81 – 10.81) | – | 8.34 (7.34 – 8.94) | – | 10.12 (9.87 – 10.37) | – | 6.89 (5.72 – 9.87) | – | 6.32 (6.32 – 6.32) | – |
| **MAD of rSO2_L (%)** | 1.48 (1.48 – 1.48) | 1 | 1.48 (1.48 – 1.48) | 1 | 6.18 (6.18 – 6.18) | 1 | 3.1 (2.98 – 5.43) | 0.8182 | 2.91 (2.83 – 3.7) | 0.2403 | 3.53 (3.53 – 3.53) | 1 | 2.56 (2.24 – 3.82) | 0.5614 | 3.42 (2.91 – 3.94) | 1 | 4 (1.49 – 4.23) | 0.4817 | 5.65 (5.65 – 5.65) | 1 |
| **MAD of rSO2_R (%)** | 1.6 (1.6 – 1.6) |  | 1.47 (1.47 – 1.47) |  | 6.03 (6.03 – 6.03) |  | 3.69 (2.75 – 5.66) |  | 2.34 (1.63 – 2.87) |  | 2.67 (2.67 – 2.67) |  | 3.45 (2.85 – 4.58) |  | 3.22 (3.09 – 3.35) |  | 1.5 (1.48 – 3.49) |  | 3.87 (3.87 – 3.87) |  |
| **MAD of COx-a_L (au)** | 0.58 (0.58 – 0.58) | 1 | 0.34 (0.34 – 0.34) | 1 | 0.44 (0.44 – 0.44) | 1 | 0.31 (0.29 – 0.41) | 0.4848 | 0.43 (0.38 – 0.54) | 0.8182 | 0.46 (0.46 – 0.46) | 1 | 0.56 (0.52 – 0.62) | 0.8857 | 0.52 (0.5 – 0.54) | 0.6667 | 0.34 (0.29 – 0.4) | 0.535 | 0.54 (0.54 – 0.54) | 1 |
| **MAD of COx-a_R (au)** | 0.51 (0.51 – 0.51) |  | 0.35 (0.35 – 0.35) |  | 0.55 (0.55 – 0.55) |  | 0.42 (0.34 – 0.53) |  | 0.41 (0.33 – 0.56) |  | 0.41 (0.41 – 0.41) |  | 0.56 (0.49 – 0.61) |  | 0.48 (0.47 – 0.49) |  | 0.42 (0.35 – 0.43) |  | 0.56 (0.56 – 0.56) |  |
| **% time rSO2_L > 60%** | 13.47 (13.47 – 13.47; 0) | 1 | 99.76 (99.76 – 99.76; 0) | 1 | 0 (0 – 0; 0) | – | 82.7 (37.31 – 99.77; 25.5) | 0.8721 | 98.44 (77.78 – 99.76; 2.32) | 0.6836 | 68.23 (68.23 – 68.23; 0) | 1 | 54.55 (12.54 – 96.65; 63.66) | 0.4857 | 89.94 (87.41 – 92.46; 7.5) | 0.6667 | 99.76 (50.44 – 99.93; 0.35) | 0.5644 | 99.71 (99.71 – 99.71; 0) | 1 |
| **% time rSO2_R > 60%** | 18.54 (18.54 – 18.54; 0) |  | 99.88 (99.88 – 99.88; 0) |  | 0 (0 – 0; 0) |  | 51.63 (20.99 – 91.34; 62.6) |  | 99.7 (98.03 – 99.94; 0.45) |  | 67.39 (67.39 – 67.39; 0) |  | 85.68 (58.9 – 99.23; 20.43) |  | 95.21 (92.81 – 97.6; 7.11) |  | 97.56 (26.92 – 98.98; 3.54) |  | 99.76 (99.76 – 99.76; 0) |  |
| **% time rSO2_L > 70%** | 0 (0 – 0; 0) | 1 | 99.76 (99.76 – 99.76; 0) | 1 | 0 (0 – 0; 0) | – | 4.12 (0.21 – 9.94; 6.05) | 1 | 65.45 (12.56 – 99.38; 51.16) | 1 | 10.34 (10.34 – 10.34; 0) | 1 | 10.79 (0 – 37.71; 15.99) | 0.4678 | 49.58 (32.11 – 67.05; 51.8) | 0.6667 | 54.98 (1.31 – 82.2; 66.39) | 0.6012 | 36.23 (36.23 – 36.23; 0) | 1 |
| **% time rSO2_R > 70%** | 6.6 (6.6 – 6.6; 0) |  | 32.54 (32.54 – 32.54; 0) |  | 0 (0 – 0; 0) |  | 6.95 (0.1 – 44.89; 10.3) |  | 37.6 (17.68 – 83.34; 41.72) |  | 2.73 (2.73 – 2.73; 0) |  | 29.19 (5.1 – 62.64; 37.95) |  | 34.29 (21.71 – 46.87; 37.3) |  | 8.28 (0 – 52.45; 12.27) |  | 91.08 (91.08 – 91.08; 0) |  |
| **% time rSO2_L > 80%** | 0 (0 – 0; 0) | 1 | 4.62 (4.62 – 4.62; 0) | 1 | 0 (0 – 0; 0) | – | 0 (0 – 0; 0) | 0.7526 | 9.79 (1.91 – 15.63; 12.5) | 0.4632 | 0 (0 – 0; 0) | 1 | 2.19 (0 – 15.22; 3.25) | 0.8824 | 4.55 (2.28 – 6.83; 6.75) | 0.6171 | 0 (0 – 2.99; 0) | 1 | 0 (0 – 0; 0) | 1 |
| **% time rSO2_R > 80%** | 0.18 (0.18 – 0.18; 0) |  | 1.19 (1.19 – 1.19; 0) |  | 0 (0 – 0; 0) |  | 0 (0 – 0.48; 0) |  | 1.28 (0.27 – 1.65; 1.27) |  | 0.09 (0.09 – 0.09; 0) |  | 11.22 (0.14 – 26.58; 16.5) |  | 0 (0 – 0; 0) |  | 0.43 (0 – 1.28; 0.64) |  | 20.72 (20.72 – 20.72; 0) |  |
| **% time rSO2_L > 90%** | 0 (0 – 0; 0) | – | 0 (0 – 0; 0) | – | 0 (0 – 0; 0) | – | 0 (0 – 0; 0) | 1 | 0 (0 – 2.55; 0) | 0.5993 | 0 (0 – 0; 0) | – | 0 (0 – 0; 0) | 0.1859 | 0 (0 – 0; 0) | – | 0 (0 – 0; 0) | 1 | 0 (0 – 0; 0) | 1 |
| **% time rSO2_R > 90%** | 0 (0 – 0; 0) |  | 0 (0 – 0; 0) |  | 0 (0 – 0; 0) |  | 0 (0 – 0; 0) |  | 0 (0 – 0; 0) |  | 0 (0 – 0; 0) |  | 2.93 (0 – 6.1; 4.35) |  | 0 (0 – 0; 0) |  | 0 (0 – 0; 0) |  | 1.93 (1.93 – 1.93; 0) |  |
| **% time COx-a_L > 0** | 63.46 (63.46 – 63.46; 0) | 1 | 55.48 (55.48 – 55.48; 0) | 1 | 55.57 (55.57 – 55.57; 0) | 1 | 68.68 (66.4 – 79.92; 11.63) | 1 | 58.42 (53.76 – 63.84; 8.53) | 0.9372 | 60.42 (60.42 – 60.42; 0) | 1 | 57.03 (50.55 – 64.71; 9.68) | 0.4857 | 70.66 (68.36 – 72.96; 6.83) | 0.6667 | 64.39 (56.61 – 73.24; 13.21) | 0.62 | 61.88 (61.88 – 61.88; 0) | 1 |
| **% time COx-a_R > 0** | 71.79 (71.79 – 71.79; 0) |  | 63.18 (63.18 – 63.18; 0) |  | 53.78 (53.78 – 53.78; 0) |  | 72.91 (58.72 – 78.07; 16.42) |  | 60.98 (53.31 – 67.72; 13.67) |  | 66.6 (66.6 – 66.6; 0) |  | 63.34 (58.92 – 68.11; 7.85) |  | 74.68 (73.79 – 75.57; 2.65) |  | 67.1 (64.34 – 74.84; 6.97) |  | 46.25 (46.25 – 46.25; 0) |  |
| **% time COx-a_L > 0.2** | 49.73 (49.73 – 49.73; 0) | 1 | 42.58 (42.58 – 42.58; 0) | 1 | 37.2 (37.2 – 37.2; 0) | 1 | 54.31 (48.31 – 58.05; 7.85) | 0.9372 | 40.64 (32.78 – 44.28; 10.03) | 0.9372 | 46.62 (46.62 – 46.62; 0) | 1 | 44.88 (39.02 – 51.51; 10.19) | 0.8857 | 58.16 (56.88 – 59.44; 3.79) | 0.6667 | 45.82 (42.48 – 52.78; 6.98) | 0.4557 | 47.34 (47.34 – 47.34; 0) | 1 |
| **% time COx-a_R > 0.2** | 55.95 (55.95 – 55.95; 0) |  | 51.81 (51.81 – 51.81; 0) |  | 39.78 (39.78 – 39.78; 0) |  | 55.27 (47.05 – 58.96; 9.89) |  | 41.42 (25.32 – 56.15; 28.55) |  | 43.53 (43.53 – 43.53; 0) |  | 48.3 (46.66 – 50.32; 4.66) |  | 62.36 (61.35 – 63.37; 2.99) |  | 51.81 (46.82 – 55.08; 7.04) |  | 32.56 (32.56 – 32.56; 0) |  |
| **% time COx-a_L > 0.3** | 44.32 (44.32 – 44.32; 0) | 1 | 33.94 (33.94 – 33.94; 0) | 1 | 29.69 (29.69 – 29.69; 0) | 1 | 42.04 (33 – 44.57; 9.9) | 0.3095 | 32.69 (18.91 – 37.33; 17.01) | 0.9372 | 36.97 (36.97 – 36.97; 0) | 1 | 39.5 (33.07 – 45.77; 11.45) | 1 | 51.5 (50.87 – 52.14; 1.89) | 0.3333 | 35.66 (33.36 – 43.51; 10.2) | 0.2593 | 39.69 (39.69 – 39.69; 0) | 1 |
| **% time COx-a_R > 0.3** | 49.73 (49.73 – 49.73; 0) |  | 45.48 (45.48 – 45.48; 0) |  | 31.65 (31.65 – 31.65; 0) |  | 44.97 (42.6 – 45.58; 2.4) |  | 30.86 (19.63 – 48.86; 24.46) |  | 34.75 (34.75 – 34.75; 0) |  | 41.71 (38.92 – 44.71; 6.29) |  | 56.13 (54.86 – 57.39; 3.75) |  | 39.84 (36.56 – 47.8; 8.36) |  | 27.95 (27.95 – 27.95; 0) |  |
| **1-Minute Data Resolution** | | | | | | | | | | | | | | | | | | | | |
| **ABP (mmHg)** | 77.03 (72.94 – 85.81) | – | 82.56 (77.01 – 86.49) | – | 77.34 (73.85 – 81.69) | – | 83.78 (77.51 – 90.1) | – | 83.77 (75.53 – 90.13) | – | 92.34 (86.75 – 101.52) | – | 83.45 (77.98 – 89.82) | – | 80.69 (73.82 – 87.22) | – | 84.79 (81.23 – 89.5) | – | 75.89 (72.39 – 82.61) | – |
| **rSO2_L (%)** | 49.08 (47.99 – 51.06) | 1 | 71.99 (71.7 – 74.39) | 1 | 31.6 (27.69 – 36.02) | 1 | 63.69 (57.41 – 64.79) | 1 | 69.37 (67.88 – 75.47) | 0.9372 | 66.99 (58.98 – 68.18) | 1 | 61.01 (59.5 – 64.14) | 0.8857 | 70.45 (67.6 – 72.48) | 0.6667 | 70.47 (68.13 – 72.97) | 0.8982 | 67.43 (63.99 – 72.84) | 1 |
| **rSO2_R (%)** | 56.09 (54.99 – 58.84) |  | 69.92 (69 – 73.78) |  | 35.21 (30.7 – 38.87) |  | 60.98 (55.1 – 64.85) |  | 69.32 (67.8 – 72.28) |  | 65.77 (55.33 – 66.79) |  | 66.35 (64.05 – 72.07) |  | 67.79 (64.92 – 69.42) |  | 63.95 (62.99 – 65) |  | 74.14 (72.07 – 79.25) |  |
| **COx-a_L (au)** | 0.22 (-0.11 – 0.69) | 1 | 0.21 (-0.11 – 0.47) | 1 | 0.06 (-0.24 – 0.35) | 1 | 0.25 (-0.01 – 0.45) | 0.9372 | 0.1 (-0.19 – 0.41) | 0.6991 | 0.15 (-0.16 – 0.41) | 1 | 0.14 (-0.27 – 0.56) | 0.8857 | 0.34 (-0.09 – 0.65) | 0.6667 | 0.2 (-0.11 – 0.47) | 0.535 | 0.17 (-0.16 – 0.52) | 1 |
| **COx-a_R (au)** | 0.29 (0.03 – 0.68) |  | 0.32 (-0.06 – 0.49) |  | 0.06 (-0.31 – 0.4) |  | 0.25 (0 – 0.48) |  | 0.13 (-0.17 – 0.38) |  | 0.13 (-0.08 – 0.45) |  | 0.19 (-0.17 – 0.57) |  | 0.4 (0.03 – 0.64) |  | 0.23 (-0.06 – 0.43) |  | -0.04 (-0.41 – 0.29) |  |
| **MAD of ABP (mmHg)** | 8.31 (8.31 – 8.31) | – | 6.6 (6.6 – 6.6) | – | 5.64 (5.64 – 5.64) | – | 7.42 (5.87 – 10.23) | – | 8.93 (6.6 – 12.41) | – | 11.33 (11.33 – 11.33) | – | 8.12 (7.25 – 8.66) | – | 9.96 (9.7 – 10.22) | – | 6.6 (5.42 – 9.91) | – | 6.2 (6.2 – 6.2) | – |
| **MAD of rSO2_L (%)** | 1.62 (1.62 – 1.62) | 1 | 1.41 (1.41 – 1.41) | 1 | 6.17 (6.17 – 6.17) | 1 | 3.04 (2.74 – 5.41) | 0.9372 | 3 (2.68 – 3.61) | 0.3095 | 3.42 (3.42 – 3.42) | 1 | 2.47 (2.15 – 3.8) | 0.4857 | 3.52 (2.96 – 4.08) | 1 | 3.5 (1.47 – 4.2) | 0.4557 | 5.28 (5.28 – 5.28) | 1 |
| **MAD of rSO2_R (%)** | 1.86 (1.86 – 1.86) |  | 1.37 (1.37 – 1.37) |  | 6.41 (6.41 – 6.41) |  | 3.49 (2.38 – 5.94) |  | 2.33 (1.78 – 2.86) |  | 2.56 (2.56 – 2.56) |  | 3.33 (2.94 – 4.43) |  | 3.25 (3.11 – 3.4) |  | 1.49 (1.41 – 3.28) |  | 4.01 (4.01 – 4.01) |  |
| **MAD of COx-a_L (au)** | 0.6 (0.6 – 0.6) | 1 | 0.42 (0.42 – 0.42) | 1 | 0.44 (0.44 – 0.44) | 1 | 0.3 (0.28 – 0.41) | 0.4848 | 0.42 (0.36 – 0.52) | 0.8182 | 0.44 (0.44 – 0.44) | 1 | 0.58 (0.54 – 0.62) | 0.8857 | 0.51 (0.5 – 0.52) | 0.3333 | 0.42 (0.27 – 0.46) | 1 | 0.5 (0.5 – 0.5) | 1 |
| **MAD of COx-a_R (au)** | 0.5 (0.5 – 0.5) |  | 0.35 (0.35 – 0.35) |  | 0.52 (0.52 – 0.52) |  | 0.4 (0.31 – 0.51) |  | 0.4 (0.3 – 0.56) |  | 0.39 (0.39 – 0.39) |  | 0.55 (0.48 – 0.61) |  | 0.46 (0.46 – 0.47) |  | 0.39 (0.34 – 0.43) |  | 0.54 (0.54 – 0.54) |  |
| **% time rSO2_L > 60%** | 13.59 (13.59 – 13.59; 0) | 1 | 100 (100 – 100; 0) | – | 0 (0 – 0; 0) | – | 82.8 (37.37 – 100; 25.51) | 0.6769 | 98.56 (78.51 – 99.87; 2.14) | 0.5611 | 68.54 (68.54 – 68.54; 0) | 1 | 55.08 (13.89 – 96.94; 61.22) | 0.4857 | 90.1 (87.81 – 92.4; 6.8) | 0.6667 | 99.71 (51.37 – 100; 0.43) | 0.556 | 100 (100 – 100; 0) | – |
| **% time rSO2_R > 60%** | 17.93 (17.93 – 17.93; 0) |  | 100 (100 – 100; 0) |  | 0 (0 – 0; 0) |  | 51.17 (20.53 – 91.4; 62.92) |  | 99.74 (98.06 – 100; 0.38) |  | 67.42 (67.42 – 67.42; 0) |  | 88.17 (62.7 – 99.04; 16.59) |  | 95.7 (93.54 – 97.85; 6.38) |  | 97.92 (29.24 – 99.11; 3.09) |  | 100 (100 – 100; 0) |  |
| **% time rSO2_L > 70%** | 0 (0 – 0; 0) | 1 | 98.61 (98.61 – 98.61; 0) | 1 | 0 (0 – 0; 0) | – | 5.39 (0.27 – 10.69; 7.99) | 1 | 65.48 (11.79 – 99.01; 50.78) | 1 | 10.11 (10.11 – 10.11; 0) | 1 | 11.24 (0 – 38.3; 16.67) | 0.6573 | 49.87 (33.21 – 66.53; 49.4) | 0.6667 | 55.77 (1.3 – 84.1; 63.52) | 0.6012 | 37.39 (37.39 – 37.39; 0) | 1 |
| **% time rSO2_R > 70%** | 7.07 (7.07 – 7.07; 0) |  | 34.48 (34.48 – 34.48; 0) |  | 0 (0 – 0; 0) |  | 6.84 (0 – 46.21; 10.14) |  | 38.53 (17.48 – 84.05; 42.74) |  | 2.81 (2.81 – 2.81; 0) |  | 29.5 (5.3 – 62.73; 38.49) |  | 34.88 (22.41 – 47.35; 36.98) |  | 2 (0 – 53.35; 2.97) |  | 91.43 (91.43 – 91.43; 0) |  |
| **% time rSO2_L > 80%** | 0 (0 – 0; 0) | – | 4.86 (4.86 – 4.86; 0) | 1 | 0 (0 – 0; 0) | – | 0 (0 – 0; 0) | 0.7526 | 10.16 (2.07 – 17.29; 14.12) | 0.4632 | 0 (0 – 0; 0) | – | 2.15 (0 – 15.19; 3.19) | 1 | 4.87 (2.43 – 7.3; 7.22) | 0.6171 | 0 (0 – 2.43; 0) | 0.8816 | 0 (0 – 0; 0) | 1 |
| **% time rSO2_R > 80%** | 0 (0 – 0; 0) |  | 0.69 (0.69 – 0.69; 0) |  | 0 (0 – 0; 0) |  | 0 (0 – 0.46; 0) |  | 1.12 (0.26 – 1.51; 1.19) |  | 0 (0 – 0; 0) |  | 11.41 (0 – 27.01; 16.91) |  | 0 (0 – 0; 0) |  | 0 (0 – 0.49; 0) |  | 21.43 (21.43 – 21.43; 0) |  |
| **% time rSO2_L > 90%** | 0 (0 – 0; 0) | – | 0 (0 – 0; 0) | – | 0 (0 – 0; 0) | – | 0 (0 – 0; 0) | 0.4047 | 0 (0 – 2.87; 0) | 0.5993 | 0 (0 – 0; 0) | – | 0 (0 – 0; 0) | 0.1859 | 0 (0 – 0; 0) | – | 0 (0 – 0; 0) | 1 | 0 (0 – 0; 0) | 1 |
| **% time rSO2_R > 90%** | 0 (0 – 0; 0) |  | 0 (0 – 0; 0) |  | 0 (0 – 0; 0) |  | 0 (0 – 0; 0) |  | 0 (0 – 0; 0) |  | 0 (0 – 0; 0) |  | 3.47 (0 – 7.15; 5.15) |  | 0 (0 – 0; 0) |  | 0 (0 – 0; 0) |  | 2.14 (2.14 – 2.14; 0) |  |
| **% time COx-a_L > 0** | 65.91 (65.91 – 65.91; 0) | 1 | 70.91 (70.91 – 70.91; 0) | 1 | 54.19 (54.19 – 54.19; 0) | 1 | 75.28 (70.57 – 82.81; 11.26) | 0.9372 | 59.27 (54.84 – 65.01; 8.6) | 1 | 60.34 (60.34 – 60.34; 0) | 1 | 58.91 (51.08 – 69.16; 12.84) | 0.6857 | 71.26 (68.81 – 73.71; 7.26) | 0.6667 | 70.91 (68.24 – 73.15; 4.42) | 1 | 63.89 (63.89 – 63.89; 0) | 1 |
| **% time COx-a_R > 0** | 75.28 (75.28 – 75.28; 0) |  | 71.55 (71.55 – 71.55; 0) |  | 55.56 (55.56 – 55.56; 0) |  | 74.3 (60.07 – 78.65; 14.62) |  | 61.29 (51.18 – 67.81; 16.11) |  | 68.39 (68.39 – 68.39; 0) |  | 66.29 (60.32 – 71.84; 9.94) |  | 75.29 (74.01 – 76.58; 3.81) |  | 71.55 (66.54 – 76.13; 7.97) |  | 45.69 (45.69 – 45.69; 0) |  |
| **% time COx-a_L > 0.2** | 51.7 (51.7 – 51.7; 0) | 1 | 50.91 (50.91 – 50.91; 0) | 1 | 38.55 (38.55 – 38.55; 0) | 1 | 57.51 (48.55 – 59.27; 3.78) | 0.8182 | 40.46 (33.01 – 45.8; 11.21) | 1 | 46.55 (46.55 – 46.55; 0) | 1 | 45.25 (38.78 – 55.23; 9.62) | 0.8857 | 58.5 (56.98 – 60.02; 4.51) | 0.6667 | 50.31 (46.5 – 52.36; 5.17) | 0.535 | 49.07 (49.07 – 49.07; 0) | 1 |
| **% time COx-a_R > 0.2** | 58.99 (58.99 – 58.99; 0) |  | 60.34 (60.34 – 60.34; 0) |  | 38.89 (38.89 – 38.89; 0) |  | 54.79 (48.64 – 59.75; 9.39) |  | 41.55 (23.28 – 55.86; 27.87) |  | 40.8 (40.8 – 40.8; 0) |  | 49.18 (47.44 – 51.64; 5.14) |  | 61.99 (60.54 – 63.44; 4.3) |  | 53.22 (45.91 – 57.99; 10.57) |  | 32.76 (32.76 – 32.76; 0) |  |
| **% time COx-a_L > 0.3** | 45.45 (45.45 – 45.45; 0) | 1 | 41.82 (41.82 – 41.82; 0) | 1 | 30.17 (30.17 – 30.17; 0) | 1 | 43.08 (33.75 – 44.14; 9.77) | 0.3939 | 32.47 (17.99 – 35.76; 16.06) | 0.9372 | 35.06 (35.06 – 35.06; 0) | 1 | 38.85 (31.05 – 48.91; 13.34) | 0.8857 | 51.92 (50.96 – 52.88; 2.85) | 0.3333 | 41.82 (34.6 – 44.21; 9.62) | 0.7104 | 37.96 (37.96 – 37.96; 0) | 1 |
| **% time COx-a_R > 0.3** | 48.88 (48.88 – 48.88; 0) |  | 52.59 (52.59 – 52.59; 0) |  | 32.78 (32.78 – 32.78; 0) |  | 44.69 (42.68 – 45.46; 2.69) |  | 30.48 (17.52 – 48.56; 26.52) |  | 33.91 (33.91 – 33.91; 0) |  | 42.64 (40.88 – 44.28; 4.7) |  | 56.66 (55.6 – 57.72; 3.14) |  | 41.01 (34.8 – 50.1; 12.29) |  | 24.14 (24.14 – 24.14; 0) |  |
| **5-Minute Data Resolution** | | | | | | | | | | | | | | | | | | | | |
| **ABP (mmHg)** | 76.73 (72.72 – 84.07) | – | 82.95 (78.74 – 85.95) | – | 77.47 (74.14 – 81.49) | – | 83.42 (77.65 – 88.53) | – | 84.23 (76.66 – 89.79) | – | 92.98 (86.85 – 101.28) | – | 83.86 (78.29 – 89.57) | – | 80.71 (75.09 – 87.5) | – | 85.02 (80.92 – 89.42) | – | 75.64 (73.97 – 81.13) | – |
| **rSO2_L (%)** | 49 (48.05 – 51) | 1 | 71.99 (71.68 – 75.12) | 1 | 31.69 (28.29 – 35.56) | 1 | 63.71 (57.54 – 64.77) | 1 | 69.41 (67.94 – 75.04) | 1 | 66.99 (59.11 – 68.29) | 1 | 60.76 (59.54 – 63.67) | 0.8857 | 70.31 (67.54 – 72.5) | 0.6667 | 69.84 (68.35 – 71.6) | 1 | 68.66 (64.26 – 72.25) | 1 |
| **rSO2_R (%)** | 56.04 (55.22 – 58.61) |  | 69.9 (69.02 – 73.95) |  | 34.81 (30.58 – 37.1) |  | 61.08 (55.31 – 64.81) |  | 69.55 (67.95 – 71.73) |  | 65.74 (55.38 – 66.73) |  | 66.76 (64.05 – 71.56) |  | 67.67 (64.87 – 69.2) |  | 63.95 (62.99 – 64.91) |  | 74.12 (72.8 – 77.88) |  |
| **COx-a_L (au)** | 0.26 (-0.07 – 0.5) | 1 | 0.24 (-0.06 – 0.34) | 1 | 0.11 (-0.18 – 0.29) | 1 | 0.24 (0.06 – 0.39) | 0.9372 | 0.08 (-0.1 – 0.32) | 0.6991 | 0.15 (-0.12 – 0.38) | 1 | 0.13 (-0.17 – 0.41) | 0.8857 | 0.27 (-0.05 – 0.59) | 0.3333 | 0.18 (-0.06 – 0.38) | 0.62 | 0.16 (0.03 – 0.32) | 1 |
| **COx-a_R (au)** | 0.27 (0.11 – 0.58) |  | 0.33 (-0.04 – 0.45) |  | 0.13 (-0.23 – 0.35) |  | 0.26 (0.03 – 0.45) |  | 0.12 (-0.05 – 0.29) |  | 0.12 (-0.07 – 0.36) |  | 0.21 (-0.02 – 0.43) |  | 0.34 (0.06 – 0.63) |  | 0.22 (-0.04 – 0.42) |  | -0.05 (-0.27 – 0.16) |  |
| **MAD of ABP (mmHg)** | 7.35 (7.35 – 7.35) | – | 5.57 (5.57 – 5.57) | – | 5.56 (5.56 – 5.56) | – | 7.55 (5.91 – 9.68) | – | 8.5 (7.2 – 11.63) | – | 11.75 (11.75 – 11.75) | – | 7.51 (6.72 – 8.13) | – | 7.98 (6.81 – 9.15) | – | 5.57 (5.05 – 9) | – | 4.66 (4.66 – 4.66) | – |
| **MAD of rSO2_L (%)** | 1.63 (1.63 – 1.63) | 1 | 1.38 (1.38 – 1.38) | 1 | 5.53 (5.53 – 5.53) | 1 | 3.12 (2.55 – 4.97) | 0.9372 | 2.78 (2.67 – 2.89) | 0.4848 | 3.18 (3.18 – 3.18) | 1 | 2.13 (2 – 3.45) | 0.6857 | 3.66 (3.13 – 4.2) | 1 | 2.67 (1.55 – 4.05) | 0.62 | 6.5 (6.5 – 6.5) | 1 |
| **MAD of rSO2_R (%)** | 1.57 (1.57 – 1.57) |  | 1.68 (1.68 – 1.68) |  | 5.38 (5.38 – 5.38) |  | 3.41 (2.23 – 5.16) |  | 2.25 (1.87 – 2.87) |  | 2.61 (2.61 – 2.61) |  | 2.94 (2.52 – 4.4) |  | 3.26 (3.08 – 3.44) |  | 1.68 (1.47 – 3.17) |  | 3.99 (3.99 – 3.99) |  |
| **MAD of COx-a_L (au)** | 0.46 (0.46 – 0.46) | 1 | 0.29 (0.29 – 0.29) | 1 | 0.36 (0.36 – 0.36) | 1 | 0.24 (0.21 – 0.34) | 0.5887 | 0.3 (0.25 – 0.35) | 1 | 0.41 (0.41 – 0.41) | 1 | 0.43 (0.4 – 0.44) | 0.4857 | 0.45 (0.4 – 0.5) | 1 | 0.29 (0.24 – 0.36) | 1 | 0.22 (0.22 – 0.22) | 1 |
| **MAD of COx-a_R (au)** | 0.35 (0.35 – 0.35) |  | 0.29 (0.29 – 0.29) |  | 0.45 (0.45 – 0.45) |  | 0.33 (0.25 – 0.43) |  | 0.32 (0.24 – 0.36) |  | 0.32 (0.32 – 0.32) |  | 0.37 (0.34 – 0.4) |  | 0.41 (0.41 – 0.41) |  | 0.29 (0.23 – 0.36) |  | 0.35 (0.35 – 0.35) |  |
| **% time rSO2_L > 60%** | 11.11 (11.11 – 11.11; 0) | 1 | 100 (100 – 100; 0) | – | 0 (0 – 0; 0) | – | 82.89 (38.32 – 100; 25.36) | 0.6769 | 98.26 (78.86 – 100; 2.59) | 0.6553 | 69.44 (69.44 – 69.44; 0) | 1 | 54.46 (13.12 – 96.34; 62.28) | 0.5614 | 91.77 (89.82 – 93.71; 5.76) | 0.6667 | 100 (52.27 – 100; 0) | 0.7093 | 100 (100 – 100; 0) | – |
| **% time rSO2_R > 60%** | 19.44 (19.44 – 19.44; 0) |  | 100 (100 – 100; 0) |  | 0 (0 – 0; 0) |  | 51.64 (22.23 – 91.45; 61.42) |  | 100 (97.32 – 100; 0) |  | 66.67 (66.67 – 66.67; 0) |  | 86.67 (61.76 – 98.1; 17.89) |  | 95.45 (93.18 – 97.73; 6.74) |  | 100 (33.01 – 100; 0) |  | 100 (100 – 100; 0) |  |
| **% time rSO2_L > 70%** | 0 (0 – 0; 0) | 1 | 100 (100 – 100; 0) | 1 | 0 (0 – 0; 0) | – | 4.26 (0.66 – 7.72; 6.18) | 1 | 61.77 (8.68 – 99.13; 56.68) | 1 | 11.11 (11.11 – 11.11; 0) | 1 | 9.76 (0 – 36.15; 14.46) | 0.6573 | 48.88 (32.02 – 65.74; 50.01) | 0.6667 | 41.67 (1.41 – 83.99; 61.78) | 0.5491 | 41.67 (41.67 – 41.67; 0) | 1 |
| **% time rSO2_R > 70%** | 5.56 (5.56 – 5.56; 0) |  | 43.33 (43.33 – 43.33; 0) |  | 0 (0 – 0; 0) |  | 8.33 (0 – 47.35; 12.36) |  | 42.31 (18.33 – 85.14; 48.72) |  | 2.78 (2.78 – 2.78; 0) |  | 29.36 (4.17 – 63.65; 39.41) |  | 35.23 (23.67 – 46.78; 34.26) |  | 0 (0 – 57.87; 0) |  | 89.29 (89.29 – 89.29; 0) |  |
| **% time rSO2_L > 80%** | 0 (0 – 0; 0) | – | 6.67 (6.67 – 6.67; 0) | 1 | 0 (0 – 0; 0) | – | 0 (0 – 0; 0) | 1 | 11.41 (2.33 – 14.92; 11.4) | 0.4055 | 0 (0 – 0; 0) | – | 1.22 (0 – 14.17; 1.81) | 1 | 4.35 (2.17 – 6.52; 6.45) | 0.6171 | 0 (0 – 3.33; 0) | 0.5938 | 0 (0 – 0; 0) | 1 |
| **% time rSO2_R > 80%** | 0 (0 – 0; 0) |  | 0 (0 – 0; 0) |  | 0 (0 – 0; 0) |  | 0 (0 – 0; 0) |  | 0.6 (0 – 2.22; 0.88) |  | 0 (0 – 0; 0) |  | 10.98 (0 – 26.59; 16.27) |  | 0 (0 – 0; 0) |  | 0 (0 – 0; 0) |  | 25 (25 – 25; 0) |  |
| **% time rSO2_L > 90%** | 0 (0 – 0; 0) | – | 0 (0 – 0; 0) | – | 0 (0 – 0; 0) | – | 0 (0 – 0; 0) | – | 0 (0 – 2.03; 0) | 0.6733 | 0 (0 – 0; 0) | – | 0 (0 – 0; 0) | 0.1859 | 0 (0 – 0; 0) | – | 0 (0 – 0; 0) | 1 | 0 (0 – 0; 0) | 1 |
| **% time rSO2_R > 90%** | 0 (0 – 0; 0) |  | 0 (0 – 0; 0) |  | 0 (0 – 0; 0) |  | 0 (0 – 0; 0) |  | 0 (0 – 0; 0) |  | 0 (0 – 0; 0) |  | 3.16 (0 – 6.58; 4.69) |  | 0 (0 – 0; 0) |  | 0 (0 – 0; 0) |  | 3.57 (3.57 – 3.57; 0) |  |
| **% time COx-a_L > 0** | 69.44 (69.44 – 69.44; 0) | 1 | 73.08 (73.08 – 73.08; 0) | – | 60.53 (60.53 – 60.53; 0) | 1 | 78.63 (70.32 – 86.49; 14.69) | 0.9372 | 62.14 (59.23 – 67.25; 6.87) | 0.8182 | 63.89 (63.89 – 63.89; 0) | 1 | 59.72 (49.57 – 71.7; 15.69) | 0.6612 | 70.21 (69.1 – 71.31; 3.27) | 0.6667 | 73.08 (67.95 – 82.45; 12.96) | 0.949 | 79.17 (79.17 – 79.17; 0) | 1 |
| **% time COx-a_R > 0** | 83.33 (83.33 – 83.33; 0) |  | 73.08 (73.08 – 73.08; 0) |  | 55.26 (55.26 – 55.26; 0) |  | 81.5 (63.45 – 84.69; 13.73) |  | 67.83 (48.11 – 74.2; 12.23) |  | 66.67 (66.67 – 66.67; 0) |  | 71.43 (64.15 – 75.9; 10.3) |  | 75.66 (73.83 – 77.48; 5.42) |  | 73.08 (67.04 – 83.68; 11.8) |  | 43.48 (43.48 – 43.48; 0) |  |
| **% time COx-a_L > 0.2** | 52.78 (52.78 – 52.78; 0) | 1 | 53.85 (53.85 – 53.85; 0) | 1 | 42.11 (42.11 – 42.11; 0) | 1 | 58.13 (45.64 – 65; 13.39) | 0.6884 | 35.24 (29.76 – 42.82; 11.96) | 0.9372 | 36.11 (36.11 – 36.11; 0) | 1 | 40.18 (26.25 – 56.99; 22.65) | 0.6857 | 61.03 (60.52 – 61.55; 1.53) | 1 | 46.15 (41.96 – 57.48; 11.4) | 0.4557 | 37.5 (37.5 – 37.5; 0) | 1 |
| **% time COx-a_R > 0.2** | 63.89 (63.89 – 63.89; 0) |  | 65.38 (65.38 – 65.38; 0) |  | 39.47 (39.47 – 39.47; 0) |  | 56.06 (43.24 – 61.76; 17.56) |  | 32.7 (17.12 – 53.69; 28.22) |  | 38.89 (38.89 – 38.89; 0) |  | 52.22 (46.98 – 56.8; 10.3) |  | 64.48 (62.24 – 66.72; 6.65) |  | 55.17 (44.78 – 60.47; 15.14) |  | 26.09 (26.09 – 26.09; 0) |  |
| **% time COx-a_L > 0.3** | 41.67 (41.67 – 41.67; 0) | 1 | 34.62 (34.62 – 34.62; 0) | 1 | 21.05 (21.05 – 21.05; 0) | 1 | 37.08 (28.9 – 40.26; 11.02) | 0.3095 | 26.46 (16.89 – 30.62; 12.71) | 0.6991 | 27.78 (27.78 – 27.78; 0) | 1 | 34.63 (24.16 – 45.81; 20.59) | 1 | 46.14 (45.07 – 47.21; 3.17) | 0.3333 | 33.33 (28.85 – 42.31; 9.5) | 0.4428 | 29.17 (29.17 – 29.17; 0) | 1 |
| **% time COx-a_R > 0.3** | 47.22 (47.22 – 47.22; 0) |  | 53.85 (53.85 – 53.85; 0) |  | 34.21 (34.21 – 34.21; 0) |  | 46.06 (37.02 – 47.19; 7.26) |  | 22.07 (9.73 – 43.25; 23.63) |  | 36.11 (36.11 – 36.11; 0) |  | 38.31 (33.98 – 42.19; 8.24) |  | 55.59 (55.38 – 55.79; 0.61) |  | 36.11 (32.43 – 50.61; 18.04) |  | 13.04 (13.04 – 13.04; 0) |  |
| *The p-values in the table are derived using Mann-Whitney U test between the bilateral signals.*  *ABP, arterial blood pressure; au, arbitrary units; ACDF, anterior cervical discectomy and fusion; CPP, cerebral perfusion pressure; COx, cerebral oximetry index with CPP; COx-a, cerebral oximetry index with ABP; IQR, interquartile range; mmHg, millimeters of mercury; MAD, median absolute deviation; PCDF, posterior cervical discectomy and fusion; rSO2, regional cerebral oxygen saturation; SP, elective spinal surgery patient group.* | | | | | | | | | | | | | | | | | | | | |

File S8i: Sub-grouped Physiologic Results using 10-Second, 1-Minute Data, and 5-Minute Resolutions for TBI-GLR Population

| **Physiologic Variable** | **Sub-groups** | | | | | | | | | | | | | | | | | |
| --- | --- | --- | --- | --- | --- | --- | --- | --- | --- | --- | --- | --- | --- | --- | --- | --- | --- | --- |
|  | **Age < 40 [n = 28]** | | **Age 40 – 60 [n = 23]** | | **Age > 60 [n = 13]** | | **Males [n = 51]** | | **Females [n = 13]** | | **Focal Injury (aSDH, SDH, EDH, or Contusion) [n = 42]** | | **Diffuse Injury (DAI or tSAH) [n = 22]** | | **Marshall CT V [n = 29]** | | **Marshall CT IV [n = 8]** | |
|  | **Median (IQR) or  Median (IQR; MAD)** | **p-value** | **Median (IQR) or  Median (IQR; MAD)** | **p-value** | **Median (IQR) or  Median (IQR; MAD)** | **p-value** | **Median (IQR) or  Median (IQR; MAD)** | **p-value** | **Median (IQR) or  Median (IQR; MAD)** | **p-value** | **Median (IQR) or  Median (IQR; MAD)** | **p-value** | **Median (IQR) or  Median (IQR; MAD)** | **p-value** | **Median (IQR) or  Median (IQR; MAD)** | **p-value** | **Median (IQR) or  Median (IQR; MAD)** | **p-value** |
| **10-Second Data Resolution** | | | | | | | | | | | | | | | | | | |
| **ABP (mmHg)** | 83.5 (78.27 – 90.24) | – | 81.73 (74.53 – 90.41) | – | 79.02 (73.38 – 84.22) | – | 82.02 (75.81 – 89.81) | – | 81.99 (75.71 – 87.76) | – | 81.04 (75.66 – 89.02) | – | 83.23 (76.98 – 89.75) | – | 79.64 (74.7 – 87.76) | – | 87.08 (80.96 – 93.7) | – |
| **CPP (mmHg)** | 73.85 (66.9 – 80.61) |  | 73.18 (67.31 – 80.99) |  | 73.4 (67.54 – 81.5) |  | 73.97 (67.73 – 81.96) |  | 70.73 (65.17 – 76.83) |  | 73.68 (67.25 – 80.98) |  | 73.08 (67.48 – 80.12) |  | 73.32 (66.62 – 81.63) |  | 74.64 (68.21 – 82.28) |  |
| **rSO2_L (%)** | 68.2 (65 – 74.07) | 0.6581 | 69.97 (66.86 – 71.15) | 0.6366 | 73.55 (69.85 – 75.84) | 0.6444 | 71.99 (68.5 – 75.84) | 0.8278 | 62.5 (56.53 – 66.37) | 0.4118 | 67.99 (65 – 72.07) | 0.7037 | 71.99 (67.6 – 76.39) | 0.8972 | 67.99 (62.99 – 75.57) | 0.4889 | 70.75 (68.5 – 74.81) | 0.9163 |
| **rSO2_R (%)** | 70 (64.5 – 75.44) |  | 68.11 (65.99 – 72.99) |  | 69 (62.99 – 70.99) |  | 70.07 (65.99 – 74.99) |  | 66.99 (63.14 – 70.99) |  | 68.7 (63.99 – 72.24) |  | 71.37 (66.25 – 75.67) |  | 69.99 (65.99 – 72.99) |  | 70.66 (65.49 – 73.78) |  |
| **COx_L (au)** | 0 (-0.19 – 0.23) | 0.5441 | 0 (-0.19 – 0.22) | 0.523 | 0 (-0.08 – 0.29) | 0.4021 | 0 (-0.17 – 0.24) | 0.713 | 0 (-0.23 – 0.2) | 0.9585 | 0 (-0.15 – 0.24) | 0.3253 | 0.01 (-0.18 – 0.22) | 0.5017 | 0 (-0.14 – 0.24) | 0.118 | 0 (-0.17 – 0.27) | 0.4761 |
| **COx_R (au)** | 0 (-0.19 – 0.23) |  | 0 (-0.2 – 0.23) |  | 0.02 (-0.06 – 0.32) |  | 0.01 (-0.18 – 0.23) |  | 0 (-0.18 – 0.22) |  | 0 (-0.17 – 0.24) |  | 0 (-0.18 – 0.23) |  | 0.01 (-0.13 – 0.27) |  | 0 (-0.23 – 0.23) |  |
| **COx-a_L (au)** | 0.04 (-0.11 – 0.28) | 0.7491 | 0.06 (-0.12 – 0.28) | 1 | 0.02 (-0.04 – 0.3) | 0.4239 | 0.05 (-0.11 – 0.28) | 0.9599 | 0.04 (-0.1 – 0.28) | 0.8775 | 0.03 (-0.11 – 0.27) | 0.6505 | 0.08 (-0.07 – 0.3) | 0.7158 | 0.02 (-0.1 – 0.25) | 0.4261 | 0.04 (-0.13 – 0.29) | 0.7124 |
| **COx-a_R (au)** | 0.04 (-0.12 – 0.27) |  | 0.05 (-0.15 – 0.26) |  | 0.1 (-0.03 – 0.3) |  | 0.05 (-0.12 – 0.27) |  | 0.07 (-0.07 – 0.3) |  | 0.03 (-0.12 – 0.27) |  | 0.07 (-0.09 – 0.31) |  | 0.05 (-0.11 – 0.28) |  | 0.02 (-0.14 – 0.25) |  |
| **MAD of ABP (mmHg)** | 8.47 (7.38 – 10.43) | – | 9.84 (8.01 – 11.22) | – | 8.63 (6.26 – 10.88) | – | 9.4 (7.33 – 11.1) | – | 7.86 (6.92 – 10.98) | – | 8.81 (6.81 – 11.06) | – | 9.41 (8.02 – 10.79) | – | 9.3 (6.54 – 11.58) | – | 9.33 (7.95 – 10.3) | – |
| **MAD of CPP (mmHg)** | 8.83 (7.29 – 12.15) |  | 8.7 (7.65 – 11.3) |  | 6.49 (6.36 – 11.34) |  | 8.93 (6.66 – 12.09) |  | 8.35 (7.4 – 10.07) |  | 8.61 (6.42 – 12.21) |  | 9.41 (7.94 – 11.1) |  | 7.49 (6.38 – 12.35) |  | 9.26 (7.71 – 10.94) |  |
| **MAD of rSO2_L (%)** | 5.63 (3.9 – 8.37) | 0.4708 | 4.46 (3.18 – 6.34) | 0.9212 | 3.17 (2.91 – 5.95) | 0.959 | 5.07 (3.02 – 6.94) | 0.6708 | 4.89 (3.5 – 10.27) | 0.801 | 3.84 (2.81 – 6.55) | 0.4796 | 5.63 (4.57 – 8.51) | 0.8326 | 4.46 (2.64 – 5.95) | 0.8641 | 4.83 (2.89 – 9.16) | 0.7209 |
| **MAD of rSO2_R (%)** | 6 (4.67 – 8.4) |  | 4.45 (3.22 – 6.32) |  | 4.27 (2.97 – 4.45) |  | 4.49 (3.12 – 7.03) |  | 5.78 (3.6 – 7.21) |  | 4.3 (2.97 – 7.42) |  | 5.86 (4.79 – 6.6) |  | 4.26 (2.95 – 7.4) |  | 5.66 (4.08 – 6.35) |  |
| **MAD of COx_L (au)** | 0.31 (0.27 – 0.37) | 0.6903 | 0.31 (0.28 – 0.34) | 0.4857 | 0.3 (0.26 – 0.32) | 0.8798 | 0.31 (0.26 – 0.36) | 0.2296 | 0.32 (0.28 – 0.34) | 0.4417 | 0.31 (0.27 – 0.36) | 0.645 | 0.31 (0.28 – 0.34) | 0.4494 | 0.31 (0.26 – 0.36) | 0.4583 | 0.32 (0.29 – 0.35) | 0.9591 |
| **MAD of COx_R (au)** | 0.33 (0.29 – 0.37) |  | 0.32 (0.29 – 0.36) |  | 0.32 (0.23 – 0.33) |  | 0.33 (0.28 – 0.37) |  | 0.31 (0.29 – 0.33) |  | 0.33 (0.26 – 0.35) |  | 0.32 (0.28 – 0.36) |  | 0.33 (0.27 – 0.36) |  | 0.33 (0.26 – 0.36) |  |
| **MAD of COx-a_L (au)** | 0.3 (0.25 – 0.35) | 0.4203 | 0.28 (0.27 – 0.31) | 0.6764 | 0.29 (0.25 – 0.33) | 0.8403 | 0.29 (0.25 – 0.33) | 0.1785 | 0.29 (0.26 – 0.31) | 0.6139 | 0.29 (0.26 – 0.33) | 0.4551 | 0.29 (0.26 – 0.31) | 0.5073 | 0.29 (0.25 – 0.33) | 0.3671 | 0.29 (0.27 – 0.32) | 1 |
| **MAD of COx-a_R (au)** | 0.31 (0.27 – 0.35) |  | 0.29 (0.27 – 0.31) |  | 0.31 (0.26 – 0.32) |  | 0.3 (0.27 – 0.34) |  | 0.28 (0.26 – 0.3) |  | 0.3 (0.27 – 0.33) |  | 0.29 (0.26 – 0.32) |  | 0.3 (0.27 – 0.35) |  | 0.29 (0.28 – 0.33) |  |
| **% time rSO2_L > 60%** | 87.72 (70.24 – 97.21; 18) | 0.4755 | 95.37 (52.25 – 98.76; 5.9) | 0.75 | 98.58 (50.91 – 99.89; 2.1) | 0.9591 | 95.37 (75.45 – 99.68; 6.85) | 0.9813 | 63.81 (38.49 – 86.23; 37.53) | 0.3559 | 93.33 (52.37 – 99.68; 9.9) | 0.5575 | 91.2 (74.43 – 96.44; 10.99) | 0.6726 | 93.08 (39.55 – 99.89; 10.26) | 0.3104 | 91.72 (84.66 – 99.68; 11.79) | 0.9591 |
| **% time rSO2_R > 60%** | 92.59 (62.06 – 99.88; 10.94) |  | 93.79 (74.58 – 99.63; 8.94) |  | 93.15 (54.12 – 99.95; 10.14) |  | 93.3 (74.58 – 99.85; 9.86) |  | 93.65 (30.08 – 96.49; 9.41) |  | 93.72 (63.04 – 99.95; 9.31) |  | 92.59 (66.77 – 99.31; 10.76) |  | 97.83 (61.86 – 99.99; 3.22) |  | 92.4 (86.84 – 95.33; 7.31) |  |
| **% time rSO2_L > 70%** | 42.35 (9.22 – 76.47; 50.62) | 0.5526 | 46.85 (7.16 – 82.32; 60.95) | 0.553 | 69.49 (0 – 94.89; 45.03) | 0.6059 | 62.8 (9.21 – 85.66; 49.75) | 0.96 | 9.59 (1.22 – 43.49; 14.17) | 0.7005 | 36.66 (3.51 – 76.52; 54.19) | 0.6838 | 60.54 (19.84 – 83.24; 41.54) | 0.9352 | 32.11 (0.14 – 95.36; 47.61) | 0.3545 | 54.72 (42.92 – 76.8; 24.44) | 0.2786 |
| **% time rSO2_R > 70%** | 48.84 (32.11 – 72.03; 31.43) |  | 36.35 (10.48 – 93.76; 52.51) |  | 36.32 (2.08 – 69.66; 50.77) |  | 50.21 (20.79 – 82.75; 46.63) |  | 32.39 (0.93 – 42.45; 46.64) |  | 37.45 (8.62 – 80.52; 47.03) |  | 54.47 (32.95 – 72.05; 31.81) |  | 41.59 (14.58 – 90.37; 52.74) |  | 47.57 (25.27 – 68.19; 32.47) |  |
| **% time rSO2_L > 80%** | 2.09 (0.6 – 21.24; 3.11) | 0.6225 | 1.22 (0 – 20.5; 1.81) | 0.5485 | 10.19 (0 – 38.88; 15.1) | 0.2457 | 5.52 (0.02 – 28.7; 8.19) | 0.8823 | 0.48 (0 – 2.37; 0.71) | 1 | 1.23 (0 – 13.27; 1.82) | 0.9672 | 10.18 (0.54 – 26.69; 15.09) | 0.7691 | 1.24 (0 – 29.86; 1.84) | 1 | 4.7 (0.7 – 28.68; 6.96) | 0.4275 |
| **% time rSO2_R > 80%** | 6.22 (1.44 – 20.17; 9) |  | 4.67 (0.05 – 24.24; 6.93) |  | 0 (0 – 4.02; 0) |  | 5.83 (0.08 – 21.24; 8.65) |  | 0.27 (0 – 5.05; 0.39) |  | 1.41 (0 – 19.02; 2.09) |  | 5.44 (1.85 – 20.42; 7.86) |  | 6.61 (0 – 21.62; 9.8) |  | 2.39 (0.05 – 8.64; 3.54) |  |
| **% time rSO2_L > 90%** | 0.01 (0 – 0.8; 0.01) | 0.8981 | 0 (0 – 0.02; 0) | 0.1553 | 0 (0 – 0.03; 0) | 0.4149 | 0 (0 – 0.51; 0) | 0.7618 | 0 (0 – 0; 0) | 0.7778 | 0 (0 – 0.04; 0) | 0.709 | 0.01 (0 – 0.57; 0.02) | 0.6751 | 0 (0 – 0.34; 0) | 0.8833 | 0 (0 – 0.04; 0) | 0.7496 |
| **% time rSO2_R > 90%** | 0.02 (0 – 0.74; 0.03) |  | 0 (0 – 0.14; 0) |  | 0 (0 – 0; 0) |  | 0 (0 – 0.42; 0) |  | 0 (0 – 0.01; 0) |  | 0 (0 – 0.14; 0) |  | 0.02 (0 – 0.92; 0.03) |  | 0 (0 – 0.15; 0) |  | 0 (0 – 0.13; 0) |  |
| **% time COx_L > 0** | 50.12 (45.82 – 54.95; 6.91) | 0.5099 | 47.24 (41.51 – 53.52; 9.28) | 0.8448 | 49.81 (47 – 67.24; 16.84) | 0.4483 | 49.81 (45.95 – 56.58; 8.08) | 0.9893 | 43.91 (42.54 – 52.37; 5.44) | 0.9197 | 49.11 (42.9 – 58.71; 9.88) | 0.6025 | 50.63 (43.61 – 55.15; 7.22) | 0.6337 | 48.92 (43.91 – 60.52; 9.77) | 0.3376 | 50.11 (45.93 – 53.66; 7.17) | 0.3823 |
| **% time COx_R > 0** | 47.48 (42.45 – 53.92; 8.05) |  | 49.53 (38.83 – 53.95; 9.51) |  | 52.83 (47.77 – 68.02; 12.34) |  | 50.75 (44.54 – 57.67; 9.85) |  | 46.82 (40.46 – 50.58; 9.43) |  | 50.24 (44.62 – 57.08; 8.79) |  | 47.02 (42.11 – 56.43; 11.57) |  | 51.09 (46.52 – 58.95; 9.77) |  | 45.91 (42.34 – 49.06; 5.08) |  |
| **% time COx_L > 0.2** | 27.74 (24.55 – 35.25; 6.43) | 0.9159 | 26.81 (19.9 – 29.84; 6.59) | 0.7114 | 34.86 (25.39 – 42.86; 14.03) | 0.5446 | 27.9 (24.69 – 35.99; 7.74) | 0.9307 | 24.58 (21.95 – 34.94; 6.75) | 0.8798 | 28.03 (24.11 – 34.92; 7.66) | 0.7052 | 26.58 (23.12 – 36.55; 10.98) | 0.9907 | 27.9 (24.64 – 36.17; 8.83) | 0.3952 | 30.82 (24.72 – 37.71; 10.7) | 0.7984 |
| **% time COx_R > 0.2** | 27.56 (20.32 – 37.06; 11.89) |  | 27.77 (19.33 – 32.59; 11.56) |  | 32.98 (24.5 – 47.2; 16.39) |  | 28.39 (22.04 – 38.71; 11.48) |  | 26.7 (18.69 – 32.34; 11.88) |  | 28.79 (22.39 – 39.43; 10.72) |  | 27.56 (18.67 – 35; 13.16) |  | 31.33 (24.64 – 40.34; 13.07) |  | 27.39 (21 – 33.79; 10.84) |  |
| **% time COx_L > 0.3** | 18.54 (16.68 – 26.37; 7.39) | 0.9935 | 17.95 (12.97 – 20.88; 5.68) | 0.6792 | 23.74 (16.14 – 32.61; 13.15) | 0.5788 | 18.79 (16.46 – 27.27; 7.34) | 0.7735 | 17.78 (13.83 – 24.11; 8.65) | 1 | 19.36 (16.37 – 24.32; 7.01) | 0.6725 | 18.06 (14.2 – 27.86; 10.44) | 0.9907 | 18.79 (16.83 – 27.85; 7.34) | 0.4489 | 22.54 (15.47 – 29.38; 11.48) | 0.9591 |
| **% time COx_R > 0.3** | 19.61 (13.76 – 28.17; 9.01) |  | 17.81 (13.25 – 23.35; 7.65) |  | 26.18 (16.3 – 39.37; 15.16) |  | 19.86 (14.92 – 29.41; 9.14) |  | 17.58 (10.78 – 26.18; 10.78) |  | 20.69 (15.23 – 31.59; 9.43) |  | 18.53 (12.55 – 25.82; 10.38) |  | 22.11 (16 – 32.15; 11.75) |  | 19.88 (15.38 – 26.85; 8.59) |  |
| **% time COx-a_L > 0** | 55.07 (51.13 – 61.06; 7.41) | 0.8137 | 58.05 (48.17 – 65.77; 14.05) | 0.6792 | 51.07 (45.91 – 71.07; 18.42) | 0.4184 | 55.69 (49.86 – 64.11; 10.88) | 0.9786 | 53.51 (47.42 – 64.72; 14.48) | 0.9197 | 52.98 (47.91 – 63.04; 10.85) | 0.8274 | 58.37 (53.16 – 67.42; 11.59) | 0.8435 | 52.44 (47.61 – 63.49; 11.4) | 0.5677 | 54.67 (49.99 – 58.23; 5.53) | 0.5054 |
| **% time COx-a_R > 0** | 55.73 (50.21 – 61.13; 8.17) |  | 55.69 (48.58 – 60.78; 8.61) |  | 65.96 (50.98 – 73.94; 19.38) |  | 55.69 (50.1 – 64.55; 8.49) |  | 57.23 (47.16 – 65.05; 14.11) |  | 54.75 (49.23 – 61.18; 9.04) |  | 57.83 (51.39 – 69.7; 13.68) |  | 55.69 (50.21 – 61.32; 8.34) |  | 50.79 (47.43 – 55.8; 8.64) |  |
| **% time COx-a_L > 0.2** | 33.35 (28.81 – 37.73; 6.83) | 1 | 30.95 (26.82 – 38.69; 9.89) | 0.9134 | 36.78 (25.26 – 48.65; 17.59) | 0.5446 | 32.99 (27.55 – 39.56; 8.64) | 0.9147 | 34.57 (27.91 – 37.73; 7.68) | 0.8403 | 30.74 (26.39 – 38.05; 8.03) | 0.6403 | 36.54 (29.51 – 43.5; 11.14) | 0.88 | 29.68 (26.03 – 39.48; 10.04) | 0.3782 | 33.23 (29.21 – 36.14; 5.06) | 0.7984 |
| **% time COx-a_R > 0.2** | 32.41 (28.19 – 38.45; 8.11) |  | 30.28 (24.73 – 40.64; 14.2) |  | 33.28 (28.74 – 53.34; 18.48) |  | 31.14 (28.2 – 41.23; 10.83) |  | 36.29 (26.87 – 41.02; 8.27) |  | 31.05 (28.17 – 38.59; 9.78) |  | 36.78 (27.94 – 42.14; 12.34) |  | 32.69 (28.25 – 40.45; 8.84) |  | 29.52 (26.31 – 37.43; 11.44) |  |
| **% time COx-a_L > 0.3** | 23.24 (19.4 – 27.83; 6.58) | 0.9417 | 23.94 (16.77 – 25.71; 6.64) | 0.8962 | 24.98 (17.45 – 35.67; 15.59) | 0.5788 | 23.17 (18.21 – 29.42; 8.48) | 0.8409 | 23.42 (19.85 – 26.99; 5.29) | 0.5446 | 22.2 (17.58 – 25.62; 6.67) | 0.6986 | 25.45 (19.15 – 30.97; 9.29) | 0.8983 | 21.27 (17.11 – 29.09; 7.16) | 0.5065 | 23.83 (19.3 – 27.83; 6.5) | 0.7209 |
| **% time COx-a_R > 0.3** | 22.86 (18.93 – 29.8; 7.08) |  | 22.11 (16.91 – 28.17; 10.43) |  | 25.31 (19.13 – 41.68; 15.13) |  | 22.43 (18.85 – 31.4; 10.72) |  | 25.34 (17.07 – 27.19; 12.26) |  | 22.53 (18.84 – 29.53; 7.09) |  | 26.27 (18.02 – 32.96; 10.59) |  | 22.93 (19.05 – 30.21; 9.22) |  | 20.75 (17.89 – 28.33; 8.49) |  |
| **1-Minute Data Resolution** | | | | | | | | | | | | | | | | | | |
| **ABP (mmHg)** | 83.71 (78.38 – 90.24) | – | 81.96 (74.7 – 90.44) | – | 79 (73.6 – 84.07) | – | 82.02 (75.89 – 89.84) | – | 82.16 (75.95 – 88.07) | – | 81.2 (75.84 – 89.03) | – | 83.23 (77.13 – 89.72) | – | 79.92 (74.79 – 88.07) | – | 87.04 (81.11 – 93.73) | – |
| **CPP (mmHg)** | 73.9 (67.07 – 80.47) |  | 73.18 (67.42 – 80.79) |  | 73.66 (67.61 – 81.42) |  | 73.79 (68.28 – 81.86) |  | 70.66 (65.31 – 76.56) |  | 73.7 (67.37 – 80.94) |  | 73.16 (67.81 – 80.18) |  | 73.66 (66.81 – 81.42) |  | 74.53 (68.49 – 81.63) |  |
| **rSO2_L (%)** | 68.17 (65.07 – 74.06) | 0.6941 | 69.83 (66.99 – 71.26) | 0.6445 | 73.58 (69.69 – 75.66) | 0.6498 | 71.99 (68.5 – 75.66) | 0.8252 | 62.59 (56.59 – 66.35) | 0.3622 | 67.98 (65.07 – 72.12) | 0.6807 | 72.04 (67.72 – 76.36) | 0.88 | 67.91 (62.99 – 75.58) | 0.4889 | 70.81 (68.6 – 74.79) | 0.9163 |
| **rSO2_R (%)** | 69.8 (64.72 – 75.44) |  | 68.32 (65.99 – 72.99) |  | 69 (63.07 – 70.99) |  | 70.27 (65.99 – 75) |  | 67.15 (63.34 – 71.03) |  | 68.56 (63.99 – 72.25) |  | 71.21 (66.37 – 75.61) |  | 69.76 (66.05 – 72.99) |  | 70.66 (65.53 – 73.8) |  |
| **COx_L (au)** | 0.02 (-0.19 – 0.24) | 0.6314 | 0.01 (-0.2 – 0.22) | 0.794 | 0.12 (-0.08 – 0.33) | 0.6498 | 0.03 (-0.17 – 0.24) | 0.8909 | 0 (-0.22 – 0.23) | 0.6498 | 0.02 (-0.17 – 0.24) | 0.6986 | 0.02 (-0.18 – 0.22) | 0.6672 | 0.03 (-0.16 – 0.24) | 0.4869 | 0.03 (-0.18 – 0.27) | 0.7984 |
| **COx_R (au)** | 0.02 (-0.21 – 0.23) |  | 0.01 (-0.2 – 0.22) |  | 0.15 (-0.09 – 0.36) |  | 0.03 (-0.18 – 0.25) |  | 0.01 (-0.21 – 0.21) |  | 0.03 (-0.18 – 0.26) |  | 0.02 (-0.2 – 0.22) |  | 0.03 (-0.17 – 0.27) |  | 0.01 (-0.23 – 0.26) |  |
| **COx-a_L (au)** | 0.08 (-0.12 – 0.29) | 0.597 | 0.07 (-0.13 – 0.29) | 0.8278 | 0.17 (-0.04 – 0.35) | 0.801 | 0.08 (-0.12 – 0.29) | 0.7078 | 0.11 (-0.1 – 0.29) | 0.6866 | 0.07 (-0.13 – 0.28) | 0.8903 | 0.11 (-0.09 – 0.3) | 0.8073 | 0.07 (-0.13 – 0.27) | 0.5996 | 0.08 (-0.13 – 0.29) | 0.5737 |
| **COx-a_R (au)** | 0.08 (-0.13 – 0.28) |  | 0.05 (-0.16 – 0.26) |  | 0.18 (-0.04 – 0.38) |  | 0.07 (-0.14 – 0.27) |  | 0.12 (-0.1 – 0.31) |  | 0.07 (-0.15 – 0.27) |  | 0.11 (-0.1 – 0.31) |  | 0.08 (-0.13 – 0.28) |  | 0.03 (-0.16 – 0.26) |  |
| **MAD of ABP (mmHg)** | 8.29 (7.17 – 10.24) | – | 9.41 (7.82 – 11.27) | – | 8.48 (6.03 – 10.67) | – | 9.15 (7.16 – 11.1) | – | 7.56 (6.7 – 10.76) | – | 8.5 (6.41 – 11.13) | – | 9.28 (7.83 – 10.58) | – | 9.15 (6.24 – 11.38) | – | 9.1 (7.77 – 10.01) | – |
| **MAD of CPP (mmHg)** | 8.55 (7.05 – 11.83) |  | 8.55 (7.38 – 11.28) |  | 6.43 (6.18 – 11.05) |  | 8.55 (6.61 – 11.94) |  | 8 (7.24 – 10.02) |  | 8.39 (6.34 – 11.8) |  | 9.16 (7.6 – 10.87) |  | 7.53 (6.32 – 11.83) |  | 9.29 (7.41 – 10.53) |  |
| **MAD of rSO2_L (%)** | 5.55 (3.82 – 8.22) | 0.5204 | 4.64 (3.25 – 6.35) | 0.9307 | 3.1 (2.64 – 6.04) | 1 | 4.75 (3.04 – 6.8) | 0.6394 | 4.94 (3.24 – 10.03) | 0.7241 | 3.91 (2.75 – 6.59) | 0.5761 | 5.55 (4.5 – 8.24) | 0.8983 | 4.45 (2.72 – 6.04) | 1 | 4.98 (2.84 – 8.89) | 0.7209 |
| **MAD of rSO2_R (%)** | 6.05 (4.72 – 8.38) |  | 4.45 (3.35 – 6.34) |  | 3.85 (3 – 4.45) |  | 4.49 (3.21 – 6.91) |  | 5.79 (3.75 – 7) |  | 4.23 (3.02 – 7.38) |  | 5.75 (4.63 – 6.55) |  | 3.97 (2.69 – 7.17) |  | 5.7 (4.11 – 6.55) |  |
| **MAD of COx_L (au)** | 0.34 (0.29 – 0.37) | 0.8773 | 0.3 (0.29 – 0.34) | 0.3371 | 0.31 (0.27 – 0.32) | 0.5788 | 0.31 (0.29 – 0.35) | 0.2232 | 0.32 (0.3 – 0.36) | 0.7241 | 0.32 (0.29 – 0.35) | 0.4901 | 0.3 (0.28 – 0.35) | 0.6009 | 0.32 (0.3 – 0.36) | 0.3782 | 0.34 (0.28 – 0.36) | 0.9591 |
| **MAD of COx_R (au)** | 0.34 (0.3 – 0.37) |  | 0.32 (0.3 – 0.35) |  | 0.32 (0.3 – 0.33) |  | 0.33 (0.3 – 0.36) |  | 0.31 (0.28 – 0.35) |  | 0.33 (0.31 – 0.35) |  | 0.33 (0.28 – 0.36) |  | 0.33 (0.31 – 0.35) |  | 0.34 (0.3 – 0.39) |  |
| **MAD of COx-a_L (au)** | 0.31 (0.28 – 0.34) | 0.7431 | 0.28 (0.27 – 0.31) | 0.4995 | 0.3 (0.28 – 0.32) | 0.8403 | 0.29 (0.27 – 0.33) | 0.2348 | 0.28 (0.28 – 0.34) | 0.8403 | 0.29 (0.28 – 0.33) | 0.3203 | 0.29 (0.26 – 0.33) | 0.8144 | 0.29 (0.28 – 0.33) | 0.2584 | 0.32 (0.29 – 0.34) | 1 |
| **MAD of COx-a_R (au)** | 0.32 (0.29 – 0.34) |  | 0.29 (0.28 – 0.32) |  | 0.3 (0.29 – 0.33) |  | 0.3 (0.29 – 0.34) |  | 0.3 (0.26 – 0.32) |  | 0.3 (0.29 – 0.34) |  | 0.3 (0.27 – 0.33) |  | 0.3 (0.29 – 0.34) |  | 0.31 (0.28 – 0.34) |  |
| **% time rSO2_L > 60%** | 88.12 (71.53 – 97.16; 17.54) | 0.4703 | 95.42 (53.56 – 98.72; 5.99) | 0.7251 | 98.56 (51.42 – 99.95; 2.14) | 0.9382 | 95.42 (75.85 – 99.65; 6.79) | 0.9439 | 64.34 (39.54 – 86.31; 36.76) | 0.3171 | 93.77 (52.92 – 99.66; 9.24) | 0.5012 | 91.74 (74.94 – 96.38; 10.26) | 0.7247 | 93.78 (39.54 – 99.83; 9.22) | 0.2529 | 91.8 (85.14 – 99.64; 11.64) | 0.9591 |
| **% time rSO2_R > 60%** | 93 (62.29 – 99.89; 10.38) |  | 94.16 (75.7 – 99.67; 8.33) |  | 93.13 (54.22 – 99.93; 10.18) |  | 93.68 (75.7 – 99.83; 9.37) |  | 93.88 (32.62 – 96.47; 9.07) |  | 94.02 (63.92 – 99.95; 8.86) |  | 93 (65.55 – 99.34; 10.14) |  | 97.86 (62.73 – 100; 3.17) |  | 92.58 (87.47 – 96.27; 7.14) |  |
| **% time rSO2_L > 70%** | 43.07 (9.35 – 78.12; 51.5) | 0.5526 | 48.97 (7.39 – 84.21; 62.38) | 0.5678 | 70.14 (0 – 95.32; 44.01) | 0.588 | 64.99 (9.48 – 86.48; 48.21) | 0.976 | 9.67 (1.66 – 43.93; 14.34) | 0.7388 | 37.17 (3.69 – 78.68; 54.96) | 0.6706 | 61.41 (20.65 – 83.88; 41.38) | 0.9352 | 32.13 (0.17 – 96.31; 47.64) | 0.3229 | 57.69 (43.5 – 79.26; 26.07) | 0.2786 |
| **% time rSO2_R > 70%** | 49.18 (32.77 – 72.53; 33.7) |  | 37.08 (10.75 – 94.21; 53.53) |  | 38.56 (2.32 – 70.62; 53.73) |  | 52.65 (21.33 – 83.77; 46.22) |  | 33.39 (0.98 – 43.39; 48.06) |  | 39.46 (8.79 – 82.35; 49.16) |  | 55.36 (33.59 – 72.67; 32.2) |  | 43.39 (14.92 – 90.85; 55.05) |  | 49.01 (26.37 – 69.1; 31.47) |  |
| **% time rSO2_L > 80%** | 2.26 (0.71 – 21.67; 3.36) | 0.6395 | 1.29 (0 – 21.43; 1.91) | 0.5786 | 10.96 (0 – 40.4; 16.25) | 0.2457 | 5.89 (0.04 – 29.22; 8.73) | 0.9115 | 0.49 (0 – 2.41; 0.73) | 0.9151 | 1.22 (0 – 14.34; 1.81) | 0.9562 | 10.59 (0.57 – 27.27; 15.7) | 0.7691 | 1.29 (0 – 30.22; 1.91) | 0.9426 | 4.99 (0.83 – 29.02; 7.4) | 0.4275 |
| **% time rSO2_R > 80%** | 6.54 (1.54 – 20.03; 9.67) |  | 4.91 (0.06 – 25.53; 7.28) |  | 0 (0 – 4.26; 0) |  | 6.35 (0.1 – 22.25; 9.42) |  | 0.24 (0 – 5.73; 0.36) |  | 1.49 (0 – 19.64; 2.21) |  | 6.04 (1.92 – 20.63; 8.77) |  | 6.73 (0 – 22.82; 9.98) |  | 2.51 (0.06 – 9.26; 3.73) |  |
| **% time rSO2_L > 90%** | 0.01 (0 – 0.84; 0.02) | 0.9376 | 0 (0 – 0.02; 0) | 0.356 | 0 (0 – 0.03; 0) | 0.6459 | 0 (0 – 0.58; 0) | 0.9439 | 0 (0 – 0; 0) | 0.5546 | 0 (0 – 0.02; 0) | 0.9866 | 0.01 (0 – 0.6; 0.02) | 0.6373 | 0 (0 – 0.42; 0) | 0.6847 | 0 (0 – 0.02; 0) | 1 |
| **% time rSO2_R > 90%** | 0.01 (0 – 0.74; 0.01) |  | 0 (0 – 0.13; 0) |  | 0 (0 – 0; 0) |  | 0 (0 – 0.41; 0) |  | 0 (0 – 0.02; 0) |  | 0 (0 – 0.15; 0) |  | 0.03 (0 – 1.03; 0.04) |  | 0 (0 – 0.17; 0) |  | 0 (0 – 0.14; 0) |  |
| **% time COx_L > 0** | 53.72 (48.99 – 58.28; 7.61) | 0.597 | 51.25 (42.03 – 55.62; 12.76) | 1 | 65.32 (51.48 – 73.38; 19.12) | 0.6629 | 53.61 (49.46 – 64.56; 9.97) | 0.7607 | 50.33 (43.99 – 60.41; 11.38) | 0.7241 | 53.02 (48.23 – 64.73; 13.48) | 0.8161 | 53.72 (48.19 – 57.19; 7.61) | 0.7187 | 53.97 (49.24 – 64.81; 12.16) | 0.6104 | 53.02 (47.98 – 65.24; 19) | 0.8335 |
| **% time COx_R > 0** | 52.09 (44.33 – 58.41; 12.01) |  | 51.82 (45.51 – 56.4; 7.36) |  | 66.42 (54.52 – 76.81; 16.77) |  | 53.25 (48.52 – 62.42; 12.24) |  | 50.98 (43.37 – 70.64; 15.39) |  | 52.98 (49.23 – 65.42; 12.46) |  | 52.05 (43.01 – 57.44; 13.07) |  | 54.08 (50.72 – 65.86; 7.67) |  | 50.76 (45.88 – 63.79; 16.58) |  |
| **% time COx_L > 0.2** | 29.61 (25.04 – 36.31; 8.19) | 0.776 | 26.46 (20.75 – 30.74; 6.57) | 0.7114 | 38.98 (26.54 – 47.48; 18.45) | 0.4184 | 29.37 (25.23 – 39.96; 9.15) | 0.8409 | 26.96 (22.66 – 36.05; 11.16) | 0.9598 | 29.18 (25.16 – 40.78; 9.93) | 0.6213 | 26.63 (24.08 – 36.42; 8.5) | 0.88 | 29 (25.35 – 40.94; 10.45) | 0.3952 | 31.69 (24.68 – 43.39; 16.47) | 0.9591 |
| **% time COx_R > 0.2** | 28.51 (20.64 – 36.87; 12.09) |  | 27.96 (20.92 – 35.31; 10.86) |  | 44.12 (25.03 – 53.28; 18.88) |  | 29.77 (23.26 – 40.82; 12.72) |  | 26.2 (19.27 – 49.71; 12.69) |  | 30.58 (24.19 – 42.36; 12.31) |  | 27.63 (19.54 – 34.47; 11.98) |  | 32.32 (25.03 – 43.8; 13.83) |  | 31 (23.99 – 38.82; 13.51) |  |
| **% time COx_L > 0.3** | 19.67 (15.88 – 26.79; 7.04) | 0.839 | 17.6 (13.08 – 21.01; 6.58) | 0.5713 | 28 (16.18 – 41.73; 20.35) | 0.6866 | 19.74 (15.99 – 28.45; 9.13) | 0.688 | 19.43 (14.77 – 26.33; 9.45) | 0.9197 | 19.83 (15.9 – 28.61; 8.57) | 0.5902 | 18.49 (13.98 – 27.34; 9.65) | 0.8983 | 19.59 (16.14 – 30.7; 9.69) | 0.3952 | 22.84 (15.39 – 33.13; 13.13) | 0.9591 |
| **% time COx_R > 0.3** | 19.56 (13.23 – 27.59; 9.49) |  | 17.99 (13.86 – 23.62; 7.85) |  | 32.15 (16.17 – 43.85; 21.59) |  | 20.5 (15.12 – 29.85; 10.75) |  | 16.97 (13.16 – 37.05; 10.38) |  | 21.1 (15.62 – 32.65; 11.3) |  | 17.78 (12.24 – 25.28; 9.03) |  | 22.9 (15.97 – 33.2; 13.19) |  | 21.86 (16.43 – 30.45; 10.07) |  |
| **% time COx-a_L > 0** | 61.38 (54.25 – 65.03; 6.05) | 0.6198 | 59.05 (52.73 – 67.92; 12.59) | 0.6476 | 71.5 (53.4 – 75.83; 16.23) | 0.6139 | 61.04 (54.22 – 67.68; 10.08) | 0.6394 | 64.81 (53.13 – 67.21; 7.55) | 0.5788 | 59.23 (52.53 – 67.3; 11.4) | 0.9965 | 64.29 (59.39 – 68.02; 6.09) | 0.9352 | 59.62 (51.2 – 67.54; 12.15) | 0.805 | 60.54 (54.03 – 65.39; 9.15) | 0.5054 |
| **% time COx-a_R > 0** | 60.78 (52.7 – 64.26; 8.9) |  | 55.81 (53.46 – 68.12; 12.28) |  | 71.18 (54.93 – 79.36; 12.44) |  | 59.38 (53.09 – 69.08; 10.17) |  | 65.4 (53.82 – 71.63; 10.33) |  | 57.76 (53.03 – 67.11; 8.98) |  | 63.41 (54.13 – 70.91; 11.81) |  | 59.47 (53.73 – 66.84; 10.31) |  | 54.38 (52.74 – 64.28; 6.7) |  |
| **% time COx-a_L > 0.2** | 34.46 (32.53 – 38.9; 5.67) | 0.6547 | 34.71 (27.73 – 40.24; 10.03) | 0.9134 | 45.29 (28.42 – 57.82; 22.42) | 0.6866 | 34.55 (28.46 – 41.23; 9.55) | 0.8304 | 37.07 (28 – 44.04; 12.19) | 0.7623 | 33.48 (27.94 – 40.31; 8.87) | 0.7861 | 37.62 (33.44 – 43.97; 8.19) | 0.7893 | 33.57 (27.51 – 40.59; 9.34) | 0.4773 | 34.04 (29.52 – 42.53; 9.61) | 0.6454 |
| **% time COx-a_R > 0.2** | 33.84 (28.62 – 39.45; 7.76) |  | 30.99 (27.99 – 41.35; 13.8) |  | 47.21 (27.87 – 54.95; 21.62) |  | 33.04 (28.31 – 41.82; 10.21) |  | 40.32 (26.07 – 45.41; 15.65) |  | 32.85 (27.98 – 41.54; 9.98) |  | 38.88 (28.61 – 43.63; 12.18) |  | 34.39 (29.49 – 41.68; 10.01) |  | 30.32 (27.86 – 41.83; 7.33) |  |
| **% time COx-a_L > 0.3** | 23.89 (21.62 – 29.34; 4.55) | 0.9288 | 23.29 (16.79 – 28.32; 8.76) | 0.9134 | 31.15 (18.09 – 47.82; 24.7) | 0.7196 | 24.44 (19.42 – 29.99; 7.82) | 1 | 23.93 (21 – 31.15; 10.71) | 0.5788 | 23.39 (18.36 – 29.3; 8.57) | 0.7576 | 25.27 (22 – 31.33; 7.01) | 0.9537 | 21.67 (17.64 – 29.37; 11.16) | 0.5545 | 24.14 (19.64 – 31.44; 7.8) | 0.7984 |
| **% time COx-a_R > 0.3** | 23.46 (18.81 – 29.31; 8.21) |  | 20.99 (18.1 – 28.22; 9.03) |  | 34.54 (19.74 – 42.84; 21.59) |  | 22.74 (18.63 – 31.47; 9.05) |  | 26.2 (16.13 – 33.64; 14.92) |  | 22.81 (18.53 – 31.07; 8.21) |  | 26.26 (17.83 – 32.22; 10.74) |  | 22.97 (18.82 – 32.92; 10.14) |  | 21.36 (19.05 – 30.61; 6.53) |  |
| **5-Minute Data Resolution** | | | | | | | | | | | | | | | | | | |
| **ABP (mmHg)** | 84.04 (78.49 – 90.23) | – | 81.69 (74.86 – 91.15) | – | 79.16 (74.05 – 83.87) | – | 82.14 (76.02 – 89.77) | – | 82.28 (76.22 – 88.22) | – | 81.1 (76 – 89.09) | – | 83.34 (77.39 – 89.75) | – | 79.95 (74.77 – 88.22) | – | 87.02 (81.09 – 93.76) | – |
| **CPP (mmHg)** | 73.96 (67.3 – 80.26) |  | 69.91 (67.08 – 71.26) |  | 73.62 (69.47 – 75.72) |  | 71.96 (68.43 – 75.72) |  | 62.51 (56.66 – 66.36) |  | 67.97 (65.03 – 72.12) |  | 72.06 (67.77 – 76.41) |  | 67.85 (62.99 – 75.48) |  | 70.86 (68.61 – 74.76) |  |
| **rSO2_L (%)** | 68.18 (65.03 – 74.08) | 0.7145 | 68.34 (65.99 – 72.97) | 0.632 | 68.88 (63.13 – 71.05) | 0.6498 | 70.3 (65.99 – 74.98) | 0.8252 | 67.2 (63.41 – 71.02) | 0.3622 | 68.58 (63.99 – 72.29) | 0.679 | 71.15 (66.34 – 75.61) | 0.88 | 69.77 (66.19 – 72.97) | 0.4773 | 70.56 (65.62 – 73.69) | 0.8785 |
| **rSO2_R (%)** | 69.73 (64.8 – 75.45) |  | 73.32 (67.61 – 80.75) |  | 73.91 (67.73 – 81.3) |  | 73.93 (68.56 – 82.03) |  | 70.78 (65.33 – 76.66) |  | 73.73 (67.42 – 80.71) |  | 73.22 (67.98 – 80.4) |  | 73.32 (67.19 – 81.3) |  | 74.55 (68.67 – 81.52) |  |
| **COx_L (au)** | 0.03 (-0.15 – 0.21) | 0.7145 | 0.01 (-0.16 – 0.19) | 0.9134 | 0.12 (-0.04 – 0.29) | 0.8403 | 0.03 (-0.15 – 0.21) | 0.8671 | 0 (-0.19 – 0.19) | 0.7241 | 0.02 (-0.15 – 0.22) | 0.7998 | 0.03 (-0.15 – 0.19) | 0.7187 | 0.03 (-0.13 – 0.22) | 0.5783 | 0.03 (-0.16 – 0.24) | 0.8785 |
| **COx_R (au)** | 0.02 (-0.18 – 0.22) |  | 0 (-0.17 – 0.2) |  | 0.15 (-0.05 – 0.32) |  | 0.03 (-0.14 – 0.22) |  | 0.01 (-0.17 – 0.18) |  | 0.03 (-0.15 – 0.23) |  | 0.02 (-0.16 – 0.19) |  | 0.03 (-0.13 – 0.23) |  | 0.02 (-0.18 – 0.23) |  |
| **COx-a_L (au)** | 0.08 (-0.09 – 0.25) | 0.643 | 0.07 (-0.1 – 0.24) | 0.8109 | 0.17 (-0.01 – 0.31) | 0.7623 | 0.08 (-0.09 – 0.25) | 0.597 | 0.1 (-0.09 – 0.27) | 0.6866 | 0.08 (-0.1 – 0.25) | 0.9823 | 0.1 (-0.05 – 0.27) | 0.8073 | 0.08 (-0.1 – 0.25) | 0.7342 | 0.07 (-0.09 – 0.25) | 0.6454 |
| **COx-a_R (au)** | 0.08 (-0.1 – 0.25) |  | 0.05 (-0.13 – 0.21) |  | 0.18 (-0.01 – 0.35) |  | 0.07 (-0.12 – 0.24) |  | 0.12 (-0.07 – 0.28) |  | 0.06 (-0.12 – 0.24) |  | 0.11 (-0.07 – 0.28) |  | 0.07 (-0.12 – 0.25) |  | 0.04 (-0.13 – 0.21) |  |
| **MAD of ABP (mmHg)** | 8.22 (7.15 – 10.11) | – | 9.36 (7.87 – 11.13) | – | 8.19 (6.06 – 10.69) | – | 9.32 (7.03 – 11.01) | – | 7.44 (6.47 – 10.73) | – | 8.24 (6.35 – 11.01) | – | 9.34 (7.78 – 10.48) | – | 9.14 (6.06 – 11.27) | – | 9 (7.66 – 9.91) | – |
| **MAD of CPP (mmHg)** | 8.55 (6.96 – 11.53) |  | 4.52 (3.15 – 6.16) |  | 3.14 (2.65 – 6.03) |  | 4.72 (3.01 – 6.78) |  | 4.91 (3.17 – 9.72) |  | 3.81 (2.69 – 6.53) |  | 5.57 (4.51 – 8.18) |  | 4.07 (2.65 – 6.03) |  | 5.01 (2.84 – 8.81) |  |
| **MAD of rSO2_L (%)** | 5.57 (3.76 – 8.19) | 0.4891 | 4.33 (3.35 – 6.41) | 0.8618 | 3.8 (3.17 – 4.34) | 0.9598 | 4.42 (3.22 – 6.92) | 0.6684 | 5.71 (3.72 – 6.98) | 0.7623 | 4.12 (3.17 – 7.26) | 0.536 | 5.67 (4.5 – 6.7) | 0.88 | 3.82 (2.43 – 7.11) | 0.9754 | 5.77 (4.12 – 6.55) | 0.7209 |
| **MAD of rSO2_R (%)** | 6.05 (4.67 – 8.38) |  | 8.66 (7.24 – 11.17) |  | 6.34 (6.15 – 10.86) |  | 8.66 (6.44 – 11.7) |  | 8.03 (7.21 – 9.84) |  | 8.12 (6.26 – 11.87) |  | 9.04 (7.43 – 10.7) |  | 7.48 (6.15 – 12.18) |  | 8.93 (7.03 – 10.22) |  |
| **MAD of COx_L (au)** | 0.28 (0.24 – 0.32) | 0.9546 | 0.25 (0.23 – 0.3) | 0.2293 | 0.26 (0.21 – 0.28) | 0.5114 | 0.26 (0.24 – 0.31) | 0.1639 | 0.28 (0.24 – 0.31) | 0.5788 | 0.27 (0.25 – 0.31) | 0.4253 | 0.25 (0.24 – 0.3) | 0.4925 | 0.27 (0.25 – 0.31) | 0.3146 | 0.29 (0.24 – 0.31) | 0.8785 |
| **MAD of COx_R (au)** | 0.3 (0.25 – 0.31) |  | 0.27 (0.25 – 0.3) |  | 0.27 (0.24 – 0.29) |  | 0.29 (0.26 – 0.31) |  | 0.27 (0.23 – 0.3) |  | 0.28 (0.26 – 0.3) |  | 0.28 (0.24 – 0.31) |  | 0.28 (0.26 – 0.31) |  | 0.3 (0.26 – 0.31) |  |
| **MAD of COx-a_L (au)** | 0.26 (0.23 – 0.29) | 0.8773 | 0.24 (0.23 – 0.26) | 0.7114 | 0.25 (0.24 – 0.26) | 0.7241 | 0.25 (0.23 – 0.28) | 0.4104 | 0.25 (0.24 – 0.27) | 0.4793 | 0.25 (0.24 – 0.28) | 0.5419 | 0.24 (0.22 – 0.27) | 0.8983 | 0.25 (0.24 – 0.29) | 0.4397 | 0.26 (0.25 – 0.28) | 0.8785 |
| **MAD of COx-a_R (au)** | 0.27 (0.23 – 0.29) |  | 0.24 (0.23 – 0.26) |  | 0.26 (0.24 – 0.27) |  | 0.26 (0.24 – 0.28) |  | 0.24 (0.22 – 0.28) |  | 0.26 (0.24 – 0.28) |  | 0.24 (0.22 – 0.28) |  | 0.26 (0.24 – 0.29) |  | 0.25 (0.24 – 0.27) |  |
| **% time rSO2_L > 60%** | 88.72 (72.57 – 96.94; 16.61) | 0.4448 | 95.57 (53.26 – 98.62; 6.04) | 0.668 | 98.6 (52.05 – 99.77; 2.08) | 0.7954 | 95.57 (75.78 – 99.57; 6.56) | 0.8588 | 64.62 (39.52 – 85.95; 37.21) | 0.3041 | 94.05 (53.44 – 99.6; 8.82) | 0.431 | 91.87 (75.38 – 96.25; 10.06) | 0.7247 | 94.26 (39.52 – 99.77; 8.51) | 0.2251 | 91.8 (85.46 – 99.36; 11.35) | 0.8785 |
| **% time rSO2_R > 60%** | 93.21 (62.49 – 99.88; 10.07) |  | 94.31 (75.65 – 99.67; 8.18) |  | 92.99 (54.39 – 100; 10.39) |  | 93.98 (75.65 – 99.87; 8.92) |  | 94.01 (32.83 – 96.6; 8.88) |  | 94.16 (64.13 – 100; 8.66) |  | 93.21 (64.92 – 99.25; 9.78) |  | 97.79 (62.68 – 100; 3.27) |  | 92.66 (87.44 – 96.59; 7.15) |  |
| **% time rSO2_L > 70%** | 43.44 (9.39 – 77.92; 50.87) | 0.5228 | 49.4 (7.23 – 84.62; 61.29) | 0.5456 | 71.6 (0 – 95.27; 41.76) | 0.5189 | 66.33 (9.49 – 86.8; 46.3) | 0.968 | 9.66 (2.07 – 44.03; 14.32) | 0.6442 | 37.52 (3.8 – 79.83; 55.63) | 0.664 | 62.05 (20.69 – 83.94; 40.18) | 0.9537 | 32.2 (0.26 – 96.23; 47.74) | 0.3149 | 58.55 (43.73 – 80.15; 26.43) | 0.2786 |
| **% time rSO2_R > 70%** | 49.32 (32.7 – 72.73; 34.41) |  | 36.94 (10.69 – 93.88; 53.63) |  | 39.71 (2.35 – 71.45; 55.38) |  | 53.37 (21.46 – 84.9; 47.73) |  | 33.39 (0.84 – 45.06; 48.25) |  | 41.22 (8.66 – 81.85; 51.7) |  | 55.52 (33.93 – 72.81; 31.7) |  | 45.06 (15 – 91.62; 57.36) |  | 49.67 (26.68 – 69.73; 31.21) |  |
| **% time rSO2_L > 80%** | 2.03 (0.65 – 21.78; 3.02) | 0.6159 | 1.19 (0 – 21.77; 1.76) | 0.5024 | 11.09 (0 – 40.59; 16.45) | 0.1884 | 5.74 (0 – 29.41; 8.5) | 0.8553 | 0.39 (0 – 2.22; 0.58) | 1 | 1.38 (0 – 14.4; 2.05) | 0.9523 | 10.51 (0.48 – 27.72; 15.58) | 0.8232 | 1.56 (0 – 30.16; 2.31) | 0.9554 | 5.05 (0.89 – 29.5; 7.48) | 0.424 |
| **% time rSO2_R > 80%** | 6.67 (1.52 – 19.92; 9.89) |  | 5.03 (0.08 – 24.96; 7.46) |  | 0 (0 – 3.25; 0) |  | 5.98 (0.06 – 22.62; 8.87) |  | 0.25 (0 – 6.48; 0.37) |  | 1.53 (0 – 19.77; 2.26) |  | 6.67 (1.85 – 21.38; 9.71) |  | 5.98 (0 – 23.62; 8.87) |  | 2.6 (0 – 9.78; 3.85) |  |
| **% time rSO2_L > 90%** | 0 (0 – 1.18; 0) | 0.9774 | 0 (0 – 0; 0) | 0.4248 | 0 (0 – 0; 0) | 0.6019 | 0 (0 – 0.61; 0) | 0.8484 | 0 (0 – 0; 0) | 0.253 | 0 (0 – 0; 0) | 0.7317 | 0 (0 – 0.89; 0) | 0.9674 | 0 (0 – 0; 0) | 0.8359 | 0 (0 – 0.01; 0) | 1 |
| **% time rSO2_R > 90%** | 0 (0 – 0.63; 0) |  | 0 (0 – 0; 0) |  | 0 (0 – 0; 0) |  | 0 (0 – 0.3; 0) |  | 0 (0 – 0; 0) |  | 0 (0 – 0; 0) |  | 0 (0 – 0.87; 0) |  | 0 (0 – 0; 0) |  | 0 (0 – 0.13; 0) |  |
| **% time COx_L > 0** | 56.32 (49.77 – 60.17; 9.84) | 0.6665 | 51.85 (41.71 – 55.24; 14.11) | 1 | 67.57 (52.92 – 72.38; 13.88) | 0.898 | 54.97 (50.05 – 67.58; 11.2) | 0.5315 | 50.52 (42.88 – 63.18; 11.43) | 0.7623 | 54.56 (49.51 – 67.46; 15.01) | 0.9287 | 54.77 (47.52 – 58.71; 9.57) | 0.6672 | 54.97 (49.93 – 67.58; 12.83) | 0.8169 | 56.43 (48.48 – 68.1; 17.37) | 0.7525 |
| **% time COx_R > 0** | 52.89 (41.62 – 60.82; 16.09) |  | 49.45 (42.57 – 58.03; 13.4) |  | 67.51 (56.29 – 76.79; 16.63) |  | 54.07 (48.13 – 64.03; 13.84) |  | 51.37 (40.41 – 72.29; 18.34) |  | 54.07 (48.83 – 66.04; 14.66) |  | 52.74 (41.11 – 59.97; 16.22) |  | 55.45 (50.2 – 67.51; 12.14) |  | 52 (46.12 – 64.32; 18.32) |  |
| **% time COx_L > 0.2** | 27.13 (21.24 – 33.87; 9.35) | 0.7389 | 24.09 (15.9 – 28.19; 8.63) | 0.5566 | 36.12 (22.96 – 50; 20.58) | 0.5446 | 26.83 (20.5 – 37.39; 10.76) | 0.7786 | 24.09 (19.75 – 35.01; 11.61) | 1 | 27.53 (20.51 – 39.15; 13.39) | 0.5419 | 24.42 (19.84 – 32.97; 10.58) | 0.7361 | 27.43 (20.35 – 40.06; 15.09) | 0.3146 | 28.96 (21.95 – 42.21; 17.53) | 1 |
| **% time COx_R > 0.2** | 26.65 (18.16 – 34.47; 13.44) |  | 24.18 (16.86 – 33.01; 12.13) |  | 43.06 (20.25 – 50.6; 20.22) |  | 27.38 (19.79 – 39.45; 13.46) |  | 23.36 (17.28 – 47.84; 16.24) |  | 27.82 (20.3 – 41.78; 13.41) |  | 23.77 (14.22 – 31.55; 13.89) |  | 28.98 (21.05 – 43.06; 14.97) |  | 28.58 (20.47 – 37.16; 14.69) |  |
| **% time COx_L > 0.3** | 15.76 (12.85 – 22.53; 6.04) | 0.776 | 14.79 (9.06 – 17.41; 7.33) | 0.6367 | 23.88 (10.45 – 36.67; 19.92) | 0.6866 | 15.19 (11.4 – 24.11; 8.71) | 0.6954 | 16.06 (11.34 – 19.39; 7) | 1 | 15.29 (11.62 – 24.03; 8.06) | 0.5137 | 15.61 (9.82 – 23.37; 9.49) | 0.88 | 15.38 (12.15 – 27.42; 7.91) | 0.3881 | 18.01 (10.3 – 29.92; 12.97) | 0.9591 |
| **% time COx_R > 0.3** | 15.74 (10.12 – 23.85; 9.61) |  | 12.91 (10 – 20.04; 8.2) |  | 28.98 (12.88 – 39.88; 18.69) |  | 17.64 (11.2 – 26.14; 10.65) |  | 13.1 (9.55 – 32.68; 11.08) |  | 18.17 (11.99 – 29.71; 11.49) |  | 13.4 (8.16 – 21.06; 8.22) |  | 18.39 (13.35 – 30.18; 12.01) |  | 19.23 (11.98 – 25.83; 12.12) |  |
| **% time COx-a_L > 0** | 63.37 (55.8 – 67.34; 8.5) | 0.839 | 60.42 (53.69 – 70.69; 15.03) | 0.6166 | 71.98 (55.01 – 78.76; 20.93) | 0.6866 | 63.29 (54.72 – 70.69; 11.96) | 0.5249 | 65.69 (52.96 – 70.5; 9.32) | 0.3897 | 60.74 (53.86 – 69.63; 12.38) | 0.9397 | 66.44 (60.54 – 70.75; 7.54) | 0.8983 | 61.06 (52.96 – 69.75; 12.89) | 0.9385 | 62.63 (54.81 – 69.59; 11.48) | 0.8785 |
| **% time COx-a_R > 0** | 63.11 (54.27 – 66.45; 9.91) |  | 55.85 (53.76 – 70.42; 14.27) |  | 73.37 (54.6 – 83.28; 17.09) |  | 59.33 (54.28 – 72.06; 10.25) |  | 66.73 (53.53 – 73.37; 11.63) |  | 58.46 (54.27 – 70.23; 11.1) |  | 66.13 (54.78 – 74.55; 13.38) |  | 61.52 (54.29 – 68.99; 11.08) |  | 56.18 (54.26 – 67.85; 7.92) |  |
| **% time COx-a_L > 0.2** | 32.24 (29.52 – 37.59; 6.54) | 0.6701 | 30.3 (23.09 – 38.17; 12.34) | 0.9307 | 41.76 (24.53 – 56.99; 25.55) | 0.6139 | 31.25 (25.81 – 39.84; 9.97) | 0.7354 | 33.87 (22.28 – 41.76; 13.4) | 0.7623 | 30.91 (24.29 – 38.43; 10.85) | 0.8483 | 34.38 (29.91 – 42.85; 10.39) | 0.7537 | 31.25 (22.38 – 38.84; 13.15) | 0.4967 | 31.14 (27.42 – 40.75; 9.35) | 0.5737 |
| **% time COx-a_R > 0.2** | 31.15 (24.85 – 38.81; 10.63) |  | 26.58 (24.49 – 38.63; 13) |  | 46.6 (26.22 – 55.92; 25.21) |  | 29.59 (24.76 – 40.13; 11.13) |  | 37.43 (20.92 – 43.92; 17.78) |  | 29.11 (24.8 – 39.02; 10.68) |  | 36.55 (24.54 – 42.8; 13.97) |  | 31.05 (25.74 – 39.04; 11.69) |  | 26.49 (24.5 – 39.84; 7.41) |  |
| **% time COx-a_L > 0.3** | 19.39 (17.73 – 25; 5.36) | 0.7145 | 18.43 (14.15 – 23.33; 6.82) | 0.8347 | 26.37 (14.47 – 46.94; 26.41) | 0.6139 | 19.62 (15.59 – 26.38; 7.64) | 0.7863 | 20.83 (16.06 – 26.37; 8.22) | 0.801 | 19.14 (14.51 – 25.89; 7.54) | 1 | 22.05 (18.17 – 27.7; 6.82) | 0.9537 | 19.17 (14.47 – 26.37; 8.72) | 0.7931 | 19.8 (15.99 – 27.93; 8.72) | 0.8785 |
| **% time COx-a_R > 0.3** | 18.79 (14.88 – 26.01; 9.53) |  | 17.21 (13.9 – 24.45; 8.26) |  | 32.18 (15.95 – 40; 21.49) |  | 17.95 (14.85 – 27.92; 8.39) |  | 21.31 (10.56 – 30.58; 15.94) |  | 17.82 (14.9 – 27.26; 8.43) |  | 22.09 (14.35 – 28.94; 11.18) |  | 18.75 (15.41 – 28.24; 10.05) |  | 17.52 (14.12 – 27.16; 7.85) |  |
| *The p-values in the table are derived using Mann-Whitney U test between the bilateral signals.*  *ABP, arterial blood pressure; au, arbitrary units; CPP, cerebral perfusion pressure; COx, cerebral oximetry index with CPP; COx-a, cerebral oximetry index with ABP; CT, computed tomography; DAI, diffuse axonal injury; EDH, epidural hematoma; MAD, median absolute deviation; IQR, interquartile range; mmHg, millimeters of mercury; rSO2, regional cerebral oxygen saturation; tSAH, traumatic subarachnoid hemorrhage; SDH, subdural hematoma; aSDH, acute subdural hematoma; TBI-GLR, traumatic brain injury patient group without bifrontal lobe pathology.* | | | | | | | | | | | | | | | | | | |

| **Physiologic Variable** | **Sub-groups** | | | | | | | | | | | | | | | |
| --- | --- | --- | --- | --- | --- | --- | --- | --- | --- | --- | --- | --- | --- | --- | --- | --- |
|  | **Marshall CT III [n = 25]** | | **Marshall CT II [n = 2]** | | **Rotterdam CT 6 [n = 14]** | | **Rotterdam CT 5 [n = 16]** | | **Rotterdam CT 4 [n = 17]** | | **Rotterdam CT 3 [n = 13]** | | **Rotterdam CT 2 [n = 3]** | | **Rotterdam CT 1 [n = 1]** | |
|  | **Median (IQR) or  Median (IQR; MAD)** | **p-value** | **Median (IQR) or  Median (IQR; MAD)** | **p-value** | **Median (IQR) or  Median (IQR; MAD)** | **p-value** | **Median (IQR) or  Median (IQR; MAD)** | **p-value** | **Median (IQR) or  Median (IQR; MAD)** | **p-value** | **Median (IQR) or  Median (IQR; MAD)** | **p-value** | **Median (IQR) or  Median (IQR; MAD)** | **p-value** | **Median (IQR) or  Median (IQR; MAD)** | **p-value** |
| **10-Second Data Resolution** | | | | | | | | | | | | | | | | |
| **ABP (mmHg)** | 82.08 (76.89 – 89.41) | – | 87.29 (78.65 – 97.58) | – | 80.38 (74.46 – 88.79) | – | 82.04 (76.53 – 89.41) | – | 83.26 (75.59 – 89.81) | – | 82.02 (77.08 – 89.27) | – | 84.2 (74.53 – 99.84) | – | 76.47 (70.31 – 84.25) | – |
| **CPP (mmHg)** | 72.76 (67.09 – 80) |  | 79.84 (71.11 – 90.36) |  | 72.95 (66.79 – 80.98) |  | 74.5 (67.87 – 82.27) |  | 73.4 (67.09 – 81.63) |  | 72.76 (67.73 – 78.77) |  | 74.58 (62.54 – 91.4) |  | 70.39 (64.42 – 77.97) |  |
| **rSO2_L (%)** | 69.97 (65.99 – 74) | 0.9768 | 67.53 (61.25 – 71.39) | 0.6667 | 66.99 (64.93 – 72.51) | 0.7131 | 68 (63.72 – 74.37) | 0.559 | 70.99 (67.99 – 74.63) | 0.2702 | 71.33 (68.5 – 75.99) | 1 | 62.07 (55.5 – 65.99) | 1 | 67.99 (63.99 – 70.99) | 1 |
| **rSO2_R (%)** | 68.11 (63.99 – 74.99) |  | 64.49 (57.25 – 68.75) |  | 66.51 (62.99 – 73.68) |  | 71.45 (68 – 73.5) |  | 67.99 (61.99 – 73.8) |  | 69 (65 – 74.5) |  | 71.99 (67.99 – 76.99) |  | 68.11 (65.99 – 71.99) |  |
| **COx_L (au)** | 0 (-0.19 – 0.21) | 0.4027 | -0.01 (-0.26 – 0.27) | 0.6667 | 0 (-0.12 – 0.22) | 0.3759 | 0 (-0.14 – 0.28) | 0.6258 | 0.01 (-0.18 – 0.25) | 0.7043 | 0 (-0.19 – 0.22) | 1 | -0.07 (-0.33 – 0.17) | 1 | 0.04 (-0.2 – 0.26) | 1 |
| **COx_R (au)** | 0 (-0.19 – 0.22) |  | 0.05 (-0.21 – 0.32) |  | 0 (-0.13 – 0.24) |  | 0.01 (-0.13 – 0.28) |  | 0 (-0.19 – 0.23) |  | 0.01 (-0.15 – 0.23) |  | -0.07 (-0.29 – 0.15) |  | -0.01 (-0.25 – 0.18) |  |
| **COx-a_L (au)** | 0.08 (-0.08 – 0.29) | 0.6412 | 0.03 (-0.2 – 0.29) | 0.6667 | 0 (-0.1 – 0.24) | 0.8335 | 0.03 (-0.1 – 0.28) | 0.4389 | 0.09 (-0.08 – 0.29) | 0.3794 | 0.06 (-0.09 – 0.29) | 0.6629 | 0.04 (-0.18 – 0.23) | 1 | 0.08 (-0.16 – 0.3) | 1 |
| **COx-a_R (au)** | 0.07 (-0.08 – 0.3) |  | 0.08 (-0.16 – 0.35) |  | 0 (-0.12 – 0.26) |  | 0.07 (-0.11 – 0.3) |  | 0.03 (-0.14 – 0.3) |  | 0.08 (-0.08 – 0.3) |  | 0.06 (-0.18 – 0.27) |  | 0.02 (-0.2 – 0.24) |  |
| **MAD of ABP (mmHg)** | 8.81 (7.86 – 9.84) | – | 13.43 (12.48 – 14.38) | – | 9.64 (7.63 – 11.05) | – | 7.42 (6.41 – 10.91) | – | 9.4 (8.12 – 11.58) | – | 8.58 (7.69 – 9.84) | – | 15.33 (10.45 – 24.05) | – | 10.22 (10.22 – 10.22) | – |
| **MAD of CPP (mmHg)** | 8.73 (8.05 – 10.6) |  | 13.8 (13.22 – 14.38) |  | 8.09 (6.45 – 11.31) |  | 9.3 (6.37 – 12.33) |  | 8.73 (8.15 – 11.34) |  | 8.35 (7.21 – 10.6) |  | 14.96 (10.32 – 24.77) |  | 9.89 (9.89 – 9.89) |  |
| **MAD of rSO2_L (%)** | 5.22 (3.99 – 7.24) | 0.9693 | 7.44 (6.68 – 8.19) | 1 | 3.67 (2.92 – 5.93) | 0.9268 | 4.64 (2.88 – 6.51) | 0.6109 | 5.71 (3.17 – 7.41) | 0.7435 | 5.23 (4.09 – 7.13) | 0.2281 | 6.97 (5.4 – 7.95) | 0.7 | 5.22 (5.22 – 5.22) | 1 |
| **MAD of rSO2_R (%)** | 5.49 (4.27 – 6.66) |  | 8.03 (6.98 – 9.08) |  | 4.05 (2.97 – 7.04) |  | 5.19 (3.73 – 7.66) |  | 5.49 (4.45 – 8.18) |  | 4.27 (2.98 – 5.65) |  | 7.21 (6.83 – 8.67) |  | 4.49 (4.49 – 4.49) |  |
| **MAD of COx_L (au)** | 0.31 (0.27 – 0.32) | 0.4788 | 0.39 (0.38 – 0.39) | 1 | 0.3 (0.24 – 0.35) | 0.8743 | 0.33 (0.27 – 0.38) | 0.5147 | 0.31 (0.29 – 0.32) | 1 | 0.31 (0.26 – 0.36) | 0.6866 | 0.3 (0.29 – 0.34) | 0.7 | 0.35 (0.35 – 0.35) | 1 |
| **MAD of COx_R (au)** | 0.31 (0.28 – 0.35) |  | 0.38 (0.38 – 0.39) |  | 0.33 (0.24 – 0.34) |  | 0.34 (0.3 – 0.38) |  | 0.31 (0.26 – 0.32) |  | 0.34 (0.28 – 0.36) |  | 0.36 (0.32 – 0.37) |  | 0.32 (0.32 – 0.32) |  |
| **MAD of COx-a_L (au)** | 0.29 (0.26 – 0.31) | 0.5768 | 0.36 (0.35 – 0.37) | 1 | 0.28 (0.24 – 0.3) | 0.6294 | 0.28 (0.25 – 0.36) | 0.4016 | 0.3 (0.27 – 0.32) | 0.9729 | 0.29 (0.24 – 0.32) | 0.6866 | 0.3 (0.28 – 0.32) | 1 | 0.34 (0.34 – 0.34) | 1 |
| **MAD of COx-a_R (au)** | 0.29 (0.26 – 0.32) |  | 0.37 (0.35 – 0.39) |  | 0.3 (0.25 – 0.34) |  | 0.31 (0.28 – 0.35) |  | 0.3 (0.27 – 0.31) |  | 0.29 (0.26 – 0.31) |  | 0.33 (0.3 – 0.34) |  | 0.32 (0.32 – 0.32) |  |
| **% time rSO2_L > 60%** | 92.79 (57.65 – 96.99; 10.3) | 0.8626 | 74 (65.38 – 82.62; 25.56) | 1 | 93.1 (37.62 – 99.69; 10.23) | 0.7648 | 89.37 (65.64 – 98.8; 15.74) | 0.1568 | 95.57 (63.81 – 99.68; 6.46) | 0.3435 | 91.24 (81.59 – 96.24; 12.02) | 0.7388 | 56.76 (43.35 – 76.63; 39.78) | 1 | 92.79 (92.79 – 92.79; 0) | 1 |
| **% time rSO2_R > 60%** | 92.95 (55.09 – 98.14; 10.34) |  | 65.75 (52.52 – 78.99; 39.25) |  | 95.87 (45.93 – 99.88; 6.13) |  | 94.02 (91.08 – 99.96; 8.78) |  | 80.62 (55.09 – 98.14; 28.26) |  | 93.9 (80.7 – 99.82; 8.93) |  | 92.95 (66.11 – 94.72; 5.25) |  | 89.87 (89.87 – 89.87; 0) |  |
| **% time rSO2_L > 70%** | 48.48 (8.59 – 76.54; 52.79) | 0.9613 | 39.8 (25.44 – 54.16; 42.58) | 0.6667 | 28.64 (2.55 – 88.89; 42.46) | 0.6788 | 42.35 (2.93 – 87.23; 61.78) | 0.6689 | 58.29 (19.94 – 74.17; 42.06) | 0.3096 | 67.69 (8.59 – 76.44; 34.12) | 1 | 11.08 (5.9 – 50.78; 15.36) | 0.7 | 29.02 (29.02 – 29.02; 0) | 1 |
| **% time rSO2_R > 70%** | 39.05 (16.41 – 69.66; 36.56) |  | 29.69 (17.57 – 41.82; 35.95) |  | 31.88 (6.11 – 94.49; 47.2) |  | 63.43 (34.6 – 73.5; 31.31) |  | 38.58 (14.39 – 60.69; 35.86) |  | 36.35 (16.41 – 91.67; 52.51) |  | 62.78 (34.11 – 79.48; 49.53) |  | 32.88 (32.88 – 32.88; 0) |  |
| **% time rSO2_L > 80%** | 1.82 (0.25 – 20.93; 2.69) | 0.6974 | 5.54 (2.78 – 8.3; 8.17) | 1 | 1.23 (0 – 11.5; 1.82) | 0.7943 | 4.77 (0 – 38.66; 7.08) | 0.7741 | 1.82 (0.48 – 20.75; 2.69) | 0.5799 | 9.3 (0.73 – 25.1; 13.79) | 0.8369 | 0.03 (0.02 – 16.46; 0.03) | 0.4 | 0.25 (0.25 – 0.25; 0) | 1 |
| **% time rSO2_R > 80%** | 4.02 (0.57 – 20.05; 5.97) |  | 1.16 (0.69 – 1.64; 1.41) |  | 3.46 (0 – 34.27; 5.14) |  | 5.83 (0.03 – 20.29; 8.64) |  | 2.4 (0.06 – 7.99; 3.56) |  | 2.12 (0.96 – 21.62; 3.14) |  | 9.27 (4.74 – 37.76; 13.42) |  | 0.57 (0.57 – 0.57; 0) |  |
| **% time rSO2_L > 90%** | 0.01 (0 – 0.06; 0.02) | 0.5102 | 0 (0 – 0; 0) | 0.6171 | 0 (0 – 0.25; 0) | 0.4815 | 0 (0 – 1.4; 0) | 0.6873 | 0 (0 – 0.02; 0) | 0.9396 | 0 (0 – 0.21; 0.01) | 1 | 0 (0 – 0.02; 0) | 0.3537 | 0 (0 – 0; 0) | 1 |
| **% time rSO2_R > 90%** | 0.02 (0 – 1.05; 0.02) |  | 0.05 (0.03 – 0.08; 0.08) |  | 0.01 (0 – 0.19; 0.02) |  | 0 (0 – 0.69; 0) |  | 0 (0 – 0.06; 0) |  | 0.01 (0 – 0.1; 0.02) |  | 0.1 (0.05 – 12.4; 0.15) |  | 0 (0 – 0; 0) |  |
| **% time COx_L > 0** | 50.08 (42.84 – 54.84; 8.26) | 0.5004 | 48.65 (45.35 – 51.95; 9.8) | 0.6667 | 46.25 (41.26 – 50.91; 7.85) | 0.5112 | 50.2 (49.18 – 55.93; 4.22) | 0.9852 | 50.91 (43.91 – 64.54; 12.63) | 0.5177 | 47.73 (42.84 – 52.4; 7.25) | 0.6866 | 42.04 (35.95 – 45.92; 11.5) | 1 | 54.64 (54.64 – 54.64; 0) | 1 |
| **% time COx_R > 0** | 46.82 (40.46 – 55.7; 9.97) |  | 55.25 (52.39 – 58.11; 8.49) |  | 48.48 (44.67 – 56.48; 9.7) |  | 51.36 (47.23 – 59.34; 8.83) |  | 50.58 (38.59 – 56.67; 15.54) |  | 50.75 (43.11 – 55.7; 11.33) |  | 41.94 (38.95 – 45.73; 8.87) |  | 45.66 (45.66 – 45.66; 0) |  |
| **% time COx_L > 0.2** | 26.34 (23.35 – 31.78; 7.31) | 0.6581 | 29.74 (26.06 – 33.43; 10.93) | 0.6667 | 26.79 (24.11 – 29.17; 4.13) | 0.7345 | 30.78 (26.23 – 36.47; 7.83) | 0.5896 | 29.61 (22.68 – 39.46; 11.37) | 0.6832 | 26.81 (23.35 – 30.98; 6.17) | 0.8403 | 22.37 (17.54 – 23.55; 3.51) | 1 | 31.27 (31.27 – 31.27; 0) | 1 |
| **% time COx_R > 0.2** | 26.7 (18.67 – 31.6; 11.88) |  | 34.24 (31 – 37.47; 9.58) |  | 28.14 (21.86 – 38.33; 10.19) |  | 32.06 (25.3 – 41.09; 11.78) |  | 27.5 (18.67 – 36.13; 12.92) |  | 28.23 (20.09 – 32.85; 12.07) |  | 20.4 (18.23 – 24.09; 6.43) |  | 23.26 (23.26 – 23.26; 0) |  |
| **% time COx_L > 0.3** | 17.95 (14.53 – 24.06; 6.02) | 0.6581 | 21.71 (17.92 – 25.51; 11.25) | 0.6667 | 18.11 (16.79 – 20.66; 3.63) | 0.6027 | 23.33 (17.45 – 28; 8.13) | 0.6156 | 20.69 (14.09 – 27.18; 9.79) | 0.6584 | 17.95 (15.9 – 22.01; 6.02) | 0.8798 | 14.12 (11.11 – 14.33; 0.61) | 1 | 21.73 (21.73 – 21.73; 0) | 1 |
| **% time COx_R > 0.3** | 17.58 (12.27 – 22.69; 7.87) |  | 25.77 (22.27 – 29.27; 10.38) |  | 20.69 (14.9 – 30.66; 10.22) |  | 23.45 (16.95 – 32.86; 11.94) |  | 19.25 (12.27 – 25.83; 10.34) |  | 17.81 (13.78 – 23.72; 6.6) |  | 13.7 (12 – 16.23; 5.02) |  | 15.1 (15.1 – 15.1; 0) |  |
| **% time COx-a_L > 0** | 60.32 (53.05 – 66.46; 10.78) | 0.8626 | 53.36 (50.97 – 55.75; 7.09) | 0.6667 | 49.93 (45.85 – 59.61; 10.61) | 0.982 | 52.79 (48.74 – 60.04; 8.74) | 0.5391 | 61.7 (53.51 – 67.75; 12.14) | 0.2897 | 58.15 (51.07 – 64.72; 10.5) | 0.5788 | 54.46 (51.52 – 60.21; 8.72) | 1 | 58.6 (58.6 – 58.6; 0) | 1 |
| **% time COx-a_R > 0** | 57.4 (49.97 – 70.13; 15.17) |  | 59.1 (58.12 – 60.09; 2.92) |  | 50.39 (44.44 – 58.97; 10.2) |  | 57.4 (51.03 – 61.36; 8.99) |  | 54.22 (49.99 – 68.42; 13.77) |  | 59.37 (54.53 – 66.75; 9.91) |  | 57.13 (52.02 – 63.9; 15.15) |  | 51.73 (51.73 – 51.73; 0) |  |
| **% time COx-a_L > 0.2** | 34.57 (29 – 41.92; 9.36) | 0.7292 | 31.83 (28.61 – 35.06; 9.57) | 0.6667 | 28.66 (26.42 – 37.24; 4.33) | 0.7006 | 32.72 (27.58 – 36.93; 6.67) | 0.2703 | 34.72 (30.95 – 45.18; 13.8) | 0.4332 | 33.74 (27.5 – 38.29; 9.25) | 0.7623 | 28.25 (26.81 – 32.99; 4.25) | 1 | 35.18 (35.18 – 35.18; 0) | 1 |
| **% time COx-a_R > 0.2** | 36.29 (27.81 – 42.23; 12.57) |  | 37.48 (35.3 – 39.67; 6.48) |  | 30.11 (23.75 – 39.17; 11.92) |  | 35.65 (30.37 – 39.1; 7.62) |  | 33.28 (26.87 – 42.23; 13.27) |  | 36.77 (28.74 – 41.86; 11.9) |  | 33.11 (28.47 – 37.49; 12.98) |  | 28.37 (28.37 – 28.37; 0) |  |
| **% time COx-a_L > 0.3** | 24.06 (18.59 – 28.42; 8.12) | 0.9234 | 23.3 (19.82 – 26.78; 10.33) | 0.6667 | 20.14 (16.61 – 25.21; 6.16) | 0.8036 | 23.4 (19.4 – 27.83; 6.58) | 0.423 | 24.04 (21.27 – 32.84; 10.62) | 0.6339 | 24.06 (18.45 – 30.27; 9.04) | 0.6498 | 17.24 (16.79 – 20.61; 1.34) | 1 | 25.11 (25.11 – 25.11; 0) | 1 |
| **% time COx-a_R > 0.3** | 24.5 (17.58 – 30.29; 10.25) |  | 27.99 (25.09 – 30.9; 8.61) |  | 21.16 (15.9 – 31.65; 9.8) |  | 25.41 (19.74 – 30.94; 8.45) |  | 24.62 (17.07 – 30.29; 11.19) |  | 24.5 (19.36 – 32.51; 7.95) |  | 22.18 (18.67 – 24.68; 7.42) |  | 19.34 (19.34 – 19.34; 0) |  |
| **1-Minute Data Resolution** | | | | | | | | | | | | | | | | |
| **ABP (mmHg)** | 82.18 (76.92 – 89.31) | – | 87.63 (78.86 – 98.14) | – | 80.48 (74.56 – 88.76) | – | 82.17 (76.56 – 89.33) | – | 83.34 (75.66 – 89.84) | – | 82.02 (77.19 – 89.25) | – | 84.41 (74.7 – 99.99) | – | 76.7 (70.37 – 84.19) | – |
| **CPP (mmHg)** | 72.77 (67.41 – 80.09) |  | 80.27 (71.51 – 91.02) |  | 73.07 (66.83 – 80.9) |  | 74.51 (67.92 – 82.09) |  | 73.55 (67.41 – 81.73) |  | 72.77 (68.21 – 78.52) |  | 74.39 (62.66 – 91.68) |  | 70.65 (64.52 – 77.89) |  |
| **rSO2_L (%)** | 69.83 (65.87 – 73.95) | 0.9387 | 67.65 (61.2 – 71.36) | 0.6667 | 67.02 (64.99 – 72.7) | 0.7131 | 68 (63.75 – 74.29) | 0.5896 | 71.1 (68.13 – 74.58) | 0.2743 | 71.34 (68.5 – 76.15) | 0.9598 | 62.32 (55.58 – 66.14) | 0.7 | 68.24 (63.83 – 70.83) | 1 |
| **rSO2_R (%)** | 68.32 (64.2 – 75) |  | 64.46 (57.41 – 68.73) |  | 66.6 (63.07 – 73.74) |  | 71.56 (67.95 – 73.57) |  | 68.09 (61.99 – 73.75) |  | 68.78 (65.1 – 74.29) |  | 71.82 (68.15 – 76.83) |  | 68.32 (65.99 – 72.28) |  |
| **COx_L (au)** | 0.02 (-0.19 – 0.21) | 0.5253 | -0.01 (-0.26 – 0.26) | 0.6667 | 0.02 (-0.17 – 0.23) | 0.9459 | 0.04 (-0.15 – 0.28) | 0.423 | 0.03 (-0.17 – 0.24) | 0.6339 | 0.02 (-0.18 – 0.21) | 0.9598 | -0.08 (-0.33 – 0.16) | 0.8248 | 0.04 (-0.19 – 0.26) | 1 |
| **COx_R (au)** | 0.01 (-0.21 – 0.21) |  | 0.05 (-0.2 – 0.31) |  | 0.02 (-0.18 – 0.25) |  | 0.06 (-0.16 – 0.29) |  | 0.01 (-0.21 – 0.22) |  | 0.03 (-0.15 – 0.22) |  | -0.07 (-0.29 – 0.14) |  | -0.02 (-0.25 – 0.19) |  |
| **COx-a_L (au)** | 0.1 (-0.09 – 0.29) | 0.6169 | 0.04 (-0.2 – 0.28) | 0.6667 | 0.06 (-0.14 – 0.25) | 0.8743 | 0.08 (-0.12 – 0.29) | 0.5147 | 0.1 (-0.1 – 0.31) | 0.5629 | 0.09 (-0.09 – 0.29) | 1 | 0.04 (-0.17 – 0.22) | 1 | 0.08 (-0.16 – 0.29) | 1 |
| **COx-a_R (au)** | 0.1 (-0.09 – 0.29) |  | 0.08 (-0.15 – 0.34) |  | 0.03 (-0.15 – 0.25) |  | 0.09 (-0.12 – 0.3) |  | 0.1 (-0.14 – 0.3) |  | 0.11 (-0.09 – 0.29) |  | 0.06 (-0.18 – 0.26) |  | 0.03 (-0.2 – 0.24) |  |
| **MAD of ABP (mmHg)** | 8.51 (7.56 – 9.84) | – | 13.59 (12.63 – 14.55) | – | 9.5 (7.61 – 11.13) | – | 7.25 (6.05 – 10.69) | – | 9.14 (7.95 – 11.38) | – | 8.46 (7.45 – 9.41) | – | 15.52 (10.53 – 24.02) | – | 10.02 (10.02 – 10.02) | – |
| **MAD of CPP (mmHg)** | 8.44 (7.67 – 10.39) |  | 13.92 (13.38 – 14.46) |  | 7.87 (6.38 – 11.31) |  | 9.18 (6.3 – 11.91) |  | 8.55 (7.79 – 11.11) |  | 8 (7.03 – 10.39) |  | 15 (10.24 – 25.11) |  | 9.83 (9.83 – 9.83) |  |
| **MAD of rSO2_L (%)** | 5.2 (3.93 – 6.93) | 0.8475 | 7.36 (6.61 – 8.12) | 1 | 3.73 (2.72 – 5.86) | 0.91 | 4.54 (2.76 – 6.56) | 0.7804 | 5.66 (3.1 – 7.29) | 0.7084 | 5.2 (4.19 – 6.83) | 0.2226 | 6.87 (5.35 – 7.87) | 0.7 | 5.19 (5.19 – 5.19) | 1 |
| **MAD of rSO2_R (%)** | 5.29 (4.36 – 6.56) |  | 7.82 (6.76 – 8.87) |  | 3.87 (3.12 – 6.91) |  | 5.17 (3.56 – 7.95) |  | 5.45 (4.26 – 8.32) |  | 4.47 (3.21 – 5.57) |  | 7 (6.77 – 8.47) |  | 4.49 (4.49 – 4.49) |  |
| **MAD of COx_L (au)** | 0.3 (0.28 – 0.34) | 0.6581 | 0.38 (0.37 – 0.38) | 0.6667 | 0.32 (0.3 – 0.37) | 0.91 | 0.35 (0.29 – 0.39) | 0.3045 | 0.3 (0.28 – 0.31) | 0.4332 | 0.32 (0.29 – 0.35) | 0.9197 | 0.29 (0.28 – 0.33) | 1 | 0.33 (0.33 – 0.33) | 1 |
| **MAD of COx_R (au)** | 0.32 (0.28 – 0.35) |  | 0.37 (0.37 – 0.37) |  | 0.33 (0.31 – 0.35) |  | 0.36 (0.31 – 0.4) |  | 0.31 (0.28 – 0.34) |  | 0.33 (0.29 – 0.35) |  | 0.35 (0.32 – 0.36) |  | 0.32 (0.32 – 0.32) |  |
| **MAD of COx-a_L (au)** | 0.28 (0.27 – 0.31) | 0.8325 | 0.35 (0.34 – 0.36) | 1 | 0.29 (0.28 – 0.36) | 0.7006 | 0.31 (0.28 – 0.36) | 0.5147 | 0.29 (0.27 – 0.31) | 0.4958 | 0.28 (0.27 – 0.31) | 0.8403 | 0.29 (0.27 – 0.31) | 1 | 0.34 (0.34 – 0.34) | 1 |
| **MAD of COx-a_R (au)** | 0.29 (0.27 – 0.32) |  | 0.36 (0.34 – 0.38) |  | 0.3 (0.28 – 0.35) |  | 0.33 (0.3 – 0.35) |  | 0.3 (0.29 – 0.32) |  | 0.29 (0.27 – 0.3) |  | 0.33 (0.29 – 0.33) |  | 0.32 (0.32 – 0.32) |  |
| **% time rSO2_L > 60%** | 93.17 (58.36 – 96.93; 10.08) | 0.8843 | 74.39 (65.91 – 82.87; 25.15) | 1 | 93.61 (37.87 – 99.74; 9.48) | 0.6284 | 89.57 (66.86 – 98.77; 15.46) | 0.125 | 95.66 (64.34 – 99.66; 6.43) | 0.3175 | 92.13 (81.6 – 96.12; 10.87) | 0.7388 | 57.43 (43.94 – 76.95; 40.01) | 0.8248 | 93.17 (93.17 – 93.17; 0) | 1 |
| **% time rSO2_R > 60%** | 93.43 (55.51 – 98.04; 9.61) |  | 66.31 (53.19 – 79.44; 38.92) |  | 96.51 (45.75 – 99.87; 5.17) |  | 94.59 (91.14 – 100; 7.97) |  | 80.93 (55.51 – 98.04; 28.11) |  | 94.33 (80.62 – 99.78; 8.28) |  | 93.43 (66.75 – 94.95; 4.5) |  | 90.19 (90.19 – 90.19; 0) |  |
| **% time rSO2_L > 70%** | 48.97 (9.01 – 78.96; 53.95) | 0.9613 | 40.73 (26.23 – 55.23; 42.99) | 0.6667 | 29.27 (2.61 – 89.77; 43.39) | 0.6127 | 43.07 (3.06 – 87.52; 61.71) | 0.6923 | 60.05 (21.08 – 77.38; 40.84) | 0.2934 | 69.73 (9.01 – 77.84; 31.6) | 1 | 11.73 (6.2 – 51.8; 16.4) | 0.7 | 30.57 (30.57 – 30.57; 0) | 1 |
| **% time rSO2_R > 70%** | 40.48 (17.27 – 70.62; 37.98) |  | 30.6 (18.13 – 43.07; 36.97) |  | 32.42 (6.34 – 95.36; 48) |  | 65.39 (35.64 – 74.09; 29.82) |  | 40.48 (14.86 – 61.81; 37.98) |  | 37.08 (17.27 – 92.06; 53.53) |  | 64.21 (34.94 – 80.2; 47.41) |  | 33.75 (33.75 – 33.75; 0) |  |
| **% time rSO2_L > 80%** | 2.12 (0.25 – 21.53; 3.14) | 0.6971 | 5.62 (2.85 – 8.39; 8.21) | 1 | 1.22 (0 – 12.17; 1.81) | 0.7943 | 4.93 (0 – 41.01; 7.31) | 0.6582 | 2.12 (0.49 – 21.02; 3.14) | 0.5799 | 10.03 (0.78 – 26.69; 14.87) | 0.8369 | 0.09 (0.04 – 16.94; 0.13) | 0.4 | 0.25 (0.25 – 0.25; 0) | 1 |
| **% time rSO2_R > 80%** | 4.26 (0.74 – 20.6; 6.31) |  | 1.21 (0.71 – 1.71; 1.47) |  | 3.7 (0 – 35.86; 5.48) |  | 6.23 (0 – 20.86; 9.24) |  | 2.5 (0.07 – 8.44; 3.71) |  | 2.2 (1 – 22.82; 3.27) |  | 9.95 (5.09 – 38.19; 14.43) |  | 0.74 (0.74 – 0.74; 0) |  |
| **% time rSO2_L > 90%** | 0.01 (0 – 0.08; 0.02) | 0.4557 | 0 (0 – 0; 0) | 0.6171 | 0 (0 – 0.31; 0) | 0.8463 | 0 (0 – 1.47; 0) | 0.7187 | 0 (0 – 0.02; 0) | 0.847 | 0 (0 – 0.15; 0) | 0.9333 | 0 (0 – 0.02; 0) | 0.3537 | 0 (0 – 0; 0) | – |
| **% time rSO2_R > 90%** | 0.02 (0 – 1.19; 0.04) |  | 0.04 (0.02 – 0.07; 0.06) |  | 0 (0 – 0.2; 0) |  | 0 (0 – 0.7; 0) |  | 0 (0 – 0.07; 0) |  | 0 (0 – 0.03; 0) |  | 0.09 (0.04 – 12.92; 0.13) |  | 0 (0 – 0; 0) |  |
| **% time COx_L > 0** | 52.18 (47.78 – 57.57; 7.98) | 0.4882 | 48.65 (45.03 – 52.27; 10.73) | 0.6667 | 52.55 (48.66 – 63.9; 11.33) | 0.982 | 55.47 (50.67 – 64.69; 8.43) | 0.5147 | 54.12 (47.78 – 65.32; 15.02) | 0.5018 | 52.18 (46.04 – 57.57; 9.12) | 0.9598 | 41.41 (35.58 – 45.41; 11.84) | 1 | 55.19 (55.19 – 55.19; 0) | 1 |
| **% time COx_R > 0** | 51.37 (42.54 – 57.13; 12.55) |  | 55.07 (52.11 – 58.04; 8.79) |  | 52.53 (49.59 – 68.78; 8.41) |  | 56.45 (52.66 – 66; 8.38) |  | 51.37 (42.54 – 57.54; 13.08) |  | 56.01 (44.69 – 61; 12.94) |  | 41.55 (38.85 – 45.35; 8.02) |  | 47.65 (47.65 – 47.65; 0) |  |
| **% time COx_L > 0.2** | 26.62 (24.07 – 34.45; 6.32) | 0.5768 | 29.57 (25.82 – 33.32; 11.12) | 0.6667 | 27.1 (25.53 – 38.51; 7.15) | 0.8388 | 32.41 (27.5 – 41.18; 9.78) | 0.4909 | 29.55 (25.36 – 38.98; 12.33) | 0.8119 | 26.62 (23.19 – 37.07; 5.88) | 0.9197 | 22.08 (17.08 – 23.09; 3) | 1 | 30.89 (30.89 – 30.89; 0) | 1 |
| **% time COx_R > 0.2** | 26.2 (19.27 – 31.65; 10.27) |  | 33.99 (30.67 – 37.32; 9.85) |  | 29.2 (22.87 – 45.7; 11.14) |  | 33.74 (30.16 – 43.88; 9.88) |  | 27.29 (19.46 – 35.94; 12.82) |  | 27.96 (20.93 – 38.8; 12.88) |  | 19.77 (17.63 – 23.56; 6.35) |  | 23.52 (23.52 – 23.52; 0) |  |
| **% time COx_L > 0.3** | 17.39 (15.17 – 23.89; 7.48) | 0.5768 | 20.94 (17.05 – 24.84; 11.54) | 0.6667 | 18.17 (15.67 – 27.95; 6.35) | 0.7345 | 23.64 (18.08 – 31.16; 10.77) | 0.4909 | 20.49 (15.9 – 28; 11.03) | 0.8119 | 17.39 (15.17 – 28.16; 7.48) | 0.8798 | 13.16 (10.43 – 13.37; 0.63) | 0.7 | 21.01 (21.01 – 21.01; 0) | 1 |
| **% time COx_R > 0.3** | 16.94 (12.07 – 23.4; 8.35) |  | 25.24 (21.61 – 28.86; 10.74) |  | 20.73 (15.41 – 35.93; 11.42) |  | 24.06 (20.22 – 32.91; 11.11) |  | 18.6 (12.07 – 25.96; 10.92) |  | 16.97 (13.16 – 27.65; 6.41) |  | 13.25 (11.46 – 15.62; 5.3) |  | 15.01 (15.01 – 15.01; 0) |  |
| **% time COx-a_L > 0** | 63.77 (59.05 – 68.49; 7) | 0.8175 | 53.74 (51.33 – 56.14; 7.14) | 0.6667 | 58.16 (51.68 – 66; 11) | 0.8743 | 60.06 (53.14 – 66.58; 9.77) | 0.5391 | 62.67 (60.41 – 69.9; 8.35) | 0.4134 | 63.77 (58.52 – 68.49; 7.78) | 0.9197 | 54.2 (51.56 – 60.71; 7.83) | 1 | 59.05 (59.05 – 59.05; 0) | 1 |
| **% time COx-a_R > 0** | 64.07 (53.82 – 70.64; 12.3) |  | 59.5 (58.42 – 60.57; 3.18) |  | 54.7 (51.87 – 71.18; 7.8) |  | 60.78 (56.56 – 67.32; 9.14) |  | 59.38 (52.06 – 69.4; 14.79) |  | 62.75 (60.39 – 70.36; 11.28) |  | 57.35 (52.12 – 64.49; 15.52) |  | 53.82 (53.82 – 53.82; 0) |  |
| **% time COx-a_L > 0.2** | 36.45 (33.39 – 43.75; 8.23) | 0.5637 | 31.05 (27.68 – 34.42; 10) | 0.6667 | 29.37 (27.33 – 40.31; 10.03) | 0.8388 | 34.08 (28.37 – 39.11; 8.64) | 0.4909 | 36.45 (33.44 – 45.03; 10.83) | 0.5401 | 37.07 (32.55 – 44.04; 10.28) | 1 | 27.78 (26.04 – 32.61; 5.15) | 1 | 34.71 (34.71 – 34.71; 0) | 1 |
| **% time COx-a_R > 0.2** | 36.93 (27.87 – 43.66; 12.31) |  | 36.98 (34.8 – 39.16; 6.47) |  | 29.76 (26.61 – 47.18; 8.89) |  | 36.31 (32.38 – 40.12; 5.75) |  | 36.99 (26.07 – 41.97; 13.93) |  | 36.93 (28.62 – 43.66; 12.3) |  | 32.61 (27.82 – 37.09; 13.26) |  | 28.98 (28.98 – 28.98; 0) |  |
| **% time COx-a_L > 0.3** | 24.61 (21.91 – 30.5; 5.05) | 0.8026 | 23.01 (19.47 – 26.55; 10.49) | 0.6667 | 20.15 (16.31 – 29.3; 9.06) | 0.91 | 24.65 (20.69 – 29.5; 7.14) | 0.5896 | 25.77 (21.26 – 31.41; 7.62) | 0.6584 | 23.93 (21.84 – 31.36; 8.66) | 0.8403 | 15.93 (15.88 – 19.61; 0.16) | 1 | 24.61 (24.61 – 24.61; 0) | 1 |
| **% time COx-a_R > 0.3** | 24.09 (17.39 – 31.19; 10.52) |  | 27.37 (24.18 – 30.56; 9.46) |  | 20.5 (17.85 – 36.69; 9.26) |  | 25.36 (22.07 – 31.26; 7.9) |  | 25.1 (16.13 – 30.56; 11.44) |  | 23.95 (19.15 – 31.75; 10.73) |  | 20.99 (17.66 – 23.74; 8.14) |  | 19.3 (19.3 – 19.3; 0) |  |
| **5-Minute Data Resolution** | | | | | | | | | | | | | | | | |
| **ABP (mmHg)** | 82.37 (77.16 – 89.28) | – | 88.29 (79.49 – 97.91) | – | 80.43 (74.85 – 88.76) | – | 82.33 (76.76 – 89.09) | – | 83.4 (75.95 – 91.86) | – | 82.14 (78.25 – 89.17) | – | 84.34 (74.86 – 100.46) | – | 77.01 (70.62 – 84.02) | – |
| **CPP (mmHg)** | 72.63 (67.43 – 79.87) |  | 80.95 (71.73 – 90.98) |  | 72.66 (66.94 – 80.52) |  | 74.44 (68.17 – 81.96) |  | 74.08 (67.61 – 82.03) |  | 72.63 (68.35 – 78.38) |  | 74.64 (62.87 – 91.93) |  | 70.87 (64.84 – 77.95) |  |
| **rSO2_L (%)** | 69.91 (65.86 – 73.96) | 0.9387 | 67.62 (61.19 – 71.31) | 0.6667 | 66.98 (65.03 – 72.69) | 0.7006 | 67.99 (63.79 – 74.18) | 0.5896 | 71.08 (68.13 – 74.52) | 0.2594 | 71.35 (68.41 – 76.27) | 1 | 62.2 (55.57 – 66.06) | 0.7 | 68.23 (63.86 – 70.8) | 1 |
| **rSO2_R (%)** | 68.34 (64.31 – 74.98) |  | 64.45 (57.4 – 68.68) |  | 66.61 (63.12 – 73.67) |  | 71.54 (67.83 – 73.5) |  | 68.22 (61.99 – 73.59) |  | 68.75 (65.09 – 74.13) |  | 71.68 (68.1 – 76.8) |  | 68.34 (65.99 – 72.27) |  |
| **COx_L (au)** | 0.02 (-0.16 – 0.19) | 0.5004 | -0.01 (-0.22 – 0.21) | 0.6667 | 0.02 (-0.14 – 0.19) | 0.7688 | 0.04 (-0.14 – 0.23) | 0.423 | 0.04 (-0.15 – 0.2) | 0.4745 | 0.02 (-0.15 – 0.19) | 1 | -0.07 (-0.29 – 0.13) | 1 | 0.03 (-0.16 – 0.22) | 1 |
| **COx_R (au)** | 0.01 (-0.18 – 0.18) |  | 0.05 (-0.17 – 0.27) |  | 0.03 (-0.14 – 0.22) |  | 0.06 (-0.11 – 0.27) |  | 0.01 (-0.16 – 0.18) |  | 0.03 (-0.12 – 0.2) |  | -0.07 (-0.26 – 0.1) |  | -0.02 (-0.21 – 0.15) |  |
| **COx-a_L (au)** | 0.1 (-0.07 – 0.25) | 0.5637 | 0.02 (-0.16 – 0.23) | 0.6667 | 0.06 (-0.11 – 0.22) | 0.8743 | 0.08 (-0.1 – 0.25) | 0.5896 | 0.1 (-0.07 – 0.27) | 0.5629 | 0.1 (-0.07 – 0.27) | 0.9197 | 0.04 (-0.16 – 0.18) | 1 | 0.07 (-0.12 – 0.24) | 1 |
| **COx-a_R (au)** | 0.1 (-0.06 – 0.26) |  | 0.08 (-0.13 – 0.29) |  | 0.04 (-0.13 – 0.21) |  | 0.09 (-0.08 – 0.27) |  | 0.09 (-0.11 – 0.26) |  | 0.11 (-0.06 – 0.26) |  | 0.05 (-0.16 – 0.21) |  | 0.03 (-0.16 – 0.2) |  |
| **MAD of ABP (mmHg)** | 8.45 (7.61 – 9.69) | – | 13.93 (13.03 – 14.82) | – | 9.3 (7.53 – 11.01) | – | 7.15 (5.96 – 10.7) | – | 9.32 (7.82 – 11.29) | – | 8.19 (7.44 – 9.36) | – | 15.72 (10.63 – 24.6) | – | 9.74 (9.74 – 9.74) | – |
| **MAD of CPP (mmHg)** | 8.52 (7.37 – 9.98) |  | 14.25 (13.75 – 14.76) |  | 7.72 (6.23 – 11.22) |  | 8.9 (6.29 – 12.08) |  | 8.57 (7.37 – 10.98) |  | 8.03 (6.99 – 9.98) |  | 15.26 (10.32 – 25.25) |  | 9.55 (9.55 – 9.55) |  |
| **MAD of rSO2_L (%)** | 5.15 (3.86 – 6.87) | 0.8626 | 7.09 (6.37 – 7.81) | 1 | 3.67 (2.73 – 5.91) | 0.8743 | 4.29 (2.7 – 6.58) | 0.752 | 5.46 (3.14 – 7.3) | 0.5629 | 4.96 (4.11 – 6.87) | 0.2642 | 6.82 (5.32 – 7.68) | 0.7 | 5.15 (5.15 – 5.15) | 1 |
| **MAD of rSO2_R (%)** | 5.27 (4.25 – 6.73) |  | 7.73 (6.68 – 8.77) |  | 3.68 (3.17 – 6.85) |  | 5.1 (3.48 – 7.95) |  | 5.57 (4.12 – 8.4) |  | 4.25 (3.14 – 5.5) |  | 6.98 (6.8 – 8.4) |  | 4.28 (4.28 – 4.28) |  |
| **MAD of COx_L (au)** | 0.25 (0.24 – 0.28) | 0.6305 | 0.32 (0.31 – 0.32) | 1 | 0.27 (0.25 – 0.31) | 0.982 | 0.29 (0.25 – 0.32) | 0.4016 | 0.25 (0.23 – 0.26) | 0.5401 | 0.26 (0.24 – 0.31) | 0.8798 | 0.24 (0.23 – 0.28) | 0.4 | 0.28 (0.28 – 0.28) | 1 |
| **MAD of COx_R (au)** | 0.27 (0.24 – 0.3) |  | 0.32 (0.32 – 0.32) |  | 0.28 (0.26 – 0.3) |  | 0.31 (0.28 – 0.34) |  | 0.26 (0.22 – 0.27) |  | 0.3 (0.25 – 0.31) |  | 0.31 (0.28 – 0.32) |  | 0.27 (0.27 – 0.27) |  |
| **MAD of COx-a_L (au)** | 0.24 (0.22 – 0.27) | 0.7148 | 0.29 (0.28 – 0.3) | 0.6667 | 0.25 (0.23 – 0.3) | 0.6673 | 0.26 (0.24 – 0.29) | 0.6963 | 0.25 (0.23 – 0.26) | 0.9729 | 0.24 (0.22 – 0.28) | 0.7623 | 0.24 (0.23 – 0.25) | 0.7 | 0.27 (0.27 – 0.27) | 1 |
| **MAD of COx-a_R (au)** | 0.23 (0.22 – 0.27) |  | 0.31 (0.29 – 0.32) |  | 0.26 (0.24 – 0.3) |  | 0.27 (0.25 – 0.29) |  | 0.24 (0.23 – 0.27) |  | 0.24 (0.22 – 0.26) |  | 0.28 (0.24 – 0.29) |  | 0.26 (0.26 – 0.26) |  |
| **% time rSO2_L > 60%** | 93.05 (58.77 – 96.66; 10.31) | 0.8996 | 74.42 (66.01 – 82.82; 24.93) | 1 | 93.5 (37.59 – 99.55; 9.64) | 0.4305 | 89.62 (68.05 – 98.82; 15.32) | 0.1345 | 95.87 (65.92 – 99.64; 6.12) | 0.3341 | 92.51 (81.39 – 95.84; 10.35) | 0.7191 | 57.6 (44.02 – 76.99; 40.26) | 0.8248 | 93.05 (93.05 – 93.05; 0) | 1 |
| **% time rSO2_R > 60%** | 93.66 (55.37 – 98.21; 9.31) |  | 66.77 (53.77 – 79.76; 38.53) |  | 96.75 (45.81 – 100; 4.83) |  | 94.71 (91.06 – 100; 7.76) |  | 81.37 (55.37 – 98.21; 27.53) |  | 94.42 (80.48 – 99.75; 8.06) |  | 93.66 (67.22 – 95.02; 4.03) |  | 90.41 (90.41 – 90.41; 0) |  |
| **% time rSO2_L > 70%** | 49.4 (9.66 – 80.27; 54.3) | 0.9768 | 40.79 (26.18 – 55.4; 43.33) | 0.6667 | 29.41 (2.5 – 90.08; 43.6) | 0.6454 | 43.44 (3.16 – 87.64; 61.55) | 0.6646 | 60.52 (20.75 – 78.5; 40.1) | 0.2934 | 70.01 (9.77 – 77.14; 30.73) | 1 | 11.56 (5.89 – 51.75; 16.81) | 0.7 | 30.46 (30.46 – 30.46; 0) | 1 |
| **% time rSO2_R > 70%** | 39.14 (18.08 – 71.45; 37.88) |  | 30.91 (18.38 – 43.44; 37.15) |  | 32.51 (5.76 – 95.4; 48.15) |  | 66 (37.53 – 73.82; 28.68) |  | 39.71 (14.91 – 61.92; 36.76) |  | 36.94 (18.08 – 91.88; 53.63) |  | 64.69 (35.28 – 80.3; 46.28) |  | 34.56 (34.56 – 34.56; 0) |  |
| **% time rSO2_L > 80%** | 1.85 (0.26 – 21.65; 2.74) | 0.6537 | 5.52 (2.76 – 8.27; 8.18) | 1 | 1.38 (0 – 12.33; 2.05) | 0.758 | 4.86 (0 – 41.26; 7.2) | 0.6862 | 1.85 (0.39 – 20.19; 2.74) | 0.5089 | 9.98 (0.74 – 28.57; 14.8) | 0.8567 | 0 (0 – 16.78; 0) | 0.3758 | 0.26 (0.26 – 0.26; 0) | 1 |
| **% time rSO2_R > 80%** | 3.25 (0.79 – 20.64; 4.82) |  | 1.46 (0.84 – 2.08; 1.84) |  | 3.63 (0 – 36.72; 5.38) |  | 6.23 (0 – 21.01; 9.24) |  | 2.68 (0 – 8.82; 3.97) |  | 2.7 (1.07 – 23.62; 4) |  | 9.51 (4.87 – 37.8; 13.78) |  | 0.79 (0.79 – 0.79; 0) |  |
| **% time rSO2_L > 90%** | 0 (0 – 0.04; 0) | 0.4968 | 0 (0 – 0; 0) | – | 0 (0 – 0; 0) | 0.7857 | 0 (0 – 1.42; 0) | 0.9823 | 0 (0 – 0; 0) | 0.9815 | 0 (0 – 0.06; 0) | 0.7427 | 0 (0 – 0; 0) | 0.505 | 0 (0 – 0; 0) | – |
| **% time rSO2_R > 90%** | 0 (0 – 0.99; 0) |  | 0 (0 – 0; 0) |  | 0 (0 – 0.15; 0) |  | 0 (0 – 0.66; 0) |  | 0 (0 – 0; 0) |  | 0 (0 – 0; 0) |  | 0 (0 – 12.87; 0) |  | 0 (0 – 0; 0) |  |
| **% time COx_L > 0** | 53.07 (46.72 – 59.16; 9.41) | 0.4185 | 48.88 (44.98 – 52.77; 11.55) | 0.6667 | 53.69 (48.32 – 66.77; 14.31) | 0.9459 | 57.84 (51.46 – 67.7; 11.12) | 0.5896 | 55.5 (46.72 – 67.57; 17.89) | 0.4182 | 53.07 (45.63 – 59.16; 11.02) | 0.8403 | 41.09 (33.59 – 46.28; 15.4) | 1 | 54.03 (54.03 – 54.03; 0) | 1 |
| **% time COx_R > 0** | 52.32 (40.41 – 59.96; 16.81) |  | 56.41 (52.93 – 59.89; 10.32) |  | 54.07 (48.24 – 72.1; 14.94) |  | 58.43 (53.02 – 65.45; 8.75) |  | 51.37 (40.99 – 59.96; 15.39) |  | 54.46 (43.87 – 63.37; 15.71) |  | 39.65 (36.59 – 44.55; 9.06) |  | 47.55 (47.55 – 47.55; 0) |  |
| **% time COx_L > 0.2** | 24.3 (20.66 – 31.42; 7.01) | 0.4526 | 24.57 (20.11 – 29.03; 13.22) | 0.6667 | 24.31 (20.06 – 36.97; 9.39) | 0.6347 | 28.66 (24.04 – 39.87; 9.69) | 0.423 | 25.53 (20.66 – 36.12; 14.06) | 0.6584 | 24.3 (20.87 – 33.48; 10.59) | 0.9598 | 15.65 (12.03 – 17.61; 5.81) | 0.7 | 27.01 (27.01 – 27.01; 0) | 1 |
| **% time COx_R > 0.2** | 23.25 (13.95 – 28.16; 13.79) |  | 31.22 (26.92 – 35.52; 12.75) |  | 26.86 (20.6 – 44.69; 12.68) |  | 31.18 (27.01 – 43.33; 13.19) |  | 23.25 (13.95 – 35.9; 14.3) |  | 24.18 (18.74 – 36.84; 13.58) |  | 16.41 (14.08 – 19.52; 6.92) |  | 18.41 (18.41 – 18.41; 0) |  |
| **% time COx_L > 0.3** | 15.09 (11.34 – 19.39; 6.37) | 0.5637 | 16.48 (12.37 – 20.59; 12.18) | 0.6667 | 15.09 (11.62 – 25.75; 7.08) | 0.7345 | 18.81 (14.18 – 28.2; 10.3) | 0.386 | 16.32 (12.03 – 23.08; 10.01) | 0.7857 | 15.09 (11.34 – 24.35; 8.56) | 0.801 | 8.26 (6.63 – 8.69; 1.27) | 0.7 | 16.39 (16.39 – 16.39; 0) | 1 |
| **% time COx_R > 0.3** | 12.91 (8.02 – 18.59; 8.37) |  | 21.33 (16.87 – 25.79; 13.21) |  | 17.28 (12.29 – 34.18; 11.71) |  | 22.02 (17.89 – 30.01; 11.04) |  | 13.7 (8.84 – 21.81; 8.86) |  | 13.1 (10.54 – 23.93; 7.53) |  | 8.59 (7.86 – 10.5; 2.14) |  | 10.45 (10.45 – 10.45; 0) |  |
| **% time COx-a_L > 0** | 66.07 (60.33 – 71.36; 8.52) | 0.8026 | 53.3 (50.04 – 56.56; 9.66) | 0.6667 | 59.14 (53.18 – 68.8; 13.12) | 0.7688 | 61.18 (54.13 – 69.4; 11.99) | 0.4016 | 64.83 (61.19 – 74.36; 10.71) | 0.394 | 66.07 (59.58 – 70.81; 9.62) | 0.9197 | 56.3 (51.54 – 61.99; 14.11) | 1 | 60.33 (60.33 – 60.33; 0) | 1 |
| **% time COx-a_R > 0** | 66.25 (54.18 – 74.49; 14.31) |  | 60.49 (59.22 – 61.76; 3.76) |  | 54.99 (53.19 – 71.77; 11.66) |  | 64.49 (56.13 – 69.8; 11.88) |  | 58.97 (53.53 – 73.9; 13.74) |  | 66.01 (62.5 – 71.85; 8.67) |  | 57.95 (52.16 – 66.26; 17.19) |  | 54.18 (54.18 – 54.18; 0) |  |
| **% time COx-a_L > 0.2** | 33.33 (29.85 – 42.65; 10.28) | 0.5637 | 27 (23.05 – 30.95; 11.71) | 0.6667 | 28.31 (22.83 – 37.98; 10.04) | 0.9459 | 31.62 (26.19 – 37.7; 9.19) | 0.4909 | 33.87 (29.5 – 42.65; 10.6) | 0.4745 | 34.89 (30.07 – 42.91; 11.89) | 0.9387 | 23.14 (21.12 – 28.09; 5.99) | 1 | 30.3 (30.3 – 30.3; 0) | 1 |
| **% time COx-a_R > 0.2** | 33.48 (22.8 – 41.87; 14.6) |  | 33.9 (30.24 – 37.55; 10.85) |  | 26.06 (22.9 – 47.34; 7.6) |  | 33.81 (28.38 – 37.83; 7.72) |  | 33.48 (20.92 – 38.94; 14.6) |  | 36.24 (24.39 – 41.87; 11.27) |  | 26.58 (22.76 – 32.6; 11.34) |  | 25.07 (25.07 – 25.07; 0) |  |
| **% time COx-a_L > 0.3** | 20.02 (18.17 – 25.25; 4.72) | 0.8325 | 17.83 (14.28 – 21.38; 10.52) | 0.6667 | 15.59 (13.99 – 25.89; 7.66) | 0.8036 | 19.68 (16.43 – 26.52; 5.66) | 0.8381 | 21.9 (17.19 – 29.06; 7.5) | 0.5861 | 20.83 (18.22 – 27.21; 6.08) | 1 | 11.05 (10.89 – 14.61; 0.48) | 1 | 20.02 (20.02 – 20.02; 0) | 1 |
| **% time COx-a_R > 0.3** | 20.4 (14.15 – 27.75; 10.91) |  | 24.21 (20.71 – 27.71; 10.38) |  | 16.55 (13.53 – 34.09; 7.96) |  | 20.8 (17.6 – 26.25; 6.56) |  | 17.95 (11.56 – 25.77; 10.92) |  | 20.4 (15.73 – 29.22; 10.91) |  | 17.21 (13.34 – 19.26; 6.07) |  | 14.97 (14.97 – 14.97; 0) |  |
| *The p-values in the table are derived using Mann-Whitney U test between the bilateral signals.*  *ABP, arterial blood pressure; au, arbitrary units; CPP, cerebral perfusion pressure; COx, cerebral oximetry index with CPP; COx-a, cerebral oximetry index with ABP; CT, computed tomography; DAI, diffuse axonal injury; EDH, epidural hematoma; MAD, median absolute deviation; IQR, interquartile range; mmHg, millimeters of mercury; rSO2, regional cerebral oxygen saturation; tSAH, traumatic subarachnoid hemorrhage; SDH, subdural hematoma; aSDH, acute subdural hematoma; TBI-GLR, traumatic brain injury patient group without bifrontal lobe pathology.* | | | | | | | | | | | | | | | | |

| **Physiologic Variable** | **Sub-groups** | | | | | | | | | | | | | | | |
| --- | --- | --- | --- | --- | --- | --- | --- | --- | --- | --- | --- | --- | --- | --- | --- | --- |
|  | **No Anesthetic [n = 2]** | | **Propofol [n = 14]** | | **Fentanyl + Propofol [n = 22]** | | **Ketamine + Propofol [n = 2]** | | **Midazolam + Propofol [n = 1]** | | **Fentanyl + Ketamine + Propofol [n = 5]** | | **Fentanyl + Midazolam + Propofol [n = 13]** | | **Fentanyl + Ketamine + Midazolam + Propofol [n = 5]** | |
|  | **Median (IQR) or  Median (IQR; MAD)** | **p-value** | **Median (IQR) or  Median (IQR; MAD)** | **p-value** | **Median (IQR) or  Median (IQR; MAD)** | **p-value** | **Median (IQR) or  Median (IQR; MAD)** | **p-value** | **Median (IQR) or  Median (IQR; MAD)** | **p-value** | **Median (IQR) or  Median (IQR; MAD)** | **p-value** | **Median (IQR) or  Median (IQR; MAD)** | **p-value** | **Median (IQR) or  Median (IQR; MAD)** | **p-value** |
| **10-Second Data Resolution** | | | | | | | | | | | | | | | | |
| **ABP (mmHg)** | 86.4 (80.38 – 93.16) | – | 79.35 (74.01 – 91.15) | – | 80.23 (75.6 – 86.61) | – | 83.24 (76.76 – 90.72) | – | 77.69 (74.53 – 82.52) | – | 80.15 (73 – 88.41) | – | 83.73 (77.08 – 89.69) | – | 90.85 (84.46 – 99.41) | – |
| **CPP (mmHg)** | 82.82 (75.38 – 91.53) |  | 77 (68.69 – 87.65) |  | 71.4 (65.6 – 78.76) |  | 79.05 (70.55 – 85.94) |  | 65.9 (62.54 – 70.4) |  | 73.32 (64.42 – 78.47) |  | 73.18 (67.54 – 77.9) |  | 75.31 (67.31 – 83.87) |  |
| **rSO2_L (%)** | 65 (59.89 – 70.25) | 1 | 72.49 (69.17 – 76.18) | 1 | 68.69 (65.5 – 74.99) | 0.716 | 70.54 (66.36 – 74.25) | 1 | 76.99 (72.25 – 81.62) | 1 | 67.99 (63.99 – 70.99) | 1 | 71.5 (69 – 75.75) | 0.8979 | 65.99 (62.7 – 67.99) | 0.2492 |
| **rSO2_R (%)** | 59.3 (57.06 – 62.49) |  | 71.37 (67.75 – 73.75) |  | 69.07 (65.5 – 75.44) |  | 70.75 (66.52 – 74.15) |  | 87 (76.9 – 89.99) |  | 68.11 (65.99 – 70.99) |  | 67.02 (62.99 – 73.8) |  | 66.99 (63.99 – 69) |  |
| **COx_L (au)** | 0 (-0.16 – 0.21) | 1 | 0 (-0.19 – 0.22) | 0.5484 | 0.02 (-0.12 – 0.26) | 0.3518 | -0.04 (-0.29 – 0.2) | 0.6667 | -0.15 (-0.33 – 0.05) | 1 | 0 (-0.15 – 0.21) | 0.5179 | 0.01 (-0.18 – 0.22) | 0.2117 | 0 (-0.14 – 0.21) | 0.6558 |
| **COx_R (au)** | 0.07 (-0.16 – 0.32) |  | 0 (-0.22 – 0.21) |  | 0.06 (-0.11 – 0.32) |  | -0.01 (-0.25 – 0.23) |  | -0.1 (-0.29 – 0.1) |  | 0 (-0.09 – 0.17) |  | 0 (-0.19 – 0.23) |  | 0 (-0.19 – 0.23) |  |
| **COx-a_L (au)** | 0.01 (-0.05 – 0.31) | 1 | 0.01 (-0.16 – 0.23) | 0.5195 | 0.07 (-0.07 – 0.28) | 0.5334 | 0.05 (-0.19 – 0.27) | 0.6667 | 0.12 (-0.07 – 0.29) | 1 | 0.08 (-0.1 – 0.3) | 1 | 0.08 (-0.07 – 0.31) | 0.1578 | 0.03 (-0.08 – 0.28) | 1 |
| **COx-a_R (au)** | 0.15 (-0.05 – 0.39) |  | 0.02 (-0.17 – 0.25) |  | 0.09 (-0.07 – 0.33) |  | 0.01 (-0.22 – 0.23) |  | 0.15 (-0.03 – 0.32) |  | 0.02 (-0.07 – 0.24) |  | 0.03 (-0.12 – 0.3) |  | 0.03 (-0.12 – 0.27) |  |
| **MAD of ABP (mmHg)** | 9.37 (8.08 – 10.66) | – | 11.08 (7.55 – 12.38) | – | 8.72 (7.08 – 9.7) | – | 10.08 (10.03 – 10.13) | – | 5.57 (5.57 – 5.57) | – | 10.22 (7.62 – 11.12) | – | 7.99 (6.61 – 9.84) | – | 10.76 (10.24 – 11.92) | – |
| **MAD of CPP (mmHg)** | 11.64 (9.04 – 14.24) |  | 11.3 (6.4 – 12.62) |  | 8.16 (6.62 – 10.14) |  | 11.55 (10.13 – 12.97) |  | 5.68 (5.68 – 5.68) |  | 8.73 (7.4 – 9.89) |  | 8.35 (6.89 – 10.63) |  | 11.34 (11.25 – 12.32) |  |
| **MAD of rSO2_L (%)** | 7.7 (5.33 – 10.07) | 0.6667 | 5.93 (3.58 – 5.94) | 0.0588 | 4.64 (3.25 – 7.21) | 0.3072 | 5.97 (4.52 – 7.43) | 0.6667 | 6.97 (6.97 – 6.97) | 1 | 5.22 (2.97 – 9.98) | 0.6752 | 4.09 (3.75 – 6.75) | 0.2869 | 5.2 (2.97 – 5.92) | 0.4206 |
| **MAD of rSO2_R (%)** | 3.62 (2.92 – 4.31) |  | 4.13 (2.95 – 4.45) |  | 5.86 (3.8 – 7.99) |  | 5.2 (4.09 – 6.32) |  | 7.21 (7.21 – 7.21) |  | 4.45 (2.95 – 4.49) |  | 5.93 (4.45 – 7.46) |  | 5.93 (3.74 – 9.78) |  |
| **MAD of COx_L (au)** | 0.25 (0.25 – 0.25) | 0.3333 | 0.32 (0.28 – 0.37) | 0.7345 | 0.31 (0.27 – 0.34) | 0.6009 | 0.37 (0.35 – 0.39) | 0.6667 | 0.28 (0.28 – 0.28) | 1 | 0.34 (0.27 – 0.35) | 0.6905 | 0.31 (0.29 – 0.35) | 0.9598 | 0.25 (0.22 – 0.3) | 0.5476 |
| **MAD of COx_R (au)** | 0.35 (0.34 – 0.36) |  | 0.33 (0.29 – 0.38) |  | 0.33 (0.27 – 0.36) |  | 0.35 (0.34 – 0.36) |  | 0.29 (0.29 – 0.29) |  | 0.32 (0.18 – 0.33) |  | 0.32 (0.28 – 0.35) |  | 0.28 (0.27 – 0.31) |  |
| **MAD of COx-a_L (au)** | 0.23 (0.22 – 0.24) | 0.3333 | 0.3 (0.27 – 0.34) | 0.8388 | 0.3 (0.27 – 0.32) | 0.2578 | 0.34 (0.31 – 0.37) | 1 | 0.27 (0.27 – 0.27) | 1 | 0.29 (0.25 – 0.34) | 0.8413 | 0.28 (0.26 – 0.3) | 0.9183 | 0.25 (0.23 – 0.3) | 0.5476 |
| **MAD of COx-a_R (au)** | 0.32 (0.32 – 0.33) |  | 0.3 (0.28 – 0.34) |  | 0.32 (0.28 – 0.33) |  | 0.33 (0.32 – 0.35) |  | 0.26 (0.26 – 0.26) |  | 0.27 (0.18 – 0.32) |  | 0.28 (0.27 – 0.29) |  | 0.27 (0.27 – 0.3) |  |
| **% time rSO2_L > 60%** | 40.8 (20.4 – 61.19; 60.48) | 1 | 96.87 (91.32 – 99.85; 4.64) | 0.7284 | 86.36 (37.36 – 99.16; 20.21) | 0.275 | 83.78 (76.54 – 91.02; 21.46) | 1 | 96.5 (96.5 – 96.5; 0) | 1 | 92.79 (50.91 – 99.97; 10.68) | 0.6905 | 91.17 (72.04 – 98.4; 12.93) | 0.5214 | 88.87 (85.01 – 93.08; 6.24) | 0.8413 |
| **% time rSO2_R > 60%** | 47.89 (23.94 – 71.83; 70.99) |  | 98.2 (92.2 – 100; 2.67) |  | 94.02 (54.71 – 99.83; 8.82) |  | 80.71 (71.29 – 90.14; 27.95) |  | 96.49 (96.49 – 96.49; 0) |  | 89.87 (66.57 – 99.94; 14.94) |  | 85.96 (60.21 – 93.9; 20.81) |  | 89.82 (88.12 – 93.79; 5.88) |  |
| **% time rSO2_L > 70%** | 36.15 (18.08 – 54.23; 53.6) | 1 | 69.01 (28.54 – 75.96; 36.68) | 0.8361 | 40.3 (4.06 – 85.17; 58.31) | 0.8418 | 60.27 (42.72 – 77.81; 52.03) | 0.6667 | 90.47 (90.47 – 90.47; 0) | 1 | 29.02 (4.47 – 67.69; 42.83) | 0.8413 | 62.59 (9.59 – 84.09; 41.68) | 0.9387 | 8.59 (4.46 – 21.91; 7.46) | 0.3095 |
| **% time rSO2_R > 70%** | 45.83 (22.92 – 68.75; 67.95) |  | 60.43 (9.77 – 97.4; 58.6) |  | 43.09 (32.52 – 69.25; 38.2) |  | 52.61 (31.3 – 73.91; 63.18) |  | 96.18 (96.18 – 96.18; 0) |  | 32.5 (2.08 – 32.88; 45.1) |  | 34.58 (32.39 – 72.06; 39.08) |  | 21.05 (16.41 – 39.05; 9.87) |  |
| **% time rSO2_L > 80%** | 20.01 (10.01 – 30.02; 29.67) | 1 | 10.4 (0.23 – 29.56; 15.42) | 0.7959 | 1.53 (0.01 – 21.88; 2.27) | 0.7863 | 6.81 (3.87 – 9.74; 8.7) | 1 | 32.88 (32.88 – 32.88; 0) | 1 | 0.25 (0 – 2.37; 0.37) | 1 | 5.53 (0.48 – 24.15; 8.2) | 0.797 | 1.12 (0 – 1.48; 1.66) | 0.6752 |
| **% time rSO2_R > 80%** | 18.05 (9.03 – 27.08; 26.77) |  | 3.26 (0 – 34.45; 4.83) |  | 6.95 (0.1 – 17.88; 10.23) |  | 10.44 (5.22 – 15.66; 15.47) |  | 66.25 (66.25 – 66.25; 0) |  | 0.57 (0 – 1.86; 0.85) |  | 5.05 (0.25 – 22.85; 7.33) |  | 0.96 (0.5 – 2.19; 1.42) |  |
| **% time rSO2_L > 90%** | 9.21 (4.6 – 13.81; 13.65) | 1 | 0 (0 – 0; 0) | 0.6946 | 0.01 (0 – 0.86; 0.01) | 0.4407 | 0.17 (0.08 – 0.25; 0.25) | 1 | 0.04 (0.04 – 0.04; 0) | 1 | 0 (0 – 0; 0) | 1 | 0 (0 – 0.21; 0.01) | 0.8694 | 0 (0 – 0.01; 0) | 0.9063 |
| **% time rSO2_R > 90%** | 0.53 (0.26 – 0.79; 0.78) |  | 0 (0 – 0.02; 0) |  | 0.04 (0 – 0.58; 0.06) |  | 0.06 (0.03 – 0.09; 0.09) |  | 24.7 (24.7 – 24.7; 0) |  | 0 (0 – 0; 0) |  | 0 (0 – 0.54; 0) |  | 0 (0 – 0.02; 0) |  |
| **% time COx_L > 0** | 35.42 (28.68 – 42.17; 20.01) | 0.6667 | 48.51 (46.11 – 54.17; 5.3) | 0.982 | 53.21 (43.51 – 62.2; 13.64) | 0.4085 | 44.67 (43.38 – 45.96; 3.81) | 0.6667 | 29.86 (29.86 – 29.86; 0) | 1 | 48.82 (42.56 – 54.64; 9.29) | 0.4206 | 50.82 (42.54 – 52.65; 9.92) | 0.4184 | 49.18 (46.96 – 49.3; 1.34) | 0.5476 |
| **% time COx_R > 0** | 58.14 (46.41 – 69.88; 34.79) |  | 50.67 (45.01 – 53.06; 7.18) |  | 56.97 (45.67 – 66.48; 15.36) |  | 48.43 (46.8 – 50.07; 4.85) |  | 35.96 (35.96 – 35.96; 0) |  | 43.69 (40.46 – 45.66; 4.8) |  | 47.22 (44.11 – 48.78; 4.61) |  | 49.9 (47.4 – 51.04; 3.7) |  |
| **% time COx_L > 0.2** | 25.13 (18.62 – 31.64; 19.31) | 0.6667 | 26.65 (22.62 – 29.59; 5.68) | 0.982 | 29.91 (25.74 – 38.64; 8.48) | 0.2887 | 25.45 (24.69 – 26.21; 2.25) | 0.6667 | 12.71 (12.71 – 12.71; 0) | 1 | 26.43 (23.35 – 31.27; 7.17) | 0.4206 | 26.81 (24.45 – 37.38; 10.72) | 0.8403 | 26.11 (25.61 – 30.98; 5.08) | 1 |
| **% time COx_R > 0.2** | 39.61 (28.9 – 50.32; 31.76) |  | 25.48 (22.09 – 34.99; 7.82) |  | 34.24 (26.83 – 44.2; 14.62) |  | 27.2 (25.92 – 28.48; 3.8) |  | 16.06 (16.06 – 16.06; 0) |  | 21.93 (20.09 – 23.26; 2.72) |  | 27.5 (22.16 – 29.77; 7.91) |  | 27.62 (25.4 – 31.77; 6.15) |  |
| **% time COx_L > 0.3** | 20.71 (14.76 – 26.66; 17.64) | 0.6667 | 17.61 (14.57 – 20.74; 5.13) | 0.91 | 21.96 (17.57 – 27.68; 8.24) | 0.3219 | 17.77 (17.3 – 18.25; 1.4) | 0.6667 | 8.09 (8.09 – 8.09; 0) | 1 | 16.23 (16.14 – 21.73; 6.36) | 0.5476 | 17.95 (15.9 – 28.45; 9.06) | 0.8798 | 18.28 (17.48 – 22.01; 5.52) | 1 |
| **% time COx_R > 0.3** | 31.06 (22.21 – 39.91; 26.24) |  | 17.03 (14.9 – 25.11; 7.79) |  | 26.52 (19.04 – 33.55; 11.21) |  | 19.26 (18.14 – 20.39; 3.34) |  | 10.31 (10.31 – 10.31; 0) |  | 15.1 (13.78 – 16.27; 1.95) |  | 17.81 (13.78 – 22.6; 7.1) |  | 19.25 (16 – 23.72; 6.63) |  |
| **% time COx-a_L > 0** | 44.93 (42.05 – 47.81; 8.54) | 0.6667 | 51.75 (47.91 – 58.63; 9.57) | 0.91 | 57.73 (48.65 – 66.1; 13.36) | 0.5847 | 56.12 (51.87 – 60.37; 12.61) | 0.6667 | 65.96 (65.96 – 65.96; 0) | 1 | 58.6 (45.91 – 64.72; 15.17) | 0.8413 | 58.05 (53.51 – 67.74; 9.5) | 0.1254 | 53.05 (52.83 – 53.41; 0.53) | 0.8413 |
| **% time COx-a_R > 0** | 67.09 (58.53 – 75.65; 25.39) |  | 53.25 (45.12 – 59.12; 10.33) |  | 60.41 (51.1 – 69.88; 14.5) |  | 51.42 (49.29 – 53.56; 6.33) |  | 70.67 (70.67 – 70.67; 0) |  | 51.73 (38.69 – 66.75; 21.95) |  | 51.56 (50.98 – 58.26; 5.07) |  | 54.22 (51.61 – 57.67; 5.12) |  |
| **% time COx-a_L > 0.2** | 31.31 (27.01 – 35.62; 12.76) | 0.6667 | 27.98 (23.68 – 34.29; 8.76) | 0.7006 | 32.81 (27.34 – 41.36; 10.56) | 0.4925 | 33.82 (30.91 – 36.74; 8.63) | 0.6667 | 37.73 (37.73 – 37.73; 0) | 1 | 35.18 (25.26 – 37.57; 14.71) | 0.8413 | 36.07 (32.81 – 44.14; 7.59) | 0.3358 | 31.73 (29.68 – 33.74; 3.04) | 1 |
| **% time COx-a_R > 0.2** | 45.64 (36.98 – 54.3; 25.68) |  | 29.5 (22.71 – 38.76; 12.58) |  | 38.11 (30.26 – 44.98; 11.21) |  | 28.4 (27.46 – 29.34; 2.79) |  | 41.87 (41.87 – 41.87; 0) |  | 28.37 (20.81 – 37.46; 13.48) |  | 33.28 (28.5 – 40.53; 7.45) |  | 30.54 (28.79 – 36.77; 4.05) |  |
| **% time COx-a_L > 0.3** | 25.23 (20.88 – 29.58; 12.89) | 0.6667 | 19.07 (14.96 – 23.79; 7.1) | 0.7006 | 23.63 (18.5 – 29.58; 8.58) | 0.3335 | 22.99 (21.67 – 24.31; 3.91) | 0.3333 | 23.98 (23.98 – 23.98; 0) | 1 | 24.78 (17.45 – 25.11; 10.87) | 0.6905 | 25.79 (23.17 – 31.21; 6.7) | 0.4483 | 23.11 (19.64 – 23.62; 1.41) | 1 |
| **% time COx-a_R > 0.3** | 34.76 (27.06 – 42.46; 22.83) |  | 19.45 (15.52 – 27.59; 7.27) |  | 27.57 (21.55 – 33.6; 9.21) |  | 19.4 (18.99 – 19.8; 1.2) |  | 27.19 (27.19 – 27.19; 0) |  | 19.34 (15.11 – 24.5; 7.64) |  | 24.62 (19.31 – 26.87; 7.88) |  | 22.64 (19.05 – 25.91; 5.32) |  |
| **1-Minute Data Resolution** | | | | | | | | | | | | | | | | |
| **ABP (mmHg)** | 86.49 (80.54 – 92.99) | – | 79.39 (73.85 – 91.27) | – | 80.22 (75.73 – 86.59) | – | 83.56 (77.23 – 90.86) | – | 77.71 (74.7 – 82.68) | – | 80.44 (73.14 – 88.23) | – | 83.79 (77.19 – 89.63) | – | 90.84 (84.63 – 99.2) | – |
| **CPP (mmHg)** | 82.84 (75.59 – 91.33) |  | 77.48 (69.11 – 88) |  | 71.45 (65.68 – 78.47) |  | 79.37 (70.65 – 85.89) |  | 65.97 (62.66 – 70.06) |  | 73.66 (64.52 – 78.29) |  | 73.18 (67.61 – 77.84) |  | 75.27 (67.42 – 83.76) |  |
| **rSO2_L (%)** | 64.95 (59.82 – 70.2) | 1 | 72.49 (69.09 – 76.08) | 1 | 68.59 (65.51 – 74.92) | 0.7187 | 70.64 (66.45 – 74.42) | 1 | 76.99 (72.36 – 81.62) | 1 | 68.24 (63.83 – 70.83) | 1 | 71.56 (69.08 – 75.59) | 0.8798 | 65.99 (62.64 – 67.99) | 0.3095 |
| **rSO2_R (%)** | 59.25 (57.05 – 62.47) |  | 71.24 (67.76 – 73.64) |  | 69.16 (65.58 – 75.44) |  | 70.78 (66.74 – 74.08) |  | 87.03 (76.63 – 90.14) |  | 68.32 (65.99 – 71.03) |  | 67.21 (63.07 – 73.75) |  | 66.81 (63.99 – 69) |  |
| **COx_L (au)** | 0.05 (-0.19 – 0.32) | 0.6667 | 0 (-0.2 – 0.22) | 0.8388 | 0.06 (-0.14 – 0.29) | 0.4248 | -0.04 (-0.28 – 0.2) | 0.6667 | -0.16 (-0.33 – 0.04) | 1 | 0.02 (-0.16 – 0.21) | 0.8413 | 0.01 (-0.17 – 0.23) | 0.9197 | 0.03 (-0.16 – 0.24) | 0.6905 |
| **COx_R (au)** | 0.07 (-0.15 – 0.31) |  | 0.02 (-0.22 – 0.22) |  | 0.09 (-0.11 – 0.33) |  | -0.02 (-0.24 – 0.23) |  | -0.1 (-0.29 – 0.1) |  | 0 (-0.2 – 0.19) |  | 0.02 (-0.21 – 0.24) |  | 0.02 (-0.19 – 0.23) |  |
| **COx-a_L (au)** | 0.17 (-0.08 – 0.41) | 0.6667 | 0.03 (-0.16 – 0.23) | 0.7006 | 0.09 (-0.08 – 0.32) | 0.6672 | 0.05 (-0.18 – 0.26) | 1 | 0.12 (-0.07 – 0.29) | 1 | 0.08 (-0.16 – 0.29) | 1 | 0.12 (-0.09 – 0.31) | 0.6139 | 0.08 (-0.11 – 0.27) | 0.5476 |
| **COx-a_R (au)** | 0.16 (-0.05 – 0.38) |  | 0.03 (-0.17 – 0.25) |  | 0.12 (-0.09 – 0.35) |  | 0.01 (-0.2 – 0.23) |  | 0.15 (-0.03 – 0.31) |  | 0.03 (-0.16 – 0.24) |  | 0.1 (-0.13 – 0.31) |  | 0.07 (-0.14 – 0.27) |  |
| **MAD of ABP (mmHg)** | 9.1 (7.73 – 10.46) | – | 11.1 (7.27 – 12.71) | – | 8.54 (6.9 – 9.43) | – | 9.9 (9.88 – 9.92) | – | 5.55 (5.55 – 5.55) | – | 10.02 (7.41 – 11.22) | – | 7.79 (6.39 – 9.71) | – | 10.51 (10.06 – 11.87) | – |
| **MAD of CPP (mmHg)** | 11.38 (8.9 – 13.86) |  | 11.13 (6.4 – 12.72) |  | 7.82 (6.47 – 9.72) |  | 11.26 (9.73 – 12.8) |  | 5.49 (5.49 – 5.49) |  | 8.44 (7.24 – 9.83) |  | 8 (6.88 – 10.14) |  | 11.21 (11.11 – 12.17) |  |
| **MAD of rSO2_L (%)** | 7.66 (5.31 – 10) | 0.6667 | 5.75 (3.71 – 6.02) | 0.0556 | 4.6 (3.14 – 6.91) | 0.3105 | 5.83 (4.46 – 7.19) | 0.6667 | 6.87 (6.87 – 6.87) | 1 | 5.19 (2.77 – 9.9) | 0.4206 | 4.19 (3.78 – 6.77) | 0.3107 | 5.21 (2.97 – 5.92) | 0.5476 |
| **MAD of rSO2_R (%)** | 3.57 (2.83 – 4.31) |  | 3.91 (2.78 – 4.46) |  | 5.83 (3.9 – 8.02) |  | 5.27 (4.18 – 6.36) |  | 7 (7 – 7) |  | 4.45 (2.71 – 4.49) |  | 5.95 (4.26 – 7.83) |  | 6.16 (3.79 – 10.01) |  |
| **MAD of COx_L (au)** | 0.38 (0.36 – 0.4) | 0.6667 | 0.32 (0.29 – 0.35) | 0.5409 | 0.31 (0.29 – 0.35) | 0.7537 | 0.35 (0.34 – 0.37) | 0.6667 | 0.28 (0.28 – 0.28) | 1 | 0.33 (0.28 – 0.34) | 1 | 0.31 (0.29 – 0.36) | 0.6498 | 0.29 (0.29 – 0.3) | 0.4206 |
| **MAD of COx_R (au)** | 0.34 (0.33 – 0.35) |  | 0.33 (0.31 – 0.37) |  | 0.33 (0.29 – 0.35) |  | 0.34 (0.33 – 0.35) |  | 0.28 (0.28 – 0.28) |  | 0.32 (0.3 – 0.32) |  | 0.34 (0.33 – 0.38) |  | 0.3 (0.3 – 0.31) |  |
| **MAD of COx-a_L (au)** | 0.37 (0.35 – 0.38) | 0.3333 | 0.3 (0.29 – 0.32) | 0.6673 | 0.29 (0.28 – 0.33) | 0.3335 | 0.33 (0.3 – 0.35) | 1 | 0.26 (0.26 – 0.26) | 1 | 0.3 (0.28 – 0.34) | 0.6905 | 0.29 (0.26 – 0.34) | 0.5788 | 0.28 (0.28 – 0.28) | 0.5476 |
| **MAD of COx-a_R (au)** | 0.31 (0.31 – 0.32) |  | 0.3 (0.29 – 0.36) |  | 0.31 (0.29 – 0.34) |  | 0.32 (0.3 – 0.34) |  | 0.25 (0.25 – 0.25) |  | 0.29 (0.26 – 0.32) |  | 0.3 (0.27 – 0.33) |  | 0.29 (0.29 – 0.3) |  |
| **% time rSO2_L > 60%** | 40.8 (20.4 – 61.2; 60.49) | 1 | 97.21 (91.49 – 99.79; 4.14) | 0.6433 | 86.36 (37.91 – 99.24; 20.23) | 0.2745 | 84.21 (77.15 – 91.27; 20.94) | 1 | 96.47 (96.47 – 96.47; 0) | – | 93.17 (51.42 – 99.97; 10.12) | 0.9166 | 92.13 (72.71 – 98.36; 11.6) | 0.538 | 89.28 (85.84 – 93.78; 6.67) | 0.8413 |
| **% time rSO2_R > 60%** | 47.84 (23.92 – 71.76; 70.93) |  | 98.69 (92.59 – 99.99; 1.94) |  | 94.59 (54.61 – 99.86; 8.02) |  | 81.16 (71.94 – 90.37; 27.31) |  | 96.47 (96.47 – 96.47; 0) |  | 90.19 (67.47 – 99.97; 14.54) |  | 85.88 (60.97 – 95.15; 20.77) |  | 90.11 (88.43 – 94.16; 6) |  |
| **% time rSO2_L > 70%** | 36.54 (18.27 – 54.82; 54.18) | 1 | 69.93 (29.09 – 77.73; 36.22) | 0.8539 | 40.55 (4.08 – 85.89; 58.39) | 0.8235 | 61.5 (43.95 – 79.04; 52.03) | 0.6667 | 91.86 (91.86 – 91.86; 0) | 1 | 30.57 (5.08 – 70.08; 45.08) | 0.8413 | 64.99 (9.67 – 84.91; 38.63) | 0.9387 | 9.01 (4.91 – 23.09; 7.1) | 0.3095 |
| **% time rSO2_R > 70%** | 46.03 (23.01 – 69.04; 68.24) |  | 61.75 (9.82 – 97.57; 56.71) |  | 43.28 (32.44 – 71.14; 41.05) |  | 53.6 (32.2 – 75; 63.47) |  | 96.19 (96.19 – 96.19; 0) |  | 33.75 (2.32 – 33.92; 46.6) |  | 35.03 (33.39 – 72.57; 39.25) |  | 21.19 (17.27 – 40.48; 9.39) |  |
| **% time rSO2_L > 80%** | 20.28 (10.14 – 30.42; 30.06) | 1 | 11.06 (0.28 – 30.98; 16.39) | 0.7959 | 1.63 (0.02 – 21.94; 2.42) | 0.8406 | 7.23 (4.1 – 10.35; 9.27) | 1 | 33.8 (33.8 – 33.8; 0) | 1 | 0.25 (0 – 2.41; 0.37) | 1 | 5.89 (0.49 – 24.42; 8.73) | 0.797 | 1.19 (0 – 1.5; 1.77) | 0.6752 |
| **% time rSO2_R > 80%** | 18.84 (9.42 – 28.26; 27.93) |  | 3.07 (0 – 36.14; 4.55) |  | 7.23 (0.11 – 17.3; 10.66) |  | 10.84 (5.42 – 16.25; 16.06) |  | 66.43 (66.43 – 66.43; 0) |  | 0.74 (0 – 1.98; 1.1) |  | 5.73 (0.24 – 23.95; 8.32) |  | 1 (0.66 – 2.41; 1.47) |  |
| **% time rSO2_L > 90%** | 9.19 (4.6 – 13.79; 13.63) | 1 | 0 (0 – 0; 0) | 0.949 | 0 (0 – 0.85; 0) | 0.4336 | 0.21 (0.1 – 0.31; 0.31) | 0.6171 | 0.05 (0.05 – 0.05; 0) | 1 | 0 (0 – 0; 0) | 0.4237 | 0 (0 – 0.15; 0) | 0.9777 | 0 (0 – 0.01; 0) | 0.9063 |
| **% time rSO2_R > 90%** | 0.6 (0.3 – 0.89; 0.88) |  | 0 (0 – 0; 0) |  | 0.05 (0 – 0.57; 0.07) |  | 0 (0 – 0; 0) |  | 25.75 (25.75 – 25.75; 0) |  | 0 (0 – 0; 0) |  | 0 (0 – 0.56; 0) |  | 0 (0 – 0.02; 0) |  |
| **% time COx_L > 0** | 53.56 (42.37 – 64.75; 33.18) | 0.6667 | 50.05 (46.69 – 55.32; 6.97) | 0.8743 | 57.05 (51.56 – 65.2; 11.18) | 0.4813 | 44.78 (43.22 – 46.33; 4.62) | 0.6667 | 29.75 (29.75 – 29.75; 0) | 1 | 53.62 (52.43 – 55.19; 2.32) | 0.6905 | 51.48 (47.78 – 64.65; 9.02) | 0.8403 | 54.12 (51.51 – 54.41; 3.88) | 0.5476 |
| **% time COx_R > 0** | 58.23 (46.13 – 70.33; 35.88) |  | 51.79 (48.32 – 54.11; 4.16) |  | 60.27 (51.17 – 68.78; 14.08) |  | 48.31 (46.56 – 50.07; 5.2) |  | 36.14 (36.14 – 36.14; 0) |  | 49.83 (47.65 – 61.51; 13.7) |  | 51.65 (46.92 – 56.01; 7.02) |  | 52.52 (51.37 – 54.08; 2.31) |  |
| **% time COx_L > 0.2** | 36.76 (26.48 – 47.05; 30.5) | 0.6667 | 27.13 (22.2 – 30.28; 6.93) | 0.982 | 34.75 (26.52 – 40.78; 9.87) | 0.2995 | 24.98 (24.25 – 25.72; 2.18) | 0.6667 | 12.09 (12.09 – 12.09; 0) | 1 | 26.54 (25.35 – 30.89; 5.75) | 0.6905 | 26.96 (24.07 – 41.33; 9.84) | 0.9197 | 29 (25.93 – 32.79; 5.4) | 1 |
| **% time COx_R > 0.2** | 39.29 (28.57 – 50.01; 31.79) |  | 27.16 (23.28 – 35.03; 8.25) |  | 38.45 (26.75 – 45.37; 14.35) |  | 27.06 (25.7 – 28.41; 4.03) |  | 15.49 (15.49 – 15.49; 0) |  | 23.52 (22.19 – 31.38; 6.3) |  | 28.79 (22.38 – 31.65; 9.5) |  | 27.95 (27.29 – 32.79; 7.18) |  |
| **% time COx_L > 0.3** | 29.53 (20.49 – 38.57; 26.81) | 0.6667 | 17.35 (13.82 – 20.82; 5.89) | 0.7688 | 24.36 (17.75 – 28.79; 8.36) | 0.3335 | 17.13 (16.32 – 17.94; 2.4) | 0.6667 | 7.71 (7.71 – 7.71; 0) | 1 | 16.18 (15.82 – 21.01; 2.09) | 0.5476 | 19.6 (15.17 – 28.16; 9.77) | 0.9598 | 18.7 (17.37 – 24.68; 4.15) | 0.8413 |
| **% time COx_R > 0.3** | 30.6 (21.66 – 39.53; 26.49) |  | 18.24 (15.29 – 23.79; 7.98) |  | 28.69 (18.43 – 35.59; 12.04) |  | 18.84 (17.4 – 20.27; 4.25) |  | 9.68 (9.68 – 9.68; 0) |  | 15.01 (13.71 – 20.5; 2.74) |  | 19.38 (14 – 23.4; 7.97) |  | 18.6 (17.17 – 24.72; 9.08) |  |
| **% time COx-a_L > 0** | 66.2 (60.22 – 72.18; 17.72) | 1 | 54.5 (48.42 – 60.47; 9.5) | 0.8743 | 63.22 (55.02 – 69.5; 10.36) | 0.6337 | 56.87 (52.26 – 61.47; 13.65) | 0.6667 | 67.21 (67.21 – 67.21; 0) | 1 | 59.05 (53.4 – 65.13; 8.78) | 1 | 64.99 (58.84 – 68.49; 9.12) | 0.4483 | 61.71 (61.04 – 62.2; 1) | 0.6905 |
| **% time COx-a_R > 0** | 68.26 (60.05 – 76.46; 24.33) |  | 54.33 (52.18 – 60.14; 8.23) |  | 64.1 (57.88 – 71.16; 10.44) |  | 51.53 (49.39 – 53.67; 6.35) |  | 71.63 (71.63 – 71.63; 0) |  | 53.82 (53.82 – 67.2; 2.71) |  | 61.03 (55.56 – 65.65; 8.11) |  | 58.17 (55.05 – 64.07; 7.69) |  |
| **% time COx-a_L > 0.2** | 45.27 (37.7 – 52.84; 22.45) | 1 | 28.18 (23.6 – 34.83; 9.85) | 0.5714 | 37.48 (29.18 – 43.32; 9.91) | 0.5073 | 33.38 (30.32 – 36.43; 9.05) | 0.6667 | 37.44 (37.44 – 37.44; 0) | 1 | 34.71 (28.5 – 37.07; 9.21) | 1 | 38.33 (33.39 – 45.76; 9.77) | 0.5788 | 33.44 (33.44 – 33.57; 0.2) | 0.4206 |
| **% time COx-a_R > 0.2** | 46.03 (37.33 – 54.74; 25.81) |  | 30.16 (26.52 – 38.36; 8.72) |  | 39.7 (30.43 – 46.71; 11.85) |  | 27.98 (27.07 – 28.9; 2.71) |  | 41.56 (41.56 – 41.56; 0) |  | 28.98 (27.57 – 36.93; 7.24) |  | 37.46 (29.49 – 41.68; 11.79) |  | 31.66 (31.31 – 36.99; 6.03) |  |
| **% time COx-a_L > 0.3** | 35.92 (28.88 – 42.96; 20.87) | 0.6667 | 18.62 (13.77 – 23.74; 8.1) | 0.4544 | 27.05 (21.23 – 30.71; 7.86) | 0.4779 | 22.4 (20.93 – 23.87; 4.35) | 0.3333 | 23.29 (23.29 – 23.29; 0) | 1 | 23.93 (19.73 – 24.61; 6.22) | 1 | 25.77 (24.54 – 31.6; 7.01) | 0.6866 | 22.25 (21.48 – 23.85; 2.38) | 0.8413 |
| **% time COx-a_R > 0.3** | 34.34 (26.75 – 41.93; 22.52) |  | 19.56 (16.63 – 25.86; 6.37) |  | 29.1 (21.36 – 35.3; 10.82) |  | 18.3 (18.03 – 18.56; 0.77) |  | 26.48 (26.48 – 26.48; 0) |  | 19.97 (19.3 – 23.95; 5.9) |  | 26.04 (19.74 – 30.56; 9.34) |  | 22.74 (20.04 – 25.1; 4) |  |
| **5-Minute Data Resolution** | | | | | | | | | | | | | | | | |
| **ABP (mmHg)** | 86.8 (80.83 – 92.94) | – | 79.45 (74.15 – 92.4) | – | 80.42 (75.99 – 86.57) | – | 83.49 (77.55 – 90.61) | – | 78.06 (74.86 – 82.67) | – | 80.5 (73.39 – 87.75) | – | 83.74 (77.29 – 89.72) | – | 90.83 (84.64 – 99.16) | – |
| **CPP (mmHg)** | 83.21 (75.95 – 91.24) |  | 78.33 (69.8 – 88) |  | 71.74 (65.82 – 78.33) |  | 79.11 (70.62 – 85.52) |  | 66.2 (62.87 – 70.03) |  | 72.62 (64.84 – 78.46) |  | 73.02 (67.73 – 77.54) |  | 75.18 (67.43 – 83.54) |  |
| **rSO2_L (%)** | 64.88 (59.84 – 70.08) | 1 | 72.5 (68.94 – 76.02) | 0.9459 | 68.64 (65.47 – 74.95) | 0.7187 | 70.75 (66.47 – 74.41) | 1 | 76.96 (72.36 – 81.54) | 1 | 68.23 (63.86 – 70.8) | 1 | 71.58 (69.07 – 75.36) | 0.8798 | 66.01 (62.63 – 67.99) | 0.3095 |
| **rSO2_R (%)** | 59.25 (57.1 – 62.39) |  | 71.14 (67.65 – 73.55) |  | 69.11 (65.64 – 75.45) |  | 70.93 (66.76 – 73.92) |  | 87 (76.52 – 90.15) |  | 68.34 (65.99 – 71.02) |  | 67.23 (63.11 – 73.59) |  | 66.75 (63.99 – 69.06) |  |
| **COx_L (au)** | 0.05 (-0.17 – 0.25) | 1 | 0.01 (-0.16 – 0.18) | 0.8388 | 0.06 (-0.1 – 0.25) | 0.2995 | -0.05 (-0.24 – 0.16) | 0.6667 | -0.16 (-0.3 – 0) | 1 | 0.02 (-0.16 – 0.18) | 0.6905 | 0.01 (-0.14 – 0.2) | 0.9598 | 0.03 (-0.14 – 0.2) | 0.6905 |
| **COx_R (au)** | 0.07 (-0.11 – 0.28) |  | 0 (-0.16 – 0.2) |  | 0.1 (-0.08 – 0.29) |  | -0.03 (-0.2 – 0.16) |  | -0.1 (-0.26 – 0.06) |  | -0.01 (-0.17 – 0.15) |  | 0.02 (-0.18 – 0.22) |  | 0.02 (-0.15 – 0.19) |  |
| **COx-a_L (au)** | 0.16 (-0.07 – 0.35) | 1 | 0.05 (-0.13 – 0.19) | 0.9459 | 0.1 (-0.05 – 0.28) | 0.6009 | 0.05 (-0.15 – 0.24) | 0.6667 | 0.1 (-0.04 – 0.25) | 1 | 0.07 (-0.12 – 0.24) | 0.8413 | 0.12 (-0.05 – 0.27) | 0.5114 | 0.08 (-0.07 – 0.24) | 0.6905 |
| **COx-a_R (au)** | 0.17 (-0.01 – 0.35) |  | 0.03 (-0.14 – 0.21) |  | 0.12 (-0.05 – 0.3) |  | 0.01 (-0.19 – 0.19) |  | 0.14 (0 – 0.28) |  | 0.03 (-0.13 – 0.21) |  | 0.1 (-0.1 – 0.28) |  | 0.06 (-0.11 – 0.22) |  |
| **MAD of ABP (mmHg)** | 8.84 (7.5 – 10.18) | – | 11.01 (6.94 – 12.86) | – | 8.58 (6.86 – 9.36) | – | 9.64 (9.55 – 9.73) | – | 5.55 (5.55 – 5.55) | – | 9.74 (7.3 – 11.25) | – | 7.76 (6.31 – 9.7) | – | 10.32 (9.9 – 12.03) | – |
| **MAD of CPP (mmHg)** | 11.08 (8.64 – 13.52) |  | 10.97 (6.32 – 12.59) |  | 7.93 (6.45 – 9.56) |  | 11.39 (9.68 – 13.1) |  | 5.37 (5.37 – 5.37) |  | 8.27 (7.21 – 9.55) |  | 8.03 (6.53 – 9.84) |  | 11.36 (10.98 – 12.04) |  |
| **MAD of rSO2_L (%)** | 7.59 (5.27 – 9.9) | 0.6667 | 5.54 (3.66 – 6.03) | 0.0767 | 4.54 (3.15 – 6.86) | 0.3453 | 5.6 (4.3 – 6.89) | 1 | 6.82 (6.82 – 6.82) | 1 | 5.15 (2.83 – 9.83) | 0.4206 | 4.11 (3.56 – 6.69) | 0.3358 | 5.28 (2.93 – 5.96) | 0.5476 |
| **MAD of rSO2_R (%)** | 3.56 (2.86 – 4.27) |  | 3.81 (2.62 – 4.53) |  | 5.74 (3.87 – 7.86) |  | 5.24 (4.21 – 6.27) |  | 6.98 (6.98 – 6.98) |  | 4.28 (2.65 – 4.42) |  | 5.97 (4.12 – 7.9) |  | 6.13 (3.72 – 10.03) |  |
| **MAD of COx_L (au)** | 0.31 (0.29 – 0.33) | 1 | 0.27 (0.22 – 0.31) | 0.4013 | 0.26 (0.24 – 0.29) | 0.5224 | 0.29 (0.28 – 0.3) | 0.6667 | 0.22 (0.22 – 0.22) | 1 | 0.28 (0.26 – 0.31) | 0.8413 | 0.26 (0.25 – 0.32) | 0.6498 | 0.25 (0.24 – 0.25) | 0.6905 |
| **MAD of COx_R (au)** | 0.3 (0.29 – 0.3) |  | 0.28 (0.24 – 0.32) |  | 0.28 (0.23 – 0.3) |  | 0.27 (0.26 – 0.28) |  | 0.24 (0.24 – 0.24) |  | 0.29 (0.27 – 0.29) |  | 0.3 (0.26 – 0.31) |  | 0.25 (0.25 – 0.26) |  |
| **MAD of COx-a_L (au)** | 0.31 (0.3 – 0.33) | 0.3333 | 0.25 (0.23 – 0.28) | 0.982 | 0.25 (0.24 – 0.27) | 0.5688 | 0.29 (0.27 – 0.3) | 0.6667 | 0.22 (0.22 – 0.22) | 1 | 0.26 (0.25 – 0.27) | 0.8413 | 0.24 (0.22 – 0.29) | 0.4483 | 0.24 (0.23 – 0.25) | 0.5476 |
| **MAD of COx-a_R (au)** | 0.27 (0.26 – 0.27) |  | 0.25 (0.23 – 0.28) |  | 0.26 (0.23 – 0.28) |  | 0.27 (0.26 – 0.29) |  | 0.21 (0.21 – 0.21) |  | 0.26 (0.22 – 0.26) |  | 0.27 (0.24 – 0.28) |  | 0.26 (0.23 – 0.26) |  |
| **% time rSO2_L > 60%** | 40.7 (20.35 – 61.04; 60.34) | 1 | 97.54 (90.9 – 99.91; 3.65) | 0.6403 | 85.94 (37.87 – 99.1; 20.8) | 0.2643 | 84.27 (77.21 – 91.32; 20.91) | 1 | 96.37 (96.37 – 96.37; 0) | – | 93.05 (52.05 – 100; 10.31) | 1 | 92.51 (73.38 – 98.17; 10.77) | 0.5724 | 89.48 (86.19 – 94.26; 7.09) | 0.8413 |
| **% time rSO2_R > 60%** | 47.79 (23.9 – 71.69; 70.85) |  | 98.81 (92.98 – 100; 1.77) |  | 94.71 (54.47 – 99.83; 7.85) |  | 81.34 (72.01 – 90.67; 27.67) |  | 96.37 (96.37 – 96.37; 0) |  | 90.41 (68.47 – 100; 14.21) |  | 85.48 (61.94 – 95.7; 20.93) |  | 90.23 (88.68 – 94.31; 6.04) |  |
| **% time rSO2_L > 70%** | 36.68 (18.34 – 55.01; 54.38) | 1 | 70.81 (29.2 – 78.16; 35.97) | 0.8902 | 40.8 (3.95 – 85.8; 58.13) | 0.7962 | 62.09 (44.35 – 79.82; 52.59) | 0.6667 | 91.93 (91.93 – 91.93; 0) | 1 | 30.46 (5.74 – 70.08; 44.77) | 0.8413 | 66.33 (9.66 – 85.1; 36.19) | 0.9591 | 9.77 (5.41 – 23.57; 7.59) | 0.3095 |
| **% time rSO2_R > 70%** | 45.94 (22.97 – 68.91; 68.11) |  | 62.35 (9.77 – 97.56; 55.82) |  | 44.31 (32.21 – 72.66; 41.43) |  | 53.57 (32.07 – 75.08; 63.77) |  | 95.91 (95.91 – 95.91; 0) |  | 33.69 (2.35 – 34.56; 46.46) |  | 35.25 (33.39 – 72.7; 39.4) |  | 21.17 (18.08 – 41.23; 9.28) |  |
| **% time rSO2_L > 80%** | 20.33 (10.16 – 30.49; 30.13) | 1 | 11.06 (0.27 – 32.57; 16.4) | 0.7959 | 1.7 (0 – 22.05; 2.52) | 0.7838 | 7.11 (4.16 – 10.06; 8.75) | 1 | 33.57 (33.57 – 33.57; 0) | 1 | 0.26 (0 – 2.22; 0.39) | 1 | 6.03 (0.39 – 24.91; 8.95) | 0.8369 | 1.1 (0 – 1.55; 1.64) | 0.8325 |
| **% time rSO2_R > 80%** | 19.07 (9.54 – 28.61; 28.28) |  | 2.84 (0 – 37.26; 4.21) |  | 7.19 (0.05 – 16.55; 10.66) |  | 10.73 (5.37 – 16.1; 15.91) |  | 66.08 (66.08 – 66.08; 0) |  | 0.79 (0 – 1.99; 1.17) |  | 6.48 (0.17 – 24.64; 9.43) |  | 1.07 (0.82 – 2.15; 1.58) |  |
| **% time rSO2_L > 90%** | 9.08 (4.54 – 13.62; 13.46) | 1 | 0 (0 – 0; 0) | 0.9699 | 0 (0 – 0.94; 0) | 0.6828 | 0 (0 – 0; 0) | – | 0 (0 – 0; 0) | 1 | 0 (0 – 0; 0) | 0.4237 | 0 (0 – 0.04; 0) | 1 | 0 (0 – 0; 0) | 1 |
| **% time rSO2_R > 90%** | 0.5 (0.25 – 0.74; 0.74) |  | 0 (0 – 0; 0) |  | 0 (0 – 0.38; 0) |  | 0 (0 – 0; 0) |  | 25.73 (25.73 – 25.73; 0) |  | 0 (0 – 0; 0) |  | 0 (0 – 0.51; 0) |  | 0 (0 – 0; 0) |  |
| **% time COx_L > 0** | 51.72 (39.36 – 64.07; 36.64) | 0.6667 | 51.81 (47.17 – 56.16; 7.58) | 0.8036 | 58.6 (52.35 – 67.58; 11.58) | 0.511 | 42.63 (40.24 – 45.02; 7.09) | 0.6667 | 26.1 (26.1 – 26.1; 0) | 1 | 54.47 (54.03 – 58.21; 5.55) | 0.6905 | 52.92 (46.72 – 68.06; 10.81) | 0.801 | 56.17 (52.3 – 56.64; 5.74) | 0.6905 |
| **% time COx_R > 0** | 60.5 (46.56 – 74.43; 41.33) |  | 50.79 (45.73 – 55.65; 7.73) |  | 62.5 (54.17 – 70.87; 12.67) |  | 46.75 (44.21 – 49.29; 7.54) |  | 33.54 (33.54 – 33.54; 0) |  | 47.89 (47.55 – 61.67; 13.19) |  | 51.38 (46.83 – 57.34; 8.83) |  | 52.62 (52.32 – 54.72; 3.11) |  |
| **% time COx_L > 0.2** | 35.68 (24.59 – 46.77; 32.88) | 1 | 23.51 (17.29 – 28.47; 9.25) | 0.7006 | 30.67 (24.19 – 39.5; 13) | 0.3105 | 20.48 (18.65 – 22.31; 5.42) | 0.6667 | 8.41 (8.41 – 8.41; 0) | 1 | 23.88 (21.36 – 27.01; 4.65) | 0.4206 | 24.55 (20.87 – 39.81; 10.81) | 0.801 | 24.82 (22.98 – 29.18; 6.17) | 0.8413 |
| **% time COx_R > 0.2** | 36.38 (24.34 – 48.42; 35.71) |  | 24.65 (19.65 – 32.48; 8.56) |  | 36.78 (23.36 – 45.24; 15.37) |  | 23.58 (22.02 – 25.14; 4.63) |  | 11.74 (11.74 – 11.74; 0) |  | 18.74 (18.41 – 30; 2.18) |  | 25.92 (19.34 – 28.12; 9.74) |  | 23.51 (23.25 – 29.04; 8.2) |  |
| **% time COx_L > 0.3** | 27.94 (17.62 – 38.25; 30.6) | 0.6667 | 14.31 (9.88 – 17.61; 7.08) | 0.9633 | 19.28 (15.55 – 26.73; 10.01) | 0.3824 | 11.46 (11.12 – 11.81; 1.02) | 0.3333 | 5 (5 – 5; 0) | 1 | 13.3 (11.45 – 16.39; 4.23) | 0.8413 | 15.09 (11.34 – 25.93; 7.77) | 0.9598 | 14.74 (13.79 – 20.91; 2.46) | 0.5476 |
| **% time COx_R > 0.3** | 28.43 (18.23 – 38.63; 30.26) |  | 13.39 (10.68 – 18.28; 6.9) |  | 25.39 (13.24 – 32.4; 12.28) |  | 16.5 (15.07 – 17.94; 4.25) |  | 7.14 (7.14 – 7.14; 0) |  | 10.54 (10.45 – 23.33; 1.47) |  | 15.77 (10.56 – 18.81; 7.73) |  | 13.7 (13.35 – 19.64; 8.8) |  |
| **% time COx-a_L > 0** | 66.08 (60.19 – 71.97; 17.47) | 1 | 57.2 (49.06 – 63.58; 12.05) | 0.7688 | 66.44 (56.69 – 71.82; 9.06) | 0.4779 | 57.23 (52.51 – 61.95; 14) | 0.6667 | 67.68 (67.68 – 67.68; 0) | 1 | 60.33 (55.01 – 66.12; 8.58) | 1 | 69.28 (60.42 – 70.81; 12.01) | 0.3622 | 63.45 (63.29 – 64.05; 0.88) | 0.8413 |
| **% time COx-a_R > 0** | 71.04 (61.8 – 80.29; 27.42) |  | 55.31 (53.38 – 61.62; 8.04) |  | 66.66 (59.26 – 74.46; 12.26) |  | 49.76 (47.42 – 52.11; 6.96) |  | 74.58 (74.58 – 74.58; 0) |  | 54.27 (54.18 – 70.65; 1.67) |  | 64.91 (56.24 – 66.73; 12.86) |  | 59.33 (56.6 – 66.77; 7.56) |  |
| **% time COx-a_L > 0.2** | 44.99 (35.69 – 54.28; 27.55) | 1 | 24.65 (18.63 – 32.38; 11.3) | 0.6027 | 34.95 (24.87 – 41.42; 10.75) | 0.5531 | 30.31 (27.76 – 32.85; 7.54) | 0.6667 | 33.05 (33.05 – 33.05; 0) | 1 | 30.3 (26.65 – 36.12; 8.62) | 0.8413 | 35.91 (29.89 – 45.21; 11.43) | 0.5788 | 29.85 (29.53 – 30.57; 0.52) | 0.4206 |
| **% time COx-a_R > 0.2** | 44.46 (34.42 – 54.5; 29.76) |  | 26.03 (22.16 – 34.27; 7.51) |  | 38.04 (27.08 – 45.91; 14.79) |  | 23.79 (22.81 – 24.76; 2.9) |  | 38.62 (38.62 – 38.62; 0) |  | 26.22 (25.07 – 36.24; 10.9) |  | 34.47 (26.58 – 38.94; 11.7) |  | 27.64 (26.76 – 33.48; 3.9) |  |
| **% time COx-a_L > 0.3** | 33.38 (25.78 – 40.97; 22.53) | 1 | 15.08 (9.88 – 20.19; 8.88) | 0.9268 | 22.95 (16.6 – 26.79; 8.04) | 0.4219 | 18.44 (16.96 – 19.92; 4.38) | 0.6667 | 18.17 (18.17 – 18.17; 0) | 1 | 20.02 (16.45 – 20.83; 5.3) | 0.8413 | 22.21 (19.62 – 28.14; 6.2) | 0.6866 | 17.9 (17.19 – 19.06; 1.58) | 0.8413 |
| **% time COx-a_R > 0.3** | 33.14 (24.13 – 42.15; 26.72) |  | 15.57 (12.51 – 17.78; 4.22) |  | 25.25 (17.5 – 33.41; 12.32) |  | 13.91 (12.94 – 14.87; 2.87) |  | 21.31 (21.31 – 21.31; 0) |  | 17.68 (14.97 – 20.4; 4.03) |  | 22.88 (16.11 – 25.77; 10.03) |  | 17.36 (16.35 – 20.4; 4.5) |  |
| *The p-values in the table are derived using Mann-Whitney U test between the bilateral signals.*  *ABP, arterial blood pressure; au, arbitrary units; CPP, cerebral perfusion pressure; COx, cerebral oximetry index with CPP; COx-a, cerebral oximetry index with ABP; CT, computed tomography; DAI, diffuse axonal injury; EDH, epidural hematoma; MAD, median absolute deviation; IQR, interquartile range; mmHg, millimeters of mercury; rSO2, regional cerebral oxygen saturation; tSAH, traumatic subarachnoid hemorrhage; SDH, subdural hematoma; aSDH, acute subdural hematoma; TBI-GLR, traumatic brain injury patient group without bifrontal lobe pathology.* | | | | | | | | | | | | | | | | |

File S8j: Sub-grouped Physiologic Results using 10-Second, 1-Minute, and 5-Minute Data Resolutions for TBI-GL Population

| **Physiologic Variable** | **Sub-groups** | | | | | | | | | | | | | | | | | |
| --- | --- | --- | --- | --- | --- | --- | --- | --- | --- | --- | --- | --- | --- | --- | --- | --- | --- | --- |
|  | **Age < 40 [n = 5]** | | **Age 40 – 60 [n = 6]** | | **Age > 60 [n = 4]** | | **Males [n = 14]** | | **Females [n = 1]** | | **Focal Injury (aSDH, SDH, EDH, or Contusion) [n = 14]** | | **Diffuse Injury (DAI or tSAH) [n = 1]** | | **Marshall CT V [n = 8]** | | **Marshall CT IV [n = 5]** | |
|  | **Median (IQR) or  Median (IQR; MAD)** | **p-value** | **Median (IQR) or  Median (IQR; MAD)** | **p-value** | **Median (IQR) or  Median (IQR; MAD)** | **p-value** | **Median (IQR) or  Median (IQR; MAD)** | **p-value** | **Median (IQR) or  Median (IQR; MAD)** | **p-value** | **Median (IQR) or  Median (IQR; MAD)** | **p-value** | **Median (IQR) or  Median (IQR; MAD)** | **p-value** | **Median (IQR) or  Median (IQR; MAD)** | **p-value** | **Median (IQR) or  Median (IQR; MAD)** | **p-value** |
| **10-Second Data Resolution** | | | | | | | | | | | | | | | | | | |
| **ABP (mmHg)** | 81.08 (76.01 – 87.6) | – | 87.32 (79.66 – 95.79) | – | 81.52 (74.93 – 89.55) | – | 83.33 (76.77 – 91.57) | – | 79.86 (70.24 – 86.45) | – | 82.14 (76.3 – 89.55) | – | 90.5 (83.15 – 99.84) | – | 84.75 (77.73 – 91.79) | – | 81.21 (75.41 – 88.34) | – |
| **CPP (mmHg)** | 72.05 (66.2 – 77.45) |  | 78.04 (70.76 – 85.9) |  | 71.84 (67.34 – 80.4) |  | 73.23 (67.94 – 81.59) |  | 73.54 (68.94 – 80.1) |  | 73.23 (67.94 – 80.25) |  | 79.51 (72.09 – 89.18) |  | 76.47 (69.29 – 84.38) |  | 72.05 (66.2 – 78.03) |  |
| **rSO2_L (%)** | 66.99 (60.99 – 74) | 0.7533 | 67.14 (61.87 – 71.49) | 0.4696 | 63.38 (56.44 – 65.99) | 0.4857 | 66.88 (60.99 – 71.49) | 0.4906 | 74 (69 – 76.99) | 1 | 66.88 (60.99 – 73) | 0.6791 | 67.5 (65 – 70.99) | 1 | 71.99 (65.87 – 75) | 0.8785 | 63.99 (60.99 – 67.99) | 0.2222 |
| **rSO2_R (%)** | 70.99 (68.94 – 76.99) |  | 65.23 (62.84 – 70.35) |  | 67.81 (59.75 – 77.58) |  | 70.14 (65.29 – 75.01) |  | 60.99 (56.5 – 62.74) |  | 70.14 (65.29 – 75.01) |  | 63.93 (61.61 – 66.99) |  | 71.52 (66.17 – 78.49) |  | 69.35 (66.51 – 71.71) |  |
| **COx_L (au)** | 0 (-0.18 – 0.19) | 0.8294 | 0 (-0.22 – 0.25) | 1 | 0.01 (-0.1 – 0.26) | 1 | 0 (-0.14 – 0.25) | 0.7959 | 0 (-0.22 – 0.22) | 1 | 0 (-0.14 – 0.25) | 0.9433 | -0.13 (-0.32 – 0.12) | 1 | 0 (-0.17 – 0.24) | 0.7875 | 0.02 (-0.09 – 0.27) | 0.6723 |
| **COx_R (au)** | 0.01 (-0.18 – 0.22) |  | 0 (-0.09 – 0.18) |  | 0.05 (-0.03 – 0.28) |  | 0 (-0.14 – 0.22) |  | 0.11 (-0.09 – 0.3) |  | 0.01 (-0.1 – 0.22) |  | -0.16 (-0.36 – 0.07) |  | 0 (-0.13 – 0.22) |  | 0.03 (-0.07 – 0.22) |  |
| **COx-a_L (au)** | 0.02 (-0.15 – 0.25) | 0.3976 | 0.08 (-0.12 – 0.33) | 0.9357 | 0.01 (-0.07 – 0.27) | 1 | 0.03 (-0.1 – 0.27) | 0.2932 | 0.05 (-0.15 – 0.29) | 1 | 0.03 (-0.12 – 0.27) | 0.3382 | 0.12 (-0.1 – 0.33) | 1 | 0.04 (-0.14 – 0.28) | 0.2202 | 0.02 (-0.08 – 0.27) | 0.9166 |
| **COx-a_R (au)** | 0.01 (-0.16 – 0.22) |  | 0.05 (-0.05 – 0.27) |  | 0.05 (-0.03 – 0.27) |  | 0.01 (-0.09 – 0.22) |  | 0.17 (-0.04 – 0.37) |  | 0.01 (-0.09 – 0.22) |  | 0.16 (-0.04 – 0.35) |  | 0 (-0.12 – 0.22) |  | 0.03 (-0.06 – 0.22) |  |
| **MAD of ABP (mmHg)** | 7.9 (7.75 – 8.09) | – | 11.88 (11.51 – 12.36) | – | 8.67 (6.59 – 10.8) | – | 9.71 (7.79 – 11.78) | – | 11.85 (11.85 – 11.85) | – | 9.71 (7.79 – 11.73) | – | 11.91 (11.91 – 11.91) | – | 10.49 (7.71 – 11.51) | – | 9.21 (8.09 – 12.57) | – |
| **MAD of CPP (mmHg)** | 7.49 (7.31 – 9.47) |  | 11.72 (10.57 – 12.52) |  | 9.16 (6.9 – 11.18) |  | 10.43 (7.35 – 11.35) |  | 7.86 (7.86 – 7.86) |  | 9.93 (7.35 – 11.14) |  | 12.29 (12.29 – 12.29) |  | 9.12 (7.44 – 11.12) |  | 10.48 (7.22 – 11.42) |  |
| **MAD of rSO2_L (%)** | 7.43 (4.45 – 7.53) | 0.5476 | 4.8 (4.37 – 6.6) | 0.132 | 5 (3.95 – 6.95) | 1 | 5 (4.37 – 7.51) | 0.2231 | 5.14 (5.14 – 5.14) | 1 | 5.35 (4.45 – 7.51) | 0.2231 | 4.34 (4.34 – 4.34) | 1 | 6.12 (4.08 – 8.42) | 0.2345 | 4.45 (4.45 – 5.56) | 0.834 |
| **MAD of rSO2_R (%)** | 3.97 (3.83 – 5.93) |  | 3.17 (2.53 – 3.69) |  | 6.22 (3.22 – 11.91) |  | 3.83 (3.03 – 6.85) |  | 3.29 (3.29 – 3.29) |  | 3.68 (3.03 – 6.85) |  | 3.83 (3.83 – 3.83) |  | 3.42 (2.88 – 4.46) |  | 3.83 (3.02 – 7.16) |  |
| **MAD of COx_L (au)** | 0.28 (0.27 – 0.32) | 1 | 0.33 (0.32 – 0.37) | 0.0649 | 0.25 (0.25 – 0.3) | 0.8857 | 0.3 (0.26 – 0.33) | 0.3761 | 0.33 (0.33 – 0.33) | 1 | 0.3 (0.26 – 0.33) | 0.3761 | 0.32 (0.32 – 0.32) | 1 | 0.33 (0.26 – 0.34) | 0.3823 | 0.28 (0.28 – 0.32) | 0.5476 |
| **MAD of COx_R (au)** | 0.28 (0.27 – 0.28) |  | 0.3 (0.19 – 0.31) |  | 0.28 (0.25 – 0.32) |  | 0.28 (0.26 – 0.31) |  | 0.29 (0.29 – 0.29) |  | 0.28 (0.26 – 0.31) |  | 0.31 (0.31 – 0.31) |  | 0.3 (0.26 – 0.31) |  | 0.26 (0.25 – 0.28) |  |
| **MAD of COx-a_L (au)** | 0.28 (0.27 – 0.29) | 0.8413 | 0.33 (0.32 – 0.36) | 0.0649 | 0.26 (0.24 – 0.31) | 1 | 0.28 (0.27 – 0.33) | 0.1781 | 0.32 (0.32 – 0.32) | 1 | 0.28 (0.27 – 0.33) | 0.1781 | 0.32 (0.32 – 0.32) | 1 | 0.32 (0.28 – 0.35) | 0.3282 | 0.27 (0.27 – 0.28) | 0.2222 |
| **MAD of COx-a_R (au)** | 0.25 (0.25 – 0.29) |  | 0.3 (0.19 – 0.31) |  | 0.27 (0.24 – 0.29) |  | 0.27 (0.24 – 0.3) |  | 0.3 (0.3 – 0.3) |  | 0.27 (0.24 – 0.3) |  | 0.29 (0.29 – 0.29) |  | 0.3 (0.26 – 0.31) |  | 0.25 (0.25 – 0.26) |  |
| **% time rSO2_L > 60%** | 80.07 (79.38 – 91.04; 9.45) | 0.3095 | 80.82 (74.71 – 95.82; 18.49) | 0.9372 | 58.81 (29.86 – 87.9; 48.48) | 0.4857 | 79.73 (73.84 – 89.69; 12.86) | 0.1499 | 99.96 (99.96 – 99.96; 0) | 1 | 79.73 (73.84 – 89.69; 12.86) | 0.3519 | 99.22 (99.22 – 99.22; 0) | 1 | 88.34 (75.57 – 99.63; 17.11) | 0.7209 | 79.38 (56.78 – 80.07; 6.86) | 0.0952 |
| **% time rSO2_R > 60%** | 99.78 (98.57 – 99.95; 0.33) |  | 85.42 (62.26 – 92.64; 17.26) |  | 83.07 (68.11 – 96.95; 21.92) |  | 95.1 (75.33 – 99.75; 7.26) |  | 55.45 (55.45 – 55.45; 0) |  | 95.1 (70.78 – 99.75; 7.26) |  | 88.14 (88.14 – 88.14; 0) |  | 96.89 (66.42 – 99.83; 4.6) |  | 96.06 (82.7 – 98.57; 5.78) |  |
| **% time rSO2_L > 70%** | 34.32 (33.91 – 62.97; 33.93) | 0.3095 | 32.58 (16.74 – 55.18; 36.71) | 0.6991 | 16.28 (1.95 – 41.33; 22.22) | 0.4857 | 31.95 (11.65 – 55.18; 36.97) | 0.21 | 68.81 (68.81 – 68.81; 0) | 1 | 34.12 (11.65 – 62.69; 41.95) | 0.4081 | 29.99 (29.99 – 29.99; 0) | 1 | 62.41 (27.02 – 70.46; 21.52) | 0.8785 | 12.32 (11.43 – 29.97; 18.27) | 0.2222 |
| **% time rSO2_R > 70%** | 59.12 (42.16 – 78.27; 28.4) |  | 27.6 (7.89 – 51.77; 33.89) |  | 39.38 (19.17 – 65.73; 37.59) |  | 50.11 (26.85 – 65.19; 31.8) |  | 0 (0 – 0; 0) |  | 50.11 (26.85 – 65.19; 31.8) |  | 8.42 (8.42 – 8.42; 0) |  | 53.94 (37.01 – 80.31; 42.1) |  | 42.16 (24.31 – 59.12; 26.47) |  |
| **% time rSO2_L > 80%** | 6.63 (0.81 – 17.3; 9.59) | 0.8413 | 0.42 (0.23 – 0.81; 0.48) | 0.468 | 0.33 (0.24 – 0.43; 0.25) | 0.3836 | 0.61 (0.31 – 5.2; 0.56) | 0.9084 | 0.2 (0.2 – 0.2; 0) | 1 | 0.61 (0.23 – 5.2; 0.64) | 0.9449 | 0.31 (0.31 – 0.31; 0) | 1 | 0.44 (0.29 – 11.58; 0.45) | 0.8745 | 0.69 (0.16 – 0.9; 0.79) | 0.7533 |
| **% time rSO2_R > 80%** | 6.2 (0.17 – 19.1; 9.17) |  | 0.04 (0 – 5.53; 0.06) |  | 18.2 (0.49 – 50.49; 26.5) |  | 3.43 (0.04 – 24.28; 5.08) |  | 0 (0 – 0; 0) |  | 3.43 (0.04 – 24.28; 5.08) |  | 0 (0 – 0; 0) |  | 12.65 (0.06 – 37.55; 18.76) |  | 0.17 (0.02 – 0.65; 0.25) |  |
| **% time rSO2_L > 90%** | 0.02 (0 – 0.06; 0.02) | 0.6558 | 0 (0 – 0.03; 0) | 0.9241 | 0 (0 – 0; 0) | 0.1859 | 0 (0 – 0.03; 0) | 0.5146 | 0 (0 – 0; 0) | – | 0 (0 – 0.03; 0) | 0.5146 | 0 (0 – 0; 0) | – | 0 (0 – 1.88; 0) | 0.2516 | 0 (0 – 0.02; 0) | 0.7972 |
| **% time rSO2_R > 90%** | 0 (0 – 0.02; 0) |  | 0 (0 – 0.04; 0) |  | 7.29 (0 – 14.75; 10.81) |  | 0 (0 – 1.23; 0) |  | 0 (0 – 0; 0) |  | 0 (0 – 1.23; 0) |  | 0 (0 – 0; 0) |  | 0.01 (0 – 14.75; 0.01) |  | 0 (0 – 0; 0) |  |
| **% time COx_L > 0** | 50.22 (46.79 – 51.89; 5.05) | 0.8413 | 47.37 (38.66 – 52.98; 13.56) | 0.8182 | 51.11 (48.23 – 55.45; 6.93) | 0.8857 | 50.12 (45.79 – 53.27; 5.81) | 0.8036 | 46.18 (46.18 – 46.18; 0) | 1 | 50.12 (46.33 – 53.27; 5.52) | 0.8388 | 35.75 (35.75 – 35.75; 0) | 1 | 50.12 (45.35 – 52.53; 6.13) | 1 | 52.2 (48.57 – 53.63; 5.39) | 0.8413 |
| **% time COx_R > 0** | 51.96 (46.77 – 52.07; 2.39) |  | 43.09 (32.91 – 56.72; 20.99) |  | 55.43 (48.75 – 64.21; 11.51) |  | 49.04 (45.77 – 53.2; 5.98) |  | 64.36 (64.36 – 64.36; 0) |  | 50.9 (46.62 – 58.05; 7.21) |  | 31.23 (31.23 – 31.23; 0) |  | 49.04 (46.3 – 53.94; 4.87) |  | 53.57 (51.96 – 61.03; 11.06) |  |
| **% time COx_L > 0.2** | 23.77 (23.52 – 29.36; 0.42) | 1 | 28.94 (21.46 – 33.11; 10.49) | 0.5887 | 29.76 (28.14 – 34.78; 3.11) | 1 | 28.99 (23.58 – 31.91; 7.44) | 0.4544 | 26.86 (26.86 – 26.86; 0) | 1 | 28.99 (24.51 – 31.91; 5.96) | 0.6673 | 19.66 (19.66 – 19.66; 0) | 1 | 27.74 (25.91 – 30.48; 4.33) | 0.8785 | 31.01 (30.89 – 32.21; 1.77) | 0.8413 |
| **% time COx_R > 0.2** | 26.89 (25.34 – 27.46; 2.3) |  | 23.34 (17.22 – 33.92; 15.96) |  | 32.61 (27.04 – 42.58; 8.45) |  | 26.76 (23.71 – 28.3; 4.17) |  | 37.08 (37.08 – 37.08; 0) |  | 27.03 (24.66 – 34.95; 4.57) |  | 15.55 (15.55 – 15.55; 0) |  | 27.03 (26.09 – 30.7; 3.07) |  | 27.46 (25.34 – 38.04; 7.72) |  |
| **% time COx_L > 0.3** | 15.99 (14.91 – 21.7; 1.89) | 1 | 20.87 (15.56 – 24.36; 7.88) | 0.5887 | 21.78 (21.11 – 26; 1.65) | 0.8857 | 21.63 (15.18 – 22.68; 6.66) | 0.4544 | 19.21 (19.21 – 19.21; 0) | 1 | 21.63 (16.8 – 22.68; 4.28) | 0.6027 | 14.35 (14.35 – 14.35; 0) | 1 | 20.67 (18.13 – 22.52; 4.28) | 0.7984 | 22.53 (22.01 – 22.73; 0.78) | 0.6905 |
| **% time COx_R > 0.3** | 16.82 (15.75 – 17.69; 1.59) |  | 16.07 (11.88 – 22.91; 10.82) |  | 23.48 (19.15 – 31.92; 6.83) |  | 17.26 (15.79 – 21.32; 3.67) |  | 25.14 (25.14 – 25.14; 0) |  | 18 (15.99 – 24.34; 4.27) |  | 10.53 (10.53 – 10.53; 0) |  | 18.87 (16.67 – 22.74; 4.24) |  | 17.69 (15.91 – 27.52; 2.87) |  |
| **% time COx-a_L > 0** | 52.99 (50.7 – 53.68; 3.39) | 0.6905 | 59.23 (55.48 – 61; 4.92) | 0.9372 | 51.28 (49.43 – 56.32; 3.96) | 1 | 53.34 (50.37 – 59.76; 6.47) | 0.3064 | 54.59 (54.59 – 54.59; 0) | 1 | 53.34 (50.37 – 57.49; 5.18) | 0.3519 | 63.88 (63.88 – 63.88; 0) | 1 | 55.04 (50.59 – 58.69; 6.75) | 0.2345 | 52.99 (52.3 – 53.68; 1.03) | 0.8413 |
| **% time COx-a_R > 0** | 51.96 (46.96 – 52.28; 3.44) |  | 53.56 (40.22 – 68.12; 23.67) |  | 53.97 (47.33 – 62.84; 10.13) |  | 49.73 (46.8 – 58.89; 6.39) |  | 71.22 (71.22 – 71.22; 0) |  | 49.73 (46.8 – 58.89; 6.39) |  | 70.33 (70.33 – 70.33; 0) |  | 47.21 (46.49 – 54.33; 4.67) |  | 54.28 (52.28 – 60.43; 9.13) |  |
| **% time COx-a_L > 0.2** | 29.19 (27.7 – 29.19; 2.21) | 0.1508 | 37.74 (34.45 – 39.37; 4.69) | 0.9372 | 30.7 (30.02 – 35.42; 1.6) | 0.8857 | 31.1 (29.19 – 37.91; 4.19) | 0.062 | 33.47 (33.47 – 33.47; 0) | 1 | 31.1 (29.19 – 36.42; 3.43) | 0.069 | 39.8 (39.8 – 39.8; 0) | 1 | 31.94 (29.19 – 37.57; 4.33) | 0.083 | 31.2 (31.01 – 31.56; 0.54) | 0.5476 |
| **% time COx-a_R > 0.2** | 27.23 (22.86 – 27.44; 1.83) |  | 32.49 (22.38 – 43.38; 16.56) |  | 32.34 (26.51 – 40.44; 9.61) |  | 27.19 (23.15 – 35.26; 7.18) |  | 46.21 (46.21 – 46.21; 0) |  | 27.19 (23.15 – 35.26; 7.18) |  | 44.18 (44.18 – 44.18; 0) |  | 27.19 (24.42 – 31.59; 4.32) |  | 27.44 (22.86 – 37.52; 8.31) |  |
| **% time COx-a_L > 0.3** | 20.2 (18.47 – 21.47; 2.56) | 0.3095 | 27.96 (24.76 – 28.27; 3.33) | 0.6991 | 22.47 (21.76 – 26.8; 1.23) | 0.8857 | 22.51 (21.41 – 28.1; 4.71) | 0.0849 | 23.79 (23.79 – 23.79; 0) | 1 | 22.51 (21.41 – 26.69; 2.66) | 0.0939 | 28.25 (28.25 – 28.25; 0) | 1 | 23.42 (21.45 – 27.82; 3.9) | 0.1304 | 22.5 (21.88 – 22.53; 0.92) | 0.5476 |
| **% time COx-a_R > 0.3** | 17.41 (13.15 – 17.88; 6.32) |  | 23.79 (15.84 – 31.75; 12.18) |  | 23.16 (18.7 – 30.43; 7.35) |  | 17.65 (15.84 – 25.8; 6.42) |  | 32.08 (32.08 – 32.08; 0) |  | 17.65 (15.84 – 25.8; 6.42) |  | 30.96 (30.96 – 30.96; 0) |  | 18.54 (17.07 – 24.36; 3.85) |  | 17.41 (15.58 – 27.14; 6.32) |  |
| **1-Minute Data Resolution** | | | | | | | | | | | | | | | | | | |
| **ABP (mmHg)** | 81.1 (76.24 – 87.52) | – | 87.47 (79.86 – 95.78) | – | 81.54 (75.19 – 89.36) | – | 83.44 (76.92 – 91.46) | – | 79.9 (70.06 – 86.17) | – | 82.23 (76.46 – 89.36) | – | 90.57 (83.2 – 99.9) | – | 84.88 (77.89 – 91.57) | – | 81.34 (75.51 – 88.27) | – |
| **CPP (mmHg)** | 72.18 (66.28 – 77.45) |  | 78.14 (70.93 – 85.8) |  | 71.95 (67.54 – 80.83) |  | 73.3 (68.05 – 82.15) |  | 73.62 (69.13 – 80.1) |  | 73.3 (68.05 – 80.33) |  | 79.56 (72.12 – 89.02) |  | 76.45 (69.65 – 84.16) |  | 72.18 (66.28 – 77.93) |  |
| **rSO2_L (%)** | 66.99 (60.99 – 74.26) | 0.8413 | 67.04 (61.73 – 71.58) | 0.6991 | 63.49 (56.41 – 65.91) | 0.4857 | 66.79 (60.99 – 71.58) | 0.4824 | 73.92 (69 – 76.99) | 1 | 66.79 (60.99 – 73.02) | 0.6347 | 67.48 (65.08 – 71.12) | 1 | 72.07 (65.83 – 75) | 0.9591 | 63.91 (60.99 – 67.81) | 0.2222 |
| **rSO2_R (%)** | 70.99 (68.83 – 76.99) |  | 65.21 (62.97 – 70.34) |  | 67.87 (59.78 – 77.57) |  | 70.03 (65.42 – 75.07) |  | 61.11 (56.57 – 62.66) |  | 70.03 (65.42 – 75.07) |  | 63.8 (61.67 – 66.99) |  | 71.46 (66.32 – 78.41) |  | 69.34 (66.56 – 71.65) |  |
| **COx_L (au)** | 0.02 (-0.19 – 0.2) | 0.8413 | 0.02 (-0.22 – 0.26) | 0.8182 | 0.08 (-0.12 – 0.3) | 0.8857 | 0.05 (-0.17 – 0.29) | 0.8036 | -0.01 (-0.23 – 0.23) | 1 | 0.05 (-0.17 – 0.29) | 0.8036 | -0.13 (-0.32 – 0.12) | 1 | 0.04 (-0.18 – 0.28) | 0.7209 | 0.1 (-0.12 – 0.31) | 1 |
| **COx_R (au)** | 0.01 (-0.17 – 0.21) |  | 0.04 (-0.16 – 0.27) |  | 0.12 (-0.08 – 0.33) |  | 0.03 (-0.16 – 0.25) |  | 0.1 (-0.07 – 0.29) |  | 0.04 (-0.16 – 0.27) |  | -0.16 (-0.35 – 0.06) |  | 0.04 (-0.16 – 0.27) |  | 0.05 (-0.13 – 0.27) |  |
| **COx-a_L (au)** | 0.04 (-0.15 – 0.24) | 0.3095 | 0.1 (-0.12 – 0.32) | 0.6991 | 0.1 (-0.1 – 0.32) | 0.8857 | 0.09 (-0.12 – 0.32) | 0.2852 | 0.08 (-0.16 – 0.29) | 1 | 0.08 (-0.13 – 0.31) | 0.3064 | 0.11 (-0.09 – 0.33) | 1 | 0.08 (-0.14 – 0.32) | 0.3282 | 0.09 (-0.11 – 0.3) | 0.8413 |
| **COx-a_R (au)** | 0.01 (-0.16 – 0.21) |  | 0.1 (-0.12 – 0.3) |  | 0.12 (-0.07 – 0.32) |  | 0.05 (-0.15 – 0.25) |  | 0.18 (-0.03 – 0.37) |  | 0.05 (-0.15 – 0.25) |  | 0.16 (-0.04 – 0.34) |  | 0.04 (-0.17 – 0.26) |  | 0.05 (-0.12 – 0.26) |  |
| **MAD of ABP (mmHg)** | 7.87 (7.67 – 7.97) | – | 11.72 (11.24 – 12.19) | – | 8.47 (6.44 – 10.41) | – | 9.42 (7.72 – 11.67) | – | 11.59 (11.59 – 11.59) | – | 9.42 (7.72 – 11.47) | – | 11.85 (11.85 – 11.85) | – | 10.35 (7.62 – 11.24) | – | 8.99 (7.97 – 12.09) | – |
| **MAD of CPP (mmHg)** | 7.65 (7.25 – 9.26) |  | 11.59 (10.67 – 12.52) |  | 8.76 (6.68 – 10.64) |  | 10.5 (7.35 – 11.08) |  | 8.01 (8.01 – 8.01) |  | 9.87 (7.35 – 10.88) |  | 12.2 (12.2 – 12.2) |  | 9.25 (7.55 – 10.67) |  | 10.51 (7.04 – 11.12) |  |
| **MAD of rSO2_L (%)** | 7.59 (4.73 – 7.72) | 0.4206 | 4.87 (4.36 – 6.6) | 0.132 | 4.96 (3.88 – 6.93) | 1 | 5.17 (4.3 – 7.69) | 0.21 | 5.17 (5.17 – 5.17) | 1 | 5.39 (4.38 – 7.69) | 0.1936 | 4.29 (4.29 – 4.29) | 1 | 6.12 (4.07 – 8.52) | 0.1605 | 4.73 (4.56 – 5.6) | 0.8413 |
| **MAD of rSO2_R (%)** | 3.95 (3.75 – 6.05) |  | 3.12 (2.69 – 3.58) |  | 6.24 (3.25 – 11.87) |  | 3.74 (3.14 – 6.86) |  | 3.14 (3.14 – 3.14) |  | 3.66 (3.12 – 6.86) |  | 3.73 (3.73 – 3.73) |  | 3.35 (2.97 – 4.48) |  | 3.75 (3.21 – 7.13) |  |
| **MAD of COx_L (au)** | 0.31 (0.29 – 0.33) | 0.5476 | 0.34 (0.33 – 0.36) | 0.0649 | 0.33 (0.3 – 0.38) | 0.6857 | 0.32 (0.3 – 0.35) | 0.1251 | 0.34 (0.34 – 0.34) | 1 | 0.33 (0.3 – 0.35) | 0.0767 | 0.31 (0.31 – 0.31) | 1 | 0.34 (0.32 – 0.36) | 0.2345 | 0.33 (0.31 – 0.34) | 0.2222 |
| **MAD of COx_R (au)** | 0.27 (0.27 – 0.29) |  | 0.31 (0.29 – 0.32) |  | 0.31 (0.3 – 0.32) |  | 0.31 (0.29 – 0.32) |  | 0.27 (0.27 – 0.27) |  | 0.3 (0.28 – 0.32) |  | 0.3 (0.3 – 0.3) |  | 0.31 (0.28 – 0.33) |  | 0.29 (0.27 – 0.32) |  |
| **MAD of COx-a_L (au)** | 0.28 (0.28 – 0.3) | 0.2222 | 0.34 (0.33 – 0.37) | **0.026** | 0.32 (0.31 – 0.34) | 0.4857 | 0.32 (0.3 – 0.34) | 0.1251 | 0.34 (0.34 – 0.34) | 1 | 0.32 (0.3 – 0.34) | 0.1371 | 0.31 (0.31 – 0.31) | 1 | 0.33 (0.32 – 0.35) | 0.1605 | 0.3 (0.3 – 0.34) | 0.4206 |
| **MAD of COx-a_R (au)** | 0.26 (0.26 – 0.28) |  | 0.3 (0.3 – 0.3) |  | 0.31 (0.29 – 0.33) |  | 0.3 (0.28 – 0.32) |  | 0.3 (0.3 – 0.3) |  | 0.3 (0.28 – 0.32) |  | 0.28 (0.28 – 0.28) |  | 0.3 (0.3 – 0.33) |  | 0.29 (0.26 – 0.3) |  |
| **% time rSO2_L > 60%** | 81.41 (80.77 – 91.53; 11.43) | 0.2222 | 81.17 (75.79 – 96.26; 17.71) | 0.8726 | 60.87 (32.87 – 88.15; 46.1) | 0.6857 | 81.09 (74.2 – 90.22; 13.22) | 0.161 | 100 (100 – 100; 0) | 1 | 81.09 (74.2 – 90.22; 13.22) | 0.37 | 99.59 (99.59 – 99.59; 0) | 1 | 88.9 (75.97 – 99.64; 16.28) | 0.8745 | 80.77 (57.91 – 81.41; 5.29) | 0.0952 |
| **% time rSO2_R > 60%** | 99.75 (98.87 – 99.9; 0.37) |  | 85.95 (61.89 – 93.3; 16.89) |  | 83.42 (68.26 – 97.42; 21.83) |  | 95.69 (76.76 – 99.71; 6.39) |  | 54.94 (54.94 – 54.94; 0) |  | 95.69 (71.29 – 99.71; 6.39) |  | 89.17 (89.17 – 89.17; 0) |  | 97.13 (66.34 – 99.81; 4.25) |  | 96.69 (82.73 – 98.87; 4.76) |  |
| **% time rSO2_L > 70%** | 34.76 (34.15 – 65.44; 33.55) | 0.3095 | 33.44 (17.07 – 55.55; 36.82) | 0.6991 | 16.82 (1.86 – 42.46; 23.09) | 0.4857 | 32.65 (12.2 – 55.55; 37.05) | 0.21 | 69.69 (69.69 – 69.69; 0) | 1 | 34.46 (12.2 – 64.61; 43.47) | 0.4081 | 31.01 (31.01 – 31.01; 0) | 1 | 63.77 (27.52 – 71.37; 20.32) | 0.7984 | 12.43 (12.13 – 31.15; 18.43) | 0.2222 |
| **% time rSO2_R > 70%** | 61.04 (42.71 – 78.91; 27.18) |  | 27.53 (8.2 – 53.55; 34.8) |  | 39.7 (19.61 – 65.8; 37.56) |  | 50.58 (27.26 – 65.73; 31.45) |  | 0 (0 – 0; 0) |  | 50.58 (27.26 – 65.73; 31.45) |  | 8.43 (8.43 – 8.43; 0) |  | 55.19 (37 – 80.94; 41.18) |  | 42.71 (24.86 – 61.04; 27.18) |  |
| **% time rSO2_L > 80%** | 7.22 (0.83 – 17.95; 10.5) | 0.6905 | 0.4 (0.26 – 0.95; 0.41) | 0.4151 | 0.36 (0.23 – 0.49; 0.3) | 0.2454 | 0.6 (0.31 – 5.69; 0.72) | 0.9449 | 0.24 (0.24 – 0.24; 0) | 1 | 0.6 (0.26 – 5.69; 0.72) | 0.9449 | 0.32 (0.32 – 0.32; 0) | 1 | 0.45 (0.29 – 11.77; 0.44) | 0.8745 | 0.72 (0.14 – 1.11; 0.87) | 0.7533 |
| **% time rSO2_R > 80%** | 6.44 (0.13 – 20.5; 9.51) |  | 0.04 (0 – 6.06; 0.06) |  | 18.34 (0.57 – 50.67; 26.64) |  | 3.59 (0.04 – 24.94; 5.33) |  | 0 (0 – 0; 0) |  | 3.59 (0.04 – 24.94; 5.33) |  | 0 (0 – 0; 0) |  | 13.47 (0.06 – 37.69; 19.97) |  | 0.13 (0.02 – 0.75; 0.2) |  |
| **% time rSO2_L > 90%** | 0.01 (0 – 0.11; 0.01) | 0.6558 | 0 (0 – 0.03; 0) | 0.9241 | 0 (0 – 0; 0) | 0.1859 | 0 (0 – 0.03; 0) | 0.5146 | 0 (0 – 0; 0) | – | 0 (0 – 0.03; 0) | 0.5146 | 0 (0 – 0; 0) | – | 0 (0 – 2.1; 0) | 0.2516 | 0 (0 – 0.01; 0) | 0.7972 |
| **% time rSO2_R > 90%** | 0 (0 – 0.02; 0) |  | 0 (0 – 0.04; 0) |  | 7.28 (0 – 14.75; 10.8) |  | 0 (0 – 1.38; 0) |  | 0 (0 – 0; 0) |  | 0 (0 – 1.38; 0) |  | 0 (0 – 0; 0) |  | 0.01 (0 – 14.75; 0.01) |  | 0 (0 – 0; 0) |  |
| **% time COx_L > 0** | 52.51 (51.29 – 53.61; 1.81) | 0.8413 | 52.14 (39.54 – 59.34; 18.04) | 0.9372 | 61.64 (59.02 – 64; 4.39) | 0.6857 | 55.84 (51.6 – 60.64; 7.02) | 0.91 | 49.06 (49.06 – 49.06; 0) | 1 | 55.84 (51.6 – 60.64; 7.02) | 0.7345 | 35.58 (35.58 – 35.58; 0) | 1 | 54.41 (51.65 – 57.31; 5.49) | 0.7984 | 60.71 (60.43 – 63.41; 4) | 1 |
| **% time COx_R > 0** | 52.09 (51 – 52.58; 1.61) |  | 55.47 (50.28 – 62.36; 11.55) |  | 65.46 (59.79 – 73.32; 11.73) |  | 53.87 (51.27 – 60.81; 5.14) |  | 65.02 (65.02 – 65.02; 0) |  | 55.47 (52.21 – 63.81; 7.21) |  | 30.91 (30.91 – 30.91; 0) |  | 54.66 (52.19 – 62.62; 7.04) |  | 57.15 (54.68 – 68.88; 7.51) |  |
| **% time COx_L > 0.2** | 24.9 (24.35 – 30.83; 4.2) | 1 | 30.85 (21.55 – 36.95; 13.88) | 0.9372 | 35.26 (33.91 – 38.44; 3.07) | 0.6857 | 32.66 (24.49 – 35.84; 9.77) | 0.7688 | 28.15 (28.15 – 28.15; 0) | 1 | 32.66 (25.71 – 35.84; 7.36) | 0.982 | 19.35 (19.35 – 19.35; 0) | 1 | 31.3 (26.63 – 33.82; 4.8) | 0.9591 | 35.91 (35.64 – 38.08; 3.22) | 1 |
| **% time COx_R > 0.2** | 25.68 (24.52 – 28.93; 1.76) |  | 31.01 (25.12 – 35.3; 9.97) |  | 39.15 (34.14 – 47.8; 10.64) |  | 29.79 (24.81 – 35.02; 7.84) |  | 36.67 (36.67 – 36.67; 0) |  | 30.74 (26.18 – 36.58; 8.52) |  | 15.27 (15.27 – 15.27; 0) |  | 30.74 (27.16 – 36.39; 7.87) |  | 31.2 (28.93 – 42.01; 9.95) |  |
| **% time COx_L > 0.3** | 16.07 (15.23 – 22.26; 3.09) | 1 | 21.7 (15.25 – 26.56; 9.86) | 0.8182 | 25.37 (25.11 – 28.34; 0.74) | 0.6857 | 24.39 (15.44 – 25.39; 8.32) | 0.7688 | 19.03 (19.03 – 19.03; 0) | 1 | 24.39 (16.81 – 25.39; 6.12) | 0.7688 | 13.99 (13.99 – 13.99; 0) | 1 | 23.31 (17.77 – 24.65; 4.67) | 1 | 25.41 (24.86 – 27.29; 2.79) | 0.8413 |
| **% time COx_R > 0.3** | 16.15 (14.84 – 18.03; 2.08) |  | 22.34 (17.42 – 23.59; 6.05) |  | 27.67 (23.82 – 35.32; 8.43) |  | 20.29 (15.9 – 25; 7.3) |  | 23.98 (23.98 – 23.98; 0) |  | 22.34 (16.62 – 25.23; 7.77) |  | 9.99 (9.99 – 9.99; 0) |  | 22.72 (17.78 – 24.4; 5.43) |  | 22.25 (18.03 – 29.69; 10.99) |  |
| **% time COx-a_L > 0** | 55.69 (53.99 – 57.05; 2.52) | 0.3095 | 61.56 (59.83 – 63.44; 3.35) | 0.9372 | 62.75 (61.62 – 64.7; 2.47) | 0.6857 | 60.98 (57.64 – 63.04; 3.92) | 0.3519 | 57.46 (57.46 – 57.46; 0) | 1 | 60.42 (57.16 – 62.12; 4.35) | 0.3761 | 63.91 (63.91 – 63.91; 0) | 1 | 59.7 (57.36 – 61.38; 3.49) | 0.3823 | 62 (60.84 – 63.34; 1.98) | 0.6905 |
| **% time COx-a_R > 0** | 52.52 (51.51 – 52.63; 1.49) |  | 60.99 (55.77 – 69.54; 11.16) |  | 66.23 (60.72 – 71.87; 12.91) |  | 55.86 (51.77 – 65.43; 7.32) |  | 71.37 (71.37 – 71.37; 0) |  | 55.86 (51.77 – 65.43; 7.32) |  | 70.74 (70.74 – 70.74; 0) |  | 54.34 (51.42 – 64.43; 8.61) |  | 57.81 (55.68 – 68.54; 7.85) |  |
| **% time COx-a_L > 0.2** | 28.27 (28.14 – 30.24; 2.91) | 0.4206 | 37.85 (37.07 – 39.07; 1.71) | 0.6991 | 36.8 (35.67 – 40.18; 2.6) | 1 | 36.64 (31.13 – 38.12; 4.07) | 0.2456 | 34.23 (34.23 – 34.23; 0) | 1 | 35.55 (31.13 – 37.31; 3.41) | 0.2649 | 39.3 (39.3 – 39.3; 0) | 1 | 35.61 (32.92 – 37.31; 2.6) | 0.2786 | 36.29 (34.81 – 38.38; 3.1) | 0.8413 |
| **% time COx-a_R > 0.2** | 26.37 (22.65 – 28.78; 5.51) |  | 37.27 (29.7 – 43.97; 11.01) |  | 38.81 (33.34 – 45.19; 12.02) |  | 29.92 (25.65 – 40.22; 9.55) |  | 46.4 (46.4 – 46.4; 0) |  | 29.92 (25.65 – 40.22; 9.55) |  | 44.12 (44.12 – 44.12; 0) |  | 29.92 (26.13 – 37.87; 7.83) |  | 31.01 (28.78 – 41.63; 13.21) |  |
| **% time COx-a_L > 0.3** | 20.16 (18.54 – 21.99; 2.72) | 0.2222 | 27.51 (27.17 – 28.01; 0.81) | 0.6991 | 26.46 (25.59 – 29.72; 1.48) | 1 | 26.39 (22.6 – 27.53; 2.77) | 0.2273 | 24.4 (24.4 – 24.4; 0) | 1 | 25.46 (22.59 – 27.41; 3.5) | 0.2456 | 27.54 (27.54 – 27.54; 0) | 1 | 26.46 (23.8 – 27.65; 2.79) | 0.2345 | 25.21 (24.43 – 27.07; 2.76) | 0.6905 |
| **% time COx-a_R > 0.3** | 17.21 (13.03 – 17.65; 6.2) |  | 26.07 (20.96 – 31.23; 8.15) |  | 27.36 (23.38 – 33.63; 8.85) |  | 21.31 (17.26 – 28.35; 6.8) |  | 31.59 (31.59 – 31.59; 0) |  | 21.31 (17.26 – 28.35; 6.8) |  | 30.12 (30.12 – 30.12; 0) |  | 21.7 (17.35 – 26.93; 6.52) |  | 22.01 (17.65 – 29.34; 10.86) |  |
| **5-Minute Data Resolution** | | | | | | | | | | | | | | | | | | |
| **ABP (mmHg)** | 81.14 (76.32 – 87.5) | – | 87.93 (80.39 – 95.54) | – | 81.94 (75.44 – 89.4) | – | 83.58 (77.07 – 91.59) | – | 80.06 (69.99 – 86.11) | – | 82.33 (76.53 – 89.4) | – | 90.63 (83.23 – 100.2) | – | 85.17 (78.27 – 91.59) | – | 81.41 (75.65 – 88.4) | – |
| **CPP (mmHg)** | 72.3 (66.3 – 77.27) |  | 78.39 (71.21 – 85.54) |  | 71.91 (67.6 – 80.83) |  | 73.36 (68.11 – 82.01) |  | 73.71 (69.27 – 80.23) |  | 73.36 (68.11 – 80.32) |  | 79.56 (72.19 – 89.36) |  | 76.79 (70.11 – 84) |  | 72.3 (66.3 – 78.06) |  |
| **rSO2_L (%)** | 66.99 (60.99 – 74.25) | 0.8413 | 66.99 (61.67 – 71.59) | 0.5887 | 63.59 (56.49 – 65.88) | 0.4857 | 66.75 (60.99 – 71.59) | 0.4824 | 73.8 (69 – 77.04) | 1 | 66.75 (60.99 – 73.1) | 0.6673 | 67.48 (65.14 – 71.04) | 1 | 72.23 (65.82 – 75) | 0.9591 | 63.8 (60.99 – 67.71) | 0.2222 |
| **rSO2_R (%)** | 70.94 (68.8 – 76.91) |  | 65.15 (62.95 – 70.36) |  | 67.85 (59.82 – 77.57) |  | 70.01 (65.39 – 75.04) |  | 60.5 (56.57 – 62.61) |  | 70.01 (65.39 – 75.04) |  | 63.77 (61.71 – 66.9) |  | 71.39 (66.34 – 78.29) |  | 69.33 (66.58 – 71.63) |  |
| **COx_L (au)** | 0.01 (-0.15 – 0.17) | 0.8413 | 0.02 (-0.17 – 0.21) | 0.8182 | 0.08 (-0.06 – 0.27) | 0.6857 | 0.05 (-0.13 – 0.25) | 1 | 0 (-0.18 – 0.17) | 1 | 0.05 (-0.13 – 0.25) | 0.6673 | -0.12 (-0.29 – 0.1) | 1 | 0.03 (-0.14 – 0.23) | 0.6454 | 0.09 (-0.09 – 0.27) | 1 |
| **COx_R (au)** | 0.01 (-0.15 – 0.17) |  | 0.04 (-0.13 – 0.24) |  | 0.12 (-0.04 – 0.29) |  | 0.03 (-0.14 – 0.21) |  | 0.11 (-0.06 – 0.24) |  | 0.04 (-0.13 – 0.23) |  | -0.16 (-0.32 – 0.05) |  | 0.04 (-0.14 – 0.24) |  | 0.05 (-0.1 – 0.24) |  |
| **COx-a_L (au)** | 0.04 (-0.13 – 0.2) | 0.4206 | 0.09 (-0.09 – 0.29) | 0.6991 | 0.1 (-0.06 – 0.28) | 0.6857 | 0.08 (-0.09 – 0.27) | 0.3064 | 0.08 (-0.11 – 0.24) | 1 | 0.08 (-0.1 – 0.26) | 0.3287 | 0.11 (-0.07 – 0.3) | 1 | 0.08 (-0.1 – 0.27) | 0.3282 | 0.1 (-0.08 – 0.26) | 0.8413 |
| **COx-a_R (au)** | 0.02 (-0.13 – 0.17) |  | 0.09 (-0.08 – 0.27) |  | 0.12 (-0.03 – 0.28) |  | 0.04 (-0.12 – 0.23) |  | 0.17 (0.01 – 0.31) |  | 0.04 (-0.12 – 0.23) |  | 0.16 (-0.01 – 0.31) |  | 0.03 (-0.13 – 0.23) |  | 0.06 (-0.09 – 0.23) |  |
| **MAD of ABP (mmHg)** | 7.83 (7.59 – 8.09) | – | 11.45 (10.93 – 11.85) | – | 8.37 (6.32 – 10.34) | – | 9.31 (7.65 – 11.59) | – | 11.08 (11.08 – 11.08) | – | 9.31 (7.65 – 11.03) | – | 11.87 (11.87 – 11.87) | – | 10.24 (7.69 – 10.93) | – | 8.96 (7.83 – 12.4) | – |
| **MAD of CPP (mmHg)** | 7.96 (7.17 – 9.15) |  | 11.38 (10.27 – 12.56) |  | 8.65 (6.75 – 10.35) |  | 10.14 (7.38 – 10.93) |  | 7.51 (7.51 – 7.51) |  | 9.63 (7.26 – 10.54) |  | 12.18 (12.18 – 12.18) |  | 9.04 (7.42 – 10.27) |  | 10.43 (7.18 – 11.04) |  |
| **MAD of rSO2_L (%)** | 7.74 (4.86 – 7.82) | 0.4206 | 4.95 (4.36 – 6.66) | 0.0931 | 4.8 (3.54 – 7.01) | 1 | 5.29 (3.99 – 7.8) | 0.1936 | 5.29 (5.29 – 5.29) | 1 | 5.5 (4.07 – 7.8) | 0.1936 | 4.27 (4.27 – 4.27) | 1 | 6.2 (3.79 – 8.59) | 0.1949 | 4.86 (4.61 – 5.71) | 0.8413 |
| **MAD of rSO2_R (%)** | 3.8 (3.69 – 6.11) |  | 3.39 (2.77 – 3.84) |  | 6.39 (3.48 – 11.94) |  | 3.76 (3.11 – 6.76) |  | 3.89 (3.89 – 3.89) |  | 3.81 (3.11 – 6.76) |  | 3.72 (3.72 – 3.72) |  | 3.81 (2.97 – 4.44) |  | 3.69 (3.25 – 6.98) |  |
| **MAD of COx_L (au)** | 0.26 (0.25 – 0.27) | 0.5476 | 0.29 (0.26 – 0.3) | 0.5887 | 0.28 (0.25 – 0.32) | 0.6857 | 0.27 (0.26 – 0.3) | 0.3761 | 0.26 (0.26 – 0.26) | 1 | 0.26 (0.26 – 0.3) | 0.2852 | 0.28 (0.28 – 0.28) | 1 | 0.26 (0.26 – 0.3) | 0.8785 | 0.27 (0.26 – 0.3) | 0.2222 |
| **MAD of COx_R (au)** | 0.23 (0.22 – 0.24) |  | 0.27 (0.26 – 0.28) |  | 0.25 (0.24 – 0.27) |  | 0.26 (0.23 – 0.28) |  | 0.22 (0.22 – 0.22) |  | 0.25 (0.22 – 0.28) |  | 0.27 (0.27 – 0.27) |  | 0.26 (0.24 – 0.29) |  | 0.23 (0.22 – 0.26) |  |
| **MAD of COx-a_L (au)** | 0.24 (0.23 – 0.25) | 0.1508 | 0.28 (0.26 – 0.3) | 0.132 | 0.27 (0.25 – 0.28) | 0.6857 | 0.26 (0.25 – 0.29) | 0.1636 | 0.25 (0.25 – 0.25) | 1 | 0.26 (0.25 – 0.29) | 0.1371 | 0.27 (0.27 – 0.27) | 1 | 0.26 (0.26 – 0.28) | 0.5054 | 0.25 (0.24 – 0.29) | 0.2222 |
| **MAD of COx-a_R (au)** | 0.22 (0.21 – 0.22) |  | 0.25 (0.24 – 0.27) |  | 0.25 (0.24 – 0.28) |  | 0.25 (0.22 – 0.27) |  | 0.21 (0.21 – 0.21) |  | 0.24 (0.22 – 0.27) |  | 0.24 (0.24 – 0.24) |  | 0.26 (0.24 – 0.28) |  | 0.22 (0.21 – 0.25) |  |
| **% time rSO2_L > 60%** | 82.51 (81.76 – 91.94; 13.51) | 0.1508 | 81.41 (75.77 – 96.43; 17.77) | 0.8726 | 62.95 (35.5 – 88.48; 43.2) | 0.4857 | 82.13 (73.97 – 90.65; 13.75) | 0.1127 | 100 (100 – 100; 0) | 1 | 82.13 (73.97 – 90.65; 13.75) | 0.2899 | 99.65 (99.65 – 99.65; 0) | 1 | 89.36 (75.94 – 99.42; 15.56) | 0.5968 | 81.76 (58.73 – 82.51; 4.6) | 0.0952 |
| **% time rSO2_R > 60%** | 99.75 (99.08 – 99.79; 0.37) |  | 86.13 (60.03 – 93.68; 16.95) |  | 83.82 (68.34 – 98.27; 22.27) |  | 96.41 (77.33 – 99.78; 5.33) |  | 52.41 (52.41 – 52.41; 0) |  | 96.41 (71.34 – 99.78; 5.33) |  | 89.36 (89.36 – 89.36; 0) |  | 97.44 (65.57 – 100; 3.8) |  | 97.69 (82.89 – 99.08; 3.11) |  |
| **% time rSO2_L > 70%** | 34.87 (34.16 – 65.33; 33.18) | 0.3095 | 33.33 (17.22 – 55.55; 36.78) | 0.7483 | 17.22 (1.6 – 44; 23.96) | 0.4857 | 33.24 (12.51 – 55.55; 36.86) | 0.2063 | 70.28 (70.28 – 70.28; 0) | 1 | 34.51 (12.51 – 64.55; 43.38) | 0.395 | 31.07 (31.07 – 31.07; 0) | 1 | 63.77 (27.22 – 72.47; 22.71) | 0.7525 | 12.6 (12.49 – 32.32; 18.68) | 0.2222 |
| **% time rSO2_R > 70%** | 61.54 (42.37 – 79.55; 28.43) |  | 27.12 (8.02 – 55.12; 34.26) |  | 39.93 (19.98 – 65.73; 37.82) |  | 50.26 (27.71 – 65.94; 31.03) |  | 0 (0 – 0; 0) |  | 50.26 (27.71 – 65.94; 31.03) |  | 8.02 (8.02 – 8.02; 0) |  | 56.2 (36.66 – 81.52; 40.46) |  | 42.37 (25.54 – 61.54; 28.43) |  |
| **% time rSO2_L > 80%** | 7.65 (0.72 – 18.14; 11.11) | 0.6905 | 0.27 (0.06 – 1.02; 0.4) | 0.5497 | 0.46 (0.27 – 0.76; 0.42) | 0.3836 | 0.64 (0.26 – 6.07; 0.93) | 1 | 0 (0 – 0; 0) | – | 0.64 (0.18 – 6.07; 0.95) | 0.8891 | 0.29 (0.29 – 0.29; 0) | 1 | 0.54 (0.19 – 12.07; 0.79) | 0.7899 | 0.56 (0.16 – 1.26; 0.84) | 0.6723 |
| **% time rSO2_R > 80%** | 6.65 (0.13 – 21.44; 9.86) |  | 0 (0 – 6.46; 0) |  | 18.15 (0.6 – 50.1; 26.32) |  | 3.72 (0 – 25.14; 5.52) |  | 0 (0 – 0; 0) |  | 3.72 (0 – 25.14; 5.52) |  | 0 (0 – 0; 0) |  | 14.04 (0 – 37.37; 20.82) |  | 0.13 (0 – 0.8; 0.2) |  |
| **% time rSO2_L > 90%** | 0 (0 – 0.13; 0) | 0.6072 | 0 (0 – 0; 0) | 1 | 0 (0 – 0; 0) | 0.1859 | 0 (0 – 0; 0) | 0.5259 | 0 (0 – 0; 0) | – | 0 (0 – 0; 0) | 0.5259 | 0 (0 – 0; 0) | – | 0 (0 – 2.01; 0) | 0.4066 | 0 (0 – 0; 0) | – |
| **% time rSO2_R > 90%** | 0 (0 – 0; 0) |  | 0 (0 – 0; 0) |  | 7.2 (0 – 14.54; 10.67) |  | 0 (0 – 1.37; 0) |  | 0 (0 – 0; 0) |  | 0 (0 – 1.37; 0) |  | 0 (0 – 0; 0) |  | 0 (0 – 14.54; 0) |  | 0 (0 – 0; 0) |  |
| **% time COx_L > 0** | 50.88 (50.24 – 55.29; 5.86) | 1 | 52.84 (38.01 – 60.14; 20.12) | 0.9372 | 64.13 (60.47 – 68.48; 6.39) | 0.6857 | 57 (50.4 – 62.35; 9.54) | 0.91 | 50.21 (50.21 – 50.21; 0) | 1 | 57 (50.4 – 62.35; 9.54) | 0.7345 | 33.95 (33.95 – 33.95; 0) | 1 | 55.37 (50.23 – 59.18; 7.63) | 0.8785 | 62.56 (61.71 – 67.16; 6.81) | 1 |
| **% time COx_R > 0** | 51.12 (50.79 – 54.3; 1.3) |  | 55.28 (50.67 – 63.63; 12.61) |  | 69.76 (61.48 – 78.44; 16.43) |  | 54.59 (50.87 – 62.94; 6.45) |  | 69.66 (69.66 – 69.66; 0) |  | 55.28 (51.61 – 65.77; 7.07) |  | 30.42 (30.42 – 30.42; 0) |  | 54.59 (52.59 – 64.78; 6.52) |  | 58.94 (55.69 – 75.24; 12.09) |  |
| **% time COx_L > 0.2** | 21.72 (20.24 – 26.03; 6.39) | 1 | 25.62 (18.78 – 33.94; 12.93) | 1 | 33.22 (32.75 – 36.58; 1.19) | 0.6857 | 30.76 (20.61 – 33.29; 10.22) | 0.8743 | 21.46 (21.46 – 21.46; 0) | 1 | 30.76 (21.52 – 33.29; 10.22) | 1 | 17.89 (17.89 – 17.89; 0) | 1 | 27.91 (20.44 – 32.15; 8.82) | 0.7209 | 33.09 (31.75 – 35.33; 3.32) | 0.8413 |
| **% time COx_R > 0.2** | 21.96 (20.93 – 24.06; 3.11) |  | 29.54 (22.82 – 30.75; 7.52) |  | 36.84 (31.45 – 45.93; 12.13) |  | 26.21 (21.18 – 33.15; 8) |  | 31.03 (31.03 – 31.03; 0) |  | 28.78 (22.24 – 33.44; 9.28) |  | 13.73 (13.73 – 13.73; 0) |  | 29.12 (22.8 – 31.84; 8.28) |  | 29.2 (24.06 – 39.44; 15.09) |  |
| **% time COx_L > 0.3** | 12.06 (11.15 – 17.79; 1.41) | 0.6905 | 17.57 (12.89 – 22.7; 8.22) | 0.8182 | 21.6 (20.82 – 25.61; 1.26) | 0.6857 | 20.59 (12.13 – 21.97; 8.22) | 0.6347 | 14.59 (14.59 – 14.59; 0) | 1 | 20.59 (12.69 – 21.97; 6.54) | 0.8388 | 12.32 (12.32 – 12.32; 0) | 1 | 19.17 (13.72 – 21.06; 5.74) | 0.8785 | 20.88 (20.87 – 23.42; 3.76) | 0.8413 |
| **% time COx_R > 0.3** | 12.17 (10.94 – 14.82; 2.42) |  | 19.15 (12.24 – 22.01; 8.47) |  | 23.97 (19.64 – 31.75; 10.43) |  | 16.96 (11.09 – 22.5; 8.61) |  | 17.93 (17.93 – 17.93; 0) |  | 18.52 (11.7 – 22.5; 9.88) |  | 8.25 (8.25 – 8.25; 0) |  | 18.52 (12.01 – 22.39; 7.71) |  | 20.37 (14.82 – 25.61; 8.24) |  |
| **% time COx-a_L > 0** | 55.87 (55.68 – 55.98; 0.27) | 0.4206 | 63.01 (61.04 – 64.73; 3.45) | 0.9372 | 65.25 (64.34 – 67.85; 1.57) | 0.6857 | 63.1 (56.99 – 65; 4.47) | 0.4274 | 60.52 (60.52 – 60.52; 0) | 1 | 62.69 (56.99 – 64.34; 4.47) | 0.4544 | 65.17 (65.17 – 65.17; 0) | 1 | 61.96 (58.97 – 64.05; 3.32) | 0.4418 | 62.78 (62.6 – 66.03; 4.82) | 1 |
| **% time COx-a_R > 0** | 53.44 (51.71 – 54.09; 2.55) |  | 62.67 (57.15 – 71.67; 11.84) |  | 69.93 (61.59 – 76.55; 13.69) |  | 57.29 (52.14 – 67.3; 10.69) |  | 75.17 (75.17 – 75.17; 0) |  | 57.29 (52.14 – 67.3; 10.69) |  | 72.97 (72.97 – 72.97; 0) |  | 55.51 (50.78 – 66.39; 12.89) |  | 59.65 (57 – 73.93; 8.25) |  |
| **% time COx-a_L > 0.2** | 25.08 (25 – 25.81; 1.08) | 0.2222 | 34.8 (33.81 – 37.49; 2.68) | 0.6991 | 34.78 (32.75 – 39.15; 3.21) | 1 | 33.35 (27.29 – 36.44; 5.96) | 0.21 | 32.19 (32.19 – 32.19; 0) | 1 | 32.61 (27.29 – 35.27; 5.33) | 0.2456 | 38.08 (38.08 – 38.08; 0) | 1 | 33.06 (30.59 – 34.55; 3.32) | 0.3282 | 32.9 (31.75 – 35.75; 4.23) | 0.6905 |
| **% time COx-a_R > 0.2** | 22.39 (19.12 – 24.11; 4.86) |  | 34.84 (27.62 – 42.24; 11.39) |  | 35.22 (30.01 – 42.37; 11.45) |  | 27.55 (22.41 – 36.57; 10.08) |  | 45.52 (45.52 – 45.52; 0) |  | 27.55 (22.41 – 36.57; 10.08) |  | 42.64 (42.64 – 42.64; 0) |  | 27.55 (22.45 – 34.65; 7.6) |  | 28.67 (24.11 – 37.91; 13.71) |  |
| **% time COx-a_L > 0.3** | 16.28 (14.83 – 17.05; 2.15) | 0.2222 | 23.57 (22.61 – 24.54; 1.89) | 0.9372 | 23.5 (21.89 – 27.74; 3.36) | 0.8857 | 22.43 (17.76 – 24.23; 3.67) | 0.2456 | 16.31 (16.31 – 16.31; 0) | 1 | 21.25 (16.49 – 23.58; 5.48) | 0.3519 | 24.85 (24.85 – 24.85; 0) | 1 | 22.43 (16.86 – 23.8; 5.48) | 0.3823 | 20.21 (19.9 – 23.55; 4.95) | 0.6905 |
| **% time COx-a_R > 0.3** | 13.23 (9.17 – 14.11; 6.02) |  | 23.61 (18.99 – 27.34; 6.77) |  | 22.93 (18.62 – 29.51; 10.57) |  | 18.59 (13.36 – 23.67; 8.28) |  | 27.59 (27.59 – 27.59; 0) |  | 18.59 (13.36 – 23.67; 8.28) |  | 26.61 (26.61 – 26.61; 0) |  | 19.67 (13.61 – 22.98; 9.17) |  | 18.45 (14.11 – 24.41; 8.83) |  |
| *The p-values in the table are derived using Mann-Whitney U test between the bilateral signals.*  *ABP, arterial blood pressure; au, arbitrary units; CPP, cerebral perfusion pressure; COx, cerebral oximetry index with CPP; COx-a, cerebral oximetry index with ABP; CT, computed tomography; DAI, diffuse axonal injury; EDH, epidural hematoma; MAD, median absolute deviation; IQR, interquartile range; mmHg, millimeters of mercury; rSO2, regional cerebral oxygen saturation; tSAH, traumatic subarachnoid hemorrhage; SDH, subdural hematoma; aSDH, acute subdural hematoma; TBI-GL, traumatic brain injury patient group without left frontal lobe pathology.* | | | | | | | | | | | | | | | | | | |

| **Physiologic Variable** | **Sub-groups** | | | | | | | | | | | | | | | | | | | |
| --- | --- | --- | --- | --- | --- | --- | --- | --- | --- | --- | --- | --- | --- | --- | --- | --- | --- | --- | --- | --- |
|  | **Marshall CT III [n = 2]** | | **Rotterdam CT 6 [n = 1]** | | **Rotterdam CT 5 [n = 7]** | | **Rotterdam CT 4 [n = 5]** | | **Rotterdam CT 3 [n = 2]** | | **Propofol [n = 5]** | | **Fentanyl + Propofol [n = 3]** | | **Fentanyl + Ketamine + Propofol [n = 1]** | | **Fentanyl + Midazolam + Propofol [n = 4]** | | **Fentanyl + Ketamine + Midazolam + Propofol [n = 2]** | |
|  | **Median (IQR) or  Median (IQR; MAD)** | **p-value** | **Median (IQR) or  Median (IQR; MAD)** | **p-value** | **Median (IQR) or  Median (IQR; MAD)** | **p-value** | **Median (IQR) or  Median (IQR; MAD)** | **p-value** | **Median (IQR) or  Median (IQR; MAD)** | **p-value** | **Median (IQR) or  Median (IQR; MAD)** | **p-value** | **Median (IQR) or  Median (IQR; MAD)** | **p-value** | **Median (IQR) or  Median (IQR; MAD)** | **p-value** | **Median (IQR) or  Median (IQR; MAD)** | **p-value** | **Median (IQR) or  Median (IQR; MAD)** | **p-value** |
| **10-Second Data Resolution** | | | | | | | | | | | | | | | | | | | | |
| **ABP (mmHg)** | 85.79 (79.87 – 93.72) | – | 77.61 (72.37 – 82.05) | – | 83.6 (76.95 – 92.38) | – | 81.08 (76.01 – 88.04) | – | 91.25 (83.66 – 99.37) | – | 85.9 (78.51 – 92.83) | – | 79.44 (72.91 – 95.5) | – | 83.06 (74.8 – 92.38) | – | 83.78 (77.82 – 89.55) | – | 81.1 (75.71 – 87.5) | – |
| **CPP (mmHg)** | 74.08 (67.77 – 83.28) |  | 69.1 (63.59 – 73.45) |  | 76.37 (69.15 – 84.13) |  | 72.05 (66.2 – 78.03) |  | 80.53 (72.49 – 88.18) |  | 78.57 (72.89 – 86.14) |  | 69.1 (63.59 – 82.78) |  | 66.9 (58.97 – 76.31) |  | 74.94 (68.69 – 81.08) |  | 72.5 (66.93 – 78.93) |  |
| **rSO2_L (%)** | 67.25 (62.25 – 73.6) | 0.3333 | 79.99 (72.16 – 84.2) | 1 | 70.99 (62.74 – 74) | 0.7979 | 65 (60.99 – 70.99) | 1 | 64.85 (62.5 – 67.49) | 1 | 70.99 (69 – 74) | 1 | 67.5 (65 – 70.99) | 1 | 60.99 (57.47 – 63.99) | 1 | 67.12 (60.24 – 73.6) | 0.4857 | 64.5 (60.99 – 71) | 0.3333 |
| **rSO2_R (%)** | 63.56 (60.8 – 73.99) |  | 74.14 (71.99 – 76.99) |  | 72.11 (68.27 – 79.99) |  | 63.5 (61.99 – 71.71) |  | 65.23 (62.84 – 67.49) |  | 70.93 (68.27 – 72.71) |  | 63.93 (61.99 – 66.99) |  | 74 (68.54 – 78) |  | 63.09 (55.63 – 83.08) |  | 70.17 (67.73 – 72.37) |  |
| **COx_L (au)** | -0.07 (-0.25 – 0.15) | 1 | 0.01 (-0.17 – 0.19) | 1 | 0 (-0.11 – 0.26) | 0.5243 | 0.02 (-0.12 – 0.26) | 0.6723 | -0.12 (-0.33 – 0.12) | 1 | 0 (-0.21 – 0.25) | 0.8325 | 0.01 (-0.17 – 0.19) | 1 | 0 (-0.08 – 0.27) | – | 0.01 (-0.08 – 0.24) | 0.6198 | 0.02 (-0.17 – 0.23) | 1 |
| **COx_R (au)** | -0.08 (-0.27 – 0.13) |  | 0.02 (-0.17 – 0.22) |  | 0 (-0.09 – 0.23) |  | 0.03 (-0.12 – 0.22) |  | -0.09 (-0.29 – 0.13) |  | 0 (-0.19 – 0.25) |  | 0.02 (-0.17 – 0.22) |  | 0 (-0.07 – 0.16) |  | 0 (-0.03 – 0.21) |  | 0.02 (-0.15 – 0.21) |  |
| **COx-a_L (au)** | 0.06 (-0.13 – 0.25) | 1 | 0.04 (-0.14 – 0.25) | 1 | 0.01 (-0.1 – 0.27) | 0.413 | 0.02 (-0.09 – 0.26) | 0.9166 | 0.1 (-0.12 – 0.33) | 1 | 0.05 (-0.15 – 0.29) | 0.6752 | 0.12 (-0.1 – 0.33) | 1 | 0 (-0.07 – 0.27) | – | 0.01 (-0.05 – 0.26) | 0.6198 | 0.03 (-0.12 – 0.25) | 0.6667 |
| **COx-a_R (au)** | 0.08 (-0.1 – 0.26) |  | 0.02 (-0.17 – 0.22) |  | 0 (-0.06 – 0.23) |  | 0.03 (-0.11 – 0.22) |  | 0.07 (-0.13 – 0.27) |  | 0 (-0.2 – 0.25) |  | 0.16 (-0.04 – 0.35) |  | 0 (-0.06 – 0.16) |  | 0 (-0.03 – 0.2) |  | 0.02 (-0.13 – 0.2) |  |
| **MAD of ABP (mmHg)** | 9.83 (8.79 – 10.87) | – | 7.12 (7.12 – 7.12) | – | 11.39 (9.06 – 12.18) | – | 8.09 (7.75 – 9.21) | – | 11.34 (11.06 – 11.63) | – | 10.77 (7.9 – 11.85) | – | 11.91 (9.51 – 12.24) | – | 12.9 (12.9 – 12.9) | – | 8.98 (7.6 – 10.51) | – | 8.65 (8.37 – 8.93) | – |
| **MAD of CPP (mmHg)** | 10.88 (10.18 – 11.59) |  | 7.31 (7.31 – 7.31) |  | 11.1 (7.67 – 11.88) |  | 9.47 (7.22 – 10.48) |  | 11.33 (10.86 – 11.81) |  | 7.86 (7.49 – 10.38) |  | 11.42 (9.37 – 11.86) |  | 12.6 (12.6 – 12.6) |  | 10.29 (8.91 – 11.12) |  | 8.8 (7.96 – 9.64) |  |
| **MAD of rSO2_L (%)** | 8.5 (6.42 – 10.57) | 0.6667 | 7.53 (7.53 – 7.53) | 1 | 5.14 (4.45 – 9.1) | 0.5224 | 5.56 (4.45 – 7.43) | 0.6905 | 3.51 (3.09 – 3.93) | 0.6667 | 4.45 (2.97 – 5.14) | 0.6905 | 4.34 (3.41 – 5.94) | 0.4 | 4.45 (4.45 – 4.45) | 1 | 11.88 (9.72 – 13.07) | 0.6857 | 5.94 (5.19 – 6.68) | 0.3333 |
| **MAD of rSO2_R (%)** | 5.76 (4.79 – 6.73) |  | 3.97 (3.97 – 3.97) |  | 3.54 (3.17 – 6.55) |  | 3.83 (3.02 – 7.69) |  | 3.09 (2.72 – 3.46) |  | 3.29 (3.05 – 3.54) |  | 3.83 (3.03 – 3.9) |  | 7.16 (7.16 – 7.16) |  | 8.3 (5.77 – 11.91) |  | 3.42 (3.22 – 3.62) |  |
| **MAD of COx_L (au)** | 0.29 (0.28 – 0.31) | 1 | 0.26 (0.26 – 0.26) | 1 | 0.32 (0.26 – 0.35) | 0.3176 | 0.28 (0.27 – 0.32) | 0.8413 | 0.33 (0.32 – 0.33) | 0.3333 | 0.33 (0.32 – 0.33) | 0.6905 | 0.32 (0.29 – 0.39) | 1 | 0.28 (0.28 – 0.28) | 1 | 0.26 (0.25 – 0.3) | 0.4857 | 0.3 (0.29 – 0.31) | 0.6667 |
| **MAD of COx_R (au)** | 0.29 (0.28 – 0.3) |  | 0.28 (0.28 – 0.28) |  | 0.29 (0.18 – 0.32) |  | 0.27 (0.26 – 0.28) |  | 0.31 (0.31 – 0.31) |  | 0.31 (0.31 – 0.33) |  | 0.31 (0.3 – 0.34) |  | 0.16 (0.16 – 0.16) |  | 0.23 (0.15 – 0.26) |  | 0.27 (0.26 – 0.28) |  |
| **MAD of COx-a_L (au)** | 0.28 (0.26 – 0.3) | 0.6667 | 0.29 (0.29 – 0.29) | 1 | 0.32 (0.27 – 0.35) | 0.3176 | 0.27 (0.25 – 0.28) | 0.5476 | 0.33 (0.33 – 0.33) | 0.3333 | 0.32 (0.32 – 0.34) | 0.5476 | 0.32 (0.3 – 0.36) | 0.4 | 0.27 (0.27 – 0.27) | 1 | 0.25 (0.24 – 0.28) | 0.3429 | 0.27 (0.27 – 0.27) | 0.3333 |
| **MAD of COx-a_R (au)** | 0.27 (0.26 – 0.28) |  | 0.29 (0.29 – 0.29) |  | 0.3 (0.17 – 0.32) |  | 0.25 (0.25 – 0.26) |  | 0.3 (0.3 – 0.3) |  | 0.31 (0.3 – 0.33) |  | 0.29 (0.29 – 0.29) |  | 0.16 (0.16 – 0.16) |  | 0.22 (0.14 – 0.25) |  | 0.25 (0.25 – 0.25) |  |
| **% time rSO2_L > 60%** | 86.46 (80.08 – 92.84; 18.92) | 0.6667 | 91.04 (91.04 – 91.04; 0) | 1 | 85.64 (66.39 – 99.69; 21) | 0.9015 | 79.38 (73.7 – 80.07; 6.86) | 0.4206 | 86.75 (80.51 – 92.98; 18.49) | 1 | 99.57 (85.64 – 99.8; 0.58) | 0.6905 | 91.04 (62.33 – 95.13; 12.12) | 0.7 | 56.78 (56.78 – 56.78; 0) | 1 | 74.85 (59.93 – 78; 7.64) | 0.3429 | 79.73 (79.55 – 79.9; 0.51) | 0.3333 |
| **% time rSO2_R > 60%** | 80.51 (76.69 – 84.33; 11.31) |  | 99.78 (99.78 – 99.78; 0) |  | 82.7 (62.76 – 99.82; 25.63) |  | 96.06 (72.88 – 98.57; 5.78) |  | 91.14 (89.64 – 92.64; 4.45) |  | 99.65 (94.14 – 99.99; 0.51) |  | 96.06 (92.1 – 97.92; 5.52) |  | 82.7 (82.7 – 82.7; 0) |  | 66.14 (58.36 – 70.78; 7.91) |  | 99.26 (98.92 – 99.61; 1.02) |  |
| **% time rSO2_L > 70%** | 32.15 (31.07 – 33.24; 3.21) | 1 | 78.43 (78.43 – 78.43; 0) | 1 | 61.85 (23.74 – 65.89; 20.12) | 0.7104 | 29.97 (11.43 – 33.91; 6.44) | 0.3095 | 15.13 (7.7 – 22.56; 22.03) | 1 | 62.97 (35.17 – 68.81; 18.45) | 1 | 29.99 (14.99 – 54.21; 44.46) | 1 | 12.32 (12.32 – 12.32; 0) | 1 | 32.14 (23.13 – 41.2; 23.52) | 0.6857 | 22.67 (17.05 – 28.29; 16.67) | 0.3333 |
| **% time rSO2_R > 70%** | 21.44 (14.93 – 27.95; 19.3) |  | 86.4 (86.4 – 86.4; 0) |  | 54.44 (50.11 – 72.74; 18.93) |  | 34.46 (24.31 – 42.16; 15.04) |  | 8.07 (7.89 – 8.25; 0.52) |  | 53.44 (7.72 – 78.27; 67.78) |  | 8.42 (6.08 – 47.41; 6.96) |  | 67.21 (67.21 – 67.21; 0) |  | 40.62 (31.92 – 48.69; 14.81) |  | 50.64 (46.4 – 54.88; 12.57) |  |
| **% time rSO2_L > 80%** | 8.81 (4.56 – 13.05; 12.59) | 1 | 45.67 (45.67 – 45.67; 0) | 1 | 0.53 (0.33 – 0.86; 0.41) | 0.4557 | 0.69 (0.16 – 6.63; 1.03) | 0.7533 | 0.16 (0.08 – 0.24; 0.23) | 1 | 0.34 (0.2 – 0.53; 0.29) | 1 | 0.31 (0.16 – 22.99; 0.47) | 0.8248 | 0.9 (0.9 – 0.9; 0) | 1 | 9 (0.6 – 23.95; 12.59) | 0.6857 | 3.4 (1.78 – 5.01; 4.8) | 0.6667 |
| **% time rSO2_R > 80%** | 13.01 (6.5 – 19.51; 19.28) |  | 6.2 (6.2 – 6.2; 0) |  | 19.1 (3.71 – 39.34; 28.19) |  | 0.17 (0.02 – 0.65; 0.25) |  | 0 (0 – 0; 0) |  | 0.09 (0 – 19.1; 0.13) |  | 0 (0 – 3.1; 0) |  | 7.34 (7.34 – 7.34; 0) |  | 30.88 (19.67 – 37.55; 12.54) |  | 0.09 (0.06 – 0.13; 0.11) |  |
| **% time rSO2_L > 90%** | 0.03 (0.01 – 0.04; 0.04) | 1 | 7.51 (7.51 – 7.51; 0) | 1 | 0 (0 – 0.02; 0) | 0.1016 | 0 (0 – 0.02; 0) | 0.7972 | 0 (0 – 0; 0) | – | 0 (0 – 0; 0) | 0.1797 | 0 (0 – 3.75; 0) | 0.505 | 0.04 (0.04 – 0.04; 0) | 1 | 0.03 (0 – 2.61; 0.04) | 0.3005 | 0.01 (0 – 0.01; 0.01) | 0.6171 |
| **% time rSO2_R > 90%** | 0.81 (0.41 – 1.22; 1.2) |  | 0 (0 – 0; 0) |  | 0.06 (0.01 – 14.92; 0.09) |  | 0 (0 – 0; 0) |  | 0 (0 – 0; 0) |  | 0 (0 – 0.02; 0) |  | 0 (0 – 0; 0) |  | 0.06 (0.06 – 0.06; 0) |  | 8.1 (1.22 – 19.65; 10.81) |  | 0 (0 – 0; 0) |  |
| **% time COx_L > 0** | 41.27 (38.51 – 44.03; 8.19) | 0.6667 | 51.89 (51.89 – 51.89; 0) | 1 | 50.02 (47.37 – 52.34; 5.7) | 0.535 | 52.2 (46.79 – 53.63; 8.03) | 0.6905 | 35.95 (35.85 – 36.05; 0.3) | 1 | 50.02 (46.18 – 50.22; 5.7) | 0.5476 | 51.89 (43.82 – 58.54; 19.7) | 1 | 48.57 (48.57 – 48.57; 0) | 1 | 49.5 (45.8 – 54.08; 6.93) | 0.6857 | 49.54 (47.5 – 51.59; 6.06) | 1 |
| **% time COx_R > 0** | 39 (35.12 – 42.89; 11.52) |  | 52.07 (52.07 – 52.07; 0) |  | 46.57 (41.73 – 54.69; 12.78) |  | 53.57 (51.96 – 61.03; 10.08) |  | 39.74 (35.48 – 43.99; 12.6) |  | 49.84 (48.24 – 59.54; 4.85) |  | 52.07 (41.65 – 62.9; 30.89) |  | 37.95 (37.95 – 37.95; 0) |  | 46.14 (36.63 – 50.34; 11.51) |  | 52.77 (52.36 – 53.17; 1.19) |  |
| **% time COx_L > 0.2** | 21.58 (20.62 – 22.53; 2.84) | 0.6667 | 23.52 (23.52 – 23.52; 0) | 1 | 29.36 (27.74 – 32.41; 3.71) | 0.3176 | 30.89 (23.77 – 32.21; 10.55) | 1 | 18.88 (18.49 – 19.27; 1.15) | 1 | 28.62 (26.86 – 29.36; 2.61) | 0.8413 | 23.52 (21.59 – 34.99; 5.73) | 1 | 31.01 (31.01 – 31.01; 0) | 1 | 28.8 (25.9 – 34.48; 5.48) | 0.4857 | 27.99 (25.88 – 30.1; 6.25) | 1 |
| **% time COx_R > 0.2** | 19.51 (17.53 – 21.49; 5.87) |  | 26.89 (26.89 – 26.89; 0) |  | 27.17 (24.45 – 32.83; 7.3) |  | 27.46 (25.34 – 38.04; 5.92) |  | 19.99 (17.77 – 22.21; 6.59) |  | 28.58 (26.64 – 37.08; 6.14) |  | 26.89 (21.22 – 41.53; 16.81) |  | 22.25 (22.25 – 22.25; 0) |  | 25.32 (19.07 – 29.89; 10.8) |  | 26.4 (25.87 – 26.93; 1.58) |  |
| **% time COx_L > 0.3** | 14.53 (14.44 – 14.63; 0.27) | 0.6667 | 14.91 (14.91 – 14.91; 0) | 1 | 21.7 (20.67 – 23.75; 2.85) | 0.3829 | 22.01 (15.99 – 22.73; 8.92) | 1 | 13.24 (12.69 – 13.79; 1.64) | 1 | 21.55 (19.21 – 21.7; 3.48) | 0.5476 | 14.91 (14.63 – 26.43; 0.83) | 1 | 22.53 (22.53 – 22.53; 0) | 1 | 20.9 (18.52 – 25.85; 5.41) | 0.3429 | 19.36 (17.68 – 21.05; 5) | 0.6667 |
| **% time COx_R > 0.3** | 12.51 (11.52 – 13.5; 2.93) |  | 16.82 (16.82 – 16.82; 0) |  | 19.43 (17.11 – 23.54; 5.22) |  | 17.69 (15.75 – 27.52; 4.75) |  | 13.38 (11.96 – 14.8; 4.22) |  | 21.94 (18.31 – 25.14; 5.39) |  | 16.82 (13.68 – 30.97; 9.33) |  | 15.91 (15.91 – 15.91; 0) |  | 16.96 (11.94 – 21.45; 9.66) |  | 16.72 (16.23 – 17.2; 1.44) |  |
| **% time COx-a_L > 0** | 55.09 (50.69 – 59.48; 13.04) | 0.6667 | 55.48 (55.48 – 55.48; 0) | 1 | 50.7 (49.84 – 56.37; 5.56) | 0.2593 | 52.99 (52.3 – 53.68; 1.03) | 0.6905 | 62.56 (61.89 – 63.22; 1.96) | 1 | 54.59 (50.7 – 58.15; 5.76) | 0.6905 | 63.88 (59.68 – 66.14; 6.71) | 0.7 | 49.42 (49.42 – 49.42; 0) | 1 | 49.62 (46.79 – 54.3; 4.45) | 0.8857 | 53.34 (53.16 – 53.51; 0.52) | 1 |
| **% time COx-a_R > 0** | 58.65 (52.8 – 64.49; 17.33) |  | 51.96 (51.96 – 51.96; 0) |  | 46.9 (42.58 – 54.49; 12.59) |  | 54.28 (52.28 – 60.43; 9.13) |  | 57.99 (51.82 – 64.16; 18.29) |  | 47.51 (46.9 – 61.46; 2.76) |  | 70.08 (61.02 – 70.21; 0.37) |  | 38.41 (38.41 – 38.41; 0) |  | 46.86 (37.58 – 50.33; 10.13) |  | 53.28 (52.78 – 53.78; 1.48) |  |
| **% time COx-a_L > 0.2** | 30.86 (26.38 – 35.33; 13.26) | 1 | 29.19 (29.19 – 29.19; 0) | 1 | 31.2 (29.8 – 35.44; 3.37) | 0.1649 | 31.01 (27.7 – 31.56; 4.9) | 0.8413 | 38.94 (38.51 – 39.37; 1.27) | 1 | 33.47 (30.4 – 37.4; 5.83) | 0.6905 | 39.8 (34.49 – 44.22; 13.13) | 1 | 31.2 (31.2 – 31.2; 0) | 1 | 29.93 (27.11 – 34.29; 6.74) | 0.3429 | 29.63 (28.67 – 30.6; 2.86) | 0.3333 |
| **% time COx-a_R > 0.2** | 32.89 (27.24 – 38.53; 16.74) |  | 27.23 (27.23 – 27.23; 0) |  | 27.16 (23.19 – 34.72; 7.89) |  | 27.44 (22.86 – 37.52; 8.66) |  | 34.09 (29.05 – 39.13; 14.96) |  | 28.47 (24.55 – 40.98; 6.62) |  | 44.18 (35.7 – 46.67; 7.4) |  | 21.83 (21.83 – 21.83; 0) |  | 24.38 (17.58 – 29.75; 11.8) |  | 25.15 (24.01 – 26.29; 3.39) |  |
| **% time COx-a_L > 0.3** | 20.66 (16.86 – 24.45; 11.26) | 1 | 20.2 (20.2 – 20.2; 0) | 1 | 23.06 (22 – 26.04; 2.35) | 0.2086 | 21.88 (18.47 – 22.5; 5.05) | 0.8413 | 27.96 (27.81 – 28.1; 0.44) | 1 | 23.79 (23.06 – 27.66; 3.44) | 0.8413 | 28.25 (24.22 – 33.14; 11.94) | 1 | 22.53 (22.53 – 22.53; 0) | 1 | 21.64 (19.31 – 25.54; 6.53) | 0.3429 | 20.48 (19.48 – 21.49; 2.98) | 0.3333 |
| **% time COx-a_R > 0.3** | 21.88 (17.33 – 26.42; 13.47) |  | 17.88 (17.88 – 17.88; 0) |  | 19.19 (16.4 – 26.91; 5.35) |  | 17.41 (13.15 – 27.14; 6.85) |  | 23.79 (20.2 – 27.38; 10.64) |  | 21.81 (17.23 – 32.01; 7.7) |  | 30.96 (24.42 – 35.64; 13.86) |  | 15.58 (15.58 – 15.58; 0) |  | 15.99 (10.59 – 21.18; 10.63) |  | 15.28 (14.22 – 16.35; 3.16) |  |
| **1-Minute Data Resolution** | | | | | | | | | | | | | | | | | | | | |
| **ABP (mmHg)** | 85.83 (79.94 – 93.71) | – | 77.65 (72.53 – 81.64) | – | 83.74 (77.16 – 92.47) | – | 81.1 (76.24 – 87.99) | – | 91.32 (83.64 – 99.36) | – | 86.01 (78.63 – 92.69) | – | 79.34 (73.22 – 96.85) | – | 83.13 (74.84 – 92.47) | – | 83.86 (77.93 – 89.36) | – | 81.15 (75.87 – 87.45) | – |
| **CPP (mmHg)** | 74.08 (67.86 – 83.14) |  | 69.17 (63.42 – 72.89) |  | 76.18 (69.57 – 83.73) |  | 72.18 (66.28 – 77.93) |  | 80.57 (72.46 – 88) |  | 78.75 (72.81 – 86.13) |  | 69.17 (63.42 – 84.4) |  | 66.91 (59.03 – 76.5) |  | 74.88 (68.94 – 80.83) |  | 72.6 (67.03 – 79.01) |  |
| **rSO2_L (%)** | 67.24 (62.15 – 73.69) | 0.3333 | 79.99 (72.34 – 84.22) | 1 | 71.07 (62.67 – 74) | 0.7104 | 65 (60.99 – 70.99) | 1 | 64.9 (62.59 – 67.56) | 1 | 71.07 (69 – 74) | 0.8413 | 67.48 (65.08 – 71.12) | 1 | 60.99 (57.47 – 63.99) | 1 | 67.19 (60.01 – 73.63) | 0.4857 | 64.45 (60.99 – 71.04) | 0.3333 |
| **rSO2_R (%)** | 63.58 (60.83 – 73.95) |  | 74.23 (71.89 – 76.99) |  | 72.19 (68.36 – 79.83) |  | 63.56 (61.99 – 71.65) |  | 65.21 (62.97 – 67.53) |  | 70.73 (68.36 – 72.62) |  | 63.8 (61.99 – 66.99) |  | 74.08 (68.67 – 78) |  | 63.17 (55.59 – 83.03) |  | 70.17 (67.7 – 72.4) |  |
| **COx_L (au)** | -0.06 (-0.25 – 0.16) | 0.6667 | 0.02 (-0.16 – 0.18) | 1 | 0.06 (-0.18 – 0.29) | 1 | 0.09 (-0.12 – 0.3) | 0.8413 | -0.12 (-0.32 – 0.11) | 1 | 0.03 (-0.21 – 0.27) | 0.4206 | 0.02 (-0.16 – 0.18) | 1 | 0.1 (-0.14 – 0.32) | 1 | 0.08 (-0.14 – 0.3) | 0.6857 | 0.03 (-0.18 – 0.25) | 1 |
| **COx_R (au)** | -0.08 (-0.27 – 0.13) |  | 0.01 (-0.16 – 0.21) |  | 0.05 (-0.16 – 0.27) |  | 0.05 (-0.13 – 0.23) |  | -0.09 (-0.28 – 0.12) |  | 0.03 (-0.21 – 0.27) |  | 0.01 (-0.16 – 0.21) |  | 0.04 (-0.16 – 0.27) |  | 0.07 (-0.13 – 0.29) |  | 0.03 (-0.15 – 0.21) |  |
| **COx-a_L (au)** | 0.06 (-0.13 – 0.26) | 1 | 0.05 (-0.14 – 0.24) | 1 | 0.08 (-0.14 – 0.32) | 0.4557 | 0.08 (-0.11 – 0.29) | 1 | 0.1 (-0.11 – 0.32) | 1 | 0.08 (-0.16 – 0.31) | 0.6905 | 0.11 (-0.09 – 0.33) | 1 | 0.1 (-0.13 – 0.32) | 1 | 0.1 (-0.1 – 0.32) | 0.6857 | 0.06 (-0.13 – 0.26) | 0.6667 |
| **COx-a_R (au)** | 0.08 (-0.1 – 0.26) |  | 0.02 (-0.16 – 0.21) |  | 0.06 (-0.15 – 0.27) |  | 0.05 (-0.12 – 0.23) |  | 0.06 (-0.13 – 0.26) |  | 0.01 (-0.22 – 0.27) |  | 0.16 (-0.04 – 0.34) |  | 0.05 (-0.15 – 0.26) |  | 0.08 (-0.13 – 0.27) |  | 0.03 (-0.14 – 0.2) |  |
| **MAD of ABP (mmHg)** | 9.76 (8.71 – 10.8) | – | 6.86 (6.86 – 6.86) | – | 11.12 (8.86 – 11.95) | – | 7.97 (7.67 – 8.99) | – | 11.34 (11.09 – 11.6) | – | 10.84 (7.87 – 11.59) | – | 11.85 (9.36 – 11.97) | – | 12.92 (12.92 – 12.92) | – | 8.76 (7.52 – 10.17) | – | 8.48 (8.22 – 8.74) | – |
| **MAD of CPP (mmHg)** | 10.73 (9.99 – 11.46) |  | 7.25 (7.25 – 7.25) |  | 10.49 (7.83 – 11.81) |  | 9.26 (7.04 – 10.51) |  | 11.38 (10.97 – 11.79) |  | 8.01 (7.65 – 10.56) |  | 11.12 (9.18 – 11.66) |  | 12.63 (12.63 – 12.63) |  | 9.87 (8.7 – 10.61) |  | 8.77 (7.9 – 9.64) |  |
| **MAD of rSO2_L (%)** | 8.56 (6.43 – 10.69) | 0.6667 | 7.72 (7.72 – 7.72) | 1 | 5.17 (4.44 – 9) | 0.3829 | 5.6 (4.73 – 7.59) | 0.6905 | 3.57 (3.21 – 3.93) | 0.6667 | 4.32 (3.32 – 5.17) | 0.4206 | 4.29 (3.41 – 6.01) | 0.4 | 4.56 (4.56 – 4.56) | 1 | 11.88 (9.59 – 13.21) | 0.6857 | 6.16 (5.45 – 6.87) | 0.3333 |
| **MAD of rSO2_R (%)** | 5.84 (4.79 – 6.89) |  | 3.95 (3.95 – 3.95) |  | 3.56 (3.12 – 6.59) |  | 3.75 (3.21 – 7.94) |  | 3.14 (2.85 – 3.44) |  | 3.14 (3.11 – 3.56) |  | 3.73 (3.03 – 3.84) |  | 7.13 (7.13 – 7.13) |  | 8.43 (5.96 – 11.87) |  | 3.48 (3.35 – 3.62) |  |
| **MAD of COx_L (au)** | 0.3 (0.29 – 0.31) | 0.6667 | 0.25 (0.25 – 0.25) | 1 | 0.34 (0.34 – 0.36) | 0.1649 | 0.31 (0.3 – 0.33) | 0.1508 | 0.32 (0.32 – 0.32) | 0.3333 | 0.34 (0.32 – 0.34) | 0.5476 | 0.31 (0.28 – 0.37) | 1 | 0.34 (0.34 – 0.34) | 1 | 0.33 (0.3 – 0.38) | 0.4857 | 0.32 (0.31 – 0.32) | 0.3333 |
| **MAD of COx_R (au)** | 0.29 (0.29 – 0.3) |  | 0.27 (0.27 – 0.27) |  | 0.32 (0.31 – 0.34) |  | 0.29 (0.27 – 0.29) |  | 0.29 (0.29 – 0.3) |  | 0.32 (0.29 – 0.35) |  | 0.3 (0.29 – 0.32) |  | 0.32 (0.32 – 0.32) |  | 0.3 (0.29 – 0.31) |  | 0.27 (0.27 – 0.27) |  |
| **MAD of COx-a_L (au)** | 0.29 (0.28 – 0.3) | 0.6667 | 0.28 (0.28 – 0.28) | 1 | 0.34 (0.33 – 0.36) | 0.0973 | 0.3 (0.28 – 0.3) | 0.2222 | 0.32 (0.32 – 0.32) | 0.3333 | 0.34 (0.32 – 0.34) | 0.4206 | 0.31 (0.3 – 0.35) | 0.7 | 0.34 (0.34 – 0.34) | 1 | 0.31 (0.29 – 0.34) | 0.3429 | 0.29 (0.29 – 0.3) | 0.3333 |
| **MAD of COx-a_R (au)** | 0.27 (0.26 – 0.28) |  | 0.28 (0.28 – 0.28) |  | 0.31 (0.3 – 0.34) |  | 0.26 (0.26 – 0.29) |  | 0.29 (0.29 – 0.3) |  | 0.32 (0.3 – 0.35) |  | 0.28 (0.28 – 0.32) |  | 0.3 (0.3 – 0.3) |  | 0.29 (0.28 – 0.3) |  | 0.25 (0.25 – 0.26) |  |
| **% time rSO2_L > 60%** | 86.64 (80.17 – 93.12; 19.19) | 1 | 91.53 (91.53 – 91.53; 0) | 1 | 86.27 (66.99 – 99.68; 20.01) | 1 | 80.77 (73.7 – 81.41; 5.29) | 0.3095 | 87.65 (81.68 – 93.62; 17.71) | 1 | 99.59 (86.27 – 99.77; 0.6) | 1 | 91.53 (64.47 – 95.56; 11.95) | 0.7 | 57.91 (57.91 – 57.91; 0) | 1 | 74.88 (60.09 – 78.13; 7.88) | 0.4857 | 81.09 (80.93 – 81.25; 0.47) | 0.3333 |
| **% time rSO2_R > 60%** | 81.97 (78.37 – 85.57; 10.68) |  | 99.75 (99.75 – 99.75; 0) |  | 82.73 (62.54 – 99.79; 25.6) |  | 96.69 (74.76 – 98.87; 4.76) |  | 91.93 (90.55 – 93.3; 4.08) |  | 99.58 (94.68 – 100; 0.62) |  | 96.69 (92.93 – 98.22; 4.53) |  | 82.73 (82.73 – 82.73; 0) |  | 66.39 (58.65 – 71.29; 8.98) |  | 99.39 (99.13 – 99.65; 0.77) |  |
| **% time rSO2_L > 70%** | 32.88 (31.94 – 33.82; 2.78) | 0.6667 | 78.57 (78.57 – 78.57; 0) | 1 | 62.11 (24.15 – 67.57; 21.18) | 0.7104 | 31.15 (12.13 – 34.15; 5.34) | 0.4206 | 15.67 (8.01 – 23.34; 22.73) | 1 | 65.44 (35.87 – 69.69; 16.23) | 1 | 31.01 (15.5 – 54.79; 45.97) | 1 | 12.43 (12.43 – 12.43; 0) | 1 | 32.95 (23.98 – 41.59; 22.95) | 0.8857 | 23.14 (17.63 – 28.65; 16.33) | 0.3333 |
| **% time rSO2_R > 70%** | 21.43 (14.93 – 27.93; 19.28) |  | 87.03 (87.03 – 87.03; 0) |  | 55.85 (50.58 – 73.1; 16.96) |  | 34.44 (24.86 – 42.71; 14.19) |  | 8.27 (8.2 – 8.35; 0.23) |  | 55.85 (8.12 – 78.91; 64.84) |  | 8.43 (6.15 – 47.73; 6.76) |  | 67.29 (67.29 – 67.29; 0) |  | 40.53 (32.04 – 48.61; 14.9) |  | 51.87 (47.29 – 56.46; 13.59) |  |
| **% time rSO2_L > 80%** | 9.13 (4.72 – 13.54; 13.07) | 1 | 47.28 (47.28 – 47.28; 0) | 1 | 0.48 (0.36 – 0.97; 0.36) | 0.4557 | 0.72 (0.14 – 7.22; 1.07) | 0.7533 | 0.16 (0.08 – 0.24; 0.23) | 0.6171 | 0.41 (0.24 – 0.48; 0.25) | 1 | 0.32 (0.16 – 23.8; 0.47) | 0.6428 | 1.11 (1.11 – 1.11; 0) | 1 | 9.34 (0.62 – 24.61; 13.07) | 0.4857 | 3.68 (1.91 – 5.45; 5.25) | 0.3333 |
| **% time rSO2_R > 80%** | 13.21 (6.61 – 19.82; 19.59) |  | 6.44 (6.44 – 6.44; 0) |  | 20.5 (4.07 – 39.45; 30.28) |  | 0.13 (0.02 – 0.75; 0.2) |  | 0 (0 – 0; 0) |  | 0.08 (0 – 20.5; 0.11) |  | 0 (0 – 3.22; 0) |  | 8.06 (8.06 – 8.06; 0) |  | 31.18 (20 – 37.69; 12.27) |  | 0.08 (0.05 – 0.11; 0.08) |  |
| **% time rSO2_L > 90%** | 0.05 (0.03 – 0.08; 0.08) | 1 | 8.39 (8.39 – 8.39; 0) | 1 | 0 (0 – 0.02; 0) | 0.1016 | 0 (0 – 0.01; 0) | 0.7972 | 0 (0 – 0; 0) | – | 0 (0 – 0; 0) | 0.1797 | 0 (0 – 4.2; 0) | 0.505 | 0.03 (0.03 – 0.03; 0) | 1 | 0.05 (0 – 2.76; 0.08) | 0.3005 | 0 (0 – 0.01; 0.01) | 0.6171 |
| **% time rSO2_R > 90%** | 0.91 (0.46 – 1.37; 1.36) |  | 0 (0 – 0; 0) |  | 0.05 (0.01 – 14.92; 0.07) |  | 0 (0 – 0; 0) |  | 0 (0 – 0; 0) |  | 0 (0 – 0.02; 0) |  | 0 (0 – 0; 0) |  | 0.05 (0.05 – 0.05; 0) |  | 8.2 (1.37 – 19.7; 10.8) |  | 0 (0 – 0; 0) |  |
| **% time COx_L > 0** | 43.44 (39.51 – 47.36; 11.65) | 0.6667 | 52.51 (52.51 – 52.51; 0) | 1 | 56.46 (54.41 – 60.29; 5.04) | 1 | 60.43 (51.29 – 63.41; 7.93) | 0.6905 | 35.98 (35.78 – 36.17; 0.59) | 1 | 53.61 (49.06 – 55.22; 6.75) | 0.6905 | 52.51 (44.05 – 59.15; 19.68) | 1 | 60.71 (60.71 – 60.71; 0) | 1 | 59.94 (55.17 – 64.54; 8.5) | 0.8857 | 54 (50.79 – 57.22; 9.54) | 1 |
| **% time COx_R > 0** | 40.56 (35.73 – 45.39; 14.32) |  | 52.58 (52.58 – 52.58; 0) |  | 56.26 (53.87 – 63.22; 7.8) |  | 57.15 (52.09 – 68.88; 10.28) |  | 39.86 (35.38 – 44.34; 13.28) |  | 53.05 (51 – 64.4; 6.29) |  | 52.58 (41.74 – 69.61; 32.13) |  | 54.68 (54.68 – 54.68; 0) |  | 59.15 (54.75 – 63.74; 8.76) |  | 54.62 (53.35 – 55.89; 3.75) |  |
| **% time COx_L > 0.2** | 22.13 (20.74 – 23.51; 4.11) | 0.6667 | 22.07 (22.07 – 22.07; 0) | 1 | 33.55 (31.3 – 36.35; 4.03) | 0.62 | 35.64 (24.9 – 35.91; 15.4) | 1 | 18.3 (17.78 – 18.83; 1.55) | 1 | 30.83 (28.15 – 31.77; 3.98) | 0.8413 | 22.07 (20.71 – 34.05; 4.03) | 1 | 38.08 (38.08 – 38.08; 0) | 1 | 35.26 (32.19 – 39.75; 8.16) | 0.8857 | 29.99 (27.17 – 32.81; 8.37) | 1 |
| **% time COx_R > 0.2** | 19.89 (17.58 – 22.21; 6.85) |  | 25.68 (25.68 – 25.68; 0) |  | 31.2 (30.74 – 36.48; 5.26) |  | 28.93 (24.52 – 42.01; 6.58) |  | 19.25 (17.26 – 21.23; 5.89) |  | 30.66 (27.65 – 36.67; 8.91) |  | 25.68 (20.48 – 45.43; 15.44) |  | 31.2 (31.2 – 31.2; 0) |  | 33.55 (29.24 – 37.72; 8.3) |  | 26.71 (25.6 – 27.82; 3.29) |  |
| **% time COx_L > 0.3** | 14.61 (14.3 – 14.92; 0.92) | 0.6667 | 13.99 (13.99 – 13.99; 0) | 1 | 24.41 (23.31 – 26.32; 3.2) | 0.4557 | 24.86 (16.07 – 25.41; 13.02) | 0.8413 | 12.93 (12.39 – 13.46; 1.58) | 1 | 22.26 (19.03 – 24.36; 3.2) | 1 | 13.99 (13.99 – 25.56; 0) | 1 | 27.29 (27.29 – 27.29; 0) | 1 | 25.37 (22.81 – 29.51; 7.54) | 0.8857 | 20.47 (18.27 – 22.66; 6.51) | 0.6667 |
| **% time COx_R > 0.3** | 12.37 (11.18 – 13.56; 3.53) |  | 16.15 (16.15 – 16.15; 0) |  | 23.03 (22.34 – 24.81; 1.41) |  | 18.03 (14.84 – 29.69; 4.87) |  | 12.9 (11.44 – 14.35; 4.32) |  | 23.03 (18.33 – 23.98; 6.97) |  | 16.15 (13.07 – 34.17; 9.14) |  | 22.25 (22.25 – 22.25; 0) |  | 24.04 (20.5 – 26.66; 5.39) |  | 16.44 (15.64 – 17.24; 2.37) |  |
| **% time COx-a_L > 0** | 57.36 (54.08 – 60.64; 9.72) | 1 | 57.05 (57.05 – 57.05; 0) | 1 | 60 (58.43 – 62.08; 3.21) | 0.7104 | 60.84 (55.69 – 63.34; 7.65) | 1 | 62.52 (61.82 – 63.22; 2.07) | 1 | 59.4 (57.46 – 60; 2.55) | 0.6905 | 63.91 (60.48 – 66.36; 7.26) | 0.7 | 62 (62 – 62; 0) | 1 | 62.75 (59.32 – 64.69; 4.89) | 0.8857 | 58.26 (56.97 – 59.55; 3.82) | 0.6667 |
| **% time COx-a_R > 0** | 60.73 (55.72 – 65.73; 14.84) |  | 52.63 (52.63 – 52.63; 0) |  | 56.04 (53.6 – 64.93; 7.28) |  | 57.81 (52.52 – 68.54; 10.53) |  | 58.33 (52.13 – 64.53; 18.4) |  | 51.51 (51.13 – 65.94; 8.29) |  | 70.74 (61.68 – 76.3; 16.49) |  | 55.68 (55.68 – 55.68; 0) |  | 59.98 (54.71 – 65.08; 9.27) |  | 55.17 (53.85 – 56.49; 3.92) |  |
| **% time COx-a_L > 0.2** | 31.25 (27.22 – 35.28; 11.94) | 1 | 28.14 (28.14 – 28.14; 0) | 1 | 37.31 (34.02 – 37.85; 4.57) | 0.3829 | 34.81 (28.27 – 36.29; 9.69) | 0.8413 | 38.15 (37.57 – 38.73; 1.71) | 1 | 34.23 (33.81 – 36.99; 4.1) | 0.8413 | 39.3 (33.72 – 44.05; 14.06) | 1 | 38.38 (38.38 – 38.38; 0) | 1 | 36.8 (33.02 – 40.48; 10.14) | 0.4857 | 31.54 (29.91 – 33.17; 4.84) | 0.6667 |
| **% time COx-a_R > 0.2** | 33.38 (28.02 – 38.75; 15.91) |  | 26.37 (26.37 – 26.37; 0) |  | 31.01 (29.92 – 39.76; 7.38) |  | 28.78 (22.65 – 41.63; 9.92) |  | 33.61 (28.36 – 38.86; 15.58) |  | 30.59 (25.42 – 43.53; 11.1) |  | 44.12 (35.24 – 49.98; 17.4) |  | 31.01 (31.01 – 31.01; 0) |  | 32.62 (27.61 – 37.4; 9.17) |  | 25.44 (23.77 – 27.11; 4.96) |  |
| **% time COx-a_L > 0.3** | 20.52 (17.01 – 24.03; 10.41) | 1 | 20.16 (20.16 – 20.16; 0) | 1 | 27.07 (25.06 – 27.68; 2.01) | 0.3176 | 24.43 (18.54 – 25.21; 8.72) | 0.6905 | 27.51 (27.5 – 27.53; 0.05) | 1 | 25.71 (24.4 – 27.48; 2.62) | 0.8413 | 27.54 (23.85 – 32.41; 10.96) | 1 | 27.07 (27.07 – 27.07; 0) | 1 | 26.21 (22.28 – 30.7; 10.16) | 0.6857 | 21.49 (20.01 – 22.96; 4.36) | 0.3333 |
| **% time COx-a_R > 0.3** | 21.58 (17.3 – 25.85; 12.67) |  | 17.21 (17.21 – 17.21; 0) |  | 22.8 (21.31 – 28.48; 3.81) |  | 17.65 (13.03 – 29.34; 7.5) |  | 23.18 (19.71 – 26.65; 10.29) |  | 22.8 (17.4 – 31.59; 9.73) |  | 30.12 (23.67 – 38.32; 19.14) |  | 22.01 (22.01 – 22.01; 0) |  | 22.99 (18.71 – 26.37; 6.47) |  | 15.12 (13.86 – 16.39; 3.75) |  |
| **5-Minute Data Resolution** | | | | | | | | | | | | | | | | | | | | |
| **ABP (mmHg)** | 85.89 (79.99 – 93.85) | – | 77.49 (72.53 – 81.47) | – | 83.93 (77.39 – 92.78) | – | 81.14 (76.32 – 88.14) | – | 91.32 (83.8 – 99.57) | – | 86.41 (79.15 – 92.78) | – | 79.96 (73.49 – 96.59) | – | 83.24 (75.13 – 92.78) | – | 84.03 (78.17 – 89.4) | – | 81.2 (75.98 – 87.52) | – |
| **CPP (mmHg)** | 74.05 (67.94 – 83.31) |  | 69.27 (63.52 – 72.66) |  | 76.36 (70 – 83.61) |  | 72.3 (66.3 – 78.06) |  | 80.59 (72.64 – 88.03) |  | 79.39 (73.09 – 86.26) |  | 69.27 (63.52 – 84.99) |  | 67.01 (59.15 – 76.9) |  | 74.99 (69.16 – 80.83) |  | 72.7 (67.09 – 78.82) |  |
| **rSO2_L (%)** | 67.24 (62.01 – 73.62) | 0.3333 | 79.96 (72.36 – 84.22) | 1 | 71.11 (62.64 – 74.06) | 0.8048 | 65.02 (60.99 – 70.99) | 1 | 64.94 (62.6 – 67.5) | 1 | 71.11 (69 – 74.06) | 0.8413 | 67.48 (65.14 – 71.04) | 1 | 61.02 (57.49 – 63.94) | 1 | 67.24 (59.79 – 73.59) | 0.4857 | 64.41 (60.99 – 70.98) | 0.3333 |
| **rSO2_R (%)** | 63.58 (60.88 – 73.87) |  | 74.31 (71.88 – 76.91) |  | 72.07 (68.48 – 79.68) |  | 63.63 (61.99 – 71.63) |  | 65.15 (62.95 – 67.55) |  | 70.7 (68.48 – 72.51) |  | 63.77 (61.99 – 66.9) |  | 74.12 (68.72 – 78.03) |  | 63.21 (55.59 – 82.94) |  | 70.14 (67.69 – 72.4) |  |
| **COx_L (au)** | -0.06 (-0.22 – 0.13) | 0.6667 | 0 (-0.12 – 0.13) | 1 | 0.06 (-0.13 – 0.26) | 0.8048 | 0.08 (-0.09 – 0.26) | 1 | -0.11 (-0.28 – 0.09) | 1 | 0.03 (-0.17 – 0.22) | 0.5476 | 0 (-0.12 – 0.13) | 1 | 0.09 (-0.12 – 0.29) | 1 | 0.08 (-0.09 – 0.27) | 0.8857 | 0.03 (-0.15 – 0.21) | 1 |
| **COx_R (au)** | -0.08 (-0.24 – 0.11) |  | 0.02 (-0.13 – 0.17) |  | 0.06 (-0.12 – 0.24) |  | 0.05 (-0.1 – 0.19) |  | -0.08 (-0.25 – 0.1) |  | 0.03 (-0.16 – 0.23) |  | 0.02 (-0.13 – 0.17) |  | 0.03 (-0.12 – 0.24) |  | 0.08 (-0.11 – 0.27) |  | 0.03 (-0.12 – 0.18) |  |
| **COx-a_L (au)** | 0.06 (-0.11 – 0.23) | 0.6667 | 0.05 (-0.11 – 0.19) | 1 | 0.08 (-0.11 – 0.29) | 0.62 | 0.07 (-0.08 – 0.26) | 1 | 0.1 (-0.07 – 0.28) | 1 | 0.07 (-0.11 – 0.26) | 0.6905 | 0.11 (-0.07 – 0.3) | 1 | 0.1 (-0.11 – 0.29) | 1 | 0.1 (-0.06 – 0.28) | 1 | 0.06 (-0.11 – 0.23) | 0.6667 |
| **COx-a_R (au)** | 0.08 (-0.08 – 0.23) |  | 0.02 (-0.13 – 0.17) |  | 0.04 (-0.1 – 0.23) |  | 0.06 (-0.09 – 0.19) |  | 0.07 (-0.1 – 0.23) |  | 0.01 (-0.16 – 0.22) |  | 0.16 (-0.01 – 0.31) |  | 0.04 (-0.1 – 0.23) |  | 0.07 (-0.1 – 0.25) |  | 0.04 (-0.11 – 0.17) |  |
| **MAD of ABP (mmHg)** | 9.73 (8.66 – 10.8) | – | 6.51 (6.51 – 6.51) | – | 10.87 (8.87 – 11.45) | – | 7.83 (7.59 – 8.96) | – | 11.35 (11.09 – 11.61) | – | 10.83 (8.09 – 11.08) | – | 11.87 (9.19 – 12.13) | – | 12.84 (12.84 – 12.84) | – | 8.62 (7.46 – 9.96) | – | 8.39 (8.11 – 8.67) | – |
| **MAD of CPP (mmHg)** | 10.66 (9.91 – 11.42) |  | 7.17 (7.17 – 7.17) |  | 10.11 (7.73 – 11.64) |  | 9.15 (7.18 – 10.43) |  | 11.17 (10.67 – 11.68) |  | 7.96 (7.51 – 10.17) |  | 11.04 (9.11 – 11.61) |  | 12.69 (12.69 – 12.69) |  | 9.63 (8.66 – 10.23) |  | 8.66 (7.78 – 9.55) |  |
| **MAD of rSO2_L (%)** | 8.6 (6.43 – 10.77) | 0.6667 | 7.82 (7.82 – 7.82) | 1 | 5.29 (4.25 – 9) | 0.3829 | 5.71 (4.86 – 7.74) | 0.6905 | 3.65 (3.34 – 3.96) | 0.6667 | 3.89 (3.47 – 5.29) | 0.5476 | 4.27 (3.37 – 6.04) | 0.4 | 4.61 (4.61 – 4.61) | 1 | 11.91 (9.6 – 13.37) | 0.6857 | 6.3 (5.58 – 7.02) | 0.3333 |
| **MAD of rSO2_R (%)** | 5.89 (4.8 – 6.97) |  | 3.8 (3.8 – 3.8) |  | 3.89 (3.44 – 6.55) |  | 3.69 (3.25 – 8.05) |  | 3.2 (2.94 – 3.46) |  | 3.82 (3.07 – 3.89) |  | 3.72 (3.09 – 3.76) |  | 6.98 (6.98 – 6.98) |  | 8.51 (6.04 – 11.94) |  | 3.47 (3.36 – 3.58) |  |
| **MAD of COx_L (au)** | 0.26 (0.25 – 0.27) | 0.6667 | 0.18 (0.18 – 0.18) | 1 | 0.3 (0.26 – 0.3) | 0.535 | 0.26 (0.25 – 0.27) | 0.1508 | 0.27 (0.26 – 0.27) | 1 | 0.26 (0.26 – 0.27) | 1 | 0.28 (0.23 – 0.33) | 1 | 0.3 (0.3 – 0.3) | 1 | 0.27 (0.24 – 0.32) | 0.6857 | 0.26 (0.26 – 0.26) | 0.3333 |
| **MAD of COx_R (au)** | 0.26 (0.25 – 0.27) |  | 0.22 (0.22 – 0.22) |  | 0.26 (0.25 – 0.3) |  | 0.23 (0.22 – 0.24) |  | 0.27 (0.26 – 0.27) |  | 0.26 (0.25 – 0.29) |  | 0.27 (0.25 – 0.29) |  | 0.26 (0.26 – 0.26) |  | 0.25 (0.24 – 0.28) |  | 0.22 (0.22 – 0.22) |  |
| **MAD of COx-a_L (au)** | 0.25 (0.24 – 0.26) | 0.6667 | 0.23 (0.23 – 0.23) | 1 | 0.27 (0.26 – 0.3) | 0.3176 | 0.24 (0.23 – 0.25) | 0.0952 | 0.27 (0.26 – 0.27) | 0.3333 | 0.26 (0.26 – 0.27) | 1 | 0.27 (0.25 – 0.3) | 0.7 | 0.29 (0.29 – 0.29) | 1 | 0.25 (0.23 – 0.29) | 0.4857 | 0.25 (0.25 – 0.25) | 0.3333 |
| **MAD of COx-a_R (au)** | 0.23 (0.23 – 0.24) |  | 0.22 (0.22 – 0.22) |  | 0.26 (0.24 – 0.28) |  | 0.22 (0.21 – 0.22) |  | 0.25 (0.25 – 0.25) |  | 0.26 (0.26 – 0.29) |  | 0.24 (0.23 – 0.28) |  | 0.25 (0.25 – 0.25) |  | 0.23 (0.22 – 0.25) |  | 0.21 (0.21 – 0.21) |  |
| **% time rSO2_L > 60%** | 86.52 (79.96 – 93.09; 19.46) | 1 | 91.94 (91.94 – 91.94; 0) | 1 | 86.79 (67.21 – 99.52; 19.17) | 0.8463 | 81.76 (73.4 – 82.51; 4.6) | 0.3095 | 87.84 (81.93 – 93.74; 17.51) | 1 | 99.32 (86.79 – 99.71; 1) | 0.59 | 91.94 (66.49 – 95.8; 11.43) | 0.7 | 58.73 (58.73 – 58.73; 0) | 1 | 74.54 (59.76 – 77.98; 8.5) | 0.4857 | 82.13 (81.95 – 82.32; 0.56) | 0.3333 |
| **% time rSO2_R > 60%** | 82.42 (78.95 – 85.89; 10.29) |  | 99.75 (99.75 – 99.75; 0) |  | 82.89 (61.18 – 100; 25.37) |  | 97.69 (75.48 – 99.08; 3.11) |  | 92.24 (90.8 – 93.68; 4.27) |  | 100 (95.12 – 100; 0) |  | 97.69 (93.53 – 98.72; 3.05) |  | 82.89 (82.89 – 82.89; 0) |  | 66.73 (59.24 – 71.34; 8.87) |  | 99.43 (99.26 – 99.61; 0.53) |  |
| **% time rSO2_L > 70%** | 32.97 (32.02 – 33.92; 2.81) | 0.6667 | 79.12 (79.12 – 79.12; 0) | 1 | 62.21 (24.09 – 67.81; 24.97) | 0.7104 | 32.32 (12.49 – 34.16; 3.78) | 0.4206 | 15.54 (7.77 – 23.31; 23.04) | 1 | 65.33 (35.58 – 70.28; 20.35) | 0.9166 | 31.07 (15.54 – 55.1; 46.07) | 1 | 12.6 (12.6 – 12.6; 0) | 1 | 33.59 (24.77 – 41.7; 22.16) | 0.8857 | 23.32 (17.9 – 28.74; 16.06) | 0.3333 |
| **% time rSO2_R > 70%** | 21.11 (14.57 – 27.66; 19.41) |  | 87.44 (87.44 – 87.44; 0) |  | 58.09 (50.26 – 73.48; 17.61) |  | 34.21 (25.54 – 42.37; 12.85) |  | 8.02 (8.02 – 8.02; 0.01) |  | 58.09 (8.01 – 79.55; 62.14) |  | 8.02 (5.66 – 47.73; 7.01) |  | 67.4 (67.4 – 67.4; 0) |  | 40.21 (32.04 – 48.24; 14.9) |  | 51.96 (47.16 – 56.75; 14.21) |  |
| **% time rSO2_L > 80%** | 9.22 (4.76 – 13.68; 13.23) | 1 | 49.45 (49.45 – 49.45; 0) | 1 | 0.72 (0.3 – 1.31; 0.81) | 0.369 | 0.56 (0.16 – 7.65; 0.84) | 0.6723 | 0.15 (0.07 – 0.22; 0.22) | 0.6171 | 0.25 (0 – 0.72; 0.38) | 1 | 0.29 (0.15 – 24.87; 0.43) | 0.6428 | 1.26 (1.26 – 1.26; 0) | 1 | 9.35 (0.51 – 24.66; 13.19) | 0.4857 | 3.9 (2.03 – 5.78; 5.55) | 0.3333 |
| **% time rSO2_R > 80%** | 13.19 (6.6 – 19.79; 19.56) |  | 6.65 (6.65 – 6.65; 0) |  | 21.44 (4.31 – 39.23; 31.78) |  | 0.13 (0 – 0.8; 0.2) |  | 0 (0 – 0; 0) |  | 0 (0 – 21.44; 0) |  | 0 (0 – 3.33; 0) |  | 8.61 (8.61 – 8.61; 0) |  | 30.95 (19.98 – 37.37; 12.28) |  | 0.07 (0.03 – 0.1; 0.1) |  |
| **% time rSO2_L > 90%** | 0.06 (0.03 – 0.09; 0.09) | 1 | 8.06 (8.06 – 8.06; 0) | 1 | 0 (0 – 0; 0) | 0.2004 | 0 (0 – 0; 0) | 1 | 0 (0 – 0; 0) | – | 0 (0 – 0; 0) | 0.4237 | 0 (0 – 4.03; 0) | 0.505 | 0 (0 – 0; 0) | – | 0.06 (0 – 2.67; 0.09) | 0.3005 | 0 (0 – 0; 0) | – |
| **% time rSO2_R > 90%** | 0.92 (0.46 – 1.37; 1.36) |  | 0 (0 – 0; 0) |  | 0 (0 – 14.68; 0) |  | 0 (0 – 0; 0) |  | 0 (0 – 0; 0) |  | 0 (0 – 0; 0) |  | 0 (0 – 0; 0) |  | 0 (0 – 0; 0) |  | 8.12 (1.37 – 19.64; 10.67) |  | 0 (0 – 0; 0) |  |
| **% time COx_L > 0** | 42.41 (38.18 – 46.65; 12.56) | 0.6667 | 50.24 (50.24 – 50.24; 0) | 1 | 58.54 (55.37 – 61.41; 4.7) | 1 | 62.56 (50.88 – 67.16; 14.68) | 0.8413 | 32.9 (32.38 – 33.42; 1.54) | 1 | 55.29 (50.21 – 55.46; 7.53) | 0.6905 | 50.24 (42.09 – 61.35; 24.16) | 1 | 61.71 (61.71 – 61.71; 0) | 1 | 62.85 (56.62 – 68.14; 9.32) | 0.8857 | 54.75 (50.84 – 58.65; 11.59) | 1 |
| **% time COx_R > 0** | 40.33 (35.38 – 45.28; 14.69) |  | 54.3 (54.3 – 54.3; 0) |  | 55.69 (53.98 – 65.28; 6.78) |  | 58.94 (50.79 – 75.24; 12.9) |  | 39.84 (35.13 – 44.55; 13.97) |  | 53.08 (51.12 – 66.27; 5.66) |  | 54.3 (42.36 – 71.17; 35.41) |  | 55.69 (55.69 – 55.69; 0) |  | 59.58 (53.72 – 67.02; 10.41) |  | 54.86 (52.82 – 56.9; 6.04) |  |
| **% time COx_L > 0.2** | 19.8 (18.85 – 20.76; 2.84) | 0.6667 | 17.39 (17.39 – 17.39; 0) | 1 | 31.75 (27.91 – 34.34; 5.31) | 0.8048 | 31.75 (21.72 – 33.09; 14.87) | 1 | 16.54 (15.87 – 17.22; 2) | 1 | 26.03 (21.46 – 29.78; 6.78) | 0.6905 | 17.89 (17.64 – 32.07; 0.74) | 1 | 35.33 (35.33 – 35.33; 0) | 1 | 33.22 (30.25 – 37.92; 8.62) | 0.8857 | 26 (23.12 – 28.87; 8.53) | 0.6667 |
| **% time COx_R > 0.2** | 17.33 (15.53 – 19.13; 5.33) |  | 21.96 (21.96 – 21.96; 0) |  | 29.88 (28.78 – 32.64; 2.26) |  | 24.06 (20.93 – 39.44; 7.46) |  | 17.21 (15.47 – 18.95; 5.16) |  | 28.35 (23.08 – 31.03; 7.82) |  | 21.96 (17.85 – 43.68; 12.19) |  | 29.2 (29.2 – 29.2; 0) |  | 32.06 (27.64 – 35.54; 7.09) |  | 21.54 (20.28 – 22.8; 3.73) |  |
| **% time COx_L > 0.3** | 11.74 (11.45 – 12.03; 0.87) | 0.3333 | 11.11 (11.11 – 11.11; 0) | 1 | 20.63 (19.17 – 22.87; 4.12) | 0.8048 | 20.87 (12.06 – 20.88; 13.06) | 0.8413 | 10.33 (9.33 – 11.33; 2.96) | 0.6667 | 17.79 (14.59 – 20.54; 4.21) | 1 | 12.32 (11.72 – 23.89; 1.8) | 1 | 23.42 (23.42 – 23.42; 0) | 1 | 21.6 (18.45 – 27.2; 8.29) | 0.8857 | 16.47 (14.26 – 18.67; 6.53) | 0.6667 |
| **% time COx_R > 0.3** | 9.6 (8.92 – 10.27; 1.99) |  | 12.17 (12.17 – 12.17; 0) |  | 20.37 (18.52 – 22.45; 3.24) |  | 14.82 (10.94 – 25.61; 6.35) |  | 9.3 (8.78 – 9.82; 1.55) |  | 17.93 (11.54 – 19.11; 9.48) |  | 12.17 (10.21 – 31.17; 5.8) |  | 20.37 (20.37 – 20.37; 0) |  | 22.45 (19.49 – 23.32; 2.43) |  | 12.68 (11.61 – 13.75; 3.17) |  |
| **% time COx-a_L > 0** | 58.28 (54.83 – 61.72; 10.22) | 0.6667 | 55.68 (55.68 – 55.68; 0) | 1 | 62.6 (60.26 – 64.19; 3.09) | 0.7104 | 62.78 (55.98 – 66.03; 10.08) | 0.8413 | 64.29 (63.85 – 64.73; 1.3) | 1 | 60.52 (60 – 63.41; 4.3) | 0.6905 | 65.17 (60.43 – 69.24; 12.06) | 1 | 62.6 (62.6 – 62.6; 0) | 1 | 65.25 (61.2 – 67.51; 5.54) | 1 | 59.38 (57.68 – 61.08; 5.04) | 0.6667 |
| **% time COx-a_R > 0** | 62.34 (57.03 – 67.66; 15.76) |  | 53.44 (53.44 – 53.44; 0) |  | 57.58 (54.26 – 66.85; 12.39) |  | 59.65 (54.09 – 73.93; 11.76) |  | 59.02 (52.05 – 66; 20.68) |  | 51.52 (48.55 – 67.76; 9.56) |  | 72.97 (63.2 – 78.69; 16.95) |  | 57 (57 – 57; 0) |  | 61.76 (56.11 – 67.94; 10.54) |  | 56.87 (55.48 – 58.26; 4.12) |  |
| **% time COx-a_L > 0.2** | 28.87 (24.27 – 33.47; 13.64) | 1 | 25 (25 – 25; 0) | 1 | 33.85 (32.26 – 36.21; 2.82) | 0.4557 | 31.75 (25.08 – 32.9; 9.89) | 0.6905 | 35.94 (34.87 – 37.01; 3.17) | 1 | 32.33 (32.19 – 33.8; 2.18) | 0.8413 | 38.08 (31.54 – 42.34; 12.65) | 1 | 35.75 (35.75 – 35.75; 0) | 1 | 34.78 (29.59 – 40.37; 12.6) | 0.4857 | 28.41 (26.75 – 30.08; 4.95) | 0.3333 |
| **% time COx-a_R > 0.2** | 30.88 (25 – 36.76; 17.44) |  | 22.39 (22.39 – 22.39; 0) |  | 28.67 (27.55 – 36.77; 5.72) |  | 24.11 (19.12 – 37.91; 10.11) |  | 30.65 (24.66 – 36.65; 17.78) |  | 27.83 (22.46 – 41.02; 13.6) |  | 42.64 (32.52 – 49.19; 19.41) |  | 28.67 (28.67 – 28.67; 0) |  | 29.9 (25.23 – 33.87; 7.89) |  | 20.7 (19 – 22.41; 5.05) |  |
| **% time COx-a_L > 0.3** | 17.4 (13.67 – 21.12; 11.06) | 1 | 17.05 (17.05 – 17.05; 0) | 1 | 23.55 (19.43 – 24.02; 1.47) | 0.62 | 19.9 (14.83 – 20.21; 7.53) | 0.6905 | 23.58 (22.94 – 24.21; 1.89) | 1 | 22.3 (16.31 – 22.56; 1.91) | 1 | 24.85 (20.95 – 31.24; 11.57) | 1 | 23.55 (23.55 – 23.55; 0) | 1 | 22.17 (17.41 – 28.5; 10.75) | 0.6857 | 17.52 (16.17 – 18.86; 3.99) | 0.3333 |
| **% time COx-a_R > 0.3** | 17.89 (13.53 – 22.25; 12.93) |  | 13.23 (13.23 – 13.23; 0) |  | 20.61 (18.59 – 24.52; 3.19) |  | 14.11 (9.17 – 24.41; 8.79) |  | 20.17 (16.95 – 23.39; 9.54) |  | 18.72 (13.73 – 27.59; 12.72) |  | 26.61 (19.92 – 35.7; 19.83) |  | 18.45 (18.45 – 18.45; 0) |  | 21.03 (17.75 – 22.19; 2.82) |  | 11.14 (9.66 – 12.62; 4.4) |  |
| *The p-values in the table are derived using Mann-Whitney U test between the bilateral signals.*  *ABP, arterial blood pressure; au, arbitrary units; CPP, cerebral perfusion pressure; COx, cerebral oximetry index with CPP; COx-a, cerebral oximetry index with ABP; CT, computed tomography; DAI, diffuse axonal injury; EDH, epidural hematoma; MAD, median absolute deviation; IQR, interquartile range; mmHg, millimeters of mercury; rSO2, regional cerebral oxygen saturation; tSAH, traumatic subarachnoid hemorrhage; SDH, subdural hematoma; aSDH, acute subdural hematoma; TBI-GL, traumatic brain injury patient group without left frontal lobe pathology.* | | | | | | | | | | | | | | | | | | | | |

File S8k: Sub-grouped Physiologic Results using 10-Second, 1-Minute, and 5-Minute Data Resolutions for TBI-GR Population

| **Physiologic Variable** | **Sub-groups** | | | | | | | | | | | | | | | | | |
| --- | --- | --- | --- | --- | --- | --- | --- | --- | --- | --- | --- | --- | --- | --- | --- | --- | --- | --- |
|  | **Age < 40 [n = 4]** | | **Age 40 – 60 [n = 3]** | | **Age > 60 [n = 4]** | | **Males [n = 7]** | | **Females [n = 4]** | | **Focal Injury (aSDH, SDH, EDH, or Contusion) [n = 10]** | | **Diffuse Injury (DAI or tSAH) [n = 1]** | | **Marshall CT V [n = 8]** | | **Marshall CT IV [n = 2]** | |
|  | **Median (IQR) or  Median (IQR; MAD)** | **p-value** | **Median (IQR) or  Median (IQR; MAD)** | **p-value** | **Median (IQR) or  Median (IQR; MAD)** | **p-value** | **Median (IQR) or  Median (IQR; MAD)** | **p-value** | **Median (IQR) or  Median (IQR; MAD)** | **p-value** | **Median (IQR) or  Median (IQR; MAD)** | **p-value** | **Median (IQR) or  Median (IQR; MAD)** | **p-value** | **Median (IQR) or  Median (IQR; MAD)** | **p-value** | **Median (IQR) or  Median (IQR; MAD)** | **p-value** |
| **10-Second Data Resolution** | | | | | | | | | | | | | | | | | | |
| **ABP (mmHg)** | 80.21 (75.6 – 85.24) | – | 89.16 (83.77 – 95.05) | – | 77.75 (74.14 – 81.79) | – | 81.52 (76.74 – 87.29) | – | 75.51 (72.14 – 79.83) | – | 78.64 (75.1 – 82.39) | – | 84.01 (79.65 – 92.97) | – | 76.87 (72.84 – 81.43) | – | 85.91 (80.75 – 91.75) | – |
| **CPP (mmHg)** | 70.5 (67.16 – 75.89) |  | 75.92 (67.42 – 82.04) |  | 70.12 (64.07 – 76.93) |  | 74.25 (67.42 – 82.04) |  | 68.08 (64.91 – 72.01) |  | 70.85 (65.12 – 76.27) |  | 72.91 (69.78 – 83.17) |  | 68.08 (64.91 – 72.01) |  | 75.09 (70.22 – 81.1) |  |
| **rSO2_L (%)** | 77.2 (72.49 – 84.66) | 0.5614 | 69.99 (66.99 – 71.99) | 0.7 | 77.73 (76 – 80.75) | 0.6857 | 75.47 (74 – 79.5) | 0.8048 | 73 (69.5 – 78.99) | 0.4857 | 73 (69.5 – 78.25) | 1 | 82.4 (74.99 – 88.33) | 1 | 74.74 (72 – 80.25) | 0.5283 | 68.84 (67.13 – 70.63) | 0.3333 |
| **rSO2_R (%)** | 80.5 (74.06 – 87) |  | 76.65 (72.99 – 78.99) |  | 71.48 (68.49 – 74.69) |  | 77.68 (72.99 – 78.99) |  | 65.99 (61.79 – 74.49) |  | 76.31 (71.06 – 78.69) |  | 83 (78.99 – 87) |  | 72.98 (68.79 – 77.69) |  | 77.84 (72.93 – 83) |  |
| **COx_L (au)** | 0 (-0.11 – 0.24) | 0.0907 | -0.06 (-0.31 – 0.13) | 1 | 0.03 (-0.12 – 0.22) | 0.8857 | 0 (-0.13 – 0.22) | 0.5224 | 0 (-0.07 – 0.21) | 0.3562 | 0 (-0.11 – 0.21) | 0.3811 | 0 (-0.21 – 0.2) | 1 | 0 (-0.11 – 0.21) | 0.4884 | -0.01 (-0.21 – 0.17) | 1 |
| **COx_R (au)** | -0.01 (-0.2 – 0.15) |  | -0.02 (-0.25 – 0.09) |  | 0.03 (-0.1 – 0.24) |  | -0.02 (-0.25 – 0.14) |  | 0 (-0.18 – 0.15) |  | 0 (-0.19 – 0.15) |  | -0.04 (-0.32 – 0.14) |  | 0 (-0.13 – 0.16) |  | -0.02 (-0.27 – 0.11) |  |
| **COx-a_L (au)** | 0 (-0.1 – 0.25) | 1 | -0.03 (-0.25 – 0.14) | 1 | 0.03 (-0.12 – 0.22) | 0.8857 | 0 (-0.14 – 0.22) | 1 | 0 (-0.08 – 0.22) | 1 | 0 (-0.11 – 0.2) | 0.7869 | 0 (-0.17 – 0.24) | – | 0 (-0.11 – 0.2) | 0.784 | 0.05 (-0.17 – 0.22) | 1 |
| **COx-a_R (au)** | 0 (-0.12 – 0.2) |  | -0.01 (-0.21 – 0.11) |  | 0.01 (-0.12 – 0.24) |  | 0 (-0.15 – 0.19) |  | 0 (-0.14 – 0.19) |  | 0 (-0.14 – 0.18) |  | 0 (-0.24 – 0.22) |  | 0 (-0.14 – 0.18) |  | 0 (-0.15 – 0.18) |  |
| **MAD of ABP (mmHg)** | 7.64 (7.11 – 7.91) | – | 8.26 (6.58 – 10.65) | – | 6.12 (5.38 – 6.91) | – | 8.01 (6.99 – 8.21) | – | 5.9 (5.51 – 6.53) | – | 6.99 (5.8 – 8.12) | – | 7.83 (7.83 – 7.83) | – | 6.32 (5.51 – 7.58) | – | 8.21 (8.18 – 8.23) | – |
| **MAD of CPP (mmHg)** | 6.75 (5.94 – 7.35) |  | 7.94 (6.68 – 10.76) |  | 8.54 (6.16 – 11.35) |  | 7.94 (7.52 – 11.83) |  | 5.81 (5.27 – 6.23) |  | 7.52 (6.06 – 10.14) |  | 6.3 (6.3 – 6.3) |  | 6.7 (5.86 – 11.35) |  | 7.89 (7.87 – 7.92) |  |
| **MAD of rSO2_L (%)** | 6.66 (3.79 – 9.04) | 0.6857 | 4.45 (3.84 – 5.2) | 1 | 2.98 (2.97 – 3.17) | 0.3065 | 3.23 (2.97 – 4.06) | 0.5644 | 5.19 (4.07 – 6.83) | 0.3836 | 3.45 (2.97 – 4.44) | 0.2721 | 9.5 (9.5 – 9.5) | 1 | 4.05 (2.99 – 4.82) | 0.4932 | 2.54 (2.2 – 2.89) | 0.6667 |
| **MAD of rSO2_R (%)** | 7.42 (5.39 – 9.99) |  | 4.2 (3.07 – 7.99) |  | 4.43 (3.87 – 4.45) |  | 4.2 (2.97 – 4.43) |  | 7.42 (5.57 – 9.62) |  | 4.43 (3.84 – 7.78) |  | 5.95 (5.95 – 5.95) |  | 4.43 (4.08 – 5.56) |  | 7.6 (4.77 – 10.43) |  |
| **MAD of COx_L (au)** | 0.27 (0.24 – 0.3) | 0.6857 | 0.27 (0.23 – 0.3) | 0.7 | 0.27 (0.26 – 0.28) | 0.4857 | 0.27 (0.25 – 0.29) | 0.62 | 0.27 (0.23 – 0.3) | 0.8857 | 0.27 (0.24 – 0.29) | 0.7394 | 0.31 (0.31 – 0.31) | 1 | 0.25 (0.24 – 0.28) | 0.9591 | 0.28 (0.28 – 0.29) | 1 |
| **MAD of COx_R (au)** | 0.26 (0.23 – 0.3) |  | 0.29 (0.26 – 0.31) |  | 0.29 (0.27 – 0.31) |  | 0.29 (0.26 – 0.31) |  | 0.26 (0.22 – 0.31) |  | 0.29 (0.22 – 0.3) |  | 0.36 (0.36 – 0.36) |  | 0.26 (0.22 – 0.3) |  | 0.29 (0.28 – 0.29) |  |
| **MAD of COx-a_L (au)** | 0.27 (0.23 – 0.31) | 0.6857 | 0.26 (0.22 – 0.29) | 0.7 | 0.27 (0.26 – 0.27) | 1 | 0.27 (0.25 – 0.29) | 0.62 | 0.25 (0.21 – 0.29) | 1 | 0.27 (0.23 – 0.28) | 0.6842 | 0.3 (0.3 – 0.3) | 1 | 0.25 (0.23 – 0.27) | 0.9591 | 0.29 (0.27 – 0.3) | 0.6667 |
| **MAD of COx-a_R (au)** | 0.23 (0.22 – 0.26) |  | 0.27 (0.23 – 0.3) |  | 0.27 (0.26 – 0.28) |  | 0.26 (0.23 – 0.28) |  | 0.24 (0.2 – 0.29) |  | 0.25 (0.23 – 0.28) |  | 0.34 (0.34 – 0.34) |  | 0.25 (0.22 – 0.28) |  | 0.25 (0.24 – 0.26) |  |
| **% time rSO2_L > 60%** | 99.95 (99.07 – 100; 0.07) | 0.6573 | 98.88 (95.65 – 99.36; 1.43) | 1 | 100 (93.98 – 100; 0) | 0.1832 | 100 (99.39 – 100; 0) | 0.121 | 98.21 (91.41 – 99.88; 2.54) | 0.3836 | 99.87 (97.15 – 100; 0.19) | 0.1198 | 100 (100 – 100; 0) | – | 99.92 (95.53 – 100; 0.12) | 0.125 | 99.39 (99.14 – 99.65; 0.76) | 0.6667 |
| **% time rSO2_R > 60%** | 99.39 (96.68 – 99.97; 0.87) |  | 97.58 (76.95 – 98.72; 3.38) |  | 96.92 (87.05 – 98.65; 3.22) |  | 98.82 (97.9 – 99.91; 1.68) |  | 75.78 (60.05 – 92.7; 25.16) |  | 97.9 (91.61 – 99.6; 3.07) |  | 100 (100 – 100; 0) |  | 96.61 (83.02 – 98.65; 4.98) |  | 99.34 (99.08 – 99.6; 0.77) |  |
| **% time rSO2_L > 70%** | 85.96 (58.1 – 100; 20.82) | 0.8824 | 44.25 (40.9 – 53.92; 9.95) | 0.7 | 99.47 (74.33 – 99.96; 0.75) | 0.4857 | 98.99 (40.9 – 99.98; 1.5) | 0.8982 | 67.75 (47.79 – 78.94; 26.99) | 0.5614 | 67.75 (39.22 – 99.71; 47.02) | 0.8501 | 100 (100 – 100; 0) | – | 85.45 (58.76 – 99.96; 21.57) | 0.3717 | 27.1 (21.88 – 32.32; 15.48) | 0.3333 |
| **% time rSO2_R > 70%** | 84.54 (63.57 – 99.96; 22.87) |  | 84.39 (56.04 – 92.12; 22.91) |  | 57.46 (22.55 – 88.68; 51.87) |  | 84.92 (76.77 – 99.89; 22.27) |  | 37.25 (20.8 – 60.12; 34.59) |  | 76.77 (34.21 – 96.11; 34.39) |  | 100 (100 – 100; 0) |  | 65.61 (29.42 – 88.67; 50.93) |  | 84.5 (76.82 – 92.17; 22.76) |  |
| **% time rSO2_L > 80%** | 39.71 (19.46 – 55.21; 25.55) | 0.4857 | 3.21 (1.61 – 4.21; 2.96) | 0.7 | 32.39 (13.21 – 56.04; 34.96) | 0.5614 | 17.61 (1.61 – 53.79; 26.12) | 0.62 | 15.58 (3.9 – 32.83; 19.24) | 0.8824 | 11.41 (0.81 – 41.86; 16.92) | 0.9094 | 53.47 (53.47 – 53.47; 0) | 1 | 21.78 (4.71 – 50.48; 29.92) | 0.5624 | 0.01 (0 – 0.01; 0.01) | 0.3333 |
| **% time rSO2_R > 80%** | 56.69 (38.61 – 73.76; 36.31) |  | 14.96 (7.48 – 16.64; 4.99) |  | 5.74 (0.57 – 25.32; 7.95) |  | 18.32 (12.84 – 58.07; 26.04) |  | 6.65 (0 – 26.56; 9.86) |  | 14.13 (3.25 – 39.86; 20.39) |  | 66.34 (66.34 – 66.34; 0) |  | 12.02 (0.57 – 28.5; 17.25) |  | 32.68 (25.5 – 39.86; 21.29) |  |
| **% time rSO2_L > 90%** | 5.58 (0 – 14.66; 8.28) | 0.6573 | 0 (0 – 0; 0) | 0.1967 | 1.79 (0 – 12.12; 2.66) | 0.6446 | 0 (0 – 14.37; 0) | 0.6012 | 0 (0 – 2.79; 0) | 1 | 0 (0 – 2.69; 0) | 0.4818 | 11.17 (11.17 – 11.17; 0) | 1 | 0 (0 – 8.98; 0) | 1 | 0 (0 – 0; 0) | 0.2207 |
| **% time rSO2_R > 90%** | 11.42 (2.03 – 24.46; 14.92) |  | 0.01 (0 – 0.12; 0.01) |  | 0.04 (0 – 0.08; 0.06) |  | 0.1 (0.04 – 10.18; 0.14) |  | 0 (0 – 0.68; 0) |  | 0.04 (0 – 0.19; 0.06) |  | 2.71 (2.71 – 2.71; 0) |  | 0 (0 – 0.08; 0.01) |  | 10.18 (5.2 – 15.15; 14.76) |  |
| **% time COx_L > 0** | 49.52 (48 – 52.67; 3.66) | **0.0286** | 37.05 (33.6 – 40.97; 10.21) | 0.7 | 55.44 (49.84 – 60.58; 10.57) | 0.4857 | 50.08 (39.76 – 55.44; 12.6) | 0.3829 | 47.05 (45.08 – 53.36; 3.01) | 0.2 | 49.52 (43.08 – 57.01; 11.95) | 0.123 | 45.14 (45.14 – 45.14; 0) | 1 | 49.52 (44.29 – 53.87; 8.65) | 0.1605 | 45.3 (37.73 – 52.87; 22.44) | 1 |
| **% time COx_R > 0** | 39.63 (36.11 – 42.71; 5.43) |  | 33.15 (30.32 – 37.19; 8.4) |  | 47.66 (40.55 – 56.3; 11.22) |  | 41 (36.17 – 41.67; 2.67) |  | 40.82 (34.74 – 46.94; 12.6) |  | 41.11 (34.66 – 43.89; 8.4) |  | 37.15 (37.15 – 37.15; 0) |  | 41.56 (37.69 – 46.94; 8.4) |  | 37.1 (35.04 – 39.17; 6.12) |  |
| **% time COx_L > 0.2** | 27.99 (25.46 – 31.41; 3.87) | **0.0286** | 18.98 (16.66 – 22.24; 6.88) | 0.7 | 27.37 (25.29 – 32.04; 5.8) | 1 | 27.12 (19.38 – 29.03; 10.61) | 0.7104 | 25.51 (25.43 – 30.48; 0.22) | 0.2 | 26.33 (21.21 – 29.74; 7.91) | 0.2799 | 25.24 (25.24 – 25.24; 0) | 1 | 26.33 (24.07 – 28.32; 4.01) | 0.3282 | 24.31 (19.33 – 29.29; 14.78) | 1 |
| **% time COx_R > 0.2** | 21.7 (19.93 – 22.93; 2.91) |  | 17.86 (15.62 – 19.03; 3.48) |  | 28.97 (21.35 – 36.76; 11.92) |  | 20.2 (18.97 – 23.45; 3.48) |  | 21.7 (19.24 – 25.68; 6.54) |  | 20.99 (18.41 – 24.4; 5.39) |  | 21.2 (21.2 – 21.2; 0) |  | 21.98 (19.52 – 27.89; 5.39) |  | 18.17 (17.15 – 19.19; 3.02) |  |
| **% time COx_L > 0.3** | 20.25 (17.86 – 22.29; 3.44) | 0.0571 | 12.97 (11.23 – 15.3; 5.15) | 1 | 17.31 (15.6 – 22.3; 3.79) | 1 | 16.45 (13.02 – 20.09; 5.16) | 0.8048 | 18.06 (17.22 – 22.53; 1.86) | 0.2 | 17.04 (13.79 – 21.05; 5.96) | 0.4359 | 18.48 (18.48 – 18.48; 0) | 1 | 17.04 (15.25 – 19.14; 3.79) | 0.5054 | 16.31 (12.9 – 19.72; 10.1) | 1 |
| **% time COx_R > 0.3** | 15.04 (13.36 – 16.39; 2.86) |  | 12.61 (11.08 – 13.09; 1.44) |  | 21.47 (14.84 – 27.74; 9.37) |  | 13.64 (13.09 – 16.69; 2.38) |  | 15.04 (13.09 – 18.83; 4.65) |  | 13.95 (12.85 – 17.41; 3.47) |  | 15.81 (15.81 – 15.81; 0) |  | 14.76 (13.38 – 20.52; 4.1) |  | 12.1 (11.36 – 12.84; 2.2) |  |
| **% time COx-a_L > 0** | 48.83 (47.95 – 53.74; 1.76) | 0.2 | 38.89 (38.16 – 41.32; 2.15) | 0.4 | 55.35 (48.68 – 59.75; 7.69) | 0.8857 | 49.41 (39.18 – 55.35; 14.72) | 0.9015 | 47.64 (46.21 – 51.72; 3.34) | 0.4857 | 48.83 (40.55 – 57.15; 14.3) | 0.4359 | 47.03 (47.03 – 47.03; 0) | 1 | 48.83 (42.68 – 53.55; 10.7) | 0.3823 | 52.09 (44.76 – 59.42; 21.73) | 1 |
| **% time COx-a_R > 0** | 44.08 (43.26 – 45.62; 2.4) |  | 36.53 (35.83 – 39.78; 2.06) |  | 48.75 (45.18 – 55.73; 7.17) |  | 43.04 (41.12 – 48.29; 5.05) |  | 44.08 (42.17 – 45.84; 5.18) |  | 43.55 (41 – 49.21; 7.03) |  | 44.11 (44.11 – 44.11; 0) |  | 42.71 (39.78 – 47.6; 7.36) |  | 46.58 (44.81 – 48.35; 5.25) |  |
| **% time COx-a_L > 0.2** | 29 (27.26 – 32.22; 4.42) | 0.2 | 20.39 (18.51 – 22.26; 5.57) | 0.7 | 27.15 (24.42 – 30.78; 7.08) | 0.8857 | 26.47 (19.33 – 28.66; 9.02) | 1 | 26.32 (23.99 – 31.28; 3.69) | 0.4857 | 25.31 (21.17 – 29.08; 6.75) | 0.7394 | 28.5 (28.5 – 28.5; 0) | 1 | 25.31 (22.75 – 28.24; 4.97) | 0.7209 | 28.5 (22.57 – 34.44; 17.6) | 1 |
| **% time COx-a_R > 0.2** | 25.31 (23.42 – 26.46; 1.81) |  | 18.94 (17.55 – 19.7; 2.25) |  | 28.38 (23.91 – 35.48; 7.54) |  | 24.23 (21.27 – 25.6; 3.63) |  | 23.67 (19.76 – 27.85; 7.57) |  | 23.16 (20.58 – 26.14; 4.62) |  | 26.38 (26.38 – 26.38; 0) |  | 23.16 (20.46 – 26.45; 4.76) |  | 23.57 (22.01 – 25.12; 4.62) |  |
| **% time COx-a_L > 0.3** | 20.4 (18.44 – 22.7; 4.86) | 0.2 | 13.78 (12.19 – 15.41; 4.71) | 0.4 | 17.31 (15.56 – 20.67; 4.59) | 0.8857 | 16.91 (12.65 – 19.38; 6.12) | 0.9015 | 18.4 (16.4 – 22.21; 3.91) | 0.4857 | 16.97 (13.96 – 20.21; 5.38) | 0.7394 | 19.76 (19.76 – 19.76; 0) | 1 | 16.97 (14.31 – 18.54; 4.21) | 0.7209 | 19.13 (14.87 – 23.4; 12.64) | 1 |
| **% time COx-a_R > 0.3** | 17.83 (16.5 – 18.24; 0.96) |  | 13.22 (11.7 – 13.34; 0.36) |  | 20.32 (15.94 – 26.43; 6.87) |  | 16.2 (14.31 – 17.83; 2.68) |  | 15.99 (12.33 – 20.31; 6.49) |  | 15.68 (13.28 – 17.92; 3.55) |  | 18.94 (18.94 – 18.94; 0) |  | 15.68 (13.18 – 19.34; 3.78) |  | 15.74 (14.6 – 16.88; 3.37) |  |
| **1-Minute Data Resolution** | | | | | | | | | | | | | | | | | | |
| **ABP (mmHg)** | 80.24 (75.72 – 85.16) | – | 89.11 (83.92 – 95.09) | – | 77.8 (74.16 – 81.66) | – | 81.65 (77.14 – 87.14) | – | 75.5 (72.18 – 79.79) | – | 78.69 (75.12 – 82.26) | – | 84.2 (79.76 – 92.75) | – | 76.93 (72.9 – 81.34) | – | 85.88 (80.95 – 91.74) | – |
| **CPP (mmHg)** | 70.49 (67.26 – 75.77) |  | 75.97 (67.64 – 82.26) |  | 70.1 (64.09 – 76.88) |  | 74.38 (67.64 – 82.26) |  | 68.11 (64.98 – 71.95) |  | 70.82 (65.14 – 76.23) |  | 72.91 (69.9 – 83.07) |  | 68.11 (64.98 – 71.95) |  | 75.17 (70.38 – 81.15) |  |
| **rSO2_L (%)** | 77.14 (72.61 – 84.49) | 0.4857 | 69.98 (66.91 – 72.23) | 0.7 | 77.73 (75.96 – 80.74) | 0.6857 | 75.39 (73.92 – 79.48) | 0.8048 | 73 (69.5 – 78.83) | 0.4857 | 73 (69.5 – 78.24) | 1 | 82.3 (75.24 – 88.32) | 1 | 74.69 (71.95 – 80.07) | 0.5737 | 68.92 (67.11 – 70.58) | 0.3333 |
| **rSO2_R (%)** | 80.5 (73.98 – 87.04) |  | 76.59 (73.1 – 78.99) |  | 71.34 (68.47 – 74.71) |  | 77.65 (73.1 – 79.07) |  | 65.95 (61.78 – 74.45) |  | 76.17 (71.05 – 78.69) |  | 82.99 (78.72 – 87.08) |  | 72.87 (68.71 – 77.65) |  | 77.82 (72.95 – 83.03) |  |
| **COx_L (au)** | 0.06 (-0.13 – 0.25) | 0.2 | -0.09 (-0.32 – 0.13) | 1 | 0.05 (-0.12 – 0.23) | 0.8857 | 0.05 (-0.14 – 0.21) | 0.62 | 0.07 (-0.12 – 0.25) | 0.3429 | 0.05 (-0.13 – 0.24) | 0.315 | 0 (-0.23 – 0.23) | 1 | 0.05 (-0.12 – 0.24) | 0.3823 | -0.03 (-0.22 – 0.17) | 1 |
| **COx_R (au)** | -0.03 (-0.26 – 0.19) |  | -0.1 (-0.29 – 0.12) |  | 0.07 (-0.14 – 0.27) |  | -0.05 (-0.26 – 0.15) |  | -0.03 (-0.22 – 0.19) |  | -0.02 (-0.22 – 0.18) |  | -0.08 (-0.34 – 0.17) |  | 0.01 (-0.19 – 0.21) |  | -0.07 (-0.29 – 0.14) |  |
| **COx-a_L (au)** | 0.07 (-0.12 – 0.29) | 0.3429 | -0.06 (-0.27 – 0.15) | 1 | 0.05 (-0.13 – 0.22) | 0.8857 | 0.04 (-0.15 – 0.21) | 1 | 0.05 (-0.13 – 0.26) | 0.2 | 0.05 (-0.13 – 0.22) | 0.5787 | 0.02 (-0.19 – 0.26) | 1 | 0.05 (-0.13 – 0.22) | 0.5054 | 0.03 (-0.17 – 0.22) | 1 |
| **COx-a_R (au)** | 0.03 (-0.17 – 0.24) |  | -0.03 (-0.22 – 0.15) |  | 0.05 (-0.16 – 0.27) |  | 0.01 (-0.2 – 0.2) |  | 0.01 (-0.19 – 0.21) |  | 0.01 (-0.19 – 0.2) |  | 0 (-0.25 – 0.23) |  | 0.01 (-0.19 – 0.2) |  | 0.02 (-0.17 – 0.21) |  |
| **MAD of ABP (mmHg)** | 7.63 (7.06 – 7.89) | – | 8.2 (6.45 – 10.56) | – | 5.9 (5.03 – 6.81) | – | 7.98 (6.92 – 8.11) | – | 5.69 (5.22 – 6.45) | – | 6.92 (5.54 – 8) | – | 7.85 (7.85 – 7.85) | – | 6.21 (5.22 – 7.56) | – | 8.11 (8.06 – 8.15) | – |
| **MAD of CPP (mmHg)** | 6.53 (5.61 – 7.28) |  | 7.92 (6.72 – 10.58) |  | 7.97 (5.95 – 10.71) |  | 7.92 (7.4 – 11.45) |  | 5.71 (5.33 – 5.91) |  | 7.4 (5.9 – 9.45) |  | 5.9 (5.9 – 5.9) |  | 6.57 (5.79 – 10.71) |  | 7.78 (7.7 – 7.85) |  |
| **MAD of rSO2_L (%)** | 6.61 (3.76 – 8.93) | 0.6857 | 4.43 (3.95 – 5.25) | 1 | 3.08 (2.93 – 3.32) | 0.3429 | 3.48 (2.9 – 4.05) | 0.535 | 5.25 (4.12 – 6.89) | 0.6857 | 3.58 (3.02 – 4.43) | 0.315 | 9.35 (9.35 – 9.35) | 1 | 4.05 (3.14 – 4.84) | 0.5737 | 2.62 (2.19 – 3.05) | 0.6667 |
| **MAD of rSO2_R (%)** | 7.54 (5.47 – 10.03) |  | 4.23 (3.05 – 8.03) |  | 4.2 (3.62 – 4.37) |  | 4.11 (2.92 – 4.42) |  | 7.54 (5.62 – 9.72) |  | 4.26 (3.79 – 7.91) |  | 6.06 (6.06 – 6.06) |  | 4.26 (4 – 5.71) |  | 7.46 (4.66 – 10.27) |  |
| **MAD of COx_L (au)** | 0.29 (0.28 – 0.32) | 0.4857 | 0.29 (0.29 – 0.31) | 1 | 0.28 (0.27 – 0.29) | 0.1143 | 0.28 (0.28 – 0.3) | **0.0379** | 0.3 (0.28 – 0.32) | 0.8857 | 0.28 (0.28 – 0.3) | 0.0753 | 0.34 (0.34 – 0.34) | 1 | 0.28 (0.28 – 0.31) | 0.1605 | 0.29 (0.28 – 0.29) | 0.3333 |
| **MAD of COx_R (au)** | 0.34 (0.32 – 0.35) |  | 0.3 (0.29 – 0.33) |  | 0.32 (0.3 – 0.34) |  | 0.33 (0.3 – 0.34) |  | 0.32 (0.28 – 0.36) |  | 0.32 (0.3 – 0.34) |  | 0.38 (0.38 – 0.38) |  | 0.32 (0.29 – 0.35) |  | 0.32 (0.31 – 0.32) |  |
| **MAD of COx-a_L (au)** | 0.3 (0.28 – 0.31) | 1 | 0.29 (0.29 – 0.31) | 0.7 | 0.28 (0.27 – 0.29) | 0.8857 | 0.29 (0.28 – 0.3) | 0.9015 | 0.3 (0.28 – 0.31) | 1 | 0.29 (0.28 – 0.3) | 0.8501 | 0.33 (0.33 – 0.33) | 1 | 0.29 (0.27 – 0.31) | 0.8747 | 0.29 (0.29 – 0.29) | 0.3333 |
| **MAD of COx-a_R (au)** | 0.31 (0.27 – 0.34) |  | 0.28 (0.28 – 0.32) |  | 0.29 (0.28 – 0.31) |  | 0.28 (0.28 – 0.32) |  | 0.3 (0.27 – 0.34) |  | 0.28 (0.28 – 0.32) |  | 0.36 (0.36 – 0.36) |  | 0.29 (0.27 – 0.33) |  | 0.28 (0.28 – 0.28) |  |
| **% time rSO2_L > 60%** | 99.92 (99.04 – 100; 0.12) | 0.6573 | 98.86 (95.61 – 99.3; 1.32) | 1 | 100 (94.32 – 100; 0) | 0.3562 | 100 (99.35 – 100; 0) | 0.2395 | 98.18 (91.77 – 99.81; 2.51) | 0.3836 | 99.8 (97.17 – 100; 0.3) | 0.1826 | 100 (100 – 100; 0) | – | 99.87 (95.55 – 100; 0.19) | 0.1827 | 99.35 (99.11 – 99.6; 0.73) | 1 |
| **% time rSO2_R > 60%** | 99.43 (96.85 – 99.95; 0.8) |  | 97.63 (77.14 – 98.74; 3.28) |  | 96.99 (87.61 – 98.65; 3.13) |  | 98.92 (97.91 – 99.89; 1.51) |  | 76.87 (61.49 – 92.98; 25.19) |  | 97.91 (91.93 – 99.61; 3.05) |  | 100 (100 – 100; 0) |  | 96.71 (83.76 – 98.63; 4.84) |  | 99.38 (99.15 – 99.61; 0.68) |  |
| **% time rSO2_L > 70%** | 86.62 (59.38 – 100; 19.83) | 0.8824 | 46.59 (43.17 – 55.23; 10.16) | 0.7 | 99.61 (74.51 – 100; 0.58) | 0.4596 | 99.22 (43.17 – 100; 1.16) | 0.9485 | 68.56 (48 – 79.93; 26.78) | 0.5614 | 68.56 (41.45 – 99.8; 46.03) | 0.8794 | 100 (100 – 100; 0) | – | 86.23 (59.55 – 100; 20.41) | 0.3973 | 28.77 (23.28 – 34.26; 16.27) | 0.3333 |
| **% time rSO2_R > 70%** | 84.57 (63.81 – 99.92; 22.8) |  | 84.53 (56.15 – 92.17; 22.64) |  | 57.89 (22.73 – 89.16; 51.72) |  | 85.54 (76.88 – 99.85; 21.28) |  | 37.66 (20.89 – 60.65; 35.08) |  | 76.88 (34.56 – 96.24; 34.19) |  | 100 (100 – 100; 0) |  | 66.03 (29.62 – 89.13; 50.28) |  | 84.52 (76.88 – 92.16; 22.66) |  |
| **% time rSO2_L > 80%** | 40.36 (20.12 – 55.69; 25.4) | 0.4857 | 3.96 (1.98 – 4.86; 2.69) | 0.5066 | 35.87 (14.76 – 59.93; 38.6) | 0.5614 | 19.68 (1.98 – 56.58; 29.17) | 0.7012 | 16.3 (4.33 – 33.59; 19.88) | 0.8824 | 12.72 (0.99 – 45.76; 18.86) | 0.909 | 53.89 (53.89 – 53.89; 0) | 1 | 23.25 (5.32 – 54.33; 31.54) | 0.5624 | 0 (0 – 0; 0) | 0.2207 |
| **% time rSO2_R > 80%** | 57.43 (38.93 – 74.68; 36.16) |  | 16.2 (8.1 – 17.67; 4.35) |  | 6.34 (0.6 – 26.58; 8.81) |  | 19.13 (14.04 – 59; 27.19) |  | 6.87 (0 – 27.19; 10.19) |  | 14.97 (3.57 – 40.28; 21.61) |  | 67.53 (67.53 – 67.53; 0) |  | 12.81 (0.6 – 29.82; 18.41) |  | 33.23 (26.18 – 40.28; 20.9) |  |
| **% time rSO2_L > 90%** | 6.34 (0 – 16.1; 9.41) | 0.6573 | 0 (0 – 0; 0) | 0.505 | 1.86 (0 – 13.14; 2.76) | 0.6446 | 0 (0 – 15.03; 0) | 0.7903 | 0 (0 – 3.17; 0) | 1 | 0 (0 – 2.79; 0) | 0.6697 | 12.69 (12.69 – 12.69; 0) | 1 | 0 (0 – 9.37; 0) | 0.8093 | 0 (0 – 0; 0) | 0.2207 |
| **% time rSO2_R > 90%** | 11.85 (2.46 – 25.1; 15.14) |  | 0 (0 – 0.18; 0) |  | 0.05 (0 – 0.1; 0.07) |  | 0.1 (0.05 – 10.39; 0.14) |  | 0 (0 – 0.82; 0) |  | 0.05 (0 – 0.29; 0.07) |  | 3.27 (3.27 – 3.27; 0) |  | 0 (0 – 0.1; 0) |  | 10.39 (5.37 – 15.41; 14.88) |  |
| **% time COx_L > 0** | 59.25 (55.29 – 62.13; 5.4) | 0.1143 | 39.5 (36.02 – 50.45; 10.34) | 1 | 58 (53.41 – 62.9; 11.64) | 0.8857 | 56.68 (41.56 – 60.34; 11.5) | 0.535 | 59.28 (55.29 – 64.47; 8.67) | 0.3429 | 58.23 (46.88 – 61.39; 6.95) | 0.2475 | 49.7 (49.7 – 49.7; 0) | 1 | 58.23 (53.41 – 62.16; 6.95) | 0.2345 | 46.94 (39.74 – 54.15; 21.37) | 1 |
| **% time COx_R > 0** | 46.72 (39.67 – 53.93; 11.04) |  | 36.5 (35.82 – 40.18; 2.01) |  | 57.81 (48.09 – 65.18; 11.84) |  | 43.86 (38.84 – 53.3; 10.68) |  | 46.72 (38.83 – 56.18; 13.53) |  | 47.46 (38.67 – 55; 12.77) |  | 40.06 (40.06 – 40.06; 0) |  | 52.23 (38.52 – 57.8; 18.81) |  | 41.18 (39.84 – 42.52; 3.97) |  |
| **% time COx_L > 0.2** | 31.43 (28.5 – 35.14; 4.79) | 0.1143 | 19.51 (17.45 – 26.98; 6.12) | 1 | 27.5 (24.9 – 33.92; 6.28) | 0.8857 | 26.53 (19.76 – 31.27; 10.41) | 0.8048 | 31.62 (28.5 – 38.4; 5.07) | 0.2 | 28.64 (21.63 – 34.35; 10.71) | 0.393 | 27.61 (27.61 – 27.61; 0) | 1 | 28.64 (24.9 – 35.42; 10.71) | 0.4418 | 24.73 (20.06 – 29.4; 13.85) | 1 |
| **% time COx_R > 0.2** | 23.95 (21.44 – 27.16; 5.36) |  | 19.21 (17.9 – 19.81; 1.8) |  | 33.09 (24.12 – 41.11; 12.69) |  | 20.42 (19.44 – 29.06; 3.37) |  | 23.95 (21.05 – 29.71; 6.51) |  | 22.9 (19.32 – 30.79; 6.26) |  | 22.53 (22.53 – 22.53; 0) |  | 25.49 (19.56 – 34.53; 9.87) |  | 19.28 (18.71 – 19.85; 1.69) |  |
| **% time COx_L > 0.3** | 20.93 (19.3 – 23.35; 3.21) | 0.2 | 12.99 (11.39 – 17.83; 4.73) | 1 | 17.08 (15.13 – 23.03; 3.91) | 0.8857 | 15.81 (13.04 – 20.18; 4.18) | 0.8048 | 21.26 (19.3 – 26.27; 3.71) | 0.2 | 18.01 (13.76 – 22.5; 7.11) | 0.7394 | 19.85 (19.85 – 19.85; 0) | 1 | 18.01 (15.13 – 23.84; 7.11) | 0.8785 | 15.9 (12.85 – 18.95; 9.05) | 1 |
| **% time COx_R > 0.3** | 16.09 (15.02 – 17.88; 3.12) |  | 13.42 (12.66 – 13.48; 0.18) |  | 23.12 (16.37 – 29.64; 10.89) |  | 13.54 (13.31 – 20.29; 2.43) |  | 16.09 (15.01 – 20.12; 3.13) |  | 14.83 (13.25 – 21.72; 4.09) |  | 16.05 (16.05 – 16.05; 0) |  | 16.77 (13.37 – 24.57; 6.26) |  | 12.72 (12.32 – 13.13; 1.21) |  |
| **% time COx-a_L > 0** | 59.69 (54.95 – 64.31; 7.96) | 0.3429 | 41.17 (40.91 – 50.64; 0.77) | 1 | 57.73 (52.86 – 61.56; 9.54) | 0.8857 | 56.49 (41.56 – 61.27; 14.89) | 0.9015 | 57.95 (54.95 – 62.42; 5.72) | 0.3429 | 57.73 (45.42 – 62.7; 10.86) | 0.4813 | 52.39 (52.39 – 52.39; 0) | 1 | 57.73 (52.34 – 60.97; 6.1) | 0.4418 | 53.59 (47.12 – 60.07; 19.19) | 1 |
| **% time COx-a_R > 0** | 53.42 (52.06 – 55.31; 3) |  | 46.04 (42.44 – 46.49; 1.31) |  | 55.91 (49.93 – 64.42; 10.53) |  | 51.05 (46.3 – 56.5; 7.42) |  | 51.4 (49.29 – 54.73; 4.29) |  | 51.88 (46.65 – 57.69; 8.28) |  | 50.07 (50.07 – 50.07; 0) |  | 51.88 (46.83 – 55.78; 7.62) |  | 52.46 (49.25 – 55.67; 9.52) |  |
| **% time COx-a_L > 0.2** | 33.64 (29.13 – 37.76; 7.27) | 0.4857 | 20.88 (19.23 – 26.57; 4.9) | 0.7 | 27.13 (24.67 – 31.43; 6.62) | 0.8857 | 26.67 (19.76 – 32.28; 11.9) | 0.8048 | 31.28 (29.13 – 34.94; 4.93) | 0.3429 | 27.13 (22.06 – 35.8; 10.91) | 0.7394 | 30.3 (30.3 – 30.3; 0) | 1 | 27.13 (24.43 – 33.44; 8.44) | 0.7209 | 28.84 (23.21 – 34.48; 16.7) | 1 |
| **% time COx-a_R > 0.2** | 29.17 (26.89 – 30.46; 2.07) |  | 20.25 (20.2 – 20.42; 0.15) |  | 31.61 (24.8 – 40.54; 10.51) |  | 25.08 (22.28 – 30.57; 7.16) |  | 25.8 (22.75 – 30.52; 5.81) |  | 24.52 (21.35 – 30.67; 6.41) |  | 27.98 (27.98 – 27.98; 0) |  | 24.52 (22.78 – 32.62; 6.41) |  | 25.48 (23.04 – 27.92; 7.24) |  |
| **% time COx-a_L > 0.3** | 23.73 (19.53 – 26.75; 4.74) | 0.3429 | 13.82 (12.33 – 18.04; 4.44) | 0.7 | 17.29 (15.26 – 21.48; 4.45) | 0.6857 | 16.23 (13.08 – 22.45; 5.79) | 0.7104 | 21.58 (19.53 – 24.42; 5.07) | 0.3429 | 17.29 (14.22 – 25.49; 7.36) | 0.9705 | 20.9 (20.9 – 20.9; 0) | 1 | 17.29 (15.02 – 23.34; 6.24) | 0.9591 | 19.06 (14.94 – 23.17; 12.2) | 1 |
| **% time COx-a_R > 0.3** | 19.88 (18.33 – 20.84; 2.22) |  | 13.48 (12.8 – 13.56; 0.24) |  | 22.89 (17.2 – 29.55; 9.22) |  | 17.72 (14.63 – 21.37; 6.05) |  | 17.22 (14.28 – 21.59; 5.43) |  | 16.67 (13.98 – 21.9; 5.07) |  | 19.44 (19.44 – 19.44; 0) |  | 16.67 (14.67 – 23.84; 5.63) |  | 16.9 (15.19 – 18.6; 5.07) |  |
| **5-Minute Data Resolution** | | | | | | | | | | | | | | | | | | |
| **ABP (mmHg)** | 80.32 (75.95 – 85.19) | – | 89.77 (83.89 – 95.24) | – | 77.8 (74.33 – 81.64) | – | 81.64 (77.42 – 86.81) | – | 75.49 (72.36 – 80) | – | 78.67 (75.37 – 82.25) | – | 83.99 (79.78 – 92.16) | – | 76.92 (73.09 – 81.28) | – | 86.31 (81.17 – 91.86) | – |
| **CPP (mmHg)** | 70.42 (67.45 – 75.8) |  | 76.31 (67.89 – 82.68) |  | 70.03 (64.42 – 76.77) |  | 74.45 (67.89 – 82.6) |  | 68.14 (65.14 – 72.02) |  | 70.72 (65.44 – 76.41) |  | 72.71 (69.95 – 83.03) |  | 68.14 (65.14 – 72.02) |  | 75.38 (70.6 – 81.43) |  |
| **rSO2_L (%)** | 77.04 (72.67 – 84.46) | 0.6857 | 69.9 (66.89 – 72.18) | 0.7 | 77.75 (76.03 – 80.77) | 0.6857 | 75.39 (73.95 – 79.42) | 0.8048 | 72.99 (69.52 – 78.79) | 0.4857 | 72.99 (69.52 – 78.2) | 1 | 82.1 (75.3 – 88.34) | 1 | 74.69 (71.99 – 80.01) | 0.5737 | 68.94 (67.11 – 70.55) | 0.3333 |
| **rSO2_R (%)** | 80.32 (74.09 – 87.05) |  | 76.56 (73.09 – 79.06) |  | 71.38 (68.52 – 74.7) |  | 77.59 (73.09 – 79.08) |  | 65.9 (61.76 – 74.43) |  | 76.16 (71.04 – 78.72) |  | 82.67 (78.92 – 87.08) |  | 72.87 (68.64 – 77.63) |  | 77.78 (72.96 – 83.05) |  |
| **COx_L (au)** | 0.07 (-0.1 – 0.21) | 0.0571 | -0.09 (-0.27 – 0.1) | 0.7 | 0.06 (-0.08 – 0.2) | 0.8857 | 0.05 (-0.1 – 0.18) | 0.4557 | 0.08 (-0.11 – 0.23) | 0.2 | 0.06 (-0.09 – 0.2) | 0.2176 | 0.04 (-0.18 – 0.19) | 1 | 0.06 (-0.09 – 0.2) | 0.2786 | -0.02 (-0.18 – 0.14) | 1 |
| **COx_R (au)** | -0.04 (-0.22 – 0.17) |  | -0.09 (-0.23 – 0.09) |  | 0.08 (-0.1 – 0.24) |  | -0.05 (-0.21 – 0.12) |  | -0.03 (-0.18 – 0.17) |  | -0.02 (-0.19 – 0.15) |  | -0.11 (-0.29 – 0.14) |  | 0.02 (-0.16 – 0.19) |  | -0.07 (-0.24 – 0.11) |  |
| **COx-a_L (au)** | 0.06 (-0.09 – 0.24) | 0.3429 | -0.06 (-0.23 – 0.11) | 1 | 0.05 (-0.09 – 0.19) | 0.8857 | 0.04 (-0.12 – 0.18) | 1 | 0.05 (-0.11 – 0.23) | 0.2 | 0.05 (-0.11 – 0.19) | 0.6305 | 0.02 (-0.16 – 0.21) | 1 | 0.05 (-0.11 – 0.19) | 0.5737 | 0.04 (-0.13 – 0.19) | 1 |
| **COx-a_R (au)** | 0.03 (-0.14 – 0.2) |  | -0.03 (-0.18 – 0.12) |  | 0.05 (-0.11 – 0.25) |  | 0.01 (-0.16 – 0.19) |  | 0 (-0.15 – 0.17) |  | 0.01 (-0.16 – 0.18) |  | -0.04 (-0.2 – 0.19) |  | 0.01 (-0.16 – 0.18) |  | 0.02 (-0.13 – 0.17) |  |
| **MAD of ABP (mmHg)** | 7.32 (6.72 – 7.68) | – | 8.01 (6.31 – 10.36) | – | 5.82 (4.91 – 6.64) | – | 7.59 (6.95 – 7.98) | – | 5.53 (5.14 – 6.07) | – | 6.95 (5.42 – 7.86) | – | 7.04 (7.04 – 7.04) | – | 6.04 (5.14 – 7.57) | – | 7.98 (7.97 – 8) | – |
| **MAD of CPP (mmHg)** | 6.25 (5.06 – 7.34) |  | 8.12 (6.8 – 10.51) |  | 7.99 (5.83 – 10.66) |  | 8.12 (7.41 – 11.23) |  | 5.35 (5.06 – 5.58) |  | 7.41 (5.74 – 9.6) |  | 5.22 (5.22 – 5.22) |  | 6.58 (5.64 – 10.66) |  | 7.82 (7.67 – 7.97) |  |
| **MAD of rSO2_L (%)** | 6.52 (3.76 – 8.8) | 0.6857 | 4.34 (3.91 – 5.55) | 1 | 3.08 (2.98 – 3.29) | 0.3429 | 3.49 (2.97 – 3.99) | 0.535 | 5.6 (4.12 – 7.41) | 0.6857 | 3.56 (3.04 – 4.41) | 0.315 | 9.36 (9.36 – 9.36) | 1 | 3.99 (3.13 – 5.02) | 0.5737 | 2.61 (2.17 – 3.05) | 0.6667 |
| **MAD of rSO2_R (%)** | 7.87 (5.81 – 10.17) |  | 4.27 (3 – 8.08) |  | 4.14 (3.61 – 4.32) |  | 4.17 (2.91 – 4.53) |  | 7.87 (5.92 – 9.89) |  | 4.22 (3.79 – 8.12) |  | 6.52 (6.52 – 6.52) |  | 4.22 (4 – 5.9) |  | 7.38 (4.56 – 10.2) |  |
| **MAD of COx_L (au)** | 0.24 (0.23 – 0.26) | 0.2 | 0.25 (0.25 – 0.26) | 0.7 | 0.23 (0.22 – 0.24) | **0.0286** | 0.24 (0.23 – 0.25) | **0.0262** | 0.25 (0.24 – 0.26) | 0.6857 | 0.24 (0.23 – 0.25) | 0.0524 | 0.29 (0.29 – 0.29) | 1 | 0.24 (0.22 – 0.25) | 0.1049 | 0.24 (0.23 – 0.24) | 0.6667 |
| **MAD of COx_R (au)** | 0.29 (0.27 – 0.3) |  | 0.24 (0.24 – 0.27) |  | 0.26 (0.25 – 0.28) |  | 0.28 (0.25 – 0.29) |  | 0.26 (0.24 – 0.29) |  | 0.26 (0.24 – 0.29) |  | 0.3 (0.3 – 0.3) |  | 0.26 (0.25 – 0.29) |  | 0.26 (0.25 – 0.27) |  |
| **MAD of COx-a_L (au)** | 0.24 (0.22 – 0.26) | 0.4857 | 0.26 (0.25 – 0.27) | 0.7 | 0.23 (0.22 – 0.24) | 0.4857 | 0.24 (0.23 – 0.25) | 0.7104 | 0.25 (0.23 – 0.27) | 0.8857 | 0.24 (0.22 – 0.25) | 0.7959 | 0.27 (0.27 – 0.27) | 1 | 0.24 (0.23 – 0.25) | 0.5737 | 0.23 (0.23 – 0.24) | 1 |
| **MAD of COx-a_R (au)** | 0.25 (0.22 – 0.29) |  | 0.23 (0.22 – 0.27) |  | 0.25 (0.23 – 0.26) |  | 0.23 (0.23 – 0.27) |  | 0.24 (0.22 – 0.27) |  | 0.23 (0.22 – 0.27) |  | 0.29 (0.29 – 0.29) |  | 0.25 (0.22 – 0.27) |  | 0.23 (0.23 – 0.23) |  |
| **% time rSO2_L > 60%** | 99.8 (98.86 – 100; 0.29) | 0.8778 | 99.22 (95.83 – 99.36; 0.43) | 1 | 100 (94.74 – 100; 0) | 0.3562 | 100 (99.41 – 100; 0) | 0.3519 | 98.07 (92.21 – 99.63; 2.5) | 0.3836 | 99.56 (97.28 – 100; 0.65) | 0.2351 | 100 (100 – 100; 0) | – | 99.76 (95.58 – 100; 0.36) | 0.2575 | 99.41 (99.32 – 99.51; 0.29) | 1 |
| **% time rSO2_R > 60%** | 99.54 (97.11 – 100; 0.69) |  | 97.55 (76.81 – 98.68; 3.33) |  | 97.11 (88.46 – 98.63; 2.93) |  | 99.07 (97.86 – 99.9; 1.38) |  | 78.45 (63.29 – 93.41; 25.43) |  | 97.86 (92.42 – 99.62; 3.02) |  | 100 (100 – 100; 0) |  | 96.8 (84.84 – 98.63; 4.74) |  | 99.44 (99.25 – 99.62; 0.54) |  |
| **% time rSO2_L > 70%** | 87.61 (60.8 – 100; 18.38) | 1 | 47.96 (44.29 – 55.77; 10.88) | 0.7 | 99.7 (74.59 – 100; 0.45) | 0.4596 | 99.4 (44.29 – 100; 0.89) | 1 | 69.39 (47.72 – 81.41; 27.01) | 0.5614 | 69.39 (42.46 – 99.85; 44.94) | 0.9696 | 100 (100 – 100; 0) | – | 87.31 (59.67 – 100; 18.82) | 0.4884 | 29.1 (23.34 – 34.86; 17.08) | 0.3333 |
| **% time rSO2_R > 70%** | 84.85 (64.35 – 100; 22.46) |  | 84.43 (56.12 – 92.02; 22.49) |  | 58.15 (22.83 – 89.4; 51.57) |  | 85.87 (77.06 – 99.8; 20.95) |  | 38.06 (20.85 – 61.23; 35.81) |  | 77.06 (34.9 – 96.17; 34) |  | 100 (100 – 100; 0) |  | 66.37 (29.78 – 89.4; 49.86) |  | 84.65 (77.17 – 92.13; 22.17) |  |
| **% time rSO2_L > 80%** | 40.6 (20.36 – 55.97; 25.63) | 0.4857 | 4.15 (2.08 – 5.13; 2.91) | 0.5066 | 38.07 (15.09 – 62.95; 41.53) | 0.5614 | 20.12 (2.08 – 58.88; 29.83) | 0.7012 | 16.63 (4.58 – 33.88; 20.13) | 0.8824 | 13.12 (1.04 – 48.81; 19.45) | 0.909 | 54.05 (54.05 – 54.05; 0) | 1 | 23.64 (5.62 – 57.45; 31.96) | 0.5624 | 0 (0 – 0; 0) | 0.2207 |
| **% time rSO2_R > 80%** | 57.81 (39.03 – 75.3; 36.39) |  | 17.27 (8.64 – 18.24; 2.87) |  | 6.72 (0.59 – 27.78; 9.37) |  | 19.21 (14.96 – 60.29; 27.31) |  | 7.01 (0 – 27.58; 10.39) |  | 15.65 (3.75 – 40.33; 22.61) |  | 68.24 (68.24 – 68.24; 0) |  | 13.33 (0.59 – 31.26; 19.18) |  | 33.29 (26.25 – 40.33; 20.88) |  |
| **% time rSO2_L > 90%** | 6.42 (0 – 16.24; 9.52) | 0.6573 | 0 (0 – 0; 0) | 0.505 | 2.13 (0 – 13.96; 3.15) | 0.1859 | 0 (0 – 15.35; 0) | 0.8875 | 0 (0 – 3.21; 0) | 1 | 0 (0 – 3.19; 0) | 0.9257 | 12.84 (12.84 – 12.84; 0) | 1 | 0 (0 – 9.8; 0) | 0.3342 | 0 (0 – 0; 0) | 0.2207 |
| **% time rSO2_R > 90%** | 11.92 (2.53 – 25.62; 15.17) |  | 0 (0 – 0.1; 0) |  | 0 (0 – 0; 0) |  | 0 (0 – 10.33; 0) |  | 0 (0 – 0.84; 0) |  | 0 (0 – 0.15; 0) |  | 3.38 (3.38 – 3.38; 0) |  | 0 (0 – 0; 0) |  | 10.33 (5.27 – 15.4; 15.03) |  |
| **% time COx_L > 0** | 59.96 (56.33 – 63.2; 6.63) | 0.1143 | 38.22 (34.58 – 50.57; 10.79) | 1 | 59.58 (54.7 – 65.85; 12.22) | 0.8857 | 58.06 (41.41 – 61.53; 13.11) | 0.535 | 60.45 (56.33 – 67.22; 8.54) | 0.3429 | 59.58 (47.94 – 62.69; 7.92) | 0.2475 | 51.41 (51.41 – 51.41; 0) | 1 | 59.58 (54.63 – 63.92; 7.92) | 0.2786 | 46.45 (38.7 – 54.21; 22.99) | 1 |
| **% time COx_R > 0** | 46.16 (38.75 – 54.06; 11.82) |  | 36.1 (35.43 – 38.83; 1.99) |  | 61.11 (49.16 – 69.51; 12.51) |  | 41.57 (37.74 – 54.98; 10.09) |  | 46.16 (38.51 – 57.16; 12.53) |  | 47.15 (37.4 – 56.17; 14.95) |  | 39.31 (39.31 – 39.31; 0) |  | 52.87 (37.85 – 60.29; 23.02) |  | 39.31 (38.19 – 40.44; 3.34) |  |
| **% time COx_L > 0.2** | 27.66 (23.91 – 32.26; 5.61) | 0.1143 | 14.3 (12.89 – 23.66; 4.19) | 0.7 | 24.81 (20.37 – 33.48; 8.83) | 0.8857 | 21.88 (15.06 – 29.56; 11.25) | 0.9015 | 28.48 (23.91 – 37.44; 6.84) | 0.2 | 25.77 (17.34 – 32.61; 12.15) | 0.6842 | 23.94 (23.94 – 23.94; 0) | 1 | 25.77 (20.37 – 33.5; 12.15) | 0.7984 | 21.42 (16.45 – 26.4; 14.75) | 1 |
| **% time COx_R > 0.2** | 21.11 (18.27 – 24.54; 5.76) |  | 15.51 (15.35 – 16.19; 0.48) |  | 31.9 (21.58 – 40.92; 13.95) |  | 16.87 (15.99 – 26.35; 2.56) |  | 21.11 (18.28 – 27.71; 5.73) |  | 19.89 (15.75 – 27.88; 6.73) |  | 19.31 (19.31 – 19.31; 0) |  | 23.1 (16.23 – 32.18; 10.54) |  | 16 (15.57 – 16.44; 1.28) |  |
| **% time COx_L > 0.3** | 16.96 (14.93 – 19.78; 3.85) | 0.2 | 8.72 (7.74 – 14.64; 2.91) | 0.7 | 13.5 (10.99 – 20.46; 4.49) | 0.8857 | 11.51 (9.07 – 16.96; 5.89) | 0.9015 | 18.03 (14.93 – 24.26; 5.42) | 0.2 | 14.36 (9.94 – 20.03; 7.84) | 0.7394 | 15.49 (15.49 – 15.49; 0) | 1 | 14.36 (10.99 – 21.38; 7.84) | 0.9591 | 12.6 (9.68 – 15.51; 8.65) | 1 |
| **% time COx_R > 0.3** | 12.96 (11.67 – 14.57; 3.62) |  | 9.17 (9.1 – 9.6; 0.2) |  | 19.76 (14.05 – 25.51; 9.98) |  | 10.98 (9.54 – 17.01; 4.09) |  | 12.96 (11.9 – 17; 2.92) |  | 11.9 (9.39 – 17.98; 4.47) |  | 13.1 (13.1 – 13.1; 0) |  | 13.94 (10.74 – 20.33; 6.43) |  | 8.63 (8.42 – 8.83; 0.61) |  |
| **% time COx-a_L > 0** | 61.72 (57.56 – 67.05; 6.46) | 0.1143 | 40.74 (40.04 – 49.81; 2.07) | 1 | 61.17 (53.76 – 66.16; 11.53) | 1 | 58.97 (40.04 – 64.53; 17.97) | 0.9015 | 58.31 (57.56 – 62.79; 1.41) | 0.2 | 58.93 (44.8 – 65.12; 14.04) | 0.4813 | 57.75 (57.75 – 57.75; 0) | 1 | 58.93 (52.92 – 63.95; 8.31) | 0.3823 | 55.22 (47.28 – 63.16; 23.54) | 1 |
| **% time COx-a_R > 0** | 54.11 (50.73 – 57.38; 6.58) |  | 45.09 (40.38 – 46.47; 4.09) |  | 57.8 (50.56 – 67.17; 13.43) |  | 52.38 (45.09 – 58.55; 10.81) |  | 49.93 (47.61 – 54.81; 3.79) |  | 52.19 (45.79 – 59.71; 10.53) |  | 46.9 (46.9 – 46.9; 0) |  | 52.19 (47.16 – 57.96; 8.48) |  | 52.98 (49.04 – 56.93; 11.7) |  |
| **% time COx-a_L > 0.2** | 30.71 (25.45 – 35.37; 7.96) | 0.3429 | 16.1 (14.3 – 24.09; 5.33) | 0.7 | 24.38 (21.12 – 29.76; 7.81) | 0.6857 | 23.12 (15.6 – 30.15; 11.88) | 0.535 | 29.42 (25.45 – 34.59; 7.84) | 0.3429 | 24.38 (17.45 – 34.02; 13.02) | 1 | 26.76 (26.76 – 26.76; 0) | 1 | 24.38 (20.16 – 32.73; 11.85) | 1 | 25 (18.75 – 31.25; 18.53) | 1 |
| **% time COx-a_R > 0.2** | 25.09 (22.89 – 27.19; 3.81) |  | 16.62 (16.57 – 17.23; 0.15) |  | 29.7 (22.56 – 38.6; 12.43) |  | 23.81 (18.33 – 27.66; 7.39) |  | 22.34 (20.07 – 26.48; 5.06) |  | 22.52 (18.08 – 28.12; 7.84) |  | 23.45 (23.45 – 23.45; 0) |  | 22.52 (18.27 – 30.34; 8.82) |  | 22.28 (20.06 – 24.51; 6.59) |  |
| **% time COx-a_L > 0.3** | 16.85 (12.72 – 20.73; 6.38) | 0.8857 | 9.8 (8.9 – 14.09; 2.68) | 1 | 10.76 (8.69 – 16.41; 3.1) | 0.2 | 9.8 (8.67 – 16.57; 2.68) | 0.2593 | 15.88 (12.72 – 20.58; 5.65) | 0.6857 | 11.79 (8.98 – 19.83; 5.15) | 0.5787 | 13.38 (13.38 – 13.38; 0) | 1 | 11.79 (9.53 – 19.28; 4.62) | 0.5054 | 14.15 (11.07 – 17.23; 9.13) | 1 |
| **% time COx-a_R > 0.3** | 16.07 (14.67 – 16.68; 1.48) |  | 9.17 (8.99 – 9.51; 0.52) |  | 19.77 (14.56 – 26.04; 8.33) |  | 14.97 (11.59 – 17.07; 4.28) |  | 13.48 (10.61 – 18.04; 4.96) |  | 14.15 (10.16 – 17.46; 5.93) |  | 15.86 (15.86 – 15.86; 0) |  | 14.15 (10.78 – 19.53; 5.93) |  | 12.55 (10.68 – 14.42; 5.53) |  |
| *The p-values in the table are derived using Mann-Whitney U test between the bilateral signals.*  *ABP, arterial blood pressure; au, arbitrary units; CPP, cerebral perfusion pressure; COx, cerebral oximetry index with CPP; COx-a, cerebral oximetry index with ABP; CT, computed tomography; DAI, diffuse axonal injury; EDH, epidural hematoma; MAD, median absolute deviation; IQR, interquartile range; mmHg, millimeters of mercury; rSO2, regional cerebral oxygen saturation; tSAH, traumatic subarachnoid hemorrhage; SDH, subdural hematoma; aSDH, acute subdural hematoma; TBI-GR, traumatic brain injury patient group without right frontal lobe pathology.* | | | | | | | | | | | | | | | | | | |

| **Physiologic Variable** | **Sub-groups** | | | | | | | | | | | | | | | |
| --- | --- | --- | --- | --- | --- | --- | --- | --- | --- | --- | --- | --- | --- | --- | --- | --- |
|  | **Marshall CT II [n = 1]** | | **Rotterdam CT 6 [n = 5]** | | **Rotterdam CT 5 [n = 1]** | | **Rotterdam CT 4 [n = 4]** | | **Rotterdam CT 2 [n = 1]** | | **Propofol [n = 3]** | | **Fentanyl + Propofol [n = 6]** | | **Fentanyl + Midazolam + Propofol [n = 2]** | |
|  | **Median (IQR) or  Median (IQR; MAD)** | **p-value** | **Median (IQR) or  Median (IQR; MAD)** | **p-value** | **Median (IQR) or  Median (IQR; MAD)** | **p-value** | **Median (IQR) or  Median (IQR; MAD)** | **p-value** | **Median (IQR) or  Median (IQR; MAD)** | **p-value** | **Median (IQR) or  Median (IQR; MAD)** | **p-value** | **Median (IQR) or  Median (IQR; MAD)** | **p-value** | **Median (IQR) or  Median (IQR; MAD)** | **p-value** |
| **10-Second Data Resolution** | | | | | | | | | | | | | | | | |
| **ABP (mmHg)** | 84.01 (79.65 – 92.97) | – | 77.76 (73.8 – 82.03) | – | 75.98 (71.88 – 80.82) | – | 81.09 (77.07 – 85.6) | – | 84.01 (79.65 – 92.97) | – | 73.25 (70.47 – 78.49) | – | 78.64 (75.27 – 82.39) | – | 87.69 (80.58 – 94.92) | – |
| **CPP (mmHg)** | 72.91 (69.78 – 83.17) |  | 68.08 (64.99 – 72.38) |  | 66.62 (62.89 – 71.21) |  | 74.68 (69.83 – 81.1) |  | 72.91 (69.78 – 83.17) |  | 68.07 (64.82 – 72.38) |  | 70.5 (67.39 – 76.84) |  | 75.25 (68.37 – 82.95) |  |
| **rSO2_L (%)** | 82.4 (74.99 – 88.33) | 1 | 74 (69.99 – 80.99) | 0.7533 | 75.47 (74 – 79.5) | 1 | 68.84 (67.13 – 70.63) | 0.8857 | 82.4 (74.99 – 88.33) | 1 | 83 (78 – 90.1) | 0.7 | 73.73 (72 – 80.25) | 0.9372 | 69.25 (66.63 – 70.89) | 0.3333 |
| **rSO2_R (%)** | 83 (78.99 – 87) |  | 76.65 (72.99 – 78.99) |  | 75.97 (72.99 – 78.38) |  | 72.34 (66.56 – 74.99) |  | 83 (78.99 – 87) |  | 81.99 (79.99 – 83) |  | 72.98 (68.79 – 77.69) |  | 77.33 (71.06 – 83) |  |
| **COx_L (au)** | 0 (-0.21 – 0.2) | 1 | 0 (-0.11 – 0.21) | 0.2652 | 0.05 (-0.11 – 0.22) | 1 | 0.03 (-0.17 – 0.21) | 0.4857 | 0 (-0.21 – 0.2) | 1 | 0 (-0.03 – 0.23) | 0.1642 | 0 (-0.16 – 0.2) | 0.8089 | 0.01 (-0.21 – 0.2) | 0.6667 |
| **COx_R (au)** | -0.04 (-0.32 – 0.14) |  | 0 (-0.15 – 0.15) |  | 0.11 (-0.06 – 0.33) |  | -0.02 (-0.27 – 0.14) |  | -0.04 (-0.32 – 0.14) |  | 0 (-0.15 – 0.15) |  | -0.01 (-0.18 – 0.16) |  | -0.05 (-0.31 – 0.09) |  |
| **COx-a_L (au)** | 0 (-0.17 – 0.24) | – | 0 (-0.11 – 0.19) | 0.6072 | 0.05 (-0.1 – 0.22) | 1 | 0.04 (-0.16 – 0.23) | 1 | 0 (-0.17 – 0.24) | – | 0 (-0.04 – 0.22) | 0.505 | 0 (-0.14 – 0.2) | 0.8068 | 0.04 (-0.19 – 0.23) | 0.6667 |
| **COx-a_R (au)** | 0 (-0.24 – 0.22) |  | 0 (-0.15 – 0.16) |  | 0.16 (0 – 0.37) |  | 0 (-0.15 – 0.21) |  | 0 (-0.24 – 0.22) |  | 0 (-0.15 – 0.16) |  | 0 (-0.16 – 0.21) |  | -0.03 (-0.21 – 0.17) |  |
| **MAD of ABP (mmHg)** | 7.83 (7.83 – 7.83) | – | 7.44 (6.09 – 8.01) | – | 6.54 (6.54 – 6.54) | – | 6.93 (5.38 – 8.18) | – | 7.83 (7.83 – 7.83) | – | 7.44 (6.18 – 7.72) | – | 6.32 (5.8 – 7.51) | – | 10.59 (9.37 – 11.81) | – |
| **MAD of CPP (mmHg)** | 6.3 (6.3 – 6.3) |  | 7.19 (5.41 – 12.79) |  | 6 (6 – 6) |  | 7.89 (7.44 – 8.67) |  | 6.3 (6.3 – 6.3) |  | 7.19 (6.3 – 9.99) |  | 6.26 (6.06 – 7.53) |  | 10.71 (9.28 – 12.15) |  |
| **MAD of rSO2_L (%)** | 9.5 (9.5 – 9.5) | 1 | 4.45 (4.43 – 5.95) | 1 | 3.68 (3.68 – 3.68) | 1 | 2.98 (2.69 – 3.05) | 0.1913 | 9.5 (9.5 – 9.5) | 1 | 5.95 (4.46 – 7.41) | 1 | 3.45 (3.05 – 4.24) | 0.3776 | 3.15 (2.5 – 3.8) | 0.6667 |
| **MAD of rSO2_R (%)** | 5.95 (5.95 – 5.95) |  | 4.2 (3.72 – 8.9) |  | 4.42 (4.42 – 4.42) |  | 4.45 (3.82 – 6.65) |  | 5.95 (5.95 – 5.95) |  | 3.72 (2.97 – 7.75) |  | 4.45 (4.43 – 5.57) |  | 8.73 (6.46 – 10.99) |  |
| **MAD of COx_L (au)** | 0.31 (0.31 – 0.31) | 1 | 0.24 (0.23 – 0.26) | 0.5476 | 0.24 (0.24 – 0.24) | 1 | 0.28 (0.28 – 0.29) | 0.2 | 0.31 (0.31 – 0.31) | 1 | 0.23 (0.21 – 0.25) | 0.7 | 0.28 (0.25 – 0.29) | 0.2403 | 0.31 (0.3 – 0.32) | 1 |
| **MAD of COx_R (au)** | 0.36 (0.36 – 0.36) |  | 0.22 (0.22 – 0.23) |  | 0.29 (0.29 – 0.29) |  | 0.29 (0.29 – 0.31) |  | 0.36 (0.36 – 0.36) |  | 0.22 (0.22 – 0.23) |  | 0.29 (0.29 – 0.33) |  | 0.3 (0.29 – 0.31) |  |
| **MAD of COx-a_L (au)** | 0.3 (0.3 – 0.3) | 1 | 0.23 (0.23 – 0.27) | 0.8413 | 0.24 (0.24 – 0.24) | 1 | 0.28 (0.27 – 0.29) | 0.6857 | 0.3 (0.3 – 0.3) | 1 | 0.23 (0.2 – 0.25) | 1 | 0.27 (0.25 – 0.28) | 0.8182 | 0.32 (0.31 – 0.32) | 1 |
| **MAD of COx-a_R (au)** | 0.34 (0.34 – 0.34) |  | 0.22 (0.21 – 0.23) |  | 0.26 (0.26 – 0.26) |  | 0.27 (0.26 – 0.28) |  | 0.34 (0.34 – 0.34) |  | 0.22 (0.21 – 0.23) |  | 0.27 (0.27 – 0.29) |  | 0.28 (0.26 – 0.3) |  |
| **% time rSO2_L > 60%** | 100 (100 – 100; 0) | – | 99.84 (96.57 – 100; 0.24) | 0.402 | 100 (100 – 100; 0) | 1 | 99.39 (93.14 – 99.93; 0.83) | 0.3429 | 100 (100 – 100; 0) | – | 100 (99.92 – 100; 0) | 0.3758 | 99.44 (97.15 – 100; 0.83) | 0.3281 | 96.16 (94.29 – 98.04; 5.55) | 1 |
| **% time rSO2_R > 60%** | 100 (100 – 100; 0) |  | 97.58 (90.27 – 99.95; 3.56) |  | 98.21 (98.21 – 98.21; 0) |  | 97.23 (87.05 – 99.08; 3.13) |  | 100 (100 – 100; 0) |  | 99.95 (78.14 – 99.97; 0.04) |  | 96.92 (91.61 – 99.45; 4.46) |  | 98.2 (97.89 – 98.51; 0.92) |  |
| **% time rSO2_L > 70%** | 100 (100 – 100; 0) | – | 71.92 (63.59 – 99.95; 41.01) | 0.8413 | 98.99 (98.99 – 98.99; 0) | 1 | 27.1 (12.59 – 53.16; 27.54) | 1 | 100 (100 – 100; 0) | – | 99.95 (81.77 – 99.98; 0.07) | 0.7 | 85.45 (46.14 – 99.75; 21.57) | 0.6868 | 30.45 (23.56 – 37.35; 20.46) | 0.3333 |
| **% time rSO2_R > 70%** | 100 (100 – 100; 0) |  | 84.39 (46.83 – 99.94; 23.12) |  | 84.92 (84.92 – 84.92; 0) |  | 49.58 (22.55 – 76.82; 51.13) |  | 100 (100 – 100; 0) |  | 99.94 (63.81 – 99.96; 0.06) |  | 65.87 (34.21 – 96.11; 50.48) |  | 76.77 (72.96 – 80.58; 11.3) |  |
| **% time rSO2_L > 80%** | 53.47 (53.47 – 53.47; 0) | 1 | 25.95 (5.21 – 60.42; 33.72) | 1 | 17.61 (17.61 – 17.61; 0) | 1 | 0.01 (0 – 11.8; 0.01) | 0.6573 | 53.47 (53.47 – 53.47; 0) | 1 | 60.42 (32.81 – 71.54; 32.99) | 1 | 21.78 (4.41 – 41.86; 32.29) | 0.7483 | 1.6 (0.8 – 2.4; 2.38) | 0.3333 |
| **% time rSO2_R > 80%** | 66.34 (66.34 – 66.34; 0) |  | 14.96 (13.3 – 69.1; 22.18) |  | 10.73 (10.73 – 10.73; 0) |  | 9.54 (0.57 – 25.5; 13.58) |  | 66.34 (66.34 – 66.34; 0) |  | 69.1 (34.55 – 82.56; 39.9) |  | 12.02 (3.25 – 17.07; 13.02) |  | 31 (22.98 – 39.02; 23.78) |  |
| **% time rSO2_L > 90%** | 11.17 (11.17 – 11.17; 0) | 1 | 0 (0 – 25.16; 0) | 1 | 0 (0 – 0; 0) | 1 | 0 (0 – 0.9; 0) | 0.6198 | 11.17 (11.17 – 11.17; 0) | 1 | 25.16 (12.58 – 31.43; 18.6) | 0.8248 | 0 (0 – 2.69; 0) | 1 | 0 (0 – 0; 0) | 0.2207 |
| **% time rSO2_R > 90%** | 2.71 (2.71 – 2.71; 0) |  | 0.01 (0 – 0.1; 0.01) |  | 0.08 (0.08 – 0.08; 0) |  | 0.11 (0 – 5.2; 0.17) |  | 2.71 (2.71 – 2.71; 0) |  | 0.1 (0.05 – 18.77; 0.14) |  | 0.04 (0 – 0.19; 0.06) |  | 10.07 (5.04 – 15.1; 14.92) |  |
| **% time COx_L > 0** | 45.14 (45.14 – 45.14; 0) | 1 | 48.96 (44.9 – 50.08; 4.95) | 0.0556 | 58.58 (58.58 – 58.58; 0) | 1 | 51.45 (39.39 – 61.97; 17.85) | 0.4857 | 45.14 (45.14 – 45.14; 0) | 1 | 50.08 (47.49 – 51.19; 3.29) | 0.1 | 47.05 (43.14 – 56.18; 11.95) | 0.5887 | 48.74 (42.89 – 54.59; 17.34) | 0.3333 |
| **% time COx_R > 0** | 37.15 (37.15 – 37.15; 0) |  | 41 (33.15 – 42.12; 5.17) |  | 62.21 (62.21 – 62.21; 0) |  | 40.21 (37.64 – 44.5; 6.12) |  | 37.15 (37.15 – 37.15; 0) |  | 41 (34.24 – 41.56; 1.66) |  | 42.86 (39.7 – 51.87; 6.94) |  | 33.07 (33.02 – 33.11; 0.13) |  |
| **% time COx_L > 0.2** | 25.24 (25.24 – 25.24; 0) | 1 | 25.54 (25.49 – 27.12; 2.35) | 0.0556 | 27.61 (27.61 – 27.61; 0) | 1 | 27.03 (18.43 – 37.04; 14.78) | 1 | 25.24 (25.24 – 25.24; 0) | 1 | 27.12 (26.31 – 28.79; 2.41) | 0.1 | 25.39 (21.15 – 27.1; 5.8) | 1 | 26.63 (22.8 – 30.45; 11.34) | 0.3333 |
| **% time COx_R > 0.2** | 21.2 (21.2 – 21.2; 0) |  | 21.78 (17.86 – 22.19; 4.97) |  | 38.57 (38.57 – 38.57; 0) |  | 20.14 (19.09 – 24.19; 3.02) |  | 21.2 (21.2 – 21.2; 0) |  | 21.78 (17.57 – 23.45; 4.97) |  | 21.7 (20.45 – 32.67; 2.31) |  | 16.99 (16.56 – 17.42; 1.28) |  |
| **% time COx_L > 0.3** | 18.48 (18.48 – 18.48; 0) | 1 | 17.63 (15.98 – 18.18; 2.45) | 0.1508 | 16.45 (16.45 – 16.45; 0) | 1 | 18.09 (12.17 – 26.02; 10.1) | 1 | 18.48 (18.48 – 18.48; 0) | 1 | 18.18 (17.9 – 20.09; 0.82) | 0.2 | 16.21 (13.79 – 17.98; 4.02) | 1 | 18.05 (15.51 – 20.59; 7.53) | 0.3333 |
| **% time COx_R > 0.3** | 15.81 (15.81 – 15.81; 0) |  | 14.27 (12.61 – 15.24; 2.47) |  | 27.69 (27.69 – 27.69; 0) |  | 13.61 (12.84 – 17.2; 2.24) |  | 15.81 (15.81 – 15.81; 0) |  | 15.24 (12.39 – 16.69; 4.29) |  | 15.04 (13.79 – 24.72; 2.13) |  | 11.61 (11.11 – 12.11; 1.48) |  |
| **% time COx-a_L > 0** | 47.03 (47.03 – 47.03; 0) | 1 | 48.25 (43.75 – 49.41; 5.18) | 0.0952 | 58.96 (58.96 – 58.96; 0) | 1 | 50.8 (38.97 – 63.27; 18.29) | 1 | 47.03 (47.03 – 47.03; 0) | 1 | 49.41 (46.58 – 50.57; 3.46) | 0.1 | 47.64 (41.37 – 56.28; 13.62) | 1 | 52.82 (45.85 – 59.78; 20.65) | 0.6667 |
| **% time COx-a_R > 0** | 44.11 (44.11 – 44.11; 0) |  | 40.87 (36.53 – 41.38; 4.72) |  | 69.81 (69.81 – 69.81; 0) |  | 48.29 (45.6 – 50.36; 3.41) |  | 44.11 (44.11 – 44.11; 0) |  | 40.87 (38.7 – 41.12; 0.75) |  | 45.28 (44.07 – 49.89; 2.57) |  | 42.63 (38.89 – 46.38; 11.11) |  |
| **% time COx-a_L > 0.2** | 28.5 (28.5 – 28.5; 0) | 1 | 24.14 (23.53 – 26.47; 3.46) | 0.1508 | 27.82 (27.82 – 27.82; 0) | 1 | 28.95 (17.86 – 39.82; 16.39) | 1 | 28.5 (28.5 – 28.5; 0) | 1 | 26.47 (25.31 – 27.98; 3.46) | 0.2 | 25.68 (19.59 – 28.33; 7.59) | 0.6991 | 30.38 (25.38 – 35.38; 14.82) | 0.6667 |
| **% time COx-a_R > 0.2** | 26.38 (26.38 – 26.38; 0) |  | 20.96 (18.94 – 22.08; 3) |  | 45.16 (45.16 – 45.16; 0) |  | 25.6 (23.5 – 28.07; 4.62) |  | 26.38 (26.38 – 26.38; 0) |  | 22.08 (19.12 – 23.16; 3.19) |  | 25.45 (21.85 – 30.78; 7.03) |  | 22.81 (20.88 – 24.75; 5.74) |  |
| **% time COx-a_L > 0.3** | 19.76 (19.76 – 19.76; 0) | 1 | 17.03 (14.48 – 17.71; 3.78) | 0.1508 | 16.91 (16.91 – 16.91; 0) | 1 | 19.59 (11.29 – 28.13; 12.64) | 1 | 19.76 (19.76 – 19.76; 0) | 1 | 17.71 (17.37 – 19.38; 1.01) | 0.2 | 15.7 (12.26 – 19.05; 6.11) | 0.5887 | 20.72 (17.25 – 24.19; 10.29) | 0.6667 |
| **% time COx-a_R > 0.3** | 18.94 (18.94 – 18.94; 0) |  | 13.22 (13.04 – 15.16; 2.87) |  | 32.44 (32.44 – 32.44; 0) |  | 17.11 (15.52 – 19.62; 3.37) |  | 18.94 (18.94 – 18.94; 0) |  | 15.16 (12.67 – 16.4; 3.7) |  | 17.57 (14.15 – 23.06; 6.4) |  | 15.62 (14.42 – 16.81; 3.55) |  |
| **1-Minute Data Resolution** | | | | | | | | | | | | | | | | |
| **ABP (mmHg)** | 84.2 (79.76 – 92.75) | – | 77.82 (73.86 – 81.94) | – | 76.03 (71.94 – 80.74) | – | 81.11 (77.36 – 85.48) | – | 84.2 (79.76 – 92.75) | – | 73.18 (70.51 – 78.64) | – | 78.69 (75.5 – 82.26) | – | 87.73 (80.75 – 94.85) | – |
| **CPP (mmHg)** | 72.91 (69.9 – 83.07) |  | 68.16 (65.03 – 72.41) |  | 66.71 (62.95 – 71.06) |  | 74.59 (70.07 – 81.15) |  | 72.91 (69.9 – 83.07) |  | 68.16 (64.93 – 72.41) |  | 70.49 (67.47 – 76.88) |  | 75.36 (68.56 – 82.76) |  |
| **rSO2_L (%)** | 82.3 (75.24 – 88.32) | 1 | 74 (69.99 – 80.66) | 0.8413 | 75.39 (73.92 – 79.48) | 1 | 68.92 (67.11 – 70.58) | 0.8857 | 82.3 (75.24 – 88.32) | 1 | 82.92 (78 – 90.31) | 0.7 | 73.69 (71.95 – 80.07) | 0.9372 | 69.24 (66.65 – 70.96) | 0.3333 |
| **rSO2_R (%)** | 82.99 (78.72 – 87.08) |  | 76.59 (73.1 – 78.99) |  | 75.75 (72.86 – 78.4) |  | 72.29 (66.66 – 75.05) |  | 82.99 (78.72 – 87.08) |  | 81.99 (79.83 – 83) |  | 72.87 (68.71 – 77.65) |  | 77.29 (71.17 – 82.99) |  |
| **COx_L (au)** | 0 (-0.23 – 0.23) | 1 | 0.05 (-0.14 – 0.24) | 0.1508 | 0.05 (-0.1 – 0.21) | 1 | 0.02 (-0.17 – 0.21) | 0.6857 | 0 (-0.23 – 0.23) | 1 | 0.09 (-0.1 – 0.28) | 0.2 | 0.02 (-0.19 – 0.22) | 0.8182 | -0.01 (-0.22 – 0.2) | 0.3333 |
| **COx_R (au)** | -0.08 (-0.34 – 0.17) |  | 0.01 (-0.19 – 0.21) |  | 0.13 (-0.07 – 0.34) |  | -0.07 (-0.28 – 0.14) |  | -0.08 (-0.34 – 0.17) |  | 0.01 (-0.19 – 0.21) |  | -0.01 (-0.21 – 0.19) |  | -0.11 (-0.34 – 0.12) |  |
| **COx-a_L (au)** | 0.02 (-0.19 – 0.26) | 1 | 0.04 (-0.14 – 0.22) | 0.1508 | 0.05 (-0.1 – 0.21) | 1 | 0.03 (-0.16 – 0.23) | 1 | 0.02 (-0.19 – 0.26) | 1 | 0.07 (-0.12 – 0.27) | 0.1 | 0.03 (-0.17 – 0.21) | 0.9372 | 0.03 (-0.19 – 0.24) | 0.6667 |
| **COx-a_R (au)** | 0 (-0.25 – 0.23) |  | 0.01 (-0.2 – 0.19) |  | 0.18 (0 – 0.37) |  | 0.02 (-0.16 – 0.22) |  | 0 (-0.25 – 0.23) |  | 0.01 (-0.2 – 0.2) |  | 0.01 (-0.18 – 0.21) |  | -0.02 (-0.23 – 0.2) |  |
| **MAD of ABP (mmHg)** | 7.85 (7.85 – 7.85) | – | 7.42 (5.99 – 7.98) | – | 6.42 (6.42 – 6.42) | – | 6.7 (5.03 – 8.06) | – | 7.85 (7.85 – 7.85) | – | 7.42 (6.06 – 7.7) | – | 6.21 (5.54 – 7.49) | – | 10.47 (9.24 – 11.69) | – |
| **MAD of CPP (mmHg)** | 5.9 (5.9 – 5.9) |  | 7.16 (5.52 – 12.94) |  | 5.88 (5.88 – 5.88) |  | 7.78 (7.22 – 8.43) |  | 5.9 (5.9 – 5.9) |  | 7.16 (6.34 – 10.05) |  | 5.93 (5.88 – 7.43) |  | 10.43 (9.03 – 11.83) |  |
| **MAD of rSO2_L (%)** | 9.35 (9.35 – 9.35) | 1 | 4.43 (4.43 – 6.07) | 1 | 3.68 (3.68 – 3.68) | 1 | 3.02 (2.57 – 3.27) | 0.2 | 9.35 (9.35 – 9.35) | 1 | 6.07 (4.52 – 7.43) | 1 | 3.58 (3.27 – 4.24) | 0.4848 | 3.09 (2.42 – 3.76) | 0.6667 |
| **MAD of rSO2_R (%)** | 6.06 (6.06 – 6.06) |  | 4.23 (3.68 – 9.02) |  | 4.11 (4.11 – 4.11) |  | 4.45 (3.68 – 6.72) |  | 6.06 (6.06 – 6.06) |  | 3.68 (2.92 – 7.76) |  | 4.45 (4.15 – 5.7) |  | 8.65 (6.44 – 10.86) |  |
| **MAD of COx_L (au)** | 0.34 (0.34 – 0.34) | 1 | 0.29 (0.28 – 0.31) | 0.6905 | 0.23 (0.23 – 0.23) | 1 | 0.29 (0.28 – 0.3) | 0.0571 | 0.34 (0.34 – 0.34) | 1 | 0.29 (0.28 – 0.3) | 0.7 | 0.29 (0.28 – 0.31) | 0.2403 | 0.31 (0.3 – 0.32) | 0.6667 |
| **MAD of COx_R (au)** | 0.38 (0.38 – 0.38) |  | 0.29 (0.28 – 0.35) |  | 0.3 (0.3 – 0.3) |  | 0.33 (0.32 – 0.34) |  | 0.38 (0.38 – 0.38) |  | 0.29 (0.29 – 0.32) |  | 0.32 (0.3 – 0.35) |  | 0.34 (0.34 – 0.35) |  |
| **MAD of COx-a_L (au)** | 0.33 (0.33 – 0.33) | 1 | 0.29 (0.28 – 0.31) | 1 | 0.23 (0.23 – 0.23) | 1 | 0.29 (0.29 – 0.3) | 0.3429 | 0.33 (0.33 – 0.33) | 1 | 0.29 (0.28 – 0.3) | 1 | 0.29 (0.27 – 0.3) | 1 | 0.31 (0.3 – 0.33) | 1 |
| **MAD of COx-a_R (au)** | 0.36 (0.36 – 0.36) |  | 0.3 (0.27 – 0.33) |  | 0.27 (0.27 – 0.27) |  | 0.28 (0.28 – 0.29) |  | 0.36 (0.36 – 0.36) |  | 0.3 (0.29 – 0.32) |  | 0.28 (0.28 – 0.32) |  | 0.32 (0.3 – 0.34) |  |
| **% time rSO2_L > 60%** | 100 (100 – 100; 0) | – | 99.75 (96.61 – 100; 0.37) | 0.5258 | 100 (100 – 100; 0) | 1 | 99.35 (93.46 – 99.88; 0.85) | 0.4857 | 100 (100 – 100; 0) | – | 100 (99.87 – 100; 0) | 0.6428 | 99.43 (97.17 – 100; 0.85) | 0.3281 | 96.11 (94.24 – 97.97; 5.54) | 1 |
| **% time rSO2_R > 60%** | 100 (100 – 100; 0) |  | 97.63 (90.64 – 99.94; 3.52) |  | 98.2 (98.2 – 98.2; 0) |  | 97.35 (87.61 – 99.15; 3.01) |  | 100 (100 – 100; 0) |  | 99.94 (78.3 – 99.97; 0.09) |  | 96.99 (91.93 – 99.43; 4.34) |  | 98.28 (97.95 – 98.6; 0.96) |  |
| **% time rSO2_L > 70%** | 100 (100 – 100; 0) | – | 73.25 (63.87 – 100; 39.52) | 0.8325 | 99.22 (99.22 – 99.22; 0) | 1 | 28.77 (13.44 – 54.81; 29.18) | 1 | 100 (100 – 100; 0) | – | 100 (81.94 – 100; 0) | 0.6428 | 86.23 (48.12 – 99.8; 20.41) | 0.6868 | 32.2 (25 – 39.39; 21.34) | 0.3333 |
| **% time rSO2_R > 70%** | 100 (100 – 100; 0) |  | 84.53 (47.53 – 99.9; 22.94) |  | 85.54 (85.54 – 85.54; 0) |  | 49.74 (22.73 – 76.88; 51.17) |  | 100 (100 – 100; 0) |  | 99.9 (63.84 – 99.95; 0.16) |  | 66.54 (34.56 – 96.24; 49.46) |  | 76.88 (73.06 – 80.71; 11.33) |  |
| **% time rSO2_L > 80%** | 53.89 (53.89 – 53.89; 0) | 1 | 26.82 (5.77 – 61.08; 33.9) | 1 | 19.68 (19.68 – 19.68; 0) | 1 | 0 (0 – 13.02; 0) | 0.5385 | 53.89 (53.89 – 53.89; 0) | 1 | 61.08 (33.43 – 72.3; 33.25) | 1 | 23.25 (4.92 – 45.76; 34.47) | 0.6868 | 1.98 (0.99 – 2.97; 2.93) | 0.3333 |
| **% time rSO2_R > 80%** | 67.53 (67.53 – 67.53; 0) |  | 16.2 (13.75 – 70.67; 24.01) |  | 11.88 (11.88 – 11.88; 0) |  | 9.97 (0.6 – 26.18; 14.18) |  | 67.53 (67.53 – 67.53; 0) |  | 70.67 (35.34 – 83.39; 37.71) |  | 12.81 (3.57 – 17.79; 13.59) |  | 31.76 (23.98 – 39.55; 23.08) |  |
| **% time rSO2_L > 90%** | 12.69 (12.69 – 12.69; 0) | 1 | 0 (0 – 26.34; 0) | 0.9063 | 0 (0 – 0; 0) | 1 | 0 (0 – 0.93; 0) | 0.6198 | 12.69 (12.69 – 12.69; 0) | 1 | 26.34 (13.17 – 33.88; 22.35) | 0.8248 | 0 (0 – 2.79; 0) | 1 | 0 (0 – 0; 0) | 0.6171 |
| **% time rSO2_R > 90%** | 3.27 (3.27 – 3.27; 0) |  | 0 (0 – 0.1; 0) |  | 0.09 (0.09 – 0.09; 0) |  | 0.18 (0 – 5.37; 0.26) |  | 3.27 (3.27 – 3.27; 0) |  | 0.1 (0.05 – 19.6; 0.14) |  | 0.05 (0 – 0.29; 0.07) |  | 10.21 (5.11 – 15.32; 15.14) |  |
| **% time COx_L > 0** | 49.7 (49.7 – 49.7; 0) | 1 | 57.15 (56.68 – 61.4; 6.31) | 0.0556 | 59.31 (59.31 – 59.31; 0) | 1 | 52.48 (40.84 – 64.44; 21.37) | 0.8857 | 49.7 (49.7 – 49.7; 0) | 1 | 61.4 (59.04 – 62.92; 4.49) | 0.1 | 53.43 (45.13 – 58.77; 11.64) | 0.9372 | 50.43 (44.97 – 55.89; 16.2) | 0.3333 |
| **% time COx_R > 0** | 40.06 (40.06 – 40.06; 0) |  | 51.06 (36.5 – 53.39; 6.64) |  | 67.04 (67.04 – 67.04; 0) |  | 41.52 (39.02 – 49.03; 3.97) |  | 40.06 (40.06 – 40.06; 0) |  | 51.06 (43.1 – 53.3; 6.64) |  | 48.62 (41.01 – 61.77; 13.34) |  | 37.5 (37 – 38; 1.48) |  |
| **% time COx_L > 0.2** | 27.61 (27.61 – 27.61; 0) | 1 | 28.8 (28.48 – 34.45; 8.37) | 0.1508 | 26.53 (26.53 – 26.53; 0) | 1 | 27.03 (18.85 – 38.12; 13.85) | 0.8857 | 27.61 (27.61 – 27.61; 0) | 1 | 34.45 (31.46 – 36.4; 5.79) | 0.2 | 27.07 (21.63 – 28.5; 6.52) | 0.9372 | 26.79 (23.15 – 30.43; 10.79) | 0.3333 |
| **% time COx_R > 0.2** | 22.53 (22.53 – 22.53; 0) |  | 25.37 (19.21 – 25.61; 9.14) |  | 40.56 (40.56 – 40.56; 0) |  | 20.05 (19.29 – 26; 1.69) |  | 22.53 (22.53 – 22.53; 0) |  | 25.61 (21.1 – 29.06; 10.25) |  | 23.95 (20.95 – 36.77; 5.79) |  | 18.68 (18.41 – 18.94; 0.79) |  |
| **% time COx_L > 0.3** | 19.85 (19.85 – 19.85; 0) | 1 | 18.35 (17.67 – 22.67; 6.4) | 0.3095 | 15.81 (15.81 – 15.81; 0) | 1 | 17.54 (12.26 – 25.77; 9.05) | 1 | 19.85 (19.85 – 19.85; 0) | 1 | 22.67 (20.51 – 25.02; 6.4) | 0.4 | 16.74 (13.76 – 19.3; 5.02) | 0.8182 | 17.5 (15.24 – 19.75; 6.68) | 0.6667 |
| **% time COx_R > 0.3** | 16.05 (16.05 – 16.05; 0) |  | 16.12 (13.42 – 17.42; 4) |  | 28.82 (28.82 – 28.82; 0) |  | 13.37 (12.88 – 18.19; 1.21) |  | 16.05 (16.05 – 16.05; 0) |  | 17.42 (14.66 – 20.29; 8.19) |  | 16.09 (14.17 – 25.64; 4.03) |  | 12.67 (12.29 – 13.04; 1.12) |  |
| **% time COx-a_L > 0** | 52.39 (52.39 – 52.39; 0) | 1 | 56.49 (55.8 – 60.11; 5.36) | 0.0952 | 58.96 (58.96 – 58.96; 0) | 1 | 54.25 (41.63 – 67.24; 19.19) | 1 | 52.39 (52.39 – 52.39; 0) | 1 | 60.11 (58.3 – 61.84; 5.13) | 0.1 | 54.09 (44.56 – 58.17; 12.61) | 0.8182 | 53.85 (47.51 – 60.2; 18.8) | 0.6667 |
| **% time COx-a_R > 0** | 50.07 (50.07 – 50.07; 0) |  | 51.05 (46.93 – 52.72; 4.55) |  | 75.39 (75.39 – 75.39; 0) |  | 52.72 (46.43 – 59.35; 9.52) |  | 50.07 (50.07 – 50.07; 0) |  | 51.05 (48.99 – 52.58; 4.55) |  | 51.4 (47.44 – 58.75; 7.55) |  | 48.86 (43.85 – 53.87; 14.85) |  |
| **% time COx-a_L > 0.2** | 30.3 (30.3 – 30.3; 0) | 1 | 27.58 (25.61 – 32.27; 6.95) | 0.1508 | 26.67 (26.67 – 26.67; 0) | 1 | 29.38 (18.38 – 40.82; 16.7) | 1 | 30.3 (30.3 – 30.3; 0) | 1 | 32.27 (29.92 – 34.62; 6.95) | 0.2 | 26.14 (20.39 – 29.39; 8.64) | 0.6991 | 30.5 (25.69 – 35.3; 14.25) | 0.6667 |
| **% time COx-a_R > 0.2** | 27.98 (27.98 – 27.98; 0) |  | 23.62 (20.25 – 25.08; 5) |  | 47.74 (47.74 – 47.74; 0) |  | 27.16 (23.12 – 32.3; 7.24) |  | 27.98 (27.98 – 27.98; 0) |  | 25.08 (22.61 – 27.93; 7.31) |  | 25.97 (23.71 – 35.6; 5.73) |  | 25.3 (22.77 – 27.83; 7.5) |  |
| **% time COx-a_L > 0.3** | 20.9 (20.9 – 20.9; 0) | 1 | 18.34 (15.42 – 22.26; 5.81) | 0.3095 | 16.23 (16.23 – 16.23; 0) | 1 | 19.81 (11.96 – 28.19; 12.2) | 0.8857 | 20.9 (20.9 – 20.9; 0) | 1 | 22.26 (20.3 – 24.41; 5.81) | 0.4 | 15.82 (13.1 – 19.73; 6.29) | 0.5887 | 20.55 (17.19 – 23.92; 9.98) | 0.6667 |
| **% time COx-a_R > 0.3** | 19.44 (19.44 – 19.44; 0) |  | 15.01 (13.64 – 17.72; 4.02) |  | 34.03 (34.03 – 34.03; 0) |  | 17.97 (15.09 – 22.25; 5.07) |  | 19.44 (19.44 – 19.44; 0) |  | 17.72 (14.91 – 20.08; 6.99) |  | 17.53 (15.16 – 25.9; 4.88) |  | 16.97 (15.31 – 18.64; 4.95) |  |
| **5-Minute Data Resolution** | | | | | | | | | | | | | | | | |
| **ABP (mmHg)** | 83.99 (79.78 – 92.16) | – | 77.79 (74.13 – 81.89) | – | 76.05 (72.04 – 80.68) | – | 81.2 (77.6 – 85.55) | – | 83.99 (79.78 – 92.16) | – | 73.2 (70.59 – 78.79) | – | 78.67 (75.77 – 82.25) | – | 88.01 (80.83 – 94.87) | – |
| **CPP (mmHg)** | 72.71 (69.95 – 83.03) |  | 68.15 (65.17 – 72.62) |  | 66.78 (63.13 – 70.95) |  | 74.52 (70.37 – 81.43) |  | 72.71 (69.95 – 83.03) |  | 68.15 (65.11 – 72.62) |  | 70.42 (67.56 – 77.05) |  | 75.49 (68.81 – 82.71) |  |
| **rSO2_L (%)** | 82.1 (75.3 – 88.34) | 1 | 73.98 (70.04 – 80.59) | 0.8413 | 75.39 (73.95 – 79.42) | 1 | 68.94 (67.11 – 70.55) | 0.8857 | 82.1 (75.3 – 88.34) | 1 | 82.94 (78 – 90.42) | 0.7 | 73.69 (71.99 – 80.01) | 0.9372 | 69.24 (66.61 – 70.92) | 0.3333 |
| **rSO2_R (%)** | 82.67 (78.92 – 87.08) |  | 76.56 (73.09 – 79.06) |  | 75.75 (72.82 – 78.39) |  | 72.3 (66.74 – 75.04) |  | 82.67 (78.92 – 87.08) |  | 81.99 (79.68 – 83) |  | 72.87 (68.64 – 77.63) |  | 77.26 (71.18 – 83.03) |  |
| **COx_L (au)** | 0.04 (-0.18 – 0.19) | 1 | 0.05 (-0.1 – 0.21) | 0.0952 | 0.06 (-0.07 – 0.18) | 1 | 0.02 (-0.14 – 0.16) | 0.6857 | 0.04 (-0.18 – 0.19) | 1 | 0.1 (-0.09 – 0.27) | 0.2 | 0.05 (-0.15 – 0.19) | 0.6991 | 0 (-0.17 – 0.16) | 0.3333 |
| **COx_R (au)** | -0.11 (-0.29 – 0.14) |  | 0.02 (-0.17 – 0.18) |  | 0.14 (-0.04 – 0.3) |  | -0.07 (-0.24 – 0.12) |  | -0.11 (-0.29 – 0.14) |  | 0.02 (-0.17 – 0.18) |  | -0.02 (-0.17 – 0.17) |  | -0.11 (-0.3 – 0.09) |  |
| **COx-a_L (au)** | 0.02 (-0.16 – 0.21) | 1 | 0.04 (-0.11 – 0.2) | 0.2222 | 0.05 (-0.07 – 0.18) | 1 | 0.04 (-0.13 – 0.19) | 1 | 0.02 (-0.16 – 0.21) | 1 | 0.08 (-0.1 – 0.25) | 0.2 | 0.03 (-0.13 – 0.17) | 0.9372 | 0.04 (-0.15 – 0.19) | 0.6667 |
| **COx-a_R (au)** | -0.04 (-0.2 – 0.19) |  | 0.01 (-0.16 – 0.16) |  | 0.19 (0.03 – 0.33) |  | 0.02 (-0.13 – 0.18) |  | -0.04 (-0.2 – 0.19) |  | 0.01 (-0.16 – 0.19) |  | 0 (-0.15 – 0.17) |  | -0.02 (-0.19 – 0.16) |  |
| **MAD of ABP (mmHg)** | 7.04 (7.04 – 7.04) | – | 7.56 (5.74 – 7.59) | – | 6.33 (6.33 – 6.33) | – | 6.63 (4.91 – 7.97) | – | 7.04 (7.04 – 7.04) | – | 7.56 (6.09 – 7.58) | – | 6.04 (5.42 – 6.87) | – | 10.33 (9.14 – 11.52) | – |
| **MAD of CPP (mmHg)** | 5.22 (5.22 – 5.22) |  | 7.28 (5.48 – 12.36) |  | 5.69 (5.69 – 5.69) |  | 7.82 (7.11 – 8.61) |  | 5.22 (5.22 – 5.22) |  | 7.28 (6.38 – 9.82) |  | 5.78 (5.34 – 7.56) |  | 10.21 (8.87 – 11.55) |  |
| **MAD of rSO2_L (%)** | 9.36 (9.36 – 9.36) | 1 | 4.43 (4.34 – 6.76) | 1 | 3.64 (3.64 – 3.64) | 1 | 3.08 (2.68 – 3.25) | 0.2 | 9.36 (9.36 – 9.36) | 1 | 6.76 (4.86 – 7.69) | 1 | 3.56 (3.25 – 4.23) | 0.4848 | 3.04 (2.39 – 3.69) | 0.6667 |
| **MAD of rSO2_R (%)** | 6.52 (6.52 – 6.52) |  | 4.27 (3.68 – 9.22) |  | 4.17 (4.17 – 4.17) |  | 4.45 (3.51 – 6.85) |  | 6.52 (6.52 – 6.52) |  | 3.68 (2.91 – 7.79) |  | 4.48 (4.12 – 6.09) |  | 8.64 (6.46 – 10.83) |  |
| **MAD of COx_L (au)** | 0.29 (0.29 – 0.29) | 1 | 0.25 (0.24 – 0.25) | 0.8413 | 0.19 (0.19 – 0.19) | 1 | 0.24 (0.23 – 0.24) | 0.1143 | 0.29 (0.29 – 0.29) | 1 | 0.25 (0.25 – 0.25) | 1 | 0.23 (0.22 – 0.24) | 0.132 | 0.25 (0.24 – 0.26) | 0.3333 |
| **MAD of COx_R (au)** | 0.3 (0.3 – 0.3) |  | 0.25 (0.24 – 0.29) |  | 0.25 (0.25 – 0.25) |  | 0.28 (0.27 – 0.28) |  | 0.3 (0.3 – 0.3) |  | 0.25 (0.24 – 0.27) |  | 0.26 (0.24 – 0.29) |  | 0.29 (0.28 – 0.29) |  |
| **MAD of COx-a_L (au)** | 0.27 (0.27 – 0.27) | 1 | 0.25 (0.24 – 0.26) | 0.6905 | 0.19 (0.19 – 0.19) | 1 | 0.23 (0.23 – 0.24) | 1 | 0.27 (0.27 – 0.27) | 1 | 0.25 (0.24 – 0.26) | 1 | 0.23 (0.21 – 0.24) | 0.8182 | 0.25 (0.23 – 0.26) | 0.6667 |
| **MAD of COx-a_R (au)** | 0.29 (0.29 – 0.29) |  | 0.26 (0.22 – 0.28) |  | 0.22 (0.22 – 0.22) |  | 0.23 (0.23 – 0.24) |  | 0.29 (0.29 – 0.29) |  | 0.26 (0.24 – 0.27) |  | 0.23 (0.23 – 0.26) |  | 0.26 (0.24 – 0.28) |  |
| **% time rSO2_L > 60%** | 100 (100 – 100; 0) | – | 99.51 (96.63 – 100; 0.72) | 0.6664 | 100 (100 – 100; 0) | 1 | 99.41 (94.15 – 99.71; 0.58) | 0.4857 | 100 (100 – 100; 0) | – | 100 (99.76 – 100; 0) | 1 | 99.61 (97.28 – 100; 0.58) | 0.3281 | 96.02 (94.23 – 97.82; 5.32) | 1 |
| **% time rSO2_R > 60%** | 100 (100 – 100; 0) |  | 97.55 (91.22 – 100; 3.63) |  | 98.17 (98.17 – 98.17; 0) |  | 97.56 (88.46 – 99.25; 2.78) |  | 100 (100 – 100; 0) |  | 100 (78.04 – 100; 0) |  | 97.11 (92.42 – 99.4; 4.14) |  | 98.31 (97.93 – 98.69; 1.13) |  |
| **% time rSO2_L > 70%** | 100 (100 – 100; 0) | – | 75.21 (63.57 – 100; 36.75) | 1 | 99.4 (99.4 – 99.4; 0) | 1 | 29.1 (13.22 – 55.47; 30) | 1 | 100 (100 – 100; 0) | – | 100 (81.78 – 100; 0) | 1 | 87.31 (49.27 – 99.85; 18.82) | 0.6868 | 32.77 (25.17 – 40.37; 22.52) | 0.3333 |
| **% time rSO2_R > 70%** | 100 (100 – 100; 0) |  | 84.43 (48.31 – 100; 23.08) |  | 85.87 (85.87 – 85.87; 0) |  | 50.07 (22.83 – 77.17; 51.28) |  | 100 (100 – 100; 0) |  | 100 (63.9 – 100; 0) |  | 67.09 (34.9 – 96.17; 48.5) |  | 77.06 (73.38 – 80.75; 10.92) |  |
| **% time rSO2_L > 80%** | 54.05 (54.05 – 54.05; 0) | 1 | 27.15 (6.11 – 61.72; 34.1) | 1 | 20.12 (20.12 – 20.12; 0) | 1 | 0 (0 – 14.01; 0) | 0.5385 | 54.05 (54.05 – 54.05; 0) | 1 | 61.72 (33.92 – 72.73; 32.63) | 1 | 23.64 (5.03 – 47.33; 35.04) | 0.6868 | 2.08 (1.04 – 3.11; 3.08) | 0.3333 |
| **% time rSO2_R > 80%** | 68.24 (68.24 – 68.24; 0) |  | 17.27 (14.02 – 73.21; 25.61) |  | 12.64 (12.64 – 12.64; 0) |  | 10 (0.59 – 26.25; 14.24) |  | 68.24 (68.24 – 68.24; 0) |  | 73.21 (36.6 – 84.83; 34.47) |  | 13.33 (3.75 – 17.91; 13.65) |  | 32.32 (24.8 – 39.85; 22.31) |  |
| **% time rSO2_L > 90%** | 12.84 (12.84 – 12.84; 0) | 1 | 0 (0 – 26.45; 0) | 0.6072 | 0 (0 – 0; 0) | – | 0 (0 – 1.06; 0) | 0.6198 | 12.84 (12.84 – 12.84; 0) | 1 | 26.45 (13.23 – 34.76; 24.63) | 0.6428 | 0 (0 – 3.19; 0) | 0.775 | 0 (0 – 0; 0) | 0.6171 |
| **% time rSO2_R > 90%** | 3.38 (3.38 – 3.38; 0) |  | 0 (0 – 0; 0) |  | 0 (0 – 0; 0) |  | 0.1 (0 – 5.27; 0.15) |  | 3.38 (3.38 – 3.38; 0) |  | 0 (0 – 20.54; 0) |  | 0 (0 – 0.15; 0) |  | 10.24 (5.12 – 15.35; 15.17) |  |
| **% time COx_L > 0** | 51.41 (51.41 – 51.41; 0) | 1 | 58.06 (57.97 – 62.93; 7.21) | 0.0556 | 61.09 (61.09 – 61.09; 0) | 1 | 53.28 (41.19 – 66.5; 22.99) | 0.6857 | 51.41 (51.41 – 51.41; 0) | 1 | 62.93 (60.5 – 64.92; 5.9) | 0.1 | 54.69 (46.31 – 60.31; 12.22) | 0.8182 | 50.09 (44.15 – 56.03; 17.6) | 0.3333 |
| **% time COx_R > 0** | 39.31 (39.31 – 39.31; 0) |  | 52.74 (36.1 – 53.01; 6.66) |  | 69.48 (69.48 – 69.48; 0) |  | 40 (38.09 – 48.58; 3.34) |  | 39.31 (39.31 – 39.31; 0) |  | 52.74 (44.42 – 54.98; 6.66) |  | 47.29 (39.87 – 65.36; 12.48) |  | 35.91 (35.34 – 36.48; 1.7) |  |
| **% time COx_L > 0.2** | 23.94 (23.94 – 23.94; 0) | 1 | 27.74 (23.8 – 33.02; 7.83) | 0.3095 | 21.88 (21.88 – 21.88; 0) | 1 | 23.6 (14.74 – 36.2; 14.75) | 1 | 23.94 (23.94 – 23.94; 0) | 1 | 33.02 (30.38 – 33.97; 2.81) | 0.2 | 22.84 (17.34 – 23.91; 6.02) | 0.9372 | 22.84 (18.57 – 27.1; 12.66) | 1 |
| **% time COx_R > 0.2** | 19.31 (19.31 – 19.31; 0) |  | 22.91 (15.51 – 23.29; 9.63) |  | 40.52 (40.52 – 40.52; 0) |  | 16.67 (16.14 – 23.18; 1.28) |  | 19.31 (19.31 – 19.31; 0) |  | 23.29 (19.24 – 26.35; 9.07) |  | 21.11 (17.48 – 36.12; 6.59) |  | 15.33 (15.23 – 15.42; 0.27) |  |
| **% time COx_L > 0.3** | 15.49 (15.49 – 15.49; 0) | 1 | 15.48 (13.24 – 20.56; 7.53) | 0.4206 | 9.42 (9.42 – 9.42; 0) | 1 | 14.97 (10.32 – 22.67; 8.65) | 0.6857 | 15.49 (15.49 – 15.49; 0) | 1 | 20.56 (18.02 – 22.2; 4.87) | 0.2 | 12.38 (9.94 – 14.93; 4.5) | 0.9372 | 13.58 (11.15 – 16; 7.2) | 0.6667 |
| **% time COx_R > 0.3** | 13.1 (13.1 – 13.1; 0) |  | 12.82 (10.04 – 15.07; 4.12) |  | 24.44 (24.44 – 24.44; 0) |  | 10.01 (8.83 – 15.41; 2.05) |  | 13.1 (13.1 – 13.1; 0) |  | 15.07 (12.12 – 17.01; 5.76) |  | 12.96 (11.44 – 21.61; 4.38) |  | 9.13 (8.67 – 9.58; 1.35) |  |
| **% time COx-a_L > 0** | 57.75 (57.75 – 57.75; 0) | 1 | 58.88 (56.98 – 58.97; 2.81) | 0.0952 | 63.36 (63.36 – 63.36; 0) | 1 | 55.22 (39.04 – 71.95; 24.44) | 1 | 57.75 (57.75 – 57.75; 0) | 1 | 58.97 (58.93 – 62.34; 0.14) | 0.1 | 57.36 (43.75 – 61.96; 17.18) | 1 | 55.92 (48.33 – 63.5; 22.5) | 0.6667 |
| **% time COx-a_R > 0** | 46.9 (46.9 – 46.9; 0) |  | 52.01 (47.85 – 52.38; 6.16) |  | 79.03 (79.03 – 79.03; 0) |  | 52.99 (45.1 – 61.46; 11.7) |  | 46.9 (46.9 – 46.9; 0) |  | 52.38 (50.12 – 54.3; 5.68) |  | 49.45 (45.55 – 60.42; 6.46) |  | 48.28 (41.98 – 54.58; 18.68) |  |
| **% time COx-a_L > 0.2** | 26.76 (26.76 – 26.76; 0) | 1 | 25.64 (21.51 – 32.09; 9.56) | 0.4206 | 23.12 (23.12 – 23.12; 0) | 1 | 26.3 (14.46 – 38.65; 18.53) | 1 | 26.76 (26.76 – 26.76; 0) | 1 | 32.09 (28.86 – 33.37; 3.82) | 0.2 | 22.32 (16.71 – 25.85; 8.64) | 0.6991 | 26.8 (21.45 – 32.15; 15.87) | 1 |
| **% time COx-a_R > 0.2** | 23.45 (23.45 – 23.45; 0) |  | 21.22 (16.62 – 23.81; 6.83) |  | 47.64 (47.64 – 47.64; 0) |  | 22.78 (18.58 – 28.95; 6.59) |  | 23.45 (23.45 – 23.45; 0) |  | 23.81 (20.21 – 26.2; 7.09) |  | 22.34 (19.42 – 32.56; 5.94) |  | 21.62 (19.07 – 24.18; 7.57) |  |
| **% time COx-a_L > 0.3** | 13.38 (13.38 – 13.38; 0) | 1 | 12.82 (10.75 – 18.38; 4.48) | 0.5476 | 8.71 (8.71 – 8.71; 0) | 1 | 14.47 (8.47 – 22.03; 9.13) | 0.8857 | 13.38 (13.38 – 13.38; 0) | 1 | 18.38 (15.6 – 20.18; 5.34) | 0.4 | 9.73 (8.65 – 12.72; 2.1) | 0.1797 | 15.06 (12.43 – 17.68; 7.79) | 1 |
| **% time COx-a_R > 0.3** | 15.86 (15.86 – 15.86; 0) |  | 11.09 (9.85 – 14.97; 2.85) |  | 30.43 (30.43 – 30.43; 0) |  | 14.81 (12.2 – 18.36; 5.53) |  | 15.86 (15.86 – 15.86; 0) |  | 14.97 (12.07 – 16.41; 4.28) |  | 14.6 (11.65 – 22.4; 6.89) |  | 13.07 (11.46 – 14.68; 4.77) |  |
| *The p-values in the table are derived using Mann-Whitney U test between the bilateral signals.*  *ABP, arterial blood pressure; au, arbitrary units; CPP, cerebral perfusion pressure; COx, cerebral oximetry index with CPP; COx-a, cerebral oximetry index with ABP; CT, computed tomography; DAI, diffuse axonal injury; EDH, epidural hematoma; MAD, median absolute deviation; IQR, interquartile range; mmHg, millimeters of mercury; rSO2, regional cerebral oxygen saturation; tSAH, traumatic subarachnoid hemorrhage; SDH, subdural hematoma; aSDH, acute subdural hematoma; TBI-GR, traumatic brain injury patient group without right frontal lobe pathology.* | | | | | | | | | | | | | | | | |

File S8l: Sub-grouped Physiologic Results using 10-Second, 1-Minute, and 5-Minute Data Resolutions for TBI-BLR Population

| **Physiologic Variable** | **Sub-groups** | | | | | | | | | | | | | |
| --- | --- | --- | --- | --- | --- | --- | --- | --- | --- | --- | --- | --- | --- | --- |
|  | **Age < 40 [n = 4]** | | **Age 40 – 60 [n = 1]** | | **Males [n = 5]** | | **Focal Injury (aSDH, SDH, EDH, or Contusion) [n = 4]** | | **Diffuse Injury (DAI or tSAH) [n = 1]** | | **Marshall CT V [n = 3]** | | **Marshall CT IV [n = 1]** | |
|  | **Median (IQR) or  Median (IQR; MAD)** | **p-value** | **Median (IQR) or  Median (IQR; MAD)** | **p-value** | **Median (IQR) or  Median (IQR; MAD)** | **p-value** | **Median (IQR) or  Median (IQR; MAD)** | **p-value** | **Median (IQR) or  Median (IQR; MAD)** | **p-value** | **Median (IQR) or  Median (IQR; MAD)** | **p-value** | **Median (IQR) or  Median (IQR; MAD)** | **p-value** |
| **10-Second Data Resolution** | | | | | | | | | | | | | | |
| **ABP (mmHg)** | 88.76 (83.5 – 95.64) | – | 88 (82.88 – 95.86) | – | 88 (82.88 – 95.86) | – | 87.12 (81.48 – 94.73) | – | 97.99 (91.42 – 113.8) | – | 86.23 (80.08 – 93.59) | – | 91.29 (86.91 – 97.7) | – |
| **CPP (mmHg)** | 72.27 (66.81 – 84.76) |  | 75.09 (69.96 – 83.73) |  | 73.92 (68.33 – 83.73) |  | 72.27 (66.81 – 82.57) |  | 81.77 (76.42 – 99.76) |  | 73.92 (68.33 – 81.41) |  | 70.62 (65.29 – 88.11) |  |
| **rSO2_L (%)** | 64.15 (60.9 – 72.75) | 0.2 | 68.55 (65 – 79.99) | 1 | 66.48 (61.99 – 74.5) | 0.0952 | 67.52 (63.5 – 74.75) | 0.3429 | 61.81 (59.81 – 63.75) | 1 | 68.55 (65 – 74.99) | 1 | -0.01 (-0.01 – 74.5) | 1 |
| **rSO2_R (%)** | 71.79 (68 – 75.5) |  | 72.74 (68.32 – 83.77) |  | 72.74 (68.32 – 77) |  | 71.14 (66.66 – 78.89) |  | 74.05 (70.99 – 77) |  | 69.53 (65 – 74) |  | 81.52 (79.99 – 84.99) |  |
| **COx_L (au)** | -0.03 (-0.16 – 0.13) | 0.6573 | 0 (-0.19 – 0.17) | – | 0 (-0.19 – 0.16) | 0.6558 | 0 (-0.11 – 0.16) | 0.8687 | -0.09 (-0.3 – 0.1) | 1 | 0 (-0.19 – 0.17) | 1 | 0 (0 – 0) | – |
| **COx_R (au)** | 0 (-0.2 – 0.21) |  | 0 (-0.15 – 0.18) |  | 0 (-0.19 – 0.2) |  | 0 (-0.17 – 0.21) |  | -0.02 (-0.22 – 0.17) |  | 0 (-0.15 – 0.2) |  | 0 (-0.2 – 0.21) |  |
| **COx-a_L (au)** | 0 (-0.11 – 0.19) | 0.6573 | 0 (-0.18 – 0.16) | – | 0 (-0.18 – 0.18) | 0.6558 | 0 (-0.11 – 0.18) | 0.8687 | 0 (-0.18 – 0.18) | 1 | 0 (-0.18 – 0.2) | 1 | 0 (0 – 0) | – |
| **COx-a_R (au)** | 0 (-0.17 – 0.22) |  | 0 (-0.14 – 0.17) |  | 0 (-0.16 – 0.21) |  | 0 (-0.15 – 0.22) |  | -0.02 (-0.21 – 0.16) |  | 0 (-0.14 – 0.23) |  | 0 (-0.18 – 0.21) |  |
| **MAD of ABP (mmHg)** | 8.53 (6.93 – 10.93) | – | 9.04 (9.04 – 9.04) | – | 9.04 (7.29 – 9.77) | – | 8.17 (6.93 – 9.23) | – | 14.4 (14.4 – 14.4) | – | 9.04 (7.45 – 9.41) | – | 7.29 (7.29 – 7.29) | – |
| **MAD of CPP (mmHg)** | 10.09 (8.51 – 11.23) |  | 9.33 (9.33 – 9.33) |  | 9.41 (9.33 – 10.76) |  | 9.37 (8.44 – 9.75) |  | 12.62 (12.62 – 12.62) |  | 9.33 (7.55 – 9.37) |  | 10.76 (10.76 – 10.76) |  |
| **MAD of rSO2_L (%)** | 4.43 (2.2 – 6.12) | 0.3429 | 7.06 (7.06 – 7.06) | 1 | 5.93 (2.94 – 6.69) | 0.3095 | 6.31 (4.45 – 6.78) | 0.3429 | 2.94 (2.94 – 2.94) | 1 | 6.69 (6.31 – 6.88) | 0.2 | 0 (0 – 0) | 1 |
| **MAD of rSO2_R (%)** | 5.63 (4.34 – 6.93) |  | 8.52 (8.52 – 8.52) |  | 6.72 (4.54 – 7.56) |  | 7.14 (5.97 – 7.8) |  | 4.54 (4.54 – 4.54) |  | 7.56 (7.14 – 8.04) |  | 3.75 (3.75 – 3.75) |  |
| **MAD of COx_L (au)** | 0.3 (0.22 – 0.31) | 0.8857 | 0.26 (0.26 – 0.26) | 1 | 0.3 (0.26 – 0.3) | 1 | 0.28 (0.2 – 0.31) | 1 | 0.3 (0.3 – 0.3) | 1 | 0.3 (0.28 – 0.31) | 0.4 | 0 (0 – 0) | 1 |
| **MAD of COx_R (au)** | 0.3 (0.29 – 0.3) |  | 0.25 (0.25 – 0.25) |  | 0.29 (0.29 – 0.3) |  | 0.3 (0.28 – 0.3) |  | 0.29 (0.29 – 0.29) |  | 0.29 (0.27 – 0.3) |  | 0.3 (0.3 – 0.3) |  |
| **MAD of COx-a_L (au)** | 0.28 (0.2 – 0.3) | 1 | 0.25 (0.25 – 0.25) | 1 | 0.27 (0.25 – 0.3) | 1 | 0.28 (0.19 – 0.3) | 0.8857 | 0.27 (0.27 – 0.27) | 1 | 0.3 (0.28 – 0.31) | 0.4 | 0 (0 – 0) | 1 |
| **MAD of COx-a_R (au)** | 0.29 (0.28 – 0.29) |  | 0.23 (0.23 – 0.23) |  | 0.29 (0.28 – 0.29) |  | 0.29 (0.27 – 0.29) |  | 0.28 (0.28 – 0.28) |  | 0.29 (0.26 – 0.29) |  | 0.29 (0.29 – 0.29) |  |
| **% time rSO2_L > 60%** | 77.74 (64.36 – 87.38; 19.19) | 0.2 | 97.38 (97.38 – 97.38; 0) | 1 | 84.08 (71.39 – 97.28; 19.56) | 0.3095 | 90.68 (73.88 – 97.3; 9.86) | 0.8857 | 71.39 (71.39 – 71.39; 0) | 1 | 97.28 (90.68 – 97.33; 0.15) | 0.4 | 43.28 (43.28 – 43.28; 0) | 1 |
| **% time rSO2_R > 60%** | 96.8 (90.92 – 99.95; 4.69) |  | 96.62 (96.62 – 96.62; 0) |  | 96.62 (93.67 – 99.93; 4.92) |  | 95.15 (90.92 – 97.46; 4.69) |  | 99.93 (99.93 – 99.93; 0) |  | 93.67 (88.16 – 95.15; 4.36) |  | 100 (100 – 100; 0) |  |
| **% time rSO2_L > 70%** | 35.4 (20.88 – 43.74; 13.45) | 0.3429 | 43.44 (43.44 – 43.44; 0) | 1 | 43.01 (27.79 – 43.44; 4.34) | 0.2222 | 43.22 (39.2 – 44.06; 2.17) | 0.6857 | 0.12 (0.12 – 0.12; 0) | 1 | 43.44 (35.62 – 44.69; 3.7) | 1 | 43.01 (43.01 – 43.01; 0) | 1 |
| **% time rSO2_R > 70%** | 62.25 (40.16 – 86.33; 36.47) |  | 65.4 (65.4 – 65.4; 0) |  | 65.4 (42.67 – 81.84; 33.71) |  | 54.03 (40.16 – 74.01; 24.29) |  | 81.84 (81.84 – 81.84; 0) |  | 42.67 (37.65 – 54.03; 14.87) |  | 99.83 (99.83 – 99.83; 0) |  |
| **% time rSO2_L > 80%** | 0.24 (0.15 – 1.87; 0.21) | 0.3429 | 24.11 (24.11 – 24.11; 0) | 1 | 0.29 (0.2 – 6.63; 0.43) | 0.3095 | 3.46 (0.27 – 11; 4.77) | 0.6857 | 0 (0 – 0; 0) | 1 | 0.29 (0.24 – 12.2; 0.13) | 1 | 6.63 (6.63 – 6.63; 0) | 1 |
| **% time rSO2_R > 80%** | 8.63 (3.56 – 26.52; 9.28) |  | 29.12 (29.12 – 29.12; 0) |  | 12.52 (4.74 – 29.12; 18.55) |  | 16.93 (3.56 – 38.97; 21.58) |  | 12.52 (12.52 – 12.52; 0) |  | 4.74 (2.38 – 16.93; 7.02) |  | 68.53 (68.53 – 68.53; 0) |  |
| **% time rSO2_L > 90%** | 0 (0 – 0.03; 0) | 0.6198 | 0.72 (0.72 – 0.72; 0) | 1 | 0 (0 – 0.13; 0) | 0.5038 | 0.07 (0 – 0.28; 0.1) | 0.4596 | 0 (0 – 0; 0) | – | 0 (0 – 0.36; 0) | 0.6428 | 0.13 (0.13 – 0.13; 0) | 1 |
| **% time rSO2_R > 90%** | 0 (0 – 0.64; 0.01) |  | 14.96 (14.96 – 14.96; 0) |  | 0.01 (0 – 2.56; 0.01) |  | 1.28 (0.01 – 5.66; 1.9) |  | 0 (0 – 0; 0) |  | 0.01 (0 – 7.49; 0.01) |  | 2.56 (2.56 – 2.56; 0) |  |
| **% time COx_L > 0** | 38.83 (33 – 46.88; 14.36) | 0.3429 | 45.85 (45.85 – 45.85; 0) | 1 | 40.79 (36.87 – 45.85; 7.5) | 0.2222 | 43.32 (35.95 – 50.67; 18.05) | 0.4857 | 36.87 (36.87 – 36.87; 0) | 1 | 45.85 (43.32 – 55.5; 7.5) | 0.7 | 21.42 (21.42 – 21.42; 0) | 1 |
| **% time COx_R > 0** | 46.58 (45.97 – 48.08; 1.18) |  | 46.42 (46.42 – 46.42; 0) |  | 46.42 (46.17 – 46.98; 0.84) |  | 46.3 (45.97 – 47.66; 0.77) |  | 46.98 (46.98 – 46.98; 0) |  | 46.42 (46.3 – 48.9; 0.37) |  | 45.39 (45.39 – 45.39; 0) |  |
| **% time COx_L > 0.2** | 18.76 (15.48 – 26.89; 6.31) | 0.3429 | 21.73 (21.73 – 21.73; 0) | 1 | 21.07 (16.45 – 21.73; 6.86) | 0.1508 | 21.4 (18.95 – 27.38; 6.79) | 0.3429 | 16.45 (16.45 – 16.45; 0) | 1 | 21.73 (21.4 – 33.04; 0.97) | 0.7 | 12.57 (12.57 – 12.57; 0) | 1 |
| **% time COx_R > 0.2** | 25.45 (24.32 – 27.48; 2.82) |  | 23.14 (23.14 – 23.14; 0) |  | 25.09 (23.14 – 25.8; 2.9) |  | 25.45 (24.61 – 27.48; 1.97) |  | 21.99 (21.99 – 21.99; 0) |  | 25.09 (24.12 – 28.81; 2.9) |  | 25.8 (25.8 – 25.8; 0) |  |
| **% time COx_L > 0.3** | 12.43 (10.36 – 18.87; 3.61) | 0.4857 | 13.49 (13.49 – 13.49; 0) | 1 | 13.49 (10.73 – 14.13; 4.1) | 0.3095 | 13.81 (12.44 – 18.87; 3.61) | 0.4857 | 10.73 (10.73 – 10.73; 0) | 1 | 14.13 (13.81 – 23.61; 0.94) | 1 | 9.27 (9.27 – 9.27; 0) | 1 |
| **% time COx_R > 0.3** | 17.99 (16.09 – 20.16; 4.06) |  | 14.05 (14.05 – 14.05; 0) |  | 16.93 (14.05 – 19.05; 4.27) |  | 17.99 (16.21 – 20.16; 3.7) |  | 13.56 (13.56 – 13.56; 0) |  | 16.93 (15.49 – 20.21; 4.27) |  | 19.05 (19.05 – 19.05; 0) |  |
| **% time COx-a_L > 0** | 48.33 (39.95 – 53.76; 13.56) | 0.8857 | 45.88 (45.88 – 45.88; 0) | 1 | 46.6 (45.88 – 50.06; 5.13) | 0.8413 | 46.24 (39.41 – 51.17; 14.09) | 0.4857 | 50.06 (50.06 – 50.06; 0) | 1 | 46.6 (46.24 – 55.74; 1.06) | 0.7 | 20.01 (20.01 – 20.01; 0) | 1 |
| **% time COx-a_R > 0** | 48.16 (46.45 – 50.24; 2.58) |  | 46.89 (46.89 – 46.89; 0) |  | 46.89 (46.48 – 49.85; 0.78) |  | 48.37 (46.79 – 50.24; 2.5) |  | 46.37 (46.37 – 46.37; 0) |  | 49.85 (48.37 – 50.64; 2.36) |  | 46.48 (46.48 – 46.48; 0) |  |
| **% time COx-a_L > 0.2** | 24.19 (20.42 – 29.71; 9.83) | 0.6857 | 20.82 (20.82 – 20.82; 0) | 1 | 23.27 (20.82 – 25.11; 3.63) | 0.5476 | 22.97 (18.58 – 29.71; 9.83) | 0.4857 | 23.27 (23.27 – 23.27; 0) | 1 | 25.11 (22.97 – 34.32; 6.36) | 1 | 11.85 (11.85 – 11.85; 0) | 1 |
| **% time COx-a_R > 0.2** | 26.65 (24.77 – 28.67; 4.54) |  | 22.23 (22.23 – 22.23; 0) |  | 25.94 (22.23 – 27.36; 5.5) |  | 26.65 (25.02 – 28.67; 3.8) |  | 21.24 (21.24 – 21.24; 0) |  | 27.36 (24.8 – 29.97; 7.6) |  | 25.94 (25.94 – 25.94; 0) |  |
| **% time COx-a_L > 0.3** | 15.8 (13.03 – 21.03; 6.22) | 0.6857 | 12.51 (12.51 – 12.51; 0) | 1 | 14.45 (12.51 – 17.15; 4) | 0.5476 | 14.83 (11.58 – 21.03; 6.22) | 0.4857 | 14.45 (14.45 – 14.45; 0) | 1 | 17.15 (14.83 – 24.92; 6.87) | 1 | 8.76 (8.76 – 8.76; 0) | 1 |
| **% time COx-a_R > 0.3** | 19.27 (17.67 – 20.43; 3.33) |  | 13.62 (13.62 – 13.62; 0) |  | 19.18 (13.62 – 19.35; 6.65) |  | 19.27 (17.79 – 20.43; 3.33) |  | 13.14 (13.14 – 13.14; 0) |  | 19.18 (16.4 – 21.42; 6.65) |  | 19.35 (19.35 – 19.35; 0) |  |
| **1-Minute Data Resolution** | | | | | | | | | | | | | | |
| **ABP (mmHg)** | 88.8 (83.58 – 95.59) | – | 88.02 (83.01 – 95.85) | – | 88.02 (83.01 – 95.85) | – | 87.16 (81.58 – 94.75) | – | 97.88 (91.47 – 113.75) | – | 86.31 (80.15 – 93.65) | – | 91.3 (87.02 – 97.53) | – |
| **CPP (mmHg)** | 72.32 (66.88 – 84.89) |  | 75.01 (70.13 – 83.73) |  | 73.85 (68.42 – 83.73) |  | 72.32 (66.88 – 82.56) |  | 81.66 (76.56 – 99.82) |  | 73.85 (68.42 – 81.39) |  | 70.79 (65.34 – 88.39) |  |
| **rSO2_L (%)** | 64.11 (60.78 – 72.74) | 0.2 | 68.59 (65 – 79.99) | 1 | 66.45 (61.75 – 74.49) | 0.0952 | 67.52 (63.37 – 74.74) | 0.3429 | 61.77 (59.81 – 63.67) | 1 | 68.59 (65 – 74.99) | 1 | -0.01 (-0.01 – 74.49) | 1 |
| **rSO2_R (%)** | 71.9 (68.04 – 75.71) |  | 72.67 (68.4 – 83.66) |  | 72.67 (68.4 – 77.19) |  | 71.12 (66.7 – 78.95) |  | 74.24 (71.08 – 77.19) |  | 69.57 (65 – 74.23) |  | 81.5 (79.91 – 84.99) |  |
| **COx_L (au)** | -0.01 (-0.22 – 0.21) | 0.8857 | -0.01 (-0.19 – 0.18) | 1 | -0.01 (-0.19 – 0.18) | 0.6905 | 0.01 (-0.18 – 0.22) | 0.8857 | -0.1 (-0.29 – 0.1) | 1 | -0.01 (-0.19 – 0.18) | 0.7 | 0.04 (-0.17 – 0.27) | 1 |
| **COx_R (au)** | 0 (-0.21 – 0.22) |  | 0.02 (-0.18 – 0.2) |  | 0 (-0.21 – 0.21) |  | 0.01 (-0.19 – 0.22) |  | -0.02 (-0.21 – 0.16) |  | 0.02 (-0.18 – 0.21) |  | 0 (-0.21 – 0.22) |  |
| **COx-a_L (au)** | 0 (-0.18 – 0.23) | 1 | -0.01 (-0.19 – 0.16) | 1 | 0 (-0.19 – 0.2) | 0.6905 | 0 (-0.19 – 0.23) | 0.4857 | 0 (-0.17 – 0.17) | 1 | -0.01 (-0.19 – 0.2) | 0.7 | 0.01 (-0.19 – 0.25) | 1 |
| **COx-a_R (au)** | 0.02 (-0.18 – 0.24) |  | 0.02 (-0.16 – 0.19) |  | 0.02 (-0.18 – 0.23) |  | 0.02 (-0.17 – 0.24) |  | -0.02 (-0.2 – 0.16) |  | 0.03 (-0.16 – 0.24) |  | 0 (-0.19 – 0.23) |  |
| **MAD of ABP (mmHg)** | 8.35 (6.71 – 10.79) | – | 8.8 (8.8 – 8.8) | – | 8.8 (7.04 – 9.65) | – | 7.92 (6.71 – 9.01) | – | 14.22 (14.22 – 14.22) | – | 8.8 (7.26 – 9.23) | – | 7.04 (7.04 – 7.04) | – |
| **MAD of CPP (mmHg)** | 10.14 (8.39 – 11.32) |  | 9.12 (9.12 – 9.12) |  | 9.32 (9.12 – 10.97) |  | 9.22 (8.24 – 9.73) |  | 12.38 (12.38 – 12.38) |  | 9.12 (7.37 – 9.22) |  | 10.97 (10.97 – 10.97) |  |
| **MAD of rSO2_L (%)** | 4.17 (2.16 – 5.81) | 0.4857 | 7.16 (7.16 – 7.16) | 1 | 5.47 (2.88 – 6.83) | 0.4206 | 6.15 (4.1 – 6.91) | 0.4857 | 2.88 (2.88 – 2.88) | 1 | 6.83 (6.15 – 6.99) | 0.4 | 0 (0 – 0) | 1 |
| **MAD of rSO2_R (%)** | 5.67 (4.41 – 7.03) |  | 8.47 (8.47 – 8.47) |  | 6.77 (4.57 – 7.8) |  | 7.28 (6.06 – 7.97) |  | 4.57 (4.57 – 4.57) |  | 7.8 (7.28 – 8.13) |  | 3.94 (3.94 – 3.94) |  |
| **MAD of COx_L (au)** | 0.32 (0.31 – 0.32) | 0.8857 | 0.27 (0.27 – 0.27) | 1 | 0.32 (0.29 – 0.32) | 1 | 0.32 (0.31 – 0.32) | 1 | 0.29 (0.29 – 0.29) | 1 | 0.32 (0.3 – 0.32) | 1 | 0.33 (0.33 – 0.33) | 1 |
| **MAD of COx_R (au)** | 0.32 (0.3 – 0.33) |  | 0.28 (0.28 – 0.28) |  | 0.31 (0.28 – 0.32) |  | 0.32 (0.3 – 0.33) |  | 0.28 (0.28 – 0.28) |  | 0.31 (0.3 – 0.33) |  | 0.32 (0.32 – 0.32) |  |
| **MAD of COx-a_L (au)** | 0.32 (0.3 – 0.32) | 0.8857 | 0.26 (0.26 – 0.26) | 1 | 0.32 (0.26 – 0.32) | 1 | 0.32 (0.31 – 0.32) | 0.6857 | 0.26 (0.26 – 0.26) | 1 | 0.32 (0.29 – 0.32) | 1 | 0.33 (0.33 – 0.33) | 1 |
| **MAD of COx-a_R (au)** | 0.31 (0.3 – 0.32) |  | 0.26 (0.26 – 0.26) |  | 0.31 (0.26 – 0.31) |  | 0.31 (0.3 – 0.32) |  | 0.26 (0.26 – 0.26) |  | 0.31 (0.29 – 0.32) |  | 0.31 (0.31 – 0.31) |  |
| **% time rSO2_L > 60%** | 78.79 (65.2 – 88.13; 18.35) | 0.2 | 97.6 (97.6 – 97.6; 0) | 1 | 85.09 (72.49 – 97.24; 18.55) | 0.3095 | 91.17 (74.65 – 97.33; 9.28) | 0.8857 | 72.49 (72.49 – 72.49; 0) | 1 | 97.24 (91.17 – 97.42; 0.54) | 0.4 | 43.32 (43.32 – 43.32; 0) | 1 |
| **% time rSO2_R > 60%** | 96.82 (91.22 – 99.94; 4.65) |  | 96.64 (96.64 – 96.64; 0) |  | 96.64 (93.72 – 99.92; 4.87) |  | 95.18 (91.22 – 97.48; 4.65) |  | 99.92 (99.92 – 99.92; 0) |  | 93.72 (88.71 – 95.18; 4.32) |  | 100 (100 – 100; 0) |  |
| **% time rSO2_L > 70%** | 36.06 (21.77 – 44.1; 13.33) | 0.2 | 43.97 (43.97 – 43.97; 0) | 1 | 43.15 (28.97 – 43.97; 5.65) | 0.0952 | 43.56 (39.6 – 44.71; 2.82) | 0.3429 | 0.15 (0.15 – 0.15; 0) | 1 | 43.97 (36.47 – 45.46; 4.43) | 0.7 | 43.15 (43.15 – 43.15; 0) | 1 |
| **% time rSO2_R > 70%** | 63.55 (41.28 – 87.21; 37.25) |  | 66.49 (66.49 – 66.49; 0) |  | 66.49 (44.13 – 82.96; 33.14) |  | 55.31 (41.28 – 74.85; 25.03) |  | 82.96 (82.96 – 82.96; 0) |  | 44.13 (38.42 – 55.31; 16.92) |  | 99.95 (99.95 – 99.95; 0) |  |
| **% time rSO2_L > 80%** | 0.3 (0.15 – 2.01; 0.3) | 0.3836 | 24.68 (24.68 – 24.68; 0) | 1 | 0.4 (0.21 – 6.81; 0.6) | 0.3457 | 3.61 (0.35 – 11.28; 4.9) | 0.6857 | 0 (0 – 0; 0) | 1 | 0.4 (0.3 – 12.54; 0.29) | 1 | 6.81 (6.81 – 6.81; 0) | 1 |
| **% time rSO2_R > 80%** | 8.95 (3.8 – 27.37; 9.51) |  | 29.27 (29.27 – 29.27; 0) |  | 12.83 (5.07 – 29.27; 19.02) |  | 17.17 (3.8 – 39.71; 21.7) |  | 12.83 (12.83 – 12.83; 0) |  | 5.07 (2.53 – 17.17; 7.52) |  | 71.02 (71.02 – 71.02; 0) |  |
| **% time rSO2_L > 90%** | 0 (0 – 0.03; 0) | 0.6198 | 0.88 (0.88 – 0.88; 0) | 1 | 0 (0 – 0.11; 0) | 0.5038 | 0.06 (0 – 0.31; 0.08) | 0.4596 | 0 (0 – 0; 0) | – | 0 (0 – 0.44; 0) | 0.6428 | 0.11 (0.11 – 0.11; 0) | 1 |
| **% time rSO2_R > 90%** | 0.01 (0 – 0.66; 0.01) |  | 15.2 (15.2 – 15.2; 0) |  | 0.01 (0 – 2.61; 0.02) |  | 1.31 (0.01 – 5.76; 1.94) |  | 0 (0 – 0; 0) |  | 0.01 (0.01 – 7.6; 0.02) |  | 2.61 (2.61 – 2.61; 0) |  |
| **% time COx_L > 0** | 48.29 (40.73 – 58.6; 13.27) | 0.8857 | 48.53 (48.53 – 48.53; 0) | 1 | 48.53 (42.11 – 54.47; 9.51) | 0.6905 | 51.5 (46.93 – 58.6; 9.16) | 0.8857 | 36.56 (36.56 – 36.56; 0) | 1 | 48.53 (45.32 – 59.76; 9.51) | 0.7 | 54.47 (54.47 – 54.47; 0) | 1 |
| **% time COx_R > 0** | 49.86 (48.83 – 52.9; 2.5) |  | 52.13 (52.13 – 52.13; 0) |  | 50.22 (49.49 – 52.13; 2.82) |  | 51.18 (50.04 – 54.32; 1.95) |  | 46.85 (46.85 – 46.85; 0) |  | 52.13 (51.18 – 56.52; 2.82) |  | 49.49 (49.49 – 49.49; 0) |  |
| **% time COx_L > 0.2** | 25.91 (19.66 – 35.02; 11.35) | 0.8857 | 22 (22 – 22; 0) | 1 | 22 (21.06 – 30.76; 9.71) | 0.6905 | 26.38 (21.77 – 35.02; 7.19) | 0.8857 | 15.45 (15.45 – 15.45; 0) | 1 | 22 (21.53 – 34.89; 1.39) | 0.7 | 30.76 (30.76 – 30.76; 0) | 1 |
| **% time COx_R > 0.2** | 26.55 (25.12 – 29.36; 4.1) |  | 25.54 (25.54 – 25.54; 0) |  | 26.44 (25.54 – 26.67; 1.33) |  | 26.55 (26.22 – 29.36; 0.83) |  | 21.14 (21.14 – 21.14; 0) |  | 26.44 (25.99 – 31.94; 1.33) |  | 26.67 (26.67 – 26.67; 0) |  |
| **% time COx_L > 0.3** | 18.4 (13.02 – 25.69; 9.5) | 1 | 13.62 (13.62 – 13.62; 0) | 1 | 14.04 (13.62 – 22.77; 6.07) | 0.8413 | 18.4 (13.93 – 25.69; 6.78) | 0.8857 | 9.95 (9.95 – 9.95; 0) | 1 | 14.04 (13.83 – 24.26; 0.61) | 0.7 | 22.77 (22.77 – 22.77; 0) | 1 |
| **% time COx_R > 0.3** | 18.89 (16.51 – 21.75; 5.42) |  | 14.98 (14.98 – 14.98; 0) |  | 17.78 (14.98 – 20; 4.15) |  | 18.89 (17.08 – 21.75; 3.72) |  | 12.69 (12.69 – 12.69; 0) |  | 17.78 (16.38 – 22.38; 4.15) |  | 20 (20 – 20; 0) |  |
| **% time COx-a_L > 0** | 50.71 (49.62 – 56.28; 2.18) | 1 | 48.59 (48.59 – 48.59; 0) | 1 | 50 (48.59 – 51.42; 2.11) | 0.6905 | 50.01 (48.56 – 56.28; 2.18) | 0.4857 | 50 (50 – 50; 0) | 1 | 48.59 (48.54 – 59.72; 0.15) | 0.7 | 51.42 (51.42 – 51.42; 0) | 1 |
| **% time COx-a_R > 0** | 52.47 (49.76 – 55.68; 5.66) |  | 52.46 (52.46 – 52.46; 0) |  | 52.46 (50.87 – 54.08; 2.4) |  | 53.27 (52.06 – 55.68; 2.39) |  | 46.45 (46.45 – 46.45; 0) |  | 54.08 (53.27 – 57.28; 2.4) |  | 50.87 (50.87 – 50.87; 0) |  |
| **% time COx-a_L > 0.2** | 27.41 (24.36 – 33.92; 5.59) | 0.8857 | 21.25 (21.25 – 21.25; 0) | 1 | 25.09 (22.18 – 29.73; 5.69) | 1 | 27.41 (24.13 – 33.92; 6.29) | 1 | 22.18 (22.18 – 22.18; 0) | 1 | 25.09 (23.17 – 35.79; 5.69) | 1 | 29.73 (29.73 – 29.73; 0) | 1 |
| **% time COx-a_R > 0.2** | 28.13 (25.54 – 31.02; 6.45) |  | 24.22 (24.22 – 24.22; 0) |  | 27.3 (24.22 – 28.95; 4.57) |  | 28.13 (26.53 – 31.02; 3.5) |  | 20.25 (20.25 – 20.25; 0) |  | 28.95 (26.59 – 33.09; 7) |  | 27.3 (27.3 – 27.3; 0) |  |
| **% time COx-a_L > 0.3** | 19.2 (16.15 – 24.64; 5.7) | 0.8857 | 12.37 (12.37 – 12.37; 0) | 1 | 16.93 (13.79 – 21.48; 6.75) | 1 | 19.2 (15.79 – 24.64; 6.76) | 1 | 13.79 (13.79 – 13.79; 0) | 1 | 16.93 (14.65 – 25.52; 6.77) | 1 | 21.48 (21.48 – 21.48; 0) | 1 |
| **% time COx-a_R > 0.3** | 20.33 (18.23 – 22.05; 4.95) |  | 14.45 (14.45 – 14.45; 0) |  | 20.22 (14.45 – 20.44; 8.56) |  | 20.33 (18.78 – 22.05; 4.44) |  | 12.28 (12.28 – 12.28; 0) |  | 20.22 (17.33 – 23.56; 8.56) |  | 20.44 (20.44 – 20.44; 0) |  |
| **5-Minute Data Resolution** | | | | | | | | | | | | | | |
| **ABP (mmHg)** | 88.87 (83.66 – 95.36) | – | 88.08 (83.18 – 96.03) | – | 88.08 (83.18 – 96.03) | – | 87.29 (81.75 – 94.77) | – | 97.84 (91.38 – 113.77) | – | 86.51 (80.32 – 93.51) | – | 91.24 (87.01 – 97.21) | – |
| **CPP (mmHg)** | 72.22 (66.98 – 84.87) |  | 75.2 (70.27 – 84.02) |  | 73.78 (68.64 – 84.02) |  | 72.22 (66.98 – 82.66) |  | 81.56 (76.58 – 100.07) |  | 73.78 (68.64 – 81.29) |  | 70.66 (65.32 – 88.45) |  |
| **rSO2_L (%)** | 64.1 (60.77 – 72.84) | 0.2 | 68.58 (65.08 – 79.88) | 1 | 66.44 (61.67 – 74.69) | 0.0952 | 67.51 (63.37 – 74.83) | 0.3429 | 61.75 (59.86 – 63.64) | 1 | 68.58 (65.08 – 74.97) | 1 | -0.01 (-0.01 – 74.69) | 1 |
| **rSO2_R (%)** | 71.89 (68.04 – 75.74) |  | 72.64 (68.43 – 83.45) |  | 72.64 (68.43 – 77.19) |  | 71.08 (66.68 – 78.87) |  | 74.27 (71.15 – 77.19) |  | 69.52 (64.93 – 74.29) |  | 81.43 (79.88 – 84.99) |  |
| **COx_L (au)** | -0.02 (-0.19 – 0.17) | 0.8857 | 0 (-0.17 – 0.15) | 1 | 0 (-0.17 – 0.15) | 0.8413 | 0.01 (-0.16 – 0.18) | 1 | -0.1 (-0.26 – 0.06) | 1 | 0 (-0.17 – 0.15) | 0.7 | 0.02 (-0.15 – 0.21) | 1 |
| **COx_R (au)** | 0 (-0.17 – 0.18) |  | 0.01 (-0.15 – 0.17) |  | 0 (-0.17 – 0.18) |  | 0.01 (-0.16 – 0.18) |  | -0.02 (-0.17 – 0.13) |  | 0.01 (-0.15 – 0.18) |  | -0.01 (-0.17 – 0.18) |  |
| **COx-a_L (au)** | 0 (-0.15 – 0.17) | 1 | -0.01 (-0.16 – 0.14) | 1 | -0.01 (-0.16 – 0.16) | 0.6905 | 0 (-0.16 – 0.17) | 0.4857 | -0.01 (-0.14 – 0.15) | 1 | -0.01 (-0.16 – 0.16) | 0.7 | 0.01 (-0.17 – 0.18) | 1 |
| **COx-a_R (au)** | 0.02 (-0.14 – 0.2) |  | 0.01 (-0.13 – 0.17) |  | 0.01 (-0.14 – 0.19) |  | 0.02 (-0.14 – 0.2) |  | -0.02 (-0.17 – 0.13) |  | 0.03 (-0.13 – 0.21) |  | 0 (-0.14 – 0.19) |  |
| **MAD of ABP (mmHg)** | 8.24 (6.57 – 10.75) | – | 8.73 (8.73 – 8.73) | – | 8.73 (6.9 – 9.59) | – | 7.82 (6.57 – 8.95) | – | 14.23 (14.23 – 14.23) | – | 8.73 (7.16 – 9.16) | – | 6.9 (6.9 – 6.9) | – |
| **MAD of CPP (mmHg)** | 9.98 (8.28 – 11.12) |  | 9.12 (9.12 – 9.12) |  | 9.17 (9.12 – 10.78) |  | 9.15 (8.24 – 9.57) |  | 12.14 (12.14 – 12.14) |  | 9.12 (7.36 – 9.15) |  | 10.78 (10.78 – 10.78) |  |
| **MAD of rSO2_L (%)** | 4.03 (2.1 – 5.68) | 0.4857 | 7.09 (7.09 – 7.09) | 1 | 5.25 (2.8 – 6.94) | 0.4206 | 6.1 (3.94 – 6.98) | 0.4857 | 2.8 (2.8 – 2.8) | 1 | 6.94 (6.1 – 7.02) | 0.4 | 0 (0 – 0) | 1 |
| **MAD of rSO2_R (%)** | 5.68 (4.4 – 7.01) |  | 8.43 (8.43 – 8.43) |  | 6.84 (4.51 – 7.52) |  | 7.18 (6.15 – 7.75) |  | 4.51 (4.51 – 4.51) |  | 7.52 (7.18 – 7.98) |  | 4.06 (4.06 – 4.06) |  |
| **MAD of COx_L (au)** | 0.26 (0.25 – 0.27) | 1 | 0.24 (0.24 – 0.24) | 1 | 0.26 (0.24 – 0.26) | 1 | 0.26 (0.25 – 0.27) | 0.8857 | 0.23 (0.23 – 0.23) | 1 | 0.26 (0.25 – 0.26) | 0.7 | 0.26 (0.26 – 0.26) | 1 |
| **MAD of COx_R (au)** | 0.26 (0.25 – 0.27) |  | 0.24 (0.24 – 0.24) |  | 0.26 (0.24 – 0.27) |  | 0.26 (0.25 – 0.27) |  | 0.22 (0.22 – 0.22) |  | 0.27 (0.25 – 0.27) |  | 0.26 (0.26 – 0.26) |  |
| **MAD of COx-a_L (au)** | 0.25 (0.24 – 0.26) | 1 | 0.22 (0.22 – 0.22) | 1 | 0.25 (0.22 – 0.26) | 1 | 0.25 (0.24 – 0.26) | 1 | 0.22 (0.22 – 0.22) | 1 | 0.25 (0.24 – 0.26) | 1 | 0.26 (0.26 – 0.26) | 1 |
| **MAD of COx-a_R (au)** | 0.25 (0.23 – 0.27) |  | 0.22 (0.22 – 0.22) |  | 0.24 (0.22 – 0.26) |  | 0.25 (0.23 – 0.27) |  | 0.22 (0.22 – 0.22) |  | 0.26 (0.24 – 0.28) |  | 0.24 (0.24 – 0.24) |  |
| **% time rSO2_L > 60%** | 78.94 (65.51 – 88.04; 17.88) | 0.2 | 97.87 (97.87 – 97.87; 0) | 1 | 85.08 (72.8 – 96.91; 18.21) | 0.3095 | 91 (74.73 – 97.15; 9.48) | 0.8857 | 72.8 (72.8 – 72.8; 0) | 1 | 96.91 (91 – 97.39; 1.42) | 0.4 | 43.66 (43.66 – 43.66; 0) | 1 |
| **% time rSO2_R > 60%** | 96.64 (91.03 – 99.91; 4.9) |  | 96.62 (96.62 – 96.62; 0) |  | 96.62 (93.39 – 99.88; 4.84) |  | 95.01 (91.03 – 97.46; 4.9) |  | 99.88 (99.88 – 99.88; 0) |  | 93.39 (88.67 – 95.01; 4.79) |  | 100 (100 – 100; 0) |  |
| **% time rSO2_L > 70%** | 36.47 (21.95 – 44.63; 13.54) | 0.2 | 44.2 (44.2 – 44.2; 0) | 1 | 43.66 (29.27 – 44.2; 5.74) | 0.0952 | 43.93 (40.06 – 45.04; 2.87) | 0.3429 | 0 (0 – 0; 0) | 1 | 44.2 (36.74 – 45.87; 4.93) | 0.7 | 43.66 (43.66 – 43.66; 0) | 1 |
| **% time rSO2_R > 70%** | 64.04 (41.63 – 87.61; 37.63) |  | 66.44 (66.44 – 66.44; 0) |  | 66.44 (44.61 – 83.47; 32.38) |  | 55.52 (41.63 – 74.83; 25) |  | 83.47 (83.47 – 83.47; 0) |  | 44.61 (38.66 – 55.52; 17.63) |  | 100 (100 – 100; 0) |  |
| **% time rSO2_L > 80%** | 0.21 (0 – 2.07; 0.31) | 0.3005 | 24.78 (24.78 – 24.78; 0) | 1 | 0.41 (0 – 7.04; 0.61) | 0.2903 | 3.73 (0.31 – 11.48; 5.22) | 0.5614 | 0 (0 – 0; 0) | 1 | 0.41 (0.21 – 12.6; 0.61) | 0.8248 | 7.04 (7.04 – 7.04; 0) | 1 |
| **% time rSO2_R > 80%** | 9.03 (3.99 – 27.88; 9.45) |  | 29.15 (29.15 – 29.15; 0) |  | 12.75 (5.32 – 29.15; 18.9) |  | 17.24 (3.99 – 40.18; 21.61) |  | 12.75 (12.75 – 12.75; 0) |  | 5.32 (2.66 – 17.24; 7.88) |  | 73.26 (73.26 – 73.26; 0) |  |
| **% time rSO2_L > 90%** | 0 (0 – 0; 0) | 0.4533 | 0.82 (0.82 – 0.82; 0) | 1 | 0 (0 – 0; 0) | 0.4407 | 0 (0 – 0.2; 0) | 0.4084 | 0 (0 – 0; 0) | – | 0 (0 – 0.41; 0) | 1 | 0 (0 – 0; 0) | 1 |
| **% time rSO2_R > 90%** | 0 (0 – 0.64; 0) |  | 15.35 (15.35 – 15.35; 0) |  | 0 (0 – 2.57; 0) |  | 1.29 (0 – 5.77; 1.91) |  | 0 (0 – 0; 0) |  | 0 (0 – 7.68; 0) |  | 2.57 (2.57 – 2.57; 0) |  |
| **% time COx_L > 0** | 47.31 (39.11 – 58.8; 15.1) | 0.8857 | 49.07 (49.07 – 49.07; 0) | 1 | 49.07 (41.1 – 53.51; 11.81) | 0.8413 | 51.29 (47.08 – 58.8; 9.2) | 1 | 33.15 (33.15 – 33.15; 0) | 1 | 49.07 (45.09 – 61.87; 11.81) | 0.7 | 53.51 (53.51 – 53.51; 0) | 1 |
| **% time COx_R > 0** | 49.51 (48.09 – 52.81; 3.16) |  | 52.32 (52.32 – 52.32; 0) |  | 50.22 (48.8 – 52.32; 3.12) |  | 51.27 (49.87 – 54.39; 2.61) |  | 45.96 (45.96 – 45.96; 0) |  | 52.32 (51.27 – 56.45; 3.12) |  | 48.8 (48.8 – 48.8; 0) |  |
| **% time COx_L > 0.2** | 21.95 (16.29 – 31.29; 10.15) | 1 | 18.74 (18.74 – 18.74; 0) | 1 | 18.74 (17.47 – 26.42; 8.91) | 0.8413 | 22.58 (18.42 – 31.29; 6.64) | 0.8857 | 12.73 (12.73 – 12.73; 0) | 1 | 18.74 (18.11 – 32.32; 1.89) | 0.7 | 26.42 (26.42 – 26.42; 0) | 1 |
| **% time COx_R > 0.2** | 22.71 (21.1 – 25.54; 4.71) |  | 21.83 (21.83 – 21.83; 0) |  | 22.67 (21.83 – 22.76; 1.23) |  | 22.71 (22.46 – 25.54; 0.69) |  | 16.41 (16.41 – 16.41; 0) |  | 22.76 (22.3 – 28.31; 1.38) |  | 22.67 (22.67 – 22.67; 0) |  |
| **% time COx_L > 0.3** | 14.29 (10.27 – 20.89; 7.34) | 1 | 9.83 (9.83 – 9.83; 0) | 1 | 11.2 (9.83 – 17.39; 5.49) | 1 | 14.29 (10.85 – 20.89; 5.61) | 1 | 7.49 (7.49 – 7.49; 0) | 1 | 11.2 (10.51 – 21.29; 2.03) | 1 | 17.39 (17.39 – 17.39; 0) | 1 |
| **% time COx_R > 0.3** | 14.53 (12.44 – 17.22; 4.8) |  | 11.04 (11.04 – 11.04; 0) |  | 13.59 (11.04 – 15.47; 3.79) |  | 14.53 (12.95 – 17.22; 3.28) |  | 8.98 (8.98 – 8.98; 0) |  | 13.59 (12.31 – 18.04; 3.79) |  | 15.47 (15.47 – 15.47; 0) |  |
| **% time COx-a_L > 0** | 50.11 (48.64 – 57.04; 2.81) | 1 | 49.02 (49.02 – 49.02; 0) | 1 | 49.06 (49.02 – 51.16; 2.5) | 0.6905 | 50.09 (48.61 – 57.04; 2.81) | 0.4857 | 49.06 (49.06 – 49.06; 0) | 1 | 49.02 (48.2 – 61.85; 2.44) | 0.7 | 51.16 (51.16 – 51.16; 0) | 1 |
| **% time COx-a_R > 0** | 52.96 (49.22 – 56.84; 7.29) |  | 52.06 (52.06 – 52.06; 0) |  | 52.06 (50.4 – 55.52; 5.13) |  | 53.79 (51.65 – 56.84; 3.8) |  | 45.7 (45.7 – 45.7; 0) |  | 55.52 (53.79 – 58.16; 5.13) |  | 50.4 (50.4 – 50.4; 0) |  |
| **% time COx-a_L > 0.2** | 22.83 (21.37 – 28.84; 3.19) | 0.8857 | 17.26 (17.26 – 17.26; 0) | 1 | 22.07 (19.29 – 23.59; 4.12) | 0.8413 | 22.83 (20.86 – 28.84; 4.69) | 0.6857 | 19.29 (19.29 – 19.29; 0) | 1 | 22.07 (19.66 – 33.33; 7.13) | 1 | 23.59 (23.59 – 23.59; 0) | 1 |
| **% time COx-a_R > 0.2** | 25.42 (22.73 – 27.67; 5.95) |  | 20.35 (20.35 – 20.35; 0) |  | 24.93 (20.35 – 25.91; 6.79) |  | 25.42 (23.79 – 27.67; 4.12) |  | 16.14 (16.14 – 16.14; 0) |  | 25.91 (23.13 – 29.44; 8.24) |  | 24.93 (24.93 – 24.93; 0) |  |
| **% time COx-a_L > 0.3** | 15.15 (12.17 – 20.12; 6.17) | 1 | 8.96 (8.96 – 8.96; 0) | 1 | 13.36 (8.96 – 16.94; 6.53) | 1 | 15.15 (12.26 – 20.12; 5.92) | 0.8857 | 8.61 (8.61 – 8.61; 0) | 1 | 13.36 (11.16 – 21.51; 6.53) | 1 | 16.94 (16.94 – 16.94; 0) | 1 |
| **% time COx-a_R > 0.3** | 17.69 (14.14 – 20.26; 5.13) |  | 10.53 (10.53 – 10.53; 0) |  | 16.02 (10.53 – 19.36; 8.14) |  | 17.69 (14.65 – 20.26; 5.13) |  | 8.48 (8.48 – 8.48; 0) |  | 16.02 (13.28 – 19.48; 8.14) |  | 19.36 (19.36 – 19.36; 0) |  |
| *The p-values in the table are derived using Mann-Whitney U test between the bilateral signals.*  *ABP, arterial blood pressure; au, arbitrary units; CPP, cerebral perfusion pressure; COx, cerebral oximetry index with CPP; COx-a, cerebral oximetry index with ABP; CT, computed tomography; DAI, diffuse axonal injury; EDH, epidural hematoma; MAD, median absolute deviation; IQR, interquartile range; mmHg, millimeters of mercury; rSO2, regional cerebral oxygen saturation; tSAH, traumatic subarachnoid hemorrhage; SDH, subdural hematoma; aSDH, acute subdural hematoma; TBI-BLR, traumatic brain injury patient group with bifrontal lobe pathology.* | | | | | | | | | | | | | | |

| **Physiologic Variable** | **Sub-groups** | | | | | | | | | | | |
| --- | --- | --- | --- | --- | --- | --- | --- | --- | --- | --- | --- | --- |
|  | **Rotterdam CT 6 [n = 2]** | | **Rotterdam CT 4 [n = 3]** | | **Fentanyl + Propofol [n = 1]** | | **Fentanyl + Ketamine + Propofol [n = 1]** | | **Fentanyl + Midazolam + Propofol [n = 1]** | | **Fentanyl + Ketamine + Midazolam + Propofol [n = 2]** | |
|  | **Median (IQR) or  Median (IQR; MAD)** | **p-value** | **Median (IQR) or  Median (IQR; MAD)** | **p-value** | **Median (IQR) or  Median (IQR; MAD)** | **p-value** | **Median (IQR) or  Median (IQR; MAD)** | **p-value** | **Median (IQR) or  Median (IQR; MAD)** | **p-value** | **Median (IQR) or  Median (IQR; MAD)** | **p-value** |
| **10-Second Data Resolution** | | | | | | | | | | | | |
| **ABP (mmHg)** | 79.33 (74.31 – 85.02) | – | 91.29 (86.91 – 97.7) | – | 72.42 (68.54 – 76.44) | – | 91.29 (86.91 – 97.7) | – | 88 (82.88 – 95.86) | – | 92.11 (85.75 – 103.7) | – |
| **CPP (mmHg)** | 70.43 (65.93 – 76.45) |  | 75.09 (69.96 – 88.11) |  | 66.94 (63.53 – 71.49) |  | 70.62 (65.29 – 88.11) |  | 75.09 (69.96 – 83.73) |  | 77.85 (72.38 – 90.58) |  |
| **rSO2_L (%)** | 68.24 (64.49 – 72.99) | 0.6667 | 61.81 (59.81 – 74.5) | 0.1 | 69.99 (66.99 – 74.99) | 1 | -0.01 (-0.01 – 74.5) | 1 | 68.55 (65 – 79.99) | 1 | 64.15 (60.9 – 67.37) | 0.3333 |
| **rSO2_R (%)** | 67.75 (63.5 – 73.07) |  | 74.05 (70.99 – 83.77) |  | 65.96 (61.99 – 72.13) |  | 81.52 (79.99 – 84.99) |  | 72.74 (68.32 – 83.77) |  | 71.79 (68 – 75.5) |  |
| **COx_L (au)** | 0.05 (-0.16 – 0.27) | 1 | 0 (-0.19 – 0.1) | 1 | 0.15 (-0.03 – 0.38) | 1 | 0 (0 – 0) | – | 0 (-0.19 – 0.17) | – | -0.07 (-0.29 – 0.13) | 0.3333 |
| **COx_R (au)** | 0.01 (-0.15 – 0.24) |  | 0 (-0.2 – 0.18) |  | 0.01 (-0.1 – 0.28) |  | 0 (-0.2 – 0.21) |  | 0 (-0.15 – 0.18) |  | -0.01 (-0.2 – 0.19) |  |
| **COx-a_L (au)** | 0.07 (-0.13 – 0.29) | 1 | 0 (-0.18 – 0.16) | 0.3017 | 0.14 (-0.03 – 0.38) | 1 | 0 (0 – 0) | – | 0 (-0.18 – 0.16) | – | 0 (-0.21 – 0.19) | 0.6667 |
| **COx-a_R (au)** | 0.01 (-0.13 – 0.25) |  | 0 (-0.18 – 0.17) |  | 0.02 (-0.1 – 0.28) |  | 0 (-0.18 – 0.21) |  | 0 (-0.14 – 0.17) |  | -0.01 (-0.19 – 0.19) |  |
| **MAD of ABP (mmHg)** | 7.81 (6.83 – 8.79) | – | 9.04 (8.17 – 11.72) | – | 5.86 (5.86 – 5.86) | – | 7.29 (7.29 – 7.29) | – | 9.04 (9.04 – 9.04) | – | 12.08 (10.93 – 13.24) | – |
| **MAD of CPP (mmHg)** | 7.6 (6.69 – 8.51) |  | 10.76 (10.04 – 11.69) |  | 5.78 (5.78 – 5.78) |  | 10.76 (10.76 – 10.76) |  | 9.33 (9.33 – 9.33) |  | 11.02 (10.22 – 11.82) |  |
| **MAD of rSO2_L (%)** | 6.31 (6.12 – 6.5) | 0.3333 | 2.94 (1.47 – 5) | 0.4 | 5.93 (5.93 – 5.93) | 1 | 0 (0 – 0) | 1 | 7.06 (7.06 – 7.06) | 1 | 4.81 (3.87 – 5.75) | 0.6667 |
| **MAD of rSO2_R (%)** | 7.14 (6.93 – 7.35) |  | 4.54 (4.14 – 6.53) |  | 7.56 (7.56 – 7.56) |  | 3.75 (3.75 – 3.75) |  | 8.52 (8.52 – 8.52) |  | 5.63 (5.08 – 6.17) |  |
| **MAD of COx_L (au)** | 0.31 (0.31 – 0.32) | 0.3333 | 0.26 (0.13 – 0.28) | 0.7 | 0.3 (0.3 – 0.3) | 1 | 0 (0 – 0) | 1 | 0.26 (0.26 – 0.26) | 1 | 0.31 (0.3 – 0.32) | 0.3333 |
| **MAD of COx_R (au)** | 0.3 (0.29 – 0.3) |  | 0.29 (0.27 – 0.3) |  | 0.3 (0.3 – 0.3) |  | 0.3 (0.3 – 0.3) |  | 0.25 (0.25 – 0.25) |  | 0.29 (0.29 – 0.29) |  |
| **MAD of COx-a_L (au)** | 0.31 (0.3 – 0.32) | 0.3333 | 0.25 (0.13 – 0.26) | 0.4 | 0.3 (0.3 – 0.3) | 1 | 0 (0 – 0) | 1 | 0.25 (0.25 – 0.25) | 1 | 0.3 (0.28 – 0.31) | 1 |
| **MAD of COx-a_R (au)** | 0.29 (0.29 – 0.29) |  | 0.28 (0.25 – 0.28) |  | 0.3 (0.3 – 0.3) |  | 0.29 (0.29 – 0.29) |  | 0.23 (0.23 – 0.23) |  | 0.28 (0.28 – 0.29) |  |
| **% time rSO2_L > 60%** | 90.68 (87.38 – 93.98; 9.78) | 0.6667 | 71.39 (57.34 – 84.39; 38.53) | 0.2 | 97.28 (97.28 – 97.28; 0) | 1 | 43.28 (43.28 – 43.28; 0) | 1 | 97.38 (97.38 – 97.38; 0) | 1 | 77.74 (74.57 – 80.91; 9.41) | 0.3333 |
| **% time rSO2_R > 60%** | 88.16 (85.41 – 90.92; 8.17) |  | 99.93 (98.27 – 99.97; 0.1) |  | 82.65 (82.65 – 82.65; 0) |  | 100 (100 – 100; 0) |  | 96.62 (96.62 – 96.62; 0) |  | 96.8 (95.24 – 98.37; 4.64) |  |
| **% time rSO2_L > 70%** | 36.87 (32.33 – 41.4; 13.45) | 1 | 43.01 (21.56 – 43.22; 0.64) | 0.1 | 45.94 (45.94 – 45.94; 0) | 1 | 43.01 (43.01 – 43.01; 0) | 1 | 43.44 (43.44 – 43.44; 0) | 1 | 13.96 (7.04 – 20.88; 20.52) | 0.3333 |
| **% time rSO2_R > 70%** | 37.65 (35.14 – 40.16; 7.44) |  | 81.84 (73.62 – 90.83; 24.36) |  | 32.64 (32.64 – 32.64; 0) |  | 99.83 (99.83 – 99.83; 0) |  | 65.4 (65.4 – 65.4; 0) |  | 62.25 (52.46 – 72.04; 29.04) |  |
| **% time rSO2_L > 80%** | 0.24 (0.22 – 0.27; 0.07) | 1 | 6.63 (3.32 – 15.37; 9.84) | 0.2 | 0.2 (0.2 – 0.2; 0) | 1 | 6.63 (6.63 – 6.63; 0) | 1 | 24.11 (24.11 – 24.11; 0) | 1 | 0.14 (0.07 – 0.22; 0.21) | 0.3333 |
| **% time rSO2_R > 80%** | 2.38 (1.19 – 3.56; 3.51) |  | 29.12 (20.82 – 48.82; 24.6) |  | 0.01 (0.01 – 0.01; 0) |  | 68.53 (68.53 – 68.53; 0) |  | 29.12 (29.12 – 29.12; 0) |  | 8.63 (6.69 – 10.58; 5.77) |  |
| **% time rSO2_L > 90%** | 0 (0 – 0; 0) | 0.6171 | 0.13 (0.07 – 0.43; 0.2) | 0.5066 | 0 (0 – 0; 0) | – | 0.13 (0.13 – 0.13; 0) | 1 | 0.72 (0.72 – 0.72; 0) | 1 | 0 (0 – 0; 0) | 0.6171 |
| **% time rSO2_R > 90%** | 0 (0 – 0.01; 0.01) |  | 2.56 (1.28 – 8.76; 3.79) |  | 0 (0 – 0; 0) |  | 2.56 (2.56 – 2.56; 0) |  | 14.96 (14.96 – 14.96; 0) |  | 0 (0 – 0.01; 0.01) |  |
| **% time COx_L > 0** | 52.97 (46.88 – 59.05; 18.05) | 1 | 36.87 (29.14 – 41.36; 13.32) | 0.2 | 65.14 (65.14 – 65.14; 0) | 1 | 21.42 (21.42 – 21.42; 0) | 1 | 45.85 (45.85 – 45.85; 0) | 1 | 38.83 (37.85 – 39.81; 2.91) | 0.3333 |
| **% time COx_R > 0** | 48.77 (47.47 – 50.07; 3.86) |  | 46.42 (45.9 – 46.7; 0.84) |  | 51.37 (51.37 – 51.37; 0) |  | 45.39 (45.39 – 45.39; 0) |  | 46.42 (46.42 – 46.42; 0) |  | 46.58 (46.37 – 46.78; 0.6) |  |
| **% time COx_L > 0.2** | 32.72 (26.89 – 38.54; 17.26) | 1 | 16.45 (14.51 – 19.09; 5.76) | 0.1 | 44.36 (44.36 – 44.36; 0) | 1 | 12.57 (12.57 – 12.57; 0) | 1 | 21.73 (21.73 – 21.73; 0) | 1 | 18.76 (17.6 – 19.92; 3.43) | 0.3333 |
| **% time COx_R > 0.2** | 28.81 (26.95 – 30.67; 5.51) |  | 23.14 (22.57 – 24.47; 1.7) |  | 32.52 (32.52 – 32.52; 0) |  | 25.8 (25.8 – 25.8; 0) |  | 23.14 (23.14 – 23.14; 0) |  | 23.54 (22.77 – 24.32; 2.3) |  |
| **% time COx_L > 0.3** | 23.61 (18.87 – 28.35; 14.06) | 1 | 10.73 (10 – 12.11; 2.17) | 0.1 | 33.09 (33.09 – 33.09; 0) | 1 | 9.27 (9.27 – 9.27; 0) | 1 | 13.49 (13.49 – 13.49; 0) | 1 | 12.43 (11.58 – 13.28; 2.52) | 0.6667 |
| **% time COx_R > 0.3** | 20.21 (18.57 – 21.84; 4.86) |  | 14.05 (13.81 – 16.55; 0.72) |  | 23.48 (23.48 – 23.48; 0) |  | 19.05 (19.05 – 19.05; 0) |  | 14.05 (14.05 – 14.05; 0) |  | 15.25 (14.41 – 16.09; 2.5) |  |
| **% time COx-a_L > 0** | 55.74 (51.17 – 60.31; 13.56) | 1 | 45.88 (32.95 – 47.97; 6.19) | 0.7 | 64.89 (64.89 – 64.89; 0) | 1 | 20.01 (20.01 – 20.01; 0) | 1 | 45.88 (45.88 – 45.88; 0) | 1 | 48.33 (47.46 – 49.19; 2.56) | 0.6667 |
| **% time COx-a_R > 0** | 50.64 (50.24 – 51.04; 1.18) |  | 46.48 (46.42 – 46.69; 0.17) |  | 51.44 (51.44 – 51.44; 0) |  | 46.48 (46.48 – 46.48; 0) |  | 46.89 (46.89 – 46.89; 0) |  | 48.11 (47.24 – 48.98; 2.58) |  |
| **% time COx-a_L > 0.2** | 34.32 (29.71 – 38.92; 13.65) | 1 | 20.82 (16.34 – 22.05; 3.63) | 0.4 | 43.53 (43.53 – 43.53; 0) | 1 | 11.85 (11.85 – 11.85; 0) | 1 | 20.82 (20.82 – 20.82; 0) | 1 | 24.19 (23.73 – 24.65; 1.36) | 1 |
| **% time COx-a_R > 0.2** | 29.97 (28.67 – 31.28; 3.87) |  | 22.23 (21.74 – 24.09; 1.48) |  | 32.58 (32.58 – 32.58; 0) |  | 25.94 (25.94 – 25.94; 0) |  | 22.23 (22.23 – 22.23; 0) |  | 24.3 (22.77 – 25.83; 4.54) |  |
| **% time COx-a_L > 0.3** | 24.92 (21.03 – 28.81; 11.52) | 1 | 12.51 (10.64 – 13.48; 2.87) | 0.4 | 32.69 (32.69 – 32.69; 0) | 1 | 8.76 (8.76 – 8.76; 0) | 1 | 12.51 (12.51 – 12.51; 0) | 1 | 15.8 (15.12 – 16.47; 2) | 1 |
| **% time COx-a_R > 0.3** | 21.42 (20.3 – 22.55; 3.33) |  | 13.62 (13.38 – 16.48; 0.71) |  | 23.67 (23.67 – 23.67; 0) |  | 19.35 (19.35 – 19.35; 0) |  | 13.62 (13.62 – 13.62; 0) |  | 16.16 (14.65 – 17.67; 4.48) |  |
| **1-Minute Data Resolution** | | | | | | | | | | | | |
| **ABP (mmHg)** | 79.4 (74.38 – 84.99) | – | 91.3 (87.02 – 97.53) | – | 72.49 (68.61 – 76.33) | – | 91.3 (87.02 – 97.53) | – | 88.02 (83.01 – 95.85) | – | 92.09 (85.81 – 103.7) | – |
| **CPP (mmHg)** | 70.36 (66.05 – 76.41) |  | 75.01 (70.13 – 88.39) |  | 66.88 (63.69 – 71.44) |  | 70.79 (65.34 – 88.39) |  | 75.01 (70.13 – 83.73) |  | 77.75 (72.49 – 90.61) |  |
| **rSO2_L (%)** | 68.14 (64.37 – 72.99) | 0.6667 | 61.77 (59.81 – 74.49) | 0.1 | 69.83 (66.99 – 74.99) | 1 | -0.01 (-0.01 – 74.49) | 1 | 68.59 (65 – 79.99) | 1 | 64.11 (60.78 – 67.33) | 0.3333 |
| **rSO2_R (%)** | 67.65 (63.5 – 73.24) |  | 74.24 (71.08 – 83.66) |  | 65.73 (61.99 – 72.24) |  | 81.5 (79.91 – 84.99) |  | 72.67 (68.4 – 83.66) |  | 71.9 (68.04 – 75.71) |  |
| **COx_L (au)** | 0.06 (-0.16 – 0.27) | 1 | -0.01 (-0.19 – 0.18) | 1 | 0.18 (-0.04 – 0.39) | 1 | 0.04 (-0.17 – 0.27) | 1 | -0.01 (-0.19 – 0.18) | 1 | -0.08 (-0.29 – 0.13) | 0.3333 |
| **COx_R (au)** | 0.05 (-0.18 – 0.27) |  | 0 (-0.21 – 0.2) |  | 0.1 (-0.15 – 0.32) |  | 0 (-0.21 – 0.22) |  | 0.02 (-0.18 – 0.2) |  | -0.01 (-0.21 – 0.19) |  |
| **COx-a_L (au)** | 0.08 (-0.14 – 0.3) | 1 | 0 (-0.19 – 0.17) | 1 | 0.17 (-0.04 – 0.39) | 1 | 0.01 (-0.19 – 0.25) | 1 | -0.01 (-0.19 – 0.16) | 1 | -0.01 (-0.2 – 0.19) | 1 |
| **COx-a_R (au)** | 0.06 (-0.16 – 0.28) |  | 0 (-0.19 – 0.19) |  | 0.1 (-0.14 – 0.32) |  | 0 (-0.19 – 0.23) |  | 0.02 (-0.16 – 0.19) |  | 0 (-0.19 – 0.2) |  |
| **MAD of ABP (mmHg)** | 7.69 (6.7 – 8.67) | – | 8.8 (7.92 – 11.51) | – | 5.72 (5.72 – 5.72) | – | 7.04 (7.04 – 7.04) | – | 8.8 (8.8 – 8.8) | – | 11.93 (10.79 – 13.08) | – |
| **MAD of CPP (mmHg)** | 7.46 (6.54 – 8.39) |  | 10.97 (10.04 – 11.67) |  | 5.61 (5.61 – 5.61) |  | 10.97 (10.97 – 10.97) |  | 9.12 (9.12 – 9.12) |  | 10.85 (10.08 – 11.61) |  |
| **MAD of rSO2_L (%)** | 6.15 (5.81 – 6.49) | 0.6667 | 2.88 (1.44 – 5.02) | 0.4 | 5.47 (5.47 – 5.47) | 1 | 0 (0 – 0) | 1 | 7.16 (7.16 – 7.16) | 1 | 4.86 (3.87 – 5.85) | 1 |
| **MAD of rSO2_R (%)** | 7.28 (7.03 – 7.54) |  | 4.57 (4.26 – 6.52) |  | 7.8 (7.8 – 7.8) |  | 3.94 (3.94 – 3.94) |  | 8.47 (8.47 – 8.47) |  | 5.67 (5.12 – 6.22) |  |
| **MAD of COx_L (au)** | 0.32 (0.32 – 0.32) | 1 | 0.29 (0.28 – 0.31) | 1 | 0.32 (0.32 – 0.32) | 1 | 0.33 (0.33 – 0.33) | 1 | 0.27 (0.27 – 0.27) | 1 | 0.31 (0.3 – 0.31) | 0.6667 |
| **MAD of COx_R (au)** | 0.33 (0.32 – 0.33) |  | 0.28 (0.28 – 0.3) |  | 0.34 (0.34 – 0.34) |  | 0.32 (0.32 – 0.32) |  | 0.28 (0.28 – 0.28) |  | 0.29 (0.29 – 0.3) |  |
| **MAD of COx-a_L (au)** | 0.32 (0.32 – 0.32) | 1 | 0.26 (0.26 – 0.29) | 1 | 0.32 (0.32 – 0.32) | 1 | 0.33 (0.33 – 0.33) | 1 | 0.26 (0.26 – 0.26) | 1 | 0.29 (0.27 – 0.31) | 1 |
| **MAD of COx-a_R (au)** | 0.32 (0.32 – 0.33) |  | 0.26 (0.26 – 0.29) |  | 0.34 (0.34 – 0.34) |  | 0.31 (0.31 – 0.31) |  | 0.26 (0.26 – 0.26) |  | 0.29 (0.28 – 0.3) |  |
| **% time rSO2_L > 60%** | 91.17 (88.13 – 94.2; 9.01) | 0.6667 | 72.49 (57.9 – 85.05; 37.23) | 0.2 | 97.24 (97.24 – 97.24; 0) | 1 | 43.32 (43.32 – 43.32; 0) | 1 | 97.6 (97.6 – 97.6; 0) | 1 | 78.79 (75.64 – 81.94; 9.34) | 0.3333 |
| **% time rSO2_R > 60%** | 88.71 (86.21 – 91.22; 7.43) |  | 99.92 (98.28 – 99.96; 0.11) |  | 83.7 (83.7 – 83.7; 0) |  | 100 (100 – 100; 0) |  | 96.64 (96.64 – 96.64; 0) |  | 96.82 (95.27 – 98.37; 4.6) |  |
| **% time rSO2_L > 70%** | 37.96 (33.47 – 42.46; 13.33) | 1 | 43.15 (21.65 – 43.56; 1.22) | 0.1 | 46.95 (46.95 – 46.95; 0) | 1 | 43.15 (43.15 – 43.15; 0) | 1 | 43.97 (43.97 – 43.97; 0) | 1 | 14.56 (7.36 – 21.77; 21.37) | 0.3333 |
| **% time rSO2_R > 70%** | 38.42 (35.57 – 41.28; 8.46) |  | 82.96 (74.72 – 91.45; 24.43) |  | 32.72 (32.72 – 32.72; 0) |  | 99.95 (99.95 – 99.95; 0) |  | 66.49 (66.49 – 66.49; 0) |  | 63.55 (53.84 – 73.25; 28.79) |  |
| **% time rSO2_L > 80%** | 0.3 (0.25 – 0.35; 0.15) | 1 | 6.81 (3.41 – 15.75; 10.1) | 0.2 | 0.21 (0.21 – 0.21; 0) | 1 | 6.81 (6.81 – 6.81; 0) | 1 | 24.68 (24.68 – 24.68; 0) | 1 | 0.2 (0.1 – 0.3; 0.3) | 0.3333 |
| **% time rSO2_R > 80%** | 2.53 (1.27 – 3.8; 3.76) |  | 29.27 (21.05 – 50.14; 24.38) |  | 0 (0 – 0; 0) |  | 71.02 (71.02 – 71.02; 0) |  | 29.27 (29.27 – 29.27; 0) |  | 8.95 (7.01 – 10.89; 5.75) |  |
| **% time rSO2_L > 90%** | 0 (0 – 0; 0) | 0.6171 | 0.11 (0.06 – 0.5; 0.17) | 0.5066 | 0 (0 – 0; 0) | – | 0.11 (0.11 – 0.11; 0) | 1 | 0.88 (0.88 – 0.88; 0) | 1 | 0 (0 – 0; 0) | 0.6171 |
| **% time rSO2_R > 90%** | 0.01 (0 – 0.01; 0.01) |  | 2.61 (1.31 – 8.9; 3.87) |  | 0 (0 – 0; 0) |  | 2.61 (2.61 – 2.61; 0) |  | 15.2 (15.2 – 15.2; 0) |  | 0.01 (0 – 0.01; 0.01) |  |
| **% time COx_L > 0** | 56.55 (49.33 – 63.78; 21.41) | 1 | 48.53 (42.55 – 51.5; 8.8) | 1 | 71 (71 – 71; 0) | 1 | 54.47 (54.47 – 54.47; 0) | 1 | 48.53 (48.53 – 48.53; 0) | 1 | 39.34 (37.95 – 40.73; 4.12) | 0.3333 |
| **% time COx_R > 0** | 55.57 (52.9 – 58.24; 7.92) |  | 49.49 (48.17 – 50.81; 3.91) |  | 60.91 (60.91 – 60.91; 0) |  | 49.49 (49.49 – 49.49; 0) |  | 52.13 (52.13 – 52.13; 0) |  | 48.54 (47.69 – 49.38; 2.5) |  |
| **% time COx_L > 0.2** | 34.42 (27.74 – 41.1; 19.81) | 1 | 22 (18.73 – 26.38; 9.71) | 1 | 47.78 (47.78 – 47.78; 0) | 1 | 30.76 (30.76 – 30.76; 0) | 1 | 22 (22 – 22; 0) | 1 | 18.26 (16.85 – 19.66; 4.16) | 0.3333 |
| **% time COx_R > 0.2** | 31.94 (29.19 – 34.69; 8.15) |  | 25.54 (23.34 – 26.1; 1.67) |  | 37.43 (37.43 – 37.43; 0) |  | 26.67 (26.67 – 26.67; 0) |  | 25.54 (25.54 – 25.54; 0) |  | 23.79 (22.46 – 25.12; 3.93) |  |
| **% time COx_L > 0.3** | 24.26 (19.15 – 29.37; 15.15) | 1 | 13.62 (11.79 – 18.2; 5.45) | 1 | 34.48 (34.48 – 34.48; 0) | 1 | 22.77 (22.77 – 22.77; 0) | 1 | 13.62 (13.62 – 13.62; 0) | 1 | 11.99 (10.97 – 13.02; 3.03) | 0.6667 |
| **% time COx_R > 0.3** | 22.38 (20.08 – 24.68; 6.82) |  | 14.98 (13.84 – 17.49; 3.4) |  | 26.98 (26.98 – 26.98; 0) |  | 20 (20 – 20; 0) |  | 14.98 (14.98 – 14.98; 0) |  | 15.24 (13.96 – 16.51; 3.77) |  |
| **% time COx-a_L > 0** | 59.67 (54.08 – 65.26; 16.58) | 1 | 50 (49.29 – 50.71; 2.09) | 1 | 70.85 (70.85 – 70.85; 0) | 1 | 51.42 (51.42 – 51.42; 0) | 1 | 48.59 (48.59 – 48.59; 0) | 1 | 49.24 (48.86 – 49.62; 1.12) | 1 |
| **% time COx-a_R > 0** | 57.28 (55.68 – 58.89; 4.75) |  | 50.87 (48.66 – 51.66; 2.37) |  | 60.49 (60.49 – 60.49; 0) |  | 50.87 (50.87 – 50.87; 0) |  | 52.46 (52.46 – 52.46; 0) |  | 50.27 (48.36 – 52.18; 5.66) |  |
| **% time COx-a_L > 0.2** | 35.79 (30.44 – 41.14; 15.87) | 1 | 22.18 (21.72 – 25.96; 1.39) | 1 | 46.5 (46.5 – 46.5; 0) | 1 | 29.73 (29.73 – 29.73; 0) | 1 | 21.25 (21.25 – 21.25; 0) | 1 | 23.63 (22.91 – 24.36; 2.15) | 1 |
| **% time COx-a_R > 0.2** | 33.09 (31.02 – 35.16; 6.14) |  | 24.22 (22.24 – 25.76; 4.57) |  | 37.23 (37.23 – 37.23; 0) |  | 27.3 (27.3 – 27.3; 0) |  | 24.22 (24.22 – 24.22; 0) |  | 24.6 (22.42 – 26.77; 6.45) |  |
| **% time COx-a_L > 0.3** | 25.52 (21.23 – 29.82; 12.74) | 1 | 13.79 (13.08 – 17.64; 2.12) | 1 | 34.12 (34.12 – 34.12; 0) | 1 | 21.48 (21.48 – 21.48; 0) | 1 | 12.37 (12.37 – 12.37; 0) | 1 | 15.36 (14.58 – 16.15; 2.32) | 1 |
| **% time COx-a_R > 0.3** | 23.56 (21.89 – 25.22; 4.95) |  | 14.45 (13.36 – 17.44; 3.21) |  | 26.89 (26.89 – 26.89; 0) |  | 20.44 (20.44 – 20.44; 0) |  | 14.45 (14.45 – 14.45; 0) |  | 16.25 (14.26 – 18.23; 5.89) |  |
| **5-Minute Data Resolution** | | | | | | | | | | | | |
| **ABP (mmHg)** | 79.57 (74.51 – 84.86) | – | 91.24 (87.01 – 97.21) | – | 72.64 (68.71 – 76.21) | – | 91.24 (87.01 – 97.21) | – | 88.08 (83.18 – 96.03) | – | 92.17 (85.85 – 103.64) | – |
| **CPP (mmHg)** | 70.37 (66.26 – 76.25) |  | 75.2 (70.27 – 88.45) |  | 66.96 (63.88 – 71.22) |  | 70.66 (65.32 – 88.45) |  | 75.2 (70.27 – 84.02) |  | 77.67 (72.61 – 90.68) |  |
| **rSO2_L (%)** | 68.07 (64.3 – 72.97) | 0.6667 | 61.75 (59.86 – 74.69) | 0.1 | 69.7 (66.93 – 74.97) | 1 | -0.01 (-0.01 – 74.69) | 1 | 68.58 (65.08 – 79.88) | 1 | 64.1 (60.77 – 67.31) | 0.3333 |
| **rSO2_R (%)** | 67.55 (63.36 – 73.24) |  | 74.27 (71.15 – 83.45) |  | 65.58 (61.79 – 72.19) |  | 81.43 (79.88 – 84.99) |  | 72.64 (68.43 – 83.45) |  | 71.89 (68.04 – 75.74) |  |
| **COx_L (au)** | 0.06 (-0.12 – 0.24) | 1 | 0 (-0.17 – 0.15) | 1 | 0.18 (0 – 0.35) | 1 | 0.02 (-0.15 – 0.21) | 1 | 0 (-0.17 – 0.15) | 1 | -0.08 (-0.25 – 0.09) | 0.3333 |
| **COx_R (au)** | 0.04 (-0.14 – 0.23) |  | -0.01 (-0.17 – 0.17) |  | 0.07 (-0.1 – 0.28) |  | -0.01 (-0.17 – 0.18) |  | 0.01 (-0.15 – 0.17) |  | -0.01 (-0.17 – 0.16) |  |
| **COx-a_L (au)** | 0.08 (-0.1 – 0.25) | 1 | -0.01 (-0.16 – 0.15) | 1 | 0.17 (0 – 0.34) | 1 | 0.01 (-0.17 – 0.18) | 1 | -0.01 (-0.16 – 0.14) | 1 | -0.01 (-0.17 – 0.16) | 1 |
| **COx-a_R (au)** | 0.05 (-0.12 – 0.25) |  | 0 (-0.14 – 0.17) |  | 0.08 (-0.1 – 0.28) |  | 0 (-0.14 – 0.19) |  | 0.01 (-0.13 – 0.17) |  | 0 (-0.16 – 0.17) |  |
| **MAD of ABP (mmHg)** | 7.59 (6.59 – 8.59) | – | 8.73 (7.82 – 11.48) | – | 5.59 (5.59 – 5.59) | – | 6.9 (6.9 – 6.9) | – | 8.73 (8.73 – 8.73) | – | 11.91 (10.75 – 13.07) | – |
| **MAD of CPP (mmHg)** | 7.39 (6.49 – 8.28) |  | 10.78 (9.95 – 11.46) |  | 5.6 (5.6 – 5.6) |  | 10.78 (10.78 – 10.78) |  | 9.12 (9.12 – 9.12) |  | 10.66 (9.91 – 11.4) |  |
| **MAD of rSO2_L (%)** | 6.1 (5.68 – 6.52) | 0.6667 | 2.8 (1.4 – 4.95) | 0.4 | 5.25 (5.25 – 5.25) | 1 | 0 (0 – 0) | 1 | 7.09 (7.09 – 7.09) | 1 | 4.87 (3.84 – 5.91) | 1 |
| **MAD of rSO2_R (%)** | 7.18 (7.01 – 7.35) |  | 4.51 (4.29 – 6.47) |  | 7.52 (7.52 – 7.52) |  | 4.06 (4.06 – 4.06) |  | 8.43 (8.43 – 8.43) |  | 5.68 (5.1 – 6.26) |  |
| **MAD of COx_L (au)** | 0.26 (0.26 – 0.27) | 0.6667 | 0.24 (0.24 – 0.25) | 1 | 0.26 (0.26 – 0.26) | 1 | 0.26 (0.26 – 0.26) | 1 | 0.24 (0.24 – 0.24) | 1 | 0.25 (0.24 – 0.26) | 0.6667 |
| **MAD of COx_R (au)** | 0.27 (0.27 – 0.28) |  | 0.24 (0.23 – 0.25) |  | 0.28 (0.28 – 0.28) |  | 0.26 (0.26 – 0.26) |  | 0.24 (0.24 – 0.24) |  | 0.24 (0.23 – 0.25) |  |
| **MAD of COx-a_L (au)** | 0.26 (0.26 – 0.27) | 0.6667 | 0.22 (0.22 – 0.24) | 0.7 | 0.25 (0.25 – 0.25) | 1 | 0.26 (0.26 – 0.26) | 1 | 0.22 (0.22 – 0.22) | 1 | 0.25 (0.23 – 0.26) | 0.6667 |
| **MAD of COx-a_R (au)** | 0.28 (0.27 – 0.28) |  | 0.22 (0.22 – 0.23) |  | 0.29 (0.29 – 0.29) |  | 0.24 (0.24 – 0.24) |  | 0.22 (0.22 – 0.22) |  | 0.24 (0.23 – 0.25) |  |
| **% time rSO2_L > 60%** | 91 (88.04 – 93.96; 8.77) | 0.6667 | 72.8 (58.23 – 85.33; 37.18) | 0.2 | 96.91 (96.91 – 96.91; 0) | 1 | 43.66 (43.66 – 43.66; 0) | 1 | 97.87 (97.87 – 97.87; 0) | 1 | 78.94 (75.87 – 82.01; 9.11) | 0.3333 |
| **% time rSO2_R > 60%** | 88.67 (86.31 – 91.03; 7) |  | 99.88 (98.25 – 99.94; 0.17) |  | 83.95 (83.95 – 83.95; 0) |  | 100 (100 – 100; 0) |  | 96.62 (96.62 – 96.62; 0) |  | 96.64 (95.01 – 98.26; 4.81) |  |
| **% time rSO2_L > 70%** | 38.4 (33.84 – 42.97; 13.54) | 1 | 43.66 (21.83 – 43.93; 0.8) | 0.1 | 47.53 (47.53 – 47.53; 0) | 1 | 43.66 (43.66 – 43.66; 0) | 1 | 44.2 (44.2 – 44.2; 0) | 1 | 14.64 (7.32 – 21.95; 21.7) | 0.3333 |
| **% time rSO2_R > 70%** | 38.66 (35.69 – 41.63; 8.81) |  | 83.47 (74.96 – 91.74; 24.5) |  | 32.72 (32.72 – 32.72; 0) |  | 100 (100 – 100; 0) |  | 66.44 (66.44 – 66.44; 0) |  | 64.04 (54.32 – 73.76; 28.81) |  |
| **% time rSO2_L > 80%** | 0.21 (0.1 – 0.31; 0.31) | 1 | 7.04 (3.52 – 15.91; 10.44) | 0.2 | 0 (0 – 0; 0) | – | 7.04 (7.04 – 7.04; 0) | 1 | 24.78 (24.78 – 24.78; 0) | 1 | 0.21 (0.1 – 0.31; 0.31) | 0.3333 |
| **% time rSO2_R > 80%** | 2.66 (1.33 – 3.99; 3.94) |  | 29.15 (20.95 – 51.21; 24.32) |  | 0 (0 – 0; 0) |  | 73.26 (73.26 – 73.26; 0) |  | 29.15 (29.15 – 29.15; 0) |  | 9.03 (7.18 – 10.89; 5.51) |  |
| **% time rSO2_L > 90%** | 0 (0 – 0; 0) | – | 0 (0 – 0.41; 0) | 0.3537 | 0 (0 – 0; 0) | – | 0 (0 – 0; 0) | 1 | 0.82 (0.82 – 0.82; 0) | 1 | 0 (0 – 0; 0) | – |
| **% time rSO2_R > 90%** | 0 (0 – 0; 0) |  | 2.57 (1.29 – 8.96; 3.81) |  | 0 (0 – 0; 0) |  | 2.57 (2.57 – 2.57; 0) |  | 15.35 (15.35 – 15.35; 0) |  | 0 (0 – 0; 0) |  |
| **% time COx_L > 0** | 57.89 (49.5 – 66.28; 24.89) | 1 | 49.07 (41.11 – 51.29; 6.59) | 1 | 74.68 (74.68 – 74.68; 0) | 1 | 53.51 (53.51 – 53.51; 0) | 1 | 49.07 (49.07 – 49.07; 0) | 1 | 37.12 (35.14 – 39.11; 5.9) | 0.3333 |
| **% time COx_R > 0** | 55.4 (52.81 – 57.99; 7.68) |  | 48.8 (47.38 – 50.56; 4.21) |  | 60.58 (60.58 – 60.58; 0) |  | 48.8 (48.8 – 48.8; 0) |  | 52.32 (52.32 – 52.32; 0) |  | 48.09 (47.03 – 49.16; 3.16) |  |
| **% time COx_L > 0.2** | 31.68 (24.57 – 38.78; 21.07) | 1 | 18.74 (15.74 – 22.58; 8.91) | 1 | 45.89 (45.89 – 45.89; 0) | 1 | 26.42 (26.42 – 26.42; 0) | 1 | 18.74 (18.74 – 18.74; 0) | 1 | 15.1 (13.92 – 16.29; 3.51) | 0.6667 |
| **% time COx_R > 0.2** | 28.31 (25.54 – 31.08; 8.22) |  | 21.83 (19.12 – 22.25; 1.23) |  | 33.85 (33.85 – 33.85; 0) |  | 22.67 (22.67 – 22.67; 0) |  | 21.83 (21.83 – 21.83; 0) |  | 19.59 (18 – 21.18; 4.71) |  |
| **% time COx_L > 0.3** | 21.29 (16.24 – 26.34; 14.97) | 1 | 9.83 (8.66 – 13.61; 3.47) | 1 | 31.39 (31.39 – 31.39; 0) | 1 | 17.39 (17.39 – 17.39; 0) | 1 | 9.83 (9.83 – 9.83; 0) | 1 | 9.34 (8.42 – 10.27; 2.75) | 0.6667 |
| **% time COx_R > 0.3** | 18.04 (15.82 – 20.27; 6.6) |  | 11.04 (10.01 – 13.25; 3.04) |  | 22.49 (22.49 – 22.49; 0) |  | 15.47 (15.47 – 15.47; 0) |  | 11.04 (11.04 – 11.04; 0) |  | 11.29 (10.14 – 12.44; 3.41) |  |
| **% time COx-a_L > 0** | 61.03 (54.2 – 67.85; 20.24) | 1 | 49.06 (49.04 – 50.11; 0.06) | 1 | 74.68 (74.68 – 74.68; 0) | 1 | 51.16 (51.16 – 51.16; 0) | 1 | 49.02 (49.02 – 49.02; 0) | 1 | 48.22 (47.8 – 48.64; 1.25) | 1 |
| **% time COx-a_R > 0** | 58.16 (56.84 – 59.48; 3.91) |  | 50.4 (48.05 – 51.23; 2.47) |  | 60.8 (60.8 – 60.8; 0) |  | 50.4 (50.4 – 50.4; 0) |  | 52.06 (52.06 – 52.06; 0) |  | 50.61 (48.15 – 53.07; 7.29) |  |
| **% time COx-a_L > 0.2** | 33.33 (27.7 – 38.96; 16.7) | 1 | 19.29 (18.27 – 21.44; 3.01) | 1 | 44.59 (44.59 – 44.59; 0) | 1 | 23.59 (23.59 – 23.59; 0) | 1 | 17.26 (17.26 – 17.26; 0) | 1 | 20.68 (19.98 – 21.37; 2.06) | 1 |
| **% time COx-a_R > 0.2** | 29.44 (27.67 – 31.2; 5.23) |  | 20.35 (18.24 – 22.64; 6.25) |  | 32.96 (32.96 – 32.96; 0) |  | 24.93 (24.93 – 24.93; 0) |  | 20.35 (20.35 – 20.35; 0) |  | 21.02 (18.58 – 23.47; 7.25) |  |
| **% time COx-a_L > 0.3** | 21.51 (17.43 – 25.58; 12.08) | 1 | 8.96 (8.78 – 12.95; 0.51) | 1 | 29.65 (29.65 – 29.65; 0) | 1 | 16.94 (16.94 – 16.94; 0) | 1 | 8.96 (8.96 – 8.96; 0) | 1 | 10.99 (9.8 – 12.17; 3.52) | 1 |
| **% time COx-a_R > 0.3** | 19.48 (17.75 – 21.21; 5.13) |  | 10.53 (9.5 – 14.95; 3.04) |  | 22.94 (22.94 – 22.94; 0) |  | 19.36 (19.36 – 19.36; 0) |  | 10.53 (10.53 – 10.53; 0) |  | 12.25 (10.36 – 14.14; 5.59) |  |
| *The p-values in the table are derived using Mann-Whitney U test between the bilateral signals.*  *ABP, arterial blood pressure; au, arbitrary units; CPP, cerebral perfusion pressure; COx, cerebral oximetry index with CPP; COx-a, cerebral oximetry index with ABP; CT, computed tomography; DAI, diffuse axonal injury; EDH, epidural hematoma; MAD, median absolute deviation; IQR, interquartile range; mmHg, millimeters of mercury; rSO2, regional cerebral oxygen saturation; tSAH, traumatic subarachnoid hemorrhage; SDH, subdural hematoma; aSDH, acute subdural hematoma; TBI-BLR, traumatic brain injury patient group with bifrontal lobe pathology.* | | | | | | | | | | | | |

File S8m: Sub-grouped Absolute Regional Hemispheric Disparity Analysis using 10-Second, 1-Minute, and 5-Minute Data Resolutions for HC Population

| **Physiologic Variable** | **Median (IQR)** | | | | | | |
| --- | --- | --- | --- | --- | --- | --- | --- |
|  | **Age < 40 [n = 95]** | **Age 40 – 60 [n = 6]** | **Age > 60 [n = 1]** | **Males [n = 42]** | **Females [n = 60]** | **Left Hand Dominance [n = 9]** | **Right Hand Dominance [n = 93]** |
| **Sub-grouped Physiologic Results for 10-Second Data Resolution** | | | | | | | |
| **ARHD of rSO2 (%)** | 3.83 (2.9 – 4.73) | 5.82 (4.6 – 6.58) | 0.89 (0.43 – 1.6) | 3.2 (2.33 – 3.95) | 4.91 (3.91 – 5.62) | 4.6 (4.1 – 5.28) | 3.46 (2.73 – 4.4) |
| **ARHD of COx (au)** | – | – | – | – | – | – | – |
| **ARHD of COx-a (au)** | 0.14 (0.07 – 0.27) | 0.17 (0.08 – 0.29) | 0.09 (0.04 – 0.15) | 0.14 (0.07 – 0.24) | 0.14 (0.08 – 0.27) | 0.14 (0.07 – 0.29) | 0.14 (0.07 – 0.25) |
| **MAD of ARHD rSO2 (%)** | 1.05 (0.78 – 1.47) | 1.41 (1.05 – 1.48) | 0.86 (0.86 – 0.86) | 1.02 (0.69 – 1.4) | 1.11 (0.86 – 1.48) | 1.19 (0.8 – 1.88) | 1.05 (0.79 – 1.47) |
| **MAD of ARHD COx (au)** | – | – | – | – | – | – | – |
| **MAD of ARHD COx-a (au)** | 0.13 (0.1 – 0.15) | 0.14 (0.12 – 0.16) | 0.08 (0.08 – 0.08) | 0.12 (0.1 – 0.15) | 0.13 (0.1 – 0.16) | 0.13 (0.1 – 0.16) | 0.13 (0.1 – 0.15) |
| **Sub-grouped Physiologic Results for 1-Minute Data Resolution** | | | | | | | |
| **ARHD of rSO2 (%)** | 3.88 (2.9 – 4.59) | 5.78 (4.51 – 6.26) | 0.84 (0.35 – 1.51) | 3.29 (2.35 – 4.01) | 4.77 (3.88 – 5.51) | 4.62 (4.26 – 5.14) | 3.5 (2.69 – 4.22) |
| **ARHD of COx (au)** | – | – | – | – | – | – | – |
| **ARHD of COx-a (au)** | 0.14 (0.07 – 0.25) | 0.16 (0.08 – 0.26) | 0.09 (0.03 – 0.14) | 0.13 (0.07 – 0.22) | 0.14 (0.07 – 0.26) | 0.14 (0.07 – 0.26) | 0.14 (0.07 – 0.25) |
| **MAD of ARHD rSO2 (%)** | 0.89 (0.59 – 1.35) | 1.33 (1.05 – 1.42) | 0.8 (0.8 – 0.8) | 0.84 (0.58 – 1.28) | 0.98 (0.69 – 1.37) | 0.9 (0.63 – 1.95) | 0.92 (0.59 – 1.33) |
| **MAD of ARHD COx (au)** | – | – | – | – | – | – | – |
| **MAD of ARHD COx-a (au)** | 0.12 (0.09 – 0.16) | 0.12 (0.12 – 0.16) | 0.09 (0.09 – 0.09) | 0.11 (0.09 – 0.14) | 0.13 (0.09 – 0.16) | 0.13 (0.1 – 0.16) | 0.12 (0.09 – 0.16) |
| **Sub-grouped Physiologic Results for 5-Minute Data Resolution** | | | | | | | |
| **ARHD of rSO2 (%)** | 3.8 (3.27 – 4.36) | 5.59 (5.03 – 6.19) | 0.76 (0.54 – 1.36) | 3.34 (2.85 – 3.66) | 4.74 (4.14 – 4.94) | 4.76 (4.51 – 5.17) | 3.36 (3.01 – 4) |
| **ARHD of COx (au)** | – | – | – | – | – | – | – |
| **ARHD of COx-a (au)** | 0.13 (0.08 – 0.2) | 0.17 (0.09 – 0.2) | 0.06 (0.06 – 0.12) | 0.14 (0.07 – 0.19) | 0.13 (0.09 – 0.21) | 0.13 (0.09 – 0.16) | 0.13 (0.08 – 0.2) |
| **MAD of ARHD rSO2 (%)** | 0.54 (0.36 – 0.95) | 0.9 (0.68 – 1.17) | 0.57 (0.57 – 0.57) | 0.5 (0.37 – 0.76) | 0.67 (0.39 – 1.03) | 0.56 (0.28 – 1.42) | 0.57 (0.38 – 0.94) |
| **MAD of ARHD COx (au)** | – | – | – | – | – | – | – |
| **MAD of ARHD COx-a (au)** | 0.09 (0.05 – 0.14) | 0.1 (0.04 – 0.13) | 0.05 (0.05 – 0.05) | 0.09 (0.06 – 0.14) | 0.09 (0.05 – 0.14) | 0.08 (0.05 – 0.11) | 0.09 (0.05 – 0.14) |
| *ARHD, Absolute Regional Hemispheric Disparity; au, arbitrary units; COx, cerebral oximetry index with CPP; COx-a, cerebral oximetry index with ABP; HC, healthy control volunteer group; IQR, interquartile range; MAD, median absolute deviation; rSO2, regional cerebral oxygen saturation.* | | | | | | | |

File S8n: Sub-grouped Absolute Regional Hemispheric Disparity Analysis using 10-Second, 1-Minute, and 5-Minute Data Resolutions for SP Population

| **Physiologic Variable** | **Median (IQR)** | | | | | | | | | | | | | | | | | | |
| --- | --- | --- | --- | --- | --- | --- | --- | --- | --- | --- | --- | --- | --- | --- | --- | --- | --- | --- | --- |
|  | **Age < 40 [n = 1]** | **Age 40 – 60 [n = 17]** | **Age > 60 [n = 9]** | **Males [n = 22]** | **Females [n = 5]** | **ACDF [n = 6]** | **PCDF [n = 25]** | **ACDF & PCDF [n = 3]** | **Cervical Incision and Drain [n = 1]** | **Corpectomy [n = 1]** | **Laminectomy [n = 1]** | **Thoracic Decompression & Instrumental Fusion [n = 1]** | **Propofol + Sufentanil [n = 6]** | **Ketamine + Propofol + Sufentanil [n = 6]** | **Midazolam + Propofol + Remi-Fentanyl [n = 1]** | **Midazolam + Propofol + Sufentanil [n = 4]** | **Propofol + Remi-Fentanyl + Sufentanil [n = 2]** | **Ketamine + Midazolam + Propofol + Sufentanil [n = 7]** | **Ketamine + Midazolam + Propofol + Remi-Fentanyl + Sufentanil [n = 1]** |
| **Sub-grouped Physiologic Results for 10-Second Data Resolution** | | | | | | | | | | | | | | | | | | | |
| **ARHD of rSO2 (%)** | 6.4 (5.99 – 8.26) | 3.3 (2.5 – 5) | 4.8 (3.41 – 7.4) | 4.26 (3.01 – 5.69) | 4.6 (4 – 5.15) | 3.4 (2.78 – 4.93) | 4.26 (3.51 – 6.5) | 4.41 (3 – 9) | 4.8 (3.41 – 5.58) | 7.01 (6.5 – 7.99) | 2 (1.99 – 2.99) | 3 (1.91 – 4.24) | 2.66 (1.93 – 3.62) | 5.3 (3.89 – 8.61) | 2.2 (1.55 – 4.72) | 5.71 (5.25 – 8.5) | 3.66 (2.74 – 5.4) | 6.4 (5.99 – 8.26) | 5.8 (4 – 7.4) |
| **ARHD of COx (au)** | – | – | – | – | – | – | – | – | – | – | – | – | – | – | – | – | – | – | – |
| **ARHD of COx-a (au)** | 0.17 (0.07 – 0.35) | 0.15 (0.06 – 0.31) | 0.15 (0.06 – 0.31) | 0.15 (0.06 – 0.3) | 0.21 (0.09 – 0.4) | 0.14 (0.06 – 0.29) | 0.16 (0.06 – 0.32) | 0.19 (0.08 – 0.35) | 0.15 (0.07 – 0.26) | 0.1 (0.04 – 0.21) | 0.2 (0.07 – 0.36) | 0.15 (0.06 – 0.31) | 0.15 (0.06 – 0.31) | 0.14 (0.06 – 0.29) | 0.12 (0.05 – 0.23) | 0.11 (0.04 – 0.24) | 0.17 (0.07 – 0.34) | 0.19 (0.07 – 0.36) | **0.25 (0.09 – 0.56)** |
| **MAD of ARHD rSO2 (%)** | 1.35 (1.35 – 1.35) | 1.48 (0.89 – 1.73) | 1.48 (1.02 – 2.91) | 1.48 (1.02 – 2.1) | 1.65 (1.17 – 1.7) | 1.12 (0.83 – 1.45) | 1.68 (1.02 – 2.75) | 1.53 (1.29 – 2.55) | 1.48 (1.48 – 1.48) | 1.17 (1.17 – 1.17) | 1.19 (1.19 – 1.19) | 1.73 (1.73 – 1.73) | 0.96 (0.83 – 1.37) | 1.04 (0.9 – 2.45) | 1.78 (1.78 – 1.78) | 1.33 (1.07 – 2.01) | 1.93 (1.79 – 2.07) | 1.53 (1.42 – 2.24) | 2.67 (2.67 – 2.67) |
| **MAD of ARHD COx (au)** | – | – | – | – | – | – | – | – | – | – | – | – | – | – | – | – | – | – | – |
| **MAD of ARHD COx-a (au)** | 0.18 (0.18 – 0.18) | 0.16 (0.12 – 0.2) | 0.16 (0.13 – 0.2) | 0.15 (0.12 – 0.19) | 0.2 (0.2 – 0.21) | 0.15 (0.12 – 0.18) | 0.17 (0.12 – 0.2) | 0.19 (0.17 – 0.19) | 0.13 (0.13 – 0.13) | 0.11 (0.11 – 0.11) | 0.21 (0.21 – 0.21) | 0.15 (0.15 – 0.15) | 0.15 (0.14 – 0.16) | 0.14 (0.1 – 0.16) | 0.12 (0.12 – 0.12) | 0.12 (0.11 – 0.15) | 0.18 (0.18 – 0.19) | 0.2 (0.19 – 0.2) | **0.28 (0.28 – 0.28)** |
| **Sub-grouped Physiologic Results for 1-Minute Data Resolution** | | | | | | | | | | | | | | | | | | | |
| **ARHD of rSO2 (%)** | 6.35 (5.98 – 8.35) | 3.36 (2.44 – 4.96) | 4.8 (3.38 – 7.36) | 4.3 (3.01 – 5.69) | 4.59 (3.99 – 5.08) | 3.37 (2.78 – 4.89) | 4.3 (3.5 – 6.5) | 4.4 (3.01 – 8.95) | 4.8 (3.38 – 5.53) | 7.11 (6.6 – 7.77) | 2.15 (1.88 – 2.94) | 3.08 (1.83 – 4.33) | 2.69 (1.76 – 3.68) | 5.33 (3.89 – 8.73) | 2.16 (1.58 – 4.7) | 5.76 (5.35 – 8.36) | 3.79 (2.65 – 5.41) | 6.35 (5.84 – 8.35) | 5.68 (4 – 7.36) |
| **ARHD of COx (au)** | – | – | – | – | – | – | – | – | – | – | – | – | – | – | – | – | – | – | – |
| **ARHD of COx-a (au)** | 0.13 (0.06 – 0.27) | 0.15 (0.06 – 0.3) | 0.15 (0.06 – 0.3) | 0.14 (0.06 – 0.29) | 0.22 (0.09 – 0.36) | 0.13 (0.05 – 0.26) | 0.16 (0.06 – 0.31) | 0.17 (0.09 – 0.33) | 0.15 (0.07 – 0.25) | 0.1 (0.04 – 0.2) | 0.15 (0.06 – 0.3) | 0.15 (0.07 – 0.32) | 0.15 (0.06 – 0.3) | 0.14 (0.06 – 0.27) | 0.12 (0.05 – 0.22) | 0.11 (0.04 – 0.23) | 0.18 (0.07 – 0.33) | 0.17 (0.06 – 0.33) | **0.26 (0.09 – 0.53)** |
| **MAD of ARHD rSO2 (%)** | 1.32 (1.32 – 1.32) | 1.33 (0.82 – 1.76) | 1.58 (1.03 – 2.57) | 1.38 (0.84 – 2.19) | 1.54 (0.95 – 1.76) | 1.09 (0.77 – 1.33) | 1.67 (1.01 – 2.48) | 1.43 (1.17 – 2.4) | 1.57 (1.57 – 1.57) | 0.95 (0.95 – 0.95) | 0.82 (0.82 – 0.82) | 1.86 (1.86 – 1.86) | 0.95 (0.77 – 1.43) | 0.96 (0.8 – 2.18) | 1.72 (1.72 – 1.72) | 1.14 (0.9 – 1.84) | 1.92 (1.73 – 2.11) | 1.58 (1.37 – 2.08) | 2.5 (2.5 – 2.5) |
| **MAD of ARHD COx (au)** | – | – | – | – | – | – | – | – | – | – | – | – | – | – | – | – | – | – | – |
| **MAD of ARHD COx-a (au)** | 0.14 (0.14 – 0.14) | 0.16 (0.12 – 0.18) | 0.15 (0.14 – 0.17) | 0.14 (0.12 – 0.17) | 0.2 (0.18 – 0.2) | 0.13 (0.12 – 0.15) | 0.16 (0.12 – 0.22) | 0.17 (0.15 – 0.17) | 0.14 (0.14 – 0.14) | 0.1 (0.1 – 0.1) | 0.16 (0.16 – 0.16) | 0.15 (0.15 – 0.15) | 0.15 (0.14 – 0.15) | 0.14 (0.1 – 0.15) | 0.12 (0.12 – 0.12) | 0.11 (0.11 – 0.13) | 0.18 (0.17 – 0.18) | 0.17 (0.15 – 0.21) | **0.29 (0.29 – 0.29)** |
| **Sub-grouped Physiologic Results for 5-Minute Data Resolution** | | | | | | | | | | | | | | | | | | | |
| **ARHD of rSO2 (%)** | 6.19 (6.02 – 8.24) | 3.38 (2.58 – 4.87) | 4.87 (3.38 – 7.88) | 4.31 (2.99 – 5.57) | 4.46 (3.8 – 4.98) | 3.32 (2.71 – 4.63) | 4.35 (3.44 – 6.31) | 4.26 (3.08 – 8.71) | 4.87 (3.38 – 5.51) | 7.13 (6.55 – 7.63) | 2.16 (1.64 – 2.74) | 2.99 (2.14 – 4.51) | 2.75 (2.05 – 3.76) | 5.17 (3.56 – 8.11) | 2.17 (1.62 – 4.27) | 5.74 (5.34 – 8.17) | 3.87 (2.69 – 5.25) | 6.19 (5.62 – 8.24) | 5.64 (3.99 – 7.88) |
| **ARHD of COx (au)** | – | – | – | – | – | – | – | – | – | – | – | – | – | – | – | – | – | – | – |
| **ARHD of COx-a (au)** | 0.14 (0.05 – 0.24) | 0.15 (0.07 – 0.26) | 0.16 (0.07 – 0.27) | 0.13 (0.07 – 0.24) | 0.19 (0.1 – 0.27) | 0.11 (0.06 – 0.25) | 0.18 (0.06 – 0.29) | 0.16 (0.07 – 0.24) | 0.16 (0.07 – 0.21) | 0.08 (0.03 – 0.22) | 0.15 (0.06 – 0.24) | 0.16 (0.09 – 0.31) | 0.16 (0.07 – 0.27) | 0.13 (0.06 – 0.23) | 0.11 (0.07 – 0.17) | 0.1 (0.04 – 0.24) | 0.18 (0.08 – 0.31) | 0.16 (0.07 – 0.24) | **0.34 (0.12 – 0.45)** |
| **MAD of ARHD rSO2 (%)** | 1.43 (1.43 – 1.43) | 0.98 (0.81 – 1.66) | 1.74 (0.87 – 2.78) | 1.32 (0.81 – 1.97) | 1.44 (0.81 – 1.69) | 0.89 (0.68 – 1.32) | 1.72 (0.84 – 2.25) | 1.34 (1.08 – 2.06) | 1.31 (1.31 – 1.31) | 0.81 (0.81 – 0.81) | 0.81 (0.81 – 0.81) | 1.66 (1.66 – 1.66) | 0.81 (0.68 – 1.19) | 0.85 (0.82 – 1.93) | 1.52 (1.52 – 1.52) | 0.9 (0.81 – 1.43) | 1.74 (1.59 – 1.9) | 1.69 (1.38 – 1.96) | 3 (3 – 3) |
| **MAD of ARHD COx (au)** | – | – | – | – | – | – | – | – | – | – | – | – | – | – | – | – | – | – | – |
| **MAD of ARHD COx-a (au)** | 0.14 (0.14 – 0.14) | 0.13 (0.09 – 0.14) | 0.13 (0.11 – 0.21) | 0.13 (0.09 – 0.16) | 0.14 (0.11 – 0.15) | 0.12 (0.09 – 0.14) | 0.14 (0.09 – 0.21) | 0.13 (0.12 – 0.15) | 0.12 (0.12 – 0.12) | 0.09 (0.09 – 0.09) | 0.13 (0.13 – 0.13) | 0.13 (0.13 – 0.13) | 0.13 (0.12 – 0.14) | 0.11 (0.09 – 0.13) | 0.09 (0.09 – 0.09) | 0.11 (0.09 – 0.14) | 0.17 (0.14 – 0.19) | 0.14 (0.13 – 0.17) | **0.31 (0.31 – 0.31)** |
| *ACDF, anterior cervical discectomy and fusion; ARHD, Absolute Regional Hemispheric Disparity; au, arbitrary units; COx-a, cerebral oximetry index with arterial blood pressure; IQR, interquartile range; MAD, median absolute deviation; PCDF, posterior cervical discectomy and fusion; rSO2, regional cerebral oxygen saturation; SP, elective spinal surgery patient group.* | | | | | | | | | | | | | | | | | | | |

File S8o: Sub-grouped Absolute Regional Hemispheric Disparity Analysis using 10-Second, 1-Minute, and 5-Minute Data Resolutions for TBI-GLR Population

| **Physiologic Variable** | **Median (IQR)** | | | | | | | | |
| --- | --- | --- | --- | --- | --- | --- | --- | --- | --- |
|  | **Age < 40 [n = 28]** | **Age 40 – 60 [n = 23]** | **Age > 60 [n = 13]** | **Males [n = 51]** | **Females [n = 13]** | **Focal Injury (aSDH, SDH, EDH, or Contusion) [n = 42]** | **Diffuse Injury (DAI or tSAH) [n = 22]** | **Marshall CT V [n = 29]** | **Marshall CT IV [n = 8]** |
| **Sub-grouped Physiologic Results for 10-Second Data Resolution** | | | | | | | | | |
| **ARHD of rSO2 (%)** | 4.03 (2.03 – 8) | 6 (3.27 – 7.73) | 6.51 (3.01 – 9.36) | 5 (2.45 – 8) | 6.61 (3.99 – 10.99) | 6.49 (3.14 – 9.42) | 3.54 (1.84 – 5.49) | 7.22 (3.52 – 9.5) | 5.99 (3.67 – 8.01) |
| **ARHD of COx (au)** | 0.16 (0.07 – 0.32) | 0.15 (0.06 – 0.28) | 0.19 (0.09 – 0.35) | 0.17 (0.07 – 0.32) | 0.16 (0.06 – 0.29) | 0.18 (0.08 – 0.33) | 0.14 (0.06 – 0.27) | 0.18 (0.08 – 0.33) | 0.2 (0.08 – 0.37) |
| **ARHD of COx-a (au)** | 0.16 (0.07 – 0.32) | 0.15 (0.06 – 0.28) | 0.18 (0.08 – 0.33) | 0.17 (0.07 – 0.32) | 0.15 (0.06 – 0.28) | 0.17 (0.08 – 0.32) | 0.14 (0.06 – 0.27) | 0.17 (0.08 – 0.33) | 0.17 (0.07 – 0.32) |
| **MAD of ARHD rSO2 (%)** | 3.15 (2.28 – 4.77) | 2.27 (1.88 – 4.17) | 2.85 (1.91 – 3.83) | 2.82 (2.04 – 4.07) | 4.43 (2.36 – 5) | 3.17 (2.21 – 4.45) | 2.34 (1.75 – 4.29) | 3.26 (2.22 – 4.46) | 2.92 (2.22 – 3.75) |
| **MAD of ARHD COx (au)** | 0.17 (0.13 – 0.18) | 0.15 (0.13 – 0.18) | 0.18 (0.17 – 0.2) | 0.16 (0.14 – 0.18) | 0.15 (0.11 – 0.19) | 0.17 (0.15 – 0.19) | 0.14 (0.12 – 0.17) | 0.18 (0.15 – 0.19) | 0.19 (0.16 – 0.21) |
| **MAD of ARHD COx-a (au)** | 0.16 (0.13 – 0.17) | 0.15 (0.13 – 0.18) | 0.18 (0.16 – 0.18) | 0.16 (0.14 – 0.18) | 0.15 (0.11 – 0.18) | 0.17 (0.15 – 0.18) | 0.14 (0.12 – 0.16) | 0.18 (0.15 – 0.18) | 0.17 (0.15 – 0.18) |
| **Sub-grouped Physiologic Results for 1-Minute Data Resolution** | | | | | | | | | |
| **ARHD of rSO2 (%)** | 4.12 (2.03 – 8) | 5.86 (3.32 – 7.76) | 6.59 (3.16 – 9.3) | 5.08 (2.49 – 8) | 6.58 (3.83 – 10.93) | 6.48 (3.24 – 9.34) | 3.56 (1.79 – 5.39) | 7.16 (3.5 – 9.41) | 5.89 (3.65 – 8.04) |
| **ARHD of COx (au)** | 0.16 (0.07 – 0.31) | 0.14 (0.06 – 0.27) | 0.19 (0.08 – 0.34) | 0.16 (0.07 – 0.3) | 0.16 (0.07 – 0.28) | 0.17 (0.08 – 0.32) | 0.14 (0.06 – 0.27) | 0.18 (0.08 – 0.32) | 0.18 (0.08 – 0.33) |
| **ARHD of COx-a (au)** | 0.16 (0.07 – 0.31) | 0.15 (0.07 – 0.27) | 0.19 (0.08 – 0.34) | 0.16 (0.07 – 0.31) | 0.15 (0.07 – 0.27) | 0.17 (0.08 – 0.32) | 0.14 (0.06 – 0.26) | 0.17 (0.08 – 0.33) | 0.17 (0.07 – 0.31) |
| **MAD of ARHD rSO2 (%)** | 3.29 (2.23 – 4.76) | 2.26 (1.87 – 4.1) | 2.77 (1.98 – 3.89) | 2.74 (1.91 – 4.07) | 4.27 (2.56 – 4.87) | 3.22 (2.11 – 4.37) | 2.38 (1.79 – 4.39) | 3.26 (2.13 – 4.41) | 3.05 (2.22 – 3.91) |
| **MAD of ARHD COx (au)** | 0.15 (0.12 – 0.17) | 0.14 (0.12 – 0.18) | 0.18 (0.15 – 0.2) | 0.15 (0.13 – 0.18) | 0.15 (0.11 – 0.18) | 0.17 (0.14 – 0.18) | 0.14 (0.11 – 0.16) | 0.17 (0.14 – 0.19) | 0.17 (0.15 – 0.2) |
| **MAD of ARHD COx-a (au)** | 0.15 (0.12 – 0.17) | 0.14 (0.13 – 0.17) | 0.18 (0.15 – 0.18) | 0.15 (0.13 – 0.17) | 0.14 (0.11 – 0.17) | 0.17 (0.14 – 0.18) | 0.14 (0.12 – 0.16) | 0.17 (0.14 – 0.18) | 0.16 (0.14 – 0.18) |
| **Sub-grouped Physiologic Results for 5-Minute Data Resolution** | | | | | | | | | |
| **ARHD of rSO2 (%)** | 4.08 (2.1 – 8.02) | 5.87 (3.31 – 7.53) | 6.54 (3.21 – 9.24) | 5.07 (2.45 – 8.04) | 6.6 (3.82 – 10.91) | 6.49 (3.28 – 9.31) | 3.52 (1.78 – 5.37) | 7.05 (3.59 – 9.39) | 5.88 (3.63 – 8.08) |
| **ARHD of COx (au)** | 0.14 (0.06 – 0.26) | 0.12 (0.06 – 0.23) | 0.17 (0.08 – 0.3) | 0.15 (0.06 – 0.26) | 0.14 (0.07 – 0.25) | 0.15 (0.07 – 0.28) | 0.12 (0.05 – 0.23) | 0.16 (0.07 – 0.28) | 0.18 (0.08 – 0.31) |
| **ARHD of COx-a (au)** | 0.14 (0.06 – 0.26) | 0.13 (0.06 – 0.23) | 0.17 (0.07 – 0.29) | 0.14 (0.06 – 0.26) | 0.13 (0.06 – 0.24) | 0.15 (0.07 – 0.27) | 0.12 (0.06 – 0.23) | 0.16 (0.07 – 0.28) | 0.15 (0.06 – 0.27) |
| **MAD of ARHD rSO2 (%)** | 3.26 (2.19 – 4.65) | 2.19 (1.88 – 4.04) | 2.8 (1.99 – 3.93) | 2.8 (1.95 – 4.09) | 4.13 (2.54 – 4.77) | 3.15 (2.02 – 4.32) | 2.34 (1.86 – 4.41) | 3.19 (1.99 – 4.36) | 3.07 (2.17 – 3.95) |
| **MAD of ARHD COx (au)** | 0.13 (0.09 – 0.15) | 0.12 (0.11 – 0.15) | 0.14 (0.13 – 0.16) | 0.13 (0.11 – 0.15) | 0.12 (0.09 – 0.16) | 0.14 (0.12 – 0.16) | 0.12 (0.09 – 0.14) | 0.14 (0.12 – 0.16) | 0.15 (0.11 – 0.18) |
| **MAD of ARHD COx-a (au)** | 0.13 (0.1 – 0.14) | 0.12 (0.1 – 0.14) | 0.15 (0.14 – 0.16) | 0.13 (0.11 – 0.15) | 0.12 (0.09 – 0.15) | 0.14 (0.12 – 0.15) | 0.12 (0.1 – 0.14) | 0.14 (0.12 – 0.16) | 0.14 (0.12 – 0.15) |
| *ARHD, Absolute Regional Hemispheric Disparity; au, arbitrary units; COx, cerebral oximetry index with cerebral perfusion pressure; COx-a, cerebral oximetry index with arterial blood pressure; CT, computed tomography; DAI, diffuse axonal injury; EDH, epidural hematoma; IQR, interquartile range; MAD, median absolute deviation; rSO2, regional cerebral oxygen saturation; tSAH, traumatic subarachnoid hemorrhage; SDH, subdural hematoma; aSDH, acute subdural hematoma; TBI-GLR, traumatic brain injury patient group without bifrontal lobe pathology.* | | | | | | | | | |

| **Physiologic Variable** | **Median (IQR)** | | | | | | | |
| --- | --- | --- | --- | --- | --- | --- | --- | --- |
|  | **Marshall CT III [n = 25]** | **Marshall CT II [n = 2]** | **Rotterdam CT 6 [n = 14]** | **Rotterdam CT 5 [n = 16]** | **Rotterdam CT 4 [n = 17]** | **Rotterdam CT 3 [n = 13]** | **Rotterdam CT 2 [n = 3]** | **Rotterdam CT 1 [n = 1]** |
| **Sub-grouped Physiologic Results for 10-Second Data Resolution** | | | | | | | | |
| **ARHD of rSO2 (%)** | 3.5 (1.99 – 5.23) | 4.5 (2.26 – 6.61) | 6.25 (3.51 – 7.5) | 7.36 (4.25 – 11.4) | 6 (3.27 – 8.52) | 3.5 (1.69 – 5) | 6.61 (3.99 – 10.99) | 2 (1 – 4) |
| **ARHD of COx (au)** | 0.14 (0.06 – 0.28) | 0.14 (0.06 – 0.28) | 0.17 (0.08 – 0.33) | 0.19 (0.08 – 0.35) | 0.16 (0.07 – 0.29) | 0.14 (0.05 – 0.26) | 0.16 (0.07 – 0.29) | 0.14 (0.06 – 0.28) |
| **ARHD of COx-a (au)** | 0.15 (0.06 – 0.27) | 0.14 (0.06 – 0.28) | 0.17 (0.08 – 0.32) | 0.18 (0.08 – 0.34) | 0.16 (0.07 – 0.28) | 0.14 (0.06 – 0.26) | 0.15 (0.07 – 0.28) | 0.14 (0.06 – 0.26) |
| **MAD of ARHD rSO2 (%)** | 2.3 (1.73 – 4.45) | 3.11 (2.71 – 3.51) | 2.8 (2.13 – 4.14) | 3.77 (2.99 – 5.39) | 2.85 (1.48 – 4.45) | 2.25 (1.97 – 2.36) | 5.03 (4.47 – 5.06) | 1.57 (1.57 – 1.57) |
| **MAD of ARHD COx (au)** | 0.14 (0.12 – 0.17) | 0.14 (0.13 – 0.15) | 0.18 (0.15 – 0.19) | 0.18 (0.17 – 0.21) | 0.15 (0.13 – 0.18) | 0.14 (0.11 – 0.16) | 0.15 (0.14 – 0.16) | 0.14 (0.14 – 0.14) |
| **MAD of ARHD COx-a (au)** | 0.15 (0.12 – 0.16) | 0.14 (0.13 – 0.15) | 0.17 (0.15 – 0.19) | 0.18 (0.17 – 0.19) | 0.15 (0.13 – 0.17) | 0.14 (0.11 – 0.16) | 0.15 (0.13 – 0.16) | 0.14 (0.14 – 0.14) |
| **Sub-grouped Physiologic Results for 1-Minute Data Resolution** | | | | | | | | |
| **ARHD of rSO2 (%)** | 3.48 (1.89 – 5.26) | 4.54 (2.33 – 6.54) | 6.26 (3.51 – 7.6) | 7.34 (4.26 – 11.39) | 5.86 (3.32 – 8.59) | 3.48 (1.68 – 4.98) | 6.58 (3.83 – 10.93) | 2.05 (0.94 – 3.92) |
| **ARHD of COx (au)** | 0.14 (0.06 – 0.27) | 0.14 (0.06 – 0.27) | 0.18 (0.08 – 0.33) | 0.18 (0.08 – 0.33) | 0.15 (0.07 – 0.3) | 0.13 (0.06 – 0.25) | 0.16 (0.07 – 0.28) | 0.14 (0.06 – 0.27) |
| **ARHD of COx-a (au)** | 0.14 (0.06 – 0.26) | 0.14 (0.06 – 0.27) | 0.18 (0.08 – 0.33) | 0.18 (0.08 – 0.33) | 0.15 (0.07 – 0.28) | 0.14 (0.06 – 0.26) | 0.15 (0.07 – 0.27) | 0.13 (0.06 – 0.25) |
| **MAD of ARHD rSO2 (%)** | 2.31 (1.77 – 4.56) | 3.09 (2.68 – 3.51) | 2.72 (1.98 – 4.07) | 3.94 (3.16 – 5.42) | 2.77 (1.49 – 4.56) | 2.23 (1.85 – 2.56) | 4.87 (4.4 – 4.96) | 1.89 (1.89 – 1.89) |
| **MAD of ARHD COx (au)** | 0.14 (0.11 – 0.15) | 0.13 (0.12 – 0.15) | 0.17 (0.14 – 0.21) | 0.17 (0.15 – 0.2) | 0.14 (0.13 – 0.18) | 0.13 (0.11 – 0.15) | 0.15 (0.13 – 0.15) | 0.14 (0.14 – 0.14) |
| **MAD of ARHD COx-a (au)** | 0.14 (0.12 – 0.16) | 0.14 (0.13 – 0.15) | 0.17 (0.14 – 0.19) | 0.17 (0.16 – 0.18) | 0.14 (0.13 – 0.16) | 0.14 (0.11 – 0.14) | 0.14 (0.13 – 0.15) | 0.13 (0.13 – 0.13) |
| **Sub-grouped Physiologic Results for 5-Minute Data Resolution** | | | | | | | | |
| **ARHD of rSO2 (%)** | 3.45 (1.84 – 5.21) | 4.53 (2.39 – 6.4) | 6.23 (3.51 – 7.57) | 7.25 (4.29 – 11.37) | 5.87 (3.31 – 8.62) | 3.45 (1.69 – 4.97) | 6.6 (3.82 – 10.91) | 2.05 (0.93 – 3.99) |
| **ARHD of COx (au)** | 0.12 (0.05 – 0.24) | 0.12 (0.05 – 0.23) | 0.16 (0.07 – 0.29) | 0.15 (0.07 – 0.28) | 0.14 (0.07 – 0.26) | 0.12 (0.05 – 0.22) | 0.14 (0.06 – 0.24) | 0.12 (0.05 – 0.23) |
| **ARHD of COx-a (au)** | 0.12 (0.05 – 0.23) | 0.12 (0.05 – 0.23) | 0.16 (0.08 – 0.28) | 0.16 (0.07 – 0.28) | 0.13 (0.06 – 0.24) | 0.12 (0.05 – 0.22) | 0.13 (0.06 – 0.24) | 0.11 (0.05 – 0.22) |
| **MAD of ARHD rSO2 (%)** | 2.24 (1.84 – 4.61) | 2.99 (2.56 – 3.43) | 2.76 (1.98 – 3.98) | 3.97 (3.1 – 5.48) | 2.8 (1.75 – 4.61) | 2.13 (1.84 – 2.54) | 4.84 (4.35 – 4.87) | 1.93 (1.93 – 1.93) |
| **MAD of ARHD COx (au)** | 0.12 (0.09 – 0.13) | 0.11 (0.1 – 0.13) | 0.14 (0.12 – 0.18) | 0.15 (0.14 – 0.18) | 0.12 (0.11 – 0.15) | 0.11 (0.09 – 0.13) | 0.12 (0.1 – 0.13) | 0.12 (0.12 – 0.12) |
| **MAD of ARHD COx-a (au)** | 0.12 (0.1 – 0.14) | 0.12 (0.11 – 0.13) | 0.15 (0.12 – 0.17) | 0.14 (0.14 – 0.15) | 0.12 (0.11 – 0.14) | 0.12 (0.09 – 0.12) | 0.12 (0.11 – 0.13) | 0.11 (0.11 – 0.11) |
| *ARHD, Absolute Regional Hemispheric Disparity; au, arbitrary units; COx, cerebral oximetry index with cerebral perfusion pressure; COx-a, cerebral oximetry index with arterial blood pressure; CT, computed tomography; DAI, diffuse axonal injury; EDH, epidural hematoma; IQR, interquartile range; MAD, median absolute deviation; rSO2, regional cerebral oxygen saturation; tSAH, traumatic subarachnoid hemorrhage; SDH, subdural hematoma; aSDH, acute subdural hematoma; TBI-GLR, traumatic brain injury patient group without bifrontal lobe pathology.* | | | | | | | | |

| **Physiologic Variable** | **Median (IQR)** | | | | | | | |
| --- | --- | --- | --- | --- | --- | --- | --- | --- |
|  | **No Anesthetic [n = 2]** | **Propofol [n = 14]** | **Fentanyl + Propofol [n = 22]** | **Ketamine + Propofol [n = 2]** | **Midazolam + Propofol [n = 1]** | **Fentanyl + Ketamine + Propofol [n = 5]** | **Fentanyl + Midazolam + Propofol [n = 13]** | **Fentanyl + Ketamine + Midazolam + Propofol [n = 5]** |
| **Sub-grouped Physiologic Results for 10-Second Data Resolution** | | | | | | | | |
| **ARHD of rSO2 (%)** | 7.53 (6.02 – 13.01) | 5.74 (2.58 – 8.59) | 5.5 (3.17 – 8.5) | 3.75 (1.98 – 5.18) | 6.61 (3.99 – 10.99) | 4.07 (1.64 – 7) | 6.18 (3.27 – 8) | 3 (1.49 – 5.22) |
| **ARHD of COx (au)** | 0.17 (0.07 – 0.33) | 0.17 (0.08 – 0.32) | 0.17 (0.07 – 0.33) | 0.16 (0.07 – 0.31) | 0.16 (0.07 – 0.29) | 0.17 (0.06 – 0.33) | 0.17 (0.07 – 0.31) | 0.15 (0.05 – 0.28) |
| **ARHD of COx-a (au)** | 0.16 (0.07 – 0.31) | 0.17 (0.07 – 0.33) | 0.17 (0.07 – 0.32) | 0.16 (0.07 – 0.29) | 0.15 (0.07 – 0.28) | 0.16 (0.06 – 0.31) | 0.16 (0.06 – 0.29) | 0.15 (0.06 – 0.28) |
| **MAD of ARHD rSO2 (%)** | 3 (2.24 – 3.76) | 2.79 (2.23 – 4.18) | 3.46 (2.31 – 4.6) | 2.46 (2.28 – 2.64) | 5.03 (5.03 – 5.03) | 2.08 (1.57 – 3.83) | 2.99 (2.21 – 5.19) | 2.22 (1.5 – 2.36) |
| **MAD of ARHD COx (au)** | 0.16 (0.16 – 0.17) | 0.16 (0.13 – 0.18) | 0.17 (0.14 – 0.19) | 0.15 (0.15 – 0.15) | 0.15 (0.15 – 0.15) | 0.19 (0.14 – 0.2) | 0.16 (0.13 – 0.18) | 0.16 (0.13 – 0.16) |
| **MAD of ARHD COx-a (au)** | 0.16 (0.16 – 0.16) | 0.17 (0.14 – 0.18) | 0.17 (0.14 – 0.18) | 0.15 (0.15 – 0.15) | 0.15 (0.15 – 0.15) | 0.17 (0.14 – 0.18) | 0.16 (0.13 – 0.18) | 0.15 (0.13 – 0.16) |
| **Sub-grouped Physiologic Results for 1-Minute Data Resolution** | | | | | | | | |
| **ARHD of rSO2 (%)** | 7.66 (6.02 – 12.95) | 5.71 (2.66 – 8.66) | 5.49 (3.24 – 8.5) | 3.66 (2.01 – 5.24) | 6.58 (3.83 – 10.93) | 4.24 (1.63 – 6.89) | 6.25 (3.32 – 8.12) | 3 (1.48 – 5.23) |
| **ARHD of COx (au)** | 0.14 (0.06 – 0.28) | 0.17 (0.07 – 0.31) | 0.16 (0.08 – 0.3) | 0.15 (0.07 – 0.3) | 0.16 (0.07 – 0.28) | 0.2 (0.1 – 0.36) | 0.16 (0.08 – 0.32) | 0.15 (0.07 – 0.28) |
| **ARHD of COx-a (au)** | 0.13 (0.06 – 0.27) | 0.17 (0.07 – 0.32) | 0.16 (0.07 – 0.31) | 0.15 (0.07 – 0.28) | 0.15 (0.07 – 0.27) | 0.18 (0.09 – 0.33) | 0.17 (0.07 – 0.32) | 0.14 (0.06 – 0.28) |
| **MAD of ARHD rSO2 (%)** | 2.97 (2.11 – 3.84) | 2.76 (2.15 – 4.07) | 3.55 (2.37 – 4.64) | 2.34 (2.13 – 2.54) | 4.87 (4.87 – 4.87) | 1.95 (1.89 – 3.89) | 3.33 (2.08 – 5.21) | 2.15 (1.77 – 2.45) |
| **MAD of ARHD COx (au)** | 0.14 (0.14 – 0.14) | 0.16 (0.13 – 0.18) | 0.15 (0.12 – 0.18) | 0.15 (0.14 – 0.15) | 0.15 (0.15 – 0.15) | 0.18 (0.14 – 0.2) | 0.16 (0.13 – 0.18) | 0.15 (0.12 – 0.15) |
| **MAD of ARHD COx-a (au)** | 0.14 (0.14 – 0.14) | 0.17 (0.13 – 0.18) | 0.16 (0.13 – 0.17) | 0.15 (0.15 – 0.15) | 0.14 (0.14 – 0.14) | 0.17 (0.13 – 0.18) | 0.14 (0.13 – 0.17) | 0.14 (0.12 – 0.14) |
| **Sub-grouped Physiologic Results for 5-Minute Data Resolution** | | | | | | | | |
| **ARHD of rSO2 (%)** | 7.64 (6.07 – 12.67) | 5.75 (2.76 – 8.69) | 5.51 (3.26 – 8.55) | 3.63 (1.93 – 5.26) | 6.6 (3.82 – 10.91) | 4.25 (1.59 – 6.88) | 6.26 (3.36 – 8.08) | 3 (1.51 – 5.21) |
| **ARHD of COx (au)** | 0.13 (0.06 – 0.24) | 0.15 (0.07 – 0.27) | 0.15 (0.07 – 0.26) | 0.14 (0.06 – 0.26) | 0.14 (0.07 – 0.24) | 0.17 (0.08 – 0.31) | 0.15 (0.07 – 0.26) | 0.14 (0.06 – 0.25) |
| **ARHD of COx-a (au)** | 0.12 (0.06 – 0.24) | 0.15 (0.07 – 0.27) | 0.14 (0.06 – 0.27) | 0.13 (0.07 – 0.25) | 0.13 (0.06 – 0.24) | 0.16 (0.08 – 0.29) | 0.15 (0.07 – 0.27) | 0.13 (0.05 – 0.23) |
| **MAD of ARHD rSO2 (%)** | 2.89 (2.03 – 3.75) | 2.71 (2.01 – 4.01) | 3.49 (2.32 – 4.6) | 2.43 (2.21 – 2.65) | 4.84 (4.84 – 4.84) | 1.96 (1.93 – 3.93) | 3.34 (2.1 – 5.28) | 2.22 (1.75 – 2.45) |
| **MAD of ARHD COx (au)** | 0.12 (0.12 – 0.12) | 0.14 (0.11 – 0.15) | 0.13 (0.11 – 0.15) | 0.13 (0.12 – 0.13) | 0.12 (0.12 – 0.12) | 0.15 (0.12 – 0.15) | 0.13 (0.11 – 0.16) | 0.13 (0.1 – 0.13) |
| **MAD of ARHD COx-a (au)** | 0.12 (0.12 – 0.12) | 0.14 (0.11 – 0.15) | 0.14 (0.11 – 0.15) | 0.12 (0.12 – 0.12) | 0.12 (0.12 – 0.12) | 0.14 (0.11 – 0.15) | 0.12 (0.1 – 0.14) | 0.12 (0.11 – 0.12) |
| *ARHD, Absolute Regional Hemispheric Disparity; au, arbitrary units; COx, cerebral oximetry index with cerebral perfusion pressure; COx-a, cerebral oximetry index with arterial blood pressure; CT, computed tomography; DAI, diffuse axonal injury; EDH, epidural hematoma; IQR, interquartile range; MAD, median absolute deviation; rSO2, regional cerebral oxygen saturation; tSAH, traumatic subarachnoid hemorrhage; SDH, subdural hematoma; aSDH, acute subdural hematoma; TBI-GLR, traumatic brain injury patient group without bifrontal lobe pathology.* | | | | | | | | |

File S8p: Sub-grouped Absolute Regional Hemispheric Disparity Analysis using 10-Second, 1-Minute, and 5-Minute Data Resolutions for TBI-GL Population

| **Physiologic Variable** | **Median (IQR)** | | | | | | |
| --- | --- | --- | --- | --- | --- | --- | --- |
|  | **Age < 40 [n = 5]** | **Age 40 – 60 [n = 6]** | **Age > 60 [n = 4]** | **Males [n = 14]** | **Females [n = 1]** | **Focal Injury (aSDH, SDH, EDH, or Contusion) [n = 14]** | **Diffuse Injury (DAI or tSAH) [n = 1]** |
| **Sub-grouped Physiologic Results for 10-Second Data Resolution** | | | | | | | |
| **ARHD of rSO2 (%)** | 6.56 (4 – 9.31) | 9.5 (4.76 – 12.01) | 11.02 (8.51 – 13.82) | 6.98 (4.35 – 10.42) | 14 (12.54 – 15) | 8 (4.78 – 11.68) | 5.01 (2.99 – 8.01) |
| **ARHD of COx (au)** | 0.18 (0.08 – 0.32) | 0.18 (0.08 – 0.33) | 0.18 (0.08 – 0.35) | 0.18 (0.07 – 0.33) | 0.19 (0.09 – 0.32) | 0.18 (0.08 – 0.33) | 0.12 (0.06 – 0.24) |
| **ARHD of COx-a (au)** | 0.18 (0.08 – 0.32) | 0.18 (0.08 – 0.33) | 0.17 (0.07 – 0.33) | 0.18 (0.08 – 0.33) | 0.18 (0.09 – 0.33) | 0.18 (0.08 – 0.33) | 0.13 (0.06 – 0.25) |
| **MAD of ARHD rSO2 (%)** | 3.8 (3.26 – 4.74) | 4.08 (2.64 – 7.22) | 3.72 (3 – 6.88) | 4.11 (3.07 – 5.59) | 1.7 (1.7 – 1.7) | 4.11 (2.99 – 5.59) | 3.71 (3.71 – 3.71) |
| **MAD of ARHD COx (au)** | 0.17 (0.16 – 0.17) | 0.17 (0.16 – 0.19) | 0.2 (0.17 – 0.22) | 0.17 (0.16 – 0.2) | 0.16 (0.16 – 0.16) | 0.17 (0.16 – 0.2) | 0.12 (0.12 – 0.12) |
| **MAD of ARHD COx-a (au)** | 0.17 (0.16 – 0.17) | 0.17 (0.17 – 0.19) | 0.18 (0.18 – 0.19) | 0.17 (0.16 – 0.19) | 0.17 (0.17 – 0.17) | 0.17 (0.17 – 0.19) | 0.13 (0.13 – 0.13) |
| **Sub-grouped Physiologic Results for 1-Minute Data Resolution** | | | | | | | |
| **ARHD of rSO2 (%)** | 6.63 (3.84 – 9.15) | 9.49 (4.8 – 12.05) | 11 (8.58 – 13.88) | 6.92 (4.3 – 10.44) | 13.94 (12.56 – 14.92) | 7.92 (4.69 – 11.63) | 5.09 (2.84 – 8.14) |
| **ARHD of COx (au)** | 0.18 (0.08 – 0.32) | 0.18 (0.08 – 0.32) | 0.2 (0.09 – 0.37) | 0.19 (0.08 – 0.33) | 0.18 (0.09 – 0.31) | 0.19 (0.09 – 0.33) | 0.12 (0.05 – 0.23) |
| **ARHD of COx-a (au)** | 0.17 (0.08 – 0.32) | 0.18 (0.08 – 0.32) | 0.18 (0.08 – 0.34) | 0.18 (0.08 – 0.32) | 0.18 (0.09 – 0.32) | 0.18 (0.08 – 0.32) | 0.13 (0.06 – 0.24) |
| **MAD of ARHD rSO2 (%)** | 3.8 (3.2 – 4.84) | 4.11 (2.52 – 7.25) | 3.76 (3.27 – 6.66) | 3.98 (3.24 – 5.46) | 1.75 (1.75 – 1.75) | 3.98 (3.06 – 5.46) | 3.7 (3.7 – 3.7) |
| **MAD of ARHD COx (au)** | 0.17 (0.15 – 0.17) | 0.16 (0.15 – 0.19) | 0.18 (0.17 – 0.2) | 0.17 (0.16 – 0.19) | 0.15 (0.15 – 0.15) | 0.17 (0.16 – 0.19) | 0.12 (0.12 – 0.12) |
| **MAD of ARHD COx-a (au)** | 0.16 (0.15 – 0.17) | 0.16 (0.16 – 0.19) | 0.18 (0.16 – 0.19) | 0.17 (0.16 – 0.19) | 0.15 (0.15 – 0.15) | 0.17 (0.16 – 0.19) | 0.12 (0.12 – 0.12) |
| **Sub-grouped Physiologic Results for 5-Minute Data Resolution** | | | | | | | |
| **ARHD of rSO2 (%)** | 6.57 (3.84 – 9.1) | 9.42 (4.78 – 12) | 10.93 (8.5 – 13.87) | 6.94 (4.3 – 10.41) | 13.97 (12.56 – 14.86) | 7.89 (4.7 – 11.59) | 5.14 (2.79 – 8.19) |
| **ARHD of COx (au)** | 0.16 (0.07 – 0.28) | 0.15 (0.08 – 0.27) | 0.18 (0.09 – 0.3) | 0.16 (0.07 – 0.28) | 0.15 (0.08 – 0.27) | 0.16 (0.07 – 0.28) | 0.1 (0.05 – 0.19) |
| **ARHD of COx-a (au)** | 0.15 (0.07 – 0.26) | 0.16 (0.08 – 0.28) | 0.16 (0.07 – 0.3) | 0.15 (0.07 – 0.28) | 0.16 (0.08 – 0.29) | 0.16 (0.07 – 0.28) | 0.11 (0.05 – 0.2) |
| **MAD of ARHD rSO2 (%)** | 3.79 (3.19 – 4.88) | 4.13 (2.49 – 7.2) | 3.77 (3.35 – 6.59) | 3.95 (3.25 – 5.49) | 1.92 (1.92 – 1.92) | 3.95 (3.11 – 5.49) | 3.73 (3.73 – 3.73) |
| **MAD of ARHD COx (au)** | 0.14 (0.11 – 0.14) | 0.14 (0.13 – 0.17) | 0.16 (0.14 – 0.18) | 0.14 (0.14 – 0.17) | 0.13 (0.13 – 0.13) | 0.14 (0.14 – 0.17) | 0.1 (0.1 – 0.1) |
| **MAD of ARHD COx-a (au)** | 0.14 (0.13 – 0.15) | 0.14 (0.14 – 0.17) | 0.16 (0.14 – 0.17) | 0.15 (0.14 – 0.16) | 0.14 (0.14 – 0.14) | 0.15 (0.14 – 0.16) | 0.11 (0.11 – 0.11) |
| *ARHD, Absolute Regional Hemispheric Disparity; au, arbitrary units; COx, cerebral oximetry index with cerebral perfusion pressure; COx-a, cerebral oximetry index with arterial blood pressure; CT, computed tomography; DAI, diffuse axonal injury; EDH, epidural hematoma; IQR, interquartile range; MAD, median absolute deviation; rSO2, regional cerebral oxygen saturation; tSAH, traumatic subarachnoid hemorrhage; SDH, subdural hematoma; aSDH, acute subdural hematoma; TBI-GL, traumatic brain injury patient group without left frontal lobe pathology.* | | | | | | | |

| **Physiologic Variable** | **Median (IQR)** | | | | | |
| --- | --- | --- | --- | --- | --- | --- |
|  | **Marshall CT V [n = 8]** | **Marshall CT IV [n = 5]** | **Marshall CT III [n = 2]** | **Rotterdam CT 6 [n = 1]** | **Rotterdam CT 5 [n = 7]** | **Rotterdam CT 4 [n = 5]** |
| **Sub-grouped Physiologic Results for 10-Second Data Resolution** | | | | | | |
| **ARHD of rSO2 (%)** | 11.52 (7.51 – 13.98) | 7 (4.87 – 10.83) | 5.26 (3.49 – 7.67) | 10 (4 – 12.95) | 13.04 (11.03 – 15.12) | 6.95 (4.7 – 10.01) |
| **ARHD of COx (au)** | 0.19 (0.09 – 0.33) | 0.18 (0.08 – 0.33) | 0.13 (0.05 – 0.24) | 0.16 (0.07 – 0.31) | 0.19 (0.08 – 0.33) | 0.18 (0.08 – 0.32) |
| **ARHD of COx-a (au)** | 0.19 (0.09 – 0.33) | 0.18 (0.08 – 0.32) | 0.13 (0.05 – 0.24) | 0.18 (0.08 – 0.33) | 0.18 (0.08 – 0.34) | 0.17 (0.08 – 0.3) |
| **MAD of ARHD rSO2 (%)** | 4.12 (2.83 – 7.95) | 4.43 (3.26 – 4.74) | 2.99 (2.64 – 3.35) | 5.87 (5.87 – 5.87) | 4.45 (3.4 – 11.17) | 3.26 (2.98 – 4.43) |
| **MAD of ARHD COx (au)** | 0.17 (0.16 – 0.21) | 0.17 (0.17 – 0.19) | 0.12 (0.12 – 0.13) | 0.16 (0.16 – 0.16) | 0.19 (0.17 – 0.21) | 0.17 (0.16 – 0.17) |
| **MAD of ARHD COx-a (au)** | 0.18 (0.17 – 0.19) | 0.17 (0.16 – 0.18) | 0.12 (0.12 – 0.13) | 0.17 (0.17 – 0.17) | 0.19 (0.18 – 0.2) | 0.16 (0.16 – 0.17) |
| **Sub-grouped Physiologic Results for 1-Minute Data Resolution** | | | | | | |
| **ARHD of rSO2 (%)** | 11.59 (7.59 – 13.8) | 7 (4.79 – 10.72) | 5.32 (3.34 – 7.76) | 10.01 (4 – 12.68) | 13.17 (11.17 – 15.21) | 6.84 (4.6 – 10.15) |
| **ARHD of COx (au)** | 0.19 (0.09 – 0.34) | 0.19 (0.09 – 0.34) | 0.12 (0.06 – 0.23) | 0.16 (0.07 – 0.29) | 0.2 (0.09 – 0.37) | 0.18 (0.08 – 0.32) |
| **ARHD of COx-a (au)** | 0.18 (0.08 – 0.32) | 0.18 (0.08 – 0.32) | 0.12 (0.06 – 0.23) | 0.17 (0.08 – 0.32) | 0.18 (0.08 – 0.37) | 0.17 (0.07 – 0.31) |
| **MAD of ARHD rSO2 (%)** | 4.16 (2.79 – 7.79) | 4.17 (3.36 – 4.84) | 3.14 (2.87 – 3.42) | 5.67 (5.67 – 5.67) | 4.53 (3.41 – 11.15) | 3.36 (3.2 – 4.17) |
| **MAD of ARHD COx (au)** | 0.17 (0.16 – 0.19) | 0.17 (0.17 – 0.19) | 0.12 (0.12 – 0.12) | 0.15 (0.15 – 0.15) | 0.19 (0.17 – 0.2) | 0.17 (0.16 – 0.17) |
| **MAD of ARHD COx-a (au)** | 0.17 (0.16 – 0.2) | 0.17 (0.16 – 0.18) | 0.12 (0.11 – 0.12) | 0.16 (0.16 – 0.16) | 0.19 (0.16 – 0.2) | 0.16 (0.15 – 0.17) |
| **Sub-grouped Physiologic Results for 5-Minute Data Resolution** | | | | | | |
| **ARHD of rSO2 (%)** | 11.54 (7.5 – 13.68) | 7.01 (4.75 – 10.69) | 5.35 (3.32 – 7.8) | 9.98 (3.95 – 12.49) | 13.1 (11.04 – 15.23) | 6.86 (4.66 – 10.12) |
| **ARHD of COx (au)** | 0.16 (0.08 – 0.28) | 0.16 (0.07 – 0.28) | 0.11 (0.05 – 0.19) | 0.12 (0.06 – 0.24) | 0.18 (0.08 – 0.32) | 0.16 (0.07 – 0.28) |
| **ARHD of COx-a (au)** | 0.16 (0.08 – 0.28) | 0.16 (0.07 – 0.28) | 0.11 (0.05 – 0.19) | 0.14 (0.06 – 0.26) | 0.17 (0.08 – 0.33) | 0.15 (0.07 – 0.26) |
| **MAD of ARHD rSO2 (%)** | 4.16 (2.83 – 7.79) | 4.1 (3.44 – 4.88) | 3.18 (2.9 – 3.45) | 5.69 (5.69 – 5.69) | 4.54 (3.44 – 11.07) | 3.44 (3.19 – 4.1) |
| **MAD of ARHD COx (au)** | 0.15 (0.13 – 0.17) | 0.14 (0.14 – 0.18) | 0.1 (0.1 – 0.1) | 0.11 (0.11 – 0.11) | 0.17 (0.14 – 0.18) | 0.14 (0.14 – 0.14) |
| **MAD of ARHD COx-a (au)** | 0.15 (0.14 – 0.17) | 0.15 (0.14 – 0.16) | 0.1 (0.1 – 0.1) | 0.14 (0.14 – 0.14) | 0.16 (0.14 – 0.17) | 0.14 (0.13 – 0.15) |
| *ARHD, Absolute Regional Hemispheric Disparity; au, arbitrary units; COx, cerebral oximetry index with cerebral perfusion pressure; COx-a, cerebral oximetry index with arterial blood pressure; CT, computed tomography; DAI, diffuse axonal injury; EDH, epidural hematoma; IQR, interquartile range; MAD, median absolute deviation; rSO2, regional cerebral oxygen saturation; tSAH, traumatic subarachnoid hemorrhage; SDH, subdural hematoma; aSDH, acute subdural hematoma; TBI-GL, traumatic brain injury patient group without left frontal lobe pathology.* | | | | | | |

| **Physiologic Variable** | **Median (IQR)** | | | | | |
| --- | --- | --- | --- | --- | --- | --- |
|  | **Rotterdam CT 3 [n = 2]** | **Propofol [n = 5]** | **Fentanyl + Propofol [n = 3]** | **Fentanyl + Ketamine + Propofol [n = 1]** | **Fentanyl + Midazolam + Propofol [n = 4]** | **Fentanyl + Ketamine + Midazolam + Propofol [n = 2]** |
| **Sub-grouped Physiologic Results for 10-Second Data Resolution** | | | | | | |
| **ARHD of rSO2 (%)** | 4.28 (2.63 – 7) | 6.56 (3.38 – 9.01) | 7 (4 – 12.52) | 13 (6.52 – 17) | **16.46 (9 – 21.15)** | 6.48 (3.94 – 9.66) |
| **ARHD of COx (au)** | 0.16 (0.07 – 0.28) | 0.19 (0.09 – 0.33) | 0.16 (0.07 – 0.31) | 0.18 (0.07 – 0.33) | 0.16 (0.06 – 0.32) | 0.18 (0.08 – 0.33) |
| **ARHD of COx-a (au)** | 0.16 (0.08 – 0.29) | 0.2 (0.09 – 0.33) | 0.18 (0.08 – 0.33) | 0.18 (0.07 – 0.34) | 0.15 (0.05 – 0.3) | 0.18 (0.08 – 0.31) |
| **MAD of ARHD rSO2 (%)** | 2.99 (2.64 – 3.35) | 3.01 (2.28 – 3.8) | 4.43 (4.07 – 5.15) | 8.14 (8.14 – 8.14) | 8.59 (2.81 – 22.51) | 4 (3.63 – 4.37) |
| **MAD of ARHD COx (au)** | 0.14 (0.13 – 0.16) | 0.17 (0.17 – 0.18) | 0.16 (0.14 – 0.19) | 0.19 (0.19 – 0.19) | 0.19 (0.15 – 0.23) | 0.17 (0.17 – 0.17) |
| **MAD of ARHD COx-a (au)** | 0.15 (0.14 – 0.16) | 0.17 (0.17 – 0.19) | 0.17 (0.15 – 0.17) | 0.19 (0.19 – 0.19) | 0.17 (0.15 – 0.21) | 0.16 (0.16 – 0.17) |
| **Sub-grouped Physiologic Results for 1-Minute Data Resolution** | | | | | | |
| **ARHD of rSO2 (%)** | 4.29 (2.6 – 6.99) | 6.63 (3.35 – 9.17) | 7 (4 – 12.55) | 12.92 (6.58 – 17) | **16.36 (8.96 – 21.06)** | 6.42 (3.91 – 9.65) |
| **ARHD of COx (au)** | 0.15 (0.07 – 0.28) | 0.18 (0.09 – 0.32) | 0.16 (0.07 – 0.29) | 0.2 (0.09 – 0.37) | 0.18 (0.07 – 0.33) | 0.19 (0.09 – 0.33) |
| **ARHD of COx-a (au)** | 0.16 (0.07 – 0.28) | 0.19 (0.09 – 0.32) | 0.17 (0.07 – 0.32) | 0.2 (0.08 – 0.37) | 0.17 (0.07 – 0.31) | 0.18 (0.08 – 0.31) |
| **MAD of ARHD rSO2 (%)** | 2.91 (2.52 – 3.31) | 3.01 (2.12 – 3.8) | 4.17 (3.94 – 4.92) | 8.16 (8.16 – 8.16) | 8.75 (3.17 – 22.19) | 4.02 (3.61 – 4.43) |
| **MAD of ARHD COx (au)** | 0.14 (0.13 – 0.15) | 0.16 (0.16 – 0.17) | 0.15 (0.13 – 0.19) | 0.19 (0.19 – 0.19) | 0.17 (0.15 – 0.19) | 0.17 (0.17 – 0.17) |
| **MAD of ARHD COx-a (au)** | 0.14 (0.13 – 0.16) | 0.17 (0.16 – 0.2) | 0.16 (0.14 – 0.17) | 0.19 (0.19 – 0.19) | 0.16 (0.15 – 0.18) | 0.16 (0.16 – 0.16) |
| **Sub-grouped Physiologic Results for 5-Minute Data Resolution** | | | | | | |
| **ARHD of rSO2 (%)** | 4.25 (2.62 – 7.03) | 6.57 (3.37 – 9.13) | 7.01 (3.95 – 12.49) | 12.83 (6.55 – 17) | **16.27 (8.98 – 21.03)** | 6.4 (3.9 – 9.61) |
| **ARHD of COx (au)** | 0.13 (0.06 – 0.23) | 0.15 (0.08 – 0.28) | 0.12 (0.06 – 0.24) | 0.19 (0.08 – 0.34) | 0.16 (0.07 – 0.29) | 0.16 (0.07 – 0.28) |
| **ARHD of COx-a (au)** | 0.14 (0.06 – 0.24) | 0.16 (0.08 – 0.29) | 0.14 (0.06 – 0.26) | 0.18 (0.08 – 0.33) | 0.15 (0.06 – 0.27) | 0.15 (0.07 – 0.27) |
| **MAD of ARHD rSO2 (%)** | 2.9 (2.49 – 3.31) | 3.08 (2.07 – 3.79) | 4.1 (3.91 – 4.9) | 8.08 (8.08 – 8.08) | 8.75 (3.24 – 21.66) | 4.04 (3.61 – 4.46) |
| **MAD of ARHD COx (au)** | 0.12 (0.11 – 0.13) | 0.14 (0.13 – 0.15) | 0.11 (0.11 – 0.16) | 0.18 (0.18 – 0.18) | 0.15 (0.13 – 0.18) | 0.14 (0.14 – 0.14) |
| **MAD of ARHD COx-a (au)** | 0.13 (0.12 – 0.14) | 0.15 (0.14 – 0.16) | 0.14 (0.12 – 0.15) | 0.17 (0.17 – 0.17) | 0.14 (0.13 – 0.15) | 0.14 (0.14 – 0.14) |
| *ARHD, Absolute Regional Hemispheric Disparity; au, arbitrary units; COx, cerebral oximetry index with cerebral perfusion pressure; COx-a, cerebral oximetry index with arterial blood pressure; CT, computed tomography; DAI, diffuse axonal injury; EDH, epidural hematoma; IQR, interquartile range; MAD, median absolute deviation; rSO2, regional cerebral oxygen saturation; tSAH, traumatic subarachnoid hemorrhage; SDH, subdural hematoma; aSDH, acute subdural hematoma; TBI-GL, traumatic brain injury patient group without left frontal lobe pathology.* | | | | | | |

File S8q: Sub-grouped Absolute Regional Hemispheric Disparity Analysis using 10-Second, 1-Minute, and 5-Minute Data Resolutions for TBI-GR Population

| **Physiologic Variable** | **Median (IQR)** | | | | | | | |
| --- | --- | --- | --- | --- | --- | --- | --- | --- |
|  | **Age < 40 [n = 4]** | **Age 40 – 60 [n = 3]** | **Age > 60 [n = 4]** | **Males [n = 7]** | **Females [n = 4]** | **Focal Injury (aSDH, SDH, EDH, or Contusion) [n = 10]** | **Diffuse Injury (DAI or tSAH) [n = 1]** | **Marshall CT V [n = 8]** |
| **Sub-grouped Physiologic Results for 10-Second Data Resolution** | | | | | | | | |
| **ARHD of rSO2 (%)** | 4.74 (2.51 – 6.51) | 9 (6.49 – 11.5) | 6.58 (4 – 8.5) | 6.16 (4 – 8.77) | 4.02 (2.32 – 6.13) | 6.12 (3.82 – 8.89) | 2.01 (1 – 3.26) | 6.12 (3.82 – 8.89) |
| **ARHD of COx (au)** | 0.19 (0.08 – 0.33) | 0.17 (0.07 – 0.35) | 0.19 (0.09 – 0.35) | 0.18 (0.08 – 0.35) | 0.16 (0.07 – 0.34) | 0.17 (0.07 – 0.33) | 0.21 (0.09 – 0.36) | 0.16 (0.07 – 0.32) |
| **ARHD of COx-a (au)** | 0.17 (0.07 – 0.31) | 0.17 (0.07 – 0.32) | 0.19 (0.08 – 0.33) | 0.17 (0.07 – 0.32) | 0.16 (0.06 – 0.31) | 0.17 (0.07 – 0.31) | 0.18 (0.08 – 0.32) | 0.16 (0.06 – 0.31) |
| **MAD of ARHD rSO2 (%)** | 3.3 (2.03 – 4.4) | 3.71 (3.62 – 3.81) | 2.7 (1.48 – 4.11) | 3.71 (2.88 – 4.15) | 2.71 (1.49 – 4.04) | 3.81 (2.54 – 4.27) | 1.5 (1.5 – 1.5) | 3.92 (3.03 – 4.4) |
| **MAD of ARHD COx (au)** | 0.18 (0.17 – 0.21) | 0.18 (0.18 – 0.2) | 0.18 (0.16 – 0.21) | 0.18 (0.17 – 0.22) | 0.18 (0.17 – 0.2) | 0.18 (0.17 – 0.21) | 0.19 (0.19 – 0.19) | 0.17 (0.17 – 0.2) |
| **MAD of ARHD COx-a (au)** | 0.17 (0.17 – 0.17) | 0.17 (0.17 – 0.18) | 0.17 (0.15 – 0.2) | 0.17 (0.17 – 0.18) | 0.16 (0.16 – 0.17) | 0.17 (0.16 – 0.19) | 0.16 (0.16 – 0.16) | 0.17 (0.16 – 0.19) |
| **Sub-grouped Physiologic Results for 1-Minute Data Resolution** | | | | | | | | |
| **ARHD of rSO2 (%)** | 4.72 (2.56 – 6.49) | 9.19 (6.46 – 11.52) | 6.58 (4 – 8.46) | 6.25 (3.92 – 8.73) | 4.21 (2.34 – 6.1) | 6.25 (3.79 – 8.86) | 2.18 (1.02 – 3.2) | 6.25 (3.79 – 8.86) |
| **ARHD of COx (au)** | 0.19 (0.09 – 0.34) | 0.19 (0.08 – 0.34) | 0.19 (0.09 – 0.34) | 0.19 (0.08 – 0.34) | 0.19 (0.08 – 0.33) | 0.18 (0.08 – 0.33) | 0.21 (0.1 – 0.35) | 0.17 (0.08 – 0.32) |
| **ARHD of COx-a (au)** | 0.17 (0.07 – 0.31) | 0.17 (0.08 – 0.31) | 0.18 (0.08 – 0.33) | 0.17 (0.08 – 0.31) | 0.16 (0.07 – 0.3) | 0.17 (0.08 – 0.31) | 0.17 (0.07 – 0.31) | 0.17 (0.08 – 0.31) |
| **MAD of ARHD rSO2 (%)** | 3.04 (1.91 – 4.08) | 3.76 (3.63 – 3.78) | 2.79 (1.49 – 4.21) | 3.76 (2.75 – 4.08) | 2.71 (1.59 – 3.87) | 3.78 (2.37 – 4.09) | 1.63 (1.63 – 1.63) | 3.93 (2.99 – 4.09) |
| **MAD of ARHD COx (au)** | 0.17 (0.16 – 0.21) | 0.18 (0.17 – 0.2) | 0.18 (0.16 – 0.21) | 0.18 (0.16 – 0.22) | 0.18 (0.16 – 0.2) | 0.17 (0.16 – 0.22) | 0.19 (0.19 – 0.19) | 0.16 (0.16 – 0.2) |
| **MAD of ARHD COx-a (au)** | 0.16 (0.16 – 0.16) | 0.16 (0.16 – 0.18) | 0.17 (0.15 – 0.19) | 0.16 (0.16 – 0.18) | 0.16 (0.15 – 0.17) | 0.16 (0.15 – 0.18) | 0.16 (0.16 – 0.16) | 0.16 (0.15 – 0.19) |
| **Sub-grouped Physiologic Results for 5-Minute Data Resolution** | | | | | | | | |
| **ARHD of rSO2 (%)** | 4.69 (2.59 – 6.47) | 9.26 (6.42 – 11.47) | 6.53 (3.99 – 8.4) | 6.28 (3.93 – 8.67) | 4.24 (2.37 – 6.13) | 6.27 (3.79 – 8.8) | 2.21 (1.09 – 3.23) | 6.27 (3.79 – 8.8) |
| **ARHD of COx (au)** | 0.17 (0.08 – 0.29) | 0.15 (0.07 – 0.3) | 0.16 (0.07 – 0.3) | 0.16 (0.07 – 0.3) | 0.16 (0.08 – 0.29) | 0.16 (0.07 – 0.29) | 0.18 (0.09 – 0.31) | 0.15 (0.07 – 0.28) |
| **ARHD of COx-a (au)** | 0.14 (0.07 – 0.26) | 0.15 (0.07 – 0.26) | 0.16 (0.08 – 0.29) | 0.15 (0.07 – 0.26) | 0.14 (0.07 – 0.26) | 0.15 (0.07 – 0.26) | 0.14 (0.07 – 0.27) | 0.15 (0.07 – 0.27) |
| **MAD of ARHD rSO2 (%)** | 2.97 (1.86 – 4.02) | 3.83 (3.67 – 3.86) | 2.78 (1.44 – 4.22) | 3.83 (2.72 – 4.05) | 2.75 (1.56 – 3.93) | 3.86 (2.33 – 4.05) | 1.62 (1.62 – 1.62) | 3.95 (2.99 – 4.07) |
| **MAD of ARHD COx (au)** | 0.15 (0.14 – 0.17) | 0.15 (0.14 – 0.17) | 0.15 (0.14 – 0.17) | 0.15 (0.14 – 0.18) | 0.15 (0.14 – 0.16) | 0.15 (0.14 – 0.19) | 0.15 (0.15 – 0.15) | 0.15 (0.14 – 0.17) |
| **MAD of ARHD COx-a (au)** | 0.13 (0.12 – 0.14) | 0.13 (0.13 – 0.16) | 0.15 (0.14 – 0.16) | 0.14 (0.13 – 0.15) | 0.13 (0.12 – 0.14) | 0.14 (0.13 – 0.16) | 0.12 (0.12 – 0.12) | 0.14 (0.13 – 0.16) |
| *ARHD, Absolute Regional Hemispheric Disparity; au, arbitrary units; COx, cerebral oximetry index with cerebral perfusion pressure; COx-a, cerebral oximetry index with arterial blood pressure; CT, computed tomography; DAI, diffuse axonal injury; EDH, epidural hematoma; IQR, interquartile range; MAD, median absolute deviation; rSO2, regional cerebral oxygen saturation; tSAH, traumatic subarachnoid hemorrhage; SDH, subdural hematoma; aSDH, acute subdural hematoma; TBI-GR, traumatic brain injury patient group without right frontal lobe pathology.* | | | | | | | | |

| **Physiologic Variable** | **Median (IQR)** | | | | | | | | |
| --- | --- | --- | --- | --- | --- | --- | --- | --- | --- |
|  | **Marshall CT IV [n = 2]** | **Marshall CT II [n = 1]** | **Rotterdam CT 6 [n = 5]** | **Rotterdam CT 5 [n = 1]** | **Rotterdam CT 4 [n = 4]** | **Rotterdam CT 2 [n = 1]** | **Propofol [n = 3]** | **Fentanyl + Propofol [n = 6]** | **Fentanyl + Midazolam + Propofol [n = 2]** |
| **Sub-grouped Physiologic Results for 10-Second Data Resolution** | | | | | | | | | |
| **ARHD of rSO2 (%)** | 6.24 (4.25 – 8.25) | 2.01 (1 – 3.26) | 6.08 (3.63 – 9) | 7 (5.99 – 8) | 6.24 (4.25 – 8.25) | 2.01 (1 – 3.26) | 6.16 (3 – 9) | 6.51 (4.81 – 8.5) | 4.78 (3.01 – 6.88) |
| **ARHD of COx (au)** | 0.23 (0.1 – 0.42) | 0.21 (0.09 – 0.36) | 0.16 (0.05 – 0.3) | 0.22 (0.1 – 0.38) | 0.23 (0.1 – 0.42) | 0.21 (0.09 – 0.36) | 0.15 (0.05 – 0.3) | 0.2 (0.09 – 0.35) | 0.23 (0.1 – 0.41) |
| **ARHD of COx-a (au)** | 0.18 (0.08 – 0.32) | 0.18 (0.08 – 0.32) | 0.15 (0.05 – 0.3) | 0.22 (0.1 – 0.37) | 0.18 (0.08 – 0.32) | 0.18 (0.08 – 0.32) | 0.14 (0.05 – 0.3) | 0.17 (0.08 – 0.31) | 0.18 (0.08 – 0.32) |
| **MAD of ARHD rSO2 (%)** | 2.96 (2.58 – 3.33) | 1.5 (1.5 – 1.5) | 4.39 (3.91 – 4.42) | 1.48 (1.48 – 1.48) | 2.96 (2.03 – 3.76) | 1.5 (1.5 – 1.5) | 4.39 (4.15 – 4.53) | 2.6 (1.49 – 3.87) | 2.88 (2.54 – 3.21) |
[truncated: 354,970 more chars]
